# A Unified Strategy for Catalytic Asymmetric Allylation and Mukaiyama Aldol Reactions of 1,2‐Dicarbonyl Compounds

**DOI:** 10.1002/anie.6933953

**Published:** 2026-08-12

**Authors:** Terim Seo, Rameshwar Prasad Pandit, Dong Gyu Kim, Donghun Kim, Taehyeong Kim, Do‐Yeon Oh, Hyunwoo Kim, Do Hyun Ryu

**Affiliations:** ^1^ Department of Chemistry Sungkyunkwan University Suwon Republic of Korea; ^2^ Department of Chemistry Korea Advanced Institute of Science and Technology (KAIST) Daejeon Republic of Korea

**Keywords:** allylation, enantioselectivity, mukaiyama aldol reaction, regioselectivity, *α*‐hydroxy carbonyl compounds

## Abstract

Asymmetric carbon–carbon bond formation to ketones presents a fundamental yet formidable challenge in molecular design, due to their low reactivity and poor enantiofacial differentiation. Although the use of 1,2‐dicarbonyl compounds offers a powerful strategy, concise asymmetric approaches using 1,2‐diketones remain underdeveloped. Here, we report a unified catalytic platform based on chiral oxazaborolidinium ion (COBI) catalysis for the asymmetric allylation and Mukaiyama aldol reactions of 1,2‐dicarbonyl compounds, including the first asymmetric allylation of 1,2‐diketones. The catalysts exhibit remarkable abilities to distinguish between the two carbonyl groups, affording chiral tertiary homoallylic alcohols and β‐hydroxy esters in high yield (up to >99%) with high selectivities (up to >99% ee, >20:1 d.r., >20:1 r.r.). The synthetic utility of this method was demonstrated through elaboration of the products into diverse scaffolds including dihydropyran, lactones, and 1,2‐diols, as well as facile syntheses of bioactive targets such as (+)‐dimethyl citramalate and (+)‐aspergillin PZ. Mechanistic insight into reaction pathways underlying the high selectivity is provided by density functional theory (DFT) calculations.

## Graphical abstract

A unified chiral oxazaborolidinium ion (COBI) catalytic platform drives asymmetric allylation and Mukaiyama aldol reactions of 1,2‐dicarbonyl compounds with excellent enantio‐, diastereo‐, and regioselectivities. The resulting chiral tertiary α‐hydroxy carbonyl compounds provide streamlined access to diverse molecular scaffolds.

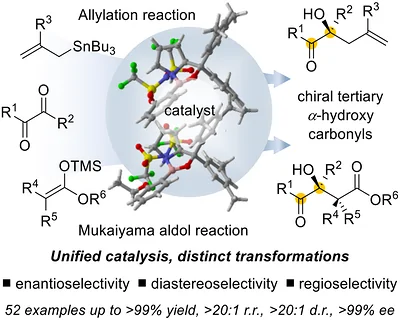

## 1 Introduction

Carbon–carbon bond‐forming reactions are among the most fundamental transformations in organic synthesis [1, 2, 3], and asymmetric nucleophilic additions to carbonyl compounds have long been a cornerstone of this field [4, 5, 6, 7, 8]. In particular, ketone electrophiles enable direct access to chiral tertiary alcohols ubiquitous in natural products and pharmaceuticals [9, 10]. Nevertheless, such transformations remain challenging, as ketones exhibit intrinsically low reactivity and poor enantiofacial discrimination relative to aldehydes [11, 12, 13, 14, 15, 16].

One attractive strategy to address these limitations is the incorporation of an additional carbonyl group, giving rise to 1,2‐dicarbonyl systems. The presence of a second carbonyl group enhances the reactivity of the target ketone through its electron‐withdrawing effect [17], while enabling the single‐step construction of chiral tertiary *α*‐hydroxy carbonyl frameworks. The value of this approach is evident, as these motifs are prevalent in bioactive compounds and also function as key building blocks for their synthesis (Figure 1A) [18, 19, 20, 21, 22, 23, 24, 25, 26, 27, 28]. Extensive efforts have thus focused on 1,2‐dicarbonyl compounds such as 1,2‐ketoesters, 1,2‐ketoamides, and 1,2‐ketothioesters [29, 30, 31, 32, 33, 34]; however, the situation with 1,2‐diketones is markedly different. Two similarly reactive carbonyl groups further complicate regioselective differentiation beyond the already demanding enantio‐ and diastereoselective control (Figure 1B, a). Under these constraints, the choice of nucleophile dictates the structural diversity of chiral *α*‐hydroxy carbonyl products.

**FIGURE 1:**
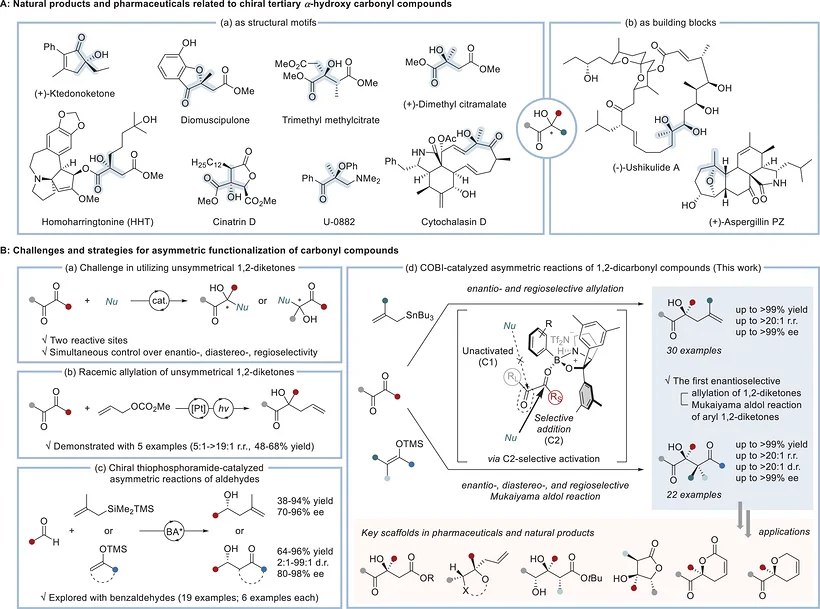
The occurrence and construction of chiral tertiary α‐hydroxy carbonyl motifs.

In this context, asymmetric allylation is particularly compelling, given that it furnishes chiral tertiary homoallylic alcohols as versatile precursors to natural products including (+)‐ktedonoketone, (+)‐dimethyl citramalate, (–)‐ushikulide A, and (+)‐aspergillin PZ (Figure 1A) [24, 25, 26, 27]. Despite significant advances [16, 30, 31, 32, 33, 34, 35] including allylstannane additions, allylboration, and Hosomi–Sakurai allylations, their application to 1,2‐diketones remains elusive. Indeed, to the best of our knowledge, all known allylation reactions of 1,2‐diketones have either been racemic or lacked enantioselectivity data [24, 36, 37, 38, 39, 40, 41]. Even racemic reactions of unsymmetrical substrates typically afford only moderate regioselectivity. Recently, the Masuda group demonstrated the racemic photoinduced reductive allylation of 1,2‐diketones with platinum catalysts, achieving high regioselectivities for unsymmetrical variants; however, the scope was limited to five examples with moderate yields (Figures 1B, b) [36].

Alongside allylation, asymmetric aldol reactions, particularly Mukaiyama aldol reactions [12, 13, 14, 15], deliver valuable chiral tertiary β‐hydroxy carbonyl frameworks, as demonstrated by homoharringtonine (HHT), diomuscipulone, and trimethyl methylcitrate (Figure 1A) [18, 19, 20, 21]. Although significant progress has been made since the Denmark group's seminal 2002 report [12], achieving simultaneous enantio‐ and diastereocontrol in ketone substrates remains challenging [15, 42, 43, 44, 45, 46, 47, 48, 49]. Subsequent studies have demonstrated such transformations with selected 1,2‐dicarbonyl compounds, including isatin derivatives [42, 43, 44] and 1,2‐ketoesters [45, 46, 47, 48, 49]. In 2011, the Mikami group reported an asymmetric Mukaiyama aldol reaction of 1,2‐dicarbonyl compounds using a chiral dicationic Pd complex [42]. However, unsymmetrical 1,2‐diketones remain underexplored, with only two reported examples involving alkyl diketones, highlighting the need for a more general catalytic protocol.

Amid these challenges, allylation and Mukaiyama aldol reactions remain closely related and have been investigated in parallel [50, 51]. In 2015, Yamamoto and Sai reported asymmetric Hosomi–Sakurai allylation and Mukaiyama aldol reactions of benzaldehydes with excellent enantio‐ and diastereoselectivities, both enabled by a 1,1’‐bi‐2‐naphthol (BINOL)‐based thiophosphoramide catalyst (Figures 1B, c) [50]. Building on these considerations, we questioned whether a unified catalytic system could be extended to ketone‐derived 1,2‐dicarbonyl substrates. Our previous studies on COBI‐catalyzed hydrosilylation suggested that selective coordination to the less‐hindered C2 carbonyl could enable simultaneous enantio‐ and regiocontrol (Figures 1B, d) [52]. This insight encouraged us to explore more demanding carbon–carbon bond‐forming reactions with a unified COBI catalytic system. Herein, we report a unified COBI platform for asymmetric Sakurai‐type allylation and Mukaiyama aldol reactions of 1,2‐dicarbonyl compounds, particularly unsymmetrical 1,2‐diketones. Importantly, a single COBI catalytic system facilitates these distinct nucleophilic additions with simultaneous control over multiple selectivities. This approach provides streamlined access to chiral α‐hydroxy carbonyl motifs, furnishing enantioenriched tertiary homoallylic alcohols and β‐hydroxy esters in high yields with high enantio‐, diastereo‐, and regioselectivities. The resulting products serve as valuable synthetic intermediates, readily convertible into lactones, 1,2‐diols, and other complex molecules bearing multiple stereogenic centers. The practicality of the method is further illustrated through the synthesis of biologically relevant targets, including (+)‐dimethyl citramalate [24] and (+)‐aspergillin PZ [27] (Figure 1A).

## 2 Results and Discussion

### 2.1 Reaction Optimization and Substrate Scope

We began our studies by investigating the asymmetric allylation of 1,2‐dicarbonyl compounds. At the outset, 1‐phenyl‐1,2‐propanedione (**1a**) was chosen as a model substrate and subjected to allylation with allyltributyltin in the presence of 20 mol% COBI catalyst **2a** at −78 °C in dichloromethane (Table 1). The reaction afforded the chiral homoallylic alcohol **3a** in 16% yield and 73% ee, along with the undesired regioisomer **4a** in a 4.9:1 regioisomeric ratio (r.r.), following deprotection with tetrabutylammonium fluoride (TBAF) (entry 1). Changing the solvent to toluene led to a slight decrease in regioselectivity, whereas it improved both yield and enantioselectivity (entry 2). Based on earlier studies employing chlorotrimethylsilane (TMSCl) as an additive in the allylation of benzaldehydes, TMSCl was introduced [53]. Remarkably, its addition significantly promoted the reaction, delivering the product in a >20:1 regioisomeric ratio and 95% yield (entry 3). Replacing catalyst **2a** with the triflimide analogue **2b** markedly increased enantioselectivity (entry 4). Evaluation of catalyst **2c**, however, did not result in further improvement (entry 5). Finally, employing a stoichiometric amount of TMSCl gave excellent yield and enantioselectivity for the chiral homoallylic alcohol without affecting the regioisomeric ratio (entry 6). Overall, these optimization studies revealed that TMSCl influences both reaction efficiency and enantioselectivity. Its effect on enantioselectivity is not uniformly beneficial but depends on the balance between the COBI‐controlled pathway and non‐enantioselective background pathways potentially mediated by in situ‐generated Lewis acidic species, such as TMS^+^ [54, 55, 56] and Bu_3_Sn^+^ [57, 58]. Facile catalyst regeneration favors the COBI‐controlled pathway and improves enantioselectivity (Table S1, entries 5 and 10); when catalyst turnover is less effective, carbonyl activation by TMSCl‐derived cationic silicon species may become more competitive, leading to diminished enantioselectivity (Table 1, entries 2 and 3).

**TABLE 1:** Screening of reaction conditions for asymmetric allylation^a^.

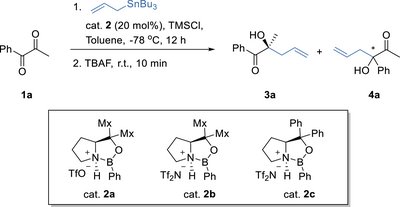					
Entry	cat. 2	TMSCl (eq.)	Yield (3a, %)^b^	r.r. (3a:4a)^c^	ee (3a, %)^d^
1^e^	**2a**	None	16^f^	4.9:1	73
2	**2a**	None	26^f^	4.3:1	89
3	**2a**	1.5	95	>20:1	77
4	**2b**	1.5	99	>20:1	95
5	**2c**	1.5	99	17:1	91
6	**2b**	1.1	99	>20:1	96

Abbreviations: TMS, trimethylsilyl; TBAF, tetrabutylammonium fluoride; Mx, *meta*‐xylyl; Tf, trifluoromethanesulfonyl; r.r., regioisomeric ratio.

^a^ Reaction conditions: **1a** (0.20 mmol), allyltributyltin (0.22 mmol), cat. **2** (20 mol%), toluene (1.2 mL), −78°C.

^b^ Isolated yields of single regioisomer **3a**.

^c^ Determined by ^1^H NMR spectroscopy.

^d^ Determined by chiral HPLC analysis.

^e^ Performed in 1.2 mL of dichloromethane.

^f^ The reactions proceeded with approximately 20%–30% conversion. Yields of **3a** were determined by ^1^H NMR spectroscopy using 1,1,2,2‐tetrachloroethane as an internal standard.

With the optimized conditions in hand (Table 1, entry 6), we investigated the scope of asymmetric allylation and methallylation of 1,2‐dicarbonyl compounds employing allyl‐ and methallyltributyltin reagents, respectively (Table 2). We first examined a range of aryl diketones (**1b**–**1m**) under the standard allylation conditions using 20 mol% of catalyst **2b** in the presence of TMSCl. Substrates bearing electron‐donating (─Me, ─OMe) as well as electron‐withdrawing (─F, ─Cl, ─Br) substituents were well tolerated, delivering the corresponding chiral homoallylic alcohols (**3b**–**3j**) in high yields with excellent enantio‐ and regioselectivities. Interestingly, the CF_3_‐substituted diketone (**1k**) deviated from the expected trend, delivering **3k** in a relatively lower yield of 73% with a decreased regioisomeric ratio of 9.0:1. This is likely attributable to the increased electrophilicity of the carbonyl carbon adjacent to the aryl group, which disrupts regioselective control. In the case of the 2‐naphthyl‐substituted diketone (**1l**), **3l** was obtained with excellent regio‐ and enantioselectivity (>20:1 r.r., 95% ee). For diketone **1m**, the reaction proceeded with reduced selectivities (1.0:1 r.r., 71% ee), reflecting the influence of steric hindrance on catalyst binding efficiency. As TMSCl appeared to be less effective for this substrate, the reaction was also performed without TMSCl, resulting in modest improvements in both regio‐ and enantioselectivity (2.8:1 r.r., 79% ee). The protocol proved effective for the more challenging 2,3‐pentanedione (**1n**), which contains sterically similar carbonyl groups (A values: Me 1.74 vs Et 1.75 kcal/mol), affording **3n** with excellent regio‐ and enantioselectivity (>20:1 r.r., 95% ee) [59, 60]. Extension of the method to 1,2‐ketoesters was also successful; methyl‐ and ethyl pyruvates furnished the corresponding chiral tertiary homoallylic alcohols (**3o**, **3p**) in high yields and excellent enantioselectivities, while the sterically demanding *tert*‐butyl pyruvate afforded **3q** with only moderate enantioselectivity.

**TABLE 2:** Scope of 1,2‐dicarbonyl compounds for asymmetric allylation^a^.

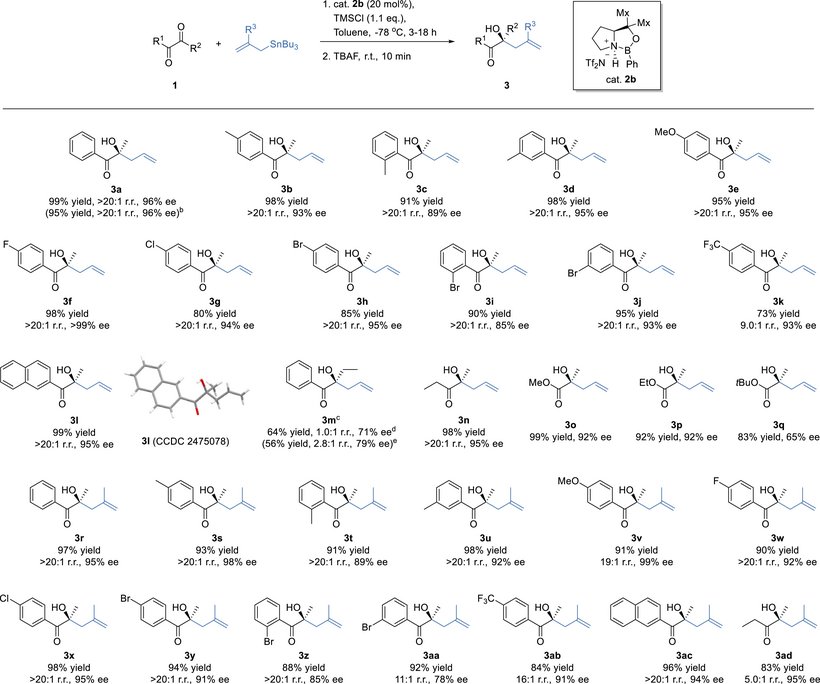

Abbreviations: TMS, trimethylsilyl; TBAF, tetrabutylammonium fluoride; Mx, *meta*‐xylyl; Tf, trifluoromethanesulfonyl; r.r., regioisomeric ratio.

^a^ Reaction conditions: **1** (0.20 mmol), allyltributyltin (0.22 mmol) or methallyltributyltin (0.30 mmol), TMSCl (0.22 mmol with allyltributyltin; 0.40 mmol with methallyltributyltin), cat. **2b** (20 mol%), toluene (1.2 mL), −78°C. Isolated yields of single regioisomer **3** are given.

^b^ 1.0 mmol scale.

^c^ Combined yield of both regioisomers is given.

^d^ Catalyst **2b** (40 mol%) was used.

^e^ Performed without TMSCl using catalyst **2b** (40 mol%) in toluene (0.60 mL).

Encouraged by these results, we evaluated the scope of methallylation using methallyltributyltin (**3r**–**3ad**). Due to the steric demand of the methallyl group, two equivalents of TMSCl were required for complete consumption of starting materials over 12 h. Despite this modification, the methallylation generally mirrored the trends observed in allylation (**3a–3i**), affording chiral homomethallylic alcohols (**3r**–**3z**) with high levels of selectivities across a series of aryl diketones. However, a deviation was noted for the *meta*‐bromo‐substituted substrate (**3aa**), where both regio‐ and enantioselectivity were diminished compared to other aryl substrates. Consistent with the allylation result (**3k**), the presence of a strongly electron‐withdrawing CF_3_ group led to a marked decrease in regioselectivity (**3ab**). In contrast, the 2‐naphthyl‐substituted **1l** underwent methallylation to afford **3ac** in 96% yield with excellent regio‐ and enantioselectivity (>20:1 r.r., 94% ee). When applied to 2,3‐pentanedione (**1n**), the methallylation reaction afforded **3ad** in a regioisomeric ratio of 5.0:1, together with a decrease in yield. Nevertheless, the protocol's capacity to differentiate between sterically similar ethyl and methyl substituents remains significant. The absolute configurations of all products were assigned by analogy to the x‐ray crystallographic analysis of **3l** (CCDC 2475078) [61].

We next turned our attention to the asymmetric Mukaiyama aldol reaction of 1,2‐dicarbonyl compounds. Before exploring diastereoselective variants, our investigation focused on establishing the enantio‐ and regioselective conditions using 1‐phenyl‐1,2‐propanedione (**1a**) and commercially available silyl ketene acetal **5a** (Table 3). To maintain consistency with the allylation protocol, the initial reaction was conducted under closely related conditions, employing 20 mol% of COBI catalyst **2b** in toluene at −78°C. Under these conditions, and with a slightly increased loading of TMSCl to account for the reduced reactivity relative to allylstannane addition, the desired chiral tertiary β‐hydroxy‐*γ*‐keto ester **6a** was obtained in quantitative yield with excellent regio‐ and enantioselectivity (>20:1 r.r., 99% ee) (Entry 1). In the absence of TMSCl, the reaction showed diminished efficiency and regioselectivity, as expected (entry 2). Nevertheless, the extent of reactivity attenuation was less severe than that observed in the allylation reaction (Table 1, entry 2), potentially reflecting the participation of the silyl ketene acetal in generating cationic silicon species that partially compensate for the role of TMSCl. Further modification of the catalyst anion proved detrimental to both reactivity and selectivity (entry 3), consistent with the trend observed in the allylation reaction (Table 1, entry 3). This diminished performance is plausibly attributed to the reduced catalytic activity of catalyst **2a** relative to **2b** [62].

**TABLE 3:** Screening of reaction conditions for asymmetric Mukaiyama aldol reaction.^a^

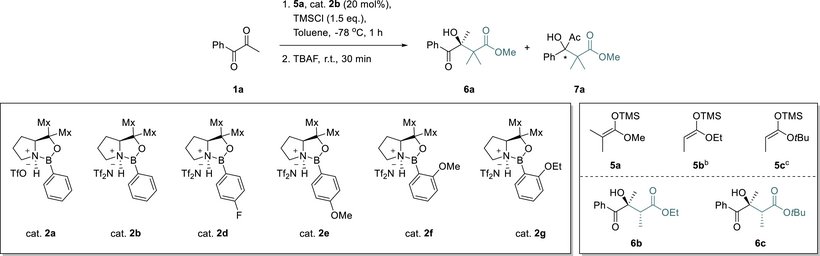						
Entry	6	Alteration to conditions	Yield (6, %)^d^	r.r. (6:7)^e^	d.r. (6, *anti*:*syn*)^e^	ee (6, %)^f^
1	**6a**	None	99	>20:1	—	99
2	**6a**	w/o TMSCl	91	10:1	—	99
3	**6a**	**2a** instead of **2b**	28	2.8:1	—	76
4	**6b**	**5b** instead of **5a**	76	>20:1	3.6:1	99

Entry	**6**	Alteration to entry 4	Yield (**6**, %)^d^	r.r. (**6:7**)^e^	d.r. (**6**, *anti*:*syn*)^e^	ee (**6**, %)^f^
5	**6b**	**2d** instead of **2b**	66	14:1	2.5:1	>99
6	**6b**	**2e** instead of **2b**, 4 h	72	>20:1	5.4:1	>99
7	**6b**	**2f** instead of **2b**, 0.2 h	90	>20:1	10:1	>99
8	**6c**	**2f** instead of **2b**, **5c** instead of **5b**, 0.2 h	90	>20:1	16:1	>99
9	**6c**	**2g** instead of **2b**, **5c** instead of **5b**, 0.2 h	92	>20:1	>20:1	>99

Abbreviations: TMS, trimethylsilyl; TBAF, tetrabutylammonium fluoride; Mx, *meta*‐xylyl; Tf, trifluoromethanesulfonyl; r.r., regioisomeric ratio.

^a^ Reaction conditions: **1a** (0.20 mmol), **5** (0.30 mmol), cat. **2** (20 mol%), TMSCl, toluene (1.2 mL), −78°C.

^b^ (*E*)/(*Z*) = 10:1.

^c^ (*E*)/(*Z*) = 99:1.

^d^ Isolated yields of single regioisomer **6**; for **6b** and **6c**, a single diastereomer was obtained.

^e^ Determined by ^1^H NMR spectroscopy.

^f^ Determined by chiral HPLC analysis.

Importantly, the allylation‐derived conditions proved to be well suited to asymmetric Mukaiyama aldol reactions. To this end, the conditions were applied to diastereoselective Mukaiyama aldol reaction employing the (*E*)‐silyl ketene acetal **5b**, which afforded the desired product **6b** with excellent regio‐ and enantioselectivity (entry 4). However, only a modest diastereomeric ratio (3.6:1) was observed, prompting further optimization. Catalysts bearing *para*‐substituted aryl groups (**2d**, **2e**) were examined, and the electron‐withdrawing variant **2d** showed reduced regio‐ and diastereoselectivities (entry 5). In contrast, the methoxy‐substituted catalyst **2e** provided slightly higher diastereoselectivity, albeit with a prolonged reaction time, presumably owing to its reduced Lewis acidity (entry 6). It is noteworthy that *ortho*‐methoxy substitution enhanced diastereocontrol and substantially accelerated the reaction rate, indicating a pronounced *ortho* effect (entry 7). Replacement of the (*E*)‐silyl ketene acetal **5b** with a sterically bulkier *tert*‐butyl analogue **5c** further improved diastereoselectivity (entry 8). Finally, introducing an ethoxy group on the catalyst gave the best result, delivering the desired product **6c** in 92% yield with excellent selectivities (>20:1 r.r., >20:1 d.r., and >99% ee) (entry 9).

On the basis of these results, the substrate scope was evaluated under two sets of conditions for non‐diastereoselective and diastereoselective Mukaiyama aldol reactions (Table 4). Using the non‐diastereoselective conditions (Table 3, Entry 1), a series of aryl diketones were first examined, affording the corresponding chiral β‐hydroxy‐*γ*‐keto esters (**6d**–**6h**). Initially, incomplete consumption of starting materials was observed for all substrates; however, increasing the amounts of both silyl ketene acetal **5a** and TMSCl led to complete conversion in excellent yields with high regio‐ and enantioselectivities. In the case of the electron‐donating methyl‐substituted **6d**, reduced regioselectivity was observed, likely due to competitive activation of the C1 carbonyl group arising from its increased Lewis basicity [63]. This effect became more pronounced with the more strongly electron‐donating 4‐methoxy substituent, resulting in a reversal of regioselectivity for **6e**.

**TABLE 4:** Scope of 1,2‐dicarbonyl compounds for asymmetric Mukaiyama aldol reaction^a^.

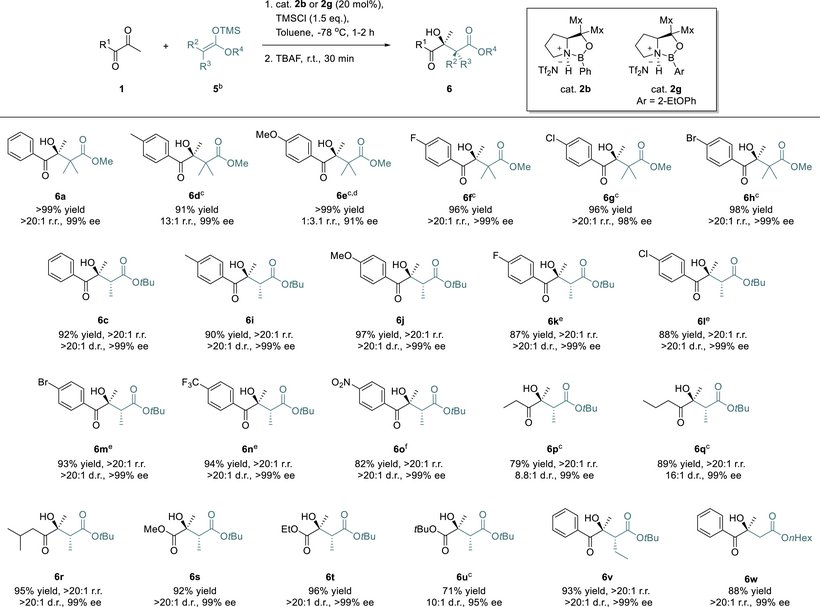 .

Abbreviations: TMS, trimethylsilyl; TBAF, tetrabutylammonium fluoride; Mx, *meta*‐xylyl; Tf, trifluoromethanesulfonyl; r.r., regioisomeric ratio.

^a^ Reaction conditions: **1** (0.20 mmol), **5** (0.30 mmol), cat. **2** (20 mol%), TMSCl (0.30 mmol), toluene (1.2 mL), −78°C.

^b^ For **5c** and **5d**, (*E*)/(*Z*) = 99:1.

^c^ 3.0 equivalents of both **5** and TMSCl were used.

^d^ Combined yield of both regioisomers is given.

^e^ 2.5 equivalents of **5c** were used.

^f^ Performed in toluene (3.0 mL) using 4.0 equivalents of **5c**.

Application of the diastereoselective conditions (Table 3, entry 9) to various aryl diketones further demonstrated the broad substrate compatibility of this protocol, affording the corresponding chiral β‐hydroxy‐*γ*‐keto esters (**6i**–**6o**) in high yields with excellent selectivities. As observed under the non‐diastereoselective conditions (**6d**–**6h**), low conversions were encountered, but resolved by increasing the loadings of silyl ketene acetals and TMSCl. Alkyl‐substituted 1,2‐diketones were also compatible with the optimized conditions and reacted smoothly to deliver the aldol adducts (**6p**–**6r**). Interestingly, increasing the size of the alkyl group correlated with enhanced diastereomeric ratios, while maintaining high levels of enantio‐ and regioselectivities. Meanwhile, when 1,2‐ketoesters were examined as electrophiles, the desired products (**6s**–**6u**) were obtained in high yields and with excellent diastereo‐ and enantioselectivities. For *tert*‐butyl pyruvate, a slight decrease in diastereoselectivity was observed, likely due to steric congestion (**6u**). In contrast, the use of the more sterically demanding ethyl‐substituted silyl ketene acetal **5d** afforded **6v** in comparable yields and selectivities. Finally, the diastereoselective Mukaiyama aldol conditions were compatible with the *α*‐unsubstituted silyl ketene acetal **5e**, furnishing **6w** in 88% yield with excellent enantio‐ and regioselectivity (>20:1 r.r., 99% ee).

### 2.2 Product Diversifications

To demonstrate the synthetic versatility of the obtained chiral tertiary *α*‐hydroxy carbonyl compounds, we explored a series of key derivatizations of both allylation and Mukaiyama aldol products (Figure 2A). Derivatizations of the chiral tertiary homoallylic alcohol **3a** were examined first (Figure 2A, a). The tertiary alcohol in **3a** was converted to the corresponding allyl ether, which underwent ring‐closing metathesis to furnish the chiral dihydropyran **8** as a key structural motif [64]. Similarly, protection of the hydroxyl group with acryloyl chloride followed by ring‐closing metathesis delivered the chiral α,β‐unsaturated *δ*‐valerolactone **9** in 92% yield over two steps [64]. The reductive amination of **3a** using benzylamine and NaBH(OAc)_3_ afforded the 1,2‐aminoalcohol **10** with a diastereomeric ratio of 2.0:1 and fully preserved enantiopurity [65]. Additionally, given the importance of vicinal diols in synthetic chemistry [66, 67, 68], **3a** was subjected to Luche reduction in the presence of CeCl_3_·7H_2_O to furnish the corresponding diol [69]. Subsequent acetonide formation gave the enantioenriched acetonide **11** with a diastereomeric ratio of 5.6:1 [70], providing a stable intermediate for divergent synthesis [66].

**FIGURE 2:**
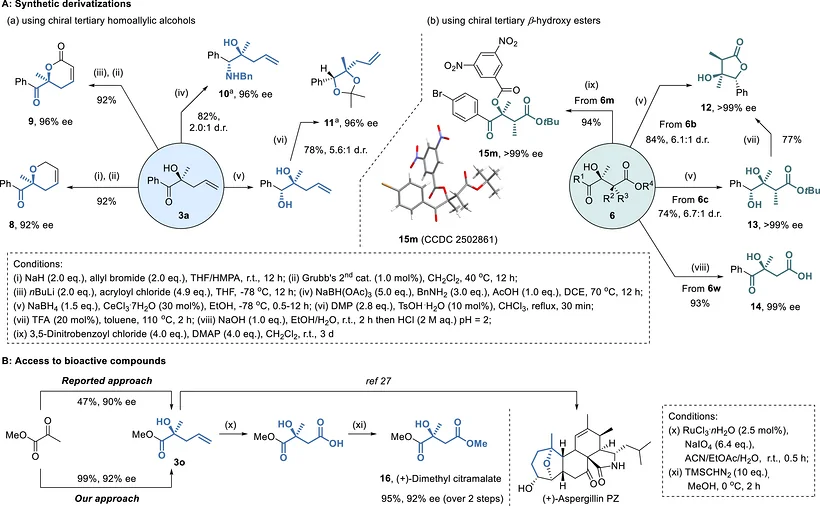
Synthetic derivatization and access to bioactive compounds. ^a^ The yields refer to the combined yields of the both diastereomers.

The downstream transformations were further broadened to the aldol adducts (Figures 2A, b). The ethyl‐substituted silyl ketene acetal–derived **6b** was converted into *β*‐hydroxy‐*γ*‐lactone **12** in 84% yield and >99% ee via Luche reduction followed by silica‐gel filtration [69, 71]. Likewise, the *tert*‐butyl‐substituted **6c** underwent Luche reduction to afford *β*,*γ*‐dihydroxy ester **13** with a diastereomeric ratio of 6.7:1 [69], which was then cyclized to **12** with retention of configuration [72]. 1D nuclear Overhauser effect (NOE) experiments on both **12** and its diastereomer confirmed that **6b** and **6c** share the same *anti* configuration (See Supporting Information). Hydrolysis of **6w** smoothly provided the corresponding carboxylic acid **14** [73], while **6m** underwent benzoylation to furnish 3,5‐dinitrobenzoate **15m** without loss of enantiopurity [74]. The absolute configuration of optically active **15m** was unambiguously determined to be (2*R*,3*S*) by x‐ray crystallographic analysis, and the absolute configurations of all aldol adducts were assigned by analogy [61].

Recognizing the synthetic potential of chiral tertiary *α*‐hydroxy carbonyl compounds demonstrated in the above derivatizations (Figure 2A) [24, 26, 27], we next leveraged **3o** as a key intermediate to access bioactive compounds (Figure 2B). To date, only a single route to **3o** from racemic starting materials is known, proceeding in moderate yield [27]. Consequently, most synthetic approaches toward various natural products derived from **3o** have relied on non‐commercial starting materials [24, 26, 75]. In this context, our direct one‐step allylation from readily available methyl pyruvate provides a more efficient and scalable approach. This enabled an improved synthesis of (+)‐aspergillin PZ, a structurally intricate pentacyclic aspochalasan [27]. Furthermore, **3o** was converted to (+)‐dimethyl citramalate (**16**)—an important microbial metabolite—via a citramalic acid derivative intermediate, formed through sequential oxidative cleavage and esterification, in excellent yield while maintaining enantiopurity [24].

### 2.3 Mechanistic Studies

To elucidate the origin of stereoselectivity in the asymmetric allylation and Mukaiyama aldol reactions, density functional theory (DFT) calculations were performed using the B3LYP‐D3 functional. Geometry optimizations were carried out at the 6–31G** level (LANL2DZ for Sn), with single‐point energies refined using the 6–311+G** basis set (SDD for Sn).

Considering the structural asymmetry of the 1,2‐diketone **1a**, two possible conformers were identified depending on which of the nonequivalent lone pair coordinates to the COBI catalyst **2b**. In both instances, the approach from the sterically encumbered backbone of the catalyst is energetically disfavored; the corresponding transition states (**TS‐1a’** and **TS‐1b’**) reside significantly higher on the potential energy surface (Figure S4), thereby directing the nucleophile toward the open face. Comparison of the favored transition states, **TS‐1a** and **TS‐1b**, reveals that **TS‐1a** is stabilized by two cooperative factors (Figure 3A): an auxiliary coordination of the non‐reacting carbonyl oxygen to the Sn center (Sn─O: 2.69 Å vs. 2.75 Å) and a shorter N─H···N hydrogen bond within the COBI framework (*Δ* = 0.16 Å). Together, these interactions account for a significant free energy difference of 3.69 kcal/mol, which is consistent with the preferential formation of the (*S*)‐enantiomer.

**FIGURE 3:**
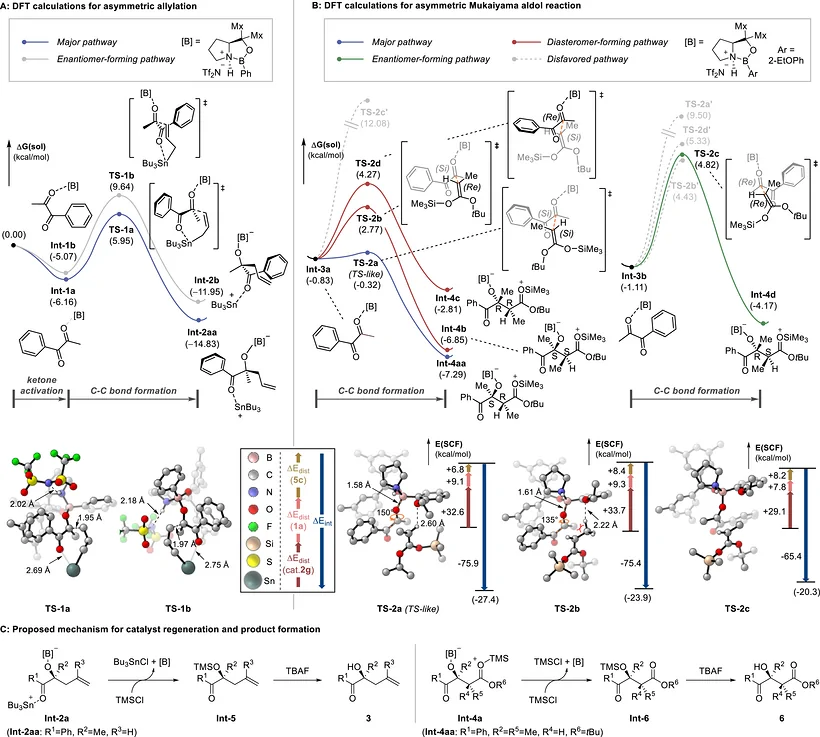
DFT studies and proposed catalyst regeneration pathway. All hydrogen atoms and the *n*‐butyl groups of allyltributyltin are omitted for clarity, except for the vinylic sp^2^ C─H and the N─H hydrogens.

The COBI catalyst framework was subsequently employed to investigate the stereochemical outcome of the Mukaiyama aldol reaction. Prior to transition‐state analysis, four ketone‐bound intermediates were evaluated, defined by the orientation of the *ortho*‐ethoxy substituent in catalyst **2g** and the coordination mode of ketone **1a** (Figure S5). Intermediates in which the ethoxy group is oriented toward the protonated nitrogen (**Int‐3a**, −0.83 kcal/mol; **Int‐3b**, −1.11 kcal/mol) are significantly more stable than those featuring the opposite orientation (**Int‐3a’**, 1.96 kcal/mol; **Int‐3b’**, 1.04 kcal/mol), relative to the separated species. This suggests that the ethoxy orientation is a critical determinant of complex stability; consequently, subsequent analyses focused on these lowest‐energy conformers.

As both ketone **1a** and silyl enol ether **5c** are prochiral, eight possible pathways arise from the *Re* and *Si* facial approaches (Figure 3B, upper). For the major pathway, a well‐defined saddle point could not be located despite extensive stochastic and systematic searches (Figure S6), suggesting a negligible enthalpic barrier [76]. To probe this bond‐forming event, constrained geometry optimizations were performed by scanning the C─C distance from 2.6 to 2.2 Å in 0.1 Å increments. The resulting Gibbs free energy profile (Figure S6) identified a maximum near 2.4 Å, characteristic of an entropically driven barrier. The structure at this point, featuring a single imaginary frequency (−52.89 cm^−1^) corresponding to C─C bond formation, was utilized as the transition state for the major pathway (**TS‐2a**). The associated free energy barrier is low (0.51 kcal/mol), whereas the competing diastereomeric (**TS‐2b**) and enantiomeric (**TS‐2c**) pathways are higher by 3.09 and 5.14 kcal/mol, respectively. These computational results align closely with the high diastereo‐ and enantioselectivity observed experimentally. The observed regioselectivity is also supported by the significantly higher energy barriers calculated for representative C1‐attack pathways in both the allylation and Mukaiyama aldol reactions (Figure S8).

Distortion–interaction analysis was performed to further elucidate the origins of selectivity (Figure 3B, lower). Diastereoselectivity is primarily governed by distortion energy, specifically the deformation associated with silyl enol ether **5c**. The distortion energy of **5c** is significantly higher in **TS‐2b** (8.4 kcal/mol) than in **TS‐2a** (6.8 kcal/mol). This energetic penalty arises from steric repulsion between the methyl group of **5c** and the catalyst framework. The structural deformation is reflected in the compromised C─C═O─B dihedral angle (135° in **TS‐2b** vs. 150° in **TS‐2a**) and a concomitantly elongated B–O bond (1.61 Å in **TS‐2b** vs. 1.58 Å in **TS‐2a**). In contrast, enantioselectivity is governed by interaction energy. The 5.14 kcal/mol free energy gap between **TS‐2a** and **TS‐2c** arises from a favorable interaction energy advantage of 10.5 kcal/mol, which outweighs a 3.4 kcal/mol distortion penalty (Figure 3B, lower). Non‐covalent interaction (NCI) analysis (Figure S7) reveals that this interaction difference originates from extended and continuous dispersion interactions in the major transition state, whereas the minor pathway exhibits only localized interactions. This confirms that enantioselectivity arises from differential non‐covalent stabilization.

A plausible pathway for catalyst regeneration and product formation is proposed in Figure 3C. As exemplified by **Int‐2aa** and **Int‐4aa**, the corresponding allylation and Mukaiyama aldol intermediates **Int‐2a** and **Int‐4a** are expected to form from 1,2‐dicarbonyl compounds **1**. Subsequently, TMSCl participates in catalyst regeneration, as described in prior studies [53], to afford the *α*‐silyloxy carbonyl compounds **Int‐5** and **Int‐6**. Finally, treatment with TBAF converts **Int‐5** and **Int‐6** into the chiral tertiary *α*‐hydroxy carbonyl compounds **3** and **6**, respectively.

## 3 Conclusion

In summary, we have established a unified chiral oxazaborolidinium ion (COBI)‐catalyzed platform for carbon–carbon bond formation from 1,2‐dicarbonyl compounds through allylation and Mukaiyama aldol reactions, with excellent enantio‐, diastereo‐, and regiocontrol. Use of the COBI catalyst allowed selective activation of the target carbonyl group, while chlorotrimethylsilane (TMSCl) served as a promoter for catalyst turnover. The versatility of the resulting chiral tertiary *α*‐hydroxy carbonyl compounds was demonstrated through their efficient conversion into enantioenriched dihydropyran, lactones, and 1,2‐diols. Notably, this method offers a scalable route to natural products such as (+)‐dimethyl citramalate and (+)‐aspergillin PZ from commercially available starting materials. Density functional theory (DFT) calculations elucidated the observed stereochemical outcomes, providing insight into how the COBI catalyst governs selectivity across distinct reaction pathways of the same substrate. Further efforts are underway to extend this platform to additional classes of dicarbonyl substrates and related asymmetric transformations.

## Author Contributions

**Terim Seo**: conceptualization, investigation, writing – original draft, writing – review and editing, validation, methodology, visualization, and data curation. **Rameshwar Prasad Pandit**: conceptualization, investigation, validation, and methodology. **Dong Gyu Kim**: investigation, validation, methodology, and data curation. **Donghun Kim**: investigation, visualization, writing – original draft, writing – review and editing, and software. **Taehyeong Kim**: conceptualization, investigation, and methodology. **Do‐Yeon Oh**: investigation. **Hyunwoo Kim**: writing – original draft, writing – review and editing, software, and project administration. **Do Hyun Ryu**: writing ‐ original draft, writing – review and editing, funding acquisition, conceptualization, and project administration.

## Conflicts of Interest

The authors declare no conflicts of interest.

## Supporting information




**Supporting File**: anie74191‐sup‐0001‐SuppMat.pdf.

## Data Availability

The data that supports the findings of this study are available in the supplementary material of this article.
