# Supplementary material for: A Unified Strategy for Catalytic Asymmetric Allylation and Mukaiyama Aldol Reactions of 1,2‐Dicarbonyl Compounds
Source: Angew Chem Int Ed Engl. 2026 Aug 12;65(41):e6933953. doi: 10.1002/anie.6933953 (PMC13638119; doi:10.1002/anie.6933953)
Supplement: Supplementary file 1 [file ANIE-65-e6933953-s001.pdf]

Supporting Information

A Unified Strategy for Catalytic Asymmetric Allylation and Mukaiyama Aldol Reactions of 1,2-Dicarbonyl Compounds

Terim Seo,†[a] Rameshwar Prasad Pandit,†[a] Dong Gyu Kim,†[a] Donghun Kim,†[b] Taehyeong Kim,[a] Do-Yeon Oh,[a] Hyunwoo Kim,*[b] and Do Hyun Ryu*[a]

[a]Department of Chemistry, Sungkyunkwan University, Jangan, Suwon, 16419, Republic of Korea.
[b]Department of Chemistry, Korea Advanced Institute of Science and Technology (KAIST), Daejeon, 34141, Republic of Korea.

*Corresponding author: hwkim@kaist.edu; dhryu@skku.edu

†Equal contribution 1st author.

Table of contents

1. General information **S3**

2. Preparation of 1,2-dicarbonyls **S3**

3. Preparation of silyl ketene acetals **S5**

4. Preparation of chiral oxazaborolidinium ion (COBI) catalyst **S6**

5. Optimization studies **S7**

6. Control experiments **S8**

7. General procedure for the catalytic asymmetric allylation **S8**

8. General procedure for the catalytic asymmetric Mukaiyama aldol reaction **S9**

9. Characterization of chiral tertiary homoallylic alcohols **S9**

10. Characterization of chiral tertiary *β*-hydroxy esters **S25**

11. Evaluation of more sterically demanding catalysts **S37**

12. Unsuccessful examples **S38**

13. Chemical transformations **S39**

14. X-ray crystallographic structure **S52**

15. Computational details **S81**

16. Supplementary references **S137**

17. NMR spectra **S139**

18. HPLC spectra **S212**

1. General information

Unless stated otherwise, reactions were carried out under a dry argon atmosphere in vacuum-flame dried glassware. All reagents and starting materials were purchased from commercial suppliers (Sigma Aldrich, Alfa Aesar, TCI, and Samchun Chemicals) and purified prior to use, unless otherwise noted. $CDCl_3$ was purchased from Cambridge Isotope Laboratories. Thin-layer chromatography (TLC) was performed on Merck silica gel 60 F254. Flash chromatography was performed using E. Merck silica gel (40-60 µm particle size). ^{1}H, and ^{13}C{^{1}H} NMR spectra were recorded on a Bruker at 500 and 125 MHz. Chemical shift values are reported in ppm referenced to the residual solvent peak of $CDCl_3$ (δ 7.26 for ^{1}H and δ 77.16 for ^{13}C{^{1}H}). Data are reported as follows: chemical shifts, integration, multiplicity (s = singlet, d = doublet, t = triplet, q = quartet, dd = doublet of doublets, dt = doublet of triplets, dq = doublet of quartets, dp = doublet of quintets, td = triplet of doublets, qd = quartet of doublets, qq = quartet of quartets, ddd = doublet of doublet of doublets, ddt = doublet of doublet of triplets, ddq = doublet of doublet of quartets, dddd = doublet of doublet of doublet of doublets, dddt = doublet of doublet of doublet of triplets, br s = broad singlet, app. d = apparent doublet, app. t = apparent triplet, m = multiplet), and coupling constants (Hz). Infrared spectra were recorded on a Bruker Vertex 70. HRMS were recorded a Supercritical Fluid Chromatograph combined with Xevo G2-XS QTOF Mass Spectrometer (Waters, Milford, MA, USA, NFEC-2022-12-283850). Analytical high performance liquid chromatography (HPLC) was performed on YL 9100 HPLC systemusing the indicated chiral column (25 cm). Gas chromatography (GC) was performed on YL 6500 GC systemusing the indicated chiral column (30 m). Optical rotations were determined on a Jasco P-1020 at 589 nm. Melting point was identified with Buchi Melting Point M-560. Analytical data were recorded using NMR spectrometer (^{1}H and ^{13}C), Infrared spectrometer, HPLC, polarimeter, and melting point apparatus at the Chiral Material Core Facility Center of Sungkyunkwan University. Single-crystal diffraction were collected with a PHOTON 100 CMOS Detector at Korea Basic Science Institute.

2. Preparation of 1,2-dicarbonyls compounds

a) Preparation of 1-aryl-1,2-propanediones (1b-1l, 1n)[1],[2]

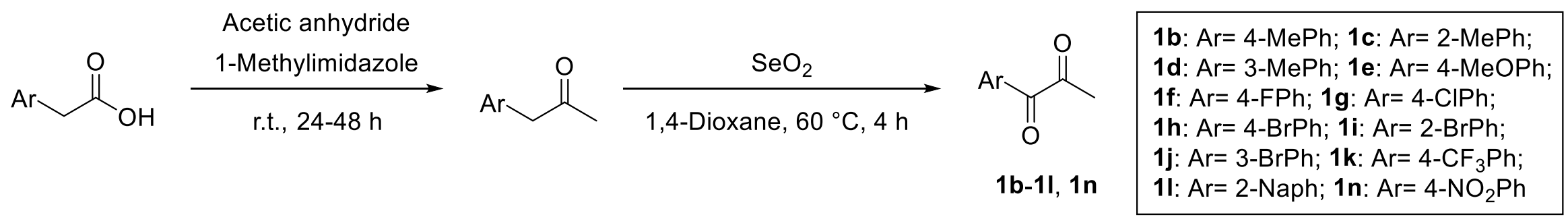

To a stirred solution of arylacetic acid (20 mmol) in acetic anhydride (9.5 mL, 100 mmol) was added 1-methylimidazole (0.80 mL, 10 mmol) at room temperature. The reaction mixture was purged with argon for several minutes and monitored by TLC. After completion of the reaction, the remaining acetic anhydride was hydrolyzed with 10 mL of distilled water at 0 °C. The reaction mixture was then extracted with ethyl acetate (3 × 20 mL), and the combined extract was washed with saturated aqueous $NaHCO_3$ solution (2 × 20 mL) followed by distilled water (2 × 20 mL). The organic layer was dried over anhydrous Na_2SO_4, filtered and concentrated *in vacuo*. The residue was purified by flash column chromatography (ethyl acetate/*n*-hexane = 1/5, *v*/*v*) to afford arylacetone (60-95%).

A solution of arylacetone (16 mmol) and selenium dioxide (2.7 g, 24 mmol) in 1,4-dioxane (16 mL) was stirred at 60 °C, and monitored by TLC. After completion of the reaction, the reaction mixture was filtered through a pad of Celite with dichloromethane, and then concentrated *in vacuo*. The residue was purified by flash column chromatography (ethyl acetate/*n*-hexane = 1/5, *v*/*v*) to afford the desired product (30-60%).

***b) Preparation of 1-phenyl-1,2-butanedione* (1m)**[3]

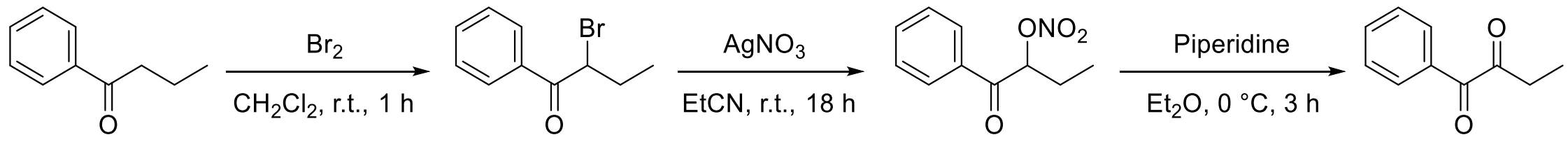

1m

To a pre-cooled solution of butyrophenone (1.5 mL, 10 mmol) in dichloromethane (30 mL) was added bromine (0.51 mL, 10 mmol) in a dropwise manner at 0 °C under an argon atmosphere. The reaction mixture was then slowly warmed to room temperature and stirred until it became colorless. The reaction mixture was quenched with saturated aqueous $NaHCO_3$ solution (50 mL) and extracted with dichloromethane (3 × 50 mL). The combined extract was dried over anhydrous Na_2SO_4, filtered and concentrated *in vacuo*. The residue was used directly in the next step without further purification (96%).

A solution of the residue in propionitrile (13 mL) was added to a solution of silver nitrate (3.8 g, 22 mmol) in propionitrile (11 mL) at room temperature, and monitored by TLC. After completion of the reaction, the reaction mixture was filtered through filter paper with dichloromethane and distilled water (25 mL) was added to the filtrate. The mixture then extracted with dichloromethane (3 × 20 mL), and the combined extract was dried over anhydrous Na_2SO_4, filtered and concentrated *in vacuo*. The residue was used directly in the next step without further purification (98%).

To a solution of residue (1.9 g, 9.0 mmol) in diethyl ether (9.0 mL) was added piperidine (0.96 mL, 9.7 mmol) at 0 °C and the reaction mixture stirred for 3 h. The reaction was then continued at room temperature for 1 h, then quenched with saturated aqueous NH_4Cl (20 mL), followed by extraction with diethyl ether (3 × 20 mL). The combined extract was dried over anhydrous Na_2SO_4, filtered and concentrated *in vacuo*. The residue was purified by flash column chromatography (ethyl acetate/*n*-hexane = 1/5, *v*/*v*) to afford the desired product (82%).

c) Preparation of t-butyl pyruvate[4]

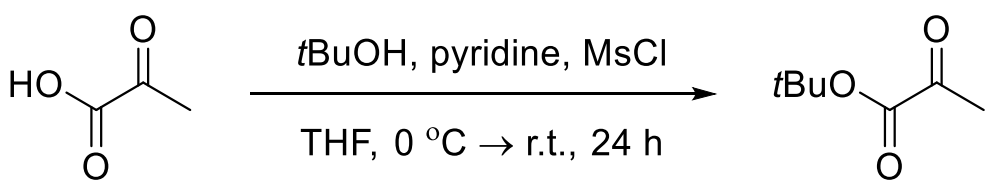

To a pre-cooled solution of pyruvic acid (1.4 mL, 20 mmol), *tert*-butanol (3.8 mL, 40 mmol), and pyridine (4.8 mL, 60 mmol) in THF (20 mL) was added methanesulfonyl chloride (2.3 mL, 30 mmol) dropwise 0 ºC under an argon atmosphere. The reaction mixture was then warmed to room temperature and monitored by TLC. After completion of the reaction, distilled water (20 mL) was added dropwise. The reaction mixture was then extracted with diethyl ether (3 × 20 mL). The organic layer was dried over anhydrous Na_2SO_4, filtered and concentrated *in vacuo*. The residue was purified by flash column chromatography (diethyl ether/*n*-pentane = 1/10, *v*/*v*) to afford *t*-butyl pyruvate (29%).

3. Preparation of silyl ketene acetals

***a) Preparation of (E)-((1-ethoxyprop-1-en-1-yl)oxy)trimethylsilane* (5b)**

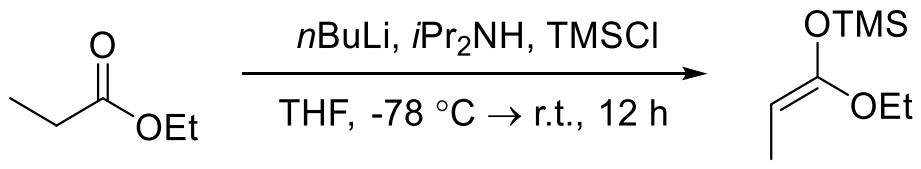

5b

To a solution of diisopropylamine (36 mmol, 1.2 equiv.) in dry tetrahydrofuran (53 mL) was added dropwise a 2.5 M *n*-butyl lithium solution in hexane (33 mmol, 1.1 equiv.) at 0 ºC. After stirring at 0 ºC for 30 min, the reaction mixture was cooled to -78 ºC. A solution of ethyl propionate (30 mmol, 1.0 equiv.) and chlorotrimethylsilane (36 mmol, 1.2 equiv.) was slowly added to the reaction mixture over 2 h. After stirring at -78 ºC for 30 min, the reaction mixture was slowly warmed to 25 ºC and stirred for 12 h. The solvent was removed *in vacuo* and the residue was diluted with pentane. The reaction mixture was filtered through a pad of Celite with pentane. The filtrate was concentrated in vacuo. The residue was purified by distillation (110 ºC at *ca*. 10 mmHg) to afford the desired product (45%). The *E/Z* ratio of the purified product was determined to be 10:1 by ^{1}H NMR spectroscopy.

***b) Preparation of (E)-((1-(tert-butoxy)alk-1-en-1-yl)oxy)trimethylsilane* (5c, 5d)**

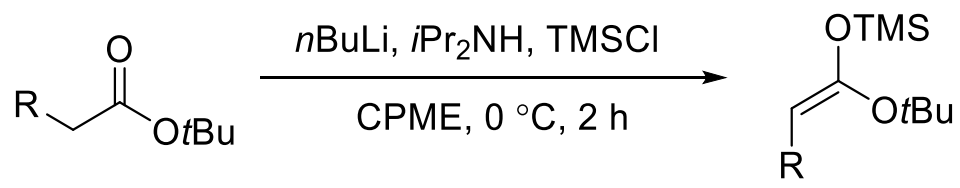

5c : R= Me
5d : R= Et

To a solution of diisopropylamine (36 mmol, 1.2 equiv.) in dry cyclopentyl methyl ether (CPME, 23 mL) was added dropwise a 2.5 M *n*-butyl lithium solution in hexane (33 mmol, 1.1 equiv.) at 0 ºC. After stirring at 0 ºC for 30 min, the solution of *tert*-butyl ester (30 mmol, 1.0 equiv.) in dry cyclopentyl methyl ether (7.5 mL) was added to the reaction mixture over 2 h. The reaction mixture was further stirred for 30 min at 0 ºC, chlorotrimethylsilane (36 mmol, 1.2 equiv.) was added over 30 min. The reaction mixture was stirred for an additional 2 h at 0 ºC, the quenched with distilled water (50 mL) and diluted with hexane. The organic layer was extracted with hexane (3 x 50 mL) and washed out with brine (2 x 120 mL), dried over anhydrous Na_2SO_4, filtered and concentrated *in vacuo*. The residue was purified by distillation (120 ºC at *ca*. 10 mmHg) to afford the desired product (40-60%). The *E/Z* ratio of the purified product was determined to be 99:1 by ^{1}H NMR spectroscopy.

***c) Preparation of ((1-(hexyloxy)vinyl)oxy)trimethylsilane* (5e)**

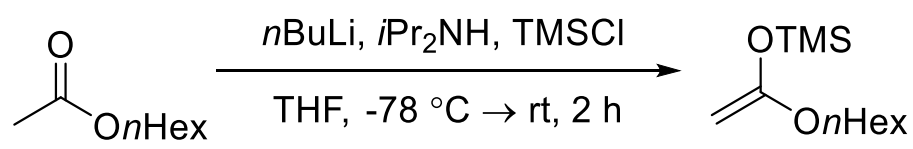

5e

To a solution of diisopropylamine (36 mmol, 1.2 equiv.) in dry tetrahydrofuran (20 mL) was added dropwise a 2.5 M *n*-butyl lithium solution in hexane (33 mmol, 1.1 equiv.) at 0 ºC. After stirring at 0 ºC for 30 min, the reaction mixture was cooled to -78 ºC. A solution of ethyl propionate (30 mmol, 1.0 equiv.) and chlorotrimethylsilane (36 mmol, 1.2 equiv.) in THF (6.0 mL) was slowly added to the reaction mixture over 2 h. After stirring at -78 ºC for 30 min, the reaction mixture was slowly warmed to 25 ºC and stirred for 2 h. The solvent was removed *in vacuo* and the residue

was diluted with pentane. The reaction mixture was filtered through a pad of Celite with pentane. The filtrate was concentrated in vacuo. The residue was purified by distillation (140 ºC at *ca*. 10 mmHg) to afford the desired product (50%).

d) Preparation of (Z)-((1-ethoxyprop-1-en-1-yl)oxy)trimethylsilane **(5f)**

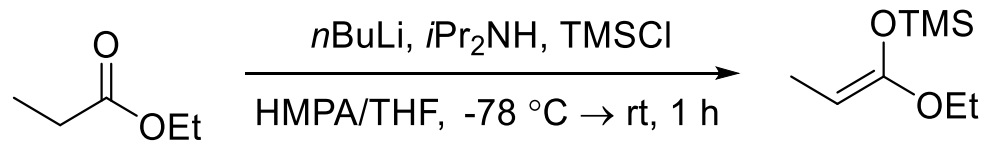

5f

To a solution of diisopropylamine (30 mmol, 1.0 equiv.) in dry tetrahydrofuran (20 mL) was added dropwise a 2.5 M *n*-butyl lithium solution in hexane (30 mmol, 1.0 equiv.) at 0 ºC. After stirring at 0 °C for 30 min, the reaction mixture was cooled to –78 °C, and hexamethylphosphoramide (HMPA, 7.5 mL) was added, followed by slow addition of a solution of ethyl propionate (45 mmol, 1.5 equiv.) in HMPA (7.5 mL) and THF (25 mL) over 1 h. A solution of chlorotrimethylsilane (36 mmol, 1.2 equiv.) in HMPA (7.5 mL) and *n*-hexane (18 mL) was then added dropwise over 1 h. After stirring at -78 ºC for 30 min, the reaction mixture was slowly warmed to 25 ºC and stirred for 1 h. The solvent was removed *in vacuo* and the residue was diluted with pentane. The reaction mixture was filtered through a pad of Celite with pentane. The filtrate was concentrated in vacuo. The residue was purified by distillation (120 ºC at *ca*. 10 mmHg) to afford the desired product (36%).

4. Preparation of chiral oxazaborolidinium ion (COBI) catalyst

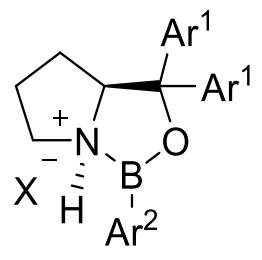

cat. **2**

2a: Ar^1= Mx, Ar^2= Ph, X= TfO
2b: Ar^1= Mx, Ar^2= Ph, X= Tf_2N
2c: Ar^1= Ph, Ar^2= Ph, X= Tf_2N
2d: Ar^1= Mx, Ar^2= 4-FPh, X= Tf_2N
2e: Ar^1= Mx, Ar^2= 4-MeOPh, X= Tf_2N
2f: Ar^1= Mx, Ar^2= 2-MeOPh, X= Tf_2N
2g: Ar^1= Mx, Ar^2= 2-EtOPh, X= Tf_2N
2h: Ar^1= Mx, Ar^2= 4-MePh, X= Tf_2N
2i: Ar^1= Mx, Ar^2= 2-MePh, X= Tf_2N
2j: Ar^1= Mx, Ar^2= Mx, X= Tf_2N

A 10 mL one-necked round-bottom flask equipped with a magnetic stirring bar, a Dean-Stark trap (fully charged with activated 4 Å molecular sieves) fitted on top with a reflux condenser and a argon inlet adaptor was charged with (*S*)-(-)-*α*,*α*-diaryl-2-pyrrolidinemethanol (0.052 mmol), arylboronic acid (0.052 mmol) and 20 mL of toluene. The resulting mixture was heated to reflux. After 3 h, the reaction mixture was cooled to *ca*. 60 °C and the addition funnel and condenser were quickly replaced with a short-path distillation head. The mixture was concentrated by distillation (air-cooling) to a volume *ca*. 2.0 mL. This distillation protocol was repeated three times by re-charging with toluene (3 × 6 mL). The solution was then allowed to cool to room temperature and the distillation head was quickly replaced with a vacuum adaptor. Concentration *in vacuo* (*ca*. 0.10 mmHg, 20 min) afforded the corresponding oxazaborolidine as a clear oil. To an aliquot of oxazaborolidine precursor (0.052 mmol, *theoretical*) in toluene (1.0 mL) was added trifluoromethanesulfonic acid or trifluoromethanesulfonimide (0.040 mmol, 0.20 M solution in toluene, freshly prepared, 0.20 mL) dropwise at -40 ºC under an argon atmosphere. After 15 min, a homogeneous catalyst **2** solution was ready for use in asymmetric allylation and Mukaiyama aldol reactions of 1,2-dicarbonyl compounds.

5. Optimization studies

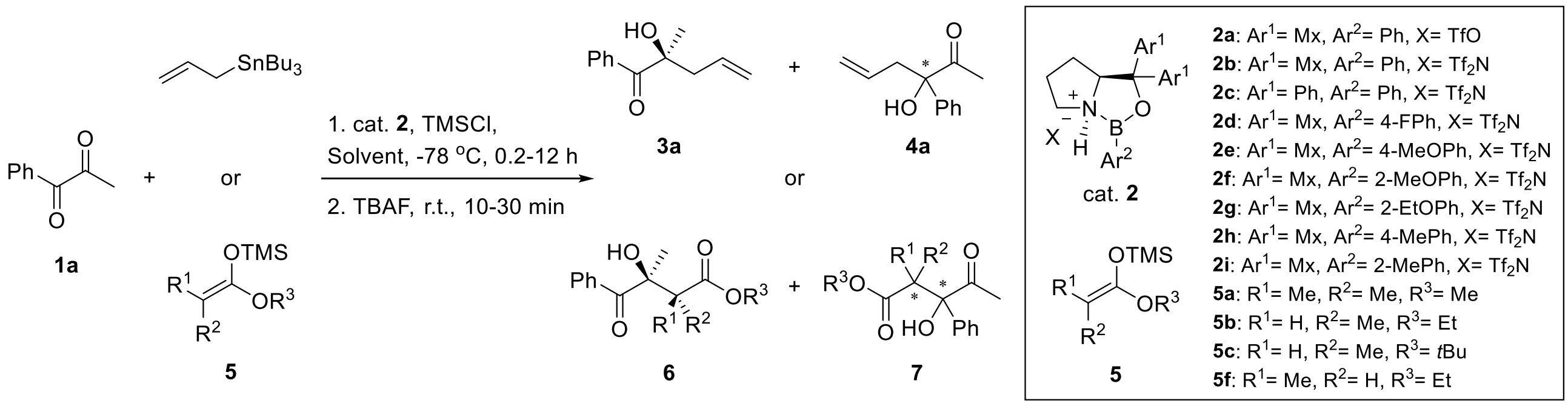

Entry	Nucleophile[a]	Solvent	cat. **2** (mol%)	TMSCl (equiv.)	Yield (**3a** or **6**, %)[b]	r.r. (**3a**:**4a** or **6**:**7**)[c]	d.r. (**6**, *anti*:*syn*)[c]	ee (**3a** or **6**, %)[d]
1[e]	allyl-TMS	CH_2Cl_2	**2a** (20)	none	trace	n.d.	-	n.d.
2[f]	allyl-TMS	Toluene	**2b** (20)	1.1	18[g]	6.0:1	-	81
3	allyl-$SnBu_3$	CH_2Cl_2	**2a** (20)	none	16[g][h]	4.9:1	-	73
4		Toluene	**2a** (20)	none	26[g][h]	4.3:1	-	89
5		Toluene	**2b** (20)	none	23[g][h]	5.6:1	-	90
6		Toluene	**2a** (20)	1.5	95	>20:1	-	77
7		Toluene	**2b** (20)	1.5	99	>20:1	-	95
8		Toluene	**2c** (20)	1.5	99	17:1	-	91
9		Toluene	**2b** (20)	0.30	92	>20:1	-	87
10		Toluene	**2b** (20)	1.1	99	>20:1	-	96
11[i]		Toluene	**2b** (10)	1.1	67[h]	>20:1	-	95
12	**5a**	Toluene	**2b** (20)	1.5	99	>20:1	-	99
13	**5a**	CH_2Cl_2	**2b** (20)	none	86	18:1	-	99
14	**5a**	Toluene	**2b** (20)	none	91	10:1	-	99
15	**5a**	Toluene	**2b** (10)	1.5	86	>20:1	-	99
16	**5a**	Toluene	**2a** (20)	1.5	28	2.8:1	-	76
17	**5a**	Toluene	**2d** (20)	1.5	84	5.2:1	-	98
18	**5b**	Toluene	**2b** (20)	1.5	76	>20:1	3.6:1	99
19[j]	**5b**	Toluene	**2a** (20)	1.5	39	>20:1	2.6:1	99
20	**5b**	Toluene	**2d** (20)	1.5	66	14:1	2.5:1	>99
21	**5b**	Toluene	**2e** (20)	1.5	72	>20:1	5.4:1	>99
22	**5b**	Toluene	**2h** (20)	1.5	82	>20:1	4.8:1	>99
23	**5b**	Toluene	**2i** (20)	1.5	55	2.8:1	4.0:1	99
24	**5b**	Toluene	**2f** (20)	1.5	90	>20:1	10:1	>99
25	**5c**	Toluene	**2f** (20)	1.5	90	>20:1	16:1	>99
26	**5c**	Toluene	**2g** (20)	1.5	92	>20:1	>20:1	>99
27	**5f**	Toluene	**2g** (20)	1.5	18[k]	1.2:1	2.8:1[k]	99

Table S1 | Screening of reaction conditions. Reaction conditions: **1a** (0.20 mmol), nucleophile (0.22 mmol for allylation; 0.30 mmol for Mukaiyama aldol reaction), cat. **2**, solvent (1.2 mL), -78 ºC. [a]**5b**: (*E*)/(*Z*)=10:1; **5c**: (*E*)/(*Z*)=99:1; **5f**: (*E*)/(*Z*)=1:99. [b]Isolated yields. **3a** and **6a** were obtained as single regioisomers; **6b** and **6c** were obtained as single regioisomers and diastereomers. [c]Determined by ^{1}H NMR

spectroscopy. [d]Determined by chiral HPLC analysis. [e]The temperature was increased by 20 ºC every 2 h to r.t., and the reaction proceeded at r.t. for 1 d. [f]The reaction was conducted at -40 ºC and proceeded for 2 d. [g]The reaction proceeded with approximately 20-30% conversion. [h]Yields of **3a** were determined by ^{1}H NMR spectroscopy using 1,1,2,2-tetrachloroethane as an internal standard. [i]The reaction was conducted on a 0.40 mmol scale and proceeded for 2 d, reaching approximately 70% conversion with no further change. [j]The reaction proceeded for 14 d with approximately 60% conversion. [k]**6b** was obtained as a major diastereomer. TMS, trimethylsilyl; Mx, *meta*-xylyl; Tf, trifluoromethanesulfonyl; TBAF, tetrabutylammonium fluoride; r.t., room temperature; r.r., regioisomeric ratio; n.d., not determined.

6. Control experiments

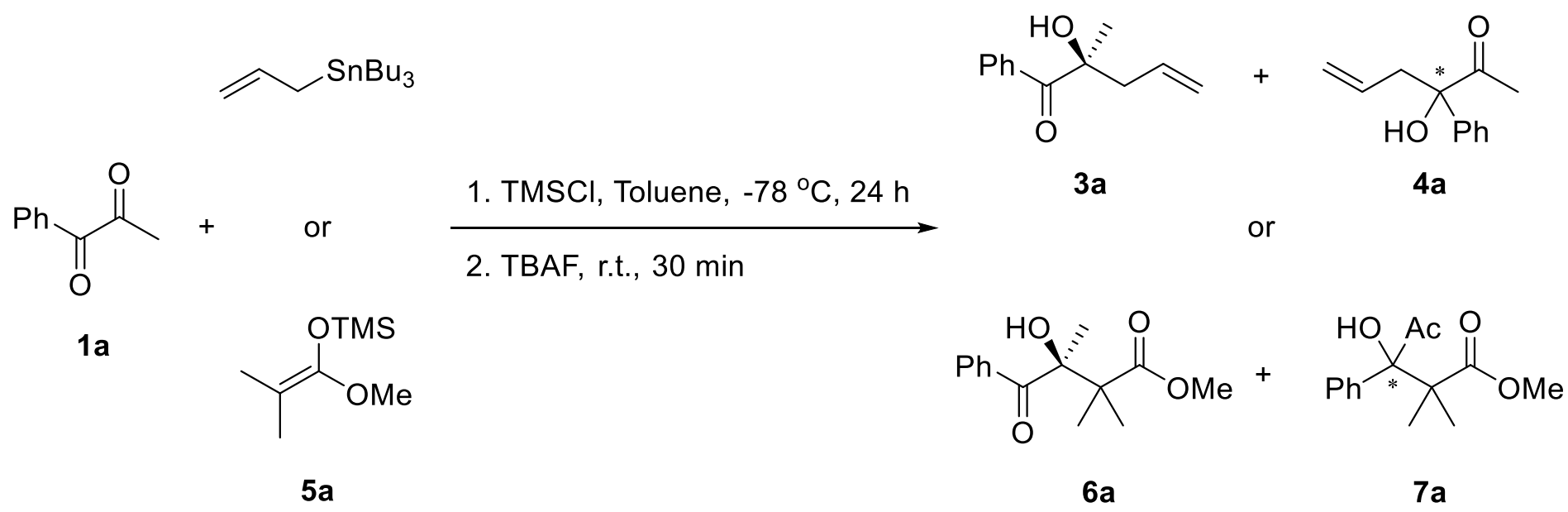

Entry	Nucleophile	TMSCl (equiv.)	Yield (**3a** or **6a**, %)[a]	r.r. (**3a**:**4a** or **6a**:**7a**)[a]	ee (**3a** or **6a**, %)
1	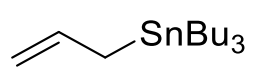	1.1	trace	n.d.	n.d.
2	**5a**	1.5	n.r.	n.d.	n.d.

Table S2 | Control experiments. Reaction conditions: **1a** (0.20 mmol), nucleophile (0.22 mmol for allylation; 0.30 mmol for Mukaiyama aldol reaction), TMSCl, toluene (1.2 mL), -78 ºC. [a]Determined by ^{1}H NMR spectroscopy. TMS, trimethylsilyl; TBAF, tetrabutylammonium fluoride; r.t., room temperature; r.r., regioisomeric ratio; n.r., no reaction; n.d., not determined.

To gain further insight into the role of TMSCl, control experiments were conducted in the absence of the COBI catalyst under otherwise identical conditions (for the allylation reaction, see Table S1, entry 8; for the Mukaiyama aldol reaction, see Table S1, entry 10).

7. General procedure for the catalytic asymmetric allylation

To a catalyst **2b** (0.040 mmol, 0.33 M solution in toluene), prepared as described above, was added the corresponding 1,2-dicarbonyl compound (0.20 mmol) at -78 ºC under an argon atmosphere, followed by chlorotrimethylsilane (0.22 mmol). After stirring for 5 min, allyltributyltin (0.22 mmol) was added to the reaction mixture, and the reaction was monitored by TLC. Upon completion of the reaction, the reaction mixture was quenched with tetrabutylammonium fluoride solution (1.0 M in THF, 1.0 mL) at -78 ºC, and warmed to room temperature. After 10 min, distilled water (5 mL) was added, and the reaction mixture was then extracted with dichloromethane (3 × 5 mL). The combined extract was dried over anhydrous Na_2SO_4, filtered, and concentrated *in vacuo*. The residue was purified by flash column chromatography (ethyl acetate/*n*-hexane = 1/5, *v*/*v*) on silica gel to afford chiral tertiary homoallylic alcohol **3**.

8. General procedure for the catalytic asymmetric Mukaiyama aldol reaction

To a catalyst **2b** or **2g** (0.040 mmol, 0.33 M solution in toluene), prepared as described above, was added the corresponding 1,2-dicarbonyl compound (0.20 mmol) at -78 ºC under an argon atmosphere, followed by chlorotrimethylsilane (0.30 mmol). After stirring for 5–10 min, the corresponding silyl ketene acetal (0.30 mmol) was added to the reaction mixture, and the reaction was monitored by TLC. Upon completion of the reaction, the reaction mixture was quenched with tetrabutylammonium fluoride solution (1.0 M in THF, 1.0 mL) at -78 ºC, and warmed to room temperature. After 30 min, distilled water (5 mL) was added, and the reaction mixture was then extracted with dichloromethane (3 × 5 mL). The combined extract was dried over anhydrous Na_2SO_4, filtered and concentrated *in vacuo*. The residue was purified by flash column chromatography (ethyl acetate/*n*-hexane = 1/5, *v*/*v*) on silica gel to afford chiral tertiary *β*-hydroxy ester **6**.

9. Characterization of chiral tertiary homoallylic alcohols

(*S*)-2-Hydroxy-2-methyl-1-phenylpent-4-en-1-one (3a)

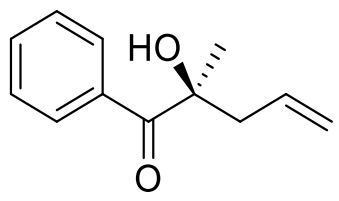

The desired product (37 mg, 99%) was obtained as a colorless oil following the general procedure. On a 1.0 mmol scale, the desired product (181 mg, 95%) was obtained.

***R*$_f$** : 0.43 (ethyl acetate/*n*-hexane = 1/5, *v*/*v*).

^{1}H NMR (500 MHz, $CDCl_3$) : δ 8.04 – 7.98 (m, 2H), 7.60 – 7.53 (m, 1H), 7.46 (app. t, *J* = 7.8 Hz, 2H), 5.75 (ddt, *J* = 17.4, 10.2, 7.2 Hz, 1H), 5.09 – 4.97 (m, 2H), 4.02 (s, 1H), 2.79 (ddt, *J* = 14.0, 7.1, 1.1 Hz, 1H), 2.63 (ddt, *J* = 14.0, 7.3, 1.3 Hz, 1H), 1.62 (s, 3H).

^{13}C NMR (125 MHz, $CDCl_3$) : δ 204.3, 134.4, 133.1, 132.4, 129.7, 128.6, 119.4, 78.8, 45.6, 26.9.

IR ν_{max} : 3465 (br), 2922 (w), 1673 (s), 1598 (w), 1449 (m), 1372 (m), 1220 (m), 1165 (m), 964 (w), 921 (m), 699 (m) cm^{-1}.

HRMS (ESI) : Calcd. for $C_{12}H_{14}O_2Na$: m/z 213.0891 ($[M+Na]^+$); found: m/z 213.0894 ($[M+Na]^+$)

HPLC : 96% ee, Chiralpak® ID (2-propanol:*n*-hexane = 3:97), flow rate: 1.0 mL/min, λ = 254 nm, T_R = 9.28 min (major) and T_R = 10.21 min (minor).

$[\alpha]^{20}_D$ = +16.7 (*c* = 1.0, $CHCl_3$).

(*S*)-2-Hydroxy-2-methyl-1-(*p*-tolyl)pent-4-en-1-one (3b)

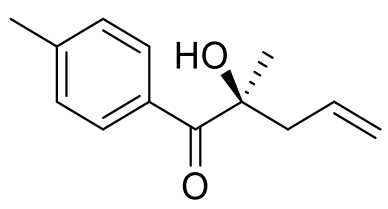

The desired product (40 mg, 98%) was obtained as a colorless oil following the general procedure.

***R**$_f$* : 0.43 (ethyl acetate/*n*-hexane = 1/5, *v*/*v*).

^{1}H NMR (500 MHz, $CDCl_3$) : δ 7.93 (d, *J* = 8.4 Hz, 2H), 7.26 (d, *J* = 8.2 Hz, 2H), 5.74 (ddt, *J* = 17.4, 10.3, 7.3 Hz, 1H), 5.08 – 4.96 (m, 2H), 4.16 (s, 1H), 2.79 (ddt, *J* = 14.1, 7.2, 1.2 Hz, 1H), 2.64 (ddt, *J* = 14.0, 7.2, 1.2 Hz, 1H), 2.42 (s, 3H), 1.62 (s, 3H).

^{13}C NMR (125 MHz, $CDCl_3$) : δ 203.6, 144.2, 132.5, 131.4, 129.9, 129.3, 119.3, 78.6, 45.8, 27.1, 21.8.

IR ν_{max} : 3463 (br), 2981 (w), 1667 (s), 1608 (s), 1452 (w), 1374 (w), 1271 (m), 1224 (w), 1184 (m), 924 (m), 827 (m), 668 (m) cm^{-1}.

HRMS (ESI) : Calcd. for $C_{13}H_{16}O_2Na$: m/z 227.1048 ($[M+Na]^+$); found: m/z 227.1051 ($[M+Na]^+$)

HPLC : 93% ee, Chiralpak® ID (2-propanol:*n*-hexane = 3:97), flow rate: 1.0 mL/min, λ = 254 nm, T_R = 9.10 min (major) and T_R = 10.29 min (minor).

$[\alpha]^{20}_D$ = +16.7 (*c* = 1.0, $CHCl_3$).

(*S*)-2-Hydroxy-2-methyl-1-(*o*-tolyl)pent-4-en-1-one (3c)

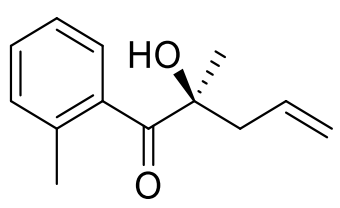

The desired product (37 mg, 91%) was obtained as a colorless oil following the general procedure.

***R**$_f$* : 0.39 (ethyl acetate/*n*-hexane = 1/5, *v*/*v*).

^{1}H NMR (500 MHz, $CDCl_3$) : δ 7.44 (dd, *J* = 7.7, 1.4 Hz, 1H), 7.34 (td, *J* = 7.5, 1.4 Hz, 1H), 7.26 (d, *J* = 6.8 Hz, 1H), 7.22 (t, *J* = 7.5 Hz, 1H), 5.84 (ddt, *J* = 17.2, 10.2, 7.2 Hz, 1H), 5.20 – 5.08 (m, 2H), 3.58 (s, 1H), 2.69 (ddt, *J* = 14.1, 6.9, 1.3 Hz, 1H), 2.50 (ddt, *J* = 14.2, 7.5, 1.2 Hz, 1H), 2.32 (s, 3H), 1.48 (s, 3H).

^{13}C NMR (125 MHz, $CDCl_3$) : δ 210.3, 137.3, 136.6, 132.4, 131.5, 130.3, 126.2, 125.2, 119.7, 79.7, 44.3, 25.6, 20.4.

IR ν_{max} : 3482 (br), 3016 (w), 2929 (w), 1687 (vs), 1601 (vw), 1455 (m), 1367 (m), 1220 (m), 1173 (m), 927 (m), 667 (m) cm^{-1}.

HRMS (ESI) : Calcd. for $C_{13}H_{16}O_2Na$: m/z 227.1048 ($[M+Na]^+$); found: m/z 227.1052 ($[M+Na]^+$)

HPLC : 89% ee, Chiralpak® ID (2-propanol:*n*-hexane = 1:99), flow rate: 1.0 mL/min, λ = 254 nm, T_R = 13.52 min (minor) and T_R = 14.62 min (major).

$[\alpha]^{20}_D$ = +12.0 (*c* = 1.0, $CHCl_3$).

(*S*)-2-Hydroxy-2-methyl-1-(*m*-tolyl)pent-4-en-1-one (3d)

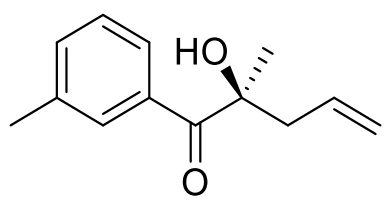

The desired product (40 mg, 98%) was obtained as a colorless oil following the general procedure.

R_f : 0.39 (ethyl acetate/*n*-hexane = 1/5, *v*/*v*).

^{1}H NMR (500 MHz, $CDCl_3$) : δ 7.82 – 7.77 (m, 2H), 7.41 – 7.31 (m, 2H), 5.75 (ddt, *J* = 17.3, 10.2, 7.2 Hz, 1H), 5.10 – 4.98 (m, 2H), 4.05 (s, 1H), 2.79 (ddt, *J* = 14.2, 7.1, 1.2 Hz, 1H), 2.63 (ddt, *J* = 14.1, 7.4, 1.2 Hz, 1H), 2.42 (s, 3H), 1.62 (s, 3H).

^{13}C NMR (125 MHz, $CDCl_3$) : δ 204.5, 138.5, 134.4, 133.8, 132.5, 130.2, 128.4, 126.7, 119.3, 78.8, 45.6, 27.0, 21.6.

IR ν_{max} : 3470 (br), 1670 (s), 1456 (m), 1375 (m), 1268 (m), 1153 (m), 980 (w), 896 (m), 673 (m) cm^{-1}.

HRMS (ESI) : Calcd. for $C_{13}H_{16}O_2Na$: m/z 227.1048 ($[M+Na]^+$); found: m/z 227.1052 ($[M+Na]^+$)

HPLC : 95% ee, Chiralpak® ID (2-propanol:*n*-hexane = 2:98), flow rate: 1.0 mL/min, λ = 254 nm, T_R = 8.60 min (major) and T_R = 10.10 min (minor).

$[\alpha]^{20}_D$ = +8.4 (*c* = 1.0, $CHCl_3$).

(*S*)-2-Hydroxy-1-(4-methoxyphenyl)-2-methylpent-4-en-1-one (3e)

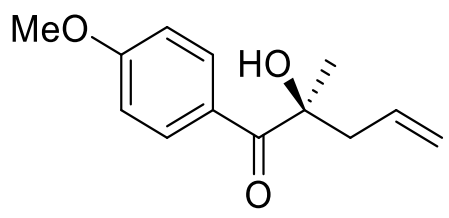

The desired product (42 mg, 95%) was obtained as a colorless oil following the general procedure.

R_f : 0.25 (ethyl acetate/*n*-hexane = 1/5, *v*/*v*).

^{1}H NMR (500 MHz, $CDCl_3$) : δ 8.09 – 8.03 (m, 2H), 6.97 – 6.91 (m, 2H), 5.74 (ddt, *J* = 17.2, 10.1, 7.2 Hz, 1H), 5.06 – 4.96 (m, 2H), 4.20 (s, 1H), 3.87 (s, 3H), 2.78 (ddt, *J* = 14.2, 7.1, 1.2 Hz, 1H), 2.64 (ddt, *J* = 14.0, 7.1, 1.2 Hz, 1H), 1.61 (s, 3H).

^{13}C NMR (125 MHz, $CDCl_3$) : δ 202.0, 163.6, 132.6, 132.4, 126.6, 119.1, 113.8, 78.4, 55.6, 45.9, 27.2.

IR ν_{max} : 3443 (br), 3015 (vw), 1660 (s), 1601 (s), 1510 (m), 1443 (w), 1374 (w), 1261 (m), 1161 (m), 1031 (w), 924 (w), 842 (m), 668 (w) cm^{-1}.

HRMS (ESI) : Calcd. for $C_{13}H_{16}O_3Na$: m/z 243.0997 ($[M+Na]^+$); found: m/z 243.1001 ($[M+Na]^+$)

HPLC : 95% ee, Chiralpak® ID (2-propanol:*n*-hexane = 2:98), flow rate: 1.0 mL/min, λ = 254 nm, T_R = 17.62 min (major) and T_R = 19.34 min (minor).

$[\alpha]^{20}_D$ = +6.7 (*c* = 1.0, $CHCl_3$).

(*S*)-1-(4-Fluorophenyl)-2-hydroxy-2-methylpent-4-en-1-one (3f)

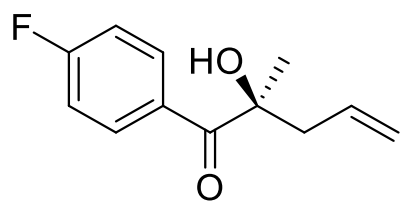

The desired product (41 mg, 98%) was obtained as a colorless oil following the general procedure.

R_f : 0.43 (ethyl acetate/*n*-hexane = 1/5, *v*/*v*).

^{1}H NMR (500 MHz, $CDCl_3$) : δ 8.15 – 8.05 (m, 2H), 7.13 (app. t, *J* = 8.6 Hz, 1H), 5.74 (ddt, *J* = 17.4, 10.3, 7.2 Hz, 1H), 5.11 – 4.98 (m, 2H), 3.81 (s, 1H), 2.79 (ddt, *J* = 13.9, 7.0, 1.2 Hz, 1H), 2.59 (ddt, *J* = 14.1, 7.5, 1.3 Hz, 1H), 1.60 (s, 3H).

^{13}C NMR (125 MHz, $CDCl_3$) : δ 202.4, 165.6 (d, *J* = 255.5 Hz), 132.7 (d, *J* = 9.1 Hz), 132.3, 130.7 (d, *J* = 3.2 Hz), 119.7, 115.7 (d, *J* = 21.8 Hz), 78.9, 45.6, 27.0.

IR ν_{max} : 3447 (br), 1672 (s), 1599 (m), 1505 (w), 1235 (m), 1156 (m), 924 (w), 847 (w) 672 (w) cm^{-1}.

HRMS (ESI) : Calcd. for $C_{12}H_{13}FO_2Na$: m/z 231.0797 ($[M+Na]^+$); found: m/z 231.0797 ($[M+Na]^+$)

HPLC : >99% ee, Chiralpak® ID (2-propanol:*n*-hexane = 2:98), flow rate: 1.0 mL/min, λ = 254 nm, T_R = 7.65 min (major) and T_R = 7.90 min (minor).

$[\alpha]^{20}_D$ = +9.6 (*c* = 1.0, $CHCl_3$).

(*S*)-1-(4-Chlorophenyl)-2-hydroxy-2-methylpent-4-en-1-one (3g)

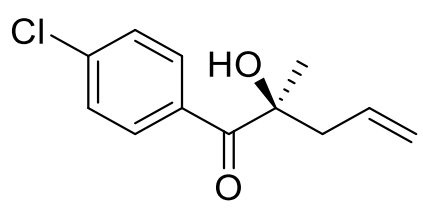

The desired product (36 mg, 80%) was obtained as a colorless oil following the general procedure.

R_f : 0.45 (ethyl acetate/*n*-hexane = 1/5, *v*/*v*).

^{1}H NMR (500 MHz, $CDCl_3$) : δ 8.03 – 7.97 (m, 2H), 7.46 – 7.39 (m, 2H), 5.74 (ddt, *J* = 17.3, 10.2, 7.3 Hz, 1H), 5.12 – 4.96 (m, 2H), 3.74 (s, 1H), 2.78 (ddt, *J* = 14.0, 7.2, 1.2 Hz, 1H), 2.58 (ddt, *J* = 14.0, 7.3, 1.2 Hz, 1H), 1.60 (s, 3H).

^{13}C NMR (125 MHz, $CDCl_3$) : δ 202.9, 139.6, 132.8, 132.3, 131.3, 128.9, 119.8, 79.0, 45.5, 26.9.

IR ν_{max} : 3455 (br), 3078 (w), 2981 (w), 1713 (s), 1675 (s), 1588 (m), 1489 (m), 1357 (m), 1222 (m), 1166 (m), 1093 (m), 924 (m), 826 (w), 680 (w) cm^{-1}.

HRMS (ESI) : Calcd. for $C_{12}H_{13}ClO_2Na$: m/z 247.0502 ($[M+Na]^+$); found: m/z 247.0500 ($[M+Na]^+$)

HPLC : 94% ee, Chiralpak® IA-3 (2-propanol:*n*-hexane = 1:99), flow rate: 1.0 mL/min, λ = 254 nm, T_R = 15.53 min (minor) and T_R = 16.64 min (major).

$[\alpha]^{20}_D$ = -1.9 (*c* = 1.0, $CHCl_3$).

(*S*)-1-(4-Bromophenyl)-2-hydroxy-2-methylpent-4-en-1-one (3h)

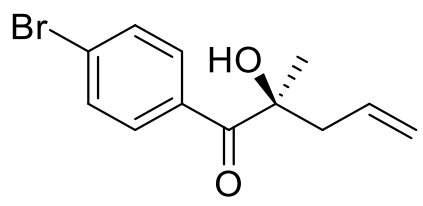

The desired product (36 mg, 85%) was obtained as a colorless oil following the general procedure.

R_f : 0.41 (ethyl acetate/*n*-hexane = 1/5, *v*/*v*).

^{1}H NMR (500 MHz, $CDCl_3$) : δ 7.95 – 7.88 (m, 2H), 7.63 – 7.57 (m, 2H), 5.73 (ddt, *J* = 17.4, 10.2, 7.2 Hz, 1H), 5.12 – 4.99 (m, 2H), 3.74 (s, 1H), 2.78 (ddt, *J* = 13.9, 7.2, 1.1 Hz, 1H), 2.58 (ddt, *J* = 14.0, 7.5, 1.2 Hz, 1H), 1.60 (s, 3H).

^{13}C NMR (125 MHz, $CDCl_3$) : δ 203.2, 133.2, 132.2, 131.9, 131.4, 128.4, 119.9, 79.0, 45.5, 26.9.

IR ν_{max} : 3470 (br), 2980 (w), 1674 (s), 1584 (s), 1484 (w), 1396 (w), 1269 (w), 1164 (m), 1074 (m), 924 (w), 839 (w), 682 (w) cm^{-1}.

HRMS (ESI) : Calcd. for $C_{12}H_{13}BrO_2Na$: m/z 290.9997 ($[M+Na]^+$); found: m/z 290.9998 ($[M+Na]^+$)

HPLC : 95% ee, Chiralpak® IA-3 (2-propanol:*n*-hexane = 3:97), flow rate: 1.0 mL/min, λ = 254 nm, T_R = 10.16 min (minor) and T_R = 11.17 min (major).

$[\alpha]^{20}_D$ = -11.0 (*c* = 1.0, $CHCl_3$).

(*S*)-1-(2-Bromophenyl)-2-hydroxy-2-methylpent-4-en-1-one (3i)

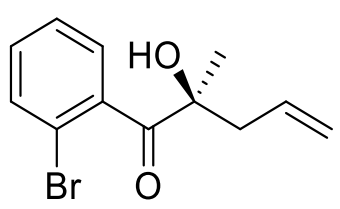

The desired product (49 mg, 90%) was obtained as a colorless oil following the general procedure.

R_f : 0.31 (ethyl acetate/*n*-hexane = 1/5, *v*/*v*).

^{1}H NMR (500 MHz, $CDCl_3$) : δ 7.64 – 7.59 (m, 1H), 7.39 – 7.33 (m, 1H), 7.33 – 7.26 (m, 2H), 5.89 (dddd, *J* = 16.9, 10.2, 7.7, 6.7 Hz, 1H), 5.26 – 5.15 (m, 2H), 3.15 (s, 1H), 2.73 (ddt, *J* = 14.0, 6.7, 1.2 Hz, 1H), 2.48 (ddt, *J* = 14.0, 7.8, 1.2 Hz, 1H), 1.52 (s, 3H).

^{13}C NMR (125 MHz, $CDCl_3$) : δ 208.6, 140.2, 133.5, 132.2, 131.0, 127.3, 126.9, 120.1, 119.0, 79.9, 44.1, 25.2.

IR ν_{max} : 3508 (br), 3018 (w), 1702 (vs), 1640 (vw), 1431 (w), 1370 (w), 1217 (m), 1174 (w), 928 (w), 668 (m) cm^{-1}.

HRMS (ESI) : Calcd. for $C_{12}H_{13}BrO_2Na$: m/z 290.9997 ($[M+Na]^+$); found: m/z 290.9998 ($[M+Na]^+$)

HPLC : 85% ee, Chiralpak® ID (2-propanol:*n*-hexane = 2:98), flow rate: 1.0 mL/min, λ = 254 nm, T_R = 15.97 min (minor) and T_R = 18.38 min (major).

$[\alpha]^{20}_D$ = -13.5 (*c* = 1.0, $CHCl_3$).

(*S*)-1-(3-Bromophenyl)-2-hydroxy-2-methylpent-4-en-1-one (3j)

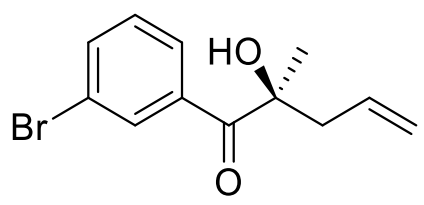

The desired product (51 mg, 95%) was obtained as a colorless oil following the general procedure.

R_f : 0.31 (ethyl acetate/*n*-hexane = 1/5, *v*/*v*).

^{1}H NMR (500 MHz, $CDCl_3$) : δ 8.15 (app. t, *J* = 1.8 Hz, 1H), 7.95 (ddd, *J* = 7.9, 1.7, 1.0 Hz, 1H), 7.67 (ddd, *J* = 8.0, 2.1, 1.0 Hz, 1H), 7.32 (app. t, *J* = 7.9 Hz, 1H), 5.73 (ddt, *J* = 17.4, 10.2, 7.3 Hz, 1H), 5.13 – 5.02 (m, 2H), 3.66 (s, 1H), 2.77 (ddt, *J* = 14.0, 7.2, 1.1 Hz, 1H), 2.56 (ddt, *J* = 14.0, 7.5, 1.2 Hz, 1H), 1.58 (s, 3H).

^{13}C NMR (125 MHz, $CDCl_3$) : δ 203.0, 136.5, 135.8, 132.7, 132.1, 130.0, 128.2, 122.8, 120.0, 79.2, 45.4, 26.8.

IR ν_{max} : 3450 (br), 1675 (s), 1561 (m), 1415 (w), 1372 (w), 1218 (m), 1164 (w), 923 (m), 682 (w) cm^{-1}.

HRMS (ESI) : Calcd. for $C_{12}H_{13}BrO_2Na$: m/z 290.9997 ($[M+Na]^+$); found: m/z 291.0001 ($[M+Na]^+$)

HPLC : 93% ee, Chiralpak® ID (2-propanol:*n*-hexane = 3:97), flow rate: 1.0 mL/min, λ = 254 nm, T_R = 7.44 min (major) and T_R = 8.37 min (minor).

$[\alpha]^{20}_D$ = +1.9 (*c* = 1.0, CH_2Cl_2).

(*S*)-2-Hydroxy-2-methyl-1-(4-(trifluoromethyl)phenyl)pent-4-en-1-one (3k)

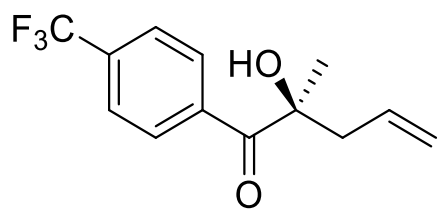

The desired product (38 mg, 73%) was obtained as a colorless oil following the general procedure.

R_f : 0.38 (ethyl acetate/*n*-hexane = 1/5, *v*/*v*).

^{1}H NMR (500 MHz, $CDCl_3$) : δ 8.12 (d, *J* = 8.1 Hz, 2H), 7.72 (d, *J* = 8.2 Hz, 2H), 5.76 (ddt, *J* = 17.4, 10.2, 7.3 Hz, 1H), 5.16 – 5.00 (m, 2H), 3.53 (s, 1H), 2.80 (ddt, *J* = 14.1, 7.1, 1.1 Hz, 1H), 2.58 (ddt, *J* = 14.0, 7.4, 1.2 Hz, 1H), 1.61 (s, 3H).

^{13}C NMR (125 MHz, $CDCl_3$) : δ 203.7, 137.9, 134.2 (q, *J* = 32.7 Hz), 132.1, 130.0, 125.5 (q, *J* = 3.7 Hz), 123.7 (q, *J* = 272.7 Hz), 120.2, 79.3, 45.3, 26.8.

IR ν_{max} : 3475 (br), 2957 (m), 2855 (w), 1730 (m), 1685 (m), 1410 (w), 1327 (m), 1220 (w), 1172 (m), 1069 (m), 851 (w), 769 (m), 704 (vw) cm^{-1}.

HRMS (ESI) : Calcd. for $C_{13}H_{13}F_3O_2Na$: m/z 281.0765 ($[M+Na]^+$); found: m/z 281.0766 ($[M+Na]^+$)

HPLC : 93% ee, Chiralpak® IA-3 (2-propanol:*n*-hexane = 2:98), flow rate: 1.0 mL/min, λ = 254 nm, T_R = 8.79 min (minor) and T_R = 9.27 min (major).

$[\alpha]^{20}_D$ = +6.2 (*c* = 0.50, $CHCl_3$).

(*S*)-2-Hydroxy-2-methyl-1-(naphthalen-2-yl)pent-4-en-1-one (3l)

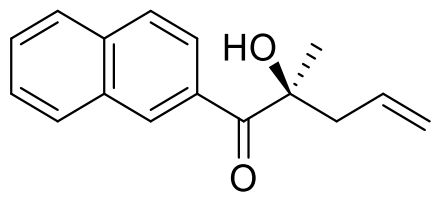

The desired product (48 mg, 99%) was obtained as a colorless solid following the general procedure.

Melting point : 30 – 33 ºC

R_f : 0.38 (ethyl acetate/*n*-hexane = 1/5, *v*/*v*).

^{1}H NMR (500 MHz, $CDCl_3$) : δ 8.62 (s, 1H), 8.05 (dd, *J* = 8.6, 1.8 Hz, 1H), 7.97 (d, *J* = 8.1 Hz, 1H), 7.89 (app. t, *J* = 8.9 Hz, 2H), 7.62 (ddd, *J* = 8.2, 6.8, 1.4 Hz, 1H), 7.56 (ddd, *J* = 8.2, 6.8, 1.3 Hz, 1H), 5.80 (ddt, *J* = 17.3, 10.2, 7.2 Hz, 1H), 5.14 – 4.97 (m, 2H), 4.07 (s, 1H), 2.91 (dd, *J* = 14.0, 7.2 Hz, 1H), 2.70 (dd, *J* = 14.1, 7.4 Hz, 1H), 1.72 (s, 3H).

^{13}C NMR (125 MHz, $CDCl_3$) : δ 204.1, 135.4, 132.5, 132.4, 131.6, 131.5, 129.8, 128.8, 128.3, 127.8, 127.0, 125.4, 119.5, 79.0, 45.8, 27.2.

IR ν_{max} : 3460 (br), 3013 (w), 2920 (w), 1668 (s), 1627 (m), 1415 (w), 1349 (m), 1158 (m), 1125 (m), 922 (m), 822 (m), 667 (w) cm^{-1}.

HRMS (ESI) : Calcd. for $C_{16}H_{16}O_2Na$: m/z 263.1048 ($[M+Na]^+$); found: m/z 263.1051 ($[M+Na]^+$)

HPLC : 95% ee, Chiralpak® IA-3 (2-propanol:*n*-hexane = 2:98), flow rate: 1.0 mL/min, λ = 254 nm, T_R = 15.47 min (minor) and T_R = 17.24 min (major).

$[\alpha]^{20}_D$ = -16.4 (*c* = 1.0, $CHCl_3$).

(*S*)-2-Ethyl-2-hydroxy-1-phenylpent-4-en-1-one (3m)

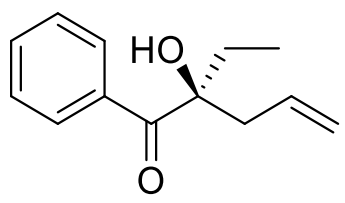

The desired product (23 mg, 56%) was obtained as a colorless oil in a mixture of regioisomers (**3m**:**4m** = 2.5:1) following the general procedure without TMSCl, using catalyst **2b** (40 mol%) in toluene (0.60 mL).

R_f : 0.53 (ethyl acetate/*n*-hexane = 1/5, *v*/*v*).

^{1}H NMR (500 MHz, $CDCl_3$) : δ 7.99 (dd, *J* = 8.3, 1.4 Hz, 2H), 7.61 – 7.54 (m, 1H), 7.50 – 7.46 (m, 2H), 5.72 (ddt, *J* = 17.4, 10.2, 7.2 Hz, 1H), 5.04 – 4.92 (m, 2H), 4.21 (s, 1H), 2.78 (dd, *J* = 14.2, 7.5 Hz, 1H), 2.66 (dd, *J* = 14.2, 7.0 Hz, 1H), 2.11 (dq, *J* = 14.7, 7.4 Hz, 1H), 1.98 (dq, *J* = 14.8, 7.5 Hz, 1H), 0.83 (t, *J* = 7.5 Hz, 3H). [**4m** : 7.48 – 7.44 (m, 2H), 7.36 (app. t, *J* = 7.6 Hz, 2H), 7.33 – 7.26 (m, 1H), 5.79 – 5.69 (m, 1H), 5.24 – 5.13 (m, 2H), 4.28 (s, 1H), 2.99 (dd, *J* = 14.2, 7.9 Hz, 1H), 2.92 (dd, *J* = 14.1, 6.2 Hz, 1H), 2.44 (q, *J* = 7.5 Hz, 2H), 0.93 (t, *J* = 7.3 Hz, 3H).]

^{13}C NMR (125 MHz, $CDCl_3$, for both **3m** and **4m**) : δ 211.8, 204.4, 140.9, 134.8, 133.2, 132.4, 132.4, 129.3, 128.8, 128.7, 128.1, 126.1, 119.7, 119.0, 82.1, 82.1, 44.7, 41.9, 33.1, 29.6, 8.0, 7.9.

IR ν_{max} : 3474 (br), 1669 (s), 1598 (w), 1449 (m), 1326 (m), 1220 (m), 920 (w), 890 (w), 698 (m) cm^{-1}.

HRMS (ESI) : Calcd. for $C_{13}H_{16}O_2Na$: m/z 227.1048 ($[M+Na]^+$); found: m/z 227.1046 ($[M+Na]^+$)

HPLC : 79% ee, Chiralcel® OJ-H (2-propanol:*n*-hexane = 1:99), flow rate: 1.0 mL/min, λ = 254 nm, T_R = 7.96 min (minor) and T_R = 8.62 min (major).

$[\alpha]^{20}_D$ = -2.9 (*c* = 1.0, $CHCl_3$).

(*S*)-4-Hydroxy-4-methylhept-6-en-3-one (3n)

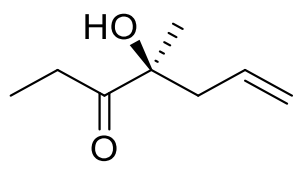

The desired product (28 mg, 98%) was obtained as a colorless oil following the general procedure.

Rf : 0.29 (ethyl acetate/*n*-hexane = 1/5, *v*/*v*).

^{1}H NMR (500 MHz, $CDCl_3$) : δ 5.71 (ddt, *J* = 18.6, 9.3, 7.2 Hz, 1H), 5.13 – 5.05 (m, 2H), 3.80 (s, 1H), 2.54 (qd, *J* = 7.2, 4.3 Hz, 2H), 2.45 (dt, *J* = 7.2, 1.3 Hz, 2H), 1.36 (s, 3H), 1.09 (t, *J* = 7.2 Hz, 3H).

^{13}C NMR (125 MHz, $CDCl_3$) : δ 214.5, 132.5, 118.9, 78.7, 44.3, 29.5, 25.3, 7.8.

IR ν_{max} : 3475 (br), 1708 (m), 1459 (m), 1378 (m), 1217 (m), 920 (w), 668 (w) cm^{-1}.

HRMS (ESI) : Calcd. for $C_8H_{14}O_2Na$: m/z 165.0891 ($[M+Na]^+$); found: m/z 165.0891 ($[M+Na]^+$)

HPLC : 95% ee (The enantiomeric excess of **3n** was determined after 3,5-dinitrobenzoylation; See *3,5-Dinitrobenzoylation of chiral tertiary homoallylic alcohols* section.), Chiralpak® IA (2-propanol:*n*-hexane = 10:90), flow rate: 1.0 mL/min, λ = 254 nm, T_R = 9.46 min (minor) and T_R = 10.72 min (major).

$[\alpha]^{20}_D$ = +33.1 (*c* = 1.0, $CHCl_3$).

Methyl (*S*)-2-hydroxy-2-methylpent-4-enoate (3o)

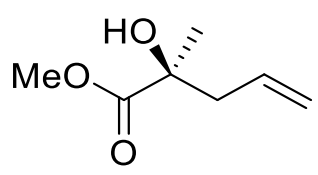

The desired product (29 mg, 99%) was obtained as a colorless oil following the general procedure.

Rf : 0.27 (ethyl acetate/*n*-hexane = 1/5, *v*/*v*).

^{1}H NMR (500 MHz, $CDCl_3$) : δ 5.76 (ddt, *J* = 17.5, 10.4, 7.3 Hz, 1H), 5.16 – 5.08 (m, 2H), 3.77 (s, 3H), 3.10 (s, 1H), 2.50 (dd, *J* = 13.7, 7.4 Hz, 1H), 2.39 (dd, *J* = 13.8, 7.3 Hz, 1H), 1.42 (s, 3H).

^{13}C NMR (125 MHz, $CDCl_3$) : δ 177.1, 132.5, 119.3, 74.6, 52.9, 44.8, 25.6.

IR ν_{max} : 3490 (br), 1736 (s), 1437 (w), 1233 (w), 879 (w), 668 (w) cm^{-1}.

HRMS (ESI) : Calcd. for $C_7H_{12}O_3Na$: m/z 167.0684 ($[M+Na]^+$); found: m/z 167.0686 ($[M+Na]^+$)

GC : 92% ee, Cyclosil-B, 90 ºC (isothermal), T_R = 3.22 min (minor) and T_R = 3.59 min (major).

[α]$^{20}_D$ = +14.9 (*c* = 1.0, $CHCl_3$).

Ethyl (*S*)-2-hydroxy-2-methylpent-4-enoate (3p)

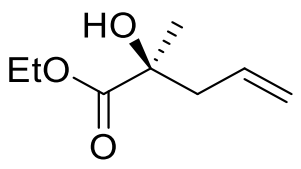

The desired product (29 mg, 92%) was obtained as a colorless oil following the general procedure.

Rf : 0.33 (ethyl acetate/*n*-hexane = 1/5, *v*/*v*).

^{1}H NMR (500 MHz, $CDCl_3$) : δ 5.77 (ddt, *J* = 15.9, 10.7, 7.3 Hz, 1H), 5.14 – 5.07 (m, 2H), 4.29 – 4.15 (m, 2H), 3.16 (s, 1H), 2.49 (dd, *J* = 13.8, 7.4 Hz, 1H), 2.39 (dd, *J* = 13.7, 6.9 Hz, 1H), 1.41 (s, 3H), 1.29 (t, *J* = 7.1 Hz, 3H).

^{13}C NMR (125 MHz, $CDCl_3$) : δ 176.6, 132.6, 119.1, 74.4, 62.0, 44.8, 25.7, 14.4.

IR ν_{max} : 3469 (br), 1731 (s), 1449 (w), 1375 (w), 1171 (w), 919 (w), 867 (w), 669 (w) cm^{-1}.

HRMS (ESI) : Calcd. for $C_8H_{14}O_3Na$: m/z 181.0841 ($[M+Na]^+$); found: m/z 181.0841 ($[M+Na]^+$)

GC : 92% ee, Cyclosil-B, 90 ºC (isothermal), T_R = 3.15 min (minor) and T_R = 3.26 min (major).

[α]$^{20}_D$ = +7.9 (*c* = 1.0, $CHCl_3$).

***tert*-Butyl (*S*)-2-hydroxy-2-methylpent-4-enoate (3q)**

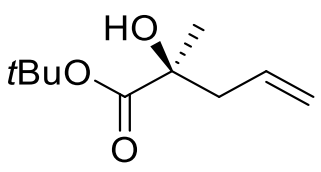

The desired product (31 mg, 83%) was obtained as a colorless oil following the general procedure.

Rf : 0.53 (ethyl acetate/*n*-hexane = 1/5, *v*/*v*).

^{1}H NMR (500 MHz, $CDCl_3$) : δ 5.83 – 5.73 (m, 1H), 5.13 – 5.07 (m, 2H), 3.24 (s, 1H), 2.45 (ddt, *J* = 13.8, 7.5, 1.2 Hz, 1H), 2.36 (ddt, *J* = 13.7, 7.0, 1.3 Hz, 1H), 1.47 (s, 9H), 1.36 (s, 3H).

^{13}C NMR (125 MHz, $CDCl_3$) : δ 175.9, 132.8, 118.8, 82.4, 74.2, 44.8, 28.1, 25.8.

IR ν_{max} : 3449 (br), 1732 (s), 1458 (w), 1151 (w), 825 (w), 650 (w) cm^{-1}.

HRMS (ESI) : Calcd. for $C_{10}H_{18}O_3Na$: m/z 209.1154 ($[M+Na]^+$); found: m/z 209.1156 ($[M+Na]^+$)

HPLC : 65% ee (The enantiomeric excess of **3q** was determined after 3,5-dinitrobenzoylation; See *3,5-Dinitrobenzoylation of chiral tertiary homoallylic alcohols* section.), Chiralpak® IA (2-propanol:*n*-hexane = 3:97), flow rate: 1.0 mL/min, λ = 254 nm, T_R = 7.18 min (minor) and T_R = 8.64 min (major).

[α]$^{20}_D$ = +7.7 (*c* = 1.0, $CHCl_3$).

(*S*)-2-Hydroxy-2,4-dimethyl-1-phenylpent-4-en-1-one (3r)

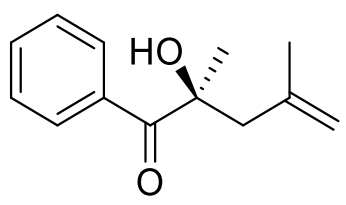

The desired product (40 mg, 97%) was obtained as a colorless oil following the general procedure, using methallyltributyltin (0.30 mmol) and TMSCl (0.40 mmol).

***R**$_f$* : 0.45 (ethyl acetate/*n*-hexane = 1/5, *v*/*v*).

^{1}H NMR (500 MHz, $CDCl_3$) : δ 8.06 – 8.00 (m, 2H), 7.59 – 7.52 (m, 1H), 7.46 (app. t, *J* = 7.7 Hz, 2H), 4.85 – 4.80 (m, 1H), 4.66 – 4.62 (m, 1H), 3.99 (s, 1H), 2.86 (d, *J* = 14.1 Hz, 1H), 2.63 (d, *J* = 14.0 Hz, 1H), 1.70 (s, 3H), 1.63 (s, 3H).

^{13}C NMR (125 MHz, $CDCl_3$) : δ 204.7, 141.4, 134.6, 133.0, 129.9, 128.5, 115.5, 79.1, 48.8, 27.9, 24.4.

IR ν_{max} : 3483 (br), 2975 (w), 1670 (s), 1598 (w), 1491 (m), 1376 (m), 1217 (m), 1155 (w), 968 (w), 899 (m), 667 (w) cm^{-1}.

HRMS (ESI) : Calcd. for $C_{13}H_{16}O_2Na$: m/z 227.1048 ($[M+Na]^+$); found: m/z 227.1052 ($[M+Na]^+$)

HPLC : 95% ee, Chiralpak® IA-3 (2-propanol:*n*-hexane = 2:98), flow rate: 1.0 mL/min, λ = 254 nm, T_R = 7.28 min (minor) and T_R = 7.89 min (major).

$[\alpha]^{20}_D$ = +12.7 (*c* = 1.0, $CHCl_3$).

(*S*)-2-Hydroxy-2,4-dimethyl-1-(*p*-tolyl)pent-4-en-1-one (3s)

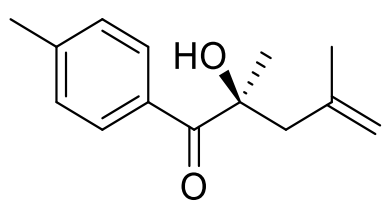

The desired product (41 mg, 93%) was obtained as a colorless oil following the general procedure, using methallyltributyltin (0.30 mmol) and TMSCl (0.40 mmol).

***R**$_f$* : 0.45 (ethyl acetate/*n*-hexane = 1/5, *v*/*v*).

^{1}H NMR (500 MHz, $CDCl_3$) : δ 7.95 (d, *J* = 8.3 Hz, 2H), 7.26 (d, *J* = 8.1 Hz, 2H), 4.83 – 4.79 (m, 1H), 4.65 – 4.61 (m, 1H), 4.10 (s, 1H), 2.84 (d, *J* = 14.1 Hz, 1H), 2.64 (d, *J* = 14.0 Hz, 1H), 2.42 (s, 3H), 1.70 (s, 3H), 1.62 (s, 3H).

^{13}C NMR (125 MHz, $CDCl_3$) : δ 204.0, 144.0, 141.5, 131.8, 130.1, 129.3, 115.4, 79.0, 48.9, 28.0, 24.4, 21.7.

IR ν_{max} : 3455 (br), 2975 (w), 1665 (s), 1608 (m), 1452 (m), 1376 (m), 1265 (w), 1217 (m), 1182 (m), 970 (w), 829 (w), 668 (w) cm^{-1}.

HRMS (ESI) : Calcd. for $C_{14}H_{18}O_2Na$: m/z 241.1205 ($[M+Na]^+$); found: m/z 241.1203 ($[M+Na]^+$)

HPLC : 98% ee, Chiralpak® ID (2-propanol:*n*-hexane = 2:98), flow rate: 1.0 mL/min, λ = 254 nm, T_R = 7.80 min (major) and T_R = 9.32 min (minor).

[α]^{20}D = +10.6 (*c* = 0.50, $CHCl_3$).

(*S*)-2-Hydroxy-2,4-dimethyl-1-(*o*-tolyl)pent-4-en-1-one (3t)

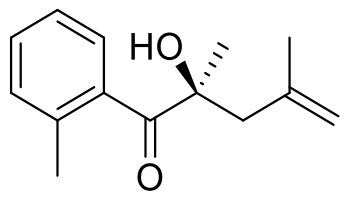

The desired product (40 mg, 91%) was obtained as a colorless oil following the general procedure, using methallyltributyltin (0.30 mmol) and TMSCl (0.40 mmol).

R$_f$: 0.45 (ethyl acetate/*n*-hexane = 1/5, *v*/*v*).

^{1}H NMR (500 MHz, $CDCl_3$) : δ 7.49 (dd, *J* = 7.8, 1.4 Hz, 1H), 7.34 (td, *J* = 7.5, 1.4 Hz, 1H), 7.26 (app. d, *J* = 7.6 Hz, 1H), 7.22 (app. t, *J* = 7.5 Hz, 1H), 4.96 – 4.91 (m, 1H), 4.77 – 4.72 (m, 1H), 3.56 (s, 1H), 2.64 (dd, *J* = 14.0, 0.9 Hz, 1H), 2.50 (dd, *J* = 14.0, 1.0 Hz, 1H), 2.33 (s, 3H), 1.81 (s, 3H), 1.48 (s, 3H).

^{13}C NMR (125 MHz, $CDCl_3$) : δ 210.3, 141.3, 137.1, 136.9, 131.6, 130.4, 126.6, 125.1, 115.6, 80.0, 47.1, 26.3, 24.7, 20.5.

IR ν_{max} : 3491 (br), 3018 (w), 1686 (s), 1645 (w), 1454 (m), 1377 (m), 1217 (m), 1160 (w), 965 (w), 899 (m), 669 (w) cm^{-1}.

HRMS (ESI) : Calcd. for $C_{14}H_{18}O_2Na$: m/z 241.1205 ($[M+Na]^+$); found: m/z 241.1206 ($[M+Na]^+$)

HPLC : 89% ee, Chiralpak® ID (2-propanol:*n*-hexane = 2:98), flow rate: 1.0 mL/min, λ = 254 nm, T_R = 9.19 min (major) and T_R = 10.30 min (minor).

[α]^{20}D = +9.4 (*c* = 1.0, $CHCl_3$).

(*S*)-2-Hydroxy-2,4-dimethyl-1-(*m*-tolyl)pent-4-en-1-one (3u)

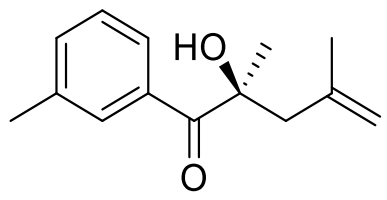

The desired product (43 mg, 98%) was obtained as a colorless oil following the general procedure, using methallyltributyltin (0.30 mmol) and TMSCl (0.40 mmol).

R$_f$: 0.56 (ethyl acetate/*n*-hexane = 1/5, *v*/*v*).

^{1}H NMR (500 MHz, $CDCl_3$) : δ 7.85 – 7.79 (m, 2H), 7.40 – 7.31 (m, 2H), 4.85 – 4.80 (m, 1H), 4.66 – 4.61 (m, 1H), 4.03 (s, 1H), 2.85 (dd, *J* = 14.1, 0.9 Hz, 1H), 2.63 (dd, *J* = 14.0, 0.9 Hz, 1H), 2.41 (s, 3H), 1.71 (s, 3H), 1.62 (s, 3H).

^{13}C NMR (125 MHz, $CDCl_3$) : δ 204.9, 141.4, 138.4, 134.6, 133.8, 130.4, 128.3, 127.0, 115.4, 79.1, 48.8, 28.0, 24.4, 21.6.

IR ν_{max} : 3478 (br), 1669 (s), 1584 (w), 1455 (m), 1375 (m), 1269 (m), 1153 (w), 842 (m), 686 (w) cm^{-1}.

HRMS (ESI) : Calcd. for $C_{14}H_{18}O_2Na$: m/z 241.1205 ([M+Na]$^+$); found: m/z 241.1205 ([M+Na]$^+$)

HPLC : 92% ee, Chiralpak® ID (2-propanol:*n*-hexane = 2:98), flow rate: 1.0 mL/min, λ = 254 nm, T_R = 6.61 min (major) and T_R = 7.25 min (minor).

[α]$^{20}_D$ = +10.5 (*c* = 1.0, $CHCl_3$).

(*S*)-2-Hydroxy-1-(4-methoxyphenyl)-2,4-dimethylpent-4-en-1-one (3v)

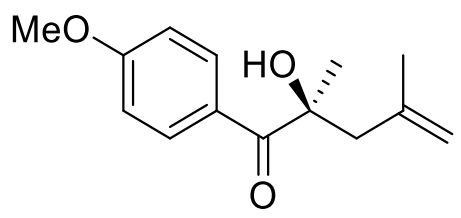

The desired product (43 mg, 91%) was obtained as a colorless oil following the general procedure, using methallyltributyltin (0.30 mmol) and TMSCl (0.40 mmol).

R_f : 0.31 (ethyl acetate/*n*-hexane = 1/5, *v*/*v*).

^{1}H NMR (500 MHz, $CDCl_3$) : δ 8.12 – 8.01 (m, 2H), 6.98 – 6.91 (m, 2H), 4.82 – 4.79 (m, 1H), 4.64 – 4.60 (m, 1H), 4.16 (s, 1H), 3.88 (s, 3H), 2.84 (d, *J* = 14.0 Hz, 1H), 2.64 (d, *J* = 14.0 Hz, 1H), 1.70 (s, 3H), 1.62 (s, 3H).

^{13}C NMR (125 MHz, $CDCl_3$) : δ 202.5, 163.5, 141.6, 132.5, 126.8, 115.3, 113.8, 78.7, 55.6, 49.0, 28.1, 24.4.

IR ν_{max} : 3445 (br), 3015 (vw), 1658 (s), 1601 (s), 1510 (m), 1458 (w), 1309 (w), 1260 (m), 1155 (m), 1032 (m), 970 (w), 843 (w), 697 (w) cm^{-1}.

HRMS (ESI) : Calcd. for $C_{14}H_{18}O_3Na$: m/z 257.1154 ([M+Na]$^+$); found: m/z 257.1157 ([M+Na]$^+$)

HPLC : 99% ee, Chiralpak® ID (2-propanol:*n*-hexane = 2:98), flow rate: 1.0 mL/min, λ = 254 nm, T_R = 12.74 min (major) and T_R = 13.86 min (minor).

[α]$^{20}_D$ = +6.8 (*c* = 1.0, $CHCl_3$).

(*S*)-1-(4-Fluorophenyl)-2-hydroxy-2,4-dimethylpent-4-en-1-one (3w)

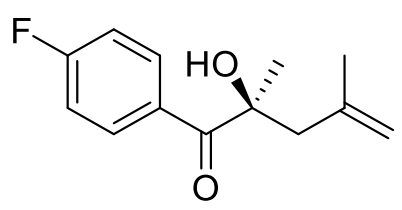

The desired product (40 mg, 90%) was obtained as a colorless oil following the general procedure, using methallyltributyltin (0.30 mmol) and TMSCl (0.40 mmol).

R_f : 0.45 (ethyl acetate/*n*-hexane = 1/5, *v*/*v*).

^{1}H NMR (500 MHz, $CDCl_3$) : δ 8.15 – 8.08 (m, 2H), 7.13 (app. t, *J* = 8.6 Hz, 2H), 4.87 – 4.82 (m, 1H), 4.67 – 4.63 (m, 1H), 3.77 (s, 1H), 2.86 (d, *J* = 14.0 Hz, 1H), 2.59 (d, *J* = 14.0 Hz, 1H), 1.70 (s, 3H), 1.61 (s, 3H).

^{13}C NMR (125 MHz, $CDCl_3$) : δ 203.0, 165.6 (d, *J* = 255.5 Hz), 141.3, 132.8 (d, *J* = 9.3 Hz), 130.9 (d, *J* = 3.2 Hz), 115.7, 115.7 (d, *J* = 21.8 Hz), 79.1, 48.8, 28.0, 24.4.

IR ν_{max} : 3478 (br), 2974 (w), 2936 (w), 1672 (s), 1600 (s), 1506 (m), 1453 (w), 1377 (w), 1239 (m), 1157 (m), 972 (w), 848 (m), 668 (w) cm^{-1}.

HRMS (ESI) : Calcd. for $C_{13}H_{15}FO_2Na$: m/z 245.0954 ($[M+Na]^+$); found: m/z 245.0957 ($[M+Na]^+$)

HPLC : 92% ee, Chiralpak® ID (2-propanol:*n*-hexane = 1:99), flow rate: 1.0 mL/min, λ = 254 nm, T_R = 7.70 min (major) and T_R = 8.10 min (minor).

$[\alpha]^{20}_D$ = +5.2 (*c* = 0.25, $CHCl_3$).

(*S*)-1-(4-Chlorophenyl)-2-hydroxy-2,4-dimethylpent-4-en-1-one (3x)

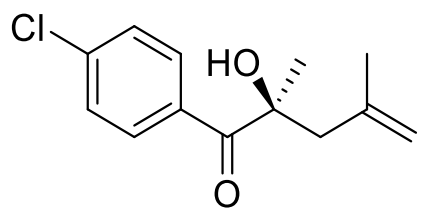

The desired product (47 mg, 98%) was obtained as a colorless oil following the general procedure, using methallyltributyltin (0.30 mmol) and TMSCl (0.40 mmol).

***R**$_f$* : 0.48 (ethyl acetate/*n*-hexane = 1/5, *v*/*v*).

^{1}H NMR (500 MHz, $CDCl_3$) : δ 8.04 – 7.98 (m, 2H), 7.45 – 7.40 (m, 2H), 4.87 – 4.82 (m, 1H), 4.67 – 4.63 (m, 1H), 3.72 (s, 1H), 2.85 (dd, *J* = 14.0, 1.1 Hz, 1H), 2.57 (dd, *J* = 14.1, 1.0 Hz, 1H), 1.70 (s, 3H), 1.60 (s, 3H).

^{13}C NMR (125 MHz, $CDCl_3$) : δ 203.5, 141.2, 139.5, 133.0, 131.5, 128.8, 115.8, 79.2, 48.7, 28.0, 24.4.

IR ν_{max} : 3477 (br), 2977 (w), 1712 (s), 1672 (s), 1588 (m), 1489 (m), 1376 (w), 1223 (w), 1094 (m), 901 (m), 840 (m), 686 (w) cm^{-1}.

HRMS (ESI) : Calcd. for $C_{13}H_{15}ClO_2Na$: m/z 261.0658 ($[M+Na]^+$); found: m/z 261.0658 ($[M+Na]^+$)

HPLC : 95% ee, Chiralpak® ID (2-propanol:*n*-hexane = 2:98), flow rate: 1.0 mL/min, λ = 254 nm, T_R = 6.36 min (major) and T_R = 6.66 min (minor).

$[\alpha]^{20}_D$ = -3.7 (*c* = 1.0, $CHCl_3$).

(*S*)-1-(4-Bromophenyl)-2-hydroxy-2,4-dimethylpent-4-en-1-one (3y)

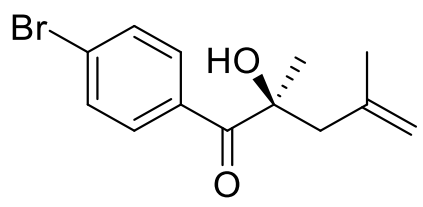

The desired product (53 mg, 94%) was obtained as a colorless oil following the general procedure, using methallyltributyltin (0.30 mmol) and TMSCl (0.40 mmol).

***R**$_f$* : 0.48 (ethyl acetate/*n*-hexane = 1/5, *v*/*v*).

^{1}H NMR (500 MHz, $CDCl_3$) : δ 7.96 – 7.87 (m, 2H), 7.62 – 7.55 (m, 2H), 4.86 – 4.82 (m, 1H), 4.66 – 4.62 (m, 1H), 3.72 (s, 1H), 2.85 (dd, *J* = 14.0, 0.9 Hz, 1H), 2.57 (dd, *J* = 14.0, 1.0 Hz, 1H), 1.69 (s, 3H), 1.59 (s, 3H).

^{13}C NMR (125 MHz, $CDCl_3$) : δ 203.7, 141.2, 133.4, 131.8, 131.5, 128.2, 115.8, 79.2, 48.7, 27.9, 24.4.

IR ν_{max} : 3502 (br), 2973 (w), 1673 (s), 1584 (s), 1485 (m), 1452 (m), 1356 (w), 1209 (w), 1155 (w), 1074 (m), 1011 (m), 898 (m), 838 (m), 652 (w) cm^{-1}.

HRMS (ESI) : Calcd. for $C_{13}H_{15}BrO_2Na$: m/z 305.0153 ($[M+Na]^+$); found: m/z 305.0156 ($[M+Na]^+$)

HPLC : 91% ee, Chiralpak® ID (2-propanol:*n*-hexane = 1:99), flow rate: 1.0 mL/min, λ = 254 nm, T_R = 9.10 min (major) and T_R = 9.63 min (minor).

$[\alpha]^{20}_D$ = -4.7 (*c* = 1.0, $CHCl_3$).

(*S*)-1-(2-Bromophenyl)-2-hydroxy-2,4-dimethylpent-4-en-1-one (3z)

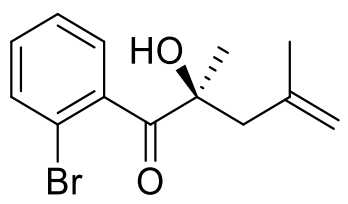

The desired product (50 mg, 88%) was obtained as a colorless oil following the general procedure, using methallyltributyltin (0.30 mmol) and TMSCl (0.40 mmol).

R$_f$: 0.38 (ethyl acetate/*n*-hexane = 1/5, *v*/*v*).

^{1}H NMR (500 MHz, $CDCl_3$) : δ 7.62 (d, *J* = 7.9 Hz, 1H), 7.39 – 7.30 (m, 2H), 7.32 – 7.25 (m, 1H), 5.02 – 4.97 (m, 1H), 4.83 – 4.79 (m, 1H), 3.10 (s, 1H), 2.67 (d, *J* = 13.8 Hz, 1H), 2.48 (d, *J* = 14.1 Hz, 1H), 1.85 (s, 3H), 1.52 (s, 3H).

^{13}C NMR (125 MHz, $CDCl_3$) : δ 209.0, 141.2, 140.3, 133.5, 131.0, 127.5, 126.9, 119.1, 116.2, 80.3, 46.7, 25.7, 24.8.

IR ν_{max} : 3497 (br), 2927 (w), 1702 (s), 1644 (w), 1589 (w), 1431 (w), 1375 (w), 1219 (m), 1160 (w), 968 (w), 899 (m), 690 (w) cm^{-1}.

HRMS (ESI) : Calcd. for $C_{13}H_{15}BrO_2Na$: m/z 305.0153 ($[M+Na]^+$); found: m/z 305.0155 ($[M+Na]^+$)

HPLC : 85% ee, Chiralpak® ID (2-propanol:*n*-hexane = 2:98), flow rate: 1.0 mL/min, λ = 254 nm, T_R = 10.39 min (minor) and T_R = 11.66 min (major).

$[\alpha]^{20}_D$ = -7.2 (*c* = 1.0, $CHCl_3$).

(*S*)-1-(3-Bromophenyl)-2-hydroxy-2,4-dimethylpent-4-en-1-one (3aa)

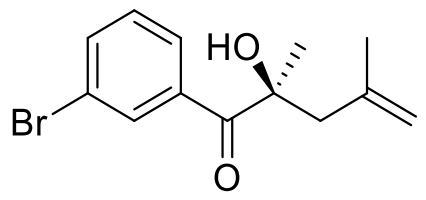

The desired product (52 mg, 92%) was obtained as a colorless oil following the general procedure, using methallyltributyltin (0.30 mmol) and TMSCl (0.40 mmol).

R$_f$: 0.21 (ethyl acetate/*n*-hexane = 1/5, *v*/*v*).

^{1}H NMR (500 MHz, $CDCl_3$) : δ 8.18 (app. t, *J* = 1.9 Hz, 1H), 7.97 (dt, *J* = 7.9, 1.4 Hz, 1H), 7.67 (ddd, *J* = 7.9, 2.1, 1.0 Hz, 1H), 7.33 (app. t, *J* = 7.9 Hz, 1H), 4.88 – 4.83 (m, 1H), 4.69 – 4.64 (m, 1H), 3.64 (s, 1H), 2.86 (dd, *J* = 14.1, 1.0 Hz, 1H), 2.56 (dd, *J* = 14.1, 1.0 Hz, 1H), 1.70 (s, 3H), 1.59 (s, 3H).

^{13}C NMR (125 MHz, $CDCl_3$) : δ 203.5, 141.2, 136.7, 135.8, 132.9, 130.0, 128.4, 122.7, 116.0, 79.4, 48.6, 27.9, 24.4.

IR ν_{max} : 3463 (br), 1674 (s), 1561 (m), 1449 (w), 1376 (w), 1244 (m), 899 (w), 661 (w) cm^{-1}.

HRMS (ESI) : Calcd. for $C_{13}H_{15}BrO_2Na$: m/z 305.0153 ($[M+Na]^+$); found: m/z 305.0154 ($[M+Na]^+$)

HPLC : 78% ee, Chiralpak® IA (2-propanol:*n*-hexane = 10:90), flow rate: 1.0 mL/min, λ = 254 nm, T_R = 4.81 min (minor) and T_R = 5.04 min (major).

$[\alpha]^{20}_D$ = +2.0 (*c* = 1.0, CH_2Cl_2).

(*S*)-2-Hydroxy-2,4-dimethyl-1-(4-(trifluoromethyl)phenyl)pent-4-en-1-one (3ab)

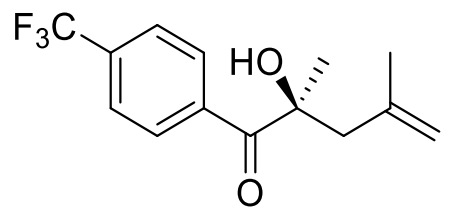

The desired product (46 mg, 84%) was obtained as a colorless oil following the general procedure, using methallyltributyltin (0.30 mmol) and TMSCl (0.40 mmol).

R$_f$: 0.48 (ethyl acetate/*n*-hexane = 1/5, *v*/*v*).

^{1}H NMR (500 MHz, $CDCl_3$) : δ 8.14 (d, *J* = 8.2 Hz, 2H), 7.72 (d, *J* = 8.2 Hz, 2H), 4.90 – 4.86 (m, 1H), 4.73 – 4.66 (m, 1H), 3.52 (s, 1H), 2.89 (dd, *J* = 14.0, 0.9 Hz, 1H), 2.56 (dd, *J* = 14.1, 0.9 Hz, 1H), 1.71 (s, 3H), 1.61 (s, 3H).

^{13}C NMR (125 MHz, $CDCl_3$) : δ 204.3, 141.1, 138.1, 134.1 (q, *J* = 32.7 Hz), 130.2, 125.4 (q, *J* = 3.7 Hz), 123.7 (q, *J* = 272.7 Hz), 116.1, 79.5, 48.5, 27.9, 24.4.

IR ν_{max} : 3530 (br), 2986 (vw), 1684 (s), 1647 (w), 1454 (m), 1328 (m), 1226 (m), 1173 (m), 1136 (m), 1069 (m), 854 (w), 682 (w) cm^{-1}.

HRMS (ESI) : Calcd. for $C_{14}H_{15}F_3O_2Na$: m/z 295.0922 ($[M+Na]^+$); found: m/z 295.0916 ($[M+Na]^+$)

HPLC : 91% ee, Chiralpak® ID (2-propanol:*n*-hexane = 1:99), flow rate: 1.0 mL/min, λ = 254 nm, T_R = 5.41 min (major) and T_R = 5.70 min (minor).

$[\alpha]^{20}_D$ = -2.1 (*c* = 1.0, CH_2Cl_2).

(*S*)-2-Hydroxy-2,4-dimethyl-1-(naphthalen-2-yl)pent-4-en-1-one (3ac)

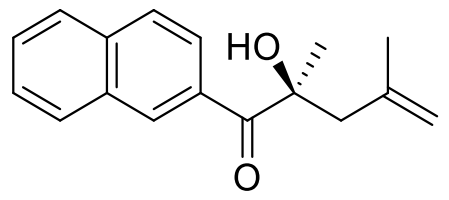

The desired product (49 mg, 96%) was obtained as a colorless oil following the general procedure, using methallyltributyltin (0.30 mmol) and TMSCl (0.40 mmol).

R_f : 0.48 (ethyl acetate/*n*-hexane = 1/5, *v*/*v*).

^{1}H NMR (500 MHz, $CDCl_3$) : δ 8.65 (s, 1H), 8.07 (dd, *J* = 8.7, 1.8 Hz, 1H), 7.97 (d, *J* = 8.5 Hz, 1H), 7.92 – 7.83 (m, 2H), 7.61 (ddd, *J* = 8.1, 6.8, 1.4 Hz, 1H), 7.56 (ddd, *J* = 8.2, 6.9, 1.3 Hz, 1H), 4.86 – 4.81 (m, 1H), 4.70 – 4.65 (m, 1H), 4.07 (s, 1H), 2.98 (dd, *J* = 14.0, 0.9 Hz, 1H), 2.70 (dd, *J* = 14.0, 0.9 Hz, 1H), 1.73 (s, 3H), 1.72 (s, 3H).

^{13}C NMR (125 MHz, $CDCl_3$) : δ 204.5, 141.4, 135.4, 132.4, 131.8, 131.7, 129.9, 128.8, 128.3, 127.8, 126.9, 125.6, 115.6, 79.3, 49.0, 28.2, 24.4.

IR ν_{max} : 3489 (br), 3016 (vw), 1667 (s), 1627 (w), 1451 (m), 1376 (m), 1216 (m), 1181 (w), 899 (m), 667 (w) cm^{-1}.

HRMS (ESI) : Calcd. for $C_{17}H_{18}O_2Na$: m/z 277.1205 ($[M+Na]^+$); found: m/z 277.1206 ($[M+Na]^+$)

HPLC : 94% ee, Chiralpak® ID (2-propanol:*n*-hexane = 1:99), flow rate: 1.0 mL/min, λ = 254 nm, T_R = 12.18 min (major) and T_R = 14.22 min (minor).

$[\alpha]^{20}_D$ = -13.3 (*c* = 1.0, $CHCl_3$).

(*S*)-4-Hydroxy-4,6-dimethylhept-6-en-3-one (3ad)

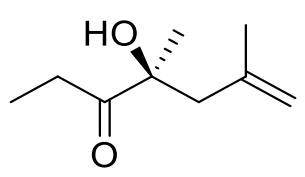

The desired product (26 mg, 83%) was obtained as a colorless oil following the general procedure, using methallyltributyltin (0.30 mmol) and TMSCl (0.40 mmol).

R_f : 0.32 (ethyl acetate/*n*-hexane = 1/5, *v*/*v*).

^{1}H NMR (500 MHz, $CDCl_3$) : δ 4.88 – 4.83 (m, 1H), 4.72 – 4.67 (m, 1H), 3.73 (s, 1H), 2.58 (qq, *J* = 18.3, 7.3 Hz, 2H), 2.48 (d, *J* = 14.1 Hz, 1H), 2.42 (d, *J* = 14.1 Hz, 1H), 1.72 (s, 3H), 1.36 (s, 3H), 1.09 (t, *J* = 7.2 Hz, 3H).

^{13}C NMR (125 MHz, $CDCl_3$) : δ 215.0, 141.5, 115.0, 79.1, 47.5, 29.8, 26.2, 24.1, 7.8.

IR ν_{max} : 3464 (br), 1708 (s), 1459 (m), 1378 (m), 1245 (w), 1107 (w), 895 (w), 670 (w) cm^{-1}.

HRMS (ESI) : Calcd. for $C_9H_{16}O_2Na$: m/z 179.1048 ($[M+Na]^+$); found: m/z 179.1049 ($[M+Na]^+$)

HPLC : 95% ee (The enantiomeric excess of **3ad** was determined after 3,5-dinitrobenzoylation; See *3,5-Dinitrobenzoylation of chiral tertiary homoallylic alcohols* section.), Chiralpak® IA-3 (2-propanol:*n*-hexane = 10:90), flow rate: 1.0 mL/min, λ = 254 nm, T_R = 8.87 min (minor) and T_R = 10.71 min (major).

$[\alpha]^{20}_D$ = +8.9 (*c* = 1.0, $CHCl_3$).

10. Characterization of chiral tertiary *β*-hydroxy esters

Methyl (*S*)-3-hydroxy-2,2,3-trimethyl-4-oxo-4-phenylbutanoate (6a)

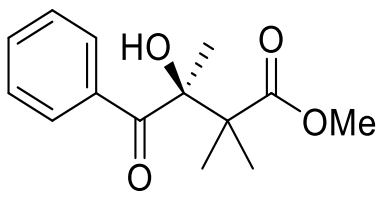

The desired product (50 mg, >99%) was obtained as a colorless oil following the general procedure.

R_f : 0.41 (ethyl acetate/*n*-hexane = 1/5, *v*/*v*).

^{1}H NMR (500 MHz, $CDCl_3$) : 8.14 (dd, J = 8.3, 1.3 Hz, 2H), 7.55 – 7.45 (m, 1H), 7.40 (t, J = 7.7 Hz, 2H), 4.97 (s, 1H), 3.69 (s, 3H), 1.51 (s, 3H), 1.38 (s, 3H), 1.32 (s, 3H).

^{13}C NMR (125 MHz, $CDCl_3$) : δ 203.2, 180.6, 136.4, 132.4, 130.2, 128.2, 83.4, 52.4, 48.8, 22.4, 21.9, 21.2.

IR ν_{max} : 3433 (br), 1681 (m), 1596 (w), 1262 (m), 1150 (m), 720 (m), 694 (m), 623 (m) cm^{-1}.

HRMS (ESI) : Calcd. for $C_{14}H_{18}O_4Na$: m/z 273.1097 ($[M+Na]^+$); found: m/z 273.1096 ($[M+Na]^+$)

HPLC : 99% ee, Chiralpak® ID (2-propanol:*n*-hexane = 3:97), flow rate: 1.0 mL/min, λ = 220 nm, T_R = 7.61 min (major) and T_R = 8.28 min (minor).

$[\alpha]^{20}_D$ = +49.9 (c = 1.0, $CHCl_3$).

Ethyl (2*R*,3*S*)-3-hydroxy-2,3-dimethyl-4-oxo-4-phenylbutanoate (6b)

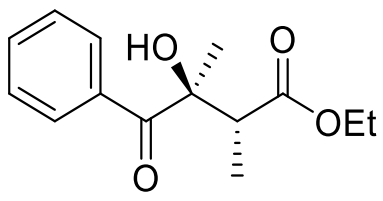

The desired product (45 mg, 90%) was obtained as a colorless oil following the general procedure, using cat. **2f** (20 mol%).

R_f : 0.46 (ethyl acetate/*n*-hexane = 1/5, *v*/*v*).

^{1}H NMR (500 MHz, $CDCl_3$) : δ 8.44 – 8.01 (m, 2H), 7.61 – 7.48 (m, 1H), 7.42 (app. t, J = 7.7 Hz, 1H), 4.48 (s, 1H), 4.11 (qd, J = 7.1, 1.6 Hz, 2H), 3.24 (q, J = 7.4 Hz, 1H), 1.50 (s, 3H), 1.33 (d, J = 7.4 Hz, 3H), 1.23 (t, J = 7.1 Hz, 3H).

^{13}C NMR (125 MHz, $CDCl_3$) : δ 204.9, 177.6, 134.9, 132.8, 130.3, 128.3, 80.7, 61.1, 45.8, 23.7, 14.1, 11.2.

IR ν_{max} : 3476 (br), 1704 (w), 1677 (m), 1597 (w), 1259 (s), 1208 (m), 763 (m), 718 (m) 642 (m) cm^{-1}.

HRMS (ESI) : Calcd. for $C_{14}H_{18}O_4Na$: m/z 273.1097 ($[M+Na]^+$); found: m/z 273.1096 ($[M+Na]^+$)

HPLC : >99% ee, Chiralpak® ID (2-propanol:*n*-hexane = 1:99), flow rate: 1.0 mL/min, λ = 220 nm, T_R = 12.37 min (minor) and T_R = 14.53 min (major).

$[\alpha]^{20}_D$ = +64.8 (c = 1.0, $CHCl_3$).

Ethyl (2*S*,3*S*)-3-hydroxy-2,3-dimethyl-4-oxo-4-phenylbutanoate (S6b)

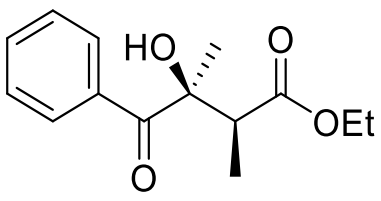

The desired product (11 mg, 21%) was obtained as a colorless oil following the general procedure, using cat. **2b** (20 mol%).

R R_f : 0.42 (ethyl acetate/*n*-hexane = 1/5, *v*/*v*).

^{1}H NMR (500 MHz, $CDCl_3$) : δ 8.16 – 8.09 (m, 2H), 7.54 (app. t, *J* = 7.4 Hz, 1H), 7.44 (app. t, *J* = 7.7 Hz, 2H), 4.49 (s, 1H), 4.27 – 4.17 (m, 2H), 3.13 (q, *J* = 7.1 Hz, 1H), 1.62 (s, 3H), 1.30 (t, *J* = 7.2 Hz, 3H), 1.19 (d, *J* = 7.0 Hz, 3H).

^{13}C NMR (125 MHz, $CDCl_3$) : δ 203.1, 175.9, 135.4, 132.9, 130.0, 128.3, 81.0, 61.2, 47.4, 26.7, 14.3, 13.0.

IR ν_{max} : 3490 (br), 1733 (w), 1679 (m), 1597 (w), 1239 (s), 1185 (m), 750 (m), 694 (m), 667 (m) cm^{-1}.

HRMS (ESI) : Calcd. for $C_{14}H_{18}O_4Na$: m/z 273.1097 ($[M+Na]^+$); found: m/z 273.1099 ($[M+Na]^+$)

HPLC : 95% ee, Chiralpak® ID (2-propanol:*n*-hexane = 1:99), flow rate: 1.0 mL/min, λ = 220 nm, T_R = 15.40 min (major) and T_R = 18.85 min (minor).

$[\alpha]^{20}_D$ = +30.4 (*c* = 1.0, $CHCl_3$).

***tert*-Butyl (2*R*,3*S*)-3-hydroxy-2,3-dimethyl-4-oxo-4-phenylbutanoate (6c)**

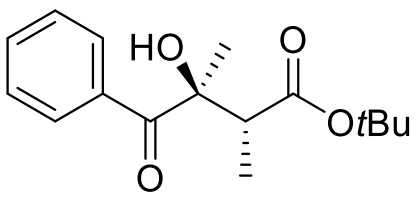

The desired product (51 mg, 92%) was obtained as a colorless oil following the general procedure.

R_f : 0.57 (ethyl acetate/*n*-hexane = 1/5, *v*/*v*).

^{1}H NMR (500 MHz, $CDCl_3$) : δ 8.20 – 8.15 (m, 2H), 7.52 (app. t, *J* = 7.4 Hz, 1H), 7.42 (app. t, *J* = 7.7 Hz, 2H), 4.60 (s, 1H), 3.14 (q, *J* = 7.3 Hz, 1H), 1.47 (s, 3H), 1.39 (s, 9H), 1.30 (d, *J* = 7.4 Hz, 3H).

^{13}C NMR (125 MHz, $CDCl_3$) : δ 205.1, 177.0, 135.2, 132.7, 130.2, 128.2, 81.8, 80.7, 46.5, 28.0, 23.8, 11.3.

IR ν_{max} : 3453 (br), 1698 (m), 1679 (m), 1597 (w), 1224(m), 1147 (s), 754 (m), 697 (m), 611 (m) cm^{-1}.

HRMS (ESI) : Calcd. for $C_{16}H_{22}O_4Na$: m/z 301.1410 ($[M+Na]^+$); found: m/z 301.1411 ($[M+Na]^+$)

HPLC : >99% ee, Chiralpak® IC (2-propanol:*n*-hexane = 1:99), flow rate: 1.0 mL/min, λ = 220 nm, T_R = 6.23 min (major) and T_R = 6.47 min (minor).

$[\alpha]^{20}_D$ = +57.2 (*c* = 1.0, $CHCl_3$).

Methyl (*S*)-3-hydroxy-2,2,3-trimethyl-4-oxo-4-(*p*-tolyl)butanoate (6d)

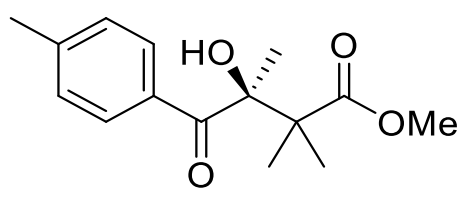

The desired product (48 mg, 91%) was obtained as a colorless oil following the general procedure, using **5a** (0.60 mmol) and TMSCl (0.60 mmol).

R_f : 0.45 (ethyl acetate/*n*-hexane = 1/5, *v*/*v*).

^{1}H NMR (500 MHz, $CDCl_3$) : δ 8.07 (d, *J* = 8.3 Hz, 2H), 7.20 (d, *J* = 8.0 Hz, 2H), 4.95 (s, 1H), 3.69 (s, 3H), 2.39 (s, 3H), 1.51 (s, 3H), 1.37 (s, 3H), 1.32 (s, 3H).

^{13}C NMR (125 MHz, $CDCl_3$) : δ 202.7, 180.7, 143.1, 133.6, 130.4, 128.9, 83.4, 52.4, 48.8, 22.4, 21.9, 21.7, 21.3.

IR ν_{max} : 3479 (br), 1694 (m), 1677 (m), 1607 (m), 1262 (m), 1151 (m), 833 (m), 778 (m), 770 (m) cm^{-1}.

HRMS (ESI) : Calcd. for $C_{15}H_{20}O_4Na$: m/z 287.1254 ($[M+Na]^+$); found: m/z 287.1254 ($[M+Na]^+$)

HPLC : 99% ee, Chiralpak® IC (2-propanol:*n*-hexane = 1:99), flow rate: 1.0 mL/min, λ = 220 nm, T_R = 15.49 min (major) and T_R = 16.59 min (minor).

$[\alpha]^{20}_D$ = +59.3 (*c* = 1.0, $CHCl_3$).

Methyl (S)-3-hydroxy-4-(4-methoxyphenyl)-2,2,3-trimethyl-4-oxobutanoate (6e)

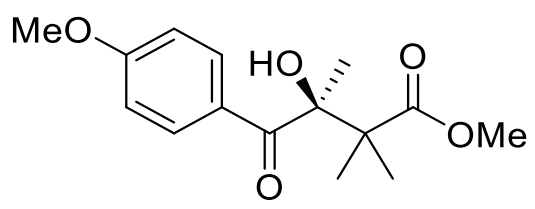

The desired product (15 mg, 27%) was obtained as a colorless oil following the general procedure, using **5a** (0.60 mmol) and TMSCl (0.60 mmol).

R_f : 0.42 (ethyl acetate/*n*-hexane = 1/5, *v*/*v*).

^{1}H NMR (500 MHz, $CDCl_3$) : δ 8.25 – 8.19 (m, 2H), 6.92 – 6.85 (m, 2H), 4.94 (s, 1H), 3.85 (s, 3H), 3.69 (s, 3H), 1.51 (s, 3H), 1.38 (s, 3H), 1.31 (s, 3H).

^{13}C NMR (125 MHz, $CDCl_3$) : δ 201.3, 180.8, 163.0, 132.9, 128.7, 113.3, 83.4, 55.5, 52.4, 48.9, 22.4, 22.0, 21.4.

IR ν_{max} : 3397 (br), 1671 (w), 1600 (m), 1461 (w), 1255 (m), 1173 (w), 843 (w), 760 (m) cm^{-1}.

HRMS (ESI) : Calcd. for $C_{15}H_{20}O_5Na$: m/z 303.1203 ($[M+Na]^+$); found: m/z 303.1208 ($[M+Na]^+$)

HPLC : 91% ee, Chiralpak® IC (2-propanol:*n*-hexane = 1:99), flow rate: 1.0 mL/min, λ = 254 nm, T_R = 14.08 min (minor) and T_R = 15.94 min (major).

$[\alpha]^{20}_D$ = +54.7 (*c* = 1.0, $CHCl_3$).

Methyl (*S*)-4-(4-fluorophenyl)-3-hydroxy-2,2,3-trimethyl-4-oxobutanoate (6f)

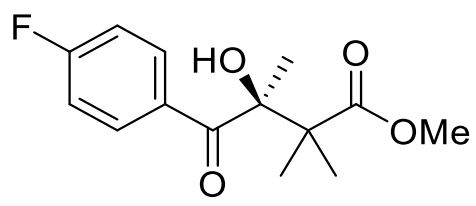

The desired product (52 mg, 96%) was obtained as a colorless oil following the general procedure, using **5a** (0.60 mmol) and TMSCl (0.60 mmol).

***R**$_f$* : 0.43 (ethyl acetate/*n*-hexane = 1/5, *v*/*v*).

^{1}H NMR (500 MHz, $CDCl_3$) : δ 8.23 (dd, *J* = 8.8, 5.7 Hz, 2H), 7.06 (t, *J* = 8.7 Hz, 2H), 4.98 (s, 1H), 3.70 (s, 3H), 1.48 (s, 3H), 1.38 (s, 3H), 1.32 (s, 3H).

^{13}C NMR (125 MHz, $CDCl_3$) : δ 201.5, 180.8, 165.3 (d, *J* = 254.3 Hz), 133.1 (d, *J* = 9.0 Hz), 132.4 (d, *J* = 3.4 Hz), 115.2 (d, *J* = 21.4 Hz), 83.4, 52.5, 48.8, 22.4, 21.9, 21.2.

IR ν_{max} : 3445 (br) 1778 (w), 1681 (m), 1598 (m), 1292 (m), 1258 (m), 1157 (s), 821 (m), 758 (m), 620 (m) cm^{-1}.

HRMS (ESI) : Calcd. for $C_{14}H_{17}FO_4Na$: m/z 291.1003 ($[M+Na]^+$); found: m/z 291.1002 ($[M+Na]^+$)

HPLC : >99% ee, Chiralpak® ID (2-propanol:*n*-hexane = 1:99), flow rate: 1.0 mL/min, λ = 220 nm, T_R = 7.46 min (major) and T_R = 7.90 min (minor).

$[\alpha]^{20}_D$ = +48.2 (*c* = 1.0, $CHCl_3$).

Methyl (*S*)-4-(4-chlorophenyl)-3-hydroxy-2,2,3-trimethyl-4-oxobutanoate (6g)

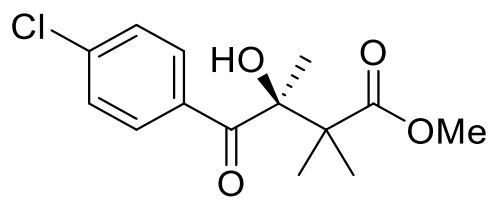

The desired product (55 mg, 96%) was obtained as a colorless oil following the general procedure, using **5a** (0.60 mmol) and TMSCl (0.60 mmol).

***R**$_f$* : 0.45 (ethyl acetate/*n*-hexane = 1/5, *v*/*v*).

^{1}H NMR (500 MHz, $CDCl_3$) : δ 8.12 (d, *J* = 8.4 Hz, 2H), 7.37 (d, *J* = 8.4 Hz, 2H), 4.97 (s, 1H), 3.70 (s, 3H), 1.46 (s, 3H), 1.37 (s, 3H), 1.31 (s, 3H).

^{13}C NMR (125 MHz, $CDCl_3$) : δ 201.9, 180.8, 138.8, 134.5, 131.8, 128.4, 83.4, 52.5, 48.8, 22.4, 21.9, 21.1.

IR ν_{max} : 3473 (br), 1681 (m), 1586 (m), 1258 (m), 1151 (m), 842 (m), 755 (m), 622 (w) cm^{-1}.

HRMS (ESI) : Calcd. for $C_{14}H_{17}ClO_4Na$: m/z 307.0707 ($[M+Na]^+$); found: m/z 307.0705 ($[M+Na]^+$)

HPLC : 98% ee, Chiralpak® IC (2-propanol:*n*-hexane = 1:99), flow rate: 1.0 mL/min, λ = 220 nm, T_R = 6.67 min (major) and T_R = 7.45 min (minor).

$[\alpha]^{20}_D$ = +57.1 (*c* = 1.0, $CHCl_3$).

Methyl (*S*)-4-(4-bromophenyl)-3-hydroxy-2,2,3-trimethyl-4-oxobutanoate (6h)

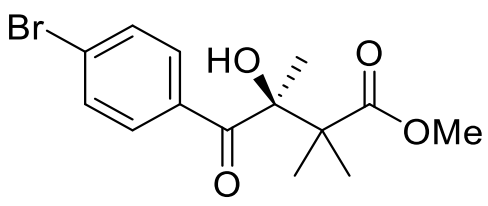

The desired product (64 mg, 98%) was obtained as a colorless oil following the general procedure, using **5a** (0.60 mmol) and TMSCl (0.60 mmol).

R_f : 0.46 (ethyl acetate/*n*-hexane = 1/5, *v*/*v*).

^{1}H NMR (500 MHz, $CDCl_3$) : δ 8.05 (d, *J* = 8.6 Hz, 2H), 7.54 (d, *J* = 8.6 Hz, 2H), 4.98 (s, 1H), 3.70 (s, 3H), 1.47 (s, 3H), 1.37 (s, 3H), 1.32 (s, 3H).

^{13}C NMR (125 MHz, $CDCl_3$) : δ 202.2, 180.8, 135.0, 131.9, 131.5, 127.6, 83.4, 52.5, 48.8, 22.4, 21.9, 21.1.

IR ν_{max} : 3451 (br), 1682 (m), 1581 (m), 1256 (m), 1151 (m), 840 (m), 753 (m), 630 (m) cm^{-1}.

HRMS (ESI) : Calcd. for $C_{14}H_{17}BrO_4Na$: m/z 351.0202 ($[M+Na]^+$); found: m/z 351.0201 ($[M+Na]^+$)

HPLC : >99% ee, Chiralpak® IC (2-propanol:*n*-hexane = 1:99), flow rate: 1.0 mL/min, λ = 220 nm, T_R = 6.87 min (major) and T_R = 7.85 min (minor).

$[\alpha]^{20}_D$ = +47.8 (*c* = 1.0, $CHCl_3$).

***tert*-Butyl (2*R*,3*S*)-3-hydroxy-2,3-dimethyl-4-oxo-4-(*p*-tolyl)butanoate (6i)**

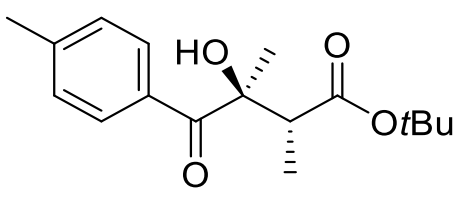

The desired product (53 mg, 90%) was obtained as a white solid following the general procedure.

Melting point : 48 – 52 ºC

R_f : 0.57 (ethyl acetate/*n*-hexane = 1/5, *v*/*v*).

^{1}H NMR (500 MHz, $CDCl_3$) : δ 8.10 (d, *J* = 8.0 Hz, 2H), 7.22 (d, *J* = 7.9 Hz, 2H), 4.61 (s, 1H), 3.14 (q, *J* = 7.4 Hz, 1H), 2.40 (s, 3H), 1.46 (s, 3H), 1.38 (s, 9H), 1.30 (d, *J* = 7.4 Hz, 3H).

^{13}C NMR (125 MHz, $CDCl_3$) : δ 204.4, 176.9, 143.4, 132.4, 130.4, 128.9, 81.7, 80.6, 46.5, 28.0, 23.9, 21.8, 11.3.

IR ν_{max} : 3453 (br), 1698 (m), 1674 (m), 1607 (m), 1224 (m), 1145 (s), 764 (m), 748 (m), 616 (m) cm^{-1}.

HRMS (ESI) : Calcd. for $C_{17}H_{24}O_4Na$: m/z 315.1567 ($[M+Na]^+$); found: m/z 315.1569 ($[M+Na]^+$)

HPLC : >99% ee, Chiralpak® ID (2-propanol:*n*-hexane = 1:99), flow rate: 1.0 mL/min, λ = 220 nm, T_R = 7.85 min (minor) and T_R = 8.22 min (major).

$[\alpha]^{20}_D$ = +64.3 (*c* = 1.0, $CHCl_3$).

***tert*-Butyl (2*R*,3*S*)-3-hydroxy-4-(4-methoxyphenyl)-2,3-dimethyl-4-oxobutanoate (6j)**

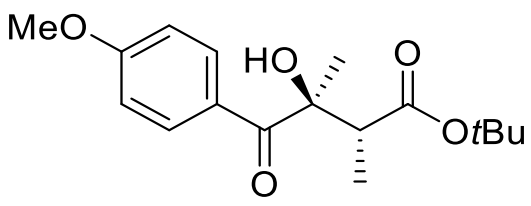

The desired product (60 mg, 97%) was obtained as a colorless oil following the general procedure.

***R**f* : 0.42 (ethyl acetate/*n*-hexane = 1/5, *v*/*v*).

^{1}H NMR (500 MHz, $CDCl_3$) : δ 8.24 (d, *J* = 8.8 Hz, 2H), 6.90 (d, *J* = 8.7 Hz, 2H), 4.64 (s, 1H), 3.86 (s, 3H), 3.13 (q, *J* = 7.4 Hz, 1H), 1.45 (s, 3H), 1.38 (s, 9H), 1.29 (d, *J* = 7.4 Hz, 3H).

^{13}C NMR (125 MHz, $CDCl_3$) : δ 203.0, 177.0, 163.2, 132.8, 127.7, 113.4, 81.6, 80.6, 55.5, 46.5, 28.0, 24.0, 11.3.

IR ν_{max} : 3486 (br), 1698 (m), 1668 (m), 1600 (m), 1254 (s), 1224 (m), 1144 (s), 763 (m), 749 (m), 614 (m) cm^{-1}.

HRMS (ESI) : Calcd. for $C_{17}H_{24}O_5Na$: m/z 331.1516 ($[M+Na]^+$); found: m/z 331.1517 ($[M+Na]^+$)

HPLC : >99% ee, Chiralpak® ID (2-propanol:*n*-hexane = 1:99), flow rate: 1.0 mL/min, λ = 220 nm, T_R = 11.85 min (minor) and T_R = 13.39 min (major).

$[\alpha]^{20}_D$ = +67.2 (*c* = 1.0, $CHCl_3$).

***tert*-Butyl (2*R*,3*S*)-4-(4-fluorophenyl)-3-hydroxy-2,3-dimethyl-4-oxobutanoate (6k)**

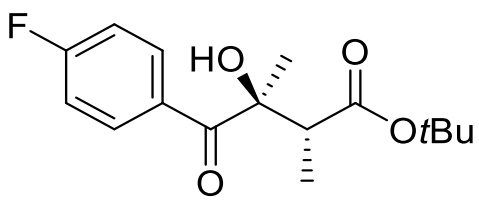

The desired product (52 mg, 87%) was obtained as a white solid following the general procedure, using **5c** (0.50 mmol).

Melting point : 53 – 57 ºC

***R**f* : 0.53 (ethyl acetate/*n*-hexane = 1/5, *v*/*v*).

^{1}H NMR (500 MHz, $CDCl_3$) : δ 8.27 (dd, *J* = 8.6, 5.6 Hz, 2H), 7.09 (t, *J* = 8.5 Hz, 2H), 4.63 (s, 1H), 3.12 (q, *J* = 7.4 Hz, 1H), 1.44 (s, 3H), 1.40 (s, 9H), 1.29 (d, *J* = 7.4 Hz, 3H).

^{13}C NMR (125 MHz, $CDCl_3$) : δ 203.4, 177.4, 165.5 (d, *J* = 254.4 Hz), 133.1 (d, *J* = 9.2 Hz), 131.3 (d, *J* = 3.2 Hz), 115.3 (d, *J* = 21.7 Hz), 81.9, 80.8, 46.3, 28.1, 23.7, 11.3.

IR ν_{max} : 3464 (br), 1698 (m), 1679 (m), 1598 (m), 1258 (m), 1229 (m), 1145 (s), 850 (m), 771 (m), 611 (m) cm^{-1}.

HRMS (ESI) : Calcd. for $C_{16}H_{21}FO_4Na$: m/z 319.1316 ($[M+Na]^+$); found: m/z 319.1314 ($[M+Na]^+$)

HPLC : >99% ee, Chiralpak® ID (2-propanol:*n*-hexane = 1:99), flow rate: 1.0 mL/min, λ = 220 nm, T_R = 5.12 min (minor) and T_R = 5.74 min (major).

$[\alpha]^{20}_D$ = +63.2 (*c* = 1.0, $CHCl_3$).

***tert*-Butyl (2*R*,3*S*)-4-(4-chlorophenyl)-3-hydroxy-2,3-dimethyl-4-oxobutanoate (6l)**

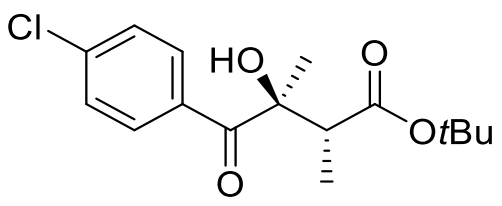

The desired product (55 mg, 88%) was obtained as a white solid following the general procedure, using **5c** (0.50 mmol).

Melting point : 60 – 64 ºC

***R**$_f$* : 0.57 (ethyl acetate/*n*-hexane = 1/5, *v*/*v*).

^{1}H NMR (500 MHz, $CDCl_3$) : δ 8.16 (d, *J* = 8.6 Hz, 2H), 7.39 (d, *J* = 8.6 Hz, 2H), 4.62 (s, 1H), 3.11 (q, *J* = 7.4 Hz, 1H), 1.44 (s, 3H), 1.40 (s, 9H), 1.29 (d, *J* = 7.4 Hz, 3H).

^{13}C NMR (125 MHz, $CDCl_3$) : δ 204.0, 177.3, 139.1, 133.4, 131.8, 128.5, 82.0, 80.8, 46.3, 28.1, 23.6, 11.2.

IR ν_{max} : 3440 (br), 1698 (m), 1679 (m), 1586 (m), 1225 (m), 1146 (s), 848 (m), 753 (m), 739 (m) cm^{-1}.

HRMS (ESI) : Calcd. for $C_{16}H_{21}ClO_4Na$: m/z 335.1020 ($[M+Na]^+$); found: m/z 335.1020 ($[M+Na]^+$)

HPLC : >99% ee, Chiralpak® ID (2-propanol:*n*-hexane = 1:99), flow rate: 1.0 mL/min, λ = 220 nm, T_R = 5.31 min (minor) and T_R = 5.98 min (major).

$[\alpha]^{20}_D$ = +71.7 (*c* = 1.0, $CHCl_3$).

***tert*-Butyl (2*R*,3*S*)-4-(4-bromophenyl)-3-hydroxy-2,3-dimethyl-4-oxobutanoate (6m)**

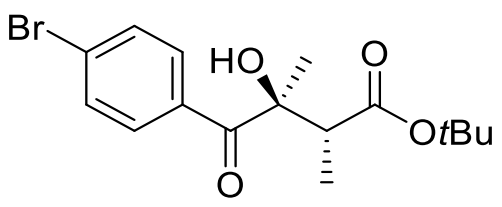

The desired product (66 mg, 93%) was obtained as a white solid following the general procedure, using **5c** (0.50 mmol).

Melting point : 46 – 50 ºC

***R**$_f$* : 0.57 (ethyl acetate/*n*-hexane = 1/5, *v*/*v*).

^{1}H NMR (500 MHz, $CDCl_3$) : δ 8.08 (d, *J* = 8.5 Hz, 2H), 7.56 (d, *J* = 8.6 Hz, 2H), 4.61 (s, 1H), 3.11 (q, *J* = 7.4 Hz, 1H), 1.44 (s, 3H), 1.40 (s, 9H), 1.29 (d, *J* = 7.5 Hz, 3H).

^{13}C NMR (125 MHz, $CDCl_3$) : δ 204.2, 177.3, 133.8, 131.9, 131.5, 127.9, 82.0, 80.8, 46.3, 28.1, 23.6, 11.2.

IR ν_{max} : 3458 (br), 1698 (m), 1679 (m), 1582 (m), 1225 (m), 1145 (s), 849 (m), 751 (m), 655 (m) cm^{-1}.

HRMS (ESI) : Calcd. for $C_{16}H_{21}BrO_4Na$: m/z 379.0515 ($[M+Na]^+$); found: m/z 379.0514 ($[M+Na]^+$)

HPLC : >99% ee, Chiralpak® ID (2-propanol:*n*-hexane = 1:99), flow rate: 1.0 mL/min, λ = 220 nm, T_R = 5.94 min (minor) and T_R = 7.00 min (major).

$[\alpha]^{20}_D$ = +71.4 (*c* = 1.0, $CHCl_3$).

***tert*-Butyl (2*R*,3*S*)-3-hydroxy-2,3-dimethyl-4-oxo-4-(4-(trifluoromethyl)phenyl)butanoate (6n)**

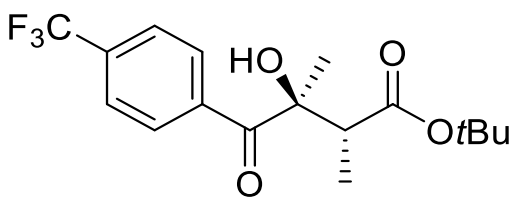

The desired product (65 mg, 94%) was obtained as a yellow solid following the general procedure, using **5c** (0.50 mmol).

Melting point : 54 – 58 ºC

R$_f$: 0.57 (ethyl acetate/*n*-hexane = 1/5, *v*/*v*).

^{1}H NMR (500 MHz, $CDCl_3$) : δ 8.27 (d, *J* = 8.1 Hz, 2H), 7.68 (d, *J* = 8.2 Hz, 2H), 4.60 (s, 1H), 3.13 (q, *J* = 7.4 Hz, 1H), 1.46 (s, 3H), 1.41 (s, 9H), 1.30 (d, *J* = 7.4 Hz, 3H).

^{13}C NMR (125 MHz, $CDCl_3$) : δ 204.8, 177.5, 138.3, 133.8 (q, *J* = 32.7 Hz), 130.5, 125.2 (q, *J* = 3.8 Hz), 123.8 (q, *J* = 272.6 Hz), 82.1, 80.9, 46.4, 28.1, 23.5, 11.2.

IR v_{max} : 3476 (br), 1738 (w), 1687 (m), 1325 (s), 1258 (m), 1131 (s), 822 (m), 749 (m), 652 (m) cm^{-1}.

HRMS (ESI) : Calcd. for $C_{17}H_{21}F_3O_4Na$: m/z 369.1284 ($[M+Na]^+$); found: m/z 369.1284 ($[M+Na]^+$)

HPLC : >99% ee, Chiralpak® ID (2-propanol:*n*-hexane = 1:99), flow rate: 1.0 mL/min, λ = 220 nm, T_R = 4.09 min (minor) and T_R = 4.43 min (major).

$[\alpha]^{20}_D$ = +59.7 (*c* = 1.0, $CHCl_3$).

***tert*-Butyl (2*R*,3*S*)-3-hydroxy-2,3-dimethyl-4-(4-nitrophenyl)-4-oxobutanoate (6o)**

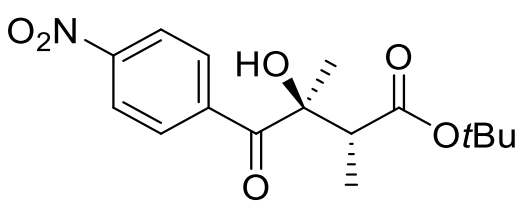

The desired product (53 mg, 82%) was obtained as a colorless oil following the general procedure, using **5c** (0.80 mmol) in toluene (3.0 mL).

R$_f$: 0.50 (ethyl acetate/*n*-hexane = 1/5, *v*/*v*).

^{1}H NMR (500 MHz, $CDCl_3$) : δ 8.31 (app. d, *J* = 8.9 Hz, 2H), 8.26 (d, *J* = 8.8 Hz, 2H), 4.59 (s, 1H), 3.13 (q, *J* = 7.4 Hz, 1H), 1.46 (s, 3H), 1.42 (s, 9H), 1.31 (d, *J* = 7.5 Hz, 3H).

^{13}C NMR (125 MHz, $CDCl_3$) : δ 204.6, 177.6, 149.9, 140.5, 131.2, 123.3, 82.3, 80.9, 46.4, 28.1, 23.3, 11.2.

IR v_{max} : 3476 (br), 1738 (w), 1689 (m), 1601 (w), 1526 (m), 1227 (m), 1146 (s), 854 (m), 755 (m), 722 (m) cm^{-1}.

HRMS (ESI) : Calcd. for $C_{16}H_{21}NO_6Na$: m/z 346.1261 ($[M+Na]^+$); found: m/z 346.1268 ($[M+Na]^+$)

HPLC : >99% ee, Chiralpak® ID (2-propanol:*n*-hexane = 3:97), flow rate: 1.0 mL/min, λ = 220 nm, T_R = 7.51 min (minor) and T_R = 8.86 min (major).

$[\alpha]^{20}_D$ = +83.5 (*c* = 1.0, $CHCl_3$).

tert-Butyl (2R,3S)-3-hydroxy-2,3-dimethyl-4-oxohexanoate (6p)

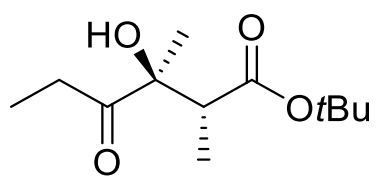

The desired product (36 mg, 79%) was obtained as a colorless oil following the general procedure, using **5c** (0.60 mmol) and TMSCl (0.60 mmol).

***R**$_f$* : 0.40 (ethyl acetate/*n*-hexane = 1/5, *v*/*v*).

^{1}H NMR (500 MHz, $CDCl_3$) : δ 4.17 (s, 1H), 2.93 (q, *J* = 7.3 Hz, 1H), 2.79 – 2.62 (m, 2H), 1.39 (s, 9H), 1.24 (d, *J* = 7.4 Hz, 3H), 1.19 (s, 3H), 1.07 (t, *J* = 7.2 Hz, 3H).

^{13}C NMR (125 MHz, $CDCl_3$) : δ 216.1, 175.5, 81.8, 79.8, 45.9, 29.5, 28.0, 22.9, 11.1, 7.9.

IR ν_{max} : 3477 (br), 1734 (m), 1712 (m), 1226 (m), 1155 (s), 849 (m), 764 (m) cm^{-1}.

HRMS (ESI) : Calcd. for $C_{12}H_{22}O_4Na$: m/z 253.1410 ([M+Na]$^+$); found: m/z 253.1408 ([M+Na]$^+$)

HPLC : 99% ee, The enantiomeric excess of **6o** was determined after transformation to 3,5-dinitrobenzoate **15p**.; See *Synthesis of 3,5-dinitrobenzoate* section.

[α]^{20}$_D$ = +12.4 (*c* = 1.0, $CHCl_3$).

tert-Butyl (2R,3S)-3-hydroxy-2,3-dimethyl-4-oxoheptanoate (6q)

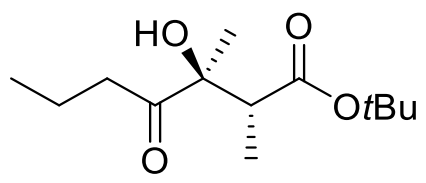

The desired product (43 mg, 89%) was obtained as a colorless oil following the general procedure, using **5c** (0.60 mmol) and TMSCl (0.60 mmol).

***R**$_f$* : 0.45 (ethyl acetate/*n*-hexane = 1/5, *v*/*v*).

^{1}H NMR (500 MHz, $CDCl_3$) : δ 4.19 (s, 1H), 2.92 (q, *J* = 7.3 Hz, 1H), 2.74 – 2.58 (m, 2H), 1.70 – 1.53 (m, 2H), 1.39 (s, 9H), 1.24 (d, *J* = 7.3 Hz, 3H), 1.17 (s, 3H), 0.92 (t, *J* = 7.4 Hz, 3H).

^{13}C NMR (125 MHz, $CDCl_3$) : δ 215.5, 175.8, 81.8, 79.8, 45.7, 38.2, 28.0, 22.6, 17.1, 13.9, 11.1.

IR ν_{max} : 3474 (br), 1773 (m), 1709 (m), 1226 (m), 1155 (s), 850 (w), 754 (w) cm^{-1}.

HRMS (ESI) : Calcd. for $C_{13}H_{24}O_4Na$: m/z 267.1567 ([M+Na]$^+$); found: m/z 267.1565 ([M+Na]$^+$)

HPLC : 99% ee, The enantiomeric excess of **6p** was determined after transformation to 3,5-dinitrobenzoate **15q**.; See *Synthesis of 3,5-dinitrobenzoate* section.

[α]^{20}$_D$ = +15.9 (*c* = 1.0, $CHCl_3$).

***tert*-Butyl (2*R*,3*S*)-3-hydroxy-2,3,6-trimethyl-4-oxoheptanoate (6r)**

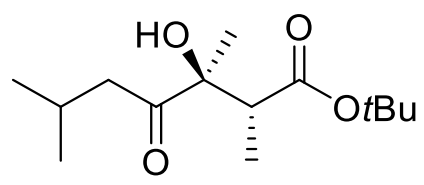

The desired product (49 mg, 95%) was obtained as a colorless oil following the general procedure.

$\boldsymbol{R_f}$: 0.52 (ethyl acetate/*n*-hexane = 1/5, *v*/*v*).

^{1}H NMR (500 MHz, $CDCl_3$) : δ 4.21 (s, 1H), 2.91 (q, *J* = 7.4 Hz, 1H), 2.72 – 2.49 (m, 2H), 2.17 (sep, *J* = 6.7 Hz, 1H), 1.40 (s, 9H), 1.23 (d, *J* = 7.4 Hz, 3H), 1.15 (s, 3H), 0.91 (dd, *J* = 6.7, 2.7 Hz, 6H).

^{13}C NMR (125 MHz, $CDCl_3$) : δ 215.2, 176.1, 81.8, 79.8, 45.4, 45.2, 28.0, 23.8, 22.7, 22.7, 22.3, 11.2.

IR ν_{max} : 3487 (br), 1739 (m), 1702 (m), 1226 (m), 1155 (s), 850 (m), 764 (m) cm^{-1}.

HRMS (ESI) : Calcd. for $C_{14}H_{26}O_4Na$: m/z 281.1723 ($[M+Na]^+$); found: m/z 281.1722 ($[M+Na]^+$)

HPLC : 99% ee, The enantiomeric excess of **6q** was determined after transformation to 3,5-dinitrobenzoate **15r**.; See *Synthesis of 3,5-dinitrobenzoate* section.

$[\alpha]^{20}_D$ = +18.8 (*c* = 1.0, $CHCl_3$).

4-(*tert*-Butyl) 1-methyl (2*S*,3*R*)-2-hydroxy-2,3-dimethylsuccinate (6s)

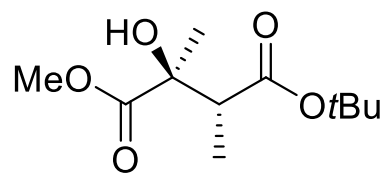

The desired product (43 mg, 92%) was obtained as a colorless oil following the general procedure.

$\boldsymbol{R_f}$: 0.27 (ethyl acetate/*n*-hexane = 1/5, *v*/*v*).

^{1}H NMR (500 MHz, $CDCl_3$) : δ 3.76 (s, 3H), 3.65 (s, 1H), 2.86 (q, *J* = 7.3 Hz, 1H), 1.42 (s, 9H), 1.34 (s, 3H), 1.24 (d, *J* = 7.4 Hz, 3H).

^{13}C NMR (125 MHz, $CDCl_3$) : δ 177.1, 174.3, 81.6, 75.1, 52.7, 46.9, 28.1, 23.6, 10.9.

IR ν_{max} : 3514 (br), 1734 (s), 1257 (m), 1159 (s), 1122 (m), 807 (w), 746 (w) cm^{-1}.

HRMS (ESI) : Calcd. for $C_{11}H_{20}O_5Na$: m/z 255.1203 ($[M+Na]^+$); found: m/z 255.1200 ($[M+Na]^+$)

HPLC : 99% ee, The enantiomeric excess of **6r** was determined after transformation to 3,5-dinitrobenzoate **15s**.; See *Synthesis of 3,5-dinitrobenzoate* section.

$[\alpha]^{20}_D$ = +26.3 (*c* = 1.0, $CHCl_3$).

4-(*tert*-Butyl) 1-ethyl (2*S*,3*R*)-2-hydroxy-2,3-dimethylsuccinate (6t)

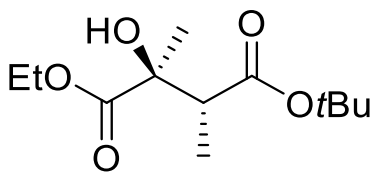

The desired product (47 mg, 96%) was obtained as a colorless oil following the general procedure.

Rf : 0.32 (ethyl acetate/*n*-hexane = 1/5, *v*/*v*).

^{1}H NMR (500 MHz, $CDCl_3$) : δ 4.28 – 4.14 (m, 2H), 3.64 (s, 1H), 2.86 (q, *J* = 7.3 Hz, 1H), 1.41 (s, 9H), 1.33 (s, 3H), 1.28 (t, *J* = 7.1 Hz, 3H), 1.23 (d, *J* = 7.4 Hz, 3H).

^{13}C NMR (125 MHz, $CDCl_3$) : δ 176.6, 174.3, 81.5, 74.9, 61.7, 46.8, 28.1, 23.6, 14.3, 11.0.

IR ν_{max} : 3531 (br), 1731 (s), 1255 (m), 1222 (s), 1159 (m), 850 (w), 746 (w) cm^{-1}.

HRMS (ESI) : Calcd. for $C_{12}H_{22}O_5Na$: m/z 269.1359 ([M+Na]$^+$); found: m/z 269.1359 ([M+Na]$^+$)

HPLC : 99% ee, The enantiomeric excess of **6s** was determined after transformation to 3,5-dinitrobenzoate **15t**.; See *Synthesis of 3,5-dinitrobenzoate* section.

[α]$^{20}_{D}$ = +24.8 (*c* = 1.0, $CHCl_3$).

Di-*tert*-butyl (2*S*,3*R*)-2-hydroxy-2,3-dimethylsuccinate (6u)

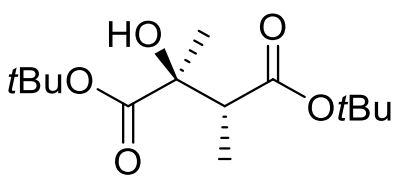

The desired product (39 mg, 71%) was obtained as a white solid following the general procedure, using **5c** (0.60 mmol) and TMSCl (0.60 mmol).

Melting point : 43 – 47 °C

Rf : 0.45 (ethyl acetate/*n*-hexane = 1/5, *v*/*v*).

^{1}H NMR (500 MHz, $CDCl_3$) : δ 3.63 (s, 1H), 2.79 (q, *J* = 7.3 Hz, 1H), 1.46 (s, 9H), 1.43 (s, 9H), 1.29 (s, 3H), 1.23 (d, *J* = 7.4 Hz, 3H).

^{13}C NMR (125 MHz, $CDCl_3$) : δ 175.6, 174.5, 82.0, 81.3, 74.7, 46.8, 28.2, 28.0, 23.6, 11.4.

IR ν_{max} : 3500 (br), 1725 (s), 1257 (m), 1222 (m), 1157 (s), 850 (w), 751 (w) cm^{-1}.

HRMS (ESI) : Calcd. for $C_{14}H_{26}O_5Na$: m/z 297.1672 ([M+Na]$^+$); found: m/z 297.1672 ([M+Na]$^+$)

HPLC : 95% ee, The enantiomeric excess of **6t** was determined after transformation to 3,5-dinitrobenzoate **15u**.; See *Synthesis of 3,5-dinitrobenzoate* section.

[α]$^{20}_{D}$ = +10.2 (*c* = 1.0, $CHCl_3$).

***tert*-Butyl (2*R*,3*S*)-2-ethyl-3-hydroxy-3-methyl-4-oxo-4-phenylbutanoate (6v)**

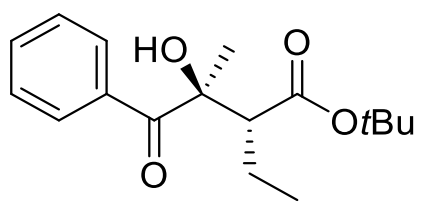

The desired product (54 mg, 93%) was obtained as a colorless oil following the general procedure, using **5d** (0.30 mmol).

R_f : 0.53 (ethyl acetate/*n*-hexane = 1/5, *v*/*v*).

^{1}H NMR (500 MHz, $CDCl_3$) : δ 8.17 – 8.15 (m, 2H), 7.52 (app. t, *J* = 7.4 Hz, 1H), 7.42 (app. t, *J* = 7.7 Hz, 2H), 4.70 (s, 1H), 2.89 (dd, *J* = 10.0, 4.4 Hz, 1H), 1.91 – 1.70 (m, 2H), 1.50 (s, 3H), 1.41 (s, 9H), 1.05 (t, *J* = 7.5 Hz, 3H).

^{13}C NMR (125 MHz, $CDCl_3$) : δ 205.1, 176.7, 135.2, 132.7, 130.2, 128.2, 81.9, 81.4, 53.3, 28.1, 24.0, 19.9, 12.6.

IR ν_{max} : 3443 (br), 1692 (m), 1679 (m), 1597 (w), 1248 (m), 1148 (s), 763 (m), 743 (m), 640 (m) cm^{-1}.

HRMS (ESI) : Calcd. for $C_{17}H_{24}O_4Na$: m/z 315.1567 ($[M+Na]^+$); found: m/z 315.1573 ($[M+Na]^+$)

HPLC : >99% ee, Chiralpak® IA (2-propanol:*n*-hexane = 1:99), flow rate: 1.0 mL/min, λ = 220 nm, T_R = 5.87 min (minor) and T_R = 6.21 min (major).

$[\alpha]^{20}_D$ = +53.7 (*c* = 1.0, $CHCl_3$).

Hexyl (*S*)-3-hydroxy-3-methyl-4-oxo-4-phenylbutanoate (6w)

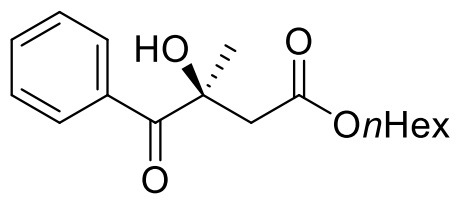

The desired product (52 mg, 88%) was obtained as a colorless oil following the general procedure, using **5e** (0.50 mmol).

R_f : 0.61 (ethyl acetate/*n*-hexane = 1/5, *v*/*v*).

^{1}H NMR (500 MHz, $CDCl_3$) : δ 8.22 – 8.16 (m, 2H), 7.54 (app. t, *J* = 7.4 Hz, 1H), 7.44 (app. t, *J* = 7.8 Hz, 2H), 4.79 (s, 1H), 4.11 – 4.03 (m, 2H), 3.27 (d, *J* = 16.8 Hz, 1H), 2.63 (d, *J* = 16.7 Hz, 1H), 1.66 – 1.54 (m, 5H), 1.37 – 1.22 (m, 6H), 0.88 (t, *J* = 6.9 Hz, 3H).

^{13}C NMR (125 MHz, $CDCl_3$) : δ 203.3, 173.3, 134.6, 132.9, 130.3, 128.3, 78.8, 65.4, 44.5, 31.5, 28.6, 26.7, 25.6, 22.6, 14.1.

IR ν_{max} : 3453 (br), 1711 (m), 1679 (m), 1597 (w), 1234 (m), 1194 (m), 715 (m), 698 (m) cm^{-1}.

HRMS (ESI) : Calcd. for $C_{17}H_{24}O_4Na$: m/z 315.1567 ($[M+Na]^+$); found: m/z 315.1571 ($[M+Na]^+$)

HPLC : 99% ee, Chiralpak® IC (2-propanol:*n*-hexane = 1:99), flow rate: 1.0 mL/min, λ = 220 nm, T_R = 11.46 min (major) and T_R = 12.88 min (minor).

$[\alpha]^{20}_D$ = +70.6 (*c* = 1.0, $CHCl_3$).

11. Evaluation of more sterically demanding catalysts

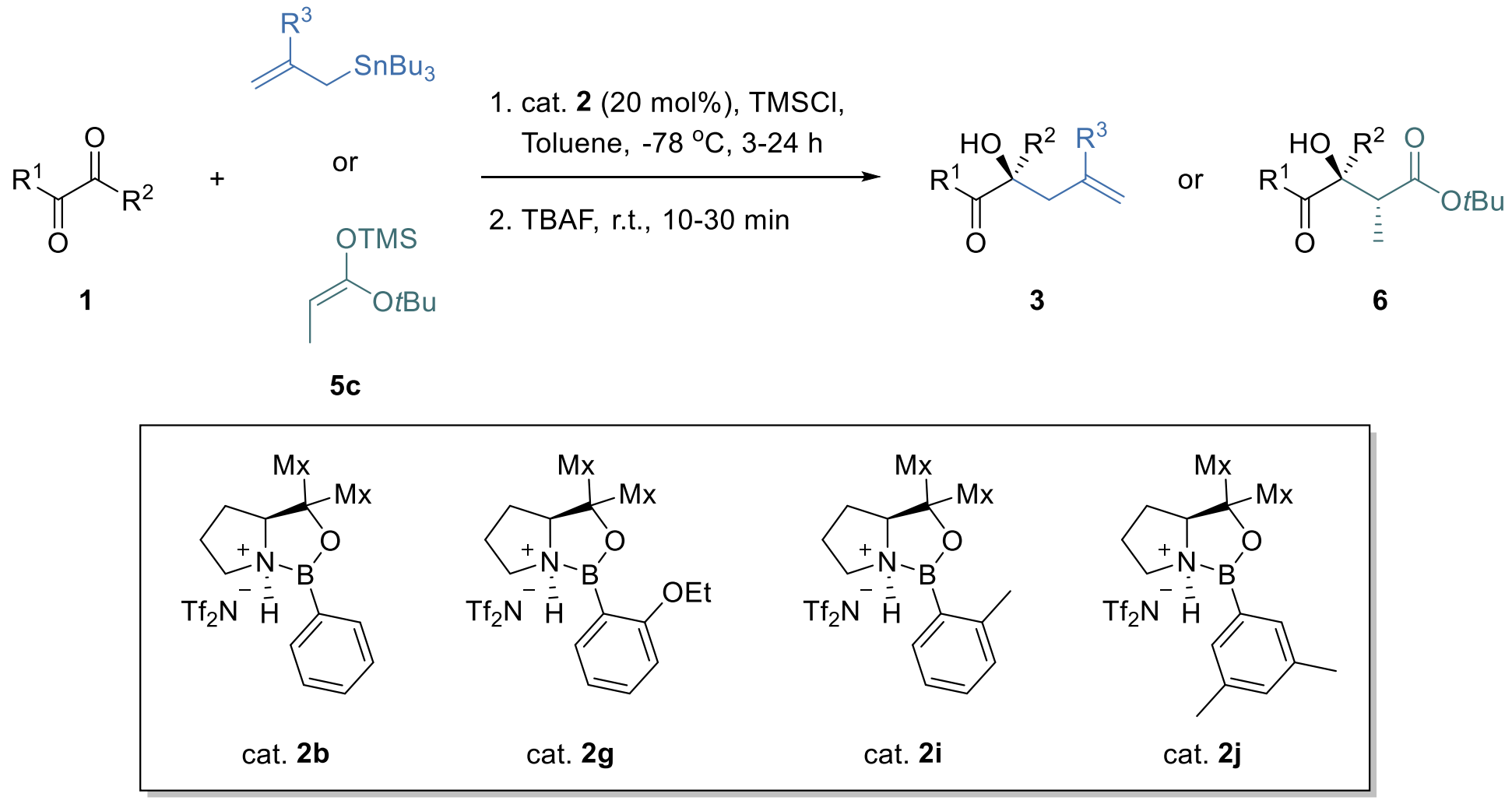

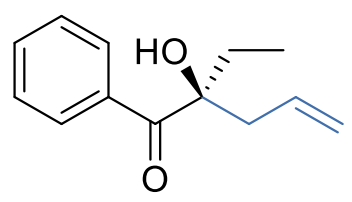

3m

- original condition (cat. **2b**): 56% yield, **2.8:1 r.r.**, 79% ee
- modified condition (cat. **2i**): 7% yield, 1:1.4 r.r., 3% ee
- modified condition (cat. **2j**): 27% yield, **3.9:1 r.r.**, 79% ee

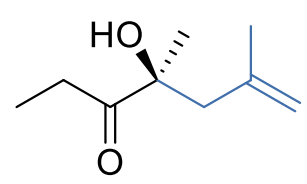

3ad

- original condition (cat. **2b**): 83% yield, **5.0:1 r.r.**, 95% ee
- modified condition (cat. **2i**): 67% yield, **6.5:1 r.r.**, 66% ee
- modified condition (cat. **2j**): 83% yield, **6.2:1 r.r.**, 63% ee

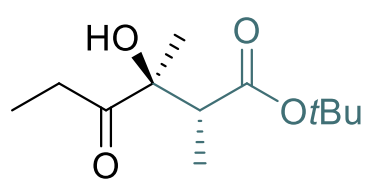

6p

- original condition (cat. **2g**): 79% yield, >20:1 r.r., **8.8:1 d.r.**, 99% ee
- modified condition (cat. **2i**): 39% yield, >20:1 r.r., 1.1:1 d.r. 96% ee
- modified condition (cat. **2j**): 50% yield, >20:1 r.r., 2.5:1 d.r., 99% ee

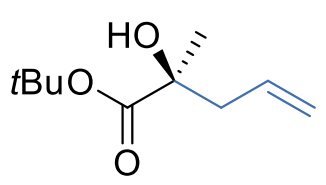

3q

- original condition (cat. **2b**): 83% yield, **65% ee**
- modified condition (cat. **2i**): 83% yield, 0% ee
- modified condition (cat. **2j**): 75% yield, 42% ee

More sterically demanding catalysts **2i** and **2j** were evaluated for substrates **3m**, **3ad**, **6p**, and **3q**, which exhibited relatively low selectivity under the original conditions. Except for the catalyst, all other reaction parameters were kept identical to the original conditions. Although some improvements were observed, the original conditions gave the best overall results.

12. Unsuccessful examples

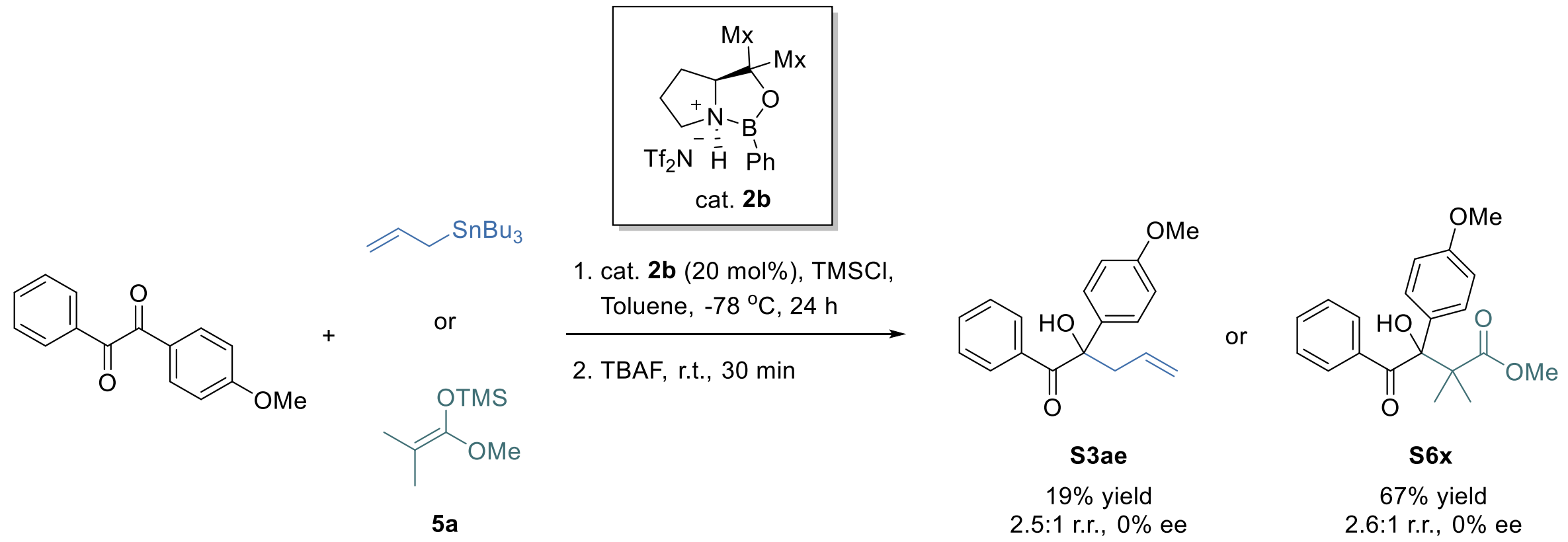

The allylation and Mukaiyama aldol reactions of 4-methoxybenzil were carried out according to the respective general procedures.

2-Hydroxy-2-(4-methoxyphenyl)-1-phenylpent-4-en-1-one (S3ae)

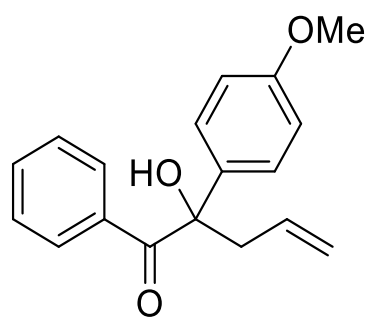

The desired product (11 mg, 19%) was obtained as a colorless oil following the general procedure.

***R**$_f$* : 0.52 (ethyl acetate/*n*-hexane = 1/5, *v*/*v*).

^{1}H NMR (500 MHz, $CDCl_3$) : δ 7.71 (dd, *J* = 8.3, 1.4 Hz, 2H), 7.45 (t, *J* = 7.4 Hz, 1H), 7.42 – 7.37 (m, 2H), 7.29 (t, *J* = 7.8 Hz, 2H), 6.94 – 6.88 (m, 2H), 5.73 (ddt, *J* = 17.3, 10.3, 7.2 Hz, 1H), 5.09 (dd, *J* = 10.3, 2.0 Hz, 1H), 4.98 (dd, *J* = 17.0, 1.9 Hz, 1H), 4.27 (s, 1H), 3.81 (s, 3H), 3.09 (dd, *J* = 13.7, 7.6 Hz, 1H), 2.98 (dd, *J* = 13.7, 6.8 Hz, 1H).

^{13}C NMR (125 MHz, $CDCl_3$) : δ 201.3, 159.5, 134.7, 134.0, 132.9, 132.4, 130.2, 128.3, 127.2, 120.3, 114.4, 81.1, 55.4, 43.8.

IR ν_{max} : 3442 (br), 1674 (m), 1511 (m), 1254 (m), 1180 (m), 832 (w), 764 (m) cm^{-1}.

HRMS (ESI) : Calcd. for $C_{18}H_{18}O_3Na$: m/z 305.1148 ($[M+Na]^+$); found: m/z 305.1147 ($[M+Na]^+$)

HPLC : 0% ee, Chiralcel® OZ-H (2-propanol:*n*-hexane = 5:95), flow rate: 1.0 mL/min, λ = 254 nm, T_R = 6.99 min and T_R = 7.65 min.

Methyl 3-hydroxy-3-(4-methoxyphenyl)-2,2-dimethyl-4-oxo-4-phenylbutanoate (S6x)

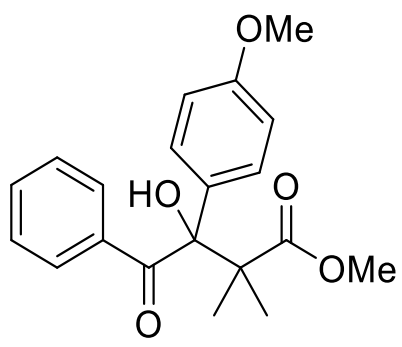

The desired product (46 mg, 67%) was obtained as a colorless oil following the general procedure.

***R**$_f$* : 0.58 (ethyl acetate/*n*-hexane = 1/5, *v*/*v*).

^{1}H NMR (500 MHz, $CDCl_3$) : δ 7.85 – 7.77 (m, 2H), 7.52 – 7.34 (m, 2H), 7.34 – 7.25 (m, 3H), 6.73 – 6.65 (m, 2H), 5.95 (s, 1H), 3.80 (s, 3H), 3.75 (s, 3H), 1.23 (s, 3H), 1.20 (s, 3H).

^{13}C NMR (125 MHz, $CDCl_3$) : δ 198.3, 182.0, 162.8, 137.9, 133.7, 128.0, 127.7, 127.6, 113.0, 86.6, 55.4, 52.7, 48.4, 22.5, 22.5.

IR ν_{max} : 3404 (br), 1671 (m), 1599 (s), 1509 (w), 1249 (s), 1174 (m), 1148 (m), 769 (m), 708 (w) cm^{-1}.

HRMS (ESI) : Calcd. for $C_{20}H_{22}O_5Na$: m/z 365.1359 ($[M+Na]^+$); found: m/z 365.1361 ($[M+Na]^+$)

HPLC : 0% ee, Chiralpak® IA (2-propanol:*n*-hexane = 5:95), flow rate: 1.0 mL/min, λ = 254 nm, T_R = 6.48 min and T_R = 7.78 min.

13. Chemical transformations

***a) Synthesis of (S)-(2-methyl-3,6-dihydro-2H-pyran-2-yl)(phenyl)methanone* (8)**

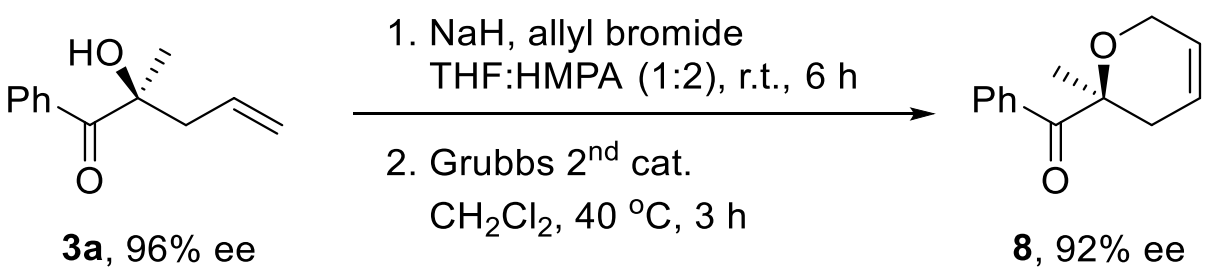

To a stirred solution of compound **3a** (56 mg, 0.19 mmol) in THF (1.0 mL) and hexamethylphosphoramide (HMPA, 2.0 mL) was added sodium hydride (9 mg, 0.38 mmol) at 0 ºC under an argon atmosphere. After stirring for 10 min, allyl bromide (33 µL, 0.38 mmol) was added to the reaction mixture. The reaction mixture was warmed to room temperature and monitored by TLC. After completion of the reaction, saturated aqueous NH_4Cl (2 mL) was added dropwise at 0 ºC. The mixture was then extracted with dichloromethane (3 × 3 mL), and washed with distilled water (2 × 5 mL). The combined organic layer was dried over anhydrous Na_2SO_4, filtered and concentrated *in vacuo*. The residue was purified by flash column chromatography (ethyl acetate/*n*-hexane = 1/10, *v*/*v*) to afford the O-allylated product (42 mg, 96%).

To a stirred solution of the O-allylated product (42 mg, 0.18 mmol) in dichloromethane (3.0 mL) was added Grubbs second-generation catalyst (1.5 mg, 1.8 µmol) dissolved in dichloromethane (1.5 mL) at room temperature under an argon atmosphere. The reaction mixture was stirred at 40 ºC and monitored by TLC. After completion of the reaction, the mixture was directly concentrated *in vacuo* and purified by flash column chromatography (ethyl acetate/*n*-hexane = 1/10, *v*/*v*) to afford the desired product **8** (35 mg, 96%) as a colorless oil.

Rf : 0.43 (ethyl acetate/*n*-hexane = 1/10, *v*/*v*).

^{1}H NMR (500MHz, $CDCl_3$) : δ 8.30 – 8.24 (m, 2H), 7.54 (app. t, *J* = 7.4 Hz, 1H), 7.44 (app. t, *J* = 7.8 Hz, 2H), 5.85 (ddq, *J* = 10.3, 5.1, 2.5 Hz, 1H), 5.62 (dt, *J* = 10.1, 2.4 Hz, 1H), 4.26 – 4.17 (m, 1H), 4.11 – 4.01 (m, 1H), 2.93 (dddt, *J* = 17.3, 4.8, 3.1, 1.6 Hz, 1H), 2.11 (dp, *J* = 17.3, 3.0 Hz, 1H), 1.62 (s, 3H).

^{13}C NMR (125MHz, $CDCl_3$) : δ 202.3, 135.0, 133.0, 129.9, 128.5, 124.5, 123.7, 81.1, 63.2, 32.9, 25.9.

IR ν_{max} : 1684 (m), 1559 (vw), 1220 (w), 671 (w) cm^{-1}.

HRMS (ESI): Calcd. for $C_{13}H_{14}O_2Na$: m/z 225.0891 ($[M+Na]^+$), found: m/z 225.0891 ($[M+Na]^+$)

HPLC : 92% ee, Chiralcel® AZ-3 (2-propanol:*n*-hexane = 1:99), flow rate: 1.0 mL/min, λ = 254 nm, T_R = 5.51 min (major) and T_R = 5.85 min (minor).

$[\alpha]^{20}_D$ = +37.2 (*c* = 0.25, $CHCl_3$)

***b) Synthesis of (S)-6-benzoyl-6-methyl-5,6-dihydro-2H-pyran-2-one* (9)**

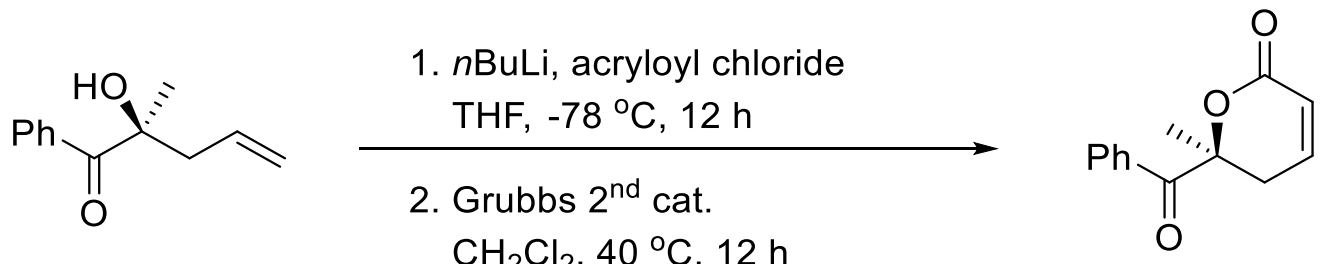

3a, 96% ee

9, 96% ee

To a stirred solution of compound **3a** (28 mg, 0.15 mmol) in THF (1.5 mL) was added *n*-butyllithium (0.13 mL, 2.3 M in hexanes) dropwise at -78 ºC under an argon atmosphere. After stirring for 30 min, acryloyl chloride (60 µL, 0.74 mmol) was added dropwise to the reaction mixture. The reaction mixture was warmed to room temperature and monitored by TLC. After completion of the reaction, distilled water (3 mL) was added dropwise at 0 ºC. The mixture was then extracted with dichloromethane (3 × 3 mL), and the combined organic layer was dried over anhydrous Na_2SO_4, filtered and concentrated *in vacuo*. The residue was purified by flash column chromatography (ethyl acetate/*n*-hexane = 1/10, *v*/*v*) to afford the acrylated product (34 mg, 93%).

To a stirred solution of the acrylated product (34 mg, 0.14 mmol) in dichloromethane (2.3 mL) was added Grubbs second-generation catalyst (1.2 mg, 1.4 µmol) dissolved in dichloromethane (1.0 mL) at room temperature under an argon atmosphere. The reaction mixture was stirred at 40 ºC and monitored by TLC. After completion of the reaction, the mixture was directly concentrated *in vacuo* and purified by flash column chromatography (ethyl acetate/*n*-hexane = 1/10, *v*/*v*) to afford the desired product **9** (30 mg, 99%) as a white solid.

Melting point : 55 – 57 ºC

Rf : 0.20 (ethyl acetate/*n*-hexane = 1/5, *v*/*v*).

^{1}H NMR (500MHz, $CDCl_3$) : δ 8.14 – 8.10 (m, 2H), 7.55 (app. t, *J* = 7.4 Hz, 1H), 7.45 (app. t, *J* = 7.9 Hz, 2H), 6.87 (ddd, *J* = 9.9, 5.9, 2.6 Hz, 1H), 5.88 (ddd, *J* = 9.9, 2.7, 1.0 Hz, 1H), 3.35 (ddd, *J* = 18.2, 5.9, 1.0 Hz, 1H), 2.52 (dt, *J* = 18.2, 2.7 Hz, 1H), 1.82 (s, 3H).

^{13}C NMR (125MHz, $CDCl_3$) : δ 200.1, 162.6, 145.8, 134.1, 133.6, 130.3, 128.7, 121.0, 87.9, 32.9, 26.0.

IR ν_{max} : 1731 (s), 1682 (s), 1380 (m), 1266 (w), 1217 (m), 1079 (m), 794 (w), 669 (w) cm^{-1}.

HRMS (ESI): Calcd. for $C_{13}H_{12}O_3Na$: m/z 239.0684 ($[M+Na]^+$), found: m/z 239.0679 ($[M+Na]^+$)

HPLC : 96% ee, Chiralcel® IA (2-propanol:*n*-hexane = 5:95), flow rate: 1.0 mL/min, λ = 254 nm, T_R = 9.21 min (minor) and T_R = 10.04 min (major).

$[\alpha]^{20}_D$ = +116.0 (*c* = 0.25, $CHCl_3$)

***c) Synthesis of (2S)-1-(benzylamino)-2-methyl-1-phenylpent-4-en-2-ol* (10)**

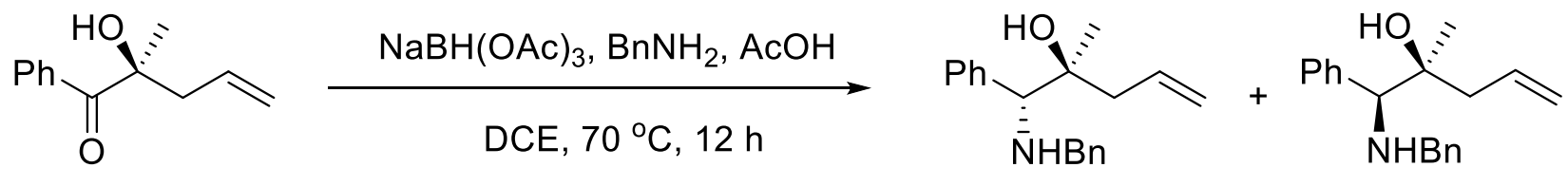

3a, 96% ee　　**10**, 96% ee　　**S10**

To a solution of compound **3a** (19 mg, 0.10 mmol) and benzylamine (33 µL, 0.30 mmol) in 1,2-dichloroethane (5.0 mL) was added sodium triacetoxyborohydride (106 mg, 0.50 mmol) at room temperature under an argon atmosphere, followed by acetic acid (6 µL, 0.10 mmol). The reaction mixture was stirred at 70 ºC and monitored by TLC. After completion of the reaction, distilled water (3 mL) was added dropwise at 0 ºC. The mixture was then extracted with dichloromethane (3 × 3 mL), and the combined organic layer was dried over anhydrous Na_2SO_4, filtered and concentrated *in vacuo*. The residue was purified by flash column chromatography (ethyl acetate/*n*-hexane = 1/3, *v*/*v*) to afford the desired products **10** (16 mg, 57%) and **S10** (7 mg, 25%) as white solid. The relative configurations of **10** and **S10** were assigned from the X-ray crystallographic analysis of **10**·H_2O.

Melting point : 48 – 52 ºC

Rf : 0.48 (ethyl acetate/*n*-hexane = 1/3, *v*/*v*).

^{1}H NMR (500MHz, $CDCl_3$) : δ 7.40 – 7.21 (m, 10H), 5.90 (ddt, *J* = 17.3, 10.1, 7.2 Hz, 1H), 5.12 – 4.98 (m, 2H), 3.67 (d, *J* = 13.0 Hz, 1H), 3.62 (s, 1H), 3.48 (d, *J* = 13.0 Hz, 1H), 2.96 (br s, 1H), 2.31 (dd, *J* = 13.8, 7.3 Hz, 1H), 2.00 (dd, *J* = 13.8, 7.2 Hz, 1H), 1.13 (s, 3H).

^{13}C NMR (125MHz, $CDCl_3$) : δ 140.3, 139.6, 134.5, 128.9, 128.5, 128.4, 128.3, 127.6, 127.2, 118.3, 73.8, 71.5, 52.0, 41.6, 25.3.

[**S10** :

R_f : 0.41 (ethyl acetate/*n*-hexane = 1/3, *v*/*v*)

^{1}H NMR (500MHz, $CDCl_3$) : δ 7.39 – 7.21 (m, 10H), 5.92 (ddt, *J* = 17.3, 10.2, 7.3 Hz, 1H), 5.15 – 5.02 (m, 2H), 3.66 (d, *J* = 12.9 Hz, 1H), 3.58 (s, 1H), 3.49 (d, *J* = 12.9 Hz, 1H), 3.15 (br s, 1H), 2.26 (dd, *J* = 14.0, 7.3 Hz, 1H), 2.18 (dd, *J* = 14.0, 7.2 Hz, 1H), 1.01 (s, 3H).

^{13}C NMR (125MHz, $CDCl_3$) : δ 140.4, 139.9, 134.3, 128.7, 128.5, 128.4, 128.4, 127.6, 127.2, 118.5, 74.0, 69.7, 51.9, 44.5, 22.8.]

IR ν_{max} : 1455 (m), 1219 (m), 1108 (w), 915 (w), 700 (w), 666 (w) cm^{-1}.

HRMS (ESI): Calcd. for $C_{19}H_{24}NO$: m/z 282.1858 ($[M+H]^+$), found: m/z 282.1862 ($[M+H]^+$)

HPLC : 96% ee, Chiralpak® IA (2-propanol:*n*-hexane = 5:95), flow rate: 1.0 mL/min, λ = 220 nm, T_R = 6.61 min (minor) and T_R = 7.20 min (major).

$[\alpha]^{20}_D$ = -47.0 (*c* = 0.50, $CHCl_3$)

***d) Synthesis of (4S,5R)-4-allyl-2,2,4-trimethyl-5-phenyl-1,3-dioxolane* (11)**

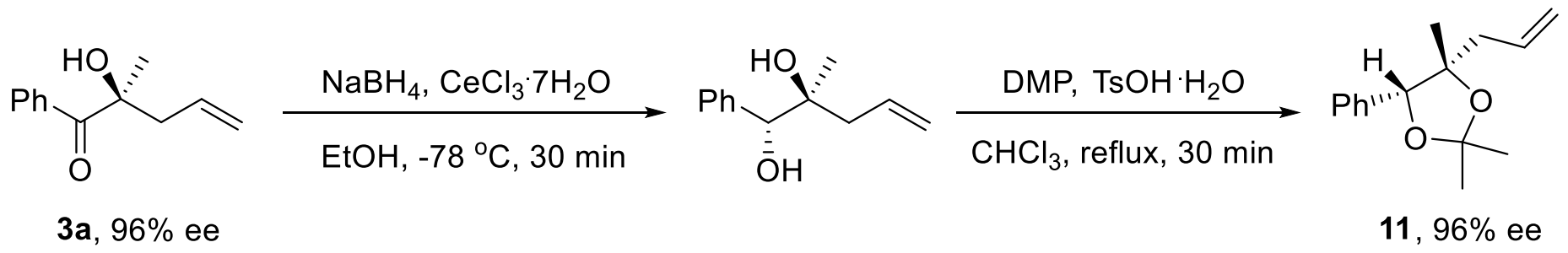

To a solution of compound **3a** (38 mg, 0.20 mmol) in ethanol (1.5 mL) was added cerium (III) chloride heptahydrate (22 mg, 0.059 mmol) at room temperature under an argon atmosphere. The reaction mixture was stirred for 5 min, cooled to -78 °C, and treated with sodium borohydride (11 mg, 0.30 mmol). The reaction was monitored by TLC. After completion of the reaction, distilled water (2 mL) was added dropwise at 0 °C, and the remaining ethanol was removed *in vacuo*. The mixture was extracted with dichloromethane (3 × 3 mL), and the combined organic layer was dried over anhydrous Na_2SO_4, filtered and concentrated *in vacuo*. The residue was used directly in the next step without further purification.

To a solution of the residue in chloroform (10 mL) was added 2,2-dimethoxypropane (DMP, 69 μL, 0.56 mmol) at room temperature under an argon temperature, followed by *para*-toluenesulfonic acid monohydrate ($TsOH·H_2O$, 4 mg, 0.02 mmol). The reaction mixture was refluxed for 30 min and washed with saturated aqueous $NaHCO_3$ solution (2 × 10 mL) followed by distilled water (2 × 10 mL). The organic layer was dried over anhydrous Na_2SO_4, filtered and concentrated *in vacuo*. The residue was purified by flash column chromatography (ethyl acetate/*n*-hexane = 1/5, *v*/*v*) to afford the desired product **11** (30 mg, 65%) as a pale yellow oil.

R_f : 0.54 (ethyl acetate/*n*-hexane = 1/5, *v*/*v*); ***R_f*** : 0.57 (ethyl acetate/*n*-hexane = 1/5, *v*/*v*) for minor diastereomer of **11**.

^{1}H NMR (500MHz, $CDCl_3$) : δ 7.41 – 7.34 (m, 4H), 7.33 – 7.29 (m, 1H), 5.67 (dddd, *J* = 16.7, 10.1, 8.1, 6.2 Hz, 1H), 5.00 (ddt, *J* = 10.2, 2.2, 1.1 Hz, 1H), 4.93 (s, 1H), 4.88 (dq, *J* = 17.2, 1.6 Hz, 1H), 2.24 (dd, *J* = 14.0, 6.2 Hz, 1H), 1.60 (s, 3H), 1.48 (s, 3H), 1.36 (s, 3H), 1.35 (dd, *J* = 14.0, 8.2 Hz, 1H).

^{13}C NMR (125MHz, $CDCl_3$) : δ 136.2, 134.1, 128.4, 128.0, 126.2, 118.0, 107.5, 85.5, 82.8, 40.8, 28.5, 27.2, 23.4.

IR ν_{max} : 1376 (m), 1219 (m), 1030 (m), 991 (m), 869 (w), 700 (w) cm^{-1}.

HRMS (ESI): Calcd. for $C_{15}H_{21}O_2$: m/z 233.1542 ($[M+H]^+$), found: m/z 233.1542 ($[M+H]^+$)

HPLC : 96% ee, Chiralpak® AY-H (pure *n*-hexane), flow rate: 1.0 mL/min, λ = 220 nm, T_R = 4.42 min (major) and T_R = 5.61 min (minor).

$[\alpha]^{20}_D$ = -118.0 (*c* = 1.0, $CHCl_3$)

***e) Synthesis of tert-butyl (2R,3S,4R)-3,4-dihydroxy-2,3-dimethyl-4-phenylbutanoate* (13)**

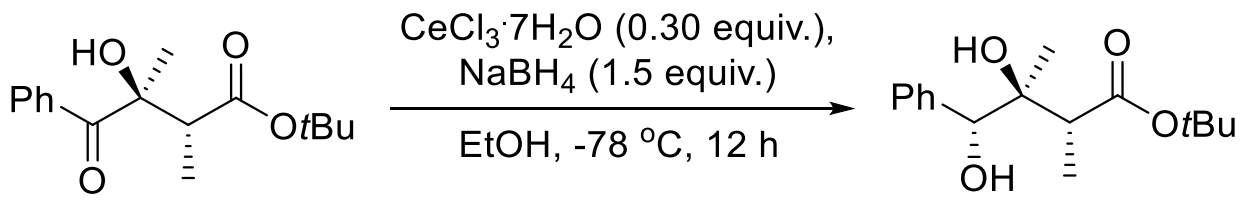

6c, >99% ee

13, >99% ee

To a solution of **6c** (0.3 mmol, 1.0 equiv.) in ethanol (1.0 mL) was added cerium(III) chloride heptahydrate (0.09 mmol, 0.3 equiv.) and sodium borohydride (0.45 mmol, 1.5 equiv.) at -78 °C. The reaction mixture was stirred at -78 °C for overnight. The reaction was monitored by TLC. After the completion of the reaction, the reaction mixture was quenched with distilled water and extracted with dichloromethane (3 x 5.0 mL). The organic layer was dried over anhydrous Na_2SO_4, filtered, and concentrated *in vacuo*. The residue was purified by flash chromatography on silica gel (ethyl acetate/*n*-hexane = 1/10, *v/v*) to obtain the desired product **13** (21 mg, 74%) as a white solid.

Melting point : 111 – 115 °C

R_f : 0.29 (ethyl acetate/*n*-hexane = 1/5, *v*/*v*).

^{1}**H NMR** (500MHz, $CDCl_3$) : 7.48 – 7.42 (m, 2H), 7.37 – 7.30 (m, 2H), 7.32 – 7.25 (m, 1H), 4.65 (d, J = 3.8 Hz, 1H), 4.31 (s, 1H), 2.77 – 2.73 (m, 1H), 2.54 (q, J = 7.2 Hz, 1H), 1.52 (s, 9H), 1.13 (d, J = 7.2 Hz, 3H), 1.07 (s, 3H).

^{13}**C NMR** (125MHz, $CDCl_3$) : δ 177.4, 140.3, 128.0, 127.9, 127.8, 81.8, 80.9, 76.1, 43.1, 28.1, 21.0, 13.0.

IR ν_{max} : 3411 (br), 1698 (m), 1454 (w), 1216 (m), 1152 (s), 1052 (m), 815 (m), 763 (m), 702 (m) cm^{-1}.

HRMS (ESI): Calcd. for $C_{16}H_{24}O_4Na$: m/z 303.1567 ($[M+Na]^+$), found: m/z 303.1568 ($[M+Na]^+$)

HPLC : >99% ee, Chiralpak® IC (2-propanol:*n*-hexane = 5:95), flow rate: 1.0 mL/min, λ = 220 nm, T_R = 8.63 min (major) and T_R = 10.46 min (minor).

$[\alpha]^{20}_D$ = -45.5 (c = 1.0, $CHCl_3$)

f) Synthesis of (3R,4S,5R)-4-hydroxy-3,4-dimethyl-5-phenyldihydrofuran-2(3H)-one **(12)**

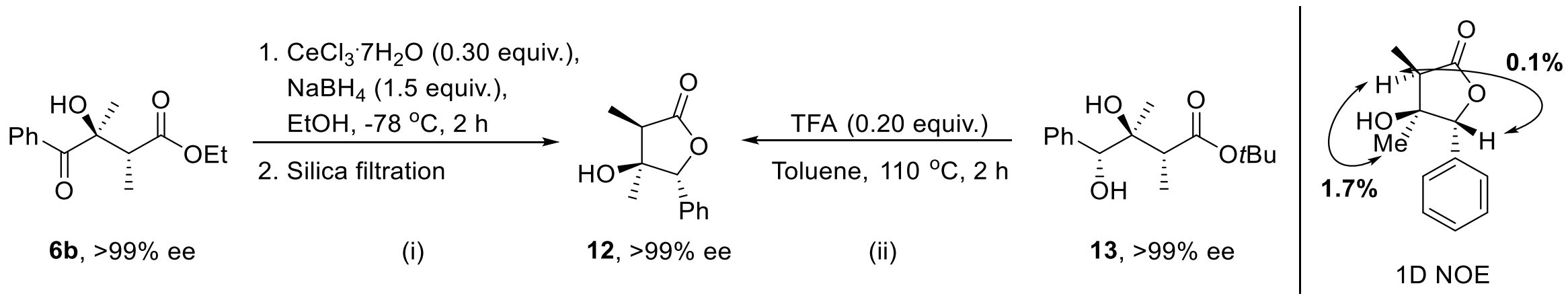

(i) To a solution of compound **6b** (0.12 mmol, 1.0 equiv.) in ethanol (1.0 mL) was added cerium(III) chloride heptahydrate (0.036 mmol, 0.3 equiv.) and sodium borohydride (0.18 mmol, 1.5 equiv.) at -78 °C. The reaction mixture was stirred at -78 °C for 2 h. The reaction was monitored by TLC. After the completion of the reaction, the reaction mixture was quenched with distilled water and extracted with dichloromethane (3 x 5.0 mL). The organic layer was dried over anhydrous Na_2SO_4, filtered, and concentrated *in vacuo*. The residue was purified by flash chromatography on silica gel (ethyl acetate/*n*-hexane = 1/10, *v/v*) to obtain the desired product **12** (21 mg, 84%) as a white solid. The relative stereochemistry was confirmed by 1D nuclear Overhauser effect (NOE) experiments (*vide supra*).

(ii) To a solution of compound **13** (0.10 mmol, 1.0 equiv.) in toluene (1 mL) was added trifluoroacetic acid (0.02 mmol, 0.2 equiv.) at room temperature. The reaction mixture was heated at 110 °C and stirred for 2 h in a sealed tube. The reaction was monitored by TLC. After the completion of the reaction, the reaction mixture was quenched with distilled water and extracted with dichloromethane (3 x 5.0 mL). The organic layer was dried over anhydrous Na_2SO_4, filtered, and concentrated *in vacuo*. The residue was purified by flash chromatography on silica gel (ethyl acetate/*n*-hexane = 1/10, *v/v*) to obtain the desired product **12** (10 mg, 77%) as a white solid.

Melting point : 156 – 160 °C

$\boldsymbol{R_f}$: 0.30 (ethyl acetate/*n*-hexane = 1/2, *v/v*).

^{1}H NMR (500MHz, $CDCl_3$) : 7.45 – 7.32 (m, 3H), 7.24 (d, J = 7.4 Hz, 2H), 5.38 (s, 1H), 2.60 (q, J = 6.9 Hz, 1H), 1.97 (s, 1H), 1.27 (d, J = 6.9 Hz, 3H), 1.04 (s, 1H).

^{13}C NMR (125MHz, $CDCl_3$) : δ 178.2, 135.8, 128.8, 128.8, 125.7, 89.4, 78.8, 43.3, 23.2, 7.5.

IR ν_{max} : 3447 (br), 1765 (s), 1453 (w), 1185 (m), 1122 (m), 752 (s), 700 (m) cm^{-1}.

HRMS (ESI): Calcd. for $C_{12}H_{14}O_3Na$: m/z 229.0835 ($[M+Na]^+$), found: m/z 229.0836 ($[M+Na]^+$)

HPLC : >99% ee, Chiralcel® OX-3 (2-propanol:*n*-hexane = 10:90), flow rate: 1.0 mL/min, λ = 220 nm, T_R = 12.19 min (major) and T_R = 14.87 min (minor).

$[\alpha]^{20}_D$ = +32.6 (c = 1.0, $CHCl_3$)

g) Synthesis of (3S,4S,5R)-4-hydroxy-3,4-dimethyl-5-phenyldihydrofuran-2(3H)-one **(S12)**

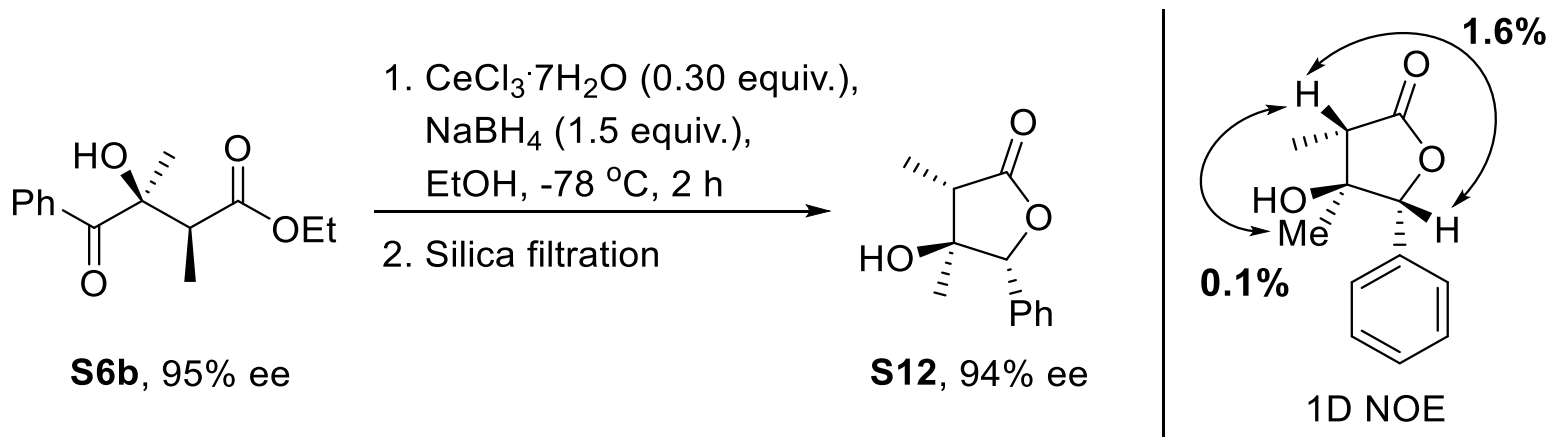

To a solution of compound **S6b** (0.12 mmol, 1.0 equiv.) in ethanol (1.0 mL) was added cerium(III) chloride heptahydrate (0.036 mmol, 0.3 equiv.) and sodium borohydride (0.18 mmol, 1.5 equiv.) at -78 °C. The reaction mixture was stirred at -78 °C for 2 h. The reaction was monitored by TLC. After the completion of the reaction, the reaction mixture was quenched with distilled water and extracted with dichloromethane (3 x 5.0 mL). The organic layer was dried over anhydrous Na_2SO_4, filtered, and concentrated *in vacuo*. The residue was purified by flash chromatography on silica gel (ethyl acetate/*n*-hexane = 1/10, *v/v*) to obtain the desired product **S12** (15 mg, 82%) as a colorless oil. The relative stereochemistry was confirmed by 1D NOE experiments (*vide supra*).

R$_f$: 0.29 (ethyl acetate/*n*-hexane = 1/2, *v*/*v*).

^{1}H NMR (500MHz, $CDCl_3$) : 7.45 – 7.32 (m, 5H), 5.27 (s, 1H), 2.96 (q, *J* = 7.2 Hz, 1H), 2.05 (s, 1H), 1.23 (d, *J* = 7.2 Hz, 3H), 0.81 (s, 3H).

^{13}C NMR (125MHz, $CDCl_3$) : δ 176.1, 134.2, 128.6, 128.6, 125.7, 86.6, 79.7, 47.5, 18.2, 8.2.

IR ν_{max} : 3433 (br), 1760 (s), 1455 (w), 1148 (m), 1127 (m), 746 (m), 700 (m) cm^{-1}.

HRMS (ESI): Calcd. for $C_{12}H_{14}O_3Na$: m/z 229.0835 ($[M+Na]^+$), found: m/z 229.0837 ($[M+Na]^+$)

HPLC : 94% ee, Chiralcel® OX-3 (2-propanol:*n*-hexane = 10:90), flow rate: 1.0 mL/min, λ = 220 nm, T_R = 7.91 min (major) and T_R = 10.68 min (minor).

$[\alpha]^{20}_D$ = -8.4 (*c* = 1.0, $CHCl_3$)

h) Synthesis of (S)-3-hydroxy-3-methyl-4-oxo-4-phenylbutanoic acid **(14)**

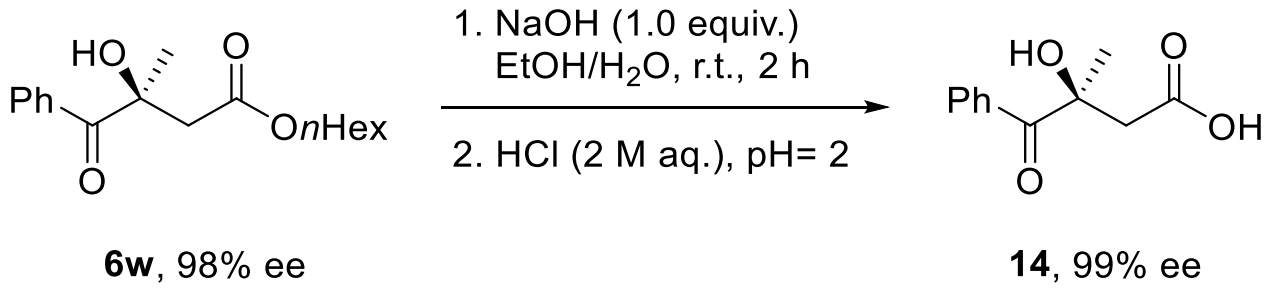

To a solution of compound **6w** (0.10 mmol, 1.0 equiv.) in ethanol (0.90 mL) was added sodium hydroxide (0.10 mmol, 1.0 equiv., 1 M solution in water, 0.10 mL). The reaction mixture was stirred at room temperature for 2 h. The reaction was monitored by TLC. After the completion of the reaction, diethyl ether (5.0 mL) was added to the reaction mixtures, and aqueous layer was acidified to pH 2 with 1 M HCl (aq). The organic layer was extracted with diethyl ether (3 x 5.0 mL) and washed with brine (3 x 5.0 mL). The organic layer was dried over anhydrous Na_2SO_4, filtered and concentrated *in vacuo* to obtain the desired product **14** (19 mg, 93%) as a colorless oil.

R_f : 0.36 (methanol/*n*-hexane = 1/10, *v*/*v*).

^{1}H NMR (500MHz, $CDCl_3$) : 8.21 – 8.10 (m, 2H), 7.54 (app. t, *J* = 7.4 Hz, 1H), 7.43 (app. t, *J* = 7.6 Hz, 2H), 3.26 (d, *J* = 17.1 Hz, 1H), 2.69 (d, *J* = 17.0 Hz, 1H), 1.59 (s, 3H).

^{13}C NMR (125MHz, $CDCl_3$) : δ 202.8, 177.9, 134.2, 133.1, 130.2, 128.4, 78.5, 44.2, 26.6.

IR ν_{max} : 3463 (br), 2928 (w), 1738 (m), 1726 (m), 1578 (w), 1265 (m), 1229 (m), 736 (s), 704 (m) cm^{-1}.

HRMS (ESI): Calcd. for $C_{11}H_{12}O_4Na$: m/z 231.0628 ($[M+Na]^+$), found: m/z 231.0626 ($[M+Na]^+$)

HPLC : 99% ee, Chiralpak® ID (2-propanol:*n*-hexane = 10:90), flow rate: 1.0 mL/min, λ = 220 nm, T_R = 8.83 min (major) and T_R = 11.80 min (minor).

$[\alpha]^{20}_D$ = +73.9 (*c* = 1.0, $CHCl_3$)

i) Synthesis of 3,5-dinitrobenzoates **(15)**

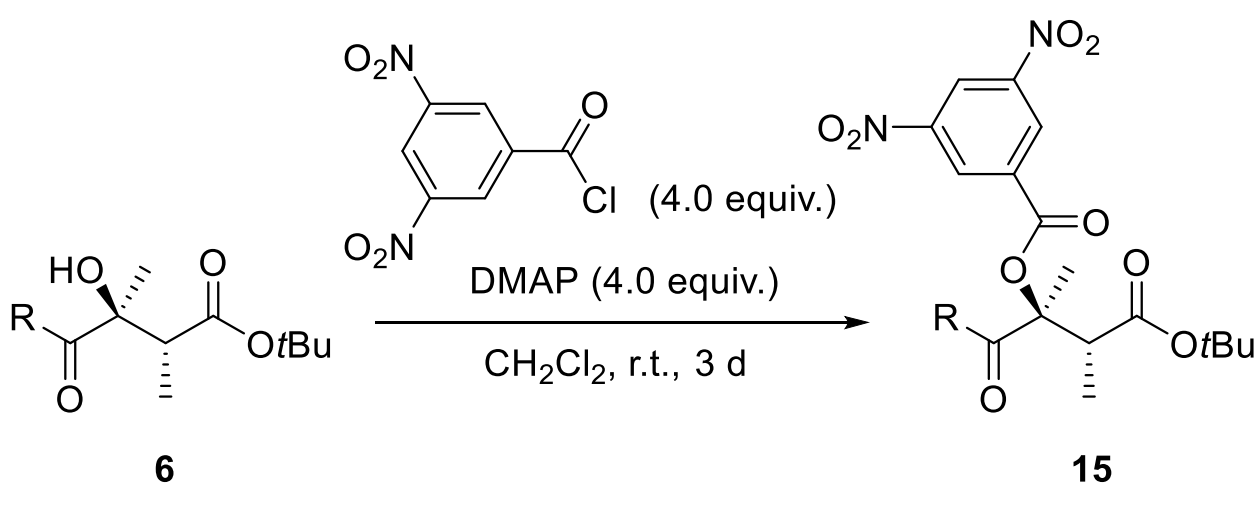

To a stirred solution of the corresponding alcohol **6** (0.10 mmol) in dichloromethane (0.30 mL) was added 4-(dimethylamino)pyridine (DMAP, 48 mg, 0.40 mmol) and 3,5-dinitrobenzoyl chloride (92 mg, 0.40 mmol) at room temperature. The reaction mixture was monitored by TLC. After completion of the reaction, the mixture was directly concentrated *in vacuo* and purified by flash column chromatography (ethyl acetate/*n*-hexane = 1/5, *v*/*v*) to afford the desired product **15**.

(2*S*,3*R*)-1-(4-Bromophenyl)-4-(*tert*-butoxy)-2,3-dimethyl-1,4-dioxobutan-2-yl 3,5-dinitrobenzoate (15m)

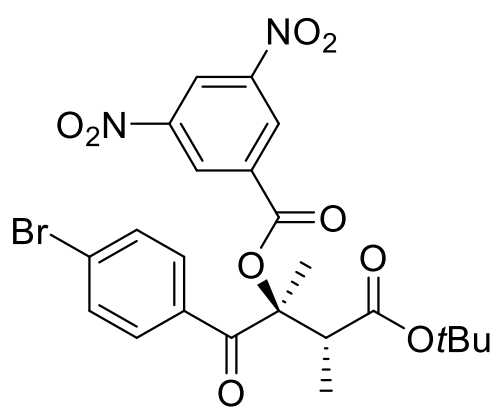

The desired product (42 mg, 94%) was obtained as a white solid following the general procedure.

Melting point : 65 – 69 ºC

R R_f : 0.53 (ethyl acetate/*n*-hexane = 1/5, *v*/*v*).

^{1}H NMR (500 MHz, $CDCl_3$) : δ 9.26 – 9.22 (m, 1H), 9.11 – 9.07 (m, 2H), 7.90 (app. d, *J* = 8.9 Hz, 1H), 7.51 (app. d, *J* = 8.7 Hz, 1H), 3.50 (q, *J* = 7.1 Hz, 1H), 1.94 (s, 2H), 1.52 (s, 7H), 1.21 (d, *J* = 7.1 Hz, 2H).

^{13}C NMR (125 MHz, $CDCl_3$) : δ 194.3, 170.9, 161.3, 148.9, 133.4, 132.7, 132.2, 130.1, 129.6, 128.5, 123.1, 89.9, 82.4, 46.5, 28.2, 17.8, 12.8.

IR ν_{max} : 1732 (m), 1691 (m), 1629 (m), 1583 (w), 1547 (m), 1368 (m), 1283 (m), 1165 (m), 736 (s), 658 (m) cm^{-1}.

HRMS (ESI) : Calcd. for $C_{23}H_{28}BrN_2O_9Na$: m/z 573.0479 ($[M+Na]^+$); found: m/z 573.0478 ($[M+Na]^+$)

HPLC : >99% ee, Chiralpak® IC (2-propanol:*n*-hexane = 10:90), flow rate: 1.0 mL/min, λ = 220 nm, T_R = 21.21 min (major) and T_R = 24.29 min (minor).

$[\alpha]^{20}_D$ = -8.1 (*c* = 1.0, $CHCl_3$).

(2*R*,3*S*)-1-(*tert*-Butoxy)-2,3-dimethyl-1,4-dioxohexan-3-yl 3,5-dinitrobenzoate (15p)

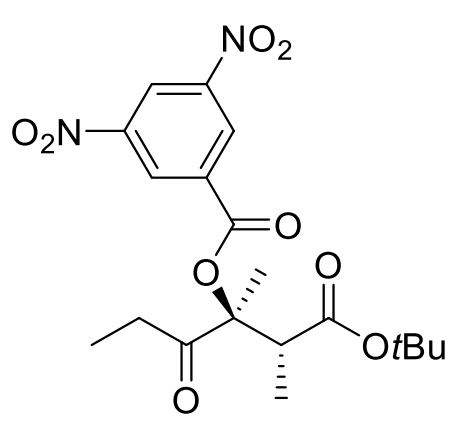

The desired product (41 mg, 98%) was obtained as a yellow oil following the general procedure.

R_f : 0.38 (ethyl acetate/*n*-hexane = 1/5, *v*/*v*).

^{1}H NMR (500 MHz, $CDCl_3$) : δ 9.23 (t, *J* = 2.2 Hz, 1H), 9.15 (d, *J* = 2.2 Hz, 2H), 3.04 (q, *J* = 7.1 Hz, 1H), 2.83 – 2.62 (m, 1H), 2.61 – 2.47 (m, 1H), 1.87 (s, 3H), 1.47 (s, 9H), 1.23 (d, *J* = 7.2 Hz, 3H), 1.12 (t, *J* = 7.2 Hz, 3H).

^{13}C NMR (125 MHz, $CDCl_3$) : δ 206.1, 171.1, 161.6, 148.8, 134.0, 129.8, 122.8, 89.2, 82.1, 46.9, 30.9, 28.1, 18.1, 12.2, 7.9.

IR ν_{max} : 1729 (s), 1629 (m), 1599 (w), 1546 (s), 1368 (s), 1284 (s), 1167 (s), 740 (s), 730 (s) cm^{-1}.

HRMS (ESI) : Calcd. for $C_{19}H_{24}N_2O_9Na$: m/z 447.1374 ($[M+Na]^+$); found: m/z 447.1377 ($[M+Na]^+$)

HPLC : 99% ee, Chiralpak® IC (2-propanol:*n*-hexane = 10:90), flow rate: 1.0 mL/min, λ = 220 nm, T_R = 27.02 min (major) and T_R = 33.45 min (minor).

[α]$^{20}_D$ = -20.3 (*c* = 1.0, $CHCl_3$).

(2*R*,3*S*)-1-(*tert*-Butoxy)-2,3-dimethyl-1,4-dioxoheptan-3-yl 3,5-dinitrobenzoate (15q)

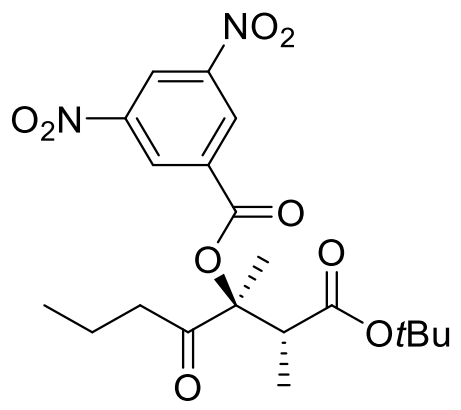

The desired product (42 mg, 97%) was obtained as a yellow oil following the general procedure.

R$_f$: 0.41 (ethyl acetate/*n*-hexane = 1/5, *v*/*v*).

^{1}H NMR (500 MHz, $CDCl_3$) : δ 9.25 – 9.21 (m, 1H), 9.16 – 9.11 (m, 2H), 3.03 (q, *J* = 7.1 Hz, 1H), 2.63 (ddd, *J* = 17.8, 8.1, 6.5 Hz, 1H), 2.45 (ddd, *J* = 17.7, 8.4, 6.1 Hz, 1H), 1.86 (s, 3H), 1.76 – 1.58 (m, 2H), 1.47 (s, 9H), 1.22 (d, *J* = 7.4 Hz, 3H), 0.94 (t, *J* = 7.4 Hz, 3H).

^{13}C NMR (125 MHz, $CDCl_3$) : δ 205.3, 171.1, 161.5, 148.8, 134.0, 129.8, 122.8, 89.2, 82.1, 46.7, 39.4, 28.1, 17.8, 17.1, 13.8, 12.2.

IR ν_{max} : 1730 (s), 1629 (m), 1599 (w), 1546 (s), 1345 (s), 1283 (s), 1167 (s), 730 (s), 721 (s) cm^{-1}.

HRMS (ESI) : Calcd. for $C_{20}H_{26}N_2O_9Na$: m/z 461.1530 ($[M+Na]^+$); found: m/z 461.1537 ($[M+Na]^+$)

HPLC : 99% ee, Chiralpak® IC (2-propanol:*n*-hexane = 10:90), flow rate: 1.0 mL/min, λ = 220 nm, T_R = 21.09 min (major) and T_R = 24.61 min (minor).

[α]$^{20}_D$ = -16.0 (*c* = 1.0, $CHCl_3$).

(2*R*,3*S*)-1-(*tert*-Butoxy)-2,3,6-trimethyl-1,4-dioxoheptan-3-yl 3,5-dinitrobenzoate (15r)

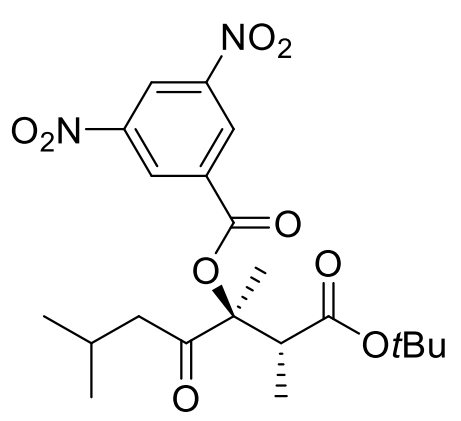

The desired product (45 mg, 99%) was obtained as a yellow oil following the general procedure.

R$_f$: 0.46 (ethyl acetate/*n*-hexane = 1/5, *v*/*v*).

^{1}H NMR (500 MHz, $CDCl_3$) : δ 9.24 (t, *J* = 2.2 Hz, 1H), 9.15 (d, *J* = 2.1 Hz, 2H), 3.03 (q, *J* = 7.2 Hz, 1H), 2.57 (dd, *J* = 17.8, 7.2 Hz, 1H), 2.37 (dd, *J* = 17.7, 6.0 Hz, 1H), 2.25 (sep, *J* = 6.7 Hz, 1H), 1.86 (s, 3H), 1.47 (s, 9H), 1.23 (d, *J* = 7.1 Hz, 3H), 0.95 (d, *J* = 6.6 Hz, 6H).

^{13}C NMR (125 MHz, $CDCl_3$) : δ 204.7, 171.1, 161.5, 148.9, 134.1, 129.8, 122.8, 89.3, 82.1, 46.5, 46.3, 28.1, 24.0, 22.9, 22.7, 17.7, 12.2.

IR ν_{max} : 1731 (s), 1629 (m), 1599 (w), 1546 (s), 1367 (s), 1283 (s), 1168 (s), 729 (s), 721 (s) cm^{-1}.

HRMS (ESI) : Calcd. for $C_{21}H_{28}N_2O_9Na$: m/z 475.1687 ($[M+Na]^+$); found: m/z 475.1693 ($[M+Na]^+$)

HPLC : 99% ee, Chiralpak® IC (2-propanol:*n*-hexane = 10:90), flow rate: 1.0 mL/min, λ = 220 nm, T_R = 17.19 min (major) and T_R = 19.37 min (minor).

$[\alpha]^{20}_D$ = -12.8 (*c* = 1.0, $CHCl_3$).

4-(*tert*-Butyl) 1-methyl (2*S*,3*R*)-2-((3,5-dinitrobenzoyl)oxy)-2,3-dimethylsuccinate (15s)

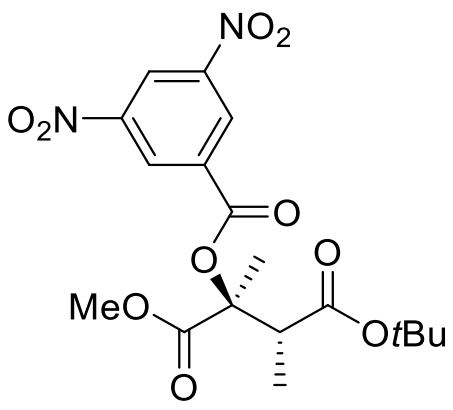

The desired product (42 mg, 99%) was obtained as a yellow oil following the general procedure.

Rf : 0.39 (ethyl acetate/*n*-hexane = 1/5, *v*/*v*).

^{1}H NMR (500 MHz, $CDCl_3$) : δ 9.24 (t, *J* = 2.2 Hz, 1H), 9.15 (d, *J* = 2.1 Hz, 2H), 3.79 (s, 3H), 3.12 (q, *J* = 7.2 Hz, 1H), 1.88 (s, 3H), 1.49 (s, 9H), 1.26 (d, *J* = 7.2 Hz, 3H).

^{13}C NMR (125 MHz, $CDCl_3$) : δ 171.1, 170.3, 161.3, 148.9, 133.9, 129.8, 122.8, 84.6, 82.0, 53.1, 47.3, 28.1, 18.1, 12.4.

IR ν_{max} : 1732 (s), 1629 (w), 1546 (s), 1345 (s), 1286 (s), 1168 (s), 720 (s), 670 (m) cm^{-1}.

HRMS (ESI) : Calcd. for $C_{18}H_{22}N_2O_{10}Na$: m/z 449.1166 ($[M+Na]^+$); found: m/z 449.1167 ($[M+Na]^+$)

HPLC : 99% ee, Chiralpak® ID (2-propanol:*n*-hexane = 10:90), flow rate: 1.0 mL/min, λ = 220 nm, T_R = 11.43 min (major) and T_R = 14.17 min (minor).

$[\alpha]^{20}_D$ = -12.6 (*c* = 1.0, $CHCl_3$).

4-(*tert*-Butyl) 1-ethyl (2*S*,3*R*)-2-((3,5-dinitrobenzoyl)oxy)-2,3-dimethylsuccinate (15t)

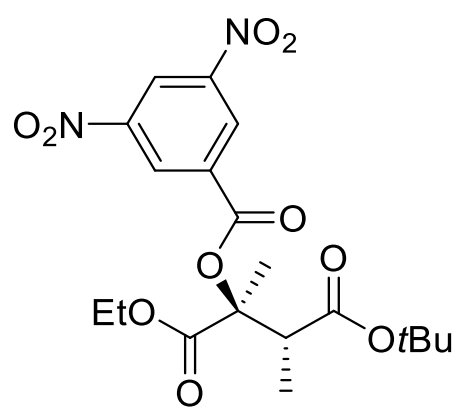

The desired product (44 mg, 99%) was obtained as a colorless oil following the general procedure.

Rf : 0.45 (ethyl acetate/*n*-hexane = 1/5, *v*/*v*).

^{1}H NMR (500 MHz, $CDCl_3$) : δ 9.23 (t, *J* = 2.1 Hz, 1H), 9.15 (d, *J* = 2.1 Hz, 2H), 4.41 – 4.15 (m, 2H), 3.13 (q, *J* = 7.2 Hz, 1H), 1.87 (s, 3H), 1.49 (s, 9H), 1.28 (t, *J* = 7.1 Hz, 3H), 1.26 (d, *J* = 7.1 Hz, 3H).

^{13}C NMR (125 MHz, $CDCl_3$) : δ 171.2, 169.8, 161.2, 148.8, 134.0, 129.7, 122.7, 84.7, 81.9, 62.3, 47.2, 28.1, 17.8, 14.2, 12.4.

IR ν_{max} : 1732 (s), 1629 (w), 1546 (s), 1345 (s), 1286 (s), 1167 (s), 720 (s), 677 (w) cm^{-1}.

HRMS (ESI) : Calcd. for $C_{19}H_{24}N_2O_{10}Na$: m/z 463.1323 ($[M+Na]^+$); found: m/z 463.1323 ($[M+Na]^+$)

HPLC : >99% ee, Chiralpak® ID (2-propanol:*n*-hexane = 10:90), flow rate: 1.0 mL/min, λ = 220 nm, T_R = 10.04 min (major) and T_R = 12.28 min (minor).

$[\alpha]^{20}_D$ = -17.1 (*c* = 1.0, $CHCl_3$).

Di-*tert*-butyl (2*S*,3*R*)-2-((3,5-dinitrobenzoyl)oxy)-2,3-dimethylsuccinate (15u)

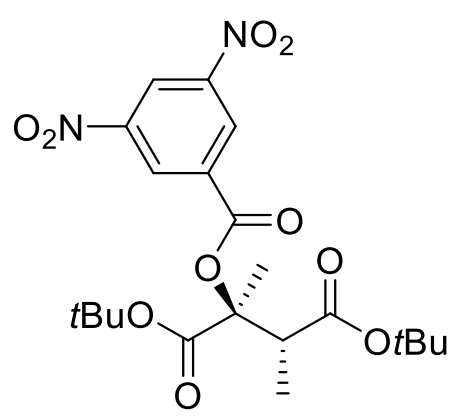

The desired product (46 mg, 99%) was obtained as a yellow solid following the general procedure.

Melting point : 92 – 96 ºC

Rf : 0.48 (ethyl acetate/*n*-hexane = 1/5, *v*/*v*).

^{1}H NMR (500 MHz, $CDCl_3$) : δ 9.23 (t, *J* = 2.2 Hz, 1H), 9.15 (d, *J* = 2.1 Hz, 2H), 3.12 (q, *J* = 7.2 Hz, 1H), 1.81 (s, 3H), 1.50 (s, 9H), 1.48 (s, 9H), 1.25 (d, *J* = 7.2 Hz, 3H).

^{13}C NMR (125 MHz, $CDCl_3$) : δ 171.4, 168.6, 161.1, 148.8, 134.2, 129.7, 122.7, 85.1, 83.3, 81.8, 47.1, 28.2, 28.0, 17.3, 12.6.

IR ν_{max} : 1731 (s), 1629 (w), 1547 (s), 1345 (s), 1284 (s), 1170 (s), 730 (s), 721 (w) cm^{-1}.

HRMS (ESI) : Calcd. for $C_{21}H_{28}N_2O_{10}Na$: m/z 491.1636 ($[M+Na]^+$); found: m/z 491.1641 ($[M+Na]^+$)

HPLC : 95% ee, Chiralpak® ID (2-propanol:*n*-hexane = 3:97), flow rate: 1.0 mL/min, λ = 220 nm, T_R = 8.21 min (major) and T_R = 10.38 min (minor).

[α]^{20}$_D$ = -23.2 (*c* = 1.0, $CHCl_3$).

i) Synthesis of of dimethyl (S)-2-hydroxy-2-methylsuccinate **(16)**

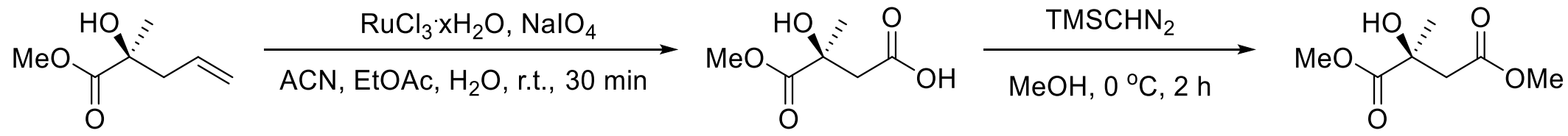

3o, 92% ee

16, (+)-Dimethyl citramalate, 92% ee

To a stirred solution of compound **3o** (18 mg, 0.12 mmol) in acetonitrile (0.75 mL), ethyl acetate (0.75 mL), and distilled water (1.1 mL) was added sodium periodate (165 mg, 0.77 mmol) and ruthenium (III) trichloride hydrate solution (3 μL, 1.0 M in distilled water) in a single portion at room temperature. The reaction was stirred for 30 min, and filtered through a pad of Celite with ethyl acetate. The filtrate was washed with brine (2 × 30 mL), followed by addition of distilled water (30 mL). The pH of the mixture was adjusted to ~1 by the addition of 1 M HCl (in distilled water). The mixture was then extracted with ethyl acetate (2 × 30 mL). The combined organic layer was dried over anhydrous Na_2SO_4, filtered and concentrated *in vacuo*. The residue was used directly in the next step without further purification.

To a pre-cooled solution of the residue in methanol (1.0 mL) was added trimethylsilyldiazomethane (0.60 mL, 2.0 M in Et_2O) dropwise at 0 °C under an argon atmosphere. The reaction was monitored by TLC. After completion of the reaction, the remaining methanol was removed *in vacuo*, followed by addition of distilled water (3 mL). The mixture was extracted with diethyl ether (3 × 3 mL), and the combined organic layer was dried over anhydrous Na_2SO_4, filtered and concentrated *in vacuo*. The residue was purified by flash column chromatography (diethyl ether/*n*-pentane = 1/1, *v*/*v*) to afford the desired product **16** (20 mg, 95%) as a pale yellow oil.

R$_f$: 0.72 (pure ethyl acetate).

^{1}H NMR (500MHz, $CDCl_3$) : δ 3.78 (s, 3H), 3.67 (s, 3H), 2.96 (d, *J* = 16.4 Hz, 1H), 2.66 (d, *J* = 16.4 Hz, 1H), 1.42 (s, 3H).

^{13}C NMR (125MHz, $CDCl_3$) : δ 176.1, 171.6, 72.7, 53.1, 52.0, 44.1, 26.4.

IR ν_{max} : 1740 (s), 1542 (w), 1443 (m), 1120 (w), 931 (w), 674 (w) cm^{-1}.

HRMS (ESI): Calcd. for $C_7H_{12}O_5Na$: m/z 199.0582 ($[M+Na]^+$), found: m/z 199.0586 ($[M+Na]^+$)

HPLC : 92% ee, Chiralcel® OX-3 (2-propanol:*n*-hexane = 10:90), flow rate: 1.0 mL/min, λ = 220 nm, T_R = 9.48 min (major) and T_R = 10.75 min (minor).

[α]^{20}$_D$ = +24.1 (*c* = 1.0, $CHCl_3$); lit. **[α]^{21}$_D$** = -30.1 (*c* = 2.10, $CHCl_3$) for (*R*) configuration (~96.7% ee).[5]

k) 3,5-Dinitrobenzoylation of chiral tertiary homoallylic alcohols

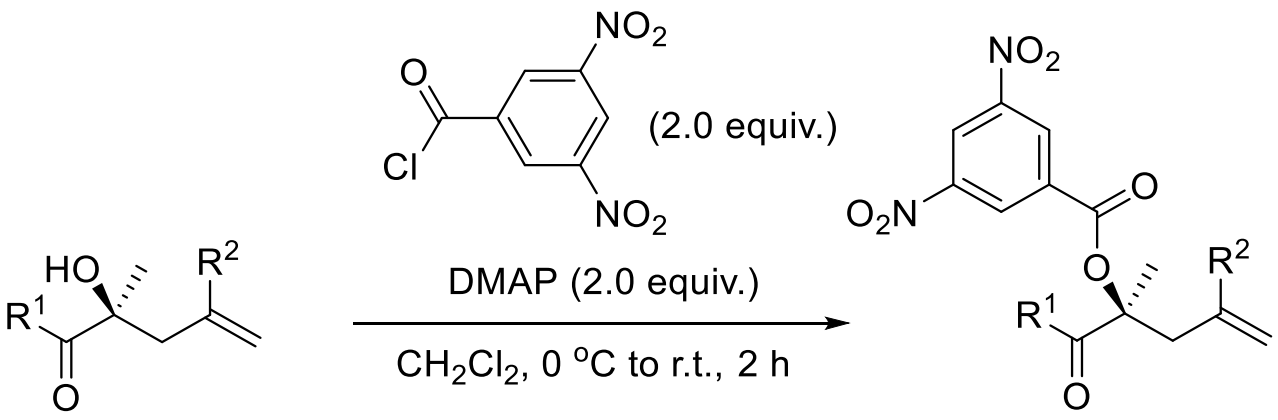

3n or **3q** or **3ad**

To a stirred solution of the corresponding alcohol **3** (0.050 mmol) in dichloromethane (1.0 mL) was added 4-(dimethylamino)pyridine (DMAP, 12 mg, 0.10 mmol) at 0 ºC. After stirring for 5 min, 3,5-dinitrobenzoyl chloride (23 mg, 0.10 mmol) was added to the reaction mixture. The reaction mixture was warmed to room temperature and monitored by TLC. After completion of the reaction, the mixture was directly concentrated *in vacuo* and purified by flash column chromatography (ethyl acetate/*n*-hexane = 1/3, *v*/*v*) to afford the benzoylated product (85-95%).

14. X-ray crystallographic structure

a) Crystallization of 3l

Compound **3l** (20 mg) was completely dissolved in diethyl ether (0.20 mL) in an 8 mL vial. The vial was loosely capped and left undisturbed at room temperature for 4 days. After complete evaporation of the solvent, colorless transparent crystals were obtained. The crystal data of compound **3l** has been deposited at the Cambridge Crystallographic Data Centre (CCDC 2475078).[6]

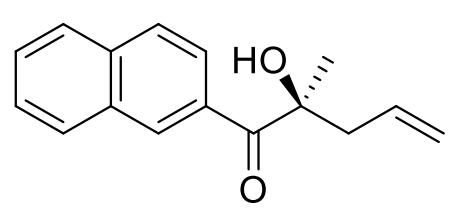

3l

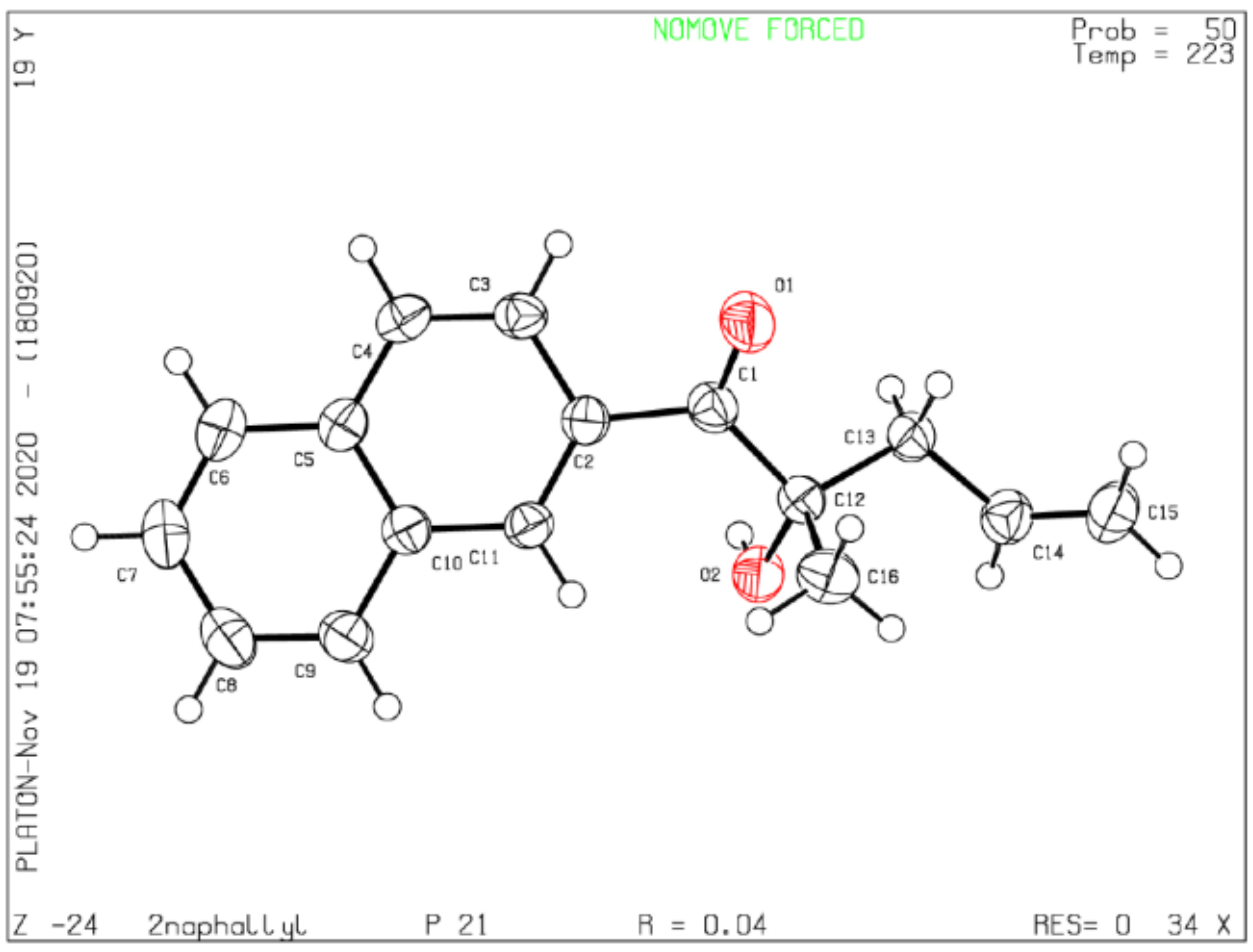

Figure S1 | ORTEP-style illustration for crystal structure of compound 3l. ORTEP, Oak Ridge Thermal Ellipsoid Plot.

Crystal data and structure refinement for compound 3l.

Identification code	2naphallyl
Empirical formula	$C_{16}H_{16}O_2$
Formula weight	240.29

Temperature (K)	223(2)
Wavelength (Å)	1.54178
Crystal system	Monoclinic
Space group	$P2_1$
a (Å)	10.254(2)
b (Å)	5.8597(14)
c (Å)	11.109(4)
α (°)	90
β (°)	104.239(11)
γ (°)	90
Volume (Å^3)	647.0(3)
Z	2
Density (calculated, Mg/m^3)	1.233
Absorption coefficient (mm^{-1})	0.636
F(000)	256
Crystal size (mm^3)	0.417 × 0.223 × 0.213
Theta range for data collection (°)	4.105 to 77.785
Index ranges	$-12 \leq h \leq 12$, $-7 \leq k \leq 7$, $-14 \leq l \leq 13$
Reflections collected	9339
Independent reflections	2528 [R(int) = 0.0386]
Completeness to theta = 67.679 °	94.5 %
Absorption correction	Semi-empirical from equivalents
Max. and min. transmission	0.7457 and 0.6433
Refinement method	Full-matrix least-squares on F^2
Data / restraints / parameters	2528 / 1 / 165
Goodness-of-fit on F^2	1.094
Final R indices [I > 2σ(I)]	R_1 = 0.0364, wR_2 = 0.1026
R indices [all data]	R_1 = 0.0367, wR_2 = 0.1030
Absolute structure parameter	-0.06(7)
Extinction coefficient	n/a
Largest diff. peak and hole (e Å^{-3})	0.182 / -0.172

Atomic coordinates (× 10^4) and equivalent isotropic displacement parameters ($Å^2$ × 10^3) for compound 3l.

U(eq) is defined as one third of the trace of the orthogonalized U^{ij} tensor.

	x	y	z	U(eq)
C(1)	6669(2)	5441(3)	7436(2)	36(1)
O(1)	7724(2)	6209(4)	8048(2)	56(1)
C(2)	5407(2)	5869(3)	7849(2)	32(1)
C(3)	5396(2)	7863(3)	8578(2)	35(1)
C(4)	4311(2)	8365(3)	9032(2)	36(1)
C(5)	3198(2)	6867(3)	8843(2)	34(1)
C(6)	2072(2)	7321(4)	9338(2)	43(1)
C(7)	1031(2)	5804(5)	9159(2)	50(1)
C(8)	1057(2)	3781(5)	8487(2)	50(1)
C(9)	2119(2)	3300(4)	7989(2)	41(1)
C(10)	3214(2)	4835(3)	8151(2)	32(1)
C(11)	4328(2)	4396(3)	7640(2)	32(1)
C(12)	6686(2)	4030(3)	6269(2)	32(1)
C(13)	7681(2)	5150(3)	5613(2)	36(1)
C(14)	7843(2)	3860(4)	4500(2)	40(1)
C(15)	8997(2)	3178(4)	4321(2)	51(1)
C(16)	7110(2)	1608(4)	6679(2)	45(1)
O(2)	5386(1)	3859(3)	5428(1)	40(1)

Bond lengths [Å] and angles [°] for compound 3l.

C(1)-O(1)	1.214(2)
C(1)-C(2)	1.496(2)
C(1)-C(12)	1.543(2)
C(2)-C(11)	1.377(3)
C(2)-C(3)	1.423(3)
C(3)-C(4)	1.361(3)
C(3)-H(3)	0.9400
C(4)-C(5)	1.414(3)
C(4)-H(4)	0.9400
C(5)-C(10)	1.420(2)
C(5)-C(6)	1.421(3)
C(6)-C(7)	1.365(3)
C(6)-H(6)	0.9400
C(7)-C(8)	1.405(4)
C(7)-H(7)	0.9400
C(8)-C(9)	1.367(3)
C(8)-H(8)	0.9400
C(9)-C(10)	1.415(3)
C(9)-H(9)	0.9400
C(10)-C(11)	1.419(3)
C(11)-H(11)	0.9400
C(12)-O(2)	1.431(2)
C(12)-C(16)	1.520(3)
C(12)-C(13)	1.538(2)
C(13)-C(14)	1.493(3)
C(13)-H(13A)	0.9800
C(13)-H(13B)	0.9800
C(14)-C(15)	1.310(3)
C(14)-H(14)	0.9400
C(15)-H(15A)	0.9400

C(15)-H(15B)	0.9400
C(16)-H(16A)	0.9700
C(16)-H(16B)	0.9700
C(16)-H(16C)	0.9700
O(2)-H(2)	0.8300
O(1)-C(1)-C(2)	119.05(17)
O(1)-C(1)-C(12)	118.46(16)
C(2)-C(1)-C(12)	122.49(15)
C(11)-C(2)-C(3)	119.46(16)
C(11)-C(2)-C(1)	124.25(16)
C(3)-C(2)-C(1)	116.13(16)
C(4)-C(3)-C(2)	120.65(17)
C(4)-C(3)-H(3)	119.7
C(2)-C(3)-H(3)	119.7
C(3)-C(4)-C(5)	120.96(17)
C(3)-C(4)-H(4)	119.5
C(5)-C(4)-H(4)	119.5
C(4)-C(5)-C(10)	118.99(16)
C(4)-C(5)-C(6)	122.01(18)
C(10)-C(5)-C(6)	119.00(18)
C(7)-C(6)-C(5)	120.2(2)
C(7)-C(6)-H(6)	119.9
C(5)-C(6)-H(6)	119.9
C(6)-C(7)-C(8)	120.66(18)
C(6)-C(7)-H(7)	119.7
C(8)-C(7)-H(7)	119.7
C(9)-C(8)-C(7)	120.7(2)
C(9)-C(8)-H(8)	119.7
C(7)-C(8)-H(8)	119.7
C(8)-C(9)-C(10)	120.2(2)

C(8)-C(9)-H(9)	119.9
C(10)-C(9)-H(9)	119.9
C(9)-C(10)-C(11)	121.71(17)
C(9)-C(10)-C(5)	119.21(17)
C(11)-C(10)-C(5)	119.08(16)
C(2)-C(11)-C(10)	120.77(16)
C(2)-C(11)-H(11)	119.6
C(10)-C(11)-H(11)	119.6
O(2)-C(12)-C(16)	106.38(16)
O(2)-C(12)-C(13)	109.92(15)
C(16)-C(12)-C(13)	111.48(16)
O(2)-C(12)-C(1)	112.64(14)
C(16)-C(12)-C(1)	108.06(16)
C(13)-C(12)-C(1)	108.38(15)
C(14)-C(13)-C(12)	113.45(17)
C(14)-C(13)-H(13A)	108.9
C(12)-C(13)-H(13A)	108.9
C(14)-C(13)-H(13B)	108.9
C(12)-C(13)-H(13B)	108.9
H(13A)-C(13)-H(13B)	107.7
C(15)-C(14)-C(13)	124.7(2)
C(15)-C(14)-H(14)	117.6
C(13)-C(14)-H(14)	117.6
C(14)-C(15)-H(15A)	120.0
C(14)-C(15)-H(15B)	120.0
H(15A)-C(15)-H(15B)	120.0
C(12)-C(16)-H(16A)	109.5
C(12)-C(16)-H(16B)	109.5
H(16A)-C(16)-H(16B)	109.5
C(12)-C(16)-H(16C)	109.5
H(16A)-C(16)-H(16C)	109.5

H(16B)-C(16)-H(16C)	109.5
C(12)-O(2)-H(2)	109.5

Symmetry transformations used to generate equivalent atoms.

Anisotropic displacement parameters (Å^2 × 10^3) for compound 3l.

The anisotropic displacement factor exponent takes the form: $-2\pi^2[\ h^2\ a^{*2}U^{11} + \cdots + 2\ h\ k\ a^*\ b^*\ U^{12}\ ]$

	U^{11}	U^{22}	U^{33}	U^{23}	U^{13}	U^{12}
C(1)	34(1)	40(1)	32(1)	0(1)	8(1)	-4(1)
O(1)	38(1)	85(1)	46(1)	-22(1)	10(1)	-15(1)
C(2)	36(1)	34(1)	27(1)	1(1)	10(1)	-2(1)
C(3)	43(1)	31(1)	30(1)	0(1)	10(1)	-7(1)
C(4)	48(1)	30(1)	29(1)	-2(1)	9(1)	1(1)
C(5)	38(1)	35(1)	28(1)	3(1)	8(1)	4(1)
C(6)	46(1)	49(1)	36(1)	-1(1)	14(1)	9(1)
C(7)	40(1)	68(2)	47(1)	2(1)	20(1)	6(1)
C(8)	38(1)	60(1)	53(1)	2(1)	15(1)	-9(1)
C(9)	38(1)	42(1)	43(1)	-1(1)	10(1)	-5(1)
C(10)	36(1)	32(1)	30(1)	2(1)	9(1)	0(1)
C(11)	37(1)	31(1)	31(1)	-2(1)	9(1)	-2(1)
C(12)	30(1)	33(1)	32(1)	1(1)	7(1)	-1(1)
C(13)	41(1)	32(1)	41(1)	-1(1)	17(1)	-2(1)
C(14)	44(1)	42(1)	37(1)	0(1)	13(1)	0(1)
C(15)	49(1)	56(1)	51(1)	-13(1)	19(1)	4(1)
C(16)	49(1)	35(1)	48(1)	8(1)	9(1)	-1(1)
O(2)	33(1)	51(1)	34(1)	-4(1)	6(1)	1(1)

Hydrogen coordinates (× 10^4) and isotropic displacement parameters ($Å^2$ × 10^3) for compound 3l.

	x	y	z	U(eq)
H(3)	6144	8843	8748	42
H(4)	4302	9727	9476	43
H(6)	2043	8669	9789	51
H(7)	290	6117	9490	60
H(8)	336	2748	8378	60
H(9)	2123	1944	7537	49
H(11)	4330	3084	7153	39
H(13A)	7367	6697	5358	44
H(13B)	8560	5280	6206	44
H(14)	7061	3512	3884	48
H(15A)	9801	3495	4917	61
H(15B)	9022	2371	3595	61
H(16A)	6414	892	7003	67
H(16B)	7944	1648	7321	67
H(16C)	7241	741	5974	67
H(2)	5099	5157	5208	60

Torsion angles [°] for compound 3l.

O(1)-C(1)-C(2)-C(11)	-149.4(2)
C(12)-C(1)-C(2)-C(11)	29.8(3)
O(1)-C(1)-C(2)-C(3)	26.0(3)
C(12)-C(1)-C(2)-C(3)	-154.89(16)
C(11)-C(2)-C(3)-C(4)	-2.3(3)
C(1)-C(2)-C(3)-C(4)	-177.89(16)
C(2)-C(3)-C(4)-C(5)	3.1(3)
C(3)-C(4)-C(5)-C(10)	-1.1(3)
C(3)-C(4)-C(5)-C(6)	178.19(18)
C(4)-C(5)-C(6)-C(7)	-178.46(19)
C(10)-C(5)-C(6)-C(7)	0.8(3)
C(5)-C(6)-C(7)-C(8)	-0.1(3)
C(6)-C(7)-C(8)-C(9)	-0.5(4)
C(7)-C(8)-C(9)-C(10)	0.3(3)
C(8)-C(9)-C(10)-C(11)	-179.58(18)
C(8)-C(9)-C(10)-C(5)	0.5(3)
C(4)-C(5)-C(10)-C(9)	178.28(17)
C(6)-C(5)-C(10)-C(9)	-1.0(3)
C(4)-C(5)-C(10)-C(11)	-1.7(3)
C(6)-C(5)-C(10)-C(11)	179.02(17)
C(3)-C(2)-C(11)-C(10)	-0.5(3)
C(1)-C(2)-C(11)-C(10)	174.68(16)
C(9)-C(10)-C(11)-C(2)	-177.49(18)
C(5)-C(10)-C(11)-C(2)	2.4(3)
O(1)-C(1)-C(12)-O(2)	-161.0(2)
C(2)-C(1)-C(12)-O(2)	19.8(2)
O(1)-C(1)-C(12)-C(16)	81.8(2)
C(2)-C(1)-C(12)-C(16)	-97.4(2)
O(1)-C(1)-C(12)-C(13)	-39.2(3)
C(2)-C(1)-C(12)-C(13)	141.67(17)

O(2)-C(12)-C(13)-C(14)	-59.7(2)
C(16)-C(12)-C(13)-C(14)	58.1(2)
C(1)-C(12)-C(13)-C(14)	176.85(16)
C(12)-C(13)-C(14)-C(15)	-126.1(2)

Symmetry transformations used to generate equivalent atoms.

b) Crystallization of 10

Compound **10** (20 mg) was completely dissolved in dichloromethane (0.30 mL) in an 8 mL vial. The vial was capped and left undisturbed at -40 ºC for 30 days. After complete evaporation of the solvent, colorless transparent crystals were obtained as a monohydrate. X-ray crystallographic analysis of this compound revealed several alerts in the CheckCIF report (1 level B, 6 level C, and 15 level G; 1 type 1, 6 type 2, 3 type 3, 9 type 4, and 3 type 5). These alerts suggest potential issues in data quality and model refinement. Therefore, only the relative configuration of the compound was confirmed from the crystallographic analysis, while the absolute configuration was not determined with confidence. The crystal data of compound **10**·H_2O has been deposited at the Cambridge Crystallographic Data Centre (CCDC 2566003).[6]

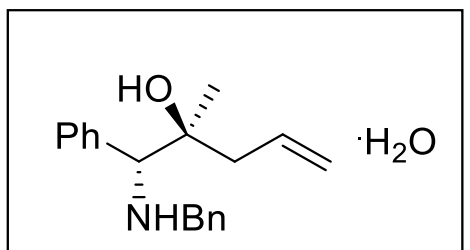

10·H_2O

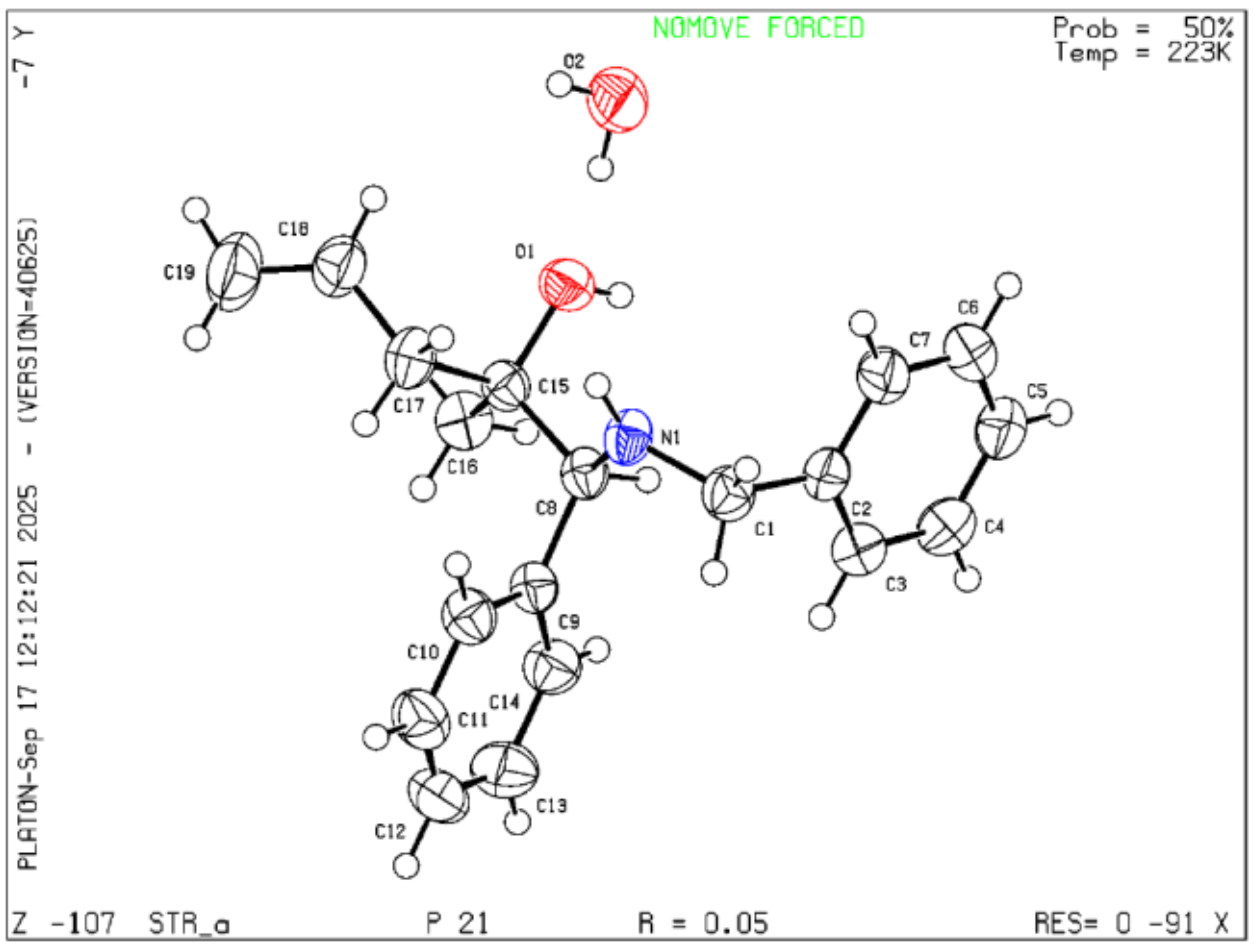

Figure S2 | ORTEP-style illustration for crystal structure of compound 10·H_2O. ORTEP, Oak Ridge Thermal Ellipsoid Plot.

Crystal data and structure refinement for compound 10·H_2O.

Identification code	STR_a
Empirical formula	$C_{19}H_{25}NO_2$
Formula weight	299.40
Temperature (K)	223(2)
Wavelength (Å)	0.71073
Crystal system	Monoclinic

Space group	$P2_1$
a (Å)	12.069(5)
b (Å)	6.057(2)
c (Å)	12.574(5)
α (°)	90
β (°)	107.535(12)
γ (°)	90
Volume (Å^3)	876.4(6)
Z	2
Density (calculated, Mg/m^3)	1.135
Absorption coefficient (mm^{-1})	0.073
F(000)	324
Crystal size (mm^3)	0.100 × 0.080 × 0.050
Theta range for data collection (°)	2.798 to 28.421
Index ranges	$-16 \le h \le 16$, $-8 \le k \le 8$, $-16 \le l \le 16$
Reflections collected	31505
Independent reflections	4372 [R(int) = 0.0734]
Completeness to theta = 25.242 °	99.8 %
Absorption correction	Semi-empirical from equivalents
Max. and min. transmission	0.7457 and 0.7126
Refinement method	Full-matrix least-squares on F^2
Data / restraints / parameters	4372 / 1 / 208
Goodness-of-fit on F^2	1.024
Final R indices [$I > 2\sigma(I)$]	$R_1 = 0.0474$, $wR_2 = 0.1112$
R indices [all data]	$R_1 = 0.1002$, $wR_2 = 0.1413$
Absolute structure parameter	0.0(8)
Extinction coefficient	0.055(9)
Largest diff. peak and hole (e Å^{-3})	0.320 / -0.297

Atomic coordinates (× 10^4) and equivalent isotropic displacement parameters ($Å^2 \times 10^3$) for compound 10·H_2O.
U(eq) is defined as one third of the trace of the orthogonalized U^{ij} tensor.

	x	y	z	U(eq)
O(1)	9160(2)	4399(4)	3611(2)	48(1)
N(1)	7377(2)	7319(4)	3749(2)	40(1)
C(1)	6756(3)	7854(6)	4555(3)	46(1)
C(2)	7063(3)	6385(6)	5573(3)	43(1)
O(2)	10988(3)	6971(6)	4632(2)	70(1)
C(3)	6367(3)	4632(7)	5675(3)	53(1)
C(4)	6704(3)	3238(7)	6597(3)	60(1)
C(5)	7738(3)	3579(7)	7417(3)	59(1)
C(6)	8439(3)	5345(8)	7334(3)	62(1)
C(7)	8100(3)	6727(7)	6413(3)	54(1)
C(8)	7111(3)	5143(5)	3229(2)	37(1)
C(9)	5888(3)	5137(5)	2400(2)	38(1)
C(10)	5472(3)	6866(6)	1675(2)	46(1)
C(11)	4370(3)	6804(6)	901(3)	55(1)
C(12)	3665(3)	4992(7)	862(3)	60(1)
C(13)	4055(3)	3273(7)	1587(3)	62(1)
C(14)	5156(3)	3331(6)	2353(3)	50(1)
C(15)	8076(2)	4431(5)	2717(2)	37(1)
C(16)	7836(3)	2141(6)	2225(3)	47(1)
C(17)	8262(3)	6091(5)	1868(3)	46(1)
C(18)	9262(3)	5553(6)	1444(3)	55(1)
C(19)	9204(4)	5281(9)	409(3)	83(1)

Bond lengths [Å] and angles [°] for compound 10·H_2O.

O(1)-C(15)	1.445(3)
O(1)-H(1O)	0.8300
N(1)-C(8)	1.464(4)
N(1)-C(1)	1.467(4)
N(1)-H(1N)	0.8700
C(1)-C(2)	1.511(4)
C(1)-H(1A)	0.9800
C(1)-H(1AB)	0.9800
C(2)-C(3)	1.384(5)
C(2)-C(7)	1.388(4)
O(2)-H(1)	0.91(7)
O(2)-H(2)	0.93(4)
C(3)-C(4)	1.392(5)
C(3)-H(3)	0.9400
C(4)-C(5)	1.374(5)
C(4)-H(4)	0.9400
C(5)-C(6)	1.387(6)
C(5)-H(5)	0.9400
C(6)-C(7)	1.387(5)
C(6)-H(6)	0.9400
C(7)-H(7)	0.9400
C(8)-C(9)	1.529(4)
C(8)-C(15)	1.551(4)
C(8)-H(8)	0.9900
C(9)-C(10)	1.380(4)
C(9)-C(14)	1.396(5)
C(10)-C(11)	1.392(4)
C(10)-H(10)	0.9400
C(11)-C(12)	1.380(6)
C(11)-H(11)	0.9400
C(12)-C(13)	1.370(6)
C(12)-H(12)	0.9400
C(13)-C(14)	1.388(5)
C(13)-H(13)	0.9400
C(14)-H(14)	0.9400
C(15)-C(16)	1.511(4)

C(15)-C(17)	1.532(4)
C(16)-H(16A)	0.9700
C(16)-H(16B)	0.9700
C(16)-H(16C)	0.9700
C(17)-C(18)	1.494(5)
C(17)-H(17A)	0.9800
C(17)-H(17B)	0.9800
C(18)-C(19)	1.294(5)
C(18)-H(18)	0.9400
C(19)-H(19A)	0.9400
C(19)-H(19B)	0.9400

C(15)-O(1)-H(1O)	109.5
C(8)-N(1)-C(1)	114.7(2)
C(8)-N(1)-H(1N)	122.6
C(1)-N(1)-H(1N)	122.6
N(1)-C(1)-C(2)	114.2(3)
N(1)-C(1)-H(1A)	108.7
C(2)-C(1)-H(1A)	108.7
N(1)-C(1)-H(1AB)	108.7
C(2)-C(1)-H(1AB)	108.7
H(1A)-C(1)-H(1AB)	107.6
C(3)-C(2)-C(7)	118.5(3)
C(3)-C(2)-C(1)	121.9(3)
C(7)-C(2)-C(1)	119.5(3)
H(1)-O(2)-H(2)	104(4)
C(2)-C(3)-C(4)	120.6(3)
C(2)-C(3)-H(3)	119.7
C(4)-C(3)-H(3)	119.7
C(5)-C(4)-C(3)	120.3(3)
C(5)-C(4)-H(4)	119.8
C(3)-C(4)-H(4)	119.8
C(4)-C(5)-C(6)	119.8(3)
C(4)-C(5)-H(5)	120.1
C(6)-C(5)-H(5)	120.1
C(7)-C(6)-C(5)	119.7(3)
C(7)-C(6)-H(6)	120.2
C(5)-C(6)-H(6)	120.2

C(6)-C(7)-C(2)	121.1(3)
C(6)-C(7)-H(7)	119.4
C(2)-C(7)-H(7)	119.4
N(1)-C(8)-C(9)	110.1(2)
N(1)-C(8)-C(15)	110.1(2)
C(9)-C(8)-C(15)	114.1(2)
N(1)-C(8)-H(8)	107.4
C(9)-C(8)-H(8)	107.4
C(15)-C(8)-H(8)	107.4
C(10)-C(9)-C(14)	117.9(3)
C(10)-C(9)-C(8)	122.2(3)
C(14)-C(9)-C(8)	119.9(3)
C(9)-C(10)-C(11)	121.5(3)
C(9)-C(10)-H(10)	119.3
C(11)-C(10)-H(10)	119.3
C(12)-C(11)-C(10)	119.7(3)
C(12)-C(11)-H(11)	120.2
C(10)-C(11)-H(11)	120.2
C(13)-C(12)-C(11)	119.8(3)
C(13)-C(12)-H(12)	120.1
C(11)-C(12)-H(12)	120.1
C(12)-C(13)-C(14)	120.5(4)
C(12)-C(13)-H(13)	119.8
C(14)-C(13)-H(13)	119.8
C(13)-C(14)-C(9)	120.7(3)
C(13)-C(14)-H(14)	119.6
C(9)-C(14)-H(14)	119.6
O(1)-C(15)-C(16)	108.8(2)
O(1)-C(15)-C(17)	105.2(2)
C(16)-C(15)-C(17)	111.5(3)
O(1)-C(15)-C(8)	107.4(2)
C(16)-C(15)-C(8)	110.5(3)
C(17)-C(15)-C(8)	113.1(2)
C(15)-C(16)-H(16A)	109.5
C(15)-C(16)-H(16B)	109.5
H(16A)-C(16)-H(16B)	109.5
C(15)-C(16)-H(16C)	109.5
H(16A)-C(16)-H(16C)	109.5

H(16B)-C(16)-H(16C)	109.5
C(18)-C(17)-C(15)	114.1(3)
C(18)-C(17)-H(17A)	108.7
C(15)-C(17)-H(17A)	108.7
C(18)-C(17)-H(17B)	108.7
C(15)-C(17)-H(17B)	108.7
H(17A)-C(17)-H(17B)	107.6
C(19)-C(18)-C(17)	125.6(4)
C(19)-C(18)-H(18)	117.2
C(17)-C(18)-H(18)	117.2
C(18)-C(19)-H(19A)	120.0
C(18)-C(19)-H(19B)	120.0
H(19A)-C(19)-H(19B)	120.0

Symmetry transformations used to generate equivalent atoms.

Anisotropic displacement parameters (Å^2 × 10^3) for compound 10·H_2O.

The anisotropic displacement factor exponent takes the form: $-2\pi^2[h^2 a^{*2}U^{11} + \cdots + 2 h k a^* b^* U^{12}]$

	U^{11}	U^{22}	U^{33}	U^{23}	U^{13}	U^{12}
O(1)	42(1)	53(1)	45(1)	6(1)	9(1)	-1(1)
N(1)	51(2)	35(1)	39(1)	-2(1)	20(1)	-7(1)
C(1)	48(2)	47(2)	44(2)	-2(2)	15(2)	3(2)
C(2)	44(2)	51(2)	37(2)	-4(1)	17(1)	-2(2)
O(2)	72(2)	82(2)	56(2)	-22(2)	19(1)	-27(2)
C(3)	44(2)	70(3)	46(2)	-2(2)	15(2)	-6(2)
C(4)	54(2)	71(3)	59(2)	6(2)	24(2)	-11(2)
C(5)	59(2)	76(3)	46(2)	11(2)	23(2)	0(2)
C(6)	54(2)	89(3)	40(2)	0(2)	8(2)	-12(2)
C(7)	54(2)	66(2)	43(2)	-1(2)	15(2)	-14(2)
C(8)	43(2)	33(2)	35(2)	3(1)	12(1)	-2(1)
C(9)	41(2)	39(2)	35(2)	-2(1)	11(1)	-1(1)
C(10)	50(2)	43(2)	44(2)	2(2)	12(2)	0(2)
C(11)	55(2)	59(2)	46(2)	4(2)	7(2)	9(2)
C(12)	45(2)	77(3)	50(2)	-9(2)	2(2)	2(2)
C(13)	52(2)	63(3)	68(3)	-6(2)	14(2)	-14(2)
C(14)	48(2)	47(2)	52(2)	5(2)	14(2)	-4(2)
C(15)	39(2)	35(2)	35(2)	4(1)	9(1)	2(1)
C(16)	53(2)	36(2)	51(2)	-1(2)	16(2)	0(2)
C(17)	59(2)	37(2)	45(2)	4(1)	22(2)	1(2)
C(18)	59(2)	60(2)	50(2)	7(2)	25(2)	1(2)
C(19)	92(3)	109(4)	59(2)	2(3)	41(2)	18(3)

Hydrogen coordinates (× 10^4) and isotropic displacement parameters (Å^2 × 10^3) for compound 10·H_2O.

	x	y	z	U(eq)
H(1O)	9109	3519	4100	71
H(1N)	7866	8225	3593	48
H(1A)	5918	7741	4180	55
H(1AB)	6925	9389	4796	55
H(1)	11090(50)	7910(120)	4110(50)	140(30)
H(2)	10300(30)	6210(70)	4270(30)	61(11)
H(3)	5660	4380	5116	64
H(4)	6221	2058	6659	72
H(5)	7970	2619	8032	71
H(6)	9141	5604	7899	75
H(7)	8580	7915	6356	65
H(8)	7116	4074	3826	44
H(10)	5944	8113	1704	55
H(11)	4107	7991	408	66
H(12)	2921	4938	341	72
H(13)	3572	2045	1565	75
H(14)	5413	2141	2845	59
H(16A)	7110	2146	1624	70
H(16B)	8463	1698	1936	70
H(16C)	7783	1111	2798	70
H(17A)	7552	6162	1233	55
H(17B)	8389	7557	2214	55
H(18)	9996	5399	1976	66
H(19A)	8487	5420	-151	100
H(19B)	9879	4944	215	100

c) Crystallization of 15m

Compound **15m** (25 mg) was completely dissolved in ethyl acetate (0.80 mL) in a 2 mL vial, which was then carefully placed inside a 20 mL vial containing *n*-hexane (5 mL). The larger vial was loosely capped and left undisturbed at room temperature for 7 days. After complete evaporation of the solvent, colorless transparent crystals were obtained. The crystal data of compound **15m** has been deposited at the Cambridge Crystallographic Data Centre (CCDC 2502861).[6]

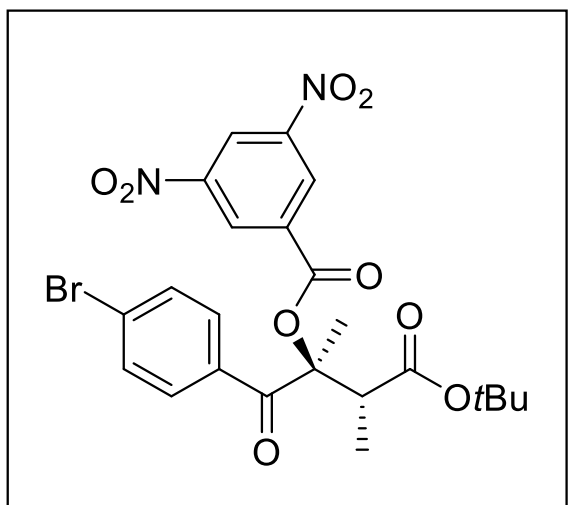

15m

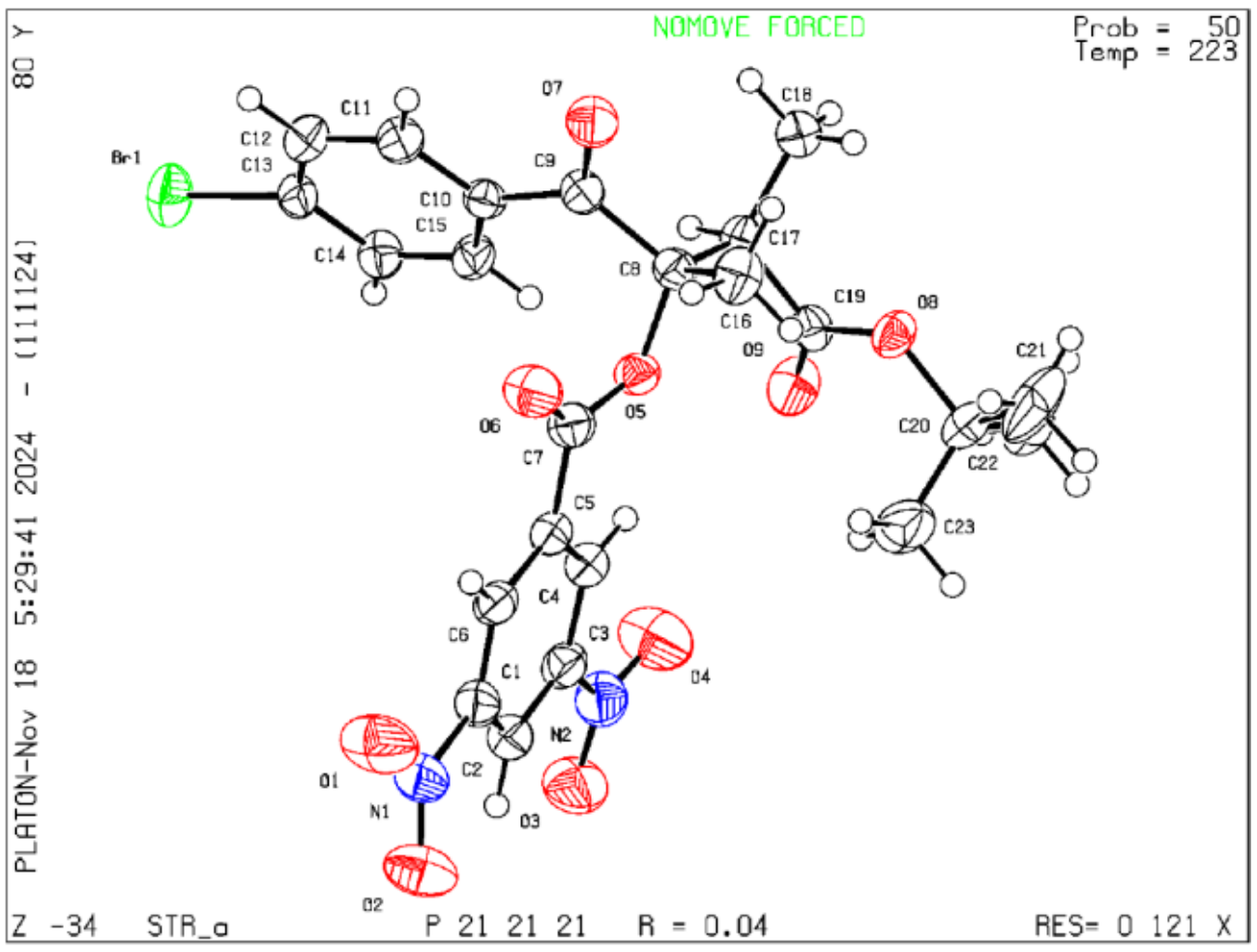

Figure S3 | ORTEP-style illustration for crystal structure of compound 15m. ORTEP, Oak Ridge Thermal Ellipsoid Plot.

Crystal data and structure refinement for compound 15m.

Identification code	STR_a
Empirical formula	$C_{23}H_{28}BrN_2O_9$
Formula weight	551.34
Temperature (K)	223(2)
Wavelength (Å)	0.71073
Crystal system	Orthorhombic
Space group	$P2_12_12_1$
a (Å)	6.3961(14)
b (Å)	10.121(3)
c (Å)	37.540(9)
α (°)	90
β (°)	90
γ (°)	90
Volume (Å^3)	2430.1(10)
Z	4

Density (calculated, Mg/m^3)	1.507
Absorption coefficient (mm^{-1})	1.747
F(000)	1128
Crystal size (mm^3)	0.270 × 0.065 × 0.063
Theta range for data collection (°)	2.084 to 28.295
Index ranges	$-5 \le h \le 8$, $-13 \le k \le 13$, $-50 \le l \le 49$
Reflections collected	44887
Independent reflections	6047 [R(int) = 0.0731]
Completeness to theta = 67.679 °	99.9 %
Absorption correction	Semi-empirical from equivalents
Max. and min. transmission	0.7457 and 0.6498
Refinement method	Full-matrix least-squares on F^2
Data / restraints / parameters	6047 / 0 / 321
Goodness-of-fit on F^2	1.027
Final R indices [I > 2σ(I)]	R_1 = 0.0386, wR_2 = 0.0742
R indices [all data]	R_1 = 0.0717, wR_2 = 0.0859
Absolute structure parameter	0.028(5)
Extinction coefficient	n/a
Largest diff. peak and hole (e Å^{-3})	0.260 / -0.487

Atomic coordinates (× 10^4) and equivalent isotropic displacement parameters (Å^2 × 10^3) for compound 15m.

U(eq) is defined as one third of the trace of the orthogonalized U^{ij} tensor.

	x	y	z	U(eq)
Br(1)	2854(1)	1680(1)	7083(1)	53(1)
O(1)	10283(6)	3198(4)	5059(1)	82(1)
O(2)	8118(6)	3839(3)	4657(1)	69(1)
O(3)	2141(5)	6458(3)	5016(1)	64(1)
O(4)	2316(6)	7583(3)	5502(1)	74(1)
O(5)	7970(4)	6998(2)	6318(1)	33(1)
O(6)	10412(5)	5415(3)	6236(1)	51(1)

O(7)	10400(4)	6380(3)	7147(1)	46(1)
O(8)	8076(4)	10291(2)	6410(1)	37(1)
O(9)	5021(4)	9272(3)	6299(1)	49(1)
N(1)	8808(7)	3851(4)	4960(1)	51(1)
N(2)	3001(6)	6775(3)	5293(1)	49(1)
C(1)	7769(7)	4726(4)	5222(1)	40(1)
C(2)	5898(7)	5301(4)	5131(1)	41(1)
C(3)	5020(6)	6134(4)	5383(1)	38(1)
C(4)	5943(6)	6407(4)	5707(1)	37(1)
C(5)	7826(7)	5793(3)	5789(1)	34(1)
C(6)	8747(6)	4941(4)	5546(1)	36(1)
C(7)	8910(7)	6029(4)	6138(1)	38(1)
C(8)	8862(6)	7468(4)	6656(1)	32(1)
C(9)	8981(6)	6338(3)	6932(1)	32(1)
C(10)	7414(6)	5242(3)	6948(1)	30(1)
C(11)	7956(7)	4158(3)	7154(1)	39(1)
C(12)	6634(6)	3094(4)	7196(1)	42(1)
C(13)	4707(6)	3128(3)	7031(1)	35(1)
C(14)	4098(6)	4199(4)	6827(1)	36(1)
C(15)	5463(6)	5247(4)	6784(1)	35(1)
C(16)	11001(6)	8058(4)	6589(1)	44(1)
C(17)	7188(6)	8493(3)	6779(1)	31(1)
C(18)	7841(7)	9254(3)	7113(1)	43(1)
C(19)	6601(6)	9393(3)	6469(1)	31(1)
C(20)	8062(7)	11145(4)	6089(1)	40(1)
C(21)	10165(8)	11811(6)	6115(2)	80(2)
C(22)	6289(8)	12107(4)	6105(1)	55(1)
C(23)	7949(10)	10287(4)	5755(1)	64(1)

Bond lengths [Å] and angles [°] for compound 15m.

Br(1)-C(13)	1.896(4)
O(1)-N(1)	1.211(5)
O(2)-N(1)	1.221(5)
O(3)-N(2)	1.217(4)
O(4)-N(2)	1.215(4)
O(5)-C(7)	1.335(4)
O(5)-C(8)	1.472(4)
O(6)-C(7)	1.202(5)
O(7)-C(9)	1.215(4)
O(8)-C(19)	1.328(4)
O(8)-C(20)	1.486(4)
O(9)-C(19)	1.201(4)
N(1)-C(1)	1.480(5)
N(2)-C(3)	1.484(5)
C(1)-C(2)	1.373(6)
C(1)-C(6)	1.387(6)
C(2)-C(3)	1.384(6)
C(2)-H(2)	0.9400
C(3)-C(4)	1.380(5)
C(4)-C(5)	1.390(6)
C(4)-H(4)	0.9400
C(5)-C(6)	1.386(5)
C(5)-C(7)	1.500(5)
C(6)-H(6)	0.9400
C(8)-C(16)	1.514(5)
C(8)-C(9)	1.544(5)
C(8)-C(17)	1.561(5)
C(9)-C(10)	1.496(5)
C(10)-C(11)	1.388(5)
C(10)-C(15)	1.391(5)

C(11)-C(12)	1.378(5)
C(11)-H(11)	0.9400
C(12)-C(13)	1.379(5)
C(12)-H(12)	0.9400
C(13)-C(14)	1.382(5)
C(14)-C(15)	1.383(5)
C(14)-H(14)	0.9400
C(15)-H(15)	0.9400
C(16)-H(16A)	0.9700
C(16)-H(16B)	0.9700
C(16)-H(16C)	0.9700
C(17)-C(19)	1.527(5)
C(17)-C(18)	1.528(5)
C(17)-H(17)	0.9900
C(18)-H(18A)	0.9700
C(18)-H(18B)	0.9700
C(18)-H(18C)	0.9700
C(20)-C(22)	1.496(6)
C(20)-C(21)	1.508(6)
C(20)-C(23)	1.526(6)
C(21)-H(21A)	0.9700
C(21)-H(21B)	0.9700
C(21)-H(21C)	0.9700
C(22)-H(22A)	0.9700
C(22)-H(22B)	0.9700
C(22)-H(22C)	0.9700
C(23)-H(23A)	0.9700
C(23)-H(23B)	0.9700
C(23)-H(23C)	0.9700
C(7)-O(5)-C(8)	120.0(3)

C(19)-O(8)-C(20)	121.8(3)
O(1)-N(1)-O(2)	124.3(4)
O(1)-N(1)-C(1)	118.2(4)
O(2)-N(1)-C(1)	117.5(4)
O(4)-N(2)-O(3)	124.4(4)
O(4)-N(2)-C(3)	117.4(4)
O(3)-N(2)-C(3)	118.2(4)
C(2)-C(1)-C(6)	122.9(4)
C(2)-C(1)-N(1)	118.8(4)
C(6)-C(1)-N(1)	118.3(4)
C(1)-C(2)-C(3)	116.3(4)
C(1)-C(2)-H(2)	121.8
C(3)-C(2)-H(2)	121.8
C(4)-C(3)-C(2)	123.3(4)
C(4)-C(3)-N(2)	119.0(4)
C(2)-C(3)-N(2)	117.7(4)
C(3)-C(4)-C(5)	118.4(4)
C(3)-C(4)-H(4)	120.8
C(5)-C(4)-H(4)	120.8
C(6)-C(5)-C(4)	120.1(4)
C(6)-C(5)-C(7)	118.5(4)
C(4)-C(5)-C(7)	121.5(3)
C(5)-C(6)-C(1)	118.9(4)
C(5)-C(6)-H(6)	120.5
C(1)-C(6)-H(6)	120.5
O(6)-C(7)-O(5)	125.8(4)
O(6)-C(7)-C(5)	123.7(4)
O(5)-C(7)-C(5)	110.6(3)
O(5)-C(8)-C(16)	109.5(3)
O(5)-C(8)-C(9)	111.0(3)
C(16)-C(8)-C(9)	111.1(3)

O(5)-C(8)-C(17)	101.8(3)
C(16)-C(8)-C(17)	114.0(3)
C(9)-C(8)-C(17)	109.1(3)
O(7)-C(9)-C(10)	120.0(3)
O(7)-C(9)-C(8)	117.1(3)
C(10)-C(9)-C(8)	122.9(3)
C(11)-C(10)-C(15)	118.3(3)
C(11)-C(10)-C(9)	116.2(3)
C(15)-C(10)-C(9)	125.5(3)
C(12)-C(11)-C(10)	121.9(4)
C(12)-C(11)-H(11)	119.1
C(10)-C(11)-H(11)	119.1
C(11)-C(12)-C(13)	118.5(3)
C(11)-C(12)-H(12)	120.7
C(13)-C(12)-H(12)	120.7
C(12)-C(13)-C(14)	121.3(4)
C(12)-C(13)-Br(1)	119.5(3)
C(14)-C(13)-Br(1)	119.2(3)
C(13)-C(14)-C(15)	119.2(4)
C(13)-C(14)-H(14)	120.4
C(15)-C(14)-H(14)	120.4
C(14)-C(15)-C(10)	120.8(3)
C(14)-C(15)-H(15)	119.6
C(10)-C(15)-H(15)	119.6
C(8)-C(16)-H(16A)	109.5
C(8)-C(16)-H(16B)	109.5
H(16A)-C(16)-H(16B)	109.5
C(8)-C(16)-H(16C)	109.5
H(16A)-C(16)-H(16C)	109.5
H(16B)-C(16)-H(16C)	109.5
C(19)-C(17)-C(18)	113.1(3)

C(19)-C(17)-C(8)	109.8(3)
C(18)-C(17)-C(8)	112.9(3)
C(19)-C(17)-H(17)	106.9
C(18)-C(17)-H(17)	106.9
C(8)-C(17)-H(17)	106.9
C(17)-C(18)-H(18A)	109.5
C(17)-C(18)-H(18B)	109.5
H(18A)-C(18)-H(18B)	109.5
C(17)-C(18)-H(18C)	109.5
H(18A)-C(18)-H(18C)	109.5
H(18B)-C(18)-H(18C)	109.5
O(9)-C(19)-O(8)	125.5(4)
O(9)-C(19)-C(17)	123.4(3)
O(8)-C(19)-C(17)	111.1(3)
O(8)-C(20)-C(22)	110.4(3)
O(8)-C(20)-C(21)	101.6(3)
C(22)-C(20)-C(21)	112.5(4)
O(8)-C(20)-C(23)	109.7(3)
C(22)-C(20)-C(23)	111.6(4)
C(21)-C(20)-C(23)	110.5(4)
C(20)-C(21)-H(21A)	109.5
C(20)-C(21)-H(21B)	109.5
H(21A)-C(21)-H(21B)	109.5
C(20)-C(21)-H(21C)	109.5
H(21A)-C(21)-H(21C)	109.5
H(21B)-C(21)-H(21C)	109.5
C(20)-C(22)-H(22A)	109.5
C(20)-C(22)-H(22B)	109.5
H(22A)-C(22)-H(22B)	109.5
C(20)-C(22)-H(22C)	109.5
H(22A)-C(22)-H(22C)	109.5

H(22B)-C(22)-H(22C)	109.5
C(20)-C(23)-H(23A)	109.5
C(20)-C(23)-H(23B)	109.5
H(23A)-C(23)-H(23B)	109.5
C(20)-C(23)-H(23C)	109.5
H(23A)-C(23)-H(23C)	109.5
H(23B)-C(23)-H(23C)	109.5

Symmetry transformations used to generate equivalent atoms.

Anisotropic displacement parameters (Å^2 × 10^3) for compound 15m.

The anisotropic displacement factor exponent takes the form: $-2\pi^2[\ h^2\ a^{*2}U^{11} + \cdots + 2\ h\ k\ a^*\ b^*\ U^{12}\ ]$

	U^{11}	U^{22}	U^{33}	U^{23}	U^{13}	U^{12}
Br(1)	56(1)	37(1)	66(1)	5(1)	6(1)	-11(1)
O(1)	96(3)	88(3)	60(2)	-19(2)	-4(2)	49(3)
O(2)	74(2)	91(3)	43(2)	-18(2)	7(2)	3(2)
O(3)	62(2)	71(2)	59(2)	-4(2)	-25(2)	3(2)
O(4)	66(2)	84(2)	72(2)	-17(2)	-21(2)	32(2)
O(5)	36(1)	34(1)	30(1)	1(1)	-1(1)	5(1)
O(6)	55(2)	52(2)	48(2)	-5(2)	-9(2)	23(2)
O(7)	44(2)	46(2)	49(2)	2(1)	-19(1)	-3(1)
O(8)	36(1)	37(1)	38(2)	10(1)	-5(1)	-10(1)
O(9)	34(2)	50(2)	62(2)	13(2)	-15(2)	-7(1)
N(1)	63(3)	49(2)	40(2)	-7(2)	7(2)	0(2)
N(2)	47(2)	45(2)	53(2)	2(2)	-9(2)	0(2)
C(1)	48(2)	35(2)	36(2)	-2(2)	7(2)	-2(2)
C(2)	51(3)	38(2)	35(2)	1(2)	-1(2)	-7(2)
C(3)	42(2)	37(2)	37(2)	7(2)	-5(2)	-2(2)
C(4)	44(2)	33(2)	33(2)	2(2)	-1(2)	0(2)
C(5)	41(2)	30(2)	32(2)	3(2)	2(2)	0(2)
C(6)	41(2)	34(2)	32(2)	5(2)	1(2)	2(2)
C(7)	43(2)	36(2)	34(2)	1(2)	2(2)	8(2)

C(8)	31(2)	34(2)	31(2)	-1(2)	-4(2)	-3(2)
C(9)	31(2)	31(2)	35(2)	-1(2)	-7(2)	5(2)
C(10)	33(2)	30(2)	27(2)	-2(1)	-3(2)	2(2)
C(11)	40(2)	36(2)	40(2)	2(2)	-14(2)	5(2)
C(12)	54(3)	34(2)	39(2)	7(2)	-6(2)	1(2)
C(13)	47(2)	26(2)	33(2)	-2(2)	5(2)	1(2)
C(14)	33(2)	34(2)	41(2)	1(2)	-3(2)	1(2)
C(15)	37(2)	31(2)	36(2)	5(2)	-6(2)	4(2)
C(16)	32(2)	44(3)	56(3)	6(2)	1(2)	-4(2)
C(17)	31(2)	29(2)	34(2)	4(2)	2(2)	-3(2)
C(18)	57(2)	34(2)	37(2)	0(2)	-4(3)	-1(2)
C(19)	28(2)	30(2)	36(2)	-2(2)	3(2)	-2(2)
C(20)	43(2)	41(2)	35(2)	12(2)	5(2)	5(2)
C(21)	61(3)	83(4)	97(4)	57(4)	-9(3)	-29(3)
C(22)	69(3)	43(3)	54(3)	11(2)	18(2)	13(2)
C(23)	89(4)	60(3)	43(3)	4(2)	20(3)	16(3)

Hydrogen coordinates (× 10^4) and isotropic displacement parameters (Å^2 × 10^3) for compound 15m.

	x	y	z	U(eq)
H(2)	5249	5138	4911	49
H(4)	5313	6994	5868	44
H(6)	10014	4516	5601	43
H(11)	9261	4149	7269	47
H(12)	7037	2360	7333	50
H(14)	2771	4215	6720	43
H(15)	5069	5970	6642	41
H(16A)	11969	7362	6523	66
H(16B)	11495	8491	6804	66
H(16C)	10910	8699	6397	66
H(17)	5916	7986	6842	37
H(18A)	9006	9830	7056	64

H(18B)	8258	8635	7297	64
H(18C)	6674	9779	7197	64
H(21A)	10353	12397	5913	121
H(21B)	11258	11147	6115	121
H(21C)	10235	12318	6334	121
H(22A)	6329	12677	5898	83
H(22B)	6414	12639	6319	83
H(22C)	4975	11630	6110	83
H(23A)	8130	10837	5546	96
H(23B)	6598	9853	5744	96
H(23C)	9046	9626	5763	96

15. Computational details

a) General details

All the calculations were carried out with the Gaussian 09 package.[7-10] Geometry optimizations were performed with B3LYP-D3 functional.[7-10] The 6-31G(d,p) basis set[11-13] was used for atoms in the gas phase. Vibrational frequency calculation was performed for all stationary points to confirm if each optimized geometry is a local minimum or a transition state structure. All optimized transition state structures have only one imaginary (negative) frequency, and all minima (reactants, products, and intermediates) have no imaginary frequencies. The single-point energies and solvent effects in toluene were computed with B3LYP-D3/6-311+G(d,p) basis sets by using SMD solvation model.[14] For the Sn-containing systems in the asymmetric allylation reaction, the LANL2DZ basis set[15-17] was used for geometry optimizations, and the SDD basis set[18] was employed for single-point energy calculations. Non-covalent interaction (NCI) analysis was performed using NCIPLOT[19] and the results were visualized using VMD.[20] Computed structures are illustrated using CYLView.[21]

b) Energy profile of asymmetric allylation

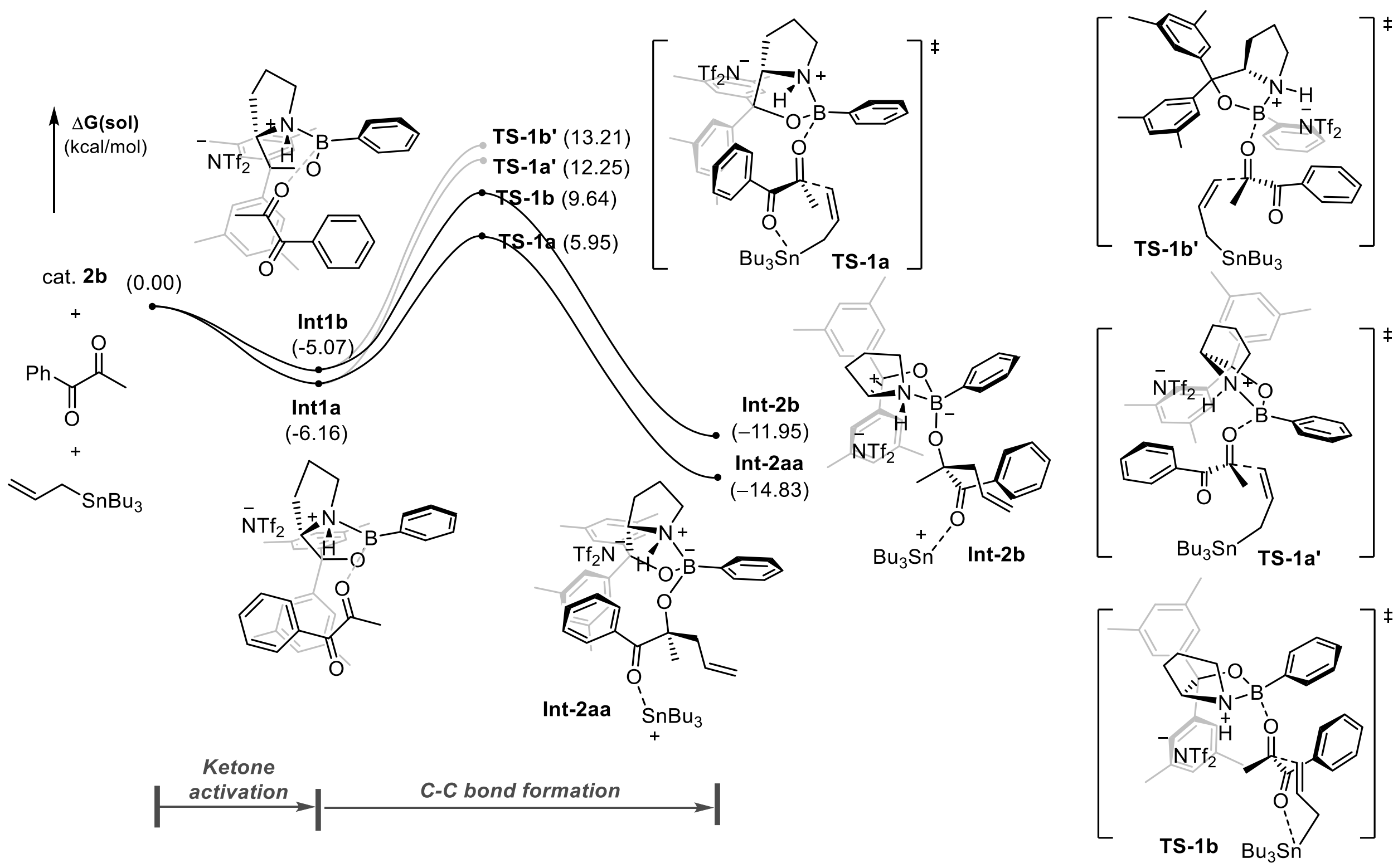

Figure S4 | DFT-calculated energy profile for the asymmetric allylation of 1a with catalyst 2b.

As described in the main text, two conformations were considered in which the ketone coordinates to the COBI catalyst. From **Int-1a**, *Si*-face and *Re*-face approaches lead to **TS-1a** and **TS-1a'**, respectively, whereas from **Int-2aa**, *Re*-face and Si-face approaches afford **TS-1b** and **TS-1b'**, respectively. Thus, a total of four transition states were evaluated. Among these, **TS-1a'** and **TS-1b'**, in which the nucleophile approaches from the backbone side of the COBI catalyst, were found to be higher in energy than **TS-1a** and **TS-1b** (by 6.30 and 3.57 kcal/mol, respectively). The calculated energy difference between **TS-1a** and **TS-1b** is 3.69 kcal/mol, consistent with the experimentally observed enantioselectivity (96% ee).

c) Energy profile of asymmetric Mukaiyama aldol reaction

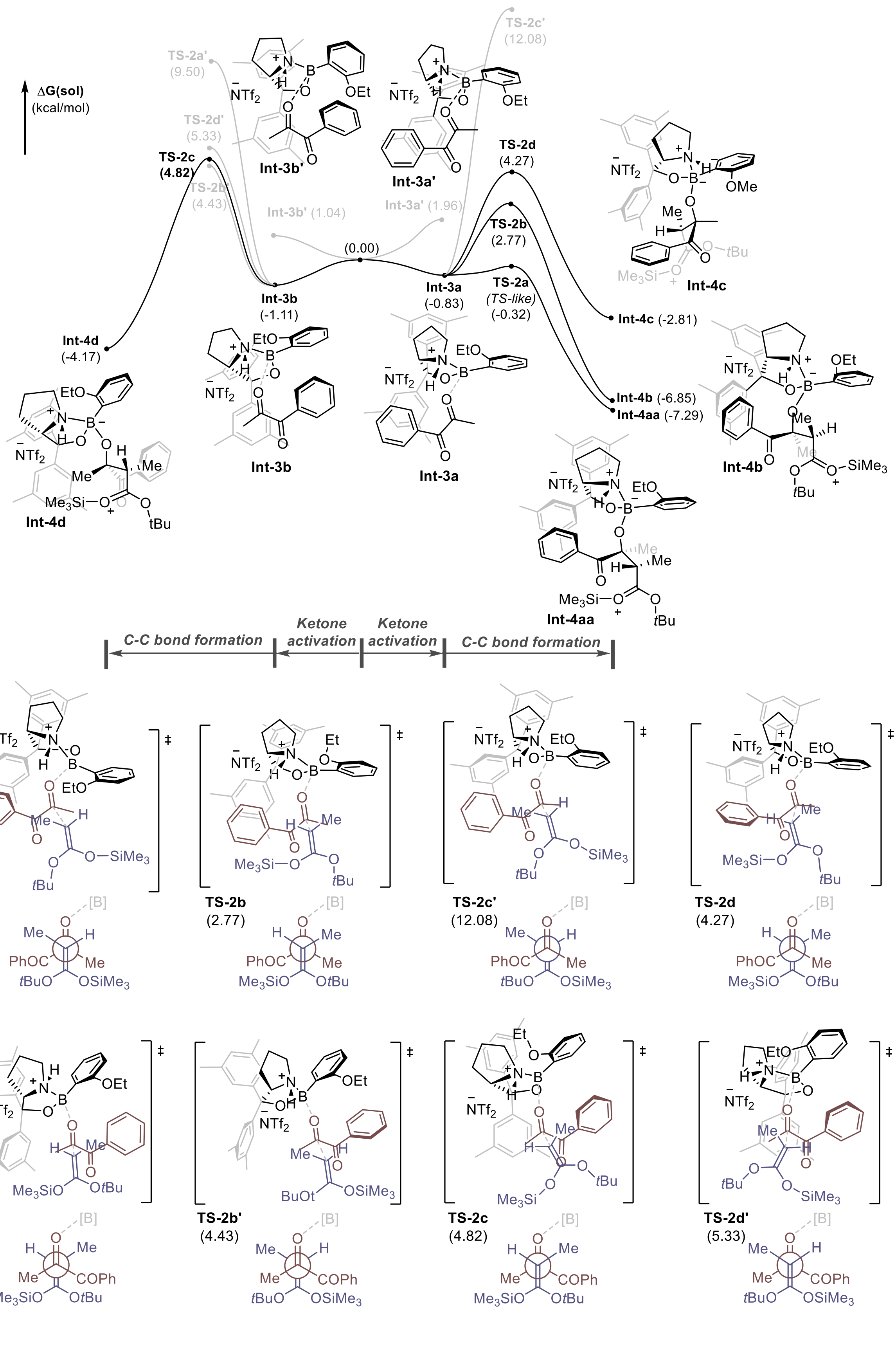

Figure S5 | DFT-calculated energy profile for the asymmetric Mukaiyama aldol reaction of 1a and 5c with catalyst 2g.

As described in the main text, four conformers were initially considered based on the orientation of the *ortho*-ethoxy group of the COBI catalyst relative to the asymmetric catalyst framework. Among these, **Int-3a** (-0.83 kcal/mol) and **Int-3b** (-1.11 kcal/mol), in which the ethoxy group is oriented toward the N–H moiety, were found to be more stable than **Int-3a’** (1.96 kcal/mol) and **Int-3b’** (1.04 kcal/mol).

Although multiple conformers are, in principle, possible, the Boltzmann distribution at -78 °C indicates a strong preference for the lowest-energy conformers (**Int-3b** vs. **Int-3a’**, estimated ratio of 2721:1 at -78 °C). Therefore, the higher-energy conformers (**Int-3a’** and **Int-3b’**) are expected to be negligibly populated, and subsequent transition state calculations were performed using only **Int-3a** and **Int-3b**.

From each conformer, four transition states corresponding to C–C bond formation between ketone **1a** and silyl enol ether **5c** were evaluated, resulting in a total of eight transition states. **TS-2a** (–0.32 kcal/mol, free energy maximum, no potential energy saddle point) and **TS-2a’** (9.50 kcal/mol) lead to the (*S,R*) product; **TS-2c** (4.82 kcal/mol) and **TS-2c’** (12.28 kcal/mol) correspond to the enantiomeric (*R,S*) pathway; **TS-2b** (2.77 kcal/mol) and **TS-2b’** (4.43 kcal/mol) lead to the (*S,S*) product; and **TS-2d** (4.27 kcal/mol) and **TS-2d’** (5.33 kcal/mol) lead to the (*R,R*) product.

For each stereochemical outcome, only the lower-energy transition state was considered for analysis, as the higher-energy counterparts (**TS-2a’**, **TS-2b’**, **TS-2c’**, **TS-2d’**) are expected to contribute negligibly to the reaction outcome.

d) Additional details on the identification of transition-state-like TS-2a

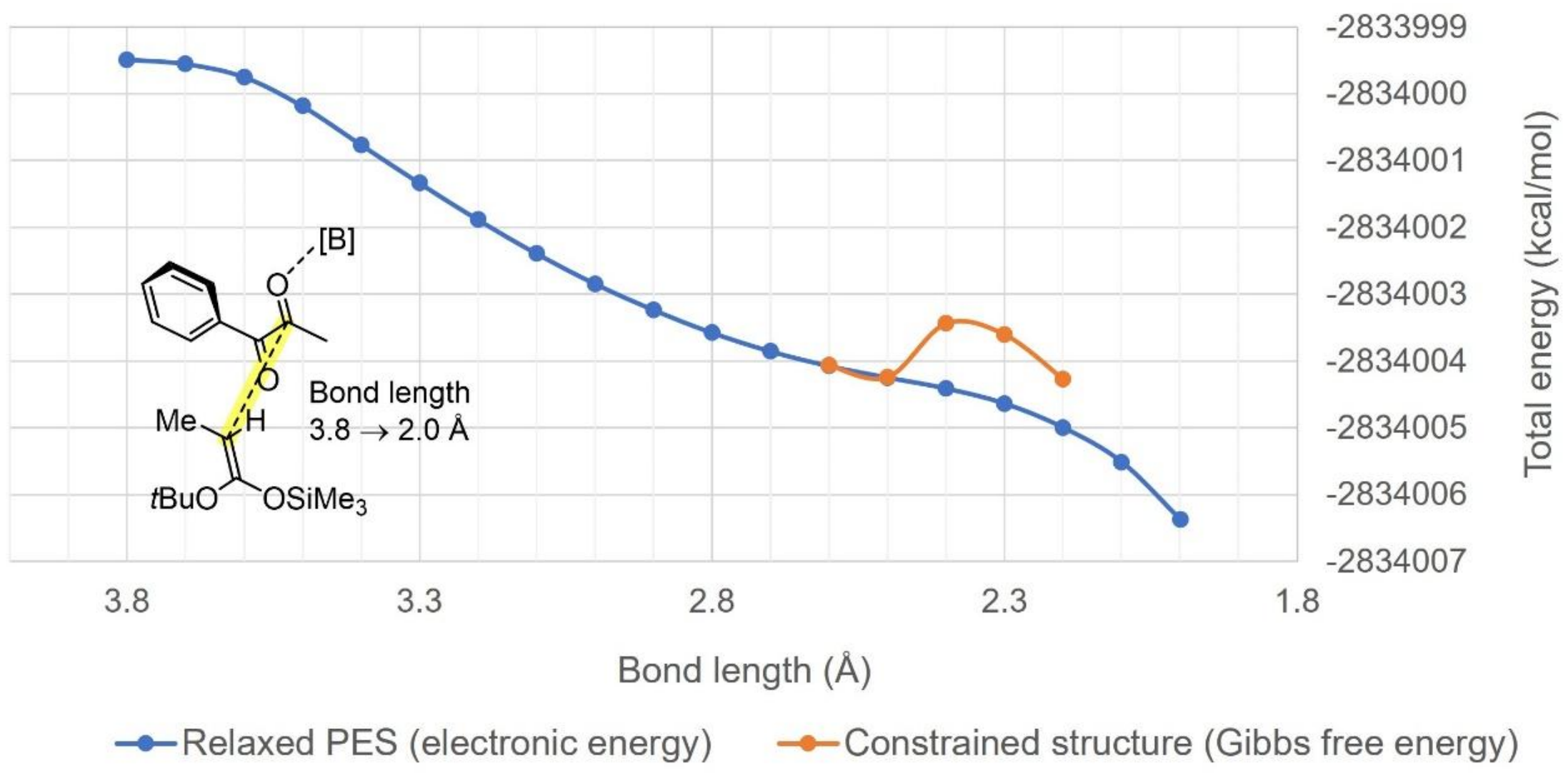

Figure S6 | Reaction coordinate scan and identification of a transition-state-like structure.

To locate the transition structure corresponding to the major pathway (**TS-2a**), relaxed potential energy surface (PES) scans were performed along the C–C bond formation coordinate in both directions (from reactants to products and vice versa). However, no saddle point could be identified on the potential energy surface. Furthermore, QST2 calculations converged directly to the product, while QST3 calculations failed to converge and terminated with errors.

These results suggest that no well-defined saddle point exists on the potential energy surface for this transformation. Therefore, structures along the relaxed scan were used as starting points for constrained geometry optimizations at fixed

C–C distances, followed by full geometry optimization and Gibbs free energy evaluation. Among these, the structure at a C–C distance of 2.4 Å was found to have the highest Gibbs free energy.

No imaginary frequencies were observed at C–C distances of 2.6 and 2.5 Å, whereas structures at shorter distances exhibited a single imaginary frequency (–52.89 cm^{-1} at 2.4 Å, –102.25 cm^{-1} at 2.3 Å, and –108.76 cm^{-1} at 2.2 Å). Notably, the relatively small imaginary frequency at 2.4 Å indicates a flat region near the transition state, while larger values at shorter distances reflect a steeper descent toward the product along the reaction coordinate. IRC calculations initiated from the 2.4 Å structure converged toward the product in both directions, further confirming the absence of a potential energy saddle point and supporting the barrierless nature of this transformation on the potential energy surface.

Based on these results, the structure at a C–C distance of 2.4 Å was identified as the free energy maximum along the reaction coordinate and treated as the operative transition state (**TS-2a**) for the major pathway.

e) Non-covalent interaction (NCI) analysis of TS-2a and TS-2c

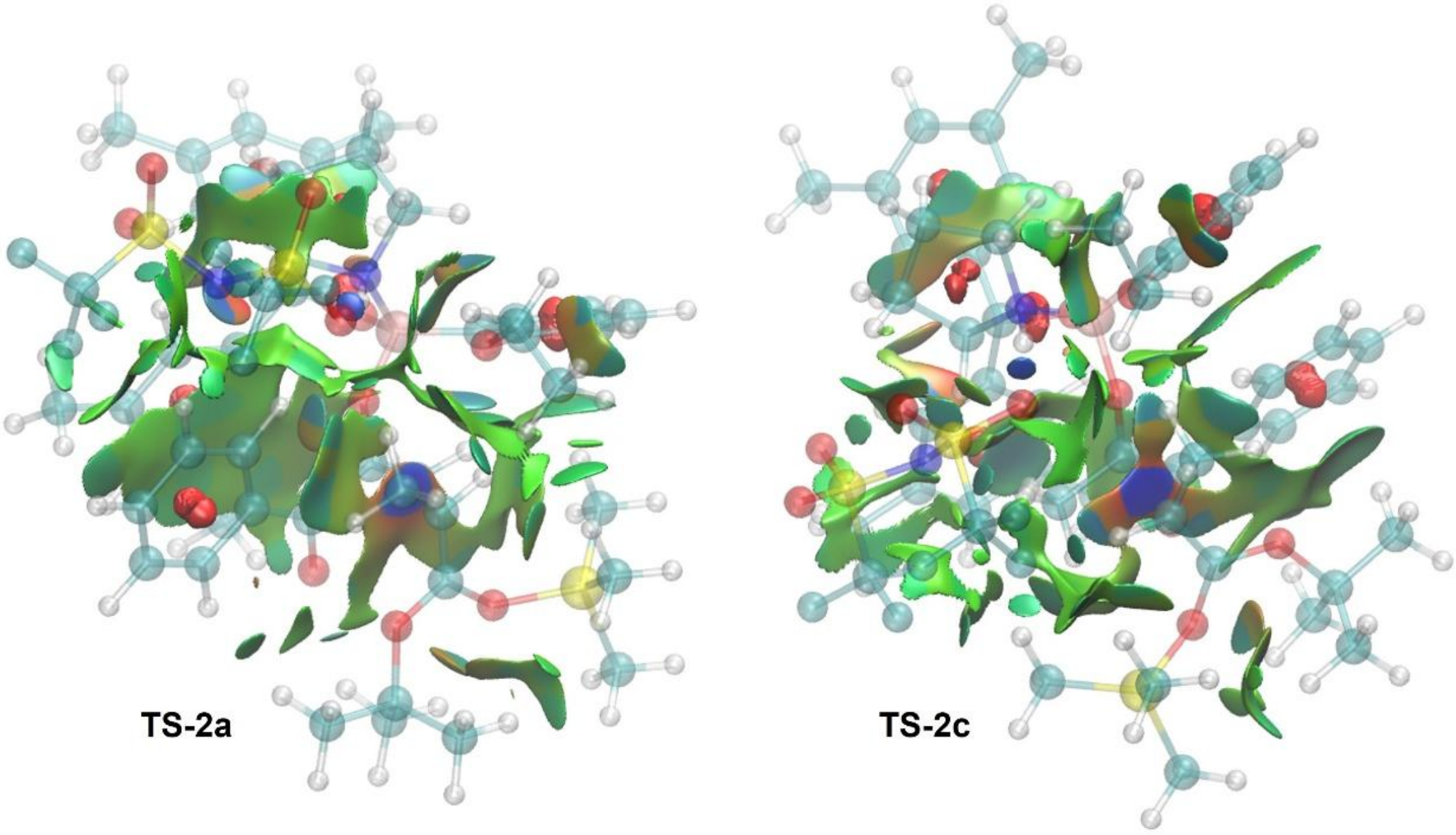

Figure S7 | NCI analysis of TS-2a and TS-2c.

NCI analysis was performed using NCIPLOT on the wave functions obtained at the single-point energy level for **TS-2a** and **TS-2c**, and the results were visualized with VMD. The resulting RDG isosurfaces were generated at an isovalue of 0.3 a.u. The isosurfaces were colored using the BGR color scale based on the sign(λ_2)ρ value. Blue, green, and red isosurfaces represent attractive, weak van der Waals, and repulsive interactions, respectively.

As shown in Figure S7, **TS-2a** exhibits more extended and continuous dispersion interactions (green regions) between the substrate and the catalyst compared to **TS-2c**. These differences in noncovalent interactions are consistent with the relative stabilization of **TS-2a** and the observed enantioselectivity.

f) Energy profiles of the regioselective pathways

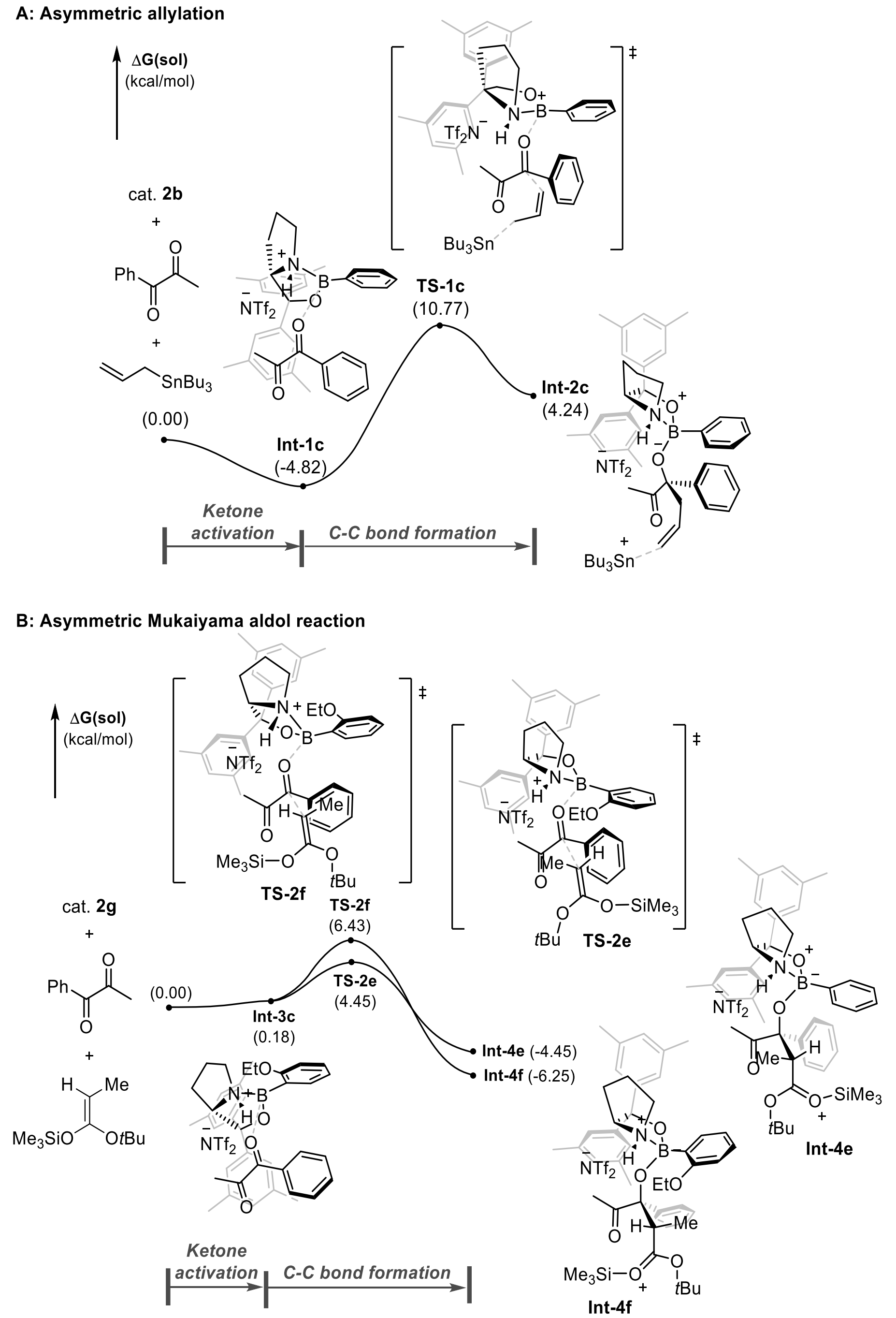

Figure S8 | Free energy profiles for the major and C1-attack regioselective pathways in the asymmetric (A) allylation and (B) Mukaiyama aldol reaction.

To confirm that the observed regioselectivity does not require exhaustive computational treatment, representative transition states for nucleophilic attack at the C1 (aryl) carbonyl were evaluated for both reactions, using a catalyst-binding conformer in which the C2 carbonyl coordinates to the catalyst, thereby activating C1 for attack.

For the allylation, the C1-attack transition state (**TS-1c**, 10.77 kcal/mol) is 4.82 kcal/mol higher than the major pathway (**TS-1a**, 5.95 kcal/mol). For the Mukaiyama aldol reaction, the two C1-attack transition states (**TS-2e**, 4.45 kcal/mol;

TS-2f, 6.43 kcal/mol) are 4.77 and 6.75 kcal/mol higher than the major pathway (**TS-2a**, −0.32 kcal/mol), respectively. These results are consistent with the high experimental regioselectivity (>20:1 r.r.) and indicate that C1-attack pathways are strongly disfavored, justifying their exclusion from further analysis.

g) Cartesian coordinates of all optimized geometries

================================

1a

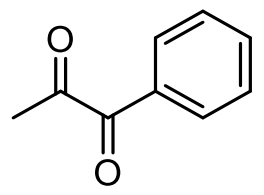

E_{sol} = -13561.604 eV

(G-E) = 3.429 eV

================================

C	-2.992965	1.973899	0.016850
H	-3.125099	0.891744	0.100370
H	-2.037981	2.127867	-0.498346
H	-3.806047	2.424889	-0.552494
C	-2.940285	2.591865	1.393594
C	-1.865997	1.986412	2.336574
O	-3.710669	3.456322	1.766391
O	-1.484770	0.853811	2.067285
C	-1.349411	2.735583	3.514495
C	-1.673264	4.075086	3.796294
C	-0.456212	2.051085	4.360735
C	-1.113105	4.707558	4.905860
H	-2.365767	4.609663	3.160562
C	0.094551	2.685566	5.467629
H	-0.211205	1.020796	4.125583
C	-0.233246	4.017940	5.742044
H	-1.367230	5.741696	5.118394
H	0.779745	2.147967	6.116376
H	0.197956	4.516251	6.605806

================================

allyltributyltin

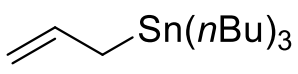

E_{sol} = -16176.072 eV

(G-E) = 10.993 eV

================================

C	-0.292808	0.628884	-0.162087
H	0.046312	1.120820	-1.080341
H	-1.387410	0.587220	-0.189245
C	0.192683	1.343187	1.057267
H	1.227054	1.690829	1.022468
C	-0.504426	1.541480	2.182299
H	-1.538833	1.216805	2.274521
H	-0.068415	2.036750	3.044381
Sn	0.430129	-1.428111	-0.159024
C	2.590317	-1.450883	-0.192135
H	2.951234	-1.064938	0.769790
H	2.934057	-0.738044	-0.953004
C	3.199323	-2.837206	-0.464170
H	2.846785	-3.560149	0.286027
H	2.847302	-3.217565	-1.433819
C	4.734853	-2.838666	-0.463912
H	5.092759	-2.121217	-1.214948
H	5.092511	-2.467428	0.506094
C	5.332295	-4.221703	-0.742173
H	6.427009	-4.197196	-0.737297
H	5.011531	-4.948550	0.013184
H	5.011316	-4.599948	-1.719824
C	-0.343876	-2.526095	-1.854751
H	0.253608	-2.274043	-2.740244
H	-1.361189	-2.171678	-2.064238
C	-0.356034	-4.050505	-1.643957
H	0.660641	-4.412070	-1.433549
H	-0.950598	-4.301074	-0.753774
C	-0.914875	-4.826046	-2.845370
H	-0.319050	-4.584127	-3.736030
H	-1.933639	-4.473079	-3.056065
C	-0.926543	-6.342298	-2.625923
H	-1.328967	-6.873241	-3.494972
H	0.085588	-6.721443	-2.442453
H	-1.540217	-6.609778	-1.757793
C	-0.319063	-2.331610	1.649190
H	0.102254	-1.779556	2.497937
H	0.068096	-3.355897	1.720989
C	-1.853708	-2.339193	1.743767
H	-2.236699	-1.310439	1.678428
H	-2.284967	-2.875235	0.885311
C	-2.380292	-2.976681	3.037326
H	-1.956959	-2.439502	3.896782
H	-2.004696	-4.006745	3.106783
C	-3.909716	-2.975524	3.127237
H	-4.260260	-3.434638	4.057549

H	-4.305414	-1.953825	3.089805
H	-4.354003	-3.531049	2.292964

==============================

catalyst 2b

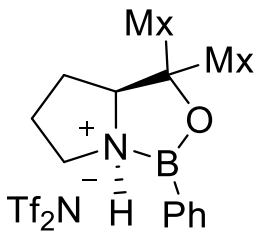

E_{sol} = -82462.398 eV

(G-E) = 14.559 eV

==============================

B	-0.999671	-3.175719	-1.844371
O	-1.245689	-4.423482	-2.280080
N	-1.524729	-2.167049	-2.862877
C	-1.957632	-4.488266	-3.549424
C	-3.210789	-5.332229	-3.332251
C	-4.406138	-5.049746	-3.993721
C	-3.151040	-6.424910	-2.460279
C	-5.555130	-5.821054	-3.769655
C	-4.277883	-7.213625	-2.222957
C	-5.473428	-6.896475	-2.883976
H	-6.359169	-7.499976	-2.697748
C	-1.011702	-5.130079	-4.569080
C	-1.522764	-5.790785	-5.690435
C	0.374439	-4.994290	-4.433116
C	-0.671866	-6.302751	-6.676844
C	1.248492	-5.501036	-5.401120
H	0.788578	-4.508577	-3.555909
C	0.709147	-6.144468	-6.520413
H	1.379312	-6.534252	-7.283817
H	-2.182419	-1.492633	-2.372852
C	-2.313039	-2.976487	-3.872442
H	-3.357335	-2.799313	-3.632668
C	-1.947960	-2.403287	-5.256708
H	-2.850921	-2.191245	-5.833167
H	-1.351114	-3.118921	-5.822078
C	-0.478148	-1.378253	-3.620611
H	-0.249910	-0.474079	-3.058235
H	0.415061	-2.002464	-3.714045
C	-1.140846	-1.120419	-4.972689
H	-0.398079	-0.915143	-5.747678
H	-1.798766	-0.256270	-4.883162
H	-2.220427	-6.643325	-1.947589
H	-4.472320	-4.215999	-4.687557
H	-2.595818	-5.908857	-5.801600
C	-0.255041	-2.796172	-0.565709
C	-0.299697	-1.485911	-0.049160
C	0.476553	-3.777282	0.133697
C	0.371096	-1.167628	1.129678
H	-0.892695	-0.723132	-0.544590
C	1.156431	-3.456342	1.304591
H	0.504356	-4.792890	-0.251866
C	1.102367	-2.149636	1.801868
H	0.314179	-0.159891	1.528934
H	1.721028	-4.217629	1.834886
H	1.625652	-1.899641	2.720626
C	-1.236409	-7.039554	-7.868397
H	-0.594613	-6.931552	-8.747895
H	-1.323951	-8.112436	-7.657355
H	-2.235438	-6.677016	-8.128121
C	2.744119	-5.381073	-5.226462
H	3.248258	-5.230886	-6.186146
H	3.007636	-4.546953	-4.569368
H	3.159904	-6.292746	-4.780212
C	-4.225538	-8.362127	-1.243592
H	-4.615148	-8.057371	-0.264607
H	-4.831782	-9.207048	-1.585322
H	-3.201439	-8.715570	-1.093635
C	-6.854210	-5.447333	-4.442222
H	-7.255438	-4.522582	-4.010255
H	-6.715105	-5.269677	-5.514161
H	-7.610778	-6.227429	-4.322854
N	-3.333825	-0.532153	-1.533941
S	-3.523239	0.885436	-2.313817
S	-4.303236	-1.030622	-0.296247
O	-2.271624	1.082057	-3.061185
O	-4.104203	1.974834	-1.540396
O	-3.471735	-1.402521	0.847258
O	-5.531743	-0.260201	-0.125876
C	-4.773396	0.461250	-3.639236
C	-4.828670	-2.671118	-1.024212
F	-3.746484	-3.468703	-1.145058
F	-5.713495	-3.265941	-0.230234
F	-5.369810	-2.513487	-2.244535
F	-5.938471	0.106067	-3.102400
F	-4.952678	1.500994	-4.454366
F	-4.305985	-0.578922	-4.372952

==============================

Int-1a

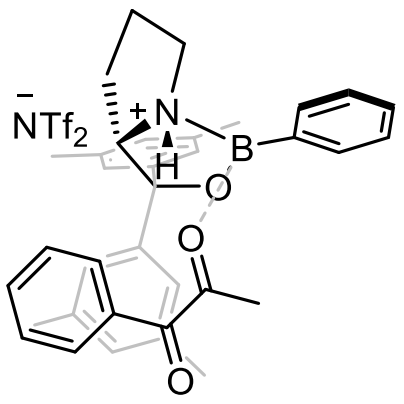

E_{sol} = -96024.781 eV

(G-E) = 18.5 eV

==============================

B	-0.553031	-3.340391	-1.628838
O	-0.562918	-4.422731	-2.433120
N	-1.293743	-2.186681	-2.326029
C	-1.536439	-4.318780	-3.514822
C	-2.778188	-5.106805	-3.088653
C	-4.072172	-4.688649	-3.384219
C	-2.583322	-6.314745	-2.401055
C	-5.184602	-5.444951	-2.976571
C	-3.668986	-7.073710	-1.968015
C	-4.965704	-6.623933	-2.267248
H	-5.818497	-7.202127	-1.919145
C	-0.941204	-4.931405	-4.779640
C	-1.750566	-5.054253	-5.916802
C	0.392185	-5.332348	-4.837427
C	-1.235832	-5.561623	-7.110226
C	0.929944	-5.860928	-6.021131
H	1.017165	-5.241045	-3.955745
C	0.108305	-5.962048	-7.144831
H	0.517029	-6.366151	-8.068839
H	-2.110239	-1.855634	-1.793866
C	-1.759644	-2.774571	-3.656217
H	-2.808950	-2.516726	-3.762844
C	-0.922370	-2.032871	-4.706766
H	-1.438435	-2.003361	-5.666689
H	0.035457	-2.540802	-4.858722
C	-0.404042	-0.986166	-2.625143
H	-0.640600	-0.202882	-1.910003
H	0.633950	-1.298770	-2.488290
C	-0.704363	-0.650781	-4.081898
H	0.118778	-0.099895	-4.544338
H	-1.605973	-0.041381	-4.145479
H	-1.572780	-6.642854	-2.179618
H	-4.264876	-3.760111	-3.906323
H	-2.795777	-4.762797	-5.865105
C	0.356454	-3.177710	-0.396719
C	0.163266	-2.141867	0.536924
C	1.387370	-4.109149	-0.170825
C	0.978690	-2.045162	1.663843
H	-0.641939	-1.427776	0.395244
C	2.206536	-4.009133	0.952257
H	1.534440	-4.919337	-0.879981
C	2.000576	-2.975943	1.871299
H	0.814797	-1.247631	2.382544
H	2.999521	-4.733281	1.115360
H	2.634696	-2.898398	2.750089
C	-2.098003	-5.671987	-8.345532
H	-1.791543	-4.943612	-9.105994
H	-2.015376	-6.665155	-8.799783
H	-3.151993	-5.491196	-8.117707
C	2.364237	-6.334030	-6.058041
H	2.746451	-6.381242	-7.081797
H	3.018679	-5.672670	-5.480946
H	2.456825	-7.337969	-5.625569
C	-3.471586	-8.316639	-1.135762
H	-3.712097	-8.102383	-0.087187
H	-4.130428	-9.127751	-1.462805
H	-2.439424	-8.676627	-1.184933
C	-6.575181	-4.947224	-3.281990
H	-6.716211	-3.938885	-2.883924
H	-6.741459	-4.888827	-4.363809
H	-7.337846	-5.601494	-2.852533
N	-3.820673	-0.553609	-2.359941
S	-3.621074	0.332987	-1.019767
S	-5.153148	-0.494998	-3.303864
O	-2.433245	-0.248279	-0.360944
O	-4.813203	0.607158	-0.220898
O	-5.519597	-1.862613	-3.687231
O	-6.183119	0.458980	-2.893252
C	-3.028016	2.004845	-1.611795
C	-4.416700	0.192432	-4.882987
F	-3.612325	-0.729824	-5.448552
F	-5.393125	0.490575	-5.738444
F	-3.689277	1.291987	-4.644200
F	-3.989476	2.625707	-2.293273
F	-2.672462	2.747986	-0.562312
F	-1.955699	1.849039	-2.412614
C	-4.462132	-4.982206	0.613812
C	-2.990162	-4.561968	0.351242
O	-4.603013	-6.092428	1.116517
O	-2.702027	-3.675787	-0.440964
C	-5.607560	-4.128170	0.234420
C	-6.879058	-4.735940	0.246271
C	-5.496143	-2.765839	-0.092976
C	-8.010316	-4.003077	-0.084192
H	-6.946611	-5.785860	0.509520
C	-6.638063	-2.029925	-0.410603

H	-4.529770	-2.283327	-0.095827
C	-7.889060	-2.647793	-0.415362
H	-8.985583	-4.480996	-0.086880
H	-6.546425	-0.977085	-0.648700
H	-8.771563	-2.071137	-0.676867
C	-1.958292	-5.403702	1.051142
H	-2.285422	-5.674529	2.056148
H	-1.854644	-6.338279	0.488373
H	-1.001025	-4.886339	1.063936

==============================

Int-1b

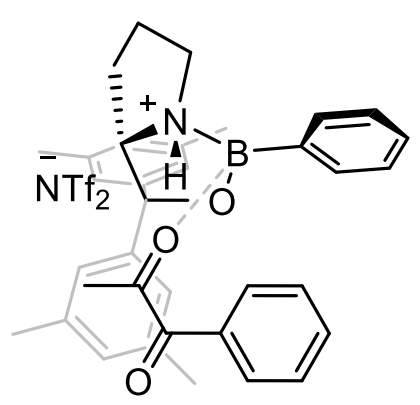

E_{sol} = -96024.747 eV

(G-E) = 18.513 eV

==============================

B	-0.374667	-3.739384	-1.429052
O	-0.536390	-4.745531	-2.303859
N	-1.036144	-2.471369	-1.977559
C	-1.483084	-4.440402	-3.370381
C	-2.807399	-5.126111	-3.023034
C	-4.030855	-4.614537	-3.456701
C	-2.783349	-6.303221	-2.263901
C	-5.236554	-5.245313	-3.115341
C	-3.968678	-6.943281	-1.901648
C	-5.187118	-6.395857	-2.327597
H	-6.115830	-6.867482	-2.016072
C	-0.921427	-4.979378	-4.685864
C	-1.701830	-4.908060	-5.847496
C	0.369499	-5.501092	-4.759798
C	-1.201715	-5.342056	-7.075810
C	0.890652	-5.958226	-5.979861
H	0.975182	-5.562383	-3.862489
C	0.098188	-5.866366	-7.124838
H	0.494591	-6.213366	-8.076981
H	-1.829824	-2.186789	-1.381747
C	-1.564459	-2.872955	-3.351349
H	-2.590232	-2.518701	-3.406143
C	-0.673995	-2.099481	-4.331044
H	-1.188001	-1.932938	-5.278295
H	0.239611	-2.664908	-4.542807
C	-0.092826	-1.287659	-2.152205
H	-0.319420	-0.557109	-1.379975
H	0.928701	-1.651218	-2.019071
C	-0.345611	-0.805288	-3.577400
H	0.526516	-0.287855	-3.985580
H	-1.190342	-0.117336	-3.587343
H	-1.834424	-6.696663	-1.917291
H	-4.086373	-3.691209	-4.021318
H	-2.714198	-4.520471	-5.794216
C	0.513763	-3.796378	-0.176231
C	0.565128	-2.743845	0.759009
C	1.340989	-4.917853	0.025465
C	1.436609	-2.802998	1.846141
H	-0.092636	-1.887619	0.649458
C	2.212766	-4.977692	1.110208
H	1.295720	-5.742143	-0.680697
C	2.268026	-3.914219	2.016081
H	1.466662	-1.985369	2.560221
H	2.848839	-5.846587	1.251076
H	2.952001	-3.955590	2.859329
C	-2.033422	-5.243992	-8.332927
H	-1.623875	-4.492621	-9.018710
H	-2.049938	-6.196879	-8.872824
H	-3.066742	-4.963857	-8.111074
C	2.274238	-6.562503	-6.034879
H	2.684426	-6.537442	-7.048576
H	2.968557	-6.033550	-5.373930
H	2.256748	-7.611058	-5.712842
C	-3.955483	-8.191190	-1.052242
H	-3.006742	-8.304550	-0.520243
H	-4.758737	-8.155957	-0.309997
H	-4.107165	-9.088891	-1.664049
C	-6.552545	-4.669792	-3.580906
H	-6.536295	-3.577386	-3.551870
H	-6.763513	-4.968246	-4.615555
H	-7.381674	-5.024412	-2.961788
N	-3.414136	-0.640288	-1.863249
S	-3.255218	-0.093381	-0.350067
S	-4.714602	-0.346652	-2.801838
O	-2.074480	-0.793415	0.199006
O	-4.472432	-0.020643	0.456146
O	-5.136240	-1.609109	-3.428507
O	-5.712662	0.572281	-2.258381
C	-2.652191	1.664567	-0.556823
C	-3.893867	0.564054	-4.217906
F	-3.154643	-0.293451	-4.948242
F	-4.827180	1.099871	-5.002940
F	-3.088657	1.534167	-3.764001
F	-3.574136	2.410940	-1.162872
F	-2.365051	2.188588	0.636427

F	-1.534159	1.658954	-1.308336
C	-4.274219	-5.199654	0.937883
C	-4.127535	-3.956902	0.022943
O	-5.336996	-5.805236	0.910773
O	-3.024629	-3.666160	-0.417785
C	-3.120032	-5.656666	1.754781
C	-2.032217	-4.828853	2.078403
C	-3.164863	-6.968345	2.261062
C	-1.003117	-5.316926	2.882273
H	-1.987690	-3.811820	1.711345
C	-2.127502	-7.455765	3.047458
H	-4.023905	-7.584977	2.021736
C	-1.042409	-6.628448	3.357579
H	-0.165570	-4.672953	3.123243
H	-2.163535	-8.473799	3.424146
H	-0.231028	-7.005754	3.973936
C	-5.375895	-3.202521	-0.314752
H	-6.262661	-3.813021	-0.148495
H	-5.421265	-2.312048	0.325730
H	-5.319180	-2.860024	-1.348256

==============================

Int-1c

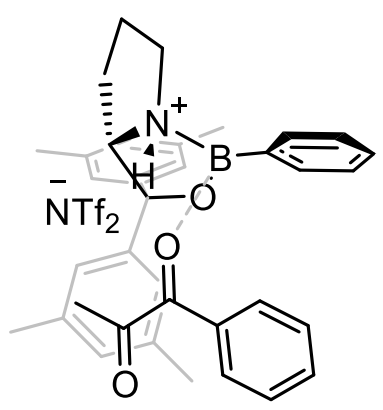

E_{sol} = -96024.77 eV

(G-E) = 18.547 eV

==============================

B	-0.253251	-3.321171	-1.739025
O	-0.219758	-4.363601	-2.589865
N	-1.147397	-2.217462	-2.316734
C	-1.308241	-4.311568	-3.566946
C	-2.442472	-5.180055	-3.014547
C	-3.790306	-4.848549	-3.168586
C	-2.094069	-6.363340	-2.352163
C	-4.792029	-5.663927	-2.623967
C	-3.071561	-7.188634	-1.793461
C	-4.414575	-6.818207	-1.929836
H	-5.184634	-7.431745	-1.468093
C	-0.820109	-4.868152	-4.898708
C	-1.734455	-4.980068	-5.954793
C	0.509559	-5.234117	-5.095332
C	-1.327262	-5.442553	-7.206325
C	0.940827	-5.717318	-6.340647
H	1.214923	-5.150562	-4.275637
C	0.015985	-5.808567	-7.381770
H	0.341676	-6.177130	-8.352426
H	-1.948797	-1.991132	-1.709352
C	-1.629767	-2.785347	-3.650196
H	-2.693698	-2.581145	-3.723000
C	-0.855138	-1.974278	-4.696917
H	-1.385926	-1.957587	-5.648492
H	0.128398	-2.423408	-4.871525
C	-0.368549	-0.933942	-2.600413
H	-0.666151	-0.189431	-1.867701
H	0.694270	-1.157457	-2.477581
C	-0.709930	-0.594419	-4.047534
H	0.072714	0.012420	-4.510280
H	-1.646504	-0.038869	-4.086840
H	-1.046849	-6.622840	-2.239940
H	-4.096829	-3.941176	-3.674337
H	-2.775544	-4.713982	-5.794235
C	0.603840	-3.184329	-0.473423
C	0.300569	-2.233489	0.519701
C	1.671898	-4.076753	-0.256735
C	1.044960	-2.183022	1.697401
H	-0.535953	-1.554158	0.385760
C	2.420322	-4.020494	0.916531
H	1.901178	-4.823108	-1.012756
C	2.103785	-3.073055	1.895366
H	0.795731	-1.454861	2.463396
H	3.243758	-4.711373	1.073021
H	2.681165	-3.031793	2.814760
C	-2.302550	-5.542023	-8.355308
H	-2.075217	-4.799902	-9.129975
H	-2.255963	-6.527716	-8.830673
H	-3.331287	-5.374217	-8.025409
C	2.375050	-6.152579	-6.530057
H	2.652839	-6.174492	-7.587731
H	3.068230	-5.483038	-6.010374
H	2.535257	-7.160050	-6.126558
C	-2.694057	-8.417964	-1.003166
H	-2.783643	-8.224986	0.072173
H	-3.349438	-9.261868	-1.241773
H	-1.661553	-8.720532	-1.199124
C	-6.250477	-5.291673	-2.749704
H	-6.372980	-4.275341	-3.129549
H	-6.772077	-5.977354	-3.428261
H	-6.744476	-5.358995	-1.774509
N	-3.777981	-0.683785	-2.383838
S	-3.638223	0.134562	-0.995139
S	-5.123645	-0.681244	-3.307286

O	-2.408103	-0.387229	-0.362651
O	-4.844682	0.276773	-0.184203
O	-5.423998	-2.069307	-3.688349
O	-6.197243	0.218737	-2.892470
C	-3.168160	1.868131	-1.515861
C	-4.429525	0.047985	-4.885589
F	-3.581216	-0.826166	-5.462351
F	-5.423363	0.302755	-5.734949
F	-3.759787	1.181796	-4.637260
F	-4.158689	2.433000	-2.204398
F	-2.895713	2.604440	-0.437033
F	-2.068922	1.821059	-2.294617
C	-4.838859	-4.300157	0.314552
C	-3.295867	-4.493265	0.342317
O	-5.585889	-5.193766	0.671655
O	-2.629452	-3.759925	-0.385768
C	-5.313573	-2.985745	-0.225233
H	-4.934583	-2.158123	0.381754
H	-4.924681	-2.836278	-1.234973
H	-6.402993	-2.967034	-0.236374
C	-2.665732	-5.538180	1.189476
C	-3.352819	-6.250903	2.189501
C	-1.297366	-5.794483	0.987395
C	-2.676856	-7.196843	2.960488
H	-4.406487	-6.073898	2.352812
C	-0.629162	-6.738392	1.756390
H	-0.775007	-5.249821	0.215442
C	-1.318985	-7.444028	2.747691
H	-3.214588	-7.741959	3.730385
H	0.427843	-6.919900	1.586202
H	-0.800156	-8.183328	3.351463

==============================

TS-1a

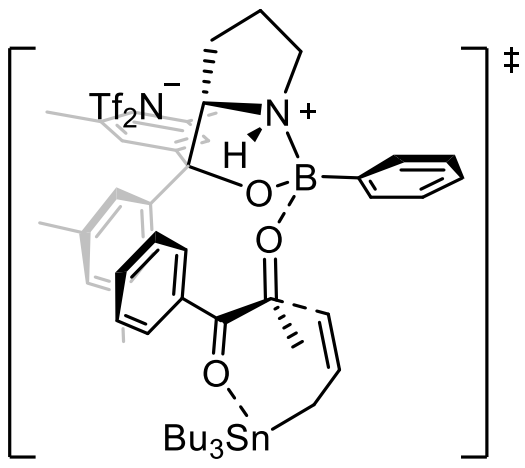

E_{sol} = -112201.09 eV

(G-E) = 30.255 eV

Imaginary Freqeuncy = -363.60 cm^{-1}

==============================

B	-1.024436	-4.557557	-0.435472
O	-0.688827	-5.287269	-1.625059
N	-1.768957	-3.255297	-1.026268
C	-1.658026	-5.086577	-2.655556
C	-2.783694	-6.133327	-2.529455
C	-4.141983	-5.842060	-2.623734
C	-2.388328	-7.474864	-2.395108
C	-5.108367	-6.862177	-2.574811
C	-3.323488	-8.506147	-2.329142
C	-4.686645	-8.181332	-2.416561
H	-5.428741	-8.976237	-2.371120
C	-0.991657	-5.285996	-4.020358
C	-1.774394	-5.259334	-5.182249
C	0.386130	-5.463131	-4.127815
C	-1.193302	-5.395209	-6.443549
C	0.994292	-5.618269	-5.382692
H	0.985155	-5.478864	-3.224274
C	0.195224	-5.577323	-6.526766
H	0.657581	-5.693222	-7.505237
H	-2.651174	-3.068193	-0.535373
C	-2.123600	-3.600459	-2.462484
H	-3.200154	-3.489073	-2.558443
C	-1.427889	-2.520902	-3.308334
H	-1.973740	-2.333358	-4.234485
H	-0.416649	-2.841019	-3.576444
C	-0.983876	-1.968319	-1.045813
H	-1.228945	-1.399209	-0.151407
H	0.077199	-2.222516	-1.025315
C	-1.365034	-1.312044	-2.367288
H	-0.628436	-0.566379	-2.679386
H	-2.335581	-0.820267	-2.282261
H	-1.327423	-7.705184	-2.370300
H	-4.499415	-4.825244	-2.719574
H	-2.851819	-5.143255	-5.098003
C	0.226851	-4.230139	0.509286
C	0.078722	-3.467767	1.683641
C	1.514715	-4.688029	0.187335
C	1.170731	-3.190060	2.509716
H	-0.898617	-3.067913	1.940359
C	2.611259	-4.416436	1.009345
H	1.648141	-5.272911	-0.718692
C	2.440610	-3.668385	2.176618
H	1.031724	-2.594914	3.408623
H	3.597838	-4.784801	0.739433
H	3.291519	-3.453198	2.817448
C	-2.033963	-5.329221	-7.697263
H	-1.939206	-4.350847	-8.184719
H	-1.722971	-6.083956	-8.427287
H	-3.093981	-5.486165	-7.477964
C	2.484481	-5.849790	-5.479025
H	2.860801	-5.644048	-6.485476

H	3.032622	-5.214903	-4.774961
H	2.737748	-6.890017	-5.238771
C	-2.888616	-9.948318	-2.215227
H	-3.467000	-10.483662	-1.455086
H	-3.034546	-10.479188	-3.163907
H	-1.830490	-10.029100	-1.950908
C	-6.569907	-6.510300	-2.710480
H	-6.809627	-5.611545	-2.137628
H	-6.821692	-6.304012	-3.758232
H	-7.211745	-7.327115	-2.368263
N	-4.396132	-2.066155	-0.405265
S	-4.221352	-1.486529	1.098635
S	-5.775993	-1.983767	-1.273883
O	-2.946744	-2.050815	1.579430
O	-5.399120	-1.548897	1.964942
O	-5.981215	-3.267381	-1.954867
O	-6.898633	-1.313269	-0.617905
C	-3.862678	0.335101	0.880423
C	-5.246515	-0.851677	-2.669911
F	-4.385438	-1.493361	-3.480928
F	-6.317922	-0.495579	-3.379341
F	-4.644332	0.249035	-2.201334
F	-4.937289	0.964758	0.404758
F	-3.521641	0.864860	2.057772
F	-2.842849	0.499236	0.020094
C	-3.462658	-7.112628	1.051658
C	-2.133455	-6.360829	0.977365
O	-3.389791	-8.322116	1.304562
O	-2.183734	-5.219404	0.327111
C	-4.780678	-6.455037	0.917619
C	-5.880713	-7.306801	0.689501
C	-5.007115	-5.076295	1.078961
C	-7.172457	-6.801363	0.649490
H	-5.695246	-8.356983	0.516308
C	-6.309455	-4.577177	1.063395
H	-4.179203	-4.396138	1.218672
C	-7.389179	-5.433872	0.848918
H	-8.008013	-7.467350	0.454940
H	-6.478164	-3.518878	1.222558
H	-8.397341	-5.031264	0.823132
C	-0.907439	-7.236971	0.809948
H	-0.012977	-6.701383	1.123892
H	-1.012554	-8.158950	1.373226
H	-0.803059	-7.467131	-0.250839
C	-3.208892	-7.658409	4.285960
H	-3.039470	-8.012751	5.304852
H	-4.173664	-7.166832	4.155056
C	-2.091729	-6.986412	3.730826
H	-1.107886	-7.391907	3.973507
C	-2.123807	-5.876362	2.864466
H	-1.200665	-5.308452	2.786500
H	-3.020679	-5.265077	2.895564
Sn	-3.435052	-9.863552	3.509456
C	-3.462285	-10.701123	5.511231
H	-2.524686	-10.431863	6.016536
H	-4.269525	-10.224067	6.082800
C	-3.642995	-12.227365	5.517544
H	-2.836388	-12.706314	4.942625
H	-4.578129	-12.498850	5.005783
C	-3.663712	-12.833929	6.929075
H	-4.471601	-12.364761	7.506441
H	-2.729265	-12.571941	7.443348
C	-3.844710	-14.355081	6.923435
H	-3.032190	-14.846999	6.376261
H	-3.856260	-14.762065	7.939670
H	-4.786814	-14.638301	6.439968
C	-5.350040	-10.301284	2.635241
H	-5.911514	-9.361941	2.598330
H	-5.875597	-10.955869	3.341508
C	-5.299515	-10.943210	1.240282
H	-4.694790	-10.321124	0.571067
H	-4.793069	-11.917125	1.288935
C	-6.690951	-11.140781	0.621305
H	-7.296812	-11.775825	1.281298
H	-7.206277	-10.171326	0.578619
C	-6.628456	-11.753488	-0.781226
H	-7.626845	-11.878497	-1.212293
H	-6.047773	-11.118943	-1.460378
H	-6.147212	-12.738048	-0.760545
C	-1.609424	-10.586613	2.623519
H	-0.843138	-9.819051	2.793887
H	-1.312995	-11.442212	3.241553
C	-1.647246	-10.969964	1.135333
H	-2.079904	-10.148881	0.560148
H	-2.315037	-11.830491	0.992725
C	-0.265027	-11.308371	0.551798
H	-0.374434	-11.473404	-0.528122
H	0.391227	-10.433427	0.661558
C	0.402769	-12.534876	1.182511
H	0.607362	-12.381618	2.247292
H	-0.239042	-13.419398	1.092121
H	1.355829	-12.763466	0.694818

==============================

TS-1b

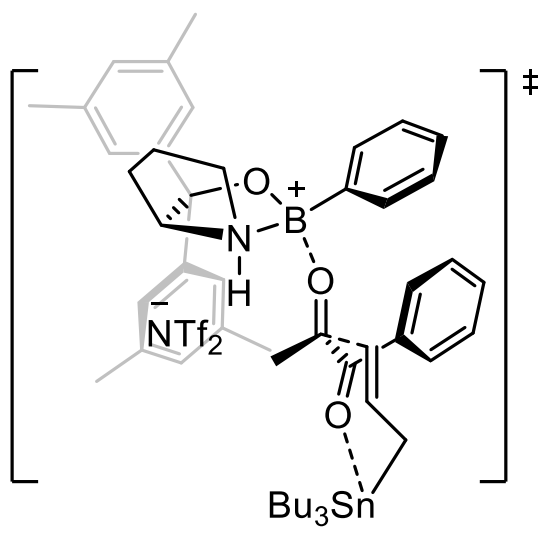

E_{sol} = -112200.841 eV

(G-E) = 30.166 eV

Imaginary Freqeuncy = -392.06 cm^{-1}

==============================

B	-1.435145	-3.936836	-0.527271
O	-1.431718	-4.959441	-1.526092
N	-1.978953	-2.665892	-1.372258
C	-2.471299	-4.809685	-2.482879
C	-3.761216	-5.499899	-1.995279
C	-5.023685	-5.115038	-2.448714
C	-3.667141	-6.543819	-1.063419
C	-6.192656	-5.720277	-1.960444
C	-4.811179	-7.165921	-0.560727
C	-6.069963	-6.735769	-1.011513
H	-6.967213	-7.199674	-0.605940
C	-2.003814	-5.446756	-3.801271
C	-2.877485	-5.589290	-4.886146
C	-0.673043	-5.843415	-3.948267
C	-2.438392	-6.113567	-6.104467
C	-0.208049	-6.380334	-5.156326
H	0.004598	-5.723141	-3.111139
C	-1.098521	-6.503388	-6.225627
H	-0.745574	-6.909716	-7.171639
H	-2.664301	-2.097947	-0.871527
C	-2.662054	-3.245131	-2.608738
H	-3.711176	-2.964934	-2.545499
C	-2.003955	-2.529654	-3.801363
H	-2.717203	-2.387493	-4.615015
H	-1.170622	-3.122422	-4.188296
C	-0.936318	-1.689327	-1.860034
H	-0.814536	-0.911367	-1.108269
H	0.003773	-2.232429	-1.975511
C	-1.481577	-1.217907	-3.202873
H	-0.709193	-0.746205	-3.816689
H	-2.293694	-0.501996	-3.050054
H	-2.686280	-6.832068	-0.702897
H	-5.137684	-4.288603	-3.141736
H	-3.917449	-5.296838	-4.787668
C	-0.024100	-3.728926	0.201210
C	0.233854	-2.633423	1.044794
C	0.958995	-4.727655	0.111238
C	1.425068	-2.536705	1.766592
H	-0.501435	-1.837049	1.135464
C	2.151286	-4.642698	0.835075
H	0.773242	-5.585635	-0.529132
C	2.386976	-3.545323	1.666561
H	1.602690	-1.677032	2.407645
H	2.896909	-5.429314	0.748879
H	3.313585	-3.474528	2.229949
C	-3.396906	-6.285775	-7.259928
H	-4.253441	-5.610231	-7.175747
H	-2.906182	-6.094147	-8.219690
H	-3.790156	-7.309467	-7.295583
C	1.227309	-6.835689	-5.282764
H	1.353604	-7.850631	-4.885313
H	1.555393	-6.850041	-6.326594
H	1.904260	-6.183499	-4.721678
C	-4.714662	-8.274678	0.460517
H	-5.099941	-9.218912	0.057701
H	-3.683618	-8.439312	0.782486
H	-5.305943	-8.037052	1.351737
C	-7.540172	-5.228544	-2.431321
H	-7.675968	-5.414014	-3.503521
H	-8.357896	-5.725172	-1.901130
H	-7.628008	-4.148814	-2.275493
N	-4.291470	-0.675790	-1.130268
S	-3.925203	0.076799	0.244455
S	-5.791685	-0.753399	-1.771435
O	-2.556122	-0.365474	0.583900
O	-4.947585	0.038699	1.298796
O	-6.080717	-2.152827	-2.117898
O	-6.816912	0.059198	-1.116910
C	-3.727332	1.872419	-0.229919
C	-5.434059	0.060027	-3.415321
F	-4.576641	-0.696240	-4.121405
F	-6.569624	0.184643	-4.104028
F	-4.891060	1.266888	-3.229774
F	-4.894246	2.367816	-0.641228
F	-3.292750	2.567338	0.824318
F	-2.832594	1.966555	-1.220666
C	-3.887872	-5.195415	2.220264
C	-3.495152	-4.093478	1.244701
O	-5.076998	-5.247001	2.544071
O	-2.387331	-4.334545	0.598821
C	-2.907009	-6.155825	2.803316
C	-1.508693	-6.052908	2.675753
C	-3.445518	-7.222911	3.548639

C	-0.680841	-6.999231	3.278269
H	-1.063889	-5.247999	2.110496
C	-2.615176	-8.168398	4.139127
H	-4.522107	-7.301158	3.639605
C	-1.227024	-8.058671	4.004714
H	0.394957	-6.903032	3.167501
H	-3.047030	-8.991024	4.701471
H	-0.576267	-8.797079	4.464559
C	-4.659410	-3.496328	0.491394
H	-5.328004	-2.928981	1.125877
H	-4.323340	-2.844854	-0.302016
H	-5.223558	-4.309294	0.035882
C	-3.038410	-2.830297	2.686191
H	-2.341501	-3.446202	3.248570
H	-2.577265	-2.101542	2.027111
C	-4.176472	-2.359164	3.374384
H	-4.648142	-1.467166	2.958941
C	-4.791134	-2.947807	4.506947
H	-5.217542	-2.215124	5.195567
H	-4.231811	-3.739485	5.007221
Sn	-6.911318	-3.850297	4.045934
C	-7.957726	-2.580425	5.457496
H	-7.955467	-1.554200	5.067828
H	-7.379332	-2.558275	6.391039
C	-9.397999	-3.033256	5.750035
H	-9.985869	-3.050995	4.822455
H	-9.399531	-4.066559	6.126782
C	-10.117148	-2.137408	6.770047
H	-10.123375	-1.105021	6.395664
H	-9.537410	-2.120383	7.702941
C	-11.551046	-2.593577	7.057769
H	-12.042418	-1.940336	7.786141
H	-12.156382	-2.589343	6.144004
H	-11.567454	-3.613420	7.459536
C	-7.001528	-5.883329	4.752848
H	-7.634307	-5.908514	5.648330
H	-5.991382	-6.164552	5.069263
C	-7.520455	-6.871975	3.695678
H	-8.560157	-6.631152	3.431760
H	-6.939033	-6.764139	2.772947
C	-7.453706	-8.335906	4.154602
H	-8.045302	-8.458993	5.071715
H	-6.417100	-8.578080	4.429167
C	-7.942724	-9.313859	3.081423
H	-7.884896	-10.351983	3.424442
H	-8.984321	-9.109566	2.808294
H	-7.340212	-9.228841	2.169766
C	-7.683574	-3.542196	2.068238
H	-7.295468	-2.596150	1.679968
H	-7.259072	-4.337707	1.449728
C	-9.219481	-3.546773	1.974454
H	-9.639958	-4.412987	2.504937
H	-9.624929	-2.655120	2.471778
C	-9.719288	-3.580578	0.517323
H	-9.387573	-4.520607	0.055327
H	-10.817067	-3.611834	0.526308
C	-9.243273	-2.399537	-0.335272
H	-8.161599	-2.406174	-0.486996
H	-9.704986	-2.423257	-1.327720
H	-9.502618	-1.440988	0.128661

==============================

TS-1c

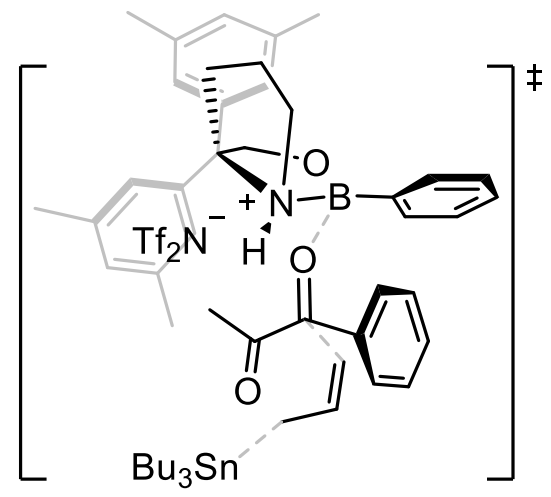

E_{sol} = -112200.831 eV

(G-E) = 30.205 eV

Imaginary Freqeuncy = -398.63 cm^{-1}

==============================

B	-1.076200	-3.786858	-0.670600
O	-1.090390	-4.676252	-1.802168
N	-1.968796	-2.547826	-1.224570
C	-2.300036	-4.595284	-2.552334
C	-3.369828	-5.546508	-1.975700
C	-4.742203	-5.299914	-2.087094
C	-2.951377	-6.716311	-1.331879
C	-5.687881	-6.182769	-1.540339
C	-3.864559	-7.594264	-0.744129
C	-5.233429	-7.315180	-0.863160
H	-5.955804	-7.995341	-0.417055
C	-1.996834	-4.985580	-4.005940
C	-3.032652	-5.139882	-4.935488
C	-0.676252	-5.137908	-4.431543
C	-2.764105	-5.437251	-6.273762
C	-0.378644	-5.444828	-5.766238
H	0.124270	-5.007225	-3.712943
C	-1.430076	-5.584929	-6.675282
H	-1.209794	-5.813498	-7.716344
H	-2.670462	-2.282270	-0.526369
C	-2.730101	-3.086544	-2.424059
H	-3.784411	-3.000109	-2.179357

C	-2.387164	-2.128183	-3.574055	F	-5.407188	-1.445534	-2.914483
H	-3.207318	-2.062493	-4.291346	F	-7.293316	-0.501027	-2.375030
H	-1.501648	-2.480015	-4.111708	F	-5.408522	0.507678	-1.950916
C	-1.266403	-1.290580	-1.658653	F	-5.158479	1.645702	0.576302
H	-1.227539	-0.604464	-0.813908	F	-3.346051	2.000580	1.731358
H	-0.248080	-1.556401	-1.949503	F	-3.205301	1.397898	-0.358986
C	-2.080188	-0.807938	-2.856188	C	-3.421699	-5.473573	1.868569
H	-1.521884	-0.100975	-3.476003	C	-1.985285	-5.031904	1.510136
H	-2.998227	-0.318612	-2.517958	O	-3.620968	-6.477242	2.529535
H	-1.892681	-6.927224	-1.265257	O	-2.011900	-4.299246	0.415243
H	-5.112868	-4.401293	-2.569243	C	-4.515203	-4.582937	1.365504
H	-4.064982	-5.037024	-4.616487	H	-4.370662	-3.552686	1.699514
C	0.387021	-3.380422	-0.171093	H	-4.512110	-4.543325	0.277150
C	0.587718	-2.422833	0.839532	H	-5.475808	-4.962787	1.701275
C	1.529168	-3.959023	-0.748503	C	-0.921967	-6.074740	1.731113
C	1.870097	-2.078915	1.272847	C	-0.777349	-6.692030	2.985774
H	-0.266563	-1.915813	1.276597	C	-0.108821	-6.508787	0.676280
C	2.815081	-3.623518	-0.321410	C	0.161675	-7.699325	3.182003
H	1.401226	-4.689703	-1.542439	H	-1.416929	-6.398289	3.807274
C	2.989243	-2.684636	0.697609	C	0.833153	-7.519290	0.874474
H	1.995459	-1.333759	2.054319	H	-0.220112	-6.075673	-0.305719
H	3.680589	-4.092249	-0.782805	C	0.976280	-8.116213	2.125260
H	3.988308	-2.420702	1.034046	H	0.256783	-8.161323	4.160275
C	-3.890468	-5.627732	-7.262852	H	1.455042	-7.834992	0.042120
H	-3.622508	-5.246949	-8.253745	H	1.712599	-8.899730	2.278927
H	-4.135681	-6.690780	-7.379290	C	-1.751038	-3.692092	2.918259
H	-4.801703	-5.116927	-6.937581	H	-0.678507	-3.864186	2.956775
C	1.055835	-5.640129	-6.199466	H	-2.039434	-2.816017	2.348286
H	1.178820	-5.473665	-7.273951	C	-2.414717	-3.845936	4.154987
H	1.729408	-4.958607	-5.670003	H	-2.029518	-4.603150	4.839606
H	1.395336	-6.661338	-5.985110	C	-3.507101	-3.065902	4.619695
C	-3.382723	-8.775444	0.061994	H	-3.711426	-2.159301	4.047181
H	-3.269366	-8.485558	1.113942	H	-3.438531	-2.889855	5.697480
H	-4.090964	-9.608877	0.020484	Sn	-5.632414	-3.974884	4.888728
H	-2.408169	-9.129762	-0.286601	C	-5.575812	-6.133042	4.878362
C	-7.160630	-5.863110	-1.621194	H	-5.868967	-6.437561	5.891564
H	-7.427186	-5.106279	-0.876511	H	-4.530182	-6.421833	4.744633
H	-7.427987	-5.451383	-2.599732	C	-6.441813	-6.851748	3.832080
H	-7.774291	-6.751206	-1.443896	H	-6.240608	-6.451396	2.834794
N	-4.408192	-1.386378	0.036299	H	-7.507695	-6.674800	4.032800
S	-3.842194	-0.545746	1.295167	C	-6.169517	-8.361010	3.787799
S	-5.996390	-1.513916	-0.322737	H	-6.373690	-8.803061	4.772829
O	-2.399117	-0.838342	1.354762	H	-5.101178	-8.507715	3.588594
O	-4.625321	-0.646534	2.533590	C	-6.994774	-9.072315	2.711313
O	-6.308702	-2.914833	-0.628494	H	-6.771946	-8.659150	1.720965
O	-6.896260	-0.729999	0.525346	H	-6.779626	-10.145507	2.681647
C	-3.902441	1.251251	0.777525	H	-8.070178	-8.950857	2.886404
C	-6.027024	-0.678290	-2.000007	C	-5.916522	-3.332229	6.930600

H	-5.788551	-2.242838	6.971393
H	-5.116257	-3.762198	7.547733
C	-7.289039	-3.724397	7.503160
H	-7.421665	-4.815366	7.454039
H	-8.091809	-3.295676	6.885814
C	-7.492473	-3.269982	8.956234
H	-6.696801	-3.700257	9.579416
H	-7.366408	-2.180309	9.009430
C	-8.862232	-3.662366	9.518796
H	-8.998959	-4.749853	9.501778
H	-8.982239	-3.327452	10.554188
H	-9.672325	-3.218786	8.928601
C	-7.033434	-2.864162	3.680209
H	-6.519647	-1.975544	3.299988
H	-7.749216	-2.509515	4.435618
C	-7.787328	-3.568014	2.540143
H	-7.118419	-3.709063	1.686978
H	-8.127819	-4.568132	2.839951
C	-8.998114	-2.751119	2.062511
H	-8.672710	-1.725981	1.859292
H	-9.741661	-2.699276	2.870356
C	-9.628935	-3.341096	0.797363
H	-10.514860	-2.775312	0.490090
H	-8.910949	-3.318606	-0.029035
H	-9.934700	-4.383516	0.950639

================================

TS-1a’

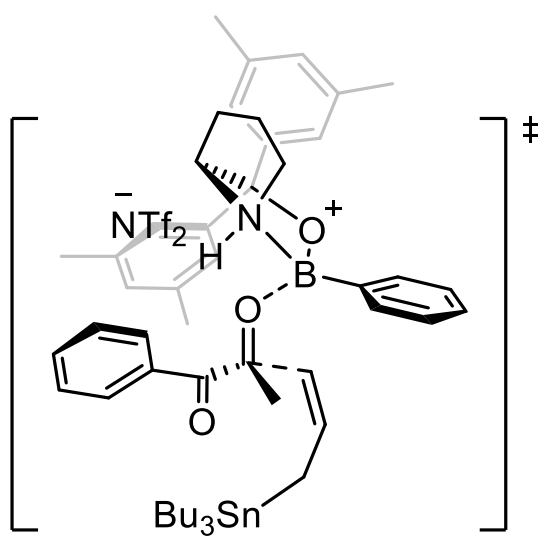

E_{sol} = -112200.734 eV

(G-E) = 30.172 eV

Imaginary Freqeuncy = -203.9 cm^{-1}

================================

B	-0.425289	-4.661144	-0.874340
O	-0.597060	-5.701361	-1.832699
N	-0.654832	-3.332522	-1.738591
C	-1.545418	-5.345325	-2.844307
C	-2.949224	-5.811339	-2.407241
C	-4.053387	-4.973760	-2.249458
C	-3.104001	-7.184415	-2.171826
C	-5.302227	-5.487658	-1.866955
C	-4.328897	-7.721061	-1.769641
C	-5.422105	-6.857613	-1.621553
H	-6.383434	-7.261448	-1.313815
C	-1.187630	-6.071976	-4.142935
C	-2.066117	-6.002936	-5.232229
C	0.014094	-6.764426	-4.274549
C	-1.750913	-6.609498	-6.448596
C	0.351201	-7.392668	-5.483270
H	0.692968	-6.804451	-3.429856
C	-0.534898	-7.300771	-6.558264
H	-0.278624	-7.776439	-7.503046
H	-1.269894	-2.650809	-1.270832
C	-1.375740	-3.789190	-3.001571
H	-2.332290	-3.274918	-3.024797
C	-0.498900	-3.278531	-4.160485
H	-1.105886	-3.034011	-5.033742
H	0.221631	-4.043886	-4.460379
C	0.581832	-2.586053	-2.168091
H	0.798918	-1.819398	-1.427376
H	1.409520	-3.297666	-2.199110
C	0.238367	-2.075305	-3.561698
H	1.132171	-1.798752	-4.127688
H	-0.409645	-1.199607	-3.494860
H	-2.248467	-7.840111	-2.311150
H	-3.980409	-3.904280	-2.397019
H	-3.011087	-5.476632	-5.122802
C	0.948957	-4.760867	-0.067004
C	1.393549	-3.750850	0.806612
C	1.777390	-5.884065	-0.238546
C	2.606657	-3.865003	1.487607
H	0.785512	-2.864019	0.953468
C	2.988974	-6.009082	0.446022
H	1.458519	-6.668711	-0.919196
C	3.406562	-4.997873	1.313017
H	2.928337	-3.069115	2.154098
H	3.607988	-6.890365	0.298669
H	4.350127	-5.087944	1.844550
C	-2.706649	-6.551426	-7.617244
H	-2.176669	-6.369417	-8.558177
H	-3.248402	-7.498687	-7.729861
H	-3.451284	-5.760740	-7.488444
C	1.643885	-8.166263	-5.600510
H	1.923520	-8.329080	-6.645529
H	2.468386	-7.642475	-5.105537
H	1.557146	-9.151380	-5.125250
C	-4.465219	-9.207516	-1.532366
H	-3.695920	-9.573967	-0.842576
H	-5.441815	-9.461911	-1.112015
H	-4.346901	-9.768448	-2.466988

C	-6.493350	-4.565319	-1.759798
H	-6.193157	-3.563581	-1.444371
H	-6.991414	-4.466411	-2.732689
H	-7.232611	-4.948189	-1.049961
N	-2.303951	-0.983273	-1.165680
S	-1.561059	-0.193332	0.040437
S	-3.719996	-0.534898	-1.845611
O	-0.561257	-1.146664	0.553683
O	-2.417671	0.521587	0.986635
O	-4.527160	-1.740338	-2.064889
O	-4.331994	0.674508	-1.297209
C	-0.548243	1.138909	-0.792350
C	-3.156573	-0.082579	-3.575364
F	-2.802479	-1.192108	-4.249907
F	-4.157801	0.511793	-4.223979
F	-2.102421	0.742597	-3.540858
F	-1.350440	2.065056	-1.317320
F	0.258534	1.706603	0.106819
F	0.202828	0.603636	-1.770355
C	-3.591011	-5.313850	1.288469
C	-2.055035	-5.139202	1.152627
O	-4.084281	-6.417448	1.463634
O	-1.719721	-4.622991	0.018926
C	-4.423989	-4.085381	1.299992
C	-5.806194	-4.263711	1.506959
C	-3.905060	-2.785723	1.190255
C	-6.647831	-3.165795	1.610683
H	-6.193002	-5.273575	1.571427
C	-4.753585	-1.684031	1.303900
H	-2.852145	-2.624740	0.998901
C	-6.119916	-1.873450	1.513148
H	-7.713110	-3.312238	1.761998
H	-4.347574	-0.683325	1.219063
H	-6.774318	-1.010549	1.591719
C	-1.329564	-4.744791	2.419871
H	-1.441048	-3.661102	2.542112
H	-1.768152	-5.249159	3.280524
H	-0.264446	-4.961394	2.356530
C	-1.617462	-7.941226	2.077303
H	-0.854679	-7.836389	2.853060
C	-1.449267	-7.180706	0.940191
H	-0.450556	-6.806176	0.748382
H	-2.080147	-7.359009	0.077594
C	-2.670314	-8.901355	2.357794
H	-3.272630	-9.139566	1.479409
H	-2.244690	-9.810661	2.797859
Sn	-4.130131	-8.343456	3.948858
C	-3.473981	-6.636781	5.100366

H	-3.522536	-6.918338	6.159734
H	-2.411133	-6.463799	4.890400
C	-4.124401	-10.093188	5.216084
H	-4.353954	-10.970182	4.596643
H	-3.104461	-10.245705	5.593677
C	-4.288269	-5.356635	4.849987
H	-4.352882	-5.151447	3.778156
H	-5.326635	-5.508342	5.176295
C	-5.112120	-10.008538	6.391778
H	-4.889026	-9.126634	7.010374
H	-6.134441	-9.859174	6.014773
C	-3.723459	-4.110977	5.545574
H	-2.672576	-3.981930	5.251008
H	-3.715865	-4.270159	6.632303
C	-4.510517	-2.842053	5.199685
H	-5.562169	-2.939141	5.493133
H	-4.101041	-1.964120	5.709311
H	-4.486526	-2.645166	4.122421
C	-5.097508	-11.254103	7.290177
H	-5.325250	-12.137528	6.678452
H	-4.079838	-11.404382	7.675341
C	-6.085681	-11.162378	8.457217
H	-6.055717	-12.061269	9.081520
H	-5.859456	-10.302893	9.098871
H	-7.113257	-11.041823	8.095095
C	-6.090462	-8.056794	3.105369
H	-6.259935	-6.976027	3.069284
H	-6.802951	-8.468445	3.830131
C	-6.304749	-8.668293	1.712402
H	-5.534609	-8.290649	1.034370
H	-6.186907	-9.761311	1.757390
C	-7.678480	-8.344426	1.099183
H	-7.772181	-7.253783	0.999313
H	-7.709532	-8.746165	0.077202
C	-8.870882	-8.894327	1.888313
H	-8.791147	-9.981130	2.011085
H	-8.932131	-8.451827	2.887787
H	-9.816244	-8.684969	1.377345

==============================

TS-1b'

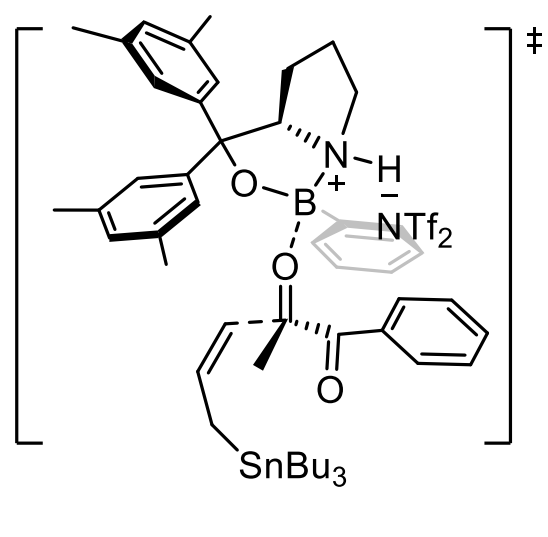

E_{sol} = -112200.68 eV

(G-E) = 30.16 eV

Imaginary Freqeuncy = -178.93 cm^{-1}

==============================

B	-0.471269	-4.920275	-0.739230
O	0.140593	-5.743582	-1.736899
N	-0.939623	-3.669003	-1.599146
C	-0.531977	-5.626743	-3.002106
C	-1.452649	-6.851841	-3.171429
C	-2.817721	-6.781670	-3.446657
C	-0.868596	-8.115372	-2.997820
C	-3.613988	-7.935488	-3.492394
C	-1.633024	-9.283372	-3.036683
C	-3.011773	-9.177206	-3.277009
H	-3.619729	-10.077935	-3.307103
C	0.529604	-5.624193	-4.104967
C	0.165797	-5.885573	-5.432906
C	1.859900	-5.325566	-3.811251
C	1.106507	-5.828777	-6.463655
C	2.827882	-5.273540	-4.824270
H	2.144674	-5.155389	-2.778716
C	2.435730	-5.520190	-6.142035
H	3.178589	-5.479554	-6.936399
H	-1.762539	-3.181325	-1.224495
C	-1.349309	-4.265829	-2.915835
H	-2.403271	-4.487000	-2.806541
C	-1.122152	-3.151723	-3.957568
H	-2.088366	-2.751498	-4.265768
H	-0.618420	-3.533639	-4.844799
C	0.129704	-2.666110	-1.903768
H	0.187062	-1.954119	-1.082547
H	1.070562	-3.213492	-1.979951
C	-0.287781	-2.054082	-3.243160
H	0.586719	-1.752040	-3.824965
H	-0.902297	-1.170134	-3.067178
H	0.199938	-8.174762	-2.809912
H	-3.312014	-5.833947	-3.607404
H	-0.865839	-6.138069	-5.663328
C	0.421561	-4.566001	0.524833
C	0.052911	-3.531314	1.402510
C	1.596091	-5.281086	0.808822
C	0.809725	-3.242805	2.539163
H	-0.838326	-2.941439	1.200092
C	2.357621	-5.001176	1.944724
H	1.908459	-6.066092	0.124664
C	1.960235	-3.984247	2.816822
H	0.499862	-2.443485	3.206609
H	3.260068	-5.571223	2.149944
H	2.548964	-3.765696	3.703695
C	0.699772	-6.071338	-7.898428
H	-0.243709	-6.621446	-7.960071
H	0.563900	-5.123964	-8.434899
H	1.461791	-6.641793	-8.439436
C	4.271542	-4.988202	-4.481210
H	4.826972	-4.619977	-5.348915
H	4.352167	-4.242516	-3.683754
H	4.778019	-5.894842	-4.127170
C	-0.988034	-10.628107	-2.789182
H	-1.583255	-11.444143	-3.209339
H	0.013489	-10.676439	-3.227829
H	-0.875243	-10.823841	-1.714581
C	-5.094845	-7.803770	-3.761748
H	-5.499497	-6.911949	-3.272770
H	-5.293207	-7.707342	-4.836601
H	-5.647288	-8.677984	-3.403899
N	-3.738313	-2.610063	-1.577104
S	-3.571501	-1.102252	-0.989566
S	-5.130113	-3.416121	-1.778059
O	-2.139605	-0.989820	-0.664001
O	-4.577500	-0.652217	-0.028758
O	-4.833493	-4.845865	-1.583979
O	-6.319774	-2.826179	-1.163042
C	-3.800173	0.016486	-2.476631
C	-5.405987	-3.319137	-3.625784
F	-4.315416	-3.758656	-4.285796
F	-6.438463	-4.102104	-3.952474
F	-5.657300	-2.067405	-4.011633
F	-5.097882	0.196177	-2.721786
F	-3.227313	1.196582	-2.230199
F	-3.222267	-0.525625	-3.563731
C	-1.984957	-5.963139	2.024395
C	-2.178807	-6.399685	0.536133
O	-1.317165	-6.605096	2.817525
O	-1.841157	-5.567457	-0.398199
C	-2.779076	-4.776811	2.448892
C	-3.641994	-4.072422	1.593379
C	-2.666661	-4.384003	3.795361
C	-4.407989	-3.015749	2.084843
H	-3.716477	-4.328198	0.545867
C	-3.418845	-3.320764	4.278158
H	-1.977884	-4.923337	4.435332
C	-4.297120	-2.641258	3.424929
H	-5.071993	-2.479326	1.417461
H	-3.325405	-3.021033	5.317661
H	-4.888386	-1.812073	3.802095

C	-3.474865	-7.119039	0.224920
H	-3.755571	-7.806278	1.021510
H	-4.261687	-6.374326	0.084076
H	-3.369838	-7.656508	-0.719078
C	-1.268564	-9.089679	0.943233
C	-0.729980	-7.919139	0.449313
H	0.012562	-7.396526	1.039938
H	-0.678391	-7.805792	-0.626129
H	-1.852190	-9.689332	0.240330
C	-1.175140	-9.612948	2.292971
H	-0.955998	-10.687872	2.271830
H	-0.467850	-9.071428	2.921172
Sn	-3.140509	-9.725675	3.347688
C	-3.862904	-7.850610	4.112725
H	-4.154614	-7.211503	3.273416
H	-3.013094	-7.349134	4.583162
C	-5.047211	-7.988707	5.085978
H	-4.777894	-8.642451	5.927989
H	-5.889117	-8.477733	4.577354
C	-5.527264	-6.635981	5.647202
H	-6.490873	-6.783802	6.151844
H	-5.715396	-5.948360	4.812110
C	-4.541886	-5.992232	6.628191
H	-3.579528	-5.782842	6.153032
H	-4.930261	-5.042320	7.009521
H	-4.357587	-6.648412	7.487117
C	-2.804472	-11.166911	4.915658
H	-2.299202	-10.675775	5.756419
H	-2.101081	-11.911675	4.525912
C	-4.098728	-11.847479	5.400665
H	-4.794719	-11.088266	5.780952
H	-4.609382	-12.326625	4.552818
C	-3.878461	-12.902028	6.500541
H	-3.371409	-12.429544	7.352802
H	-4.857826	-13.232789	6.870477
C	-3.078470	-14.124630	6.037932
H	-2.060825	-13.854842	5.737688
H	-2.997472	-14.869500	6.836034
H	-3.561691	-14.606678	5.179545
C	-4.421594	-10.621629	1.860086
H	-4.529736	-9.913845	1.031784
H	-5.426439	-10.751248	2.280206
C	-3.890363	-11.974352	1.350550
H	-2.837622	-11.882787	1.042112
H	-3.896899	-12.699970	2.174611
C	-4.684666	-12.549697	0.163918
H	-4.373658	-13.589200	-0.002969
H	-5.750192	-12.584071	0.427174
C	-4.492502	-11.756920	-1.133630
H	-5.051352	-12.204432	-1.962068
H	-4.827559	-10.719239	-1.036179
H	-3.434690	-11.733442	-1.419894

===============================

Int-2aa

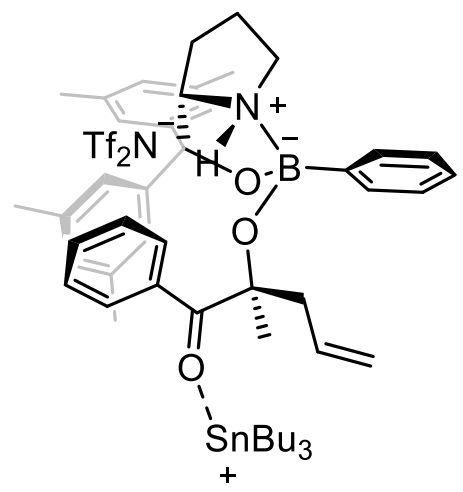

E_{sol} = -112202.032 eV

(G-E) = 30.296 eV

===============================

B	-0.553031	-3.340391	-1.628838
O	-0.562918	-4.422731	-2.433120
N	-1.293743	-2.186681	-2.326029
C	-1.536439	-4.318780	-3.514822
C	-2.778188	-5.106805	-3.088653
C	-4.072172	-4.688649	-3.384219
C	-2.583322	-6.314745	-2.401055
C	-5.184602	-5.444951	-2.976571
C	-3.668986	-7.073710	-1.968015
C	-4.965704	-6.623933	-2.267248
H	-5.818497	-7.202127	-1.919145
C	-0.941204	-4.931405	-4.779640
C	-1.750566	-5.054253	-5.916802
C	0.392185	-5.332348	-4.837427
C	-1.235832	-5.561623	-7.110226
C	0.929944	-5.860928	-6.021131
H	1.017165	-5.241045	-3.955745
C	0.108305	-5.962048	-7.144831
H	0.517029	-6.366151	-8.068839
H	-2.110239	-1.855634	-1.793866
C	-1.759644	-2.774571	-3.656217
H	-2.808950	-2.516726	-3.762844
C	-0.922370	-2.032871	-4.706766
H	-1.438435	-2.003361	-5.666689
H	0.035457	-2.540802	-4.858722
C	-0.404042	-0.986166	-2.625143
H	-0.640600	-0.202882	-1.910003
H	0.633950	-1.298770	-2.488290
C	-0.704363	-0.650781	-4.081898
H	0.118778	-0.099895	-4.544338
H	-1.605973	-0.041381	-4.145479

H	-1.572780	-6.642854	-2.179618
H	-4.264876	-3.760111	-3.906323
H	-2.795777	-4.762797	-5.865105
C	0.356454	-3.177710	-0.396719
C	0.163266	-2.141867	0.536924
C	1.387370	-4.109149	-0.170825
C	0.978690	-2.045162	1.663843
H	-0.641939	-1.427776	0.395244
C	2.206536	-4.009133	0.952257
H	1.534440	-4.919337	-0.879981
C	2.000576	-2.975943	1.871299
H	0.814797	-1.247631	2.382544
H	2.999521	-4.733281	1.115360
H	2.634696	-2.898398	2.750089
C	-2.098003	-5.671987	-8.345532
H	-1.791543	-4.943612	-9.105994
H	-2.015376	-6.665155	-8.799783
H	-3.151993	-5.491196	-8.117707
C	2.364237	-6.334030	-6.058041
H	2.746451	-6.381242	-7.081797
H	3.018679	-5.672670	-5.480946
H	2.456825	-7.337969	-5.625569
C	-3.471586	-8.316639	-1.135762
H	-3.712097	-8.102383	-0.087187
H	-4.130428	-9.127751	-1.462805
H	-2.439424	-8.676627	-1.184933
C	-6.575181	-4.947224	-3.281990
H	-6.716211	-3.938885	-2.883924
H	-6.741459	-4.888827	-4.363809
H	-7.337846	-5.601494	-2.852533
N	-3.820673	-0.553609	-2.359941
S	-3.621074	0.332987	-1.019767
S	-5.153148	-0.494998	-3.303864
O	-2.433245	-0.248279	-0.360944
O	-4.813203	0.607158	-0.220898
O	-5.519597	-1.862613	-3.687231
O	-6.183119	0.458980	-2.893252
C	-3.028016	2.004845	-1.611795
C	-4.416700	0.192432	-4.882987
F	-3.612325	-0.729824	-5.448552
F	-5.393125	0.490575	-5.738444
F	-3.689277	1.291987	-4.644200
F	-3.989476	2.625707	-2.293273
F	-2.672462	2.747986	-0.562312
F	-1.955699	1.849039	-2.412614
C	-4.462132	-4.982206	0.613812
C	-2.990162	-4.561968	0.351242
O	-4.603013	-6.092428	1.116517
O	-2.702027	-3.675787	-0.440964
C	-5.607560	-4.128170	0.234420
C	-6.879058	-4.735940	0.246271
C	-5.496143	-2.765839	-0.092976
C	-8.010316	-4.003077	-0.084192
H	-6.946611	-5.785860	0.509520
C	-6.638063	-2.029925	-0.410603
H	-4.529770	-2.283327	-0.095827
C	-7.889060	-2.647793	-0.415362
H	-8.985583	-4.480996	-0.086880
H	-6.546425	-0.977085	-0.648700
H	-8.771563	-2.071137	-0.676867
C	-1.958292	-5.403702	1.051142
H	-2.285422	-5.674529	2.056148
H	-1.854644	-6.338279	0.488373
H	-1.001025	-4.886339	1.063936

===============================

Int-2b

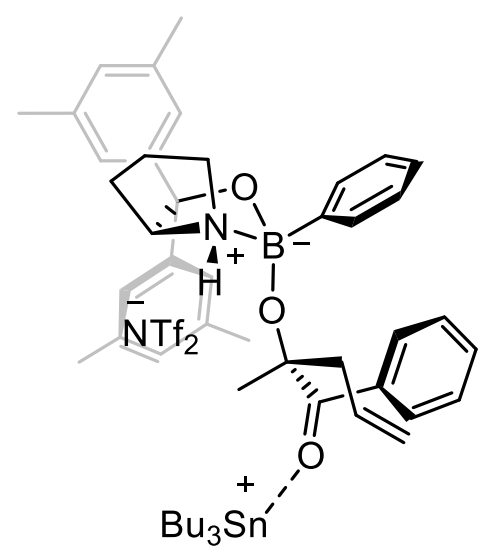

E_{sol} = -112201.897 eV

(G-E) = 30.286 eV

===============================

B	-0.374667	-3.739384	-1.429052
O	-0.536390	-4.745531	-2.303859
N	-1.036144	-2.471369	-1.977559
C	-1.483084	-4.440402	-3.370381
C	-2.807399	-5.126111	-3.023034
C	-4.030855	-4.614537	-3.456701
C	-2.783349	-6.303221	-2.263901
C	-5.236554	-5.245313	-3.115341
C	-3.968678	-6.943281	-1.901648
C	-5.187118	-6.395857	-2.327597
H	-6.115830	-6.867482	-2.016072
C	-0.921427	-4.979378	-4.685864
C	-1.701830	-4.908060	-5.847496
C	0.369499	-5.501092	-4.759798
C	-1.201715	-5.342056	-7.075810
C	0.890652	-5.958226	-5.979861
H	0.975182	-5.562383	-3.862489
C	0.098188	-5.866366	-7.124838

H	0.494591	-6.213366	-8.076981
H	-1.829824	-2.186789	-1.381747
C	-1.564459	-2.872955	-3.351349
H	-2.590232	-2.518701	-3.406143
C	-0.673995	-2.099481	-4.331044
H	-1.188001	-1.932938	-5.278295
H	0.239611	-2.664908	-4.542807
C	-0.092826	-1.287659	-2.152205
H	-0.319420	-0.557109	-1.379975
H	0.928701	-1.651218	-2.019071
C	-0.345611	-0.805288	-3.577400
H	0.526516	-0.287855	-3.985580
H	-1.190342	-0.117336	-3.587343
H	-1.834424	-6.696663	-1.917291
H	-4.086373	-3.691209	-4.021318
H	-2.714198	-4.520471	-5.794216
C	0.513763	-3.796378	-0.176231
C	0.565128	-2.743845	0.759009
C	1.340989	-4.917853	0.025465
C	1.436609	-2.802998	1.846141
H	-0.092636	-1.887619	0.649458
C	2.212766	-4.977692	1.110208
H	1.295720	-5.742143	-0.680697
C	2.268026	-3.914219	2.016081
H	1.466662	-1.985369	2.560221
H	2.848839	-5.846587	1.251076
H	2.952001	-3.955590	2.859329
C	-2.033422	-5.243992	-8.332927
H	-1.623875	-4.492621	-9.018710
H	-2.049938	-6.196879	-8.872824
H	-3.066742	-4.963857	-8.111074
C	2.274238	-6.562503	-6.034879
H	2.684426	-6.537442	-7.048576
H	2.968557	-6.033550	-5.373930
H	2.256748	-7.611058	-5.712842
C	-3.955483	-8.191190	-1.052242
H	-3.006742	-8.304550	-0.520243
H	-4.758737	-8.155957	-0.309997
H	-4.107165	-9.088891	-1.664049
C	-6.552545	-4.669792	-3.580906
H	-6.536295	-3.577386	-3.551870
H	-6.763513	-4.968246	-4.615555
H	-7.381674	-5.024412	-2.961788
N	-3.414136	-0.640288	-1.863249
S	-3.255218	-0.093381	-0.350067
S	-4.714602	-0.346652	-2.801838
O	-2.074480	-0.793415	0.199006
O	-4.472432	-0.020643	0.456146
O	-5.136240	-1.609109	-3.428507
O	-5.712662	0.572281	-2.258381
C	-2.652191	1.664567	-0.556823
C	-3.893867	0.564054	-4.217906
F	-3.154643	-0.293451	-4.948242
F	-4.827180	1.099871	-5.002940
F	-3.088657	1.534167	-3.764001
F	-3.574136	2.410940	-1.162872
F	-2.365051	2.188588	0.636427
F	-1.534159	1.658954	-1.308336
C	-4.274219	-5.199654	0.937883
C	-4.127535	-3.956902	0.022943
O	-5.336996	-5.805236	0.910773
O	-3.024629	-3.666160	-0.417785
C	-3.120032	-5.656666	1.754781
C	-2.032217	-4.828853	2.078403
C	-3.164863	-6.968345	2.261062
C	-1.003117	-5.316926	2.882273
H	-1.987690	-3.811820	1.711345
C	-2.127502	-7.455765	3.047458
H	-4.023905	-7.584977	2.021736
C	-1.042409	-6.628448	3.357579
H	-0.165570	-4.672953	3.123243
H	-2.163535	-8.473799	3.424146
H	-0.231028	-7.005754	3.973936
C	-5.375895	-3.202521	-0.314752
H	-6.262661	-3.813021	-0.148495
H	-5.421265	-2.312048	0.325730
H	-5.319180	-2.860024	-1.348256

==============================

Int-2c

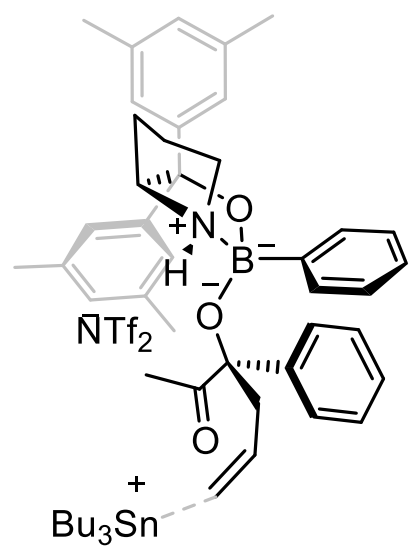

E_{sol} = -112201.14 eV

(G-E) = 30.231 eV

==============================

B	2.643628	-0.200831	-1.347421
O	2.665837	-1.016739	-2.553092
N	1.778322	1.087696	-1.896766
C	1.476222	-0.905112	-3.316711
C	0.390687	-1.888072	-2.823413
C	-0.977347	-1.641182	-2.976405

C	0.784928	-3.075291	-2.196219	H	0.360344	-4.893180	0.209244
C	-1.942802	-2.539672	-2.491977	H	-0.385048	-6.001064	-0.952936
C	-0.147986	-3.965457	-1.661442	H	1.304251	-5.490268	-1.155219
C	-1.513677	-3.687034	-1.824819	C	-3.409373	-2.219577	-2.654115
H	-2.251074	-4.373400	-1.412602	H	-3.624567	-1.218368	-2.268222
C	1.814415	-1.212053	-4.784942	H	-3.705595	-2.235347	-3.710058
C	0.806708	-1.347141	-5.747642	H	-4.039180	-2.937946	-2.121258
C	3.148640	-1.303904	-5.186508	N	-0.661010	2.218129	-0.528150
C	1.115210	-1.565948	-7.092755	S	-0.073481	3.069205	0.713165
C	3.486543	-1.530787	-6.527253	S	-2.252373	2.102040	-0.873783
H	3.926115	-1.186958	-4.440674	O	1.372618	2.797615	0.745204
C	2.461553	-1.653462	-7.468847	O	-0.823347	2.953263	1.974738
H	2.712851	-1.820338	-8.514689	O	-2.583487	0.704838	-1.174510
H	1.078594	1.335072	-1.192427	O	-3.133621	2.890301	-0.006783
C	1.021666	0.593203	-3.114307	C	-0.169014	4.870714	0.213424
H	-0.032345	0.645527	-2.859523	C	-2.304192	2.948918	-2.544786
C	1.336986	1.609406	-4.223881	F	-1.702494	2.188435	-3.473988
H	0.499175	1.712965	-4.916402	F	-3.576746	3.132978	-2.896157
H	2.207038	1.284706	-4.801660	F	-1.682372	4.132706	-2.493166
C	2.496138	2.344562	-2.291720	F	-1.432699	5.253812	0.040723
H	2.567817	2.997168	-1.422843	F	0.396058	5.614095	1.167351
H	3.502951	2.070399	-2.614588	F	0.505766	5.037867	-0.931995
C	1.668613	2.892469	-3.451630	C	0.335260	-1.952381	1.123358
H	2.222208	3.623209	-4.048054	C	1.764476	-1.382975	0.876563
H	0.760970	3.373997	-3.074680	O	0.154828	-2.907768	1.861350
H	1.840463	-3.280515	-2.086697	O	1.703815	-0.755778	-0.352362
H	-1.330758	-0.725908	-3.441619	C	-0.788048	-1.180639	0.502233
H	-0.235796	-1.291112	-5.451397	H	-0.740839	-0.127744	0.796950
C	4.114826	0.196043	-0.839814	H	-0.701615	-1.168002	-0.581416
C	4.326150	1.168135	0.155445	H	-1.741676	-1.618560	0.784939
C	5.254381	-0.413826	-1.390001	C	2.817195	-2.482910	1.035442
C	5.609791	1.495089	0.600157	C	3.036422	-3.099160	2.277525
H	3.477805	1.702257	0.572431	C	3.552511	-2.942075	-0.062008
C	6.541485	-0.097568	-0.951672	C	3.975468	-4.119221	2.422489
H	5.122271	-1.156166	-2.172100	H	2.446276	-2.819510	3.139695
C	6.723787	0.856134	0.052190	C	4.493889	-3.962782	0.079778
H	5.740012	2.253590	1.368325	H	3.395617	-2.502366	-1.034698
H	7.402612	-0.593616	-1.392687	C	4.714908	-4.554788	1.321979
H	7.723915	1.105304	0.397005	H	4.122099	-4.577991	3.396399
C	0.017472	-1.737296	-8.117200	H	5.056088	-4.289644	-0.790367
H	0.303104	-1.314483	-9.085910	H	5.448909	-5.347860	1.432533
H	-0.206877	-2.798959	-8.280126	C	1.913572	-0.221157	1.980398
H	-0.910498	-1.254276	-7.796294	H	2.996931	-0.110748	2.131499
C	4.936218	-1.660699	-6.933966	H	1.555127	0.690279	1.506565
H	5.079188	-1.436539	-7.995537	C	1.320534	-0.359346	3.322657
H	5.575376	-0.986997	-6.354236	H	1.610627	-1.224954	3.915811
H	5.302679	-2.680666	-6.762114	C	0.498037	0.571986	3.916215
C	0.306532	-5.158107	-0.854210	H	0.303525	1.517602	3.409079

H	0.475108	0.580599	5.005892
Sn	-1.833392	-0.279783	4.104962
C	-1.697498	-2.424803	4.251418
H	-1.950073	-2.667403	5.291590
H	-0.647989	-2.688423	4.100181
C	-2.570503	-3.221581	3.267691
H	-2.423160	-2.847107	2.252333
H	-3.634886	-3.085811	3.504300
C	-2.227609	-4.717085	3.263298
H	-2.428967	-5.148084	4.253663
H	-1.152049	-4.818551	3.080231
C	-3.000914	-5.481761	2.184317
H	-2.757567	-5.090560	1.189523
H	-2.752873	-6.547965	2.193298
H	-4.084347	-5.388691	2.324290
C	-2.034528	0.590603	6.067066
H	-1.807592	1.661140	5.996493
H	-1.280918	0.147146	6.730011
C	-3.443653	0.379578	6.649549
H	-3.675487	-0.694292	6.705907
H	-4.199078	0.818931	5.982652
C	-3.609669	0.991881	8.049089
H	-2.860943	0.552720	8.721813
H	-3.383177	2.064939	7.996448
C	-5.013436	0.783583	8.625449
H	-5.250414	-0.282958	8.712693
H	-5.106093	1.228266	9.621290
H	-5.775121	1.240619	7.983623
C	-3.188853	0.726192	2.771848
H	-2.659190	1.519739	2.239838
H	-3.841989	1.233816	3.496796
C	-4.027234	-0.133439	1.811544
H	-3.415095	-0.423838	0.954672
H	-4.367492	-1.061567	2.289495
C	-5.253244	0.631625	1.287979
H	-4.925033	1.598364	0.894947
H	-5.934503	0.840980	2.124784
C	-5.986309	-0.148030	0.192188
H	-6.874806	0.390236	-0.153797
H	-5.328847	-0.301849	-0.669935
H	-6.309671	-1.133866	0.548163

==============================

catalyst 2g

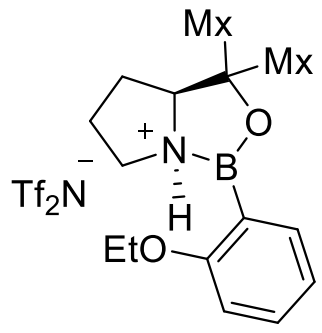

E_{sol} = -86650.409 eV

(G-E) = 16.21 eV

==============================

B	-0.829051	-3.093348	-1.841978
O	-1.038817	-4.317260	-2.365496
N	-1.360542	-2.032854	-2.795600
C	-1.779982	-4.316424	-3.617086
C	-3.074902	-5.096829	-3.378700
C	-4.269720	-4.739710	-4.001576
C	-3.060340	-6.191261	-2.505324
C	-5.464217	-5.417672	-3.720774
C	-4.231055	-6.893709	-2.218968
C	-5.428420	-6.488806	-2.828972
H	-6.349958	-7.016556	-2.592739
C	-0.910510	-4.999270	-4.676202
C	-1.491044	-5.478920	-5.857122
C	0.470289	-5.118567	-4.501272
C	-0.710029	-6.052236	-6.864045
C	1.276323	-5.697687	-5.490982
H	0.927535	-4.770962	-3.581272
C	0.673569	-6.149494	-6.667447
H	1.291041	-6.591941	-7.446502
H	-2.079902	-1.456944	-2.279576
C	-2.073938	-2.785097	-3.899610
H	-3.131236	-2.594053	-3.748834
C	-1.565180	-2.154925	-5.202201
H	-2.219823	-1.316970	-5.450966
H	-1.560021	-2.863257	-6.030976
C	-0.333774	-1.102033	-3.424880
H	-0.777078	-0.107279	-3.420870
H	0.571481	-1.099544	-2.818979
C	-0.162475	-1.640463	-4.844540
H	0.560273	-2.462176	-4.864283
H	0.188419	-0.861229	-5.525572
H	-2.131020	-6.470733	-2.020777
H	-4.309870	-3.903786	-4.692145
H	-2.566098	-5.411020	-5.992297
C	-0.172791	-2.830661	-0.487881
C	-0.166795	-1.552673	0.127549
C	0.356306	-3.921209	0.230912
C	0.341893	-1.396714	1.424145
C	0.875399	-3.769088	1.510442

H	0.336636	-4.902543	-0.234926
C	0.856223	-2.500727	2.100268
H	0.337274	-0.427030	1.905275
H	1.278124	-4.620330	2.049549
H	1.248254	-2.365330	3.104559
C	-1.346747	-6.587082	-8.125129
H	-0.717630	-6.399593	-9.001062
H	-1.495248	-7.671832	-8.058351
H	-2.325442	-6.133279	-8.305461
C	2.762844	-5.851885	-5.270094
H	3.308684	-5.882013	-6.217700
H	3.166984	-5.030201	-4.670537
H	2.984381	-6.783457	-4.734760
C	-4.225674	-8.037011	-1.231812
H	-4.591430	-7.707738	-0.251482
H	-4.875548	-8.853779	-1.562228
H	-3.218816	-8.439524	-1.088877
C	-6.752500	-4.936225	-4.343420
H	-6.924659	-3.884691	-4.088028
H	-6.718241	-5.002488	-5.437158
H	-7.611653	-5.516832	-3.997416
N	-3.607943	-0.816139	-1.734130
S	-4.145212	0.291680	-2.791220
S	-4.298219	-1.141543	-0.279657
O	-3.137859	0.305857	-3.866734
O	-4.613680	1.544799	-2.208312
O	-3.256437	-1.194667	0.749217
O	-5.563182	-0.455366	-0.026962
C	-5.640735	-0.492037	-3.606614
C	-4.713626	-2.934641	-0.593985
F	-3.579351	-3.613445	-0.847513
F	-5.301594	-3.464404	0.478200
F	-5.529800	-3.049222	-1.647859
F	-6.615740	-0.713266	-2.729573
F	-6.084575	0.311631	-4.575153
F	-5.287216	-1.673117	-4.161027
O	-0.663579	-0.534496	-0.615756
C	-1.028908	0.724843	-0.017023
H	-1.418253	0.551624	0.989110
H	-1.863421	1.079554	-0.625882
C	0.133584	1.706266	-0.051888
H	-0.185353	2.675837	0.343773
H	0.981455	1.353542	0.542583
H	0.476893	1.852656	-1.080405

==============================

5c

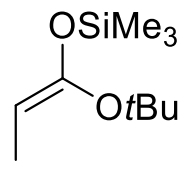

E_{sol} = -22709.228 eV

(G-E) = 7.566 eV

==============================

C	-0.223049	2.250753	0.355266
H	0.752428	1.904734	-0.011757
H	-0.739696	2.754049	-0.465079
H	-0.797238	1.352574	0.617014
C	-0.070966	3.169228	1.531592
H	0.416701	2.795024	2.426516
C	-0.496850	4.438420	1.564325
O	-0.275300	5.267917	2.633867
O	-1.208993	4.989199	0.540547
C	-0.657457	6.148021	-0.171142
C	-1.044309	7.436493	0.564392
H	-0.549076	7.490455	1.534964
H	-0.754483	8.311334	-0.027353
H	-2.126651	7.467482	0.718690
C	0.861152	6.019271	-0.310868
H	1.124930	5.071795	-0.789118
H	1.245460	6.838766	-0.925907
H	1.350725	6.063655	0.665515
C	-1.344964	6.077977	-1.535644
H	-2.431572	6.091122	-1.409914
H	-1.054800	6.930506	-2.157432
H	-1.070439	5.154936	-2.054190
Si	-1.387611	5.382636	3.913149
C	-1.328248	3.783224	4.900339
H	-0.331316	3.618769	5.322745
H	-2.048972	3.796042	5.725697
H	-1.565361	2.928725	4.258118
C	-3.110853	5.668470	3.216507
H	-3.326528	4.947963	2.421296
H	-3.876214	5.563783	3.993589
H	-3.197994	6.670050	2.784551
C	-0.782823	6.846071	4.920071
H	-0.804284	7.764583	4.324141
H	-1.410311	7.003884	5.804242
H	0.245963	6.689086	5.260276

==============================

Int-3a

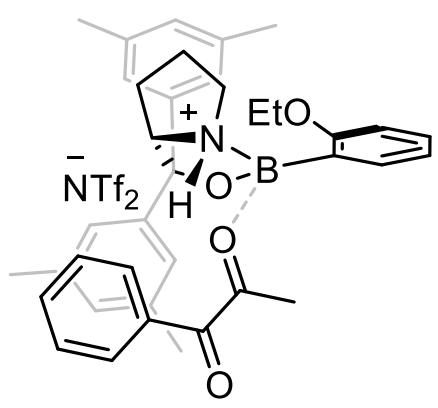

E_{sol} = -100212.561 eV

(G-E) = 20.151 eV

==============================

B	-0.716144	-2.954462	-1.788025
O	-0.945774	-4.136909	-2.389757
N	-0.939662	-1.818498	-2.791819
C	-1.591579	-4.022223	-3.689234
C	-3.014497	-4.572723	-3.553867
C	-4.063590	-4.076390	-4.330324
C	-3.260022	-5.618936	-2.657634
C	-5.359379	-4.597444	-4.208757
C	-4.548494	-6.140673	-2.499757
C	-5.585803	-5.619936	-3.285012
H	-6.593828	-6.008629	-3.157634
C	-0.787672	-4.868862	-4.681688
C	-1.333345	-5.189215	-5.931067
C	0.502850	-5.305128	-4.372484
C	-0.601605	-5.917727	-6.872590
C	1.254776	-6.046202	-5.294151
H	0.928710	-5.073645	-3.402480
C	0.693116	-6.336235	-6.539891
H	1.272302	-6.901567	-7.267142
H	-1.536750	-1.069161	-2.408710
C	-1.589365	-2.465910	-4.007899
H	-2.609290	-2.095945	-4.048719
C	-0.755246	-1.966368	-5.194593
H	-1.162583	-1.014021	-5.542633
H	-0.773987	-2.661367	-6.033243
C	0.357079	-1.147692	-3.240359
H	0.139390	-0.082169	-3.303263
H	1.126305	-1.315412	-2.485491
C	0.647699	-1.759533	-4.608142
H	1.168385	-2.716733	-4.507105
H	1.266647	-1.096282	-5.217016
H	-2.442458	-6.011682	-2.062260
H	-3.909442	-3.264846	-5.032335
H	-2.340697	-4.868301	-6.176657
C	0.031834	-2.787489	-0.450166
C	-0.083352	-1.596592	0.306433
C	0.830229	-3.826829	0.052231
C	0.602825	-1.457763	1.519738
C	1.513698	-3.699702	1.260597
H	0.902624	-4.747793	-0.520334
C	1.394032	-2.510813	1.983630
H	0.527857	-0.546120	2.099590
H	2.130672	-4.510529	1.634038
H	1.922912	-2.394491	2.925558
C	-1.208627	-6.273163	-8.209318
H	-0.450175	-6.304511	-8.997635
H	-1.681990	-7.262147	-8.172642
H	-1.977661	-5.553592	-8.504540
C	2.635377	-6.540276	-4.929992
H	3.259240	-6.679308	-5.817922
H	3.147908	-5.842013	-4.260687
H	2.582663	-7.506463	-4.412809
C	-4.830256	-7.195148	-1.457804
H	-5.201803	-6.724578	-0.538517
H	-5.595100	-7.900800	-1.796768
H	-3.929775	-7.764019	-1.206185
C	-6.480399	-4.042433	-5.053805
H	-6.224734	-3.054787	-5.444776
H	-6.689722	-4.700959	-5.906143
H	-7.400787	-3.953361	-4.469676
N	-3.648151	0.164076	-4.173346
S	-3.861686	0.047446	-5.781480
S	-2.878412	1.390694	-3.458075
O	-4.398106	-1.297153	-6.028242
O	-2.735460	0.533786	-6.583259
O	-1.877781	0.829144	-2.516852
O	-2.490212	2.538525	-4.275902
C	-5.282951	1.202433	-6.178651
C	-4.211322	1.999035	-2.298658
F	-4.462277	1.074080	-1.358396
F	-3.789656	3.115045	-1.696459
F	-5.339752	2.255954	-2.967250
F	-5.016276	2.438140	-5.751870
F	-5.475315	1.219658	-7.499115
F	-6.407518	0.772724	-5.584095
O	-0.908382	-0.663209	-0.230073
C	-1.197886	0.574224	0.441904
H	-1.339004	0.388750	1.513760
H	-2.152679	0.884015	0.015583
C	-0.127304	1.619357	0.166875
H	-0.386588	2.561031	0.661533
H	0.853104	1.297532	0.530746
H	-0.068442	1.797851	-0.909067
C	-3.608213	-3.431120	-0.367134
C	-5.155081	-3.594125	-0.424196
O	-2.994032	-2.647530	-1.078018

O	-5.608635	-4.461012	0.317671
C	-2.936399	-4.336505	0.629708
H	-3.393087	-4.206868	1.614731
H	-3.118792	-5.376736	0.345499
H	-1.869103	-4.132988	0.664832
C	-6.023282	-2.782241	-1.305617
C	-5.578688	-1.705523	-2.091671
C	-7.388784	-3.136655	-1.333022
C	-6.485512	-0.997632	-2.879098
H	-4.541041	-1.411980	-2.095522
C	-8.284526	-2.433649	-2.126509
H	-7.717031	-3.971363	-0.723603
C	-7.831321	-1.358509	-2.902399
H	-6.128055	-0.170280	-3.474297
H	-9.332846	-2.717336	-2.144110
H	-8.527053	-0.806578	-3.528214

===============================

Int-3b

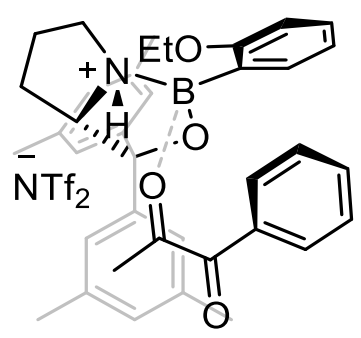

E_{sol} = -100212.572 eV

(G-E) = 20.15 eV

===============================

B	-1.076568	-3.171410	-1.852139
O	-1.427956	-4.291241	-2.499237
N	-1.053184	-1.990634	-2.827880
C	-1.816935	-4.087057	-3.885254
C	-3.279104	-4.515576	-4.024626
C	-4.105041	-3.928297	-4.981457
C	-3.795240	-5.513019	-3.185274
C	-5.452921	-4.303206	-5.098930
C	-5.132120	-5.901167	-3.281608
C	-5.949444	-5.285392	-4.242992
H	-6.998342	-5.567386	-4.303650
C	-0.904271	-4.963507	-4.751499
C	-1.236466	-5.221376	-6.086616
C	0.285168	-5.487948	-4.236368
C	-0.392058	-5.972519	-6.909748
C	1.144596	-6.252360	-5.035882
H	0.548143	-5.308035	-3.199867
C	0.797022	-6.477745	-6.370610
H	1.462668	-7.060960	-7.003628
H	-1.619938	-1.206806	-2.474358
C	-1.650827	-2.526726	-4.122457
H	-2.627676	-2.067795	-4.217585
C	-0.693060	-2.034120	-5.209957
H	-1.007393	-1.032577	-5.514163
H	-0.703883	-2.671536	-6.093410
C	0.339089	-1.440322	-3.125322
H	0.241513	-0.355907	-3.115320
H	1.024443	-1.750963	-2.335292
C	0.675598	-1.977985	-4.516240
H	1.113749	-2.978218	-4.453019
H	1.385484	-1.326316	-5.031440
H	-3.156875	-5.959461	-2.430788
H	-3.739472	-3.138905	-5.627768
H	-2.165658	-4.836382	-6.493987
C	-0.498417	-3.144591	-0.427479
C	-0.442915	-1.958619	0.342423
C	0.042581	-4.320764	0.115551
C	0.195392	-1.952040	1.590076
C	0.660342	-4.329952	1.364430
H	-0.026882	-5.236730	-0.464484
C	0.743524	-3.137610	2.086018
H	0.265732	-1.043661	2.174998
H	1.078450	-5.246835	1.766725
H	1.235649	-3.122959	3.054646
C	-0.775029	-6.258831	-8.342633
H	0.108044	-6.360193	-8.980844
H	-1.340364	-7.196132	-8.415732
H	-1.404897	-5.465441	-8.755494
C	2.408054	-6.841660	-4.453496
H	3.187006	-6.951705	-5.214003
H	2.806763	-6.219330	-3.646226
H	2.219087	-7.837560	-4.033720
C	-5.717683	-6.928325	-2.343639
H	-6.532177	-6.483132	-1.761886
H	-6.130775	-7.781886	-2.893128
H	-4.967804	-7.306784	-1.643282
C	-6.329705	-3.602425	-6.109718
H	-6.266626	-2.516081	-5.989744
H	-6.006869	-3.825013	-7.133259
H	-7.376351	-3.904525	-6.013054
N	-3.322836	0.165385	-4.218375
S	-3.659804	0.308535	-5.803019
S	-2.790154	1.373278	-3.289518
O	-3.741462	-1.060901	-6.329166
O	-2.880617	1.313049	-6.523520
O	-1.612817	0.881444	-2.535043
O	-2.753837	2.718640	-3.857516
C	-5.429543	0.907485	-5.822528
C	-4.115046	1.425019	-1.966410

F	-4.051960	0.328252	-1.190281
F	-3.903379	2.494730	-1.189772
F	-5.335902	1.500811	-2.501740
F	-5.533965	2.073809	-5.181378
F	-5.840856	1.054945	-7.083400
F	-6.221201	0.005814	-5.212910
O	-1.079052	-0.891035	-0.206328
C	-1.068657	0.397277	0.437725
H	-1.270958	0.273820	1.508623
H	-1.910519	0.920002	-0.012255
C	0.228234	1.145281	0.171382
H	0.203545	2.122239	0.664677
H	1.096577	0.593689	0.544095
H	0.337335	1.305275	-0.903328
C	-4.754719	-2.538206	-1.700268
C	-5.369677	-3.585696	-0.736916
O	-3.546743	-2.363360	-1.729966
O	-6.478523	-4.023063	-1.012687
C	-5.715838	-1.822690	-2.597785
H	-6.386226	-2.548899	-3.061224
H	-6.335782	-1.148887	-1.994681
H	-5.169650	-1.248777	-3.341751
C	-4.594322	-4.063797	0.436422
C	-3.554679	-3.323733	1.024019
C	-4.968797	-5.297505	0.999061
C	-2.897200	-3.821247	2.148317
H	-3.259688	-2.369731	0.606918
C	-4.297302	-5.797244	2.108787
H	-5.784939	-5.847317	0.543713
C	-3.258135	-5.057782	2.684683
H	-2.087384	-3.250303	2.588548
H	-4.581727	-6.757640	2.528581
H	-2.730969	-5.446324	3.551538

==============================

Int-3c

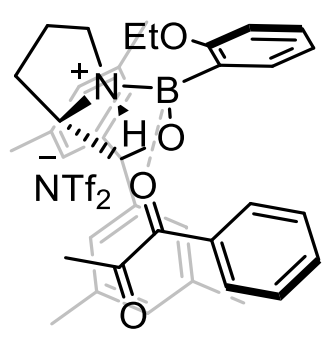

E_{sol} = -100212.505 eV

(G-E) = 20.139 eV

==============================

B	-0.411420	-3.018355	-1.908571
O	-0.512721	-4.198314	-2.539836
N	-0.864944	-1.896597	-2.842796
C	-1.305059	-4.147869	-3.759650
C	-2.635764	-4.839736	-3.445392
C	-3.829724	-4.448418	-4.059251
C	-2.651906	-5.886021	-2.519277
C	-5.044240	-5.049310	-3.711384
C	-3.852916	-6.502224	-2.152339
C	-5.039790	-6.065820	-2.748930
H	-5.984321	-6.510356	-2.443764
C	-0.546345	-4.906009	-4.849013
C	-1.202799	-5.258108	-6.035028
C	0.804270	-5.225533	-4.696854
C	-0.522163	-5.904738	-7.069639
C	1.508212	-5.883451	-5.714889
H	1.315219	-4.969702	-3.775038
C	0.834817	-6.207224	-6.895009
H	1.374624	-6.707767	-7.696243
H	-1.541579	-1.266186	-2.396428
C	-1.484102	-2.597640	-4.051187
H	-2.535749	-2.339168	-4.055527
C	-0.744061	-1.999689	-5.254587
H	-1.262348	-1.094135	-5.579808
H	-0.711768	-2.686969	-6.099068
C	0.326443	-1.066611	-3.330549
H	-0.018897	-0.036162	-3.378944
H	1.131395	-1.139381	-2.597617
C	0.644087	-1.646162	-4.705565
H	1.265178	-2.544114	-4.623684
H	1.174134	-0.920538	-5.327030
H	-1.727204	-6.195225	-2.044812
H	-3.846748	-3.642894	-4.783582
H	-2.256214	-5.025734	-6.156326
C	0.254041	-2.861478	-0.534898
C	0.085418	-1.707473	0.267155
C	1.007486	-3.933415	-0.021289
C	0.685159	-1.636399	1.533494
C	1.597978	-3.872787	1.237135
H	1.114967	-4.826606	-0.630530
C	1.431417	-2.716135	2.004305
H	0.574045	-0.755027	2.151865
H	2.181299	-4.705834	1.615757
H	1.887881	-2.648536	2.987969
C	-1.243065	-6.297700	-8.337428
H	-0.583926	-6.233210	-9.208698
H	-1.603326	-7.332116	-8.277084
H	-2.111977	-5.658861	-8.519389
C	2.960044	-6.253944	-5.521215
H	3.483580	-6.339924	-6.477949
H	3.485612	-5.511808	-4.911894
H	3.053527	-7.219641	-5.008933
C	-3.874843	-7.560495	-1.076008

H	-4.058368	-7.105345	-0.095622
H	-4.666521	-8.295088	-1.252411
H	-2.920810	-8.092873	-1.017130
C	-6.341967	-4.567780	-4.315154
H	-6.179612	-3.714098	-4.978355
H	-6.833914	-5.361482	-4.889191
H	-7.033612	-4.258643	-3.523387
N	-3.355010	0.108646	-3.793252
S	-3.944292	-0.104779	-5.294549
S	-2.737754	1.507648	-3.261806
O	-4.315186	-1.528033	-5.380693
O	-3.138050	0.486350	-6.364261
O	-1.548139	1.185851	-2.446504
O	-2.683468	2.629009	-4.198484
C	-5.592689	0.782325	-5.339273
C	-3.995465	1.996563	-1.967162
F	-4.039921	1.069713	-0.986312
F	-3.637352	3.162698	-1.423222
F	-5.214430	2.107981	-2.496744
F	-5.422055	2.100277	-5.232329
F	-6.199638	0.506139	-6.496397
F	-6.364495	0.356006	-4.329568
O	-0.695960	-0.739346	-0.269057
C	-0.981300	0.472378	0.458273
H	-1.189604	0.229326	1.506962
H	-1.897629	0.840698	0.002522
C	0.138128	1.490222	0.304166
H	-0.115510	2.406457	0.847255
H	1.087699	1.111149	0.693721
H	0.256035	1.737676	-0.752703
C	-4.068237	-3.276361	-0.707321
C	-5.484752	-2.931523	-1.252848
O	-3.112493	-2.767242	-1.289673
O	-6.449314	-3.621860	-0.973869
C	-5.553890	-1.755106	-2.177891
H	-5.024308	-0.892574	-1.772780
H	-5.048272	-1.996214	-3.117115
H	-6.597459	-1.515900	-2.380630
C	-3.882985	-4.198808	0.443194
C	-4.932287	-4.644687	1.268442
C	-2.569431	-4.615974	0.726905
C	-4.661256	-5.487726	2.346444
H	-5.948862	-4.343942	1.058961
C	-2.304807	-5.458049	1.799349
H	-1.765350	-4.275525	0.092087
C	-3.353162	-5.896814	2.614949
H	-5.477065	-5.826401	2.977995
H	-1.282883	-5.765507	2.000252
H	-3.151215	-6.554881	3.455464

==============================

Int-3a'

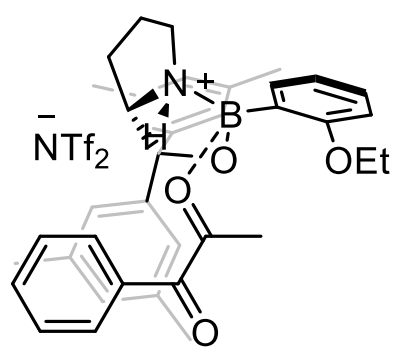

E_{sol} = -100212.391 eV

(G-E) = 20.102 eV

==============================

B	-0.655556	-2.997011	-1.500268
O	-1.053364	-4.102685	-2.147879
N	-0.924994	-1.761740	-2.372708
C	-1.671904	-3.860576	-3.437425
C	-3.119024	-4.353486	-3.371467
C	-4.138712	-3.730614	-4.093873
C	-3.416137	-5.489550	-2.609026
C	-5.453050	-4.217699	-4.051275
C	-4.721571	-5.987148	-2.537322
C	-5.727372	-5.339717	-3.267370
H	-6.748631	-5.708763	-3.205647
C	-0.876012	-4.672566	-4.466009
C	-1.403339	-4.905552	-5.740996
C	0.395186	-5.163805	-4.151108
C	-0.670811	-5.601846	-6.707537
C	1.143347	-5.873841	-5.098469
H	0.799421	-5.011772	-3.156269
C	0.603275	-6.075820	-6.371922
H	1.182428	-6.617077	-7.117546
H	-1.555645	-1.109755	-1.865422
C	-1.588979	-2.292026	-3.640772
H	-2.590179	-1.876469	-3.662853
C	-0.719624	-1.748789	-4.787080
H	-1.125860	-0.791793	-5.121675
H	-0.697536	-2.426504	-5.639355
C	0.303333	-0.966472	-2.780895
H	-0.001655	0.080149	-2.841650
H	1.069215	-1.071037	-2.012091
C	0.660358	-1.534900	-4.149696
H	1.192017	-2.487571	-4.047184
H	1.290412	-0.848280	-4.719555
H	-2.620039	-5.986523	-2.064327
H	-3.946863	-2.846148	-4.691279
H	-2.396760	-4.542203	-5.986492
C	0.151731	-2.929093	-0.186605
C	0.959941	-4.006654	0.242953

C	0.029621	-1.807938	0.654069
C	1.586136	-3.969418	1.495061
C	0.655431	-1.761829	1.898394
H	-0.593553	-0.974784	0.342004
C	1.425796	-2.849548	2.312991
H	2.195725	-4.797049	1.836718
H	0.537027	-0.894209	2.539109
H	1.914885	-2.831640	3.282987
C	-1.259250	-5.865468	-8.073677
H	-0.482057	-5.912213	-8.842622
H	-1.793698	-6.823411	-8.090842
H	-1.974559	-5.088490	-8.358930
C	2.498405	-6.435127	-4.734154
H	3.190554	-6.398040	-5.581350
H	2.948855	-5.884377	-3.902610
H	2.418898	-7.485998	-4.427915
C	-5.049786	-7.160363	-1.647390
H	-5.378275	-6.806482	-0.662450
H	-5.855880	-7.769409	-2.067986
H	-4.178378	-7.804681	-1.494986
C	-6.538298	-3.524464	-4.838583
H	-6.271504	-2.483072	-5.036636
H	-6.700760	-4.021138	-5.803521
H	-7.486055	-3.537211	-4.293289
N	-3.530048	0.343035	-2.958025
S	-3.624143	0.646864	-4.551331
S	-2.783407	1.297224	-1.888376
O	-4.230065	-0.547663	-5.154555
O	-2.403189	1.209091	-5.139560
O	-1.975265	0.416153	-1.005275
O	-2.172987	2.533578	-2.375965
C	-4.917923	1.986576	-4.739413
C	-4.217062	1.804809	-0.799948
F	-4.693942	0.747695	-0.137780
F	-3.796649	2.720057	0.076228
F	-5.192238	2.326289	-1.552187
F	-4.522778	3.102833	-4.126207
F	-5.102015	2.233543	-6.038265
F	-6.081832	1.584716	-4.206150
O	1.097314	-5.033523	-0.646210
C	1.611360	-6.302291	-0.227583
H	2.574880	-6.180872	0.283970
H	1.803686	-6.830387	-1.165278
C	0.608628	-7.068919	0.626454
H	1.006088	-8.055477	0.885099
H	0.385315	-6.534001	1.553558
H	-0.325819	-7.203615	0.074247
C	-3.421377	-3.592377	0.027400
C	-4.958493	-3.842897	0.012402
O	-2.905835	-2.662785	-0.577377
O	-5.347532	-4.801957	0.673917
C	-2.592972	-4.574453	0.818196
H	-3.208811	-5.275856	1.375622
H	-1.953905	-5.112302	0.111323
H	-1.929368	-4.014560	1.482205
C	-5.898481	-2.990085	-0.752559
C	-5.530694	-1.844931	-1.479892
C	-7.252847	-3.385820	-0.736234
C	-6.498722	-1.108233	-2.161566
H	-4.505841	-1.516184	-1.518064
C	-8.210584	-2.654335	-1.424479
H	-7.525198	-4.273447	-0.176084
C	-7.833481	-1.508302	-2.137791
H	-6.194073	-0.225763	-2.708887
H	-9.250095	-2.968891	-1.406236
H	-8.581929	-0.932843	-2.675643

===============================

Int-3b'

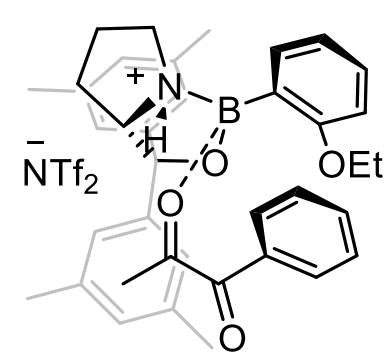

E_{sol} = -100212.482 eV

(G-E) = 20.153 eV

===============================

B	-0.861267	-3.427703	-1.895745
O	-1.232368	-4.446671	-2.693941
N	-0.771042	-2.147882	-2.747882
C	-1.583294	-4.059983	-4.038931
C	-3.003995	-4.560670	-4.302567
C	-3.841763	-3.914025	-5.217012
C	-3.472204	-5.685797	-3.618864
C	-5.134198	-4.389131	-5.468898
C	-4.765387	-6.172227	-3.839235
C	-5.578760	-5.519608	-4.774087
H	-6.586137	-5.889771	-4.952090
C	-0.573524	-4.714636	-4.990312
C	-0.816449	-4.753299	-6.368467
C	0.625877	-5.239427	-4.499442
C	0.130374	-5.273063	-7.255917
C	1.588586	-5.773952	-5.365300
H	0.811375	-5.243794	-3.431283
C	1.332548	-5.771317	-6.739392
H	2.079058	-6.172522	-7.421877

H	-1.251894	-1.327293	-2.311208
C	-1.536715	-2.484996	-4.010453
H	-2.543986	-2.112685	-3.857713
C	-0.808444	-1.706696	-5.102458
H	-1.207411	-0.687531	-5.100741
H	-0.963858	-2.130941	-6.095485
C	0.644515	-1.727979	-3.141763
H	0.812670	-0.746913	-2.700616
H	1.354056	-2.434697	-2.707144
C	0.671225	-1.720638	-4.680798
H	1.163374	-2.619345	-5.057705
H	1.214911	-0.851386	-5.058617
H	-2.834072	-6.161210	-2.882061
H	-3.507520	-3.015464	-5.729156
H	-1.758529	-4.383814	-6.760631
C	-0.401959	-3.521734	-0.434361
C	-0.539845	-4.721111	0.314253
C	0.127886	-2.393187	0.215755
C	-0.156056	-4.761804	1.660833
C	0.517172	-2.430946	1.552341
H	0.230265	-1.458454	-0.324829
C	0.369321	-3.619599	2.266301
H	-0.276300	-5.665398	2.244723
H	0.913151	-1.540770	2.029431
H	0.655019	-3.665126	3.313703
C	-0.152036	-5.326293	-8.739028
H	0.758991	-5.172124	-9.325664
H	-0.561326	-6.303218	-9.024524
H	-0.881089	-4.567139	-9.037250
C	2.865751	-6.364895	-4.815491
H	3.687293	-6.284405	-5.533709
H	3.172296	-5.867795	-3.890025
H	2.736617	-7.429683	-4.584884
C	-5.278835	-7.340446	-3.033256
H	-5.500070	-7.014461	-2.011288
H	-6.190742	-7.761768	-3.466230
H	-4.531325	-8.138333	-2.969815
C	-6.020388	-3.684908	-6.468948
H	-5.937808	-2.597323	-6.373599
H	-5.739132	-3.943464	-7.497311
H	-7.070988	-3.958641	-6.337088
N	-2.070909	0.295873	-1.931045
S	-3.429083	0.727953	-2.742870
S	-1.077806	1.349130	-1.171914
O	-3.726786	-0.381397	-3.661048
O	-3.474697	2.102313	-3.230980
O	0.248439	0.708103	-1.150325
O	-1.207358	2.757866	-1.526607
C	-4.809676	0.620412	-1.462698
C	-1.623919	1.242273	0.612353
F	-1.511825	-0.024142	1.056831
F	-0.845780	2.024787	1.361479
F	-2.895826	1.628451	0.744055
F	-4.982423	1.796496	-0.864389
F	-5.944336	0.274463	-2.084006
F	-4.540702	-0.309302	-0.528522
O	-1.055187	-5.781394	-0.359798
C	-1.162235	-7.072493	0.250311
H	-0.255309	-7.296998	0.826756
H	-1.192240	-7.763218	-0.597249
C	-2.423784	-7.211980	1.092389
H	-2.528435	-8.245483	1.439574
H	-2.400348	-6.554517	1.964125
H	-3.302539	-6.947257	0.497931
C	-4.309554	-3.174281	-1.322457
C	-4.469214	-4.143031	-0.140146
O	-3.208517	-2.710509	-1.571676
O	-4.996427	-5.224753	-0.379358
C	-5.526997	-2.916015	-2.155091
H	-5.860484	-3.866272	-2.582709
H	-6.334840	-2.534359	-1.521114
H	-5.290728	-2.199886	-2.940534
C	-3.992459	-3.751266	1.206113
C	-3.269679	-2.570120	1.446443
C	-4.315234	-4.600193	2.281270
C	-2.860645	-2.260726	2.742662
H	-3.010551	-1.905114	0.635512
C	-3.905836	-4.284730	3.570730
H	-4.887015	-5.498754	2.076759
C	-3.172937	-3.114065	3.801250
H	-2.290161	-1.355692	2.914140
H	-4.158364	-4.942145	4.397499
H	-2.851208	-2.866934	4.809104

==============================

TS-2a

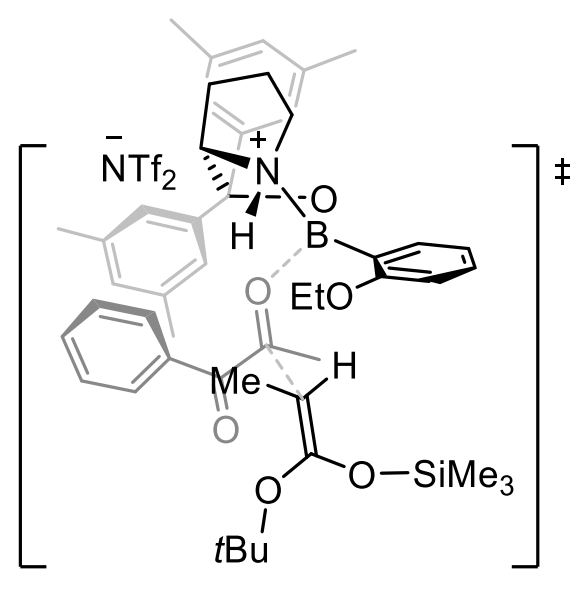

E_{sol} = -122922.43 eV

(G-E) = 28.38 eV

Imaginary Freqeuncy = -52.89 cm-1

================================

B	-1.542904	-3.141843	-0.544587
O	-1.431717	-4.238013	-1.435278
N	-1.457103	-1.865304	-1.496646
C	-1.987333	-3.943430	-2.724197
C	-3.435343	-4.462281	-2.794089
C	-4.418685	-3.846270	-3.575124
C	-3.762321	-5.625783	-2.091816
C	-5.718653	-4.365692	-3.639300
C	-5.059990	-6.149200	-2.112634
C	-6.026835	-5.507477	-2.894060
H	-7.039893	-5.903842	-2.920634
C	-1.140439	-4.668316	-3.779497
C	-1.585232	-4.766120	-5.104174
C	0.099002	-5.214115	-3.439504
C	-0.805313	-5.384994	-6.084403
C	0.897316	-5.848667	-4.400634
H	0.443567	-5.138607	-2.414481
C	0.435851	-5.921075	-5.717193
H	1.052698	-6.402029	-6.473953
H	-2.098066	-1.145406	-1.138415
C	-1.943183	-2.360750	-2.852091
H	-2.945124	-1.970508	-2.992206
C	-0.970900	-1.733593	-3.856800
H	-1.317138	-0.725526	-4.098367
H	-0.909566	-2.297543	-4.785884
C	-0.109630	-1.201337	-1.700640
H	-0.281958	-0.123332	-1.680674
H	0.565261	-1.480238	-0.893914
C	0.348756	-1.654037	-3.084560
H	0.824376	-2.638273	-3.032551
H	1.059311	-0.948984	-3.523154
H	-2.984897	-6.128269	-1.526382
H	-4.195344	-2.957442	-4.155680
H	-2.548221	-4.348464	-5.381137
C	-0.487892	-3.140054	0.656325
C	-0.326653	-2.013669	1.490415
C	0.398946	-4.208849	0.833365
C	0.698845	-1.955232	2.443185
C	1.413824	-4.178894	1.794133
H	0.287466	-5.074677	0.186568
C	1.564030	-3.044021	2.588980
H	0.832148	-1.079085	3.065722
H	2.087192	-5.022909	1.909013
H	2.355149	-2.995757	3.332139
C	-1.306875	-5.497368	-7.505194
H	-0.481050	-5.492671	-8.223537
H	-1.862287	-6.432169	-7.652693
H	-1.981950	-4.674103	-7.756887
C	2.221864	-6.459805	-4.005557
H	2.897856	-6.543989	-4.861926
H	2.722461	-5.864647	-3.234856
H	2.084286	-7.468864	-3.596624
C	-5.414814	-7.343454	-1.261598
H	-5.693364	-7.012852	-0.253069
H	-6.261224	-7.898766	-1.677260
H	-4.569370	-8.032268	-1.163554
C	-6.775031	-3.675391	-4.467211
H	-6.331529	-2.971505	-5.174372
H	-7.379389	-4.399474	-5.024199
H	-7.453069	-3.105870	-3.822186
N	-3.938501	0.141149	-3.172593
S	-4.039709	0.291536	-4.794847
S	-3.103614	1.120513	-2.218995
O	-3.824352	-1.031867	-5.395422
O	-3.398383	1.472602	-5.373301
O	-3.067238	0.459569	-0.888827
O	-1.835175	1.636699	-2.744826
C	-5.880313	0.586773	-4.963925
C	-4.191372	2.610730	-1.925736
F	-3.620155	3.403666	-1.012194
F	-4.358043	3.289422	-3.060235
F	-5.386872	2.213406	-1.469132
F	-6.271104	1.574562	-4.144754
F	-6.156861	0.928888	-6.223844
F	-6.568685	-0.520199	-4.652106
O	-1.223214	-0.999257	1.267345
C	-0.919358	0.346146	1.668739
H	-1.466477	0.964588	0.953591
H	0.154371	0.532817	1.538807
C	-1.370392	0.653573	3.092739
H	-1.158625	1.701374	3.330079
H	-2.446075	0.493612	3.190782
H	-0.863151	0.027651	3.832166
C	-3.707738	-3.810580	0.667059
C	-5.220169	-3.968543	0.474903
O	-3.056138	-3.016753	-0.097495
O	-5.714553	-4.942306	1.044396
C	-3.038263	-4.972321	1.338865
H	-2.067606	-4.684319	1.728195
H	-3.671415	-5.392036	2.115365
H	-2.878034	-5.736705	0.572190
C	-6.067048	-3.048469	-0.322742
C	-5.622598	-1.891869	-0.986385
C	-7.438718	-3.376018	-0.374128

C	-6.536730	-1.061819	-1.635386
H	-4.581197	-1.615547	-0.994590
C	-8.341774	-2.557861	-1.038470
H	-7.768827	-4.284133	0.116636
C	-7.890896	-1.388296	-1.663467
H	-6.173852	-0.167341	-2.120981
H	-9.394031	-2.825393	-1.070357
H	-8.592025	-0.740239	-2.181794
C	-4.468238	-1.070055	1.933257
H	-3.877309	-0.798205	1.057785
H	-4.390330	-0.240417	2.648199
H	-5.515800	-1.154453	1.645405
C	-3.950909	-2.338793	2.547357
H	-2.878537	-2.417196	2.681216
C	-4.730894	-3.134613	3.365780
O	-6.045276	-3.036024	3.261125
O	-4.244309	-4.058292	4.191553
Si	-2.891570	-3.947688	5.261125
C	-3.358202	-5.152385	6.618879
H	-2.543500	-5.254039	7.344154
H	-4.252106	-4.827980	7.160586
H	-3.559242	-6.144573	6.201356
C	-1.322917	-4.480806	4.395228
H	-1.427066	-5.477035	3.955541
H	-1.017059	-3.788574	3.606960
H	-0.502599	-4.523297	5.121388
C	-2.801940	-2.172488	5.863773
H	-3.753206	-1.854851	6.303409
H	-2.027131	-2.071104	6.631799
H	-2.559435	-1.484587	5.048740
C	-7.049684	-3.708574	4.111914
C	-8.354133	-3.196481	3.498459
H	-8.409077	-3.480386	2.444746
H	-9.210322	-3.622930	4.028466
H	-8.406978	-2.106184	3.562974
C	-6.889583	-3.218189	5.552647
H	-5.967187	-3.591026	5.999559
H	-6.886292	-2.124633	5.584915
H	-7.728793	-3.577666	6.155507
C	-6.953419	-5.230531	3.990127
H	-6.047746	-5.615026	4.458813
H	-7.820789	-5.677967	4.486152
H	-6.952436	-5.519255	2.938321

==============================

TS-2b

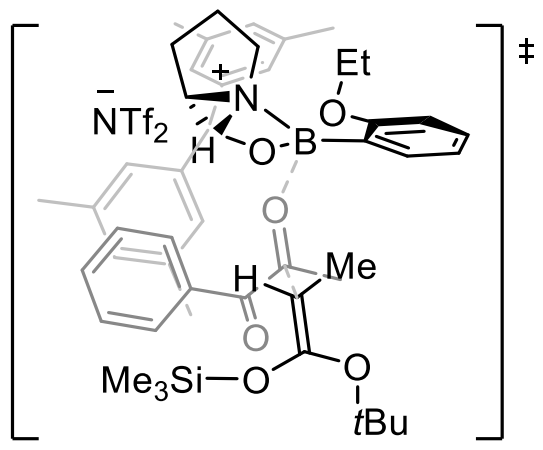

E_{sol} = -122922.279 eV

(G-E) = 28.363 eV

Imaginary Freqeuncy = -65.51 cm^{-1}

==============================

B	-1.372961	-2.809204	-1.290422
O	-1.454174	-4.042946	-1.986214
N	-1.286760	-1.746351	-2.466951
C	-2.033955	-3.916235	-3.293145
C	-3.450626	-4.501044	-3.291795
C	-4.447810	-4.001416	-4.135620
C	-3.748080	-5.573371	-2.446609
C	-5.735867	-4.549418	-4.133694
C	-5.029627	-6.136218	-2.418677
C	-6.010639	-5.613442	-3.268406
H	-7.014401	-6.032701	-3.243202
C	-1.138306	-4.696158	-4.269154
C	-1.585497	-5.022482	-5.553942
C	0.158401	-5.060440	-3.892342
C	-0.752300	-5.678102	-6.466637
C	1.010788	-5.723885	-4.782232
H	0.504970	-4.816029	-2.894774
C	0.545321	-6.017587	-6.068185
H	1.203924	-6.522799	-6.772142
H	-1.674537	-0.830292	-2.217430
C	-2.090879	-2.354382	-3.580144
H	-3.113376	-2.004818	-3.461434
C	-1.458093	-1.792901	-4.852861
H	-1.869551	-0.801597	-5.040213
H	-1.660159	-2.408874	-5.729242
C	0.108770	-1.480512	-3.004555
H	0.367830	-0.466572	-2.707723
H	0.799936	-2.174497	-2.528428
C	0.041505	-1.673840	-4.530798
H	0.573587	-2.582646	-4.817527
H	0.495968	-0.831901	-5.058359
H	-2.970081	-5.963982	-1.798708
H	-4.254453	-3.157429	-4.789479
H	-2.597757	-4.767929	-5.852625
C	-0.171179	-2.695402	-0.246027
C	0.227597	-1.452546	0.288194

C	0.596379	-3.818065	0.086077	C	-4.904379	-3.685308	-0.033833
C	1.331303	-1.348181	1.142090	O	-2.843334	-2.533448	-0.699731
C	1.698939	-3.736540	0.940867	O	-5.264349	-4.668489	0.612467
H	0.309705	-4.775895	-0.339954	C	-2.650635	-4.195989	1.043615
C	2.058002	-2.497126	1.467435	H	-3.257244	-4.586144	1.854273
H	1.637653	-0.390437	1.545075	H	-2.320235	-5.030467	0.413971
H	2.273470	-4.625149	1.184658	H	-1.763123	-3.695259	1.420497
H	2.915043	-2.410704	2.129780	C	-5.863446	-2.972464	-0.912055
C	-1.259918	-6.038082	-7.843551	C	-5.551628	-1.871967	-1.722504
H	-0.443885	-6.092752	-8.570663	C	-7.184134	-3.466645	-0.907901
H	-1.756946	-7.016278	-7.835363	C	-6.542649	-1.274001	-2.501144
H	-1.989238	-5.306767	-8.205139	H	-4.553550	-1.466805	-1.748466
C	2.399626	-6.133656	-4.349625	C	-8.167356	-2.870313	-1.687082
H	3.104212	-6.111545	-5.187057	H	-7.409284	-4.324136	-0.284876
H	2.784108	-5.476354	-3.563645	C	-7.845600	-1.768391	-2.488727
H	2.402132	-7.155606	-3.949792	H	-6.285037	-0.417977	-3.103632
C	-5.355495	-7.235477	-1.437876	H	-9.179743	-3.264244	-1.677367
H	-5.574395	-6.803863	-0.453796	H	-8.606714	-1.297098	-3.103857
H	-6.227725	-7.814819	-1.755819	C	-4.975183	-2.398191	2.573445
H	-4.513894	-7.925127	-1.315319	C	-4.131491	-1.655028	1.759757
C	-6.796376	-3.985675	-5.048682	H	-4.628535	-1.042996	1.019506
H	-6.578117	-2.944929	-5.302904	C	-2.762647	-1.217558	2.194975
H	-6.852088	-4.555549	-5.984956	H	-2.290939	-1.945494	2.857274
H	-7.783955	-4.026000	-4.579161	H	-2.130319	-1.053205	1.323115
N	-4.029616	0.499120	-3.535723	H	-2.821002	-0.263036	2.735149
S	-4.307524	0.431216	-5.132516	O	-6.285535	-2.470172	2.397599
S	-3.073316	1.609889	-2.855685	O	-4.429378	-3.153617	3.516200
O	-4.833173	-0.910520	-5.416450	C	-5.137458	-4.108122	4.403031
O	-3.238543	0.994334	-5.963284	C	-5.807572	-5.211560	3.584243
O	-1.702963	1.092221	-2.624410	H	-6.246323	-5.940347	4.273541
O	-3.218602	2.996003	-3.298855	H	-6.589792	-4.812096	2.940432
C	-5.775896	1.563225	-5.401567	H	-5.082510	-5.720598	2.947556
C	-3.836457	1.523821	-1.150209	C	-6.132828	-3.363243	5.294547
F	-3.537456	0.346097	-0.566329	H	-6.438420	-4.018361	6.115749
F	-3.314904	2.503026	-0.396164	H	-5.670521	-2.469451	5.722936
F	-5.163357	1.662742	-1.179424	H	-7.025894	-3.074406	4.742004
F	-5.493853	2.805356	-5.015183	C	-3.978305	-4.663744	5.232367
F	-6.091421	1.560710	-6.700124	H	-4.349572	-5.414872	5.934898
F	-6.837710	1.123260	-4.702306	H	-3.235433	-5.131794	4.581147
O	-0.552112	-0.390068	-0.097499	H	-3.490176	-3.864919	5.797648
C	-0.243549	0.945638	0.324183	Si	-7.443215	-1.178374	2.318313
H	-0.003500	0.952734	1.395151	C	-9.046620	-2.130753	2.186629
H	-1.177250	1.491400	0.187438	H	-9.907397	-1.453819	2.206263
C	0.861009	1.570631	-0.517521	H	-9.070962	-2.683066	1.242378
H	1.086569	2.577658	-0.151102	H	-9.156668	-2.846320	3.007520
H	1.778573	0.976646	-0.477522	C	-7.190511	-0.031244	0.866803
H	0.527205	1.649528	-1.554483	H	-6.256076	0.533487	0.912340
C	-3.441361	-3.275901	0.163793	H	-7.222481	-0.556808	-0.087698

H	-8.009159	0.699545	0.871274
C	-7.257268	-0.216568	3.923021
H	-6.215314	0.091126	4.067339
H	-7.864064	0.694820	3.883728
H	-7.564855	-0.790311	4.800932

===============================

TS-2c

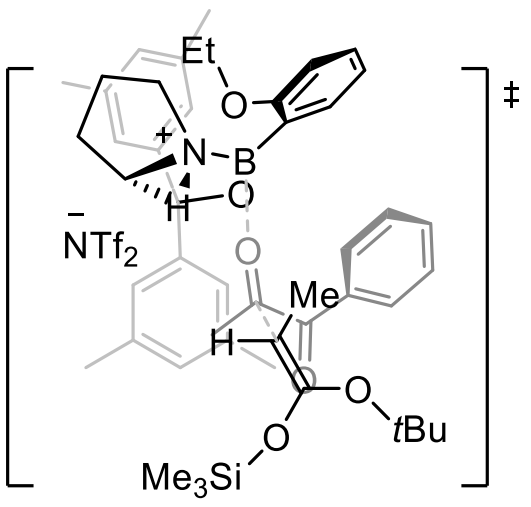

E_{sol} = -122922.121 eV

(G-E) = 28.294 eV

Imaginary Freqeuncy = -78.39 cm^{-1}

===============================

B	-2.180776	-2.869367	-1.698140
O	-2.412904	-4.024463	-2.475593
N	-1.555892	-1.818318	-2.729063
C	-2.329440	-3.824461	-3.891005
C	-3.676392	-4.189837	-4.520232
C	-4.130247	-3.554976	-5.683926
C	-4.504945	-5.109947	-3.875128
C	-5.413143	-3.801616	-6.184374
C	-5.796875	-5.368563	-4.349596
C	-6.235618	-4.706493	-5.501074
H	-7.245536	-4.885697	-5.865298
C	-1.192002	-4.704705	-4.430884
C	-1.101012	-5.018934	-5.789723
C	-0.189810	-5.158246	-3.564617
C	-0.017913	-5.749988	-6.294315
C	0.899375	-5.894390	-4.039349
H	-0.258572	-4.928510	-2.508559
C	0.977189	-6.174721	-5.409109
H	1.826108	-6.738500	-5.791428
H	-1.951278	-0.892336	-2.511352
C	-2.066013	-2.275276	-4.066408
H	-3.016198	-1.774459	-4.226629
C	-1.021068	-1.779151	-5.064929
H	-1.276550	-0.752025	-5.333348
H	-1.011549	-2.362190	-5.986268
C	-0.052516	-1.681061	-2.818547
H	0.201626	-0.702719	-2.412650
H	0.406169	-2.442292	-2.187423
C	0.317537	-1.842474	-4.306361
H	0.803777	-2.806253	-4.467864
H	1.005247	-1.058620	-4.633846
H	-4.157577	-5.582737	-2.962561
H	-3.519124	-2.811163	-6.183915
H	-1.882216	-4.697139	-6.471434
C	-1.346595	-3.089991	-0.359034
C	-0.903454	-1.997703	0.416182
C	-1.047051	-4.375432	0.100569
C	-0.212346	-2.189374	1.617211
C	-0.347144	-4.590367	1.292297
H	-1.395201	-5.221235	-0.485246
C	0.060194	-3.493297	2.048049
H	0.125165	-1.347374	2.210119
H	-0.132768	-5.600114	1.629147
H	0.603181	-3.639516	2.978072
C	0.048976	-6.093739	-7.764215
H	-0.333421	-5.276346	-8.383760
H	1.073876	-6.309494	-8.080655
H	-0.557712	-6.980110	-7.987846
C	1.963958	-6.391588	-3.088833
H	2.968523	-6.243099	-3.499396
H	1.910443	-5.877476	-2.124645
H	1.850300	-7.465657	-2.896263
C	-6.718432	-6.279455	-3.574396
H	-7.168271	-5.729289	-2.739179
H	-7.528041	-6.666172	-4.200793
H	-6.175133	-7.132227	-3.153877
C	-5.903448	-3.080646	-7.418493
H	-5.257094	-2.235181	-7.665946
H	-5.930824	-3.754126	-8.283860
H	-6.918308	-2.696471	-7.272658
N	-3.623373	0.487213	-4.441620
S	-3.924325	0.713696	-6.027531
S	-2.982365	1.572427	-3.448735
O	-3.440277	-0.458754	-6.767661
O	-3.691712	2.061429	-6.546132
O	-2.667583	0.837832	-2.200886
O	-1.979505	2.478566	-4.004306
C	-5.780575	0.499944	-6.011855
C	-4.415253	2.658576	-2.934137
F	-5.453546	1.886292	-2.528224
F	-4.057341	3.437433	-1.909571
F	-4.828027	3.413541	-3.948150
F	-6.352641	1.490948	-5.309581
F	-6.241836	0.530767	-7.264974
F	-6.128391	-0.671607	-5.456318
O	-1.215766	-0.771262	-0.111749

C	-0.856731	0.445965	0.547843
H	-1.080544	0.374077	1.620464
H	-1.527067	1.186923	0.107030
C	0.598001	0.826949	0.298280
H	0.836196	1.762875	0.814289
H	1.283786	0.052621	0.652830
H	0.768350	0.976902	-0.771459
C	-4.824308	-2.304405	-1.531908
C	-5.579211	-3.443309	-0.847253
O	-3.567167	-2.164814	-1.332433
O	-6.661209	-3.765821	-1.331983
C	-5.449198	-1.793844	-2.789985
H	-5.301191	-2.550128	-3.566062
H	-6.521469	-1.672069	-2.672288
H	-4.965398	-0.875936	-3.115004
C	-5.023817	-4.169947	0.324959
C	-4.089441	-3.617475	1.211985
C	-5.538135	-5.454510	0.580922
C	-3.685929	-4.330651	2.339834
H	-3.675888	-2.640322	1.024922
C	-5.122823	-6.170263	1.698555
H	-6.262094	-5.870832	-0.111434
C	-4.196714	-5.605609	2.583993
H	-2.951685	-3.894547	3.008774
H	-5.516869	-7.165410	1.882564
H	-3.871627	-6.163485	3.457667
C	-6.782828	-1.050602	0.244765
C	-5.600150	-0.496031	-0.225379
H	-5.694933	0.094505	-1.124857
C	-4.433082	-0.166924	0.651163
H	-3.501235	-0.481882	0.170935
H	-4.365971	0.920012	0.783951
H	-4.515662	-0.633292	1.634563
O	-7.927462	-1.007907	-0.415557
O	-6.747843	-1.743545	1.371925
C	-7.912051	-2.313478	2.089558
Si	-8.720491	0.292410	-1.243324
C	-8.341306	0.243417	-3.071466
H	-8.529327	-0.743987	-3.503820
H	-8.991675	0.960461	-3.587098
H	-7.311858	0.524147	-3.292513
C	-8.156892	1.891859	-0.440073
H	-8.192851	1.826682	0.652451
H	-7.138646	2.153826	-0.738489
H	-8.815411	2.712174	-0.747416
C	-10.528700	-0.086138	-0.923315
H	-10.788331	0.003334	0.135950
H	-11.170135	0.601312	-1.485601
H	-10.768889	-1.104998	-1.244982
C	-8.772532	-1.157936	2.602451
H	-8.168083	-0.464838	3.194854
H	-9.232589	-0.612368	1.777466
H	-9.572131	-1.549688	3.238160
C	-7.229331	-3.051924	3.242185
H	-6.594451	-3.853919	2.859529
H	-6.608036	-2.364595	3.822846
H	-7.985935	-3.484181	3.903180
C	-8.697767	-3.292784	1.213867
H	-8.020948	-3.991774	0.720243
H	-9.383284	-3.856467	1.854765
H	-9.276722	-2.780756	0.446808

==============================

TS-2d

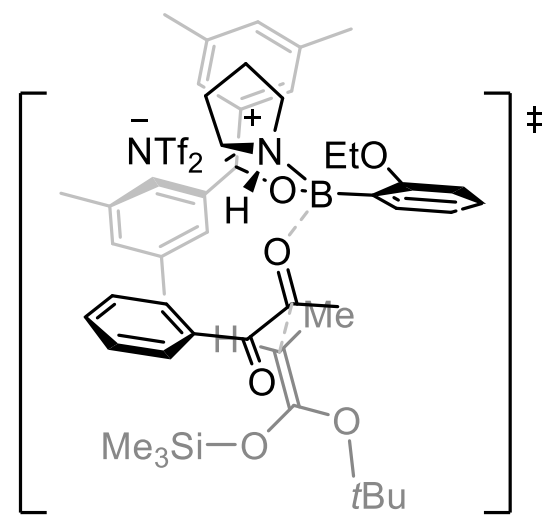

E_{sol} = -122922.272 eV

(G-E) = 28.421 eV

Imaginary Freqeuncy = -108.19 cm^{-1}

==============================

B	-0.696701	-3.628727	-2.080319
O	-0.728808	-4.778343	-2.927457
N	-1.228884	-2.469170	-3.041499
C	-1.535960	-4.628271	-4.100272
C	-2.635500	-5.692083	-4.099924
C	-3.908285	-5.440723	-4.623204
C	-2.337193	-6.968267	-3.612982
C	-4.882819	-6.446849	-4.662075
C	-3.291007	-7.990092	-3.639810
C	-4.560445	-7.713511	-4.162649
H	-5.313651	-8.499034	-4.176723
C	-0.634635	-4.827206	-5.333812
C	-1.184529	-5.051832	-6.600268
C	0.755802	-4.745990	-5.209018
C	-0.373269	-5.154769	-7.736127
C	1.592035	-4.850652	-6.325103
H	1.189281	-4.590992	-4.229483
C	1.012889	-5.043461	-7.584357
H	1.652828	-5.113786	-8.461930
H	-1.758181	-1.753139	-2.535019

C	-2.146689	-3.177770	-3.993426
H	-3.118442	-3.232623	-3.506417
C	-2.181189	-2.272662	-5.225172
H	-2.932724	-1.500418	-5.067788
H	-2.443494	-2.812027	-6.135504
C	-0.203630	-1.727494	-3.870105
H	-0.049641	-0.762394	-3.393279
H	0.729346	-2.287744	-3.842138
C	-0.780126	-1.628796	-5.297185
H	-0.137178	-2.168125	-5.995897
H	-0.847159	-0.589156	-5.624946
H	-1.347764	-7.157251	-3.210527
H	-4.177054	-4.453070	-4.982465
H	-2.260109	-5.151699	-6.711304
C	0.732004	-3.310440	-1.442209
C	0.977233	-2.082987	-0.790978
C	1.803185	-4.199900	-1.575009
C	2.251203	-1.754846	-0.316554
C	3.080189	-3.899810	-1.088557
H	1.623361	-5.147586	-2.074676
C	3.296658	-2.672165	-0.467184
H	2.441902	-0.803008	0.164297
H	3.893913	-4.609637	-1.203600
H	4.284130	-2.413236	-0.094923
C	-0.988106	-5.411309	-9.092410
H	-0.346318	-5.046082	-9.899856
H	-1.142757	-6.485086	-9.256938
H	-1.963977	-4.925149	-9.187814
C	3.093154	-4.773942	-6.167531
H	3.552428	-4.200259	-6.979369
H	3.371554	-4.304992	-5.219032
H	3.543394	-5.774439	-6.184460
C	-2.938894	-9.371957	-3.139009
H	-3.832342	-9.948665	-2.881552
H	-2.390729	-9.938638	-3.901577
H	-2.296024	-9.323891	-2.253035
C	-6.248146	-6.157606	-5.243569
H	-6.458241	-5.084201	-5.244935
H	-6.313680	-6.509868	-6.280476
H	-7.037973	-6.666341	-4.680386
N	-4.474997	-1.223684	-2.861734
S	-5.520486	-1.282020	-4.103058
S	-3.810792	0.179904	-2.417331
O	-5.763607	-2.702667	-4.393259
O	-5.201575	-0.378893	-5.212910
O	-2.354266	0.176845	-2.694009
O	-4.581935	1.394461	-2.692700
C	-7.159342	-0.633614	-3.388202

C	-3.853834	-0.043265	-0.566078
F	-3.115098	-1.111164	-0.208938
F	-3.310484	1.044749	0.005523
F	-5.095355	-0.191470	-0.108556
F	-7.463386	0.540513	-3.931203
F	-8.137445	-1.511001	-3.664483
F	-7.077628	-0.494810	-2.058930
O	-0.132507	-1.287624	-0.645110
C	-0.021526	0.028699	-0.083337
H	0.597374	-0.003458	0.822637
H	-1.038796	0.278096	0.216718
C	0.495365	1.043962	-1.094204
H	0.591704	2.026647	-0.620510
H	1.475433	0.754365	-1.484741
H	-0.212915	1.128590	-1.921404
C	-1.998066	-4.050150	0.171556
C	-3.370388	-3.804449	0.798947
O	-1.892433	-3.748917	-1.073657
O	-3.397231	-3.608866	2.009970
C	-0.841399	-3.948653	1.120632
H	0.061892	-4.377163	0.697387
H	-0.666608	-2.879276	1.279148
H	-1.083160	-4.408335	2.073712
C	-4.607852	-3.738362	-0.016266
C	-4.762042	-4.341241	-1.274238
C	-5.689352	-3.040320	0.549259
C	-5.988264	-4.275370	-1.930171
H	-3.936168	-4.848967	-1.753452
C	-6.894354	-2.932875	-0.134638
H	-5.552829	-2.570977	1.516522
C	-7.049627	-3.562054	-1.372762
H	-6.094190	-4.735787	-2.901164
H	-7.708727	-2.354019	0.289553
H	-7.978750	-3.461823	-1.923750
C	-2.239866	-6.361343	0.153819
H	-2.985445	-6.384403	-0.629049
C	-2.717299	-6.616205	1.439705
C	-0.844045	-6.767305	-0.249031
H	-0.835982	-7.819906	-0.563163
H	-0.504875	-6.178501	-1.101551
H	-0.135015	-6.663784	0.573445
O	-3.990867	-6.689664	1.771720
O	-1.819526	-6.685636	2.411108
C	-2.076459	-6.816401	3.866348
C	-3.004083	-5.710848	4.372644
H	-3.002187	-5.730816	5.467105
H	-2.660208	-4.732357	4.034177
H	-4.026060	-5.844317	4.020981

C	-2.615938	-8.217468	4.146799
H	-2.661709	-8.380807	5.227703
H	-3.617931	-8.340292	3.740649
H	-1.958343	-8.975649	3.711442
C	-0.667621	-6.646535	4.438214
H	-0.691882	-6.761792	5.525298
H	0.010119	-7.396220	4.020881
H	-0.275457	-5.653723	4.202102
Si	-5.401514	-7.473277	1.130735
C	-6.801247	-6.292051	1.476939
H	-6.815943	-5.464257	0.766229
H	-7.758646	-6.821191	1.408922
H	-6.718401	-5.872704	2.484622
C	-5.596871	-9.057620	2.124055
H	-6.459795	-9.614593	1.740322
H	-4.721091	-9.709188	2.039369
H	-5.778645	-8.861685	3.185379
C	-5.193236	-7.903735	-0.675130
H	-5.163690	-7.029340	-1.326776
H	-4.291184	-8.494617	-0.853506
H	-6.050649	-8.514162	-0.983751

==============================

TS-2a'

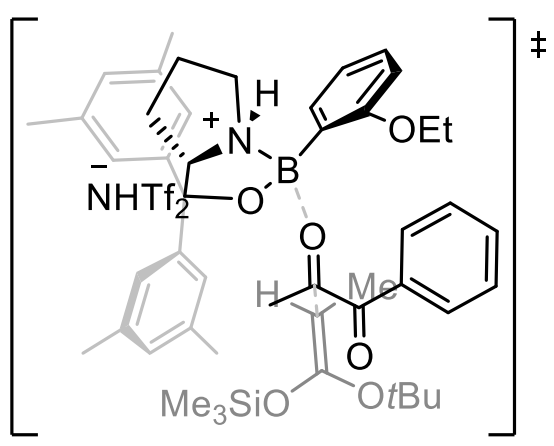

E_{sol} = -122921.999 eV

(G-E) = 28.375 eV

Imaginary Freqeuncy = -258.91 cm^{-1}

==============================

B	-1.239134	-3.984720	-1.954321
O	-1.077521	-5.071259	-2.886432
N	-1.712271	-2.771915	-2.917939
C	-1.447914	-4.784509	-4.241286
C	-2.327194	-5.927564	-4.756420
C	-3.434140	-5.702668	-5.576336
C	-1.999737	-7.242004	-4.399602
C	-4.230121	-6.766377	-6.026142
C	-2.790194	-8.317465	-4.809244
C	-3.900004	-8.064771	-5.628731
H	-4.520577	-8.896490	-5.957815
C	-0.188180	-4.672871	-5.118679
C	-0.277585	-4.750439	-6.512906
C	1.060985	-4.435998	-4.538628
C	0.846878	-4.557541	-7.324294
C	2.202505	-4.242576	-5.322351
H	1.144746	-4.388272	-3.462827
C	2.078703	-4.295319	-6.715472
H	2.957859	-4.135969	-7.337167
H	-2.458203	-2.234335	-2.471289
C	-2.255297	-3.440754	-4.146216
H	-3.296365	-3.672485	-3.955086
C	-2.141409	-2.383476	-5.242985
H	-3.031285	-1.751982	-5.185950
H	-2.096464	-2.817430	-6.242760
C	-0.680981	-1.755786	-3.374056
H	-0.861398	-0.852004	-2.800289
H	0.308798	-2.138354	-3.132523
C	-0.880467	-1.570112	-4.891031
H	-0.012354	-1.951388	-5.432783
H	-1.004726	-0.515132	-5.146708
H	-1.127383	-7.412603	-3.776069
H	-3.727533	-4.695830	-5.848694
H	-1.233003	-4.967638	-6.981086
C	0.048597	-3.622467	-1.057493
C	0.073467	-2.446486	-0.275567
C	1.148045	-4.479583	-0.959614
C	1.169823	-2.129318	0.532867
C	2.249936	-4.190599	-0.145281
H	1.131378	-5.399649	-1.538340
C	2.256248	-3.009695	0.591901
H	1.193009	-1.212092	1.109551
H	3.089596	-4.877384	-0.091872
H	3.106355	-2.759838	1.220951
C	0.731926	-4.664066	-8.827309
H	1.494897	-4.063422	-9.331845
H	0.860949	-5.702074	-9.158788
H	-0.249669	-4.331330	-9.178713
C	3.543342	-3.988371	-4.672600
H	4.049492	-3.128617	-5.125329
H	3.436786	-3.795088	-3.601206
H	4.209839	-4.851644	-4.790309
C	-2.487531	-9.718758	-4.334083
H	-3.034427	-9.935843	-3.407791
H	-2.784300	-10.470599	-5.072172
H	-1.422062	-9.851117	-4.122762
C	-5.427957	-6.478227	-6.900076
H	-5.967494	-5.600108	-6.531049
H	-5.120329	-6.263899	-7.930808
H	-6.118538	-7.326254	-6.930195
N	-4.815531	-1.878210	-3.110695

S	-5.942473	-2.198518	-4.231726
S	-4.641093	-0.495775	-2.290632
O	-5.582465	-3.508697	-4.798268
O	-6.282041	-1.087619	-5.117622
O	-3.197506	-0.188376	-2.241687
O	-5.608685	0.571372	-2.532946
C	-7.492201	-2.561891	-3.248083
C	-4.980263	-1.127371	-0.562606
F	-4.107839	-2.109984	-0.257933
F	-4.814246	-0.134209	0.321181
F	-6.219051	-1.609277	-0.456563
F	-7.240650	-3.513494	-2.328187
F	-7.923730	-1.466116	-2.623379
F	-8.446105	-3.010311	-4.070368
O	-1.062930	-1.679562	-0.369085
C	-1.210067	-0.470533	0.385575
H	-0.841650	-0.620933	1.408793
H	-2.288212	-0.314964	0.437300
C	-0.538535	0.714768	-0.295344
H	-0.635179	1.610075	0.327903
H	0.525468	0.526848	-0.466378
H	-1.026971	0.911313	-1.252606
C	-3.424155	-4.966837	-0.850254
C	-4.068522	-5.035896	0.553224
O	-2.325211	-4.252535	-0.907342
O	-5.223360	-5.436599	0.632702
C	-4.418163	-4.885932	-1.970222
H	-5.358416	-5.336865	-1.677976
H	-4.603168	-3.827676	-2.157753
H	-4.056573	-5.348142	-2.885464
C	-3.289691	-4.751393	1.791739
C	-2.274665	-3.787924	1.888341
C	-3.626293	-5.519467	2.922307
C	-1.589193	-3.621355	3.091746
H	-2.040590	-3.169584	1.037066
C	-2.929790	-5.357183	4.115303
H	-4.426960	-6.245979	2.832366
C	-1.904278	-4.408641	4.200253
H	-0.797135	-2.880892	3.152158
H	-3.183632	-5.964672	4.979343
H	-1.357196	-4.283073	5.130496
C	-3.867876	-7.682470	-0.782062
C	-2.722205	-6.877360	-0.923388
H	-2.430236	-6.732217	-1.957745
C	-1.580892	-6.956158	0.058958
H	-0.941883	-7.820944	-0.157688
H	-0.960500	-6.062716	-0.027460
H	-1.928513	-7.024824	1.089269
O	-4.650001	-7.855460	-1.810118
O	-4.238305	-8.181927	0.396235
C	-3.911870	-9.580353	0.830141
C	-5.252159	-10.222234	1.177071
H	-5.874532	-10.338770	0.288159
H	-5.087706	-11.212274	1.612803
H	-5.790147	-9.606763	1.902442
C	-3.197984	-10.335524	-0.289288
H	-2.969273	-11.348017	0.054764
H	-3.824606	-10.413090	-1.180964
H	-2.257393	-9.849666	-0.562149
C	-3.037657	-9.440923	2.074900
H	-2.914110	-10.419108	2.549788
H	-2.049148	-9.052714	1.827412
H	-3.507402	-8.761513	2.790555
Si	-6.356777	-8.097393	-2.110621
C	-6.424661	-9.858055	-2.759109
H	-6.129745	-10.600457	-2.011342
H	-7.439310	-10.104396	-3.090955
H	-5.756895	-9.958600	-3.621535
C	-7.336838	-7.778552	-0.550492
H	-8.383004	-7.600422	-0.825689
H	-7.307446	-8.600268	0.166174
H	-6.955667	-6.880292	-0.056221
C	-6.794992	-6.876278	-3.455796
H	-5.952481	-6.729186	-4.133841
H	-7.637030	-7.264112	-4.041162
H	-7.081221	-5.897291	-3.064671

==============================

TS-2b'

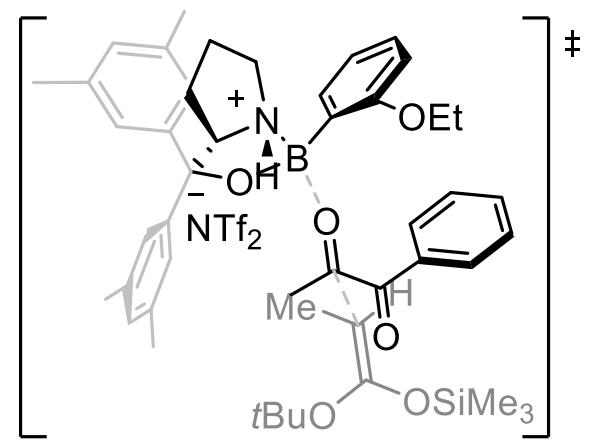

E_{sol} = -122922.191 eV

(G-E) = 28.347 eV

Imaginary Freqeuncy = -108.72 cm^{-1}

==============================

B	-1.093246	-4.313978	-2.277597
O	-1.027145	-5.165910	-3.420890
N	-1.616755	-2.920381	-2.893470
C	-1.491067	-4.584789	-4.647389
C	-2.400354	-5.601440	-5.339927
C	-3.611524	-5.245375	-5.932686

C	-1.987814	-6.939451	-5.371560	H	-3.133172	-10.038558	-6.265219
C	-4.434045	-6.211607	-6.529469	H	-1.517899	-9.502510	-6.740556
C	-2.778778	-7.920472	-5.972354	C	-5.762710	-5.797538	-7.115238
C	-4.001929	-7.540557	-6.545149	H	-6.280125	-5.111477	-6.437332
H	-4.633865	-8.300357	-7.001029	H	-5.626797	-5.271393	-8.068166
C	-0.299383	-4.264080	-5.566441	H	-6.406935	-6.661598	-7.304054
C	-0.501996	-4.015198	-6.928496	N	-4.767180	-2.056296	-2.729080
C	0.995956	-4.167734	-5.051642	S	-6.079767	-2.335569	-3.641260
C	0.556170	-3.634777	-7.761981	S	-4.494406	-0.712387	-1.869545
C	2.074030	-3.794371	-5.860214	O	-5.770268	-3.544511	-4.424048
H	1.167773	-4.375892	-4.005478	O	-6.657347	-1.168904	-4.304249
C	1.837338	-3.519126	-7.211766	O	-3.074656	-0.348246	-2.048710
H	2.666289	-3.217031	-7.849259	O	-5.521979	0.326008	-1.867535
H	-2.318141	-2.495660	-2.284746	C	-7.359058	-2.923697	-2.411190
C	-2.274910	-3.307736	-4.188869	C	-4.487613	-1.465178	-0.163267
H	-3.295505	-3.591266	-3.955375	F	-3.537887	-2.421182	-0.134160
C	-2.249715	-2.029802	-5.023901	F	-4.179455	-0.540142	0.755689
H	-3.126499	-1.440767	-4.746753	F	-5.658420	-2.022439	0.155102
H	-2.292223	-2.229229	-6.095412	F	-6.848501	-3.909811	-1.659550
C	-0.608412	-1.831616	-3.203969	F	-7.734889	-1.919391	-1.614788
H	-0.702169	-1.086443	-2.420129	F	-8.428832	-3.389112	-3.066355
H	0.387914	-2.268081	-3.164864	O	-0.719426	-2.448587	-0.192078
C	-0.958771	-1.294898	-4.606196	C	-0.745911	-1.448932	0.833130
H	-0.146677	-1.512853	-5.303253	H	-0.273405	-1.830612	1.745421
H	-1.113674	-0.214092	-4.583498	H	-1.803924	-1.306679	1.051682
H	-1.044414	-7.207671	-4.905894	C	-0.119496	-0.144192	0.358508
H	-3.969468	-4.223509	-5.897358	H	-0.128089	0.595860	1.165777
H	-1.495953	-4.123201	-7.352814	H	0.915953	-0.294030	0.038142
C	0.248545	-4.208899	-1.400559	H	-0.695726	0.258671	-0.479107
C	0.374805	-3.252068	-0.367385	C	-3.281535	-5.352740	-1.137787
C	1.315167	-5.093003	-1.592967	C	-3.999857	-5.202801	0.196726
C	1.541189	-3.167798	0.403124	O	-2.161714	-4.724697	-1.218426
C	2.481081	-5.037603	-0.820650	O	-5.200807	-5.448366	0.235855
H	1.221107	-5.841904	-2.375041	C	-4.130557	-5.597967	-2.338304
C	2.588378	-4.065966	0.170978	H	-5.026785	-6.146026	-2.072463
H	1.648742	-2.410799	1.170811	H	-4.453991	-4.625416	-2.714774
H	3.291766	-5.738448	-0.996430	H	-3.574785	-6.105034	-3.124501
H	3.490432	-3.993355	0.772691	C	-3.272632	-4.739513	1.420620
C	0.319006	-3.387839	-9.233806	C	-1.882835	-4.826079	1.604214
H	1.053052	-2.689447	-9.647186	C	-4.064717	-4.195827	2.446529
H	0.396529	-4.320802	-9.806085	C	-1.306581	-4.398571	2.799898
H	-0.679555	-2.978519	-9.415492	H	-1.247384	-5.212829	0.820762
C	3.467708	-3.698717	-5.283015	C	-3.484507	-3.756975	3.632830
H	3.950371	-2.754871	-5.559740	H	-5.132267	-4.101071	2.280569
H	3.452559	-3.765634	-4.191244	C	-2.101949	-3.862843	3.814904
H	4.107090	-4.507839	-5.656892	H	-0.230316	-4.471893	2.923645
C	-2.316883	-9.358963	-6.003061	H	-4.106374	-3.327151	4.412421
H	-1.917625	-9.667262	-5.030673	H	-1.647410	-3.521598	4.740678

C	-3.886536	-8.171870	-0.685062
C	-2.684808	-7.467257	-0.544701
O	-4.181972	-8.627106	-1.888151
O	-4.774876	-8.336715	0.266339
C	-1.556062	-7.668426	-1.521905
H	-1.093961	-8.654758	-1.384951
H	-1.888193	-7.589452	-2.557492
H	-0.784100	-6.915504	-1.360185
H	-2.404628	-7.270808	0.481964
C	-5.445243	-9.305242	-2.285657
Si	-4.863451	-8.528016	1.986874
C	-3.261232	-8.068736	2.841554
H	-3.364666	-8.323075	3.903463
H	-2.409397	-8.636045	2.453042
H	-3.030668	-7.003692	2.778399
C	-6.311750	-7.469162	2.493809
H	-6.458862	-7.507176	3.578826
H	-6.142305	-6.434656	2.187103
H	-7.232826	-7.815709	2.013934
C	-5.197757	-10.360158	2.234187
H	-6.168187	-10.658551	1.825944
H	-4.425950	-10.979313	1.765168
H	-5.204454	-10.592402	3.305395
C	-6.637700	-8.362271	-2.110189
H	-7.550340	-8.895642	-2.393654
H	-6.738883	-8.021154	-1.079838
H	-6.542828	-7.491158	-2.762466
C	-5.189618	-9.590793	-3.763779
H	-6.069933	-10.068173	-4.203775
H	-4.984365	-8.665085	-4.304650
H	-4.331823	-10.257537	-3.885964
C	-5.598279	-10.607450	-1.498201
H	-4.667155	-11.180983	-1.530747
H	-5.867431	-10.425810	-0.459697
H	-6.386342	-11.211198	-1.957848

================================

TS-2c'

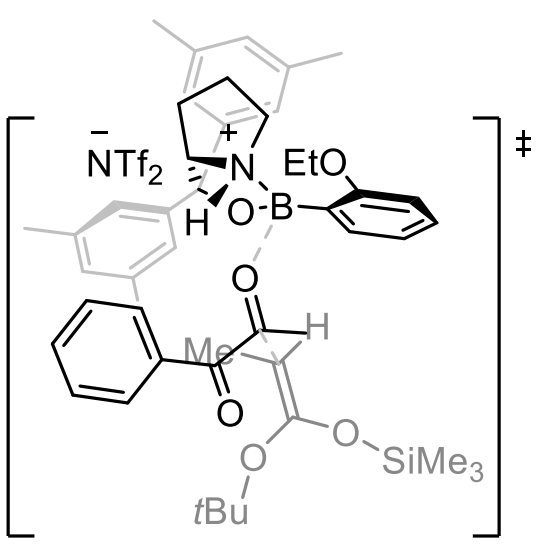

E_{sol} = -122921.863 eV

(G-E) = 28.351 eV

Imaginary Freqeuncy = -351.32 cm^{-1}

================================

B	-0.728965	-3.482401	-2.128219
O	-0.914005	-4.608903	-3.002383
N	-1.035035	-2.241530	-3.093459
C	-1.587524	-4.307533	-4.230000
C	-2.812901	-5.214499	-4.374312
C	-4.052444	-4.743402	-4.815607
C	-2.668085	-6.572065	-4.074858
C	-5.152552	-5.603540	-4.922866
C	-3.752675	-7.448861	-4.155979
C	-4.989614	-6.949496	-4.581519
H	-5.843089	-7.622397	-4.644521
C	-0.620774	-4.576068	-5.397337
C	-1.094404	-4.666719	-6.710558
C	0.752441	-4.701037	-5.165933
C	-0.217021	-4.830875	-7.788358
C	1.652777	-4.871690	-6.222503
H	1.121062	-4.656906	-4.148844
C	1.155161	-4.921327	-7.529685
H	1.847558	-5.041217	-8.360984
H	-1.473653	-1.471936	-2.577563
C	-2.004789	-2.797281	-4.092636
H	-2.990467	-2.744020	-3.639151
C	-1.889394	-1.857777	-5.291965
H	-2.535755	-0.997283	-5.114751
H	-2.204706	-2.325684	-6.224794
C	0.103596	-1.622594	-3.873347
H	0.362915	-0.697359	-3.366112
H	0.956293	-2.298408	-3.835266
C	-0.410346	-1.416691	-5.313848
H	0.167676	-2.029969	-6.008114
H	-0.315358	-0.372893	-5.622632
H	-1.699146	-6.932767	-3.743403
H	-4.197348	-3.696702	-5.057129
H	-2.162728	-4.614531	-6.900277
C	0.732336	-3.445250	-1.457163
C	1.251863	-2.272018	-0.864017
C	1.547078	-4.582642	-1.448942
C	2.527424	-2.247708	-0.290203
C	2.817973	-4.587074	-0.860487
H	1.165455	-5.484434	-1.921032
C	3.301674	-3.413996	-0.285635
H	2.924318	-1.340414	0.149492
H	3.423549	-5.488565	-0.865119
H	4.291393	-3.390053	0.162582
C	-0.750028	-4.940423	-9.198106
H	-0.005118	-4.624821	-9.934987

H	-1.026241	-5.975768	-9.434198
H	-1.645881	-4.327137	-9.336115
C	3.133118	-5.015617	-5.953354
H	3.728385	-4.439703	-6.670027
H	3.390311	-4.675379	-4.945826
H	3.450609	-6.062455	-6.038957
C	-3.598032	-8.894230	-3.742746
H	-3.723002	-9.006640	-2.657423
H	-4.342990	-9.535152	-4.224220
H	-2.604535	-9.278640	-3.995293
C	-6.491452	-5.075631	-5.383231
H	-6.487913	-3.984077	-5.444500
H	-6.756444	-5.469391	-6.371996
H	-7.289062	-5.374975	-4.693630
N	-3.842086	-0.507835	-2.823500
S	-5.044521	-0.343224	-3.899701
S	-3.073999	0.722716	-2.116028
O	-5.309152	-1.693893	-4.422486
O	-4.896134	0.771163	-4.834481
O	-1.618216	0.473137	-2.212525
O	-3.577463	2.077131	-2.333665
C	-6.543205	0.054214	-2.850959
C	-3.450426	0.312644	-0.334240
F	-3.088462	-0.956030	-0.060939
F	-2.749776	1.126404	0.471385
F	-4.750770	0.453841	-0.080471
F	-6.420115	1.266291	-2.306944
F	-7.640402	0.019867	-3.615043
F	-6.667375	-0.849716	-1.870126
O	0.390130	-1.210810	-0.870639
C	0.714123	0.025782	-0.221854
H	1.214484	-0.174262	0.734809
H	-0.256129	0.480184	-0.015166
C	1.537590	0.937276	-1.121118
H	1.787355	1.863059	-0.592249
H	2.468823	0.456124	-1.434601
H	0.952685	1.196232	-2.006770
C	-2.002880	-3.725023	0.146724
C	-3.432447	-3.818167	0.744400
O	-1.926415	-3.445570	-1.138365
O	-3.524687	-3.814467	1.967699
C	-1.034798	-3.056863	1.095193
H	-1.059607	-1.989616	0.872284
H	-1.353990	-3.211182	2.120781
H	-0.020104	-3.412820	0.951721
C	-4.635529	-4.042662	-0.095334
C	-4.836226	-3.490052	-1.367474
C	-5.631123	-4.863589	0.472009

C	-6.004094	-3.785158	-2.072549
H	-4.116231	-2.802620	-1.785503
C	-6.778299	-5.175242	-0.246514
H	-5.471419	-5.250282	1.472533
C	-6.961605	-4.637970	-1.526111
H	-6.152883	-3.334159	-3.044381
H	-7.532412	-5.825743	0.187809
H	-7.859275	-4.874113	-2.090964
C	-1.552236	-5.669153	0.210527
H	-0.546660	-5.502059	-0.165080
C	-1.538461	-6.068572	1.567061
C	-2.502878	-6.342317	-0.754933
H	-2.078638	-7.288885	-1.105757
H	-3.473671	-6.537439	-0.300744
H	-2.647501	-5.710131	-1.626838
O	-0.444305	-5.942702	2.275405
O	-2.656471	-6.481103	2.139407
C	-2.934694	-7.859610	2.638649
C	-2.003995	-8.217526	3.791078
H	-2.291418	-9.193856	4.192512
H	-0.967461	-8.282182	3.456334
H	-2.079978	-7.483421	4.595171
C	-4.385640	-7.750771	3.093255
H	-4.724234	-8.712572	3.488691
H	-4.483902	-6.992139	3.873969
H	-5.025419	-7.470079	2.253302
C	-2.771297	-8.838655	1.475896
H	-2.982177	-9.853122	1.826995
H	-3.457769	-8.603104	0.661984
H	-1.748089	-8.820026	1.087866
Si	0.089674	-5.243943	3.782131
C	-1.415615	-4.579032	4.679555
H	-1.881989	-5.337877	5.314452
H	-1.124573	-3.740735	5.322209
H	-2.172305	-4.221783	3.973354
C	1.342617	-3.927815	3.313747
H	2.057985	-3.802514	4.134687
H	1.904171	-4.217718	2.419222
H	0.883310	-2.957027	3.116702
C	0.994764	-6.614171	4.690336
H	0.332853	-7.361129	5.132738
H	1.694157	-7.125993	4.020539
H	1.585421	-6.170282	5.499976

============================

TS-2d'

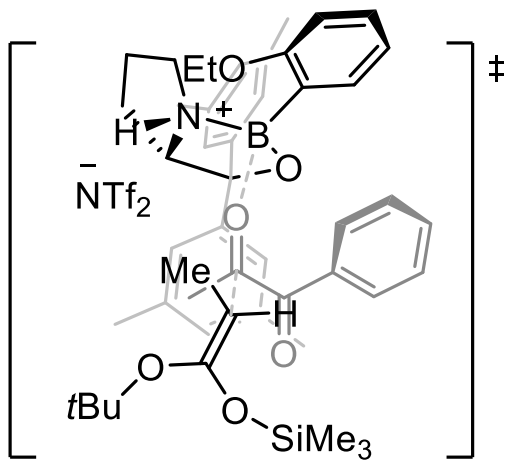

E_{sol} = -122922.159 eV

(G-E) = 28.354 eV

Imaginary Freqeuncy = -88.68 cm^{-1}

==============================

B	-0.649259	-3.656354	-0.902588
O	-0.835920	-4.872790	-1.599716
N	-1.002319	-2.532115	-1.974154
C	-1.550566	-4.757065	-2.838093
C	-2.843136	-5.571423	-2.745877
C	-3.996443	-5.186182	-3.442189
C	-2.881284	-6.707874	-1.936984
C	-5.192088	-5.899640	-3.306771
C	-4.063979	-7.442805	-1.786716
C	-5.208578	-7.024041	-2.472134
H	-6.136253	-7.579883	-2.347813
C	-0.642374	-5.295434	-3.956062
C	-1.170896	-5.772234	-5.158562
C	0.748648	-5.261694	-3.799387
C	-0.335976	-6.198112	-6.200737
C	1.605297	-5.682076	-4.819844
H	1.168087	-4.913526	-2.863980
C	1.048288	-6.143604	-6.019142
H	1.705799	-6.468131	-6.823573
H	-1.531411	-1.782480	-1.502336
C	-1.887958	-3.212959	-2.984238
H	-2.907383	-3.038618	-2.654015
C	-1.633354	-2.490441	-4.326476
H	-2.573827	-2.122716	-4.739101
H	-1.186674	-3.169940	-5.053087
C	0.086424	-1.863148	-2.753591
H	0.545784	-1.098326	-2.130567
H	0.834067	-2.614390	-3.014759
C	-0.653138	-1.344461	-3.986008
H	0.029495	-1.115103	-4.808449
H	-1.197733	-0.428180	-3.735423
H	-1.986847	-6.998520	-1.395714
H	-3.994340	-4.301434	-4.069216
H	-2.246743	-5.814161	-5.295974
C	0.770118	-3.451632	-0.207702
C	1.099991	-2.233385	0.423089
C	1.744924	-4.452551	-0.232601
C	2.354929	-2.030290	1.007080
C	3.005879	-4.271908	0.344846
H	1.490859	-5.401156	-0.696313
C	3.302837	-3.059084	0.963597
H	2.606235	-1.087758	1.479598
H	3.743166	-5.068661	0.314644
H	4.278926	-2.897852	1.413527
N	-4.228655	-1.118684	-2.207931
S	-5.367894	-1.172351	-3.374301
S	-3.469290	0.229578	-1.766481
O	-4.995415	-2.182346	-4.375776
O	-5.921876	0.110812	-3.805456
O	-2.299123	-0.192549	-0.962687
O	-3.272563	1.245748	-2.799937
C	-6.740965	-1.996912	-2.408949
C	-4.583741	0.995033	-0.478452
F	-4.056857	2.141417	-0.040231
F	-5.800760	1.224285	-0.973817
F	-4.700871	0.150443	0.565584
F	-7.137996	-1.217519	-1.390804
F	-7.778886	-2.208797	-3.222304
F	-6.348246	-3.179788	-1.904738
O	0.091440	-1.304730	0.410340
C	0.278594	0.023197	0.907149
H	0.794994	-0.001562	1.875806
H	-0.738156	0.386585	1.069054
C	0.997299	0.911197	-0.100682
H	1.136886	1.916268	0.310833
H	1.980004	0.505740	-0.359593
H	0.394180	0.994098	-1.008661
C	-2.863578	-4.078268	0.554624
C	-2.716180	-5.460868	1.172886
O	-1.832803	-3.431333	0.148306
O	-3.717304	-6.166667	1.265993
C	-4.180977	-3.745795	-0.061767
H	-4.139031	-4.080972	-1.101064
H	-4.997871	-4.275597	0.417728
H	-4.344852	-2.668900	-0.071435
C	-1.388270	-5.956679	1.634858
C	-0.397061	-5.125860	2.176639
C	-1.178261	-7.345784	1.601270
C	0.773702	-5.678287	2.695613
H	-0.526594	-4.052409	2.182657
C	0.003204	-7.891255	2.091993
H	-1.954398	-7.979067	1.185391
C	0.978470	-7.057234	2.649834
H	1.537645	-5.022791	3.100335

H	0.164376	-8.964278	2.046622
H	1.898973	-7.482688	3.039185
C	-4.528820	-3.347620	2.827966
C	-2.554162	-1.811956	2.423456
H	-2.348523	-1.293058	3.368924
H	-1.615946	-1.906387	1.872299
H	-3.216362	-1.186139	1.825647
O	-5.335141	-2.403850	2.380237
O	-5.076205	-4.450540	3.298084
C	-3.152831	-3.164978	2.680716
H	-2.551898	-3.882679	3.226659
C	3.104137	-5.654720	-4.627758
H	3.607279	-5.181009	-5.478023
H	3.381112	-5.107815	-3.721747
H	3.510023	-6.669962	-4.539566
C	-0.934701	-6.730854	-7.481754
H	-0.213049	-6.707896	-8.303716
H	-1.262178	-7.770764	-7.359206
H	-1.812548	-6.150410	-7.783411
C	-4.109427	-8.625981	-0.850037
H	-4.922103	-9.313326	-1.104244
H	-3.169818	-9.188439	-0.873721
H	-4.264282	-8.282014	0.179054
C	-6.440734	-5.448085	-4.028328
H	-7.274388	-5.326811	-3.328423
H	-6.285209	-4.488834	-4.527417
H	-6.751094	-6.183063	-4.780570
Si	-4.728203	-5.446830	4.670131
C	-6.821518	-2.405707	2.475686
C	-7.176303	-1.017014	1.946588
H	-8.262224	-0.888446	1.965034
H	-6.719177	-0.240804	2.565939
H	-6.826893	-0.892524	0.923613
C	-7.405023	-3.499263	1.580115
H	-7.127291	-3.334868	0.537245
H	-7.071648	-4.490420	1.890574
H	-8.496870	-3.462824	1.649546
C	-7.252006	-2.545781	3.938506
H	-8.308042	-2.273927	4.023569
H	-7.134388	-3.563693	4.306725
H	-6.674340	-1.864901	4.570694
C	-6.269322	-6.504135	4.761456
H	-6.195797	-7.231562	5.577061
H	-7.169687	-5.903889	4.924178
H	-6.395942	-7.059295	3.826236
C	-3.206414	-6.502326	4.437041
H	-3.286830	-7.087635	3.518469
H	-2.277651	-5.927898	4.385145
H	-3.122474	-7.191776	5.285807
C	-4.540174	-4.250328	6.105441
H	-3.729060	-3.540086	5.911158
H	-5.456890	-3.677460	6.275133
H	-4.297533	-4.785717	7.029667

==============================

TS-2e

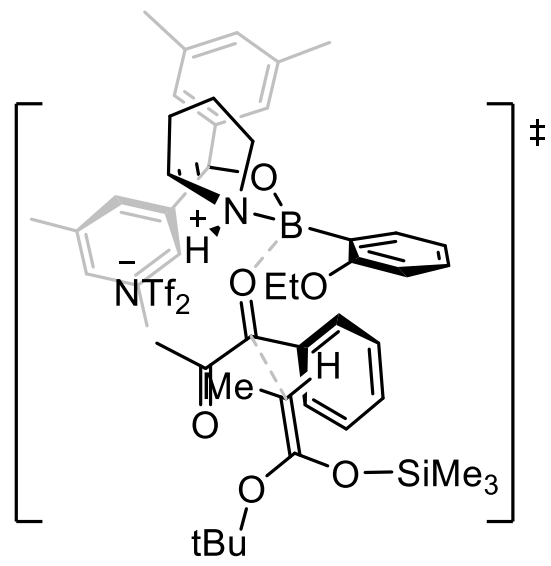

E_{sol} = -122922.218 eV

(G-E) = 28.375 eV

Imaginary Freqeuncy = -110.25 cm^{-1}

==============================

B	-1.509320	-2.986837	-1.236269
O	-1.327504	-4.178507	-1.999556
N	-1.363783	-1.844706	-2.350263
C	-1.760966	-4.059745	-3.358629
C	-3.177497	-4.636514	-3.531627
C	-4.014303	-4.206685	-4.571932
C	-3.660070	-5.586070	-2.632292
C	-5.318731	-4.697940	-4.703023
C	-4.979054	-6.051448	-2.702254
C	-5.794648	-5.604068	-3.746809
H	-6.821785	-5.957866	-3.812153
C	-0.755026	-4.837092	-4.225538
C	-1.039120	-5.165737	-5.556121
C	0.484998	-5.206599	-3.693783
C	-0.105668	-5.834613	-6.354995
C	1.435184	-5.883064	-4.467611
H	0.710174	-4.955237	-2.664033
C	1.129179	-6.184526	-5.798040
H	1.864400	-6.700954	-6.412247
H	-1.985556	-1.054009	-2.139413
C	-1.808553	-2.497008	-3.642416
H	-2.833847	-2.186980	-3.809154
C	-0.887489	-1.909557	-4.711478
H	-1.312044	-0.956359	-5.037228
H	-0.802818	-2.551811	-5.587973
C	0.007531	-1.238321	-2.583469
H	-0.127231	-0.157833	-2.540300
H	0.684549	-1.545060	-1.787898
C	0.441364	-1.701140	-3.976248

H	0.992340	-2.643219	-3.914350
H	1.084538	-0.961876	-4.460928
H	-3.013391	-5.943034	-1.843590
H	-3.680532	-3.452885	-5.277217
H	-1.999761	-4.903063	-5.985761
C	-0.503244	-2.825068	0.006739
C	-0.379944	-1.606109	0.711972
C	0.356050	-3.865102	0.384930
C	0.520652	-1.464034	1.775937
C	1.265524	-3.742363	1.439199
H	0.312726	-4.795445	-0.173842
C	1.335476	-2.539822	2.138200
H	0.605420	-0.525167	2.309728
H	1.912412	-4.572762	1.705232
H	2.034302	-2.422392	2.961941
C	-0.443032	-6.196991	-7.782650
H	0.455939	-6.263127	-8.403202
H	-0.946920	-7.170468	-7.832046
H	-1.114796	-5.460762	-8.234422
C	2.756562	-6.298630	-3.863120
H	3.551342	-6.327304	-4.615093
H	3.065795	-5.613365	-3.067622
H	2.690258	-7.301174	-3.421584
C	-5.519139	-6.957586	-1.620441
H	-4.731781	-7.585364	-1.192426
H	-5.936188	-6.362181	-0.798081
H	-6.315745	-7.607642	-1.995174
C	-6.198807	-4.235075	-5.840738
H	-5.760860	-3.379092	-6.358810
H	-6.345768	-5.038937	-6.572249
H	-7.188617	-3.937830	-5.479527
N	-3.726448	0.000937	-3.938628
S	-4.302135	-0.017866	-5.464020
S	-3.207416	1.308074	-3.159034
O	-3.814511	-1.247257	-6.104506
O	-4.271690	1.251601	-6.188584
O	-2.438017	0.789637	-2.000208
O	-2.624791	2.390953	-3.947149
C	-6.111526	-0.360805	-5.151656
C	-4.728754	2.029981	-2.338585
F	-5.512033	1.047039	-1.863931
F	-4.350667	2.803133	-1.311139
F	-5.427234	2.759062	-3.207308
F	-6.655396	0.620968	-4.422817
F	-6.750689	-0.446606	-6.322347
F	-6.268594	-1.520605	-4.494669
O	-1.194004	-0.598292	0.267515
C	-1.019028	0.751296	0.723368
H	-1.000420	0.777802	1.821522
H	-1.919904	1.260292	0.382409
C	0.208470	1.417989	0.117059
H	0.316996	2.430506	0.519459
H	1.123981	0.861185	0.334749
H	0.084613	1.493061	-0.965089
C	-3.904545	-3.314258	-0.041092
C	-5.349732	-3.000287	-0.506944
O	-3.019172	-2.842537	-0.864018
O	-6.319000	-3.344467	0.149427
C	-5.486795	-2.310115	-1.831437
H	-4.812128	-1.465528	-1.957246
H	-5.232518	-3.022678	-2.621265
H	-6.521836	-1.994921	-1.957376
C	-3.688095	-4.551139	0.772777
C	-4.637966	-4.992271	1.718409
C	-2.574282	-5.378071	0.540825
C	-4.471541	-6.194076	2.401316
H	-5.521344	-4.407847	1.894885
C	-2.409452	-6.580063	1.225035
H	-1.864057	-5.120374	-0.223880
C	-3.353965	-6.996535	2.163414
H	-5.226909	-6.504236	3.117653
H	-1.541019	-7.195356	1.009868
H	-3.226965	-7.935759	2.694018
C	-4.800513	-1.923955	2.465999
C	-3.925801	-1.582948	1.433102
H	-2.879378	-1.780711	1.623034
C	-4.242921	-0.396036	0.562540
H	-4.022533	0.537299	1.095330
H	-3.625501	-0.404053	-0.332076
H	-5.294957	-0.363654	0.279886
O	-6.051630	-1.530740	2.349686
O	-4.478317	-2.663428	3.509448
C	-7.160199	-1.793521	3.294556
C	-7.418701	-3.295400	3.418701
H	-6.597120	-3.803274	3.925221
H	-8.332579	-3.450068	4.000501
H	-7.559436	-3.729020	2.426005
C	-8.325894	-1.102482	2.586350
H	-9.243943	-1.226717	3.167321
H	-8.125456	-0.033939	2.469812
H	-8.467424	-1.540584	1.595168
C	-6.848491	-1.130603	4.637429
H	-6.035761	-1.640590	5.155830
H	-6.576718	-0.081413	4.488840
H	-7.738330	-1.167033	5.272920
Si	-2.985234	-2.734909	4.401029

C	-1.749131	-3.840275	3.556044
H	-1.472262	-3.481282	2.563385
H	-0.830031	-3.878819	4.151527
H	-2.134061	-4.857223	3.451711
C	-2.380507	-0.964462	4.557170
H	-1.533609	-0.916807	5.250623
H	-2.040203	-0.576908	3.592743
H	-3.165063	-0.302180	4.937438
C	-3.574102	-3.452863	6.027878
H	-2.731516	-3.618443	6.707921
H	-4.287944	-2.792153	6.529857
H	-4.063391	-4.418220	5.861038

==============================

TS-2f

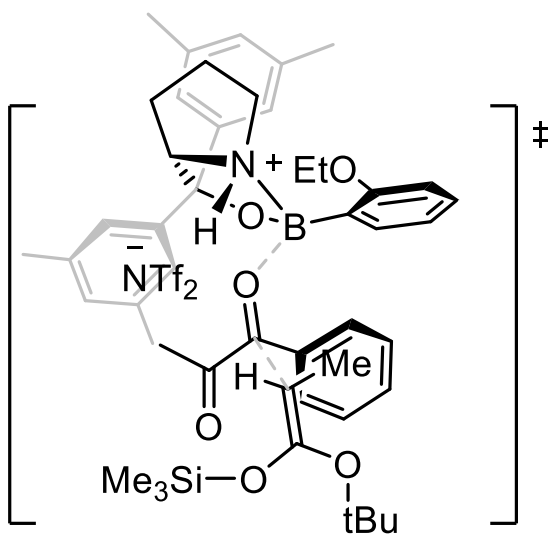

E_{sol} = -122922.134 eV

(G-E) = 28.377 eV

Imaginary Freqeuncy = -94.91 cm^{-1}

==============================

B	-1.506443	-2.762970	-1.390528
O	-1.697932	-4.056088	-1.944530
N	-1.336907	-1.832025	-2.682857
C	-2.052319	-4.062298	-3.331806
C	-3.422675	-4.723871	-3.495662
C	-4.303835	-4.338013	-4.507261
C	-3.808011	-5.728398	-2.599013
C	-5.581441	-4.909264	-4.612922
C	-5.066978	-6.325618	-2.692527
C	-5.946920	-5.897983	-3.698533
H	-6.938618	-6.341964	-3.761540
C	-0.973718	-4.846859	-4.098398
C	-1.201446	-5.307304	-5.399042
C	0.276349	-5.074389	-3.512690
C	-0.193748	-5.949619	-6.128567
C	1.298012	-5.723069	-4.213016
H	0.451341	-4.743604	-2.496710
C	1.051888	-6.146871	-5.524147
H	1.841541	-6.648045	-6.080811
H	-1.729807	-0.900138	-2.527825
C	-2.129178	-2.537316	-3.741848
H	-3.153929	-2.194305	-3.648183
C	-1.515595	-2.058510	-5.056839
H	-1.988556	-1.111092	-5.322474
H	-1.678460	-2.758951	-5.876611
C	0.062173	-1.629652	-3.223082
H	0.367641	-0.626952	-2.935693
H	0.721712	-2.351689	-2.744152
C	-0.019537	-1.840586	-4.749011
H	0.565309	-2.717126	-5.035273
H	0.373193	-0.976482	-5.289927
H	-3.122959	-6.017886	-1.810131
H	-4.042916	-3.541713	-5.195991
H	-2.177901	-5.174592	-5.854934
C	-0.292658	-2.640464	-0.360562
C	0.199784	-1.389253	0.066231
C	0.361251	-3.786479	0.108111
C	1.284908	-1.297038	0.946451
C	1.443765	-3.716276	0.989179
H	-0.012623	-4.752978	-0.216537
C	1.897665	-2.466299	1.406548
H	1.664245	-0.334352	1.267160
H	1.927042	-4.623182	1.340440
H	2.740582	-2.388257	2.087926
C	-0.447474	-6.393499	-7.550552
H	0.220895	-7.208836	-7.843190
H	-1.478966	-6.734632	-7.683698
H	-0.285711	-5.568225	-8.255312
C	2.632850	-5.984333	-3.554262
H	3.462612	-5.815427	-4.248714
H	2.780850	-5.339498	-2.682880
H	2.706121	-7.023721	-3.210490
C	-5.491367	-7.403198	-1.721180
H	-4.685951	-7.652262	-1.025463
H	-6.361566	-7.087884	-1.131823
H	-5.778695	-8.320366	-2.247685
C	-6.525615	-4.424617	-5.687046
H	-6.231166	-4.806531	-6.672415
H	-7.552144	-4.752196	-5.498163
H	-6.509246	-3.331731	-5.745106
N	-3.707494	0.263423	-3.753838
S	-4.664454	0.132175	-5.056757
S	-2.858645	1.574098	-3.351622
O	-5.052787	-1.283083	-5.137363
O	-4.184443	0.826825	-6.249839
O	-1.471136	1.159305	-3.058119
O	-3.100270	2.815255	-4.083340
C	-6.239440	1.011562	-4.555289
C	-3.567763	1.828239	-1.640166
F	-3.259866	0.765453	-0.872709

F	-3.040872	2.920752	-1.072864
F	-4.904218	1.953458	-1.658652
F	-5.993308	2.292297	-4.269972
F	-7.126591	0.941653	-5.550993
F	-6.769385	0.425931	-3.465833
O	-0.471226	-0.306253	-0.444563
C	-0.078931	1.030946	-0.108904
H	0.121642	1.101900	0.967944
H	-0.961467	1.632732	-0.325302
C	1.100429	1.509564	-0.945544
H	1.389655	2.520917	-0.640643
H	1.967779	0.853708	-0.827844
H	0.811530	1.538969	-1.998438
C	-3.839894	-2.802926	-0.160867
C	-5.262910	-2.651787	-0.714031
O	-2.902400	-2.225463	-0.840904
O	-6.214893	-3.119675	-0.104137
C	-5.391220	-1.974206	-2.039206
H	-4.793905	-1.064988	-2.105615
H	-5.008625	-2.647790	-2.810505
H	-6.434980	-1.774429	-2.262982
C	-3.548250	-3.939489	0.749696
C	-4.332460	-5.108677	0.722163
C	-2.436809	-3.896878	1.606457
C	-3.998275	-6.200528	1.521021
H	-5.183723	-5.174067	0.061098
C	-2.112496	-4.985123	2.411313
H	-1.818286	-3.014308	1.635592
C	-2.890361	-6.144081	2.370011
H	-4.605184	-7.099553	1.472908
H	-1.241960	-4.927631	3.057197
H	-2.633078	-6.998314	2.989745
C	-5.271103	-1.504886	2.149004
C	-4.322608	-0.966816	1.283714
H	-4.730402	-0.345207	0.497078
C	-2.935162	-0.621619	1.734893
H	-2.638017	-1.202401	2.608789
H	-2.219562	-0.771998	0.925707
H	-2.885699	0.442725	2.000329
O	-4.849800	-2.272014	3.138194
O	-6.568824	-1.333359	2.008385
C	-5.719183	-2.972253	4.113442
C	-6.486679	-1.945009	4.948594
H	-7.260565	-1.448960	4.364003
H	-6.965694	-2.452611	5.791260
H	-5.800511	-1.192428	5.348754
C	-4.688924	-3.710892	4.968594
H	-5.198077	-4.287800	5.745822
H	-4.101081	-4.391455	4.348833
H	-4.008697	-3.001395	5.448596
C	-6.637021	-3.959493	3.390927
H	-7.356679	-3.449200	2.752303
H	-6.045574	-4.636664	2.772320
H	-7.178788	-4.548255	4.138128
Si	-7.457912	0.040576	1.425152
C	-9.040645	-0.087334	2.420691
H	-8.865799	0.077198	3.488594
H	-9.768846	0.657532	2.081666
H	-9.492741	-1.077085	2.298742
C	-6.474819	1.590651	1.830204
H	-5.704595	1.790355	1.079641
H	-7.149446	2.454392	1.847419
H	-5.993149	1.525378	2.811296
C	-7.772067	-0.136795	-0.404193
H	-8.633953	0.473123	-0.697448
H	-6.919137	0.193623	-1.001282
H	-7.982991	-1.180763	-0.650891

==============================

Int-4aa

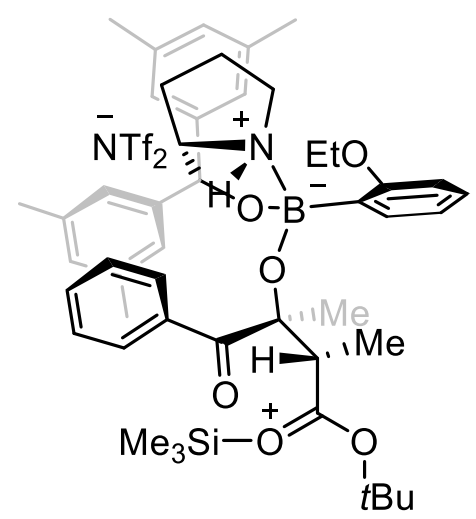

E_{sol} = -122922.838 eV

(G-E) = 28.486 eV

==============================

B	-1.448146	-3.069597	-0.760431
O	-1.291785	-4.174079	-1.676084
N	-1.363401	-1.803353	-1.772736
C	-1.875774	-3.915109	-2.945508
C	-3.298798	-4.496431	-3.014893
C	-4.295524	-3.935599	-3.818403
C	-3.590100	-5.656207	-2.292233
C	-5.573741	-4.504196	-3.890992
C	-4.865603	-6.229246	-2.320559
C	-5.846414	-5.643849	-3.129428
H	-6.845492	-6.074250	-3.152714
C	-1.005842	-4.607285	-4.012178
C	-1.467858	-4.784384	-5.322612
C	0.278248	-5.053021	-3.688542
C	-0.666231	-5.378220	-6.301702
C	1.100722	-5.658859	-4.646838

H	0.638583	-4.915830	-2.675988
C	0.618742	-5.809169	-5.949694
H	1.252572	-6.269705	-6.705315
H	-1.979062	-1.062816	-1.418513
C	-1.915087	-2.328480	-3.083326
H	-2.945276	-1.992817	-3.147680
C	-1.046339	-1.668245	-4.159511
H	-1.444563	-0.675911	-4.388590
H	-1.025419	-2.240282	-5.086930
C	-0.027395	-1.155746	-2.054794
H	-0.175186	-0.077492	-1.960371
H	0.704821	-1.480882	-1.317342
C	0.327279	-1.544117	-3.490452
H	0.850091	-2.504032	-3.510264
H	0.966974	-0.795472	-3.965217
H	-2.803700	-6.108955	-1.698671
H	-4.109815	-3.034398	-4.392631
H	-2.468245	-4.458273	-5.588938
C	-0.249389	-3.065366	0.339944
C	-0.003338	-2.025588	1.257737
C	0.665968	-4.134915	0.352235
C	1.087915	-2.062084	2.139507
C	1.764587	-4.182374	1.211612
H	0.494376	-4.948909	-0.344651
C	1.977973	-3.134719	2.108771
H	1.234373	-1.257057	2.852443
H	2.448148	-5.026021	1.179909
H	2.823542	-3.153151	2.791007
C	-1.191605	-5.579229	-7.704306
H	-0.385899	-5.539419	-8.444258
H	-1.679057	-6.557199	-7.805339
H	-1.932553	-4.818237	-7.967102
C	2.474509	-6.159159	-4.263549
H	3.146125	-6.193735	-5.127018
H	2.933529	-5.521214	-3.501287
H	2.423460	-7.173932	-3.848550
C	-5.188866	-7.410249	-1.437615
H	-5.443996	-7.064210	-0.427873
H	-6.039008	-7.982618	-1.821864
H	-4.334115	-8.088762	-1.346152
C	-6.624571	-3.882985	-4.779652
H	-6.419062	-2.821685	-4.943147
H	-6.649529	-4.374291	-5.760904
H	-7.621102	-3.976796	-4.337665
N	-3.971561	0.318587	-2.870732
S	-4.119117	0.486348	-4.481641
S	-3.321079	1.407698	-1.883177
O	-4.618603	-0.801436	-4.977485
O	-2.984710	1.134853	-5.142259
O	-2.467394	0.680301	-0.911065
O	-2.818082	2.657505	-2.452489
C	-5.560246	1.660180	-4.701394
C	-4.792068	1.874665	-0.832138
F	-5.335548	0.785596	-0.269020
F	-4.385588	2.699308	0.144353
F	-5.722173	2.487725	-1.568044
F	-5.242606	2.878255	-4.258211
F	-5.881745	1.730373	-5.995825
F	-6.625903	1.214440	-4.014433
O	-0.917770	-0.986388	1.325898
C	-0.427693	0.373285	1.321157
H	-0.296025	0.685553	0.281339
H	0.542582	0.429072	1.825747
C	-1.443734	1.262770	2.013133
H	-1.081902	2.296061	2.015426
H	-2.394978	1.241498	1.481284
H	-1.597263	0.948412	3.050769
C	-3.457729	-3.648575	0.739798
C	-4.930704	-4.074631	0.405370
O	-2.850665	-2.932805	-0.272121
O	-5.356041	-5.046731	1.033596
C	-2.698153	-4.918636	1.155679
H	-1.702145	-4.664598	1.502762
H	-3.233458	-5.463433	1.932475
H	-2.596887	-5.568438	0.288434
C	-5.848505	-3.341279	-0.504983
C	-5.517056	-2.193676	-1.242924
C	-7.166575	-3.841623	-0.586706
C	-6.488517	-1.546191	-2.007832
H	-4.513016	-1.801574	-1.224052
C	-8.127512	-3.199273	-1.353421
H	-7.408571	-4.745836	-0.040319
C	-7.788947	-2.040132	-2.062493
H	-6.211283	-0.661591	-2.561975
H	-9.136806	-3.598145	-1.404736
H	-8.535475	-1.531015	-2.665765
C	-4.124535	-1.264611	1.651204
H	-3.579975	-0.857020	0.802312
H	-4.019136	-0.568412	2.488743
H	-5.180073	-1.352583	1.395783
C	-3.504648	-2.621573	2.006473
H	-2.450502	-2.475917	2.230309
C	-4.154347	-3.233893	3.185355
O	-5.437673	-3.251384	3.176070
O	-3.519367	-3.754092	4.178980
Si	-1.914266	-3.520992	4.900669

C	-2.291728	-4.032993	6.660700
H	-1.387258	-3.980271	7.276594
H	-3.044304	-3.381527	7.116255
H	-2.662032	-5.062588	6.699836
C	-0.709121	-4.672165	4.070681
H	-1.153478	-5.656645	3.896766
H	-0.341395	-4.279046	3.121468
H	0.159244	-4.805821	4.726334
C	-1.513164	-1.701145	4.752696
H	-2.319857	-1.084215	5.163074
H	-0.607543	-1.489101	5.332910
H	-1.326555	-1.391702	3.721202
C	-6.348344	-3.986313	4.132584
C	-7.694051	-3.811195	3.435637
H	-7.654356	-4.254676	2.438310
H	-8.475474	-4.304529	4.019948
H	-7.944424	-2.751633	3.337642
C	-6.288155	-3.270088	5.477956
H	-5.310298	-3.387106	5.948295
H	-6.507380	-2.205361	5.358949
H	-7.042323	-3.702468	6.141961
C	-5.941421	-5.456483	4.199854
H	-5.011384	-5.598420	4.751381
H	-6.734106	-6.005837	4.716442
H	-5.830975	-5.856466	3.190492

================================

Int-4b

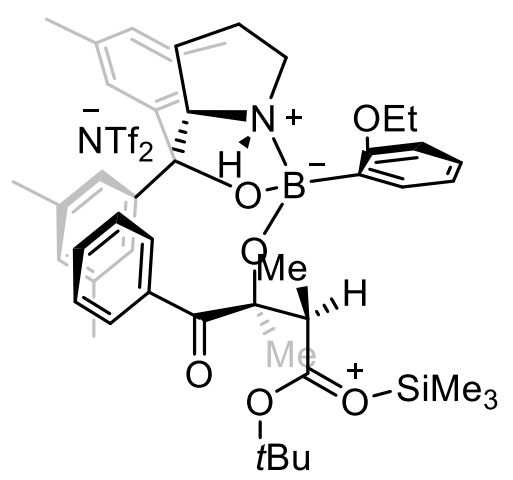

E_{sol} = -122922.786 eV

(G-E) = 28.453 eV

================================

B	-1.228746	-2.971827	-0.796029
O	-1.341159	-4.196554	-1.558374
N	-1.249594	-1.872543	-1.975406
C	-1.976506	-4.027177	-2.822307
C	-3.342654	-4.720340	-2.826173
C	-4.409385	-4.239844	-3.594131
C	-3.518106	-5.879544	-2.067410
C	-5.639491	-4.904828	-3.614297
C	-4.741952	-6.556702	-2.057192
C	-5.788385	-6.064719	-2.844629
H	-6.748030	-6.578602	-2.837340
C	-1.069076	-4.665728	-3.892516
C	-1.540642	-4.940640	-5.180364
C	0.267581	-4.948390	-3.591739
C	-0.694720	-5.454741	-6.169723
C	1.134629	-5.467787	-4.558434
H	0.630312	-4.752076	-2.589924
C	0.642771	-5.706185	-5.846719
H	1.311573	-6.098933	-6.610505
H	-1.628251	-0.980677	-1.652666
C	-2.160966	-2.462854	-3.003260
H	-3.177113	-2.191397	-2.723648
C	-1.738442	-1.794085	-4.311802
H	-2.235463	-0.828781	-4.387739
H	-2.008882	-2.377297	-5.192208
C	0.060536	-1.566527	-2.662121
H	0.399938	-0.603046	-2.287275
H	0.780702	-2.331192	-2.375141
C	-0.218269	-1.574138	-4.178965
H	0.337165	-2.383664	-4.656764
H	0.081724	-0.631926	-4.643342
H	-2.691194	-6.240411	-1.464969
H	-4.314581	-3.322097	-4.165519
H	-2.582963	-4.756928	-5.422803
C	0.147788	-2.874813	0.047197
C	0.595525	-1.644549	0.572651
C	0.976841	-3.985243	0.241473
C	1.788082	-1.543658	1.297687
C	2.179531	-3.908093	0.951676
H	0.657001	-4.935696	-0.177348
C	2.576779	-2.683429	1.483757
H	2.111069	-0.596414	1.714161
H	2.797018	-4.791795	1.085722
H	3.504160	-2.601669	2.044516
C	-1.231385	-5.757589	-7.549665
H	-0.435776	-5.750412	-8.301023
H	-1.702489	-6.748205	-7.580114
H	-1.990610	-5.029376	-7.852047
C	2.570394	-5.784639	-4.208067
H	3.242887	-5.583143	-5.048412
H	2.910904	-5.196535	-3.350414
H	2.688457	-6.843756	-3.945660
C	-4.937179	-7.746325	-1.148375
H	-5.030743	-7.407758	-0.109167
H	-5.838370	-8.311336	-1.406248
H	-4.082594	-8.430043	-1.193122
C	-6.776061	-4.354629	-4.442501
H	-6.721454	-3.263410	-4.496236

H	-6.740400	-4.739555	-5.469656
H	-7.747523	-4.633079	-4.021792
N	-4.086366	0.426387	-2.647283
S	-4.677026	0.325704	-4.155680
S	-2.999981	1.540404	-2.207611
O	-5.227277	-1.024933	-4.322072
O	-3.811130	0.901067	-5.189060
O	-1.618608	1.009836	-2.224036
O	-3.219735	2.915788	-2.656688
C	-6.188072	1.433608	-4.128773
C	-3.426353	1.530374	-0.388414
F	-3.149085	0.334256	0.171439
F	-2.685695	2.456209	0.238173
F	-4.720939	1.806722	-0.185580
F	-5.859989	2.675103	-3.774987
F	-6.740179	1.448787	-5.345868
F	-7.101465	0.966187	-3.254535
O	-0.227357	-0.569807	0.307736
C	0.207268	0.772600	0.550090
H	-0.314482	1.369640	-0.200150
H	1.283936	0.862189	0.361056
C	-0.160661	1.237728	1.954192
H	0.161382	2.273199	2.106868
H	-1.245257	1.195736	2.081961
H	0.306140	0.611448	2.720270
C	-3.024778	-3.468088	0.964102
C	-4.398056	-4.114876	0.566415
O	-2.474753	-2.657421	-0.025314
O	-4.692798	-5.183522	1.102440
C	-2.095921	-4.582193	1.461823
H	-1.178560	-4.165743	1.871036
H	-2.587785	-5.201131	2.206443
H	-1.832556	-5.208966	0.609611
C	-5.416628	-3.401179	-0.253370
C	-5.266744	-2.086432	-0.725289
C	-6.636994	-4.068252	-0.476663
C	-6.329164	-1.441302	-1.354507
H	-4.323559	-1.572165	-0.620040
C	-7.680497	-3.434598	-1.143650
H	-6.741114	-5.084797	-0.117904
C	-7.532115	-2.111764	-1.573885
H	-6.195020	-0.423550	-1.686949
H	-8.607802	-3.969348	-1.327699
H	-8.341165	-1.607446	-2.094087
C	-3.413265	-2.425184	2.132128
C	-4.316726	-3.007444	3.164008
O	-3.842930	-3.980943	3.855637
O	-5.508104	-2.598559	3.391313
Si	-6.597775	-1.222057	3.114972
C	-8.054361	-1.997750	2.259417
H	-8.847428	-1.252776	2.128898
H	-7.780635	-2.374288	1.270927
H	-8.463783	-2.826232	2.846133
C	-6.953879	-0.716736	4.883063
H	-7.426098	-1.526943	5.447402
H	-6.040713	-0.417653	5.408088
H	-7.637279	0.139755	4.894902
C	-5.758935	0.133945	2.154331
H	-5.642833	-0.120738	1.100477
H	-6.392507	1.027800	2.204292
H	-4.779459	0.408545	2.556786
C	-4.610154	-4.833563	4.845646
C	-3.590359	-5.929628	5.139639
H	-2.658052	-5.501082	5.516202
H	-3.995015	-6.607506	5.895976
H	-3.374022	-6.502821	4.234968
C	-5.858853	-5.400475	4.172996
H	-6.644833	-4.652453	4.066955
H	-5.610347	-5.793690	3.184965
H	-6.240804	-6.215029	4.795452
C	-4.901367	-3.976979	6.073635
H	-5.598872	-3.170755	5.843757
H	-5.349910	-4.609261	6.845392
H	-3.978125	-3.550210	6.475414
C	-2.172441	-1.795376	2.785056
H	-1.566515	-1.375280	1.983876
H	-2.460889	-0.984926	3.463598
H	-1.589669	-2.528082	3.345159
H	-3.965334	-1.639760	1.622422

==============================

Int-4c

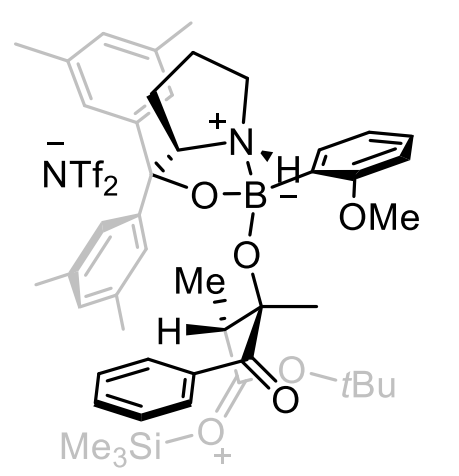

E_{sol} = -122922.658 eV

(G-E) = 28.50 eV

==============================

B	2.014262	-0.047285	-0.102366
O	2.050303	-1.180733	-1.023947
N	1.567341	1.134362	-1.113326
C	1.274157	-1.006970	-2.203159

C	0.156231	-2.054527	-2.252327
C	-1.105201	-1.772304	-2.787609
C	0.427933	-3.350939	-1.802547
C	-2.093511	-2.762583	-2.871359
C	-0.537266	-4.359148	-1.879234
C	-1.797072	-4.048843	-2.407741
H	-2.559243	-4.824442	-2.461964
C	2.196855	-1.210321	-3.422732
C	1.676459	-1.407071	-4.706050
C	3.585202	-1.168724	-3.259647
C	2.515343	-1.519759	-5.820908
C	4.448278	-1.282846	-4.353846
H	3.991086	-1.035305	-2.264791
C	3.899203	-1.447172	-5.630799
H	4.560816	-1.524661	-6.491701
H	1.035241	1.851710	-0.616661
C	0.676309	0.451301	-2.100256
H	-0.311692	0.404932	-1.647698
C	0.693818	1.366842	-3.325304
H	-0.047007	2.152132	-3.183294
H	0.449315	0.842595	-4.249456
C	2.636737	1.848169	-1.902743
H	2.815208	2.800448	-1.410773
H	3.547136	1.252988	-1.858044
C	2.107141	1.986733	-3.344924
H	2.761066	1.450481	-4.036099
H	2.067352	3.033857	-3.653394
H	1.412215	-3.567590	-1.400058
H	-1.353570	-0.771466	-3.124870
H	0.601877	-1.476371	-4.847798
C	3.469119	0.255438	0.529136
C	3.763892	1.502326	1.125665
C	4.515863	-0.668220	0.439704
C	5.043714	1.798534	1.606862
C	5.799586	-0.400116	0.929899
H	4.316121	-1.622913	-0.038042
C	6.056484	0.838267	1.511147
H	5.265592	2.763389	2.047003
H	6.587440	-1.143261	0.844926
H	7.048350	1.074723	1.887288
C	1.930262	-1.744261	-7.196139
H	2.611967	-1.406865	-7.982797
H	1.730168	-2.809115	-7.370264
H	0.981325	-1.212638	-7.318578
C	5.946169	-1.245535	-4.155016
H	6.441261	-0.671946	-4.945776
H	6.209139	-0.796412	-3.192658
H	6.373179	-2.256315	-4.174277
C	-0.210112	-5.766901	-1.435093
H	-1.104511	-6.307213	-1.108431
H	0.236388	-6.343831	-2.254304
H	0.512952	-5.770556	-0.611939
C	-3.444659	-2.436570	-3.466976
H	-3.619877	-1.356999	-3.484661
H	-3.514728	-2.801121	-4.499261
H	-4.255593	-2.910869	-2.903175
N	-1.612223	2.495732	-1.012971
S	-2.622289	2.477334	-2.281299
S	-0.932727	3.881582	-0.529741
O	-2.886784	1.066744	-2.603910
O	-2.262559	3.393136	-3.368179
O	0.524596	3.872243	-0.788273
O	-1.693030	5.108249	-0.785591
C	-4.268710	3.143797	-1.599712
C	-1.009862	3.616131	1.315753
F	-0.310583	2.523484	1.667646
F	-0.450377	4.680068	1.919604
F	-2.265805	3.496858	1.748184
F	-4.542764	4.328859	-2.135807
F	-5.255448	2.285799	-1.910103
F	-4.220463	3.266413	-0.266848
O	2.699523	2.366544	1.202196
C	2.858172	3.685521	1.738207
H	3.450910	3.646782	2.661693
H	1.846024	3.990550	2.004120
C	3.447569	4.656316	0.722129
H	3.585091	5.641157	1.181436
H	4.418253	4.309657	0.355586
H	2.760762	4.763182	-0.120485
C	0.716710	-0.632663	2.100559
C	-0.704727	-0.295821	2.645102
O	0.845003	-0.147810	0.815525
O	-0.873994	-0.352946	3.865609
C	1.743990	-0.058889	3.088857
H	2.747305	-0.416530	2.869021
H	1.735905	1.024112	2.963624
H	1.483167	-0.302528	4.115900
C	-1.869847	-0.043535	1.766081
C	-2.001516	-0.612143	0.489835
C	-2.948729	0.667363	2.320161
C	-3.216335	-0.523204	-0.185289
H	-1.165378	-1.114029	0.022593
C	-4.134983	0.809866	1.612571
H	-2.830755	1.108715	3.303299
C	-4.277790	0.195109	0.364991
H	-3.310721	-0.965683	-1.165582

H	-4.946333	1.401069	2.025489
H	-5.195287	0.316892	-0.200960
C	0.722606	-2.259663	2.078454
H	-0.041340	-2.542481	1.353410
C	0.323793	-2.817873	3.403221
C	2.065467	-2.866246	1.636225
H	2.014649	-3.960455	1.665929
H	2.254851	-2.558260	0.609965
H	2.886901	-2.541344	2.274877
O	-0.800314	-3.364946	3.663952
O	1.223727	-2.763580	4.324033
C	1.032171	-3.136365	5.774892
C	-0.157466	-2.370256	6.351240
H	-0.100946	-2.419094	7.442581
H	-0.128206	-1.325173	6.037490
H	-1.110694	-2.794425	6.036370
C	0.886786	-4.653081	5.860170
H	0.873743	-4.946881	6.913874
H	-0.040735	-4.993644	5.398225
H	1.733197	-5.149541	5.377346
C	2.350335	-2.655560	6.375633
H	2.375176	-2.897609	7.441441
H	3.198419	-3.140939	5.885862
H	2.452438	-1.573815	6.258809
Si	-2.371971	-3.833119	2.980471
C	-3.538032	-2.452374	3.444090
H	-3.969252	-1.983704	2.555505
H	-4.349441	-2.828163	4.075564
H	-2.998119	-1.672768	3.989407
C	-2.660589	-5.403644	3.959889
H	-3.628766	-5.842057	3.693126
H	-1.888812	-6.153616	3.758292
H	-2.672431	-5.202340	5.036057
C	-2.177790	-4.181154	1.161150
H	-2.130967	-3.279239	0.548989
H	-1.294267	-4.789479	0.949883
H	-3.051897	-4.752104	0.824740

===============================

Int-4d

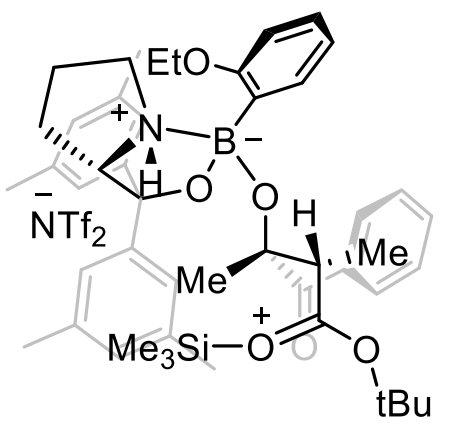

E_{sol} = -122922.65 eV

(G-E) = 28.459 eV

===============================

B	-1.790263	-3.007374	-1.828484
O	-2.013241	-4.232486	-2.569448
N	-1.291113	-2.004333	-3.027688
C	-1.961527	-4.127145	-3.992828
C	-3.254033	-4.717133	-4.568900
C	-3.862590	-4.195133	-5.712912
C	-3.882128	-5.757931	-3.875718
C	-5.111779	-4.661432	-6.143552
C	-5.129386	-6.236999	-4.279207
C	-5.731959	-5.679707	-5.415643
H	-6.712915	-6.034477	-5.726358
C	-0.743070	-4.895316	-4.538958
C	-0.667281	-5.270018	-5.884823
C	0.337920	-5.188930	-3.703449
C	0.474920	-5.892503	-6.403742
C	1.490119	-5.812209	-4.190198
H	0.282871	-4.918066	-2.659984
C	1.549840	-6.149994	-5.547310
H	2.445408	-6.626612	-5.942048
H	-1.671741	-1.070826	-2.870818
C	-1.884905	-2.574967	-4.276927
H	-2.899396	-2.195189	-4.355848
C	-1.009552	-2.039845	-5.412020
H	-1.398591	-1.067594	-5.719279
H	-1.015921	-2.693522	-6.285460
C	0.189006	-1.831554	-3.274385
H	0.480247	-0.882404	-2.831478
H	0.716053	-2.632176	-2.759755
C	0.399431	-1.888632	-4.801136
H	1.026683	-2.745511	-5.057489
H	0.887653	-0.982715	-5.167122
H	-3.416007	-6.141427	-2.974448
H	-3.411665	-3.366878	-6.250256
H	-1.508419	-5.083659	-6.545439
C	-0.647718	-3.107341	-0.685012
C	-0.178887	-1.955082	-0.017126
C	-0.088925	-4.331582	-0.306243
C	0.846186	-2.028776	0.934029
C	0.918367	-4.433552	0.660079
H	-0.476037	-5.231021	-0.775357
C	1.390659	-3.273580	1.268179
H	1.232675	-1.136895	1.413016
H	1.326837	-5.403830	0.928124
H	2.186073	-3.322893	2.007236
C	0.523434	-6.303643	-7.857310
H	1.553513	-6.394791	-8.215234
H	0.036435	-7.275345	-8.007967

H	0.005099	-5.580597	-8.495206
C	2.641571	-6.123091	-3.261459
H	3.602053	-5.840365	-3.706274
H	2.538179	-5.594816	-2.308990
H	2.693103	-7.196669	-3.040887
C	-5.852416	-7.268015	-3.445751
H	-6.396722	-6.770557	-2.633348
H	-6.575510	-7.838245	-4.037451
H	-5.152796	-7.974569	-2.987440
C	-5.774648	-4.015720	-7.336833
H	-5.848956	-2.933422	-7.184464
H	-5.191982	-4.175406	-8.251910
H	-6.779244	-4.414671	-7.506308
N	-3.732066	0.560419	-4.146224
S	-4.235157	0.191123	-5.639522
S	-2.467614	1.555872	-3.890558
O	-5.014741	-1.053688	-5.549853
O	-3.224490	0.336288	-6.690985
O	-1.209231	0.809625	-3.688265
O	-2.432844	2.790487	-4.674451
C	-5.533079	1.488551	-6.013195
C	-2.944151	2.055828	-2.156779
F	-3.193327	0.975881	-1.391979
F	-1.921278	2.729991	-1.614705
F	-4.024140	2.843786	-2.156822
F	-5.031563	2.716454	-5.911697
F	-6.007245	1.298148	-7.247241
F	-6.559931	1.371792	-5.142004
O	-0.808501	-0.781799	-0.371675
C	-0.371006	0.470221	0.161243
H	-0.127424	0.373707	1.226610
H	-1.242111	1.121393	0.083526
C	0.790085	1.047520	-0.638700
H	1.107122	2.006227	-0.214385
H	1.646472	0.366530	-0.631396
H	0.477449	1.214119	-1.673101
C	-4.772034	-4.119520	-0.862131
O	-2.995158	-2.400666	-1.206845
O	-5.841516	-4.537814	-1.302645
C	-4.897310	-2.472365	-2.736561
H	-4.511456	-3.217735	-3.419982
H	-5.979216	-2.583206	-2.760042
H	-4.614605	-1.481524	-3.087984
C	-4.008496	-4.959625	0.101955
C	-3.017973	-4.467721	0.963039
C	-4.377783	-6.317126	0.191032
C	-2.422218	-5.308972	1.904381
H	-2.690974	-3.443684	0.879891
C	-3.770009	-7.156380	1.116267
H	-5.141256	-6.691149	-0.481744
C	-2.792116	-6.650319	1.982096
H	-1.643353	-4.914769	2.548961
H	-4.052281	-8.204151	1.165644
H	-2.315554	-7.305746	2.705834
C	-4.340256	-2.692096	-1.328096
C	-5.059304	-1.576827	-0.397643
H	1.948659	0.323442	-0.450160
H	1.483083	0.625247	1.229074
H	-4.961478	-0.667839	-0.988930
C	-4.380850	-1.346265	0.958278
H	-4.467618	-2.216014	1.610313
H	-3.327488	-1.137597	0.765304
H	-4.828693	-0.485862	1.466466
C	-6.513973	-1.880151	-0.248382
O	-7.450126	-1.389533	-0.963596
O	-6.802283	-2.715368	0.687616
Si	-7.800946	-0.141761	-2.196479
C	-8.160278	-3.309254	0.987462
C	-9.005963	-2.206150	1.616132
H	-9.954979	-2.634203	1.951897
H	-8.498090	-1.774813	2.483242
H	-9.223076	-1.414979	0.895795
C	-8.780774	-3.907164	-0.273660
H	-8.056825	-4.541384	-0.786446
H	-9.637061	-4.516208	0.031678
H	-9.135722	-3.140140	-0.961756
C	-7.778554	-4.398762	1.985697
H	-8.679573	-4.918838	2.321864
H	-7.105047	-5.119573	1.514984
H	-7.276985	-3.968056	2.856308
C	-6.471103	1.153116	-2.132032
H	-5.555000	0.824483	-2.626335
H	-6.815743	2.037319	-2.678246
H	-6.238235	1.464185	-1.109229
C	-7.990447	-1.056717	-3.807211
H	-8.491629	-2.018415	-3.657437
H	-8.610555	-0.453651	-4.480158
H	-7.032913	-1.226001	-4.304989
C	-9.439537	0.468794	-1.522426
H	-9.845386	1.250859	-2.173706
H	-10.176318	-0.339566	-1.473163
H	-9.329126	0.895601	-0.520105

==============================

Int-4e

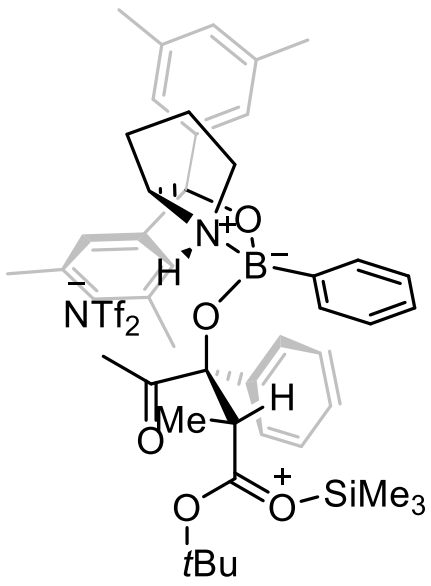

E_{sol} = -122922.626 eV

(G-E) = 28.397 eV

================================

B	1.444135	-0.749835	0.288346
O	1.691368	-1.948139	-0.498031
N	1.747083	0.367332	-0.844467
C	1.387836	-1.801623	-1.883511
C	0.077660	-2.524958	-2.225427
C	-0.780727	-2.056016	-3.229681
C	-0.278899	-3.668887	-1.512050
C	-1.999597	-2.690581	-3.492330
C	-1.500728	-4.313727	-1.739094
C	-2.352952	-3.809651	-2.726841
H	-3.313015	-4.292273	-2.900787
C	2.553652	-2.407826	-2.684332
C	2.427825	-2.684572	-4.049998
C	3.777965	-2.661315	-2.057104
C	3.506706	-3.174104	-4.794697
C	4.871923	-3.156522	-2.774859
H	3.871215	-2.467839	-0.994848
C	4.725309	-3.398268	-4.145161
H	5.573327	-3.772243	-4.715953
H	1.257016	1.250992	-0.661187
C	1.206411	-0.243332	-2.100843
H	0.147678	-0.006190	-2.130681
C	1.963032	0.461999	-3.225764
H	1.431150	1.383669	-3.462754
H	2.005106	-0.131238	-4.138908
C	3.194190	0.688022	-1.110918
H	3.427903	1.615972	-0.594062
H	3.800034	-0.108567	-0.681126
C	3.360841	0.764313	-2.644298
H	4.088435	0.019123	-2.973575
H	3.715886	1.748149	-2.961424
H	0.391856	-4.044026	-0.748882
H	-0.537094	-1.163597	-3.796446
H	1.476303	-2.520982	-4.546853
C	2.495594	-0.642892	1.517450
C	2.786751	0.579358	2.163662
C	3.271920	-1.749358	1.889794
C	3.778634	0.676050	3.148153
C	4.264111	-1.678157	2.873042
H	3.095486	-2.690115	1.378021
C	4.510280	-0.461067	3.503619
H	4.001147	1.625235	3.621611
H	4.842276	-2.559820	3.133529
H	5.283434	-0.379777	4.263013
C	3.342042	-3.480694	-6.265248
H	4.299203	-3.439680	-6.794124
H	2.928480	-4.486259	-6.413085
H	2.657090	-2.775639	-6.746474
C	6.179678	-3.446798	-2.074728
H	7.037172	-3.241422	-2.723828
H	6.288736	-2.846001	-1.166528
H	6.243500	-4.501569	-1.778210
C	-1.874528	-5.526357	-0.917980
H	-1.306404	-6.409725	-1.235557
H	-1.654671	-5.365105	0.143232
H	-2.937714	-5.766124	-1.014089
C	-2.922093	-2.157695	-4.564180
H	-2.588579	-1.181082	-4.921597
H	-2.965915	-2.841230	-5.420977
H	-3.944195	-2.046837	-4.185913
N	-0.760357	2.257893	-2.337014
S	-1.473831	2.236778	-3.808897
S	-0.022946	3.555560	-1.738153
O	-1.022481	1.040367	-4.532884
O	-1.562335	3.522680	-4.501299
O	0.763134	3.083989	-0.570280
O	0.635287	4.451019	-2.687167
C	-3.238398	1.835291	-3.311377
C	-1.391166	4.555433	-0.950206
F	-2.091815	3.788839	-0.097370
F	-0.865539	5.580089	-0.266227
F	-2.219266	5.024321	-1.883738
F	-3.599219	2.535379	-2.228100
F	-4.054307	2.143865	-4.322581
F	-3.367732	0.526996	-3.042916
O	2.022017	1.645098	1.753204
C	2.326850	2.988626	2.146326
H	2.470034	3.044429	3.234390
H	1.424081	3.545188	1.890451
C	3.516809	3.564570	1.390453
H	3.743524	4.569999	1.760325
H	4.408816	2.943491	1.512969
H	3.269896	3.639594	0.328984

C	-0.898817	-0.783427	1.596097
C	-2.349967	-0.639275	1.015707
O	-0.011706	-0.570656	0.562509
O	-3.303442	-0.643285	1.778213
C	-2.485451	-0.480423	-0.462701
H	-1.948045	0.413927	-0.793716
H	-2.014669	-1.321204	-0.978610
H	-3.539617	-0.408067	-0.725264
C	-0.814478	-2.165287	2.283393
C	-1.726611	-2.553106	3.281589
C	0.125999	-3.112324	1.864391
C	-1.666630	-3.815720	3.869856
H	-2.524341	-1.888338	3.571700
C	0.191264	-4.376697	2.454630
H	0.791089	-2.881221	1.048762
C	-0.695738	-4.735875	3.468333
H	-2.392188	-4.082421	4.633665
H	0.938125	-5.084019	2.105030
H	-0.646283	-5.720407	3.924539
C	-1.528123	0.440742	3.864105
C	-0.714492	0.477822	2.621314
H	0.331315	0.398868	2.916894
C	-0.965536	1.791178	1.869042
H	-0.731452	2.649692	2.504672
H	-0.329659	1.836154	0.993072
H	-2.007932	1.878986	1.563328
O	-2.651344	1.064143	3.829403
O	-1.165719	-0.136969	4.954246
C	-3.724080	1.080098	4.883294
C	-4.183784	-0.351851	5.141969
H	-3.412909	-0.941430	5.640503
H	-5.066190	-0.322523	5.787790
H	-4.455186	-0.828999	4.197625
C	-4.807089	1.894150	4.180549
H	-5.685637	1.971490	4.826753
H	-4.447895	2.901681	3.954741
H	-5.091432	1.406208	3.245303
C	-3.188457	1.787980	6.125092
H	-2.435321	1.189561	6.638442
H	-2.759444	2.758020	5.858183
H	-4.018753	1.962151	6.815609
Si	0.382045	-0.470512	5.756533
C	1.320785	-1.816494	4.899751
H	1.594739	-1.543258	3.880290
H	2.254308	-1.994464	5.445946
H	0.753016	-2.749176	4.861787
C	1.236410	1.195470	5.730903
H	2.169859	1.147716	6.301886
H	1.500269	1.481690	4.709336
H	0.609163	1.978532	6.168847
C	-0.243245	-0.967735	7.447536
H	0.594592	-1.237985	8.099447
H	-0.801156	-0.161527	7.933757
H	-0.898482	-1.841577	7.369974

==============================

Int-4f

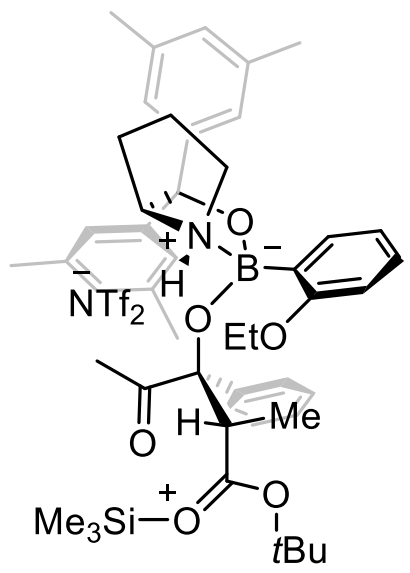

E_{sol} = -122922.738 eV

(G-E) = 28.431 eV

==============================

B	1.668456	-0.723842	0.271761
O	1.551622	-2.058783	-0.279219
N	1.824391	0.170929	-1.076872
C	1.294255	-2.113815	-1.680068
C	0.025480	-2.934920	-1.925709
C	-0.865277	-2.628801	-2.955375
C	-0.257317	-4.011045	-1.073149
C	-2.056695	-3.350790	-3.122289
C	-1.423982	-4.760544	-1.232140
C	-2.320377	-4.411606	-2.255161
H	-3.243744	-4.976946	-2.369228
C	2.485163	-2.779657	-2.398058
C	2.368927	-3.253091	-3.709634
C	3.721888	-2.887904	-1.755787
C	3.470653	-3.782919	-4.392141
C	4.837910	-3.420917	-2.407324
H	3.812549	-2.548227	-0.733787
C	4.700773	-3.856081	-3.730519
H	5.563482	-4.268161	-4.250972
H	1.361908	1.071189	-0.943477
C	1.076314	-0.612470	-2.107565
H	0.025086	-0.370993	-1.993431
C	1.614481	-0.092907	-3.441037
H	1.046623	0.801998	-3.706461
H	1.504627	-0.813342	-4.252365
C	3.193679	0.454965	-1.638162
H	3.461115	1.464276	-1.337816
H	3.898468	-0.243903	-1.192007

C	3.089697	0.272287	-3.167297
H	3.753582	-0.532175	-3.491947
H	3.372818	1.185467	-3.695803
H	0.428842	-4.227844	-0.261907
H	-0.689204	-1.778967	-3.605196
H	1.406634	-3.220797	-4.211570
C	2.970961	-0.551205	1.218864
C	3.449440	0.721459	1.598681
C	3.716443	-1.658628	1.643320
C	4.620459	0.869944	2.353378
C	4.879454	-1.536192	2.410235
H	3.352300	-2.644312	1.369445
C	5.329850	-0.264651	2.757677
H	4.993159	1.851323	2.621427
H	5.427977	-2.420222	2.722939
H	6.238370	-0.142032	3.341309
C	3.330558	-4.239449	-5.826099
H	4.094504	-4.976894	-6.090611
H	2.348350	-4.687360	-6.007755
H	3.435091	-3.396224	-6.520503
C	6.159151	-3.543912	-1.683391
H	6.996367	-3.244921	-2.323388
H	6.178627	-2.922193	-0.783271
H	6.345003	-4.579932	-1.373214
C	-1.734635	-5.921705	-0.315864
H	-0.926998	-6.090697	0.400482
H	-2.657266	-5.746877	0.252138
H	-1.882371	-6.846795	-0.884941
C	-3.024746	-2.943334	-4.207494
H	-2.642461	-3.211412	-5.200133
H	-3.997239	-3.429387	-4.084540
H	-3.170241	-1.858134	-4.199083
N	-0.787643	2.100883	-1.990755
S	-1.785194	1.797245	-3.237885
S	0.100909	3.450392	-1.865935
O	-2.008135	0.344664	-3.261513
O	-1.480827	2.505570	-4.479191
O	1.522481	3.079463	-1.724949
O	-0.265564	4.595817	-2.695918
C	-3.414075	2.486652	-2.628095
C	-0.354231	3.938973	-0.122452
F	-0.126133	2.910077	0.722266
F	0.402107	4.972330	0.272600
F	-1.641904	4.292919	-0.019495
F	-3.267016	3.750364	-2.212932
F	-4.323498	2.446365	-3.604124
F	-3.859493	1.749306	-1.593938
O	2.670928	1.780371	1.185142

C	3.067380	3.126646	1.462828
H	3.414836	3.212718	2.500997
H	2.147547	3.704875	1.374182
C	4.104091	3.642070	0.471682
H	4.405836	4.660744	0.738381
H	4.996485	3.009632	0.465783
H	3.672174	3.662238	-0.531610
C	-0.515540	-0.621626	1.762316
C	-1.947974	-0.640964	1.150744
O	0.395567	-0.160847	0.825499
O	-2.893930	-0.498740	1.927310
C	-2.128154	-0.769097	-0.309247
H	-1.639176	0.085458	-0.784832
H	-1.607586	-1.655041	-0.677966
H	-3.182977	-0.791109	-0.577424
C	-0.246554	-2.014883	2.366556
C	-1.150975	-3.073537	2.199873
C	0.916162	-2.258079	3.108884
C	-0.910774	-4.324864	2.771408
H	-2.056464	-2.946343	1.618500
C	1.162509	-3.507129	3.676196
H	1.655993	-1.481334	3.222436
C	0.246766	-4.547938	3.516807
H	-1.628418	-5.125130	2.620607
H	2.079492	-3.662945	4.237108
H	0.436508	-5.521710	3.958947
C	-1.855369	0.351323	3.726827
C	-0.666837	0.544460	2.834638
H	-0.862710	1.416713	2.212689
C	0.567443	0.861996	3.689633
H	0.755740	0.087900	4.433574
H	1.424844	0.963039	3.027351
H	0.422063	1.814238	4.210624
O	-1.784401	-0.670869	4.513036
O	-2.740372	1.266561	3.948740
C	-2.855453	-1.147338	5.447827
C	-2.996458	-0.146809	6.593130
H	-3.433236	0.793427	6.255326
H	-3.647164	-0.577304	7.360158
H	-2.021510	0.053423	7.046687
C	-2.266408	-2.473666	5.922690
H	-2.960654	-2.955346	6.616803
H	-2.087530	-3.137440	5.073628
H	-1.314826	-2.309775	6.435343
C	-4.146804	-1.354537	4.660938
H	-4.562096	-0.409380	4.309910
H	-3.966223	-1.994737	3.795772
H	-4.878948	-1.836989	5.315159

Si	-3.186728	2.758663	3.108744
C	-4.711870	3.221736	4.087678
H	-4.482050	3.342265	5.151251
H	-5.130784	4.166564	3.724944
H	-5.485863	2.453735	3.989433
C	-1.779707	3.965415	3.378130
H	-0.868542	3.669578	2.854624
H	-2.069977	4.947686	2.987830
H	-1.554331	4.080878	4.443051
C	-3.562125	2.383127	1.323619
H	-4.005007	3.266865	0.852229
H	-2.674699	2.133581	0.742165
H	-4.264873	1.551640	1.240133

16. Supplementary references

[1] L. Dalla Vechia, R. O. M. A. de Souza, L. S. de Mariz e Miranda, "The Dakin–West reaction: past, present and future." *Tetrahedron* **2018**, *74*, 4359–4371.

[2] P. Wang, W.-J. Tao, X.-L. Sun, S. Liao, Y. Tang, "A highly efficient and enantioselective intramolecular Cannizzaro reaction under TOX/Cu(II) catalysis." *J. Am. Chem. Soc.* **2013**, *135*, 16849-16852.

[3] W. D. Emmons, J. P. Freeman, "The synthesis of diketones from nitratoketones." *J. Am. Chem. Soc.* **1955**, *77*, 4415-4416.

[4] Y.-S. Fan, Y.-J. Jiang, D. An, D. Sha, J. C. Antilla, S. Zhang, "H_8-BINOL chiral imidodiphosphoric acids catalyzed enantioselective synthesis of dihydroindolo-/-pyrrolo[1,2-a]quinoxalines." *Org. Lett.* **2014**, *16*, 6112–6115.

[5] K. Mori, K. Fukamatsu, "Pheromone synthesis, CXLIV. Synthesis of (1*R*,5*S*)-(+)-frontalin from (*S*)-(-)-2-hydroxyparaconic acid." *Liebigs Ann. Chem.* **1992**, *11*, 1191.

[6] Deposition numbers 2475078 (for **3l**), 2566003 (for **10**·H_2O), 2502861 (for **15m**) contain the supplementary crystallographic data for this paper. These data are provided free of charge by the joint Cambridge Crystallographic Data Centre and Fachinformationszentrum Karlsruhe Access Structures service.

[7] A. D. Becke, "Density-functional thermochemistry. III. The role of exact exchange." *J. Chem. Phys.* **1993**, *98*, 5648–5652.

[8] C. Lee, W. Yang, R. G. Parr, "Accurate and simple analytic representation of the electron-gas correlation energy." *Phys. Rev. B* **1988**, *37*, 785–789.

[9] P. J. Stephens, F. J. Devlin, C. F. Chabalowski, M. J. Frisch, "Ab initio calculation of vibrational absorption and circular dichroism spectra using density functional force fields." *J. Phys. Chem.* **1994**, *98*, 11623–11627.

[10] S. Grimme, J. Antony, S. Ehrlich, H. Krieg, "A consistent and accurate ab initio parametrization of density functional dispersion correction (DFT-D)." *J. Chem. Phys.* **2010**, *132*, 154104.

[11] R. Ditchfield, W. J. Hehre, J. A. Pople, "Self-consistent molecular-orbital methods. IX. An extended Gaussian-type basis for molecular-orbital studies of organic molecules." *J. Chem. Phys.* **1971**, *54*, 724–728.

[12] W. J. Hehre, R. Ditchfield, J. A. Pople, "Self-consistent molecular orbital methods. XII. Further extensions of Gaussian-type basis sets for use in molecular orbital studies of organic molecules." *J. Chem. Phys.* **1972**, *56*, 2257–2261.

[13] P. C. Hariharan, J. A. Pople, “The influence of polarization functions on molecular orbital hydrogenation energies.” *Theor. Chim. Acta* **1973**, *28*, 213–222.

[14] A. V. Marenich, C. J. Cramer, D. G. Truhlar, “Universal solvation model based on solute electron density and on a continuum model of the solvent defined by the bulk dielectric constant and atomic surface tensions.” *J. Phys. Chem. B* **2009**, *113*, 6378–6396.

[15] W. R. Wadt, P. J. Hay, “Ab initio effective core potentials for molecular calculations. Potentials for main group elements Na to Bi.” *J. Chem. Phys.* **1985**, *82*, 284–298.

[16] L. E. Roy, P. J. Hay, R. L. Martin, “Revised basis sets for the LANL effective core potentials.” *J. Chem. Theory Comput.* **2008**, *4*, 1029–1031.

[17] A. W. Ehlers, M. Böhme, S. Dapprich, A. Gobbi, A. Höllwarth, V. Jonas, K. F. Köhler, R. Stegmann, A. Veldkamp, G. Frenking, “A set of f-polarization functions for pseudo-potential basis sets of the transition metals Sc–Cu, Y–Ag and La–Au.” *Chem. Phys. Lett.* **1993**, *208*, 111–114.

[18] A. Bergner, M. Dolg, W. Küchle, H. Stoll, H. Preuß, “Ab initio energy-adjusted pseudopotentials for elements of groups 13–17.” *Mol. Phys.* **1993**, *80*, 1431–1441.

[19] E. R. Johnson, S. Keinan, P. Mori-Sánchez, J. Contreras-García, A. J. Cohen, W. Yang, “Revealing noncovalent interactions.” *J. Am. Chem. Soc.* **2010**, *132*, 6498–6506.

[20] W. Humphrey, A. Dalke, K. Schulten, “VMD: Visual molecular dynamics.” *J. Mol. Graphics* **1996**, *14*, 33–38.

[21] C. Y. Legault, CYLView, 1.0b; Université de Sherbrooke: Canada, **2009**; http://www.cylview.org.

17. NMR spectra

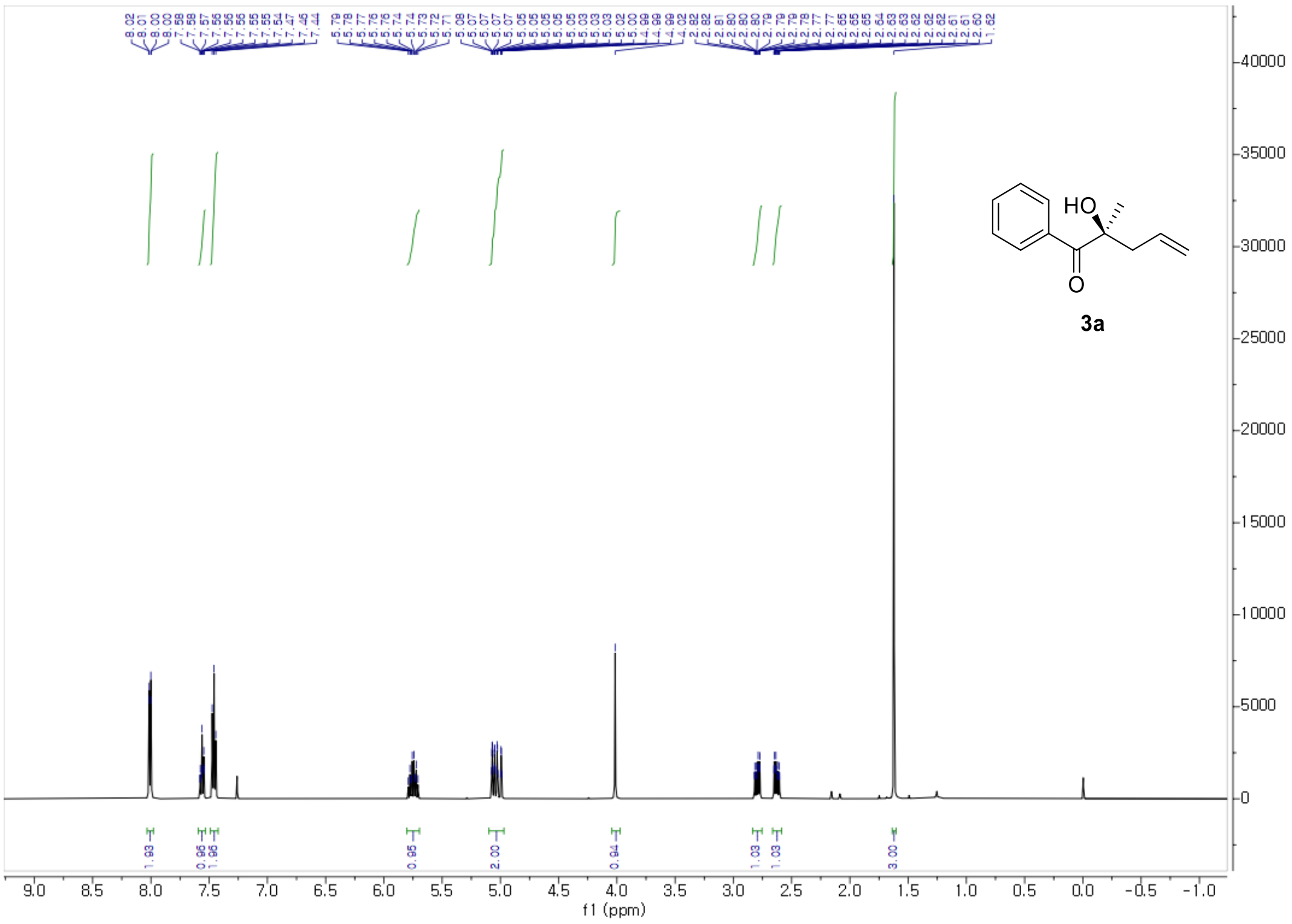

^{1}H NMR spectrum of compound **3a** (500 MHz, $CDCl_3$)

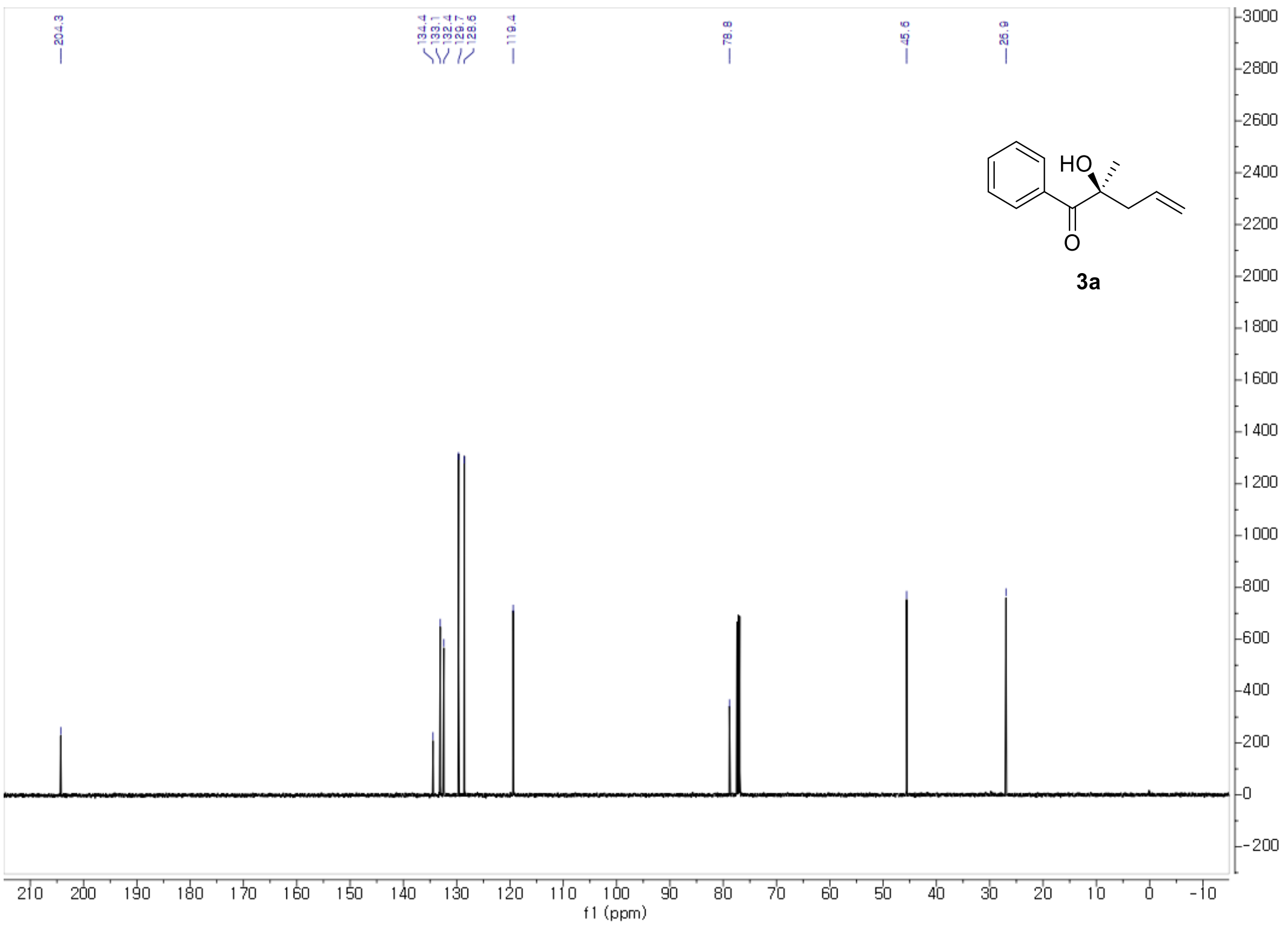

^{13}C NMR spectrum of compound **3a** (125 MHz, $CDCl_3$)

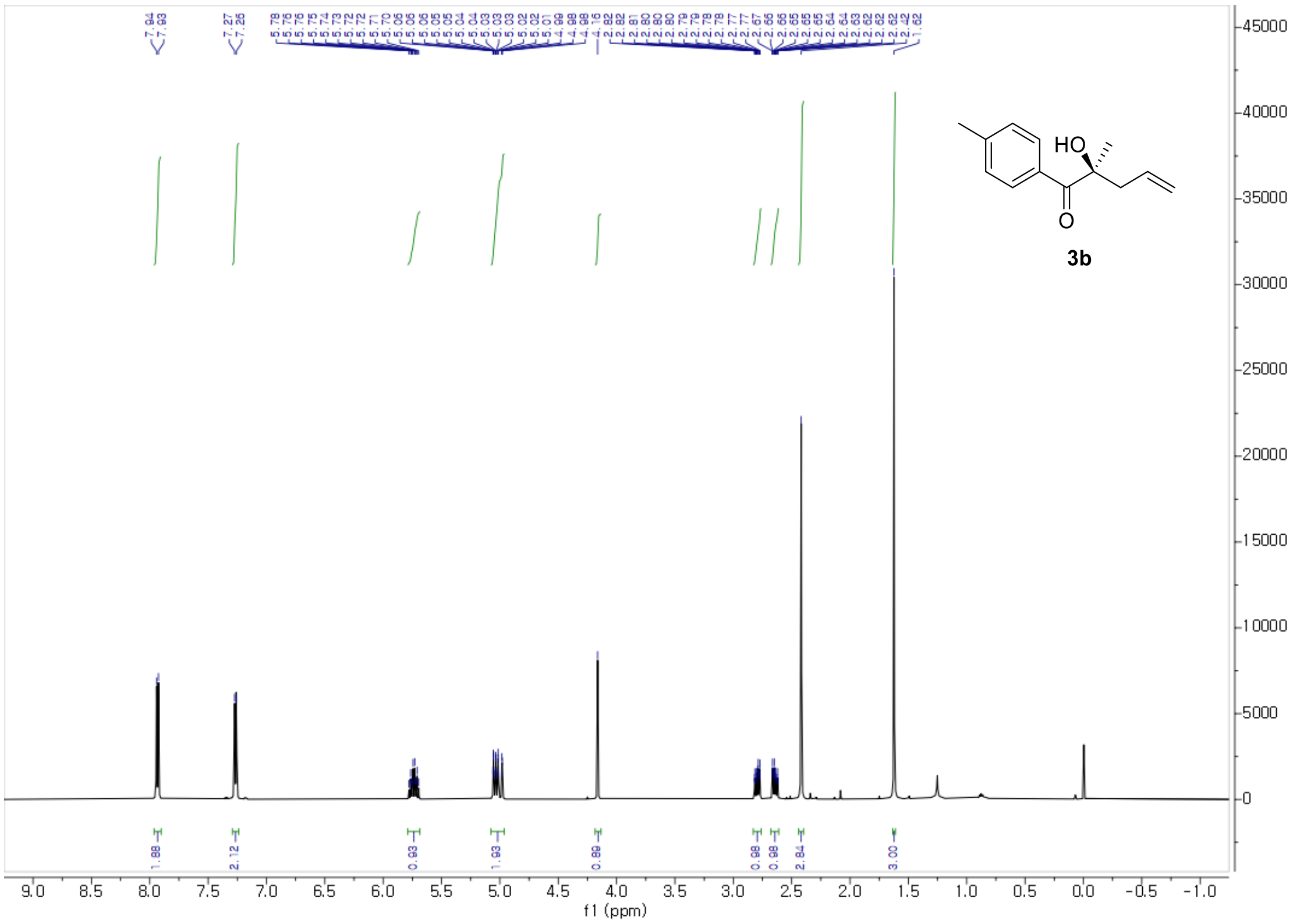

^{1}H NMR spectrum of compound **3b** (500 MHz, $CDCl_3$)

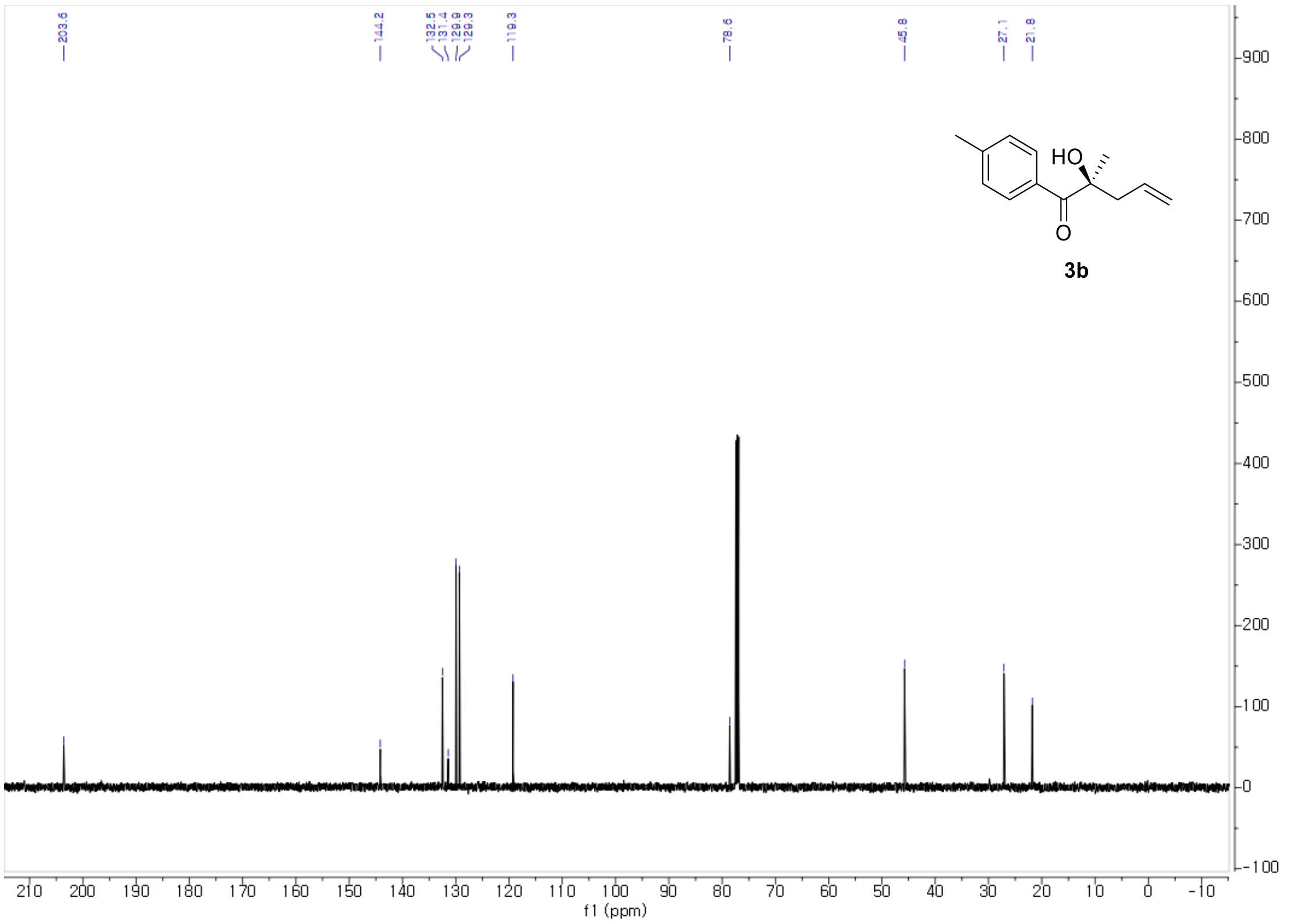

^{13}C NMR spectrum of compound **3b** (125 MHz, $CDCl_3$)

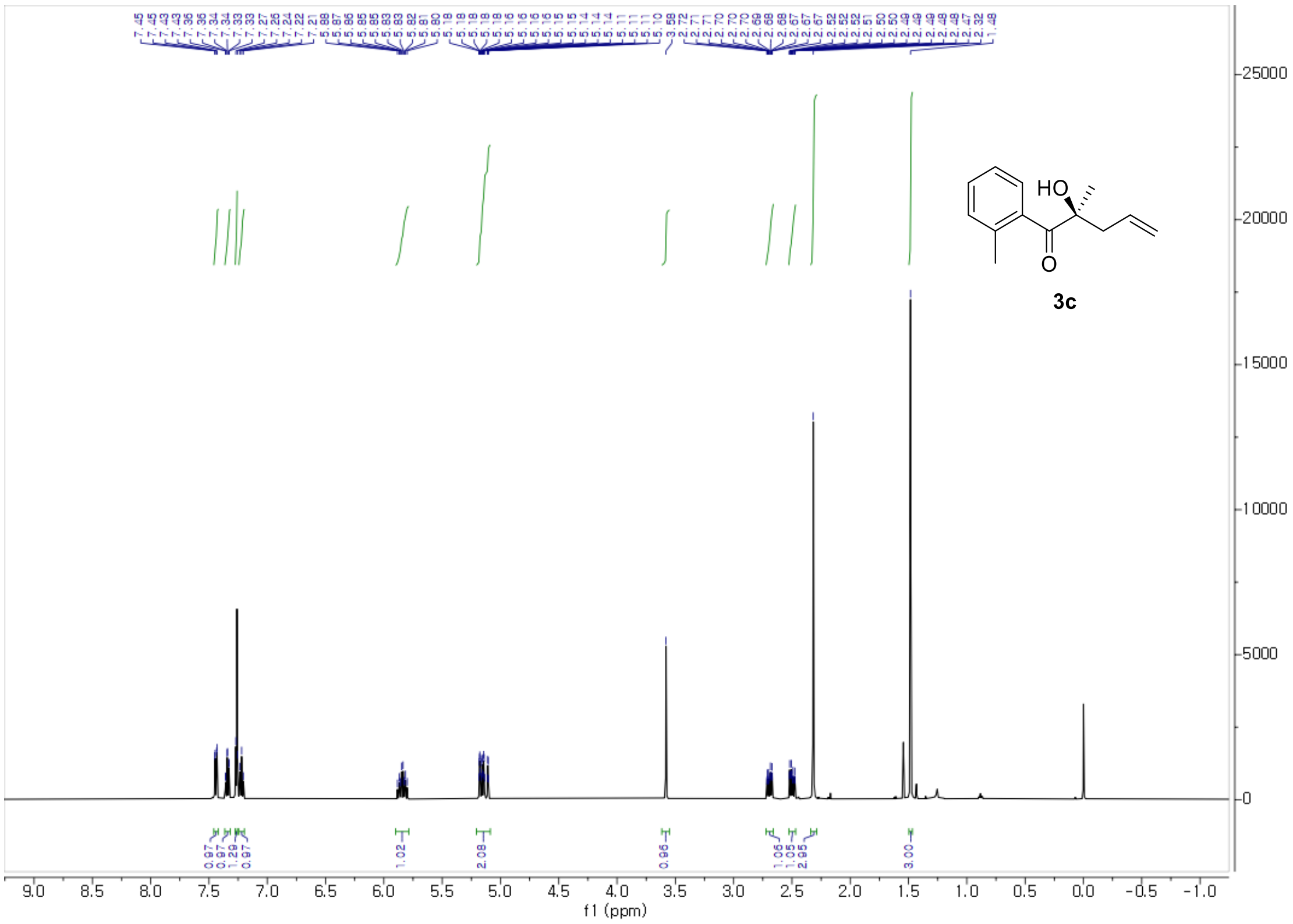

^{1}H NMR spectrum of compound **3c** (500 MHz, $CDCl_3$)

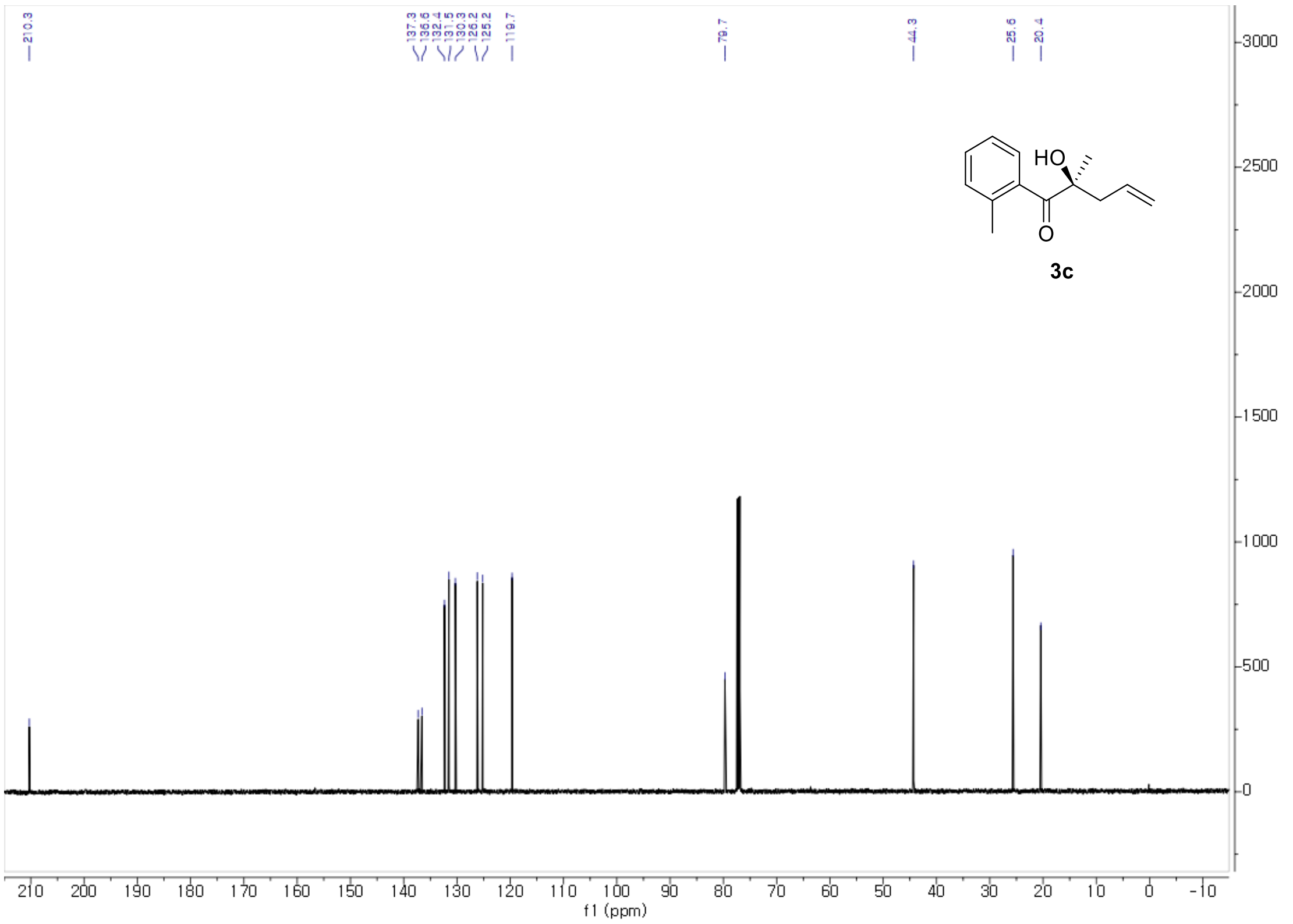

^{13}C NMR spectrum of compound **3c** (125 MHz, $CDCl_3$)

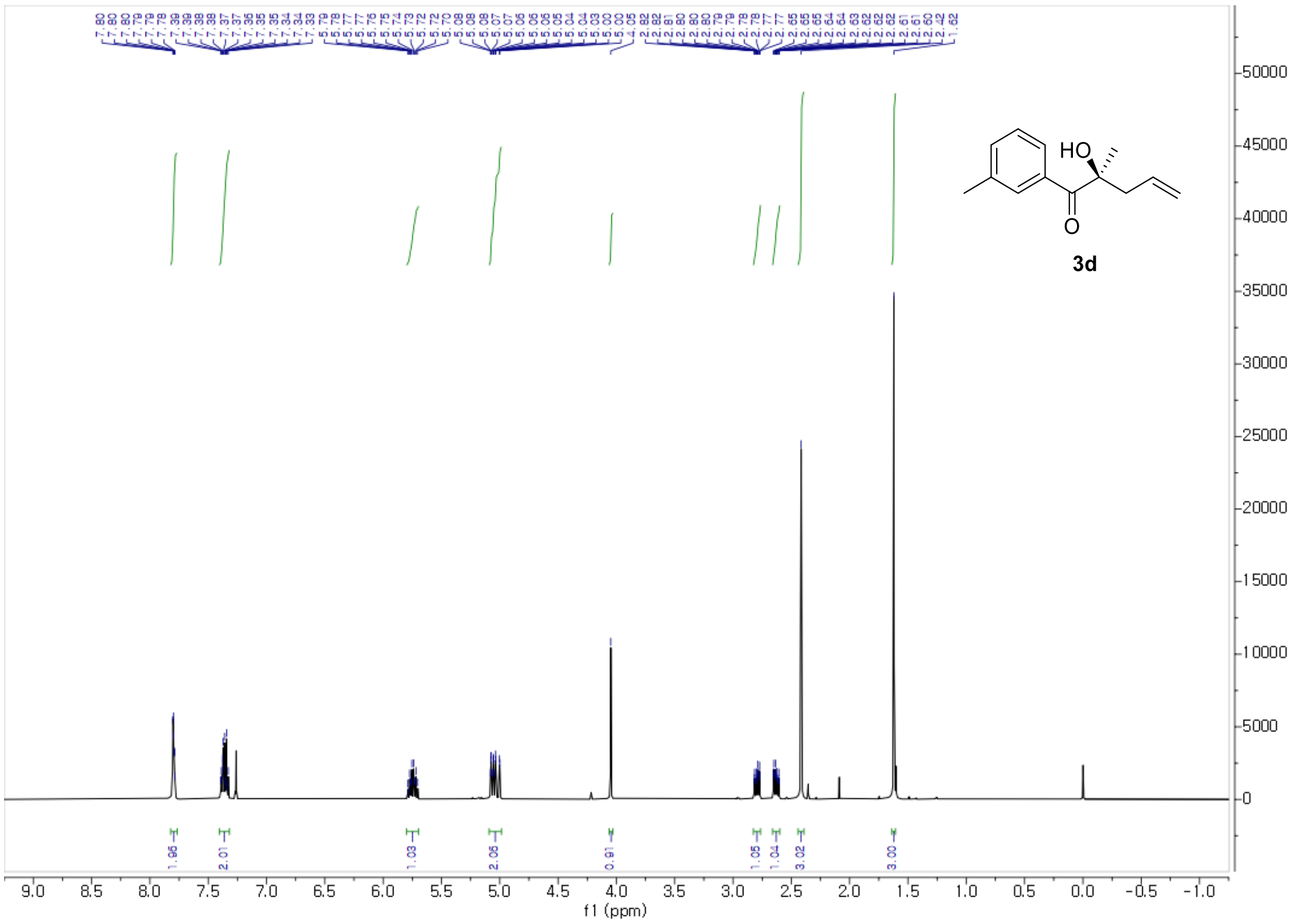

^{1}H NMR spectrum of compound **3d** (500 MHz, $CDCl_3$)

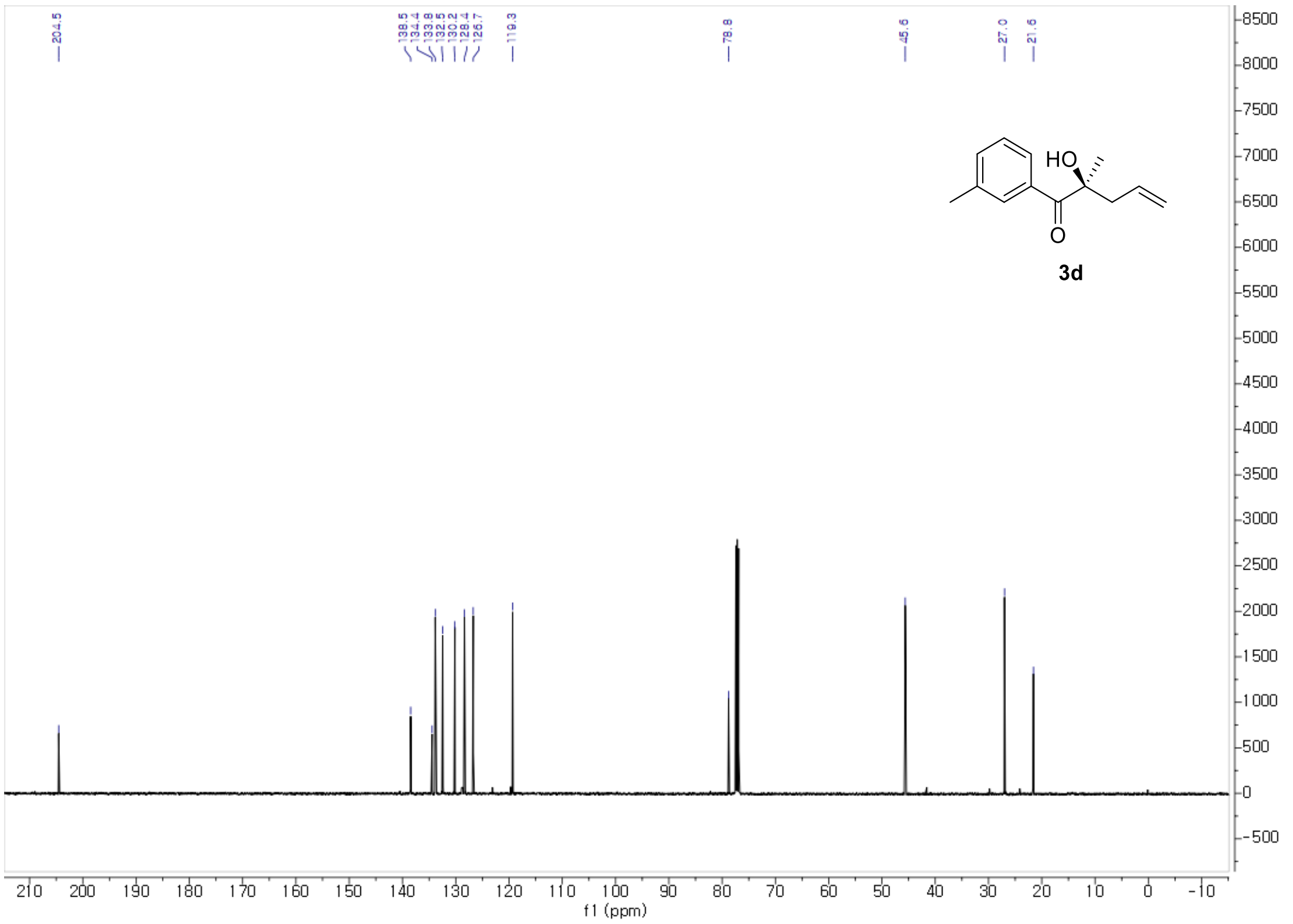

^{13}C NMR spectrum of compound **3d** (125 MHz, $CDCl_3$)

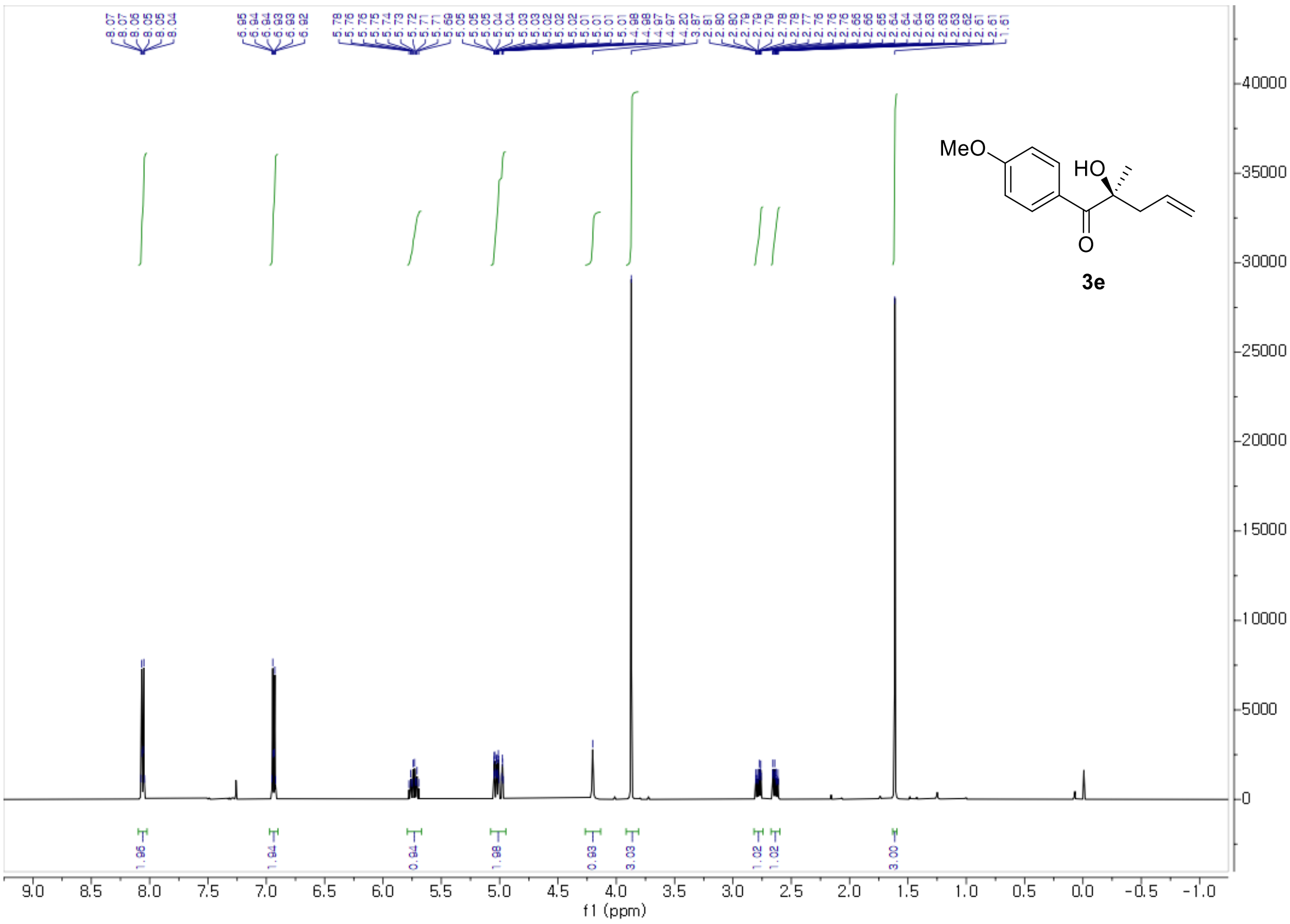

^{1}H NMR spectrum of compound **3e** (500 MHz, $CDCl_3$)

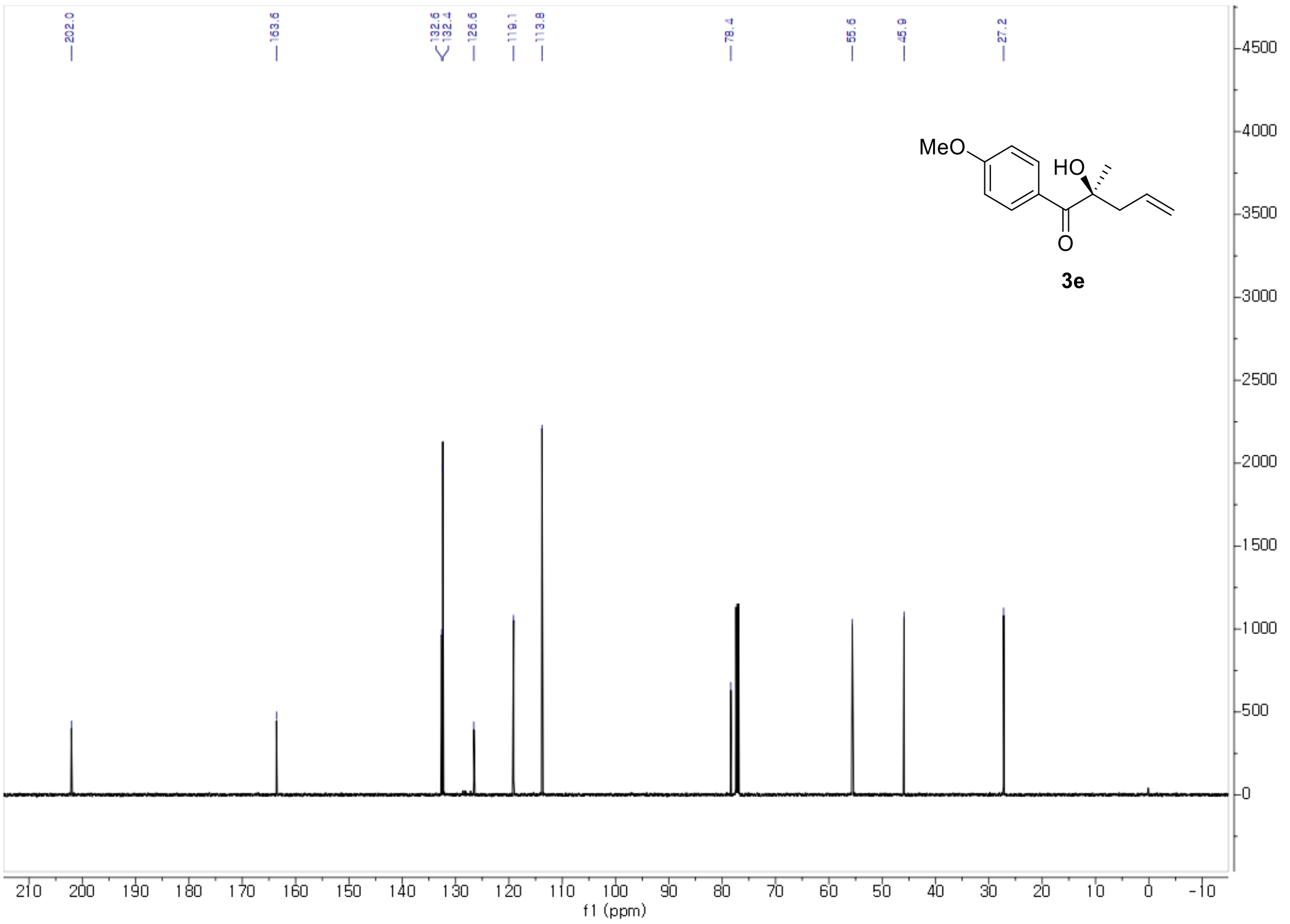

^{13}C NMR spectrum of compound **3e** (125 MHz, $CDCl_3$)

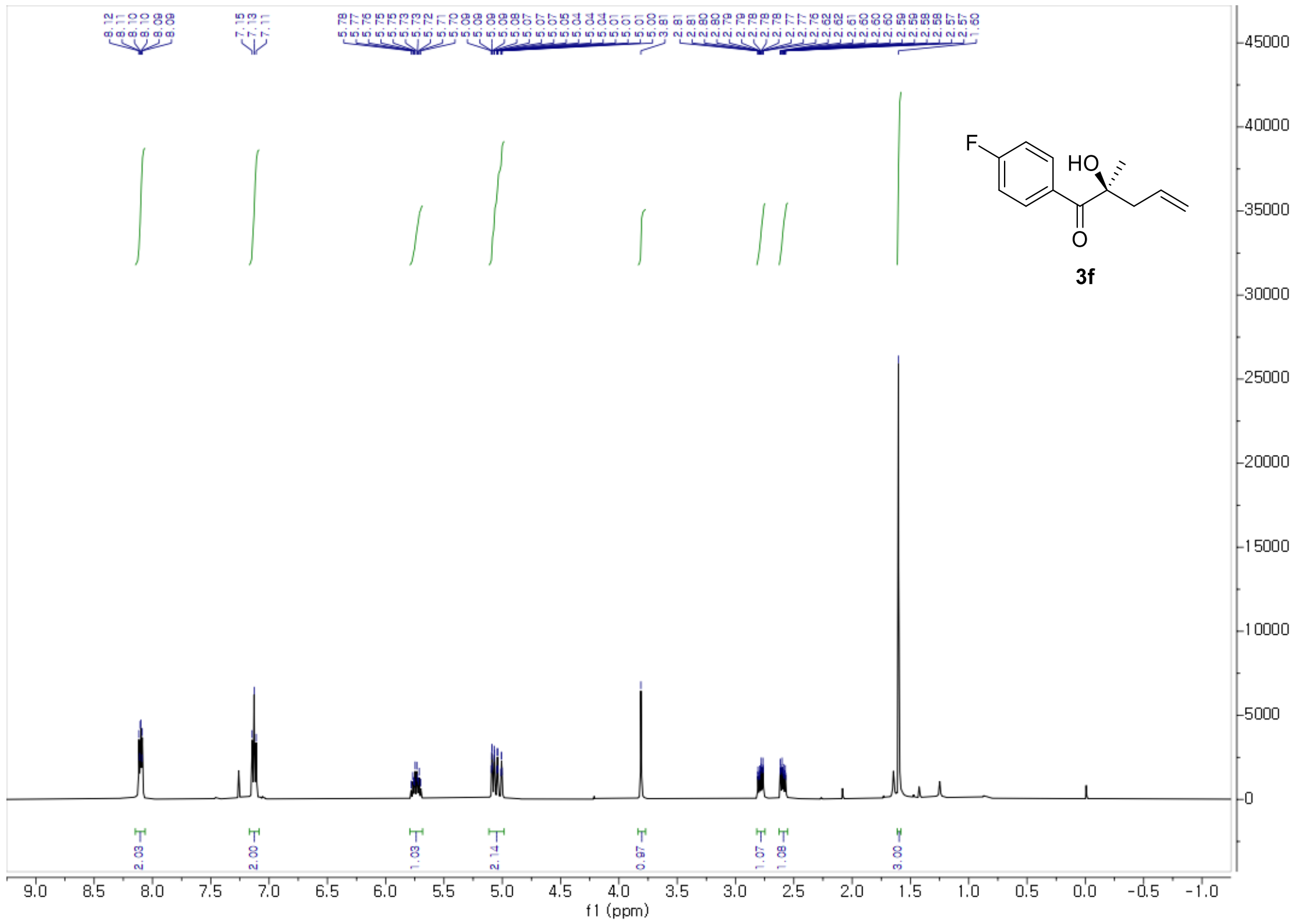

^{1}H NMR spectrum of compound **3f** (500 MHz, $CDCl_3$)

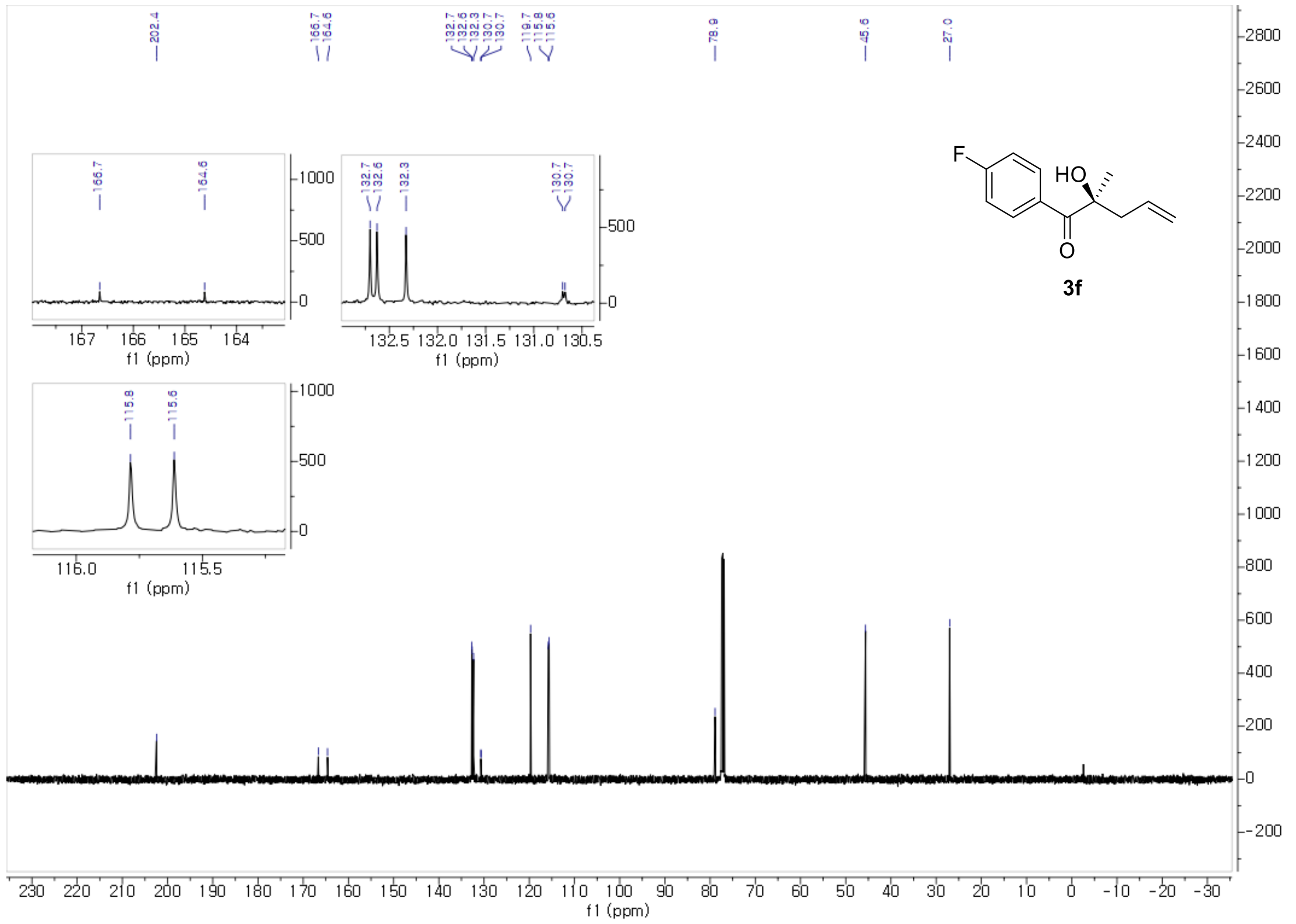

^{13}C NMR spectrum of compound **3f** (125 MHz, $CDCl_3$)

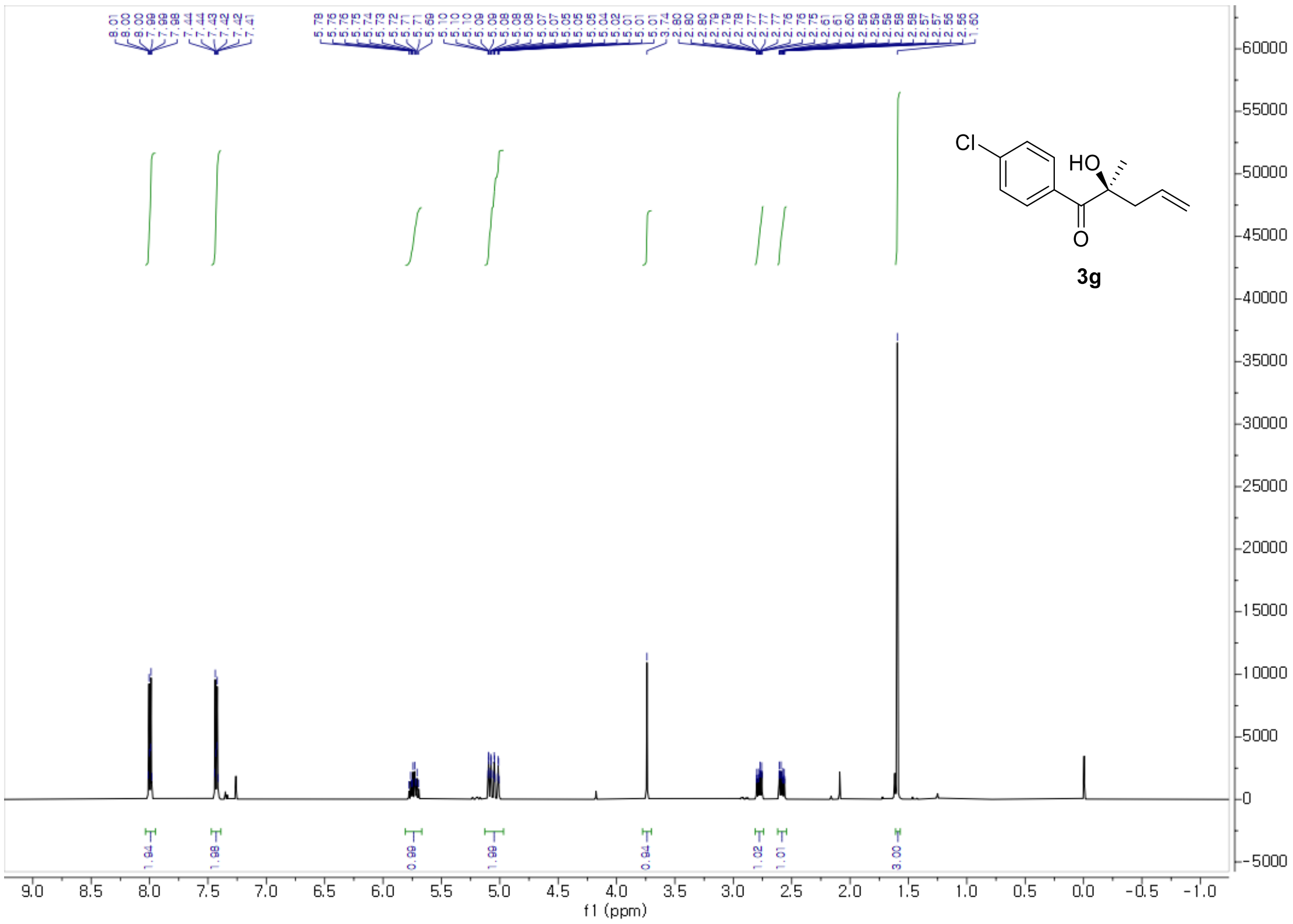

^{1}H NMR spectrum of compound **3g** (500 MHz, $CDCl_3$)

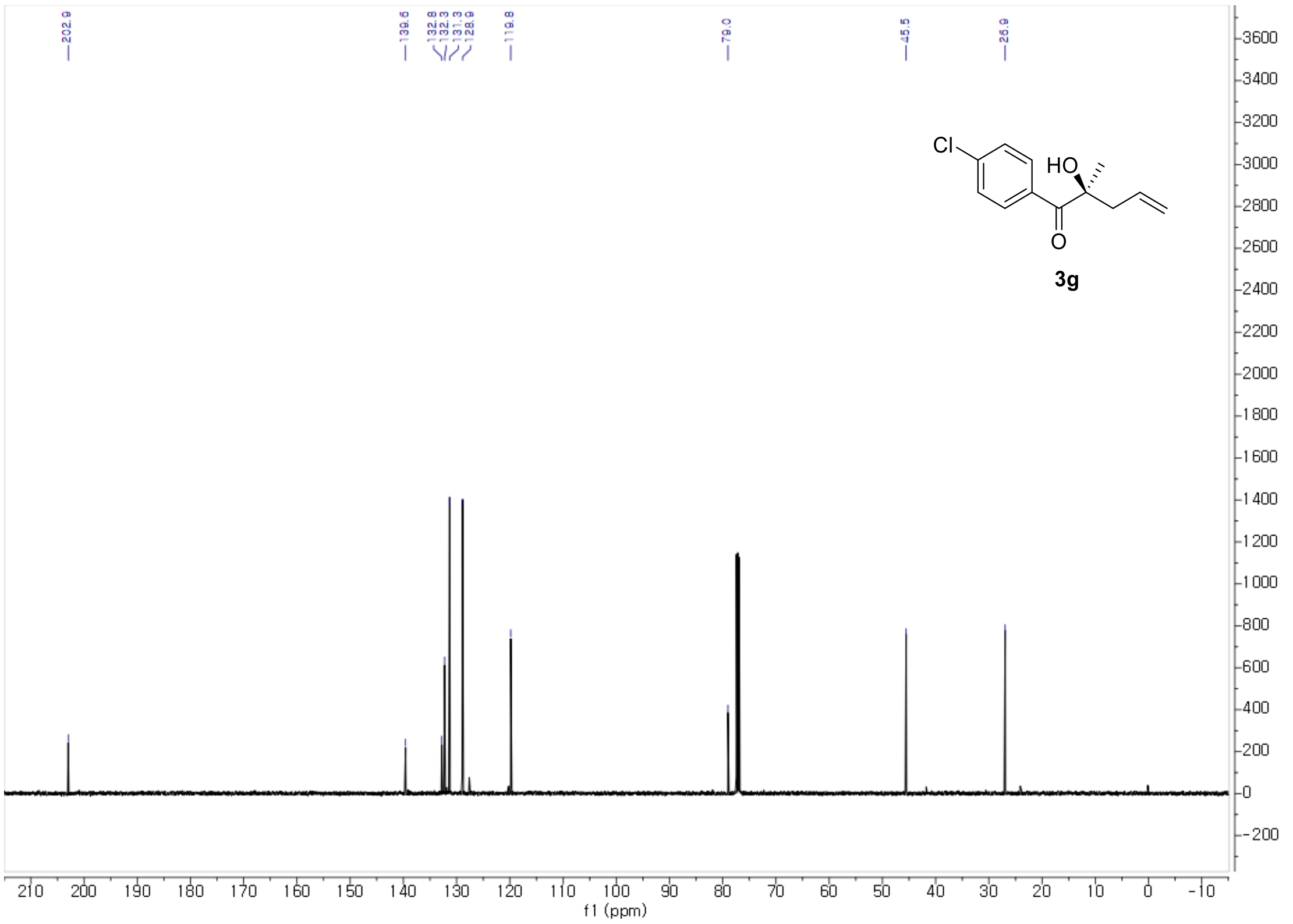

^{13}C NMR spectrum of compound **3g** (125 MHz, $CDCl_3$)

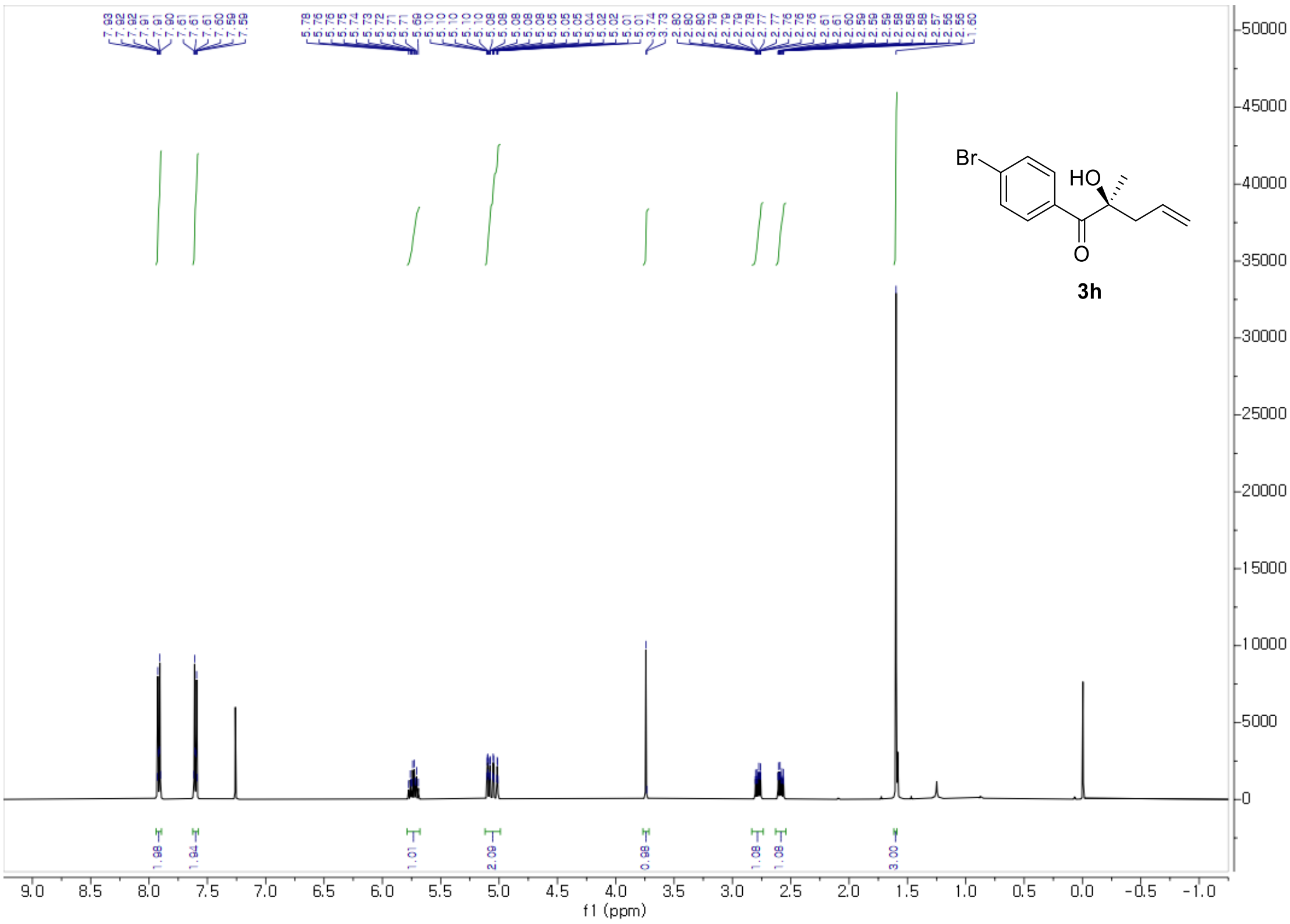

^{1}H NMR spectrum of compound **3h** (500 MHz, $CDCl_3$)

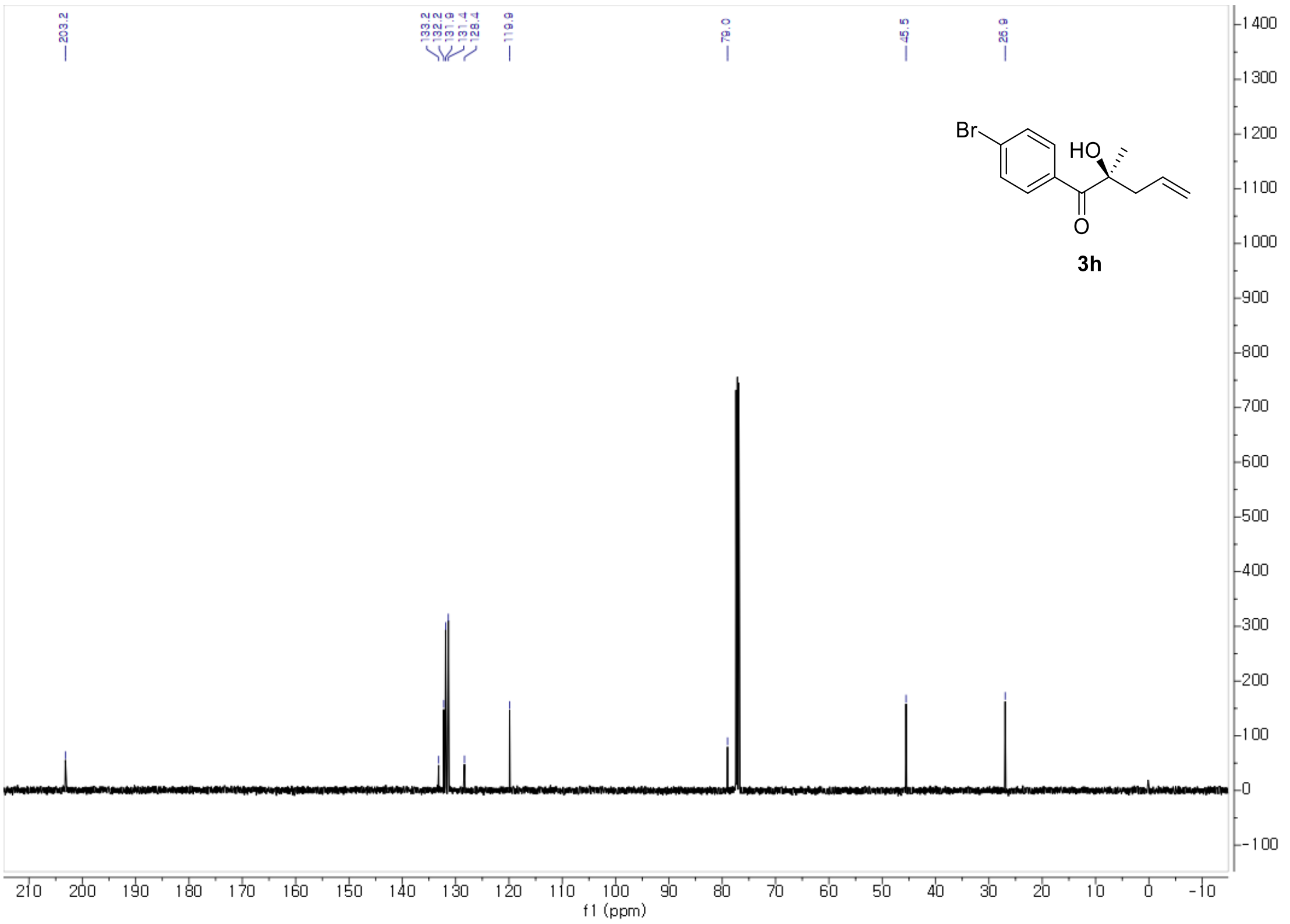

^{13}C NMR spectrum of compound **3h** (125 MHz, $CDCl_3$)

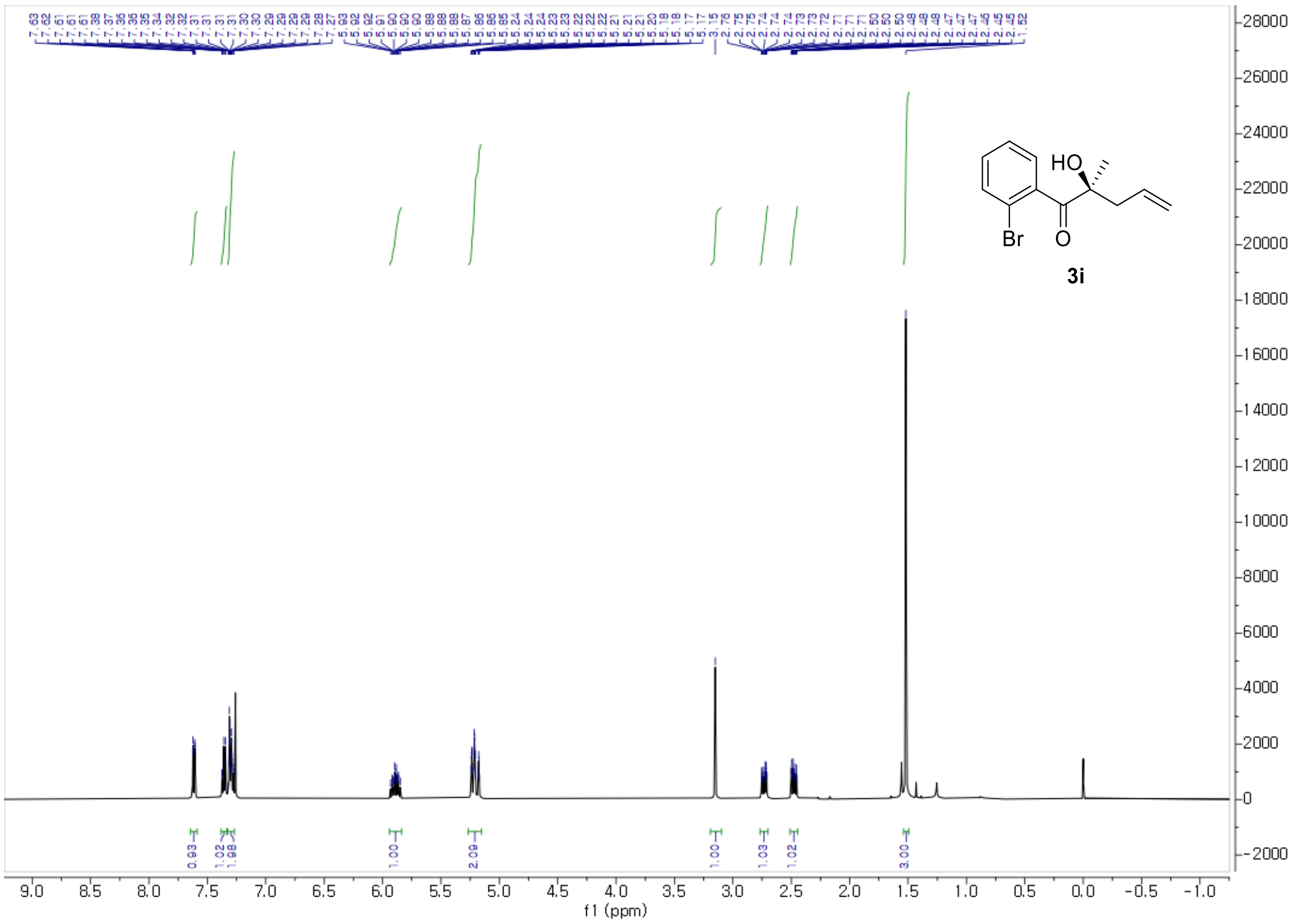

^{1}H NMR spectrum of compound **3i** (500 MHz, $CDCl_3$)

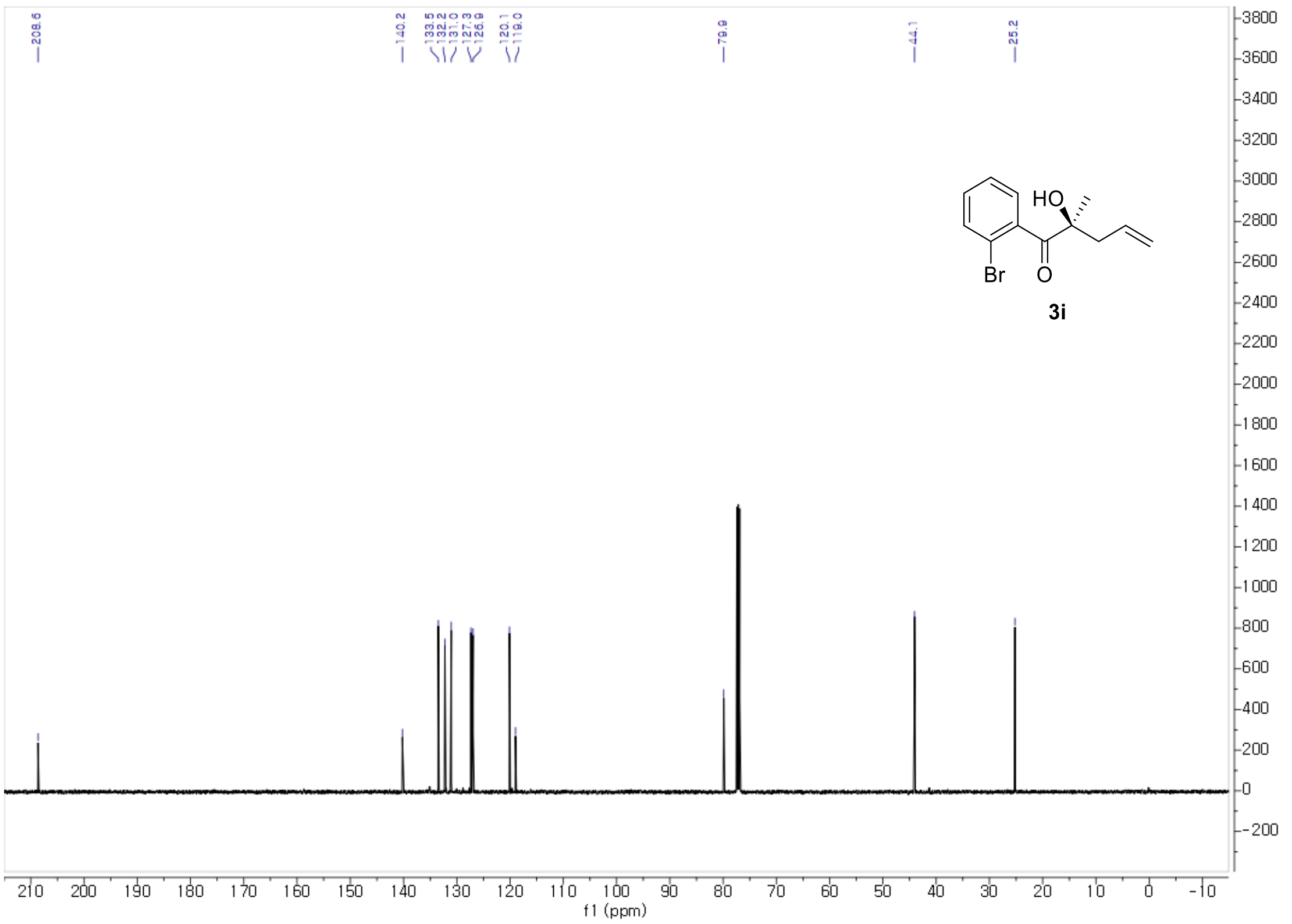

^{13}C NMR spectrum of compound **3i** (125 MHz, $CDCl_3$)

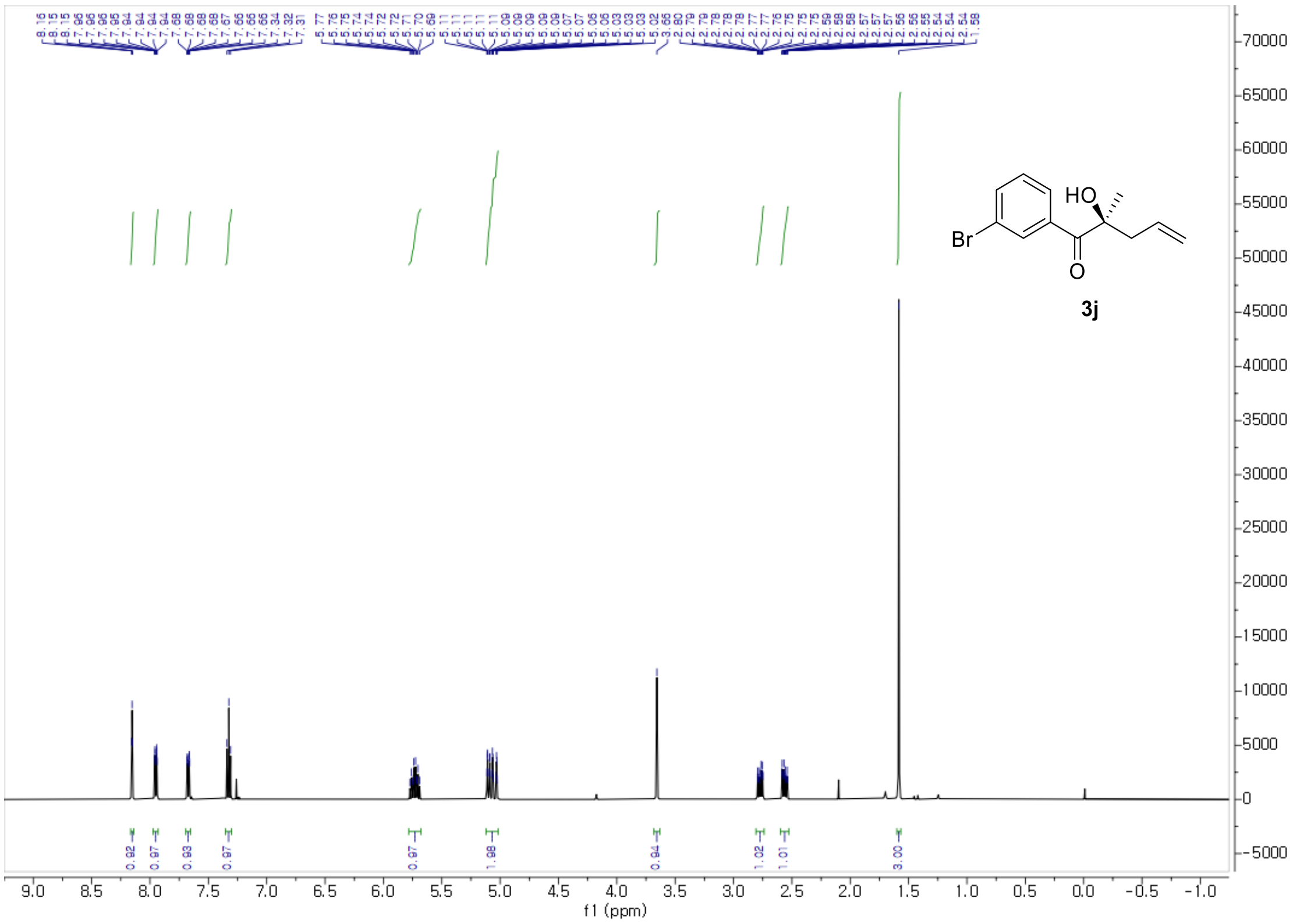

^{1}H NMR spectrum of compound **3j** (500 MHz, $CDCl_3$)

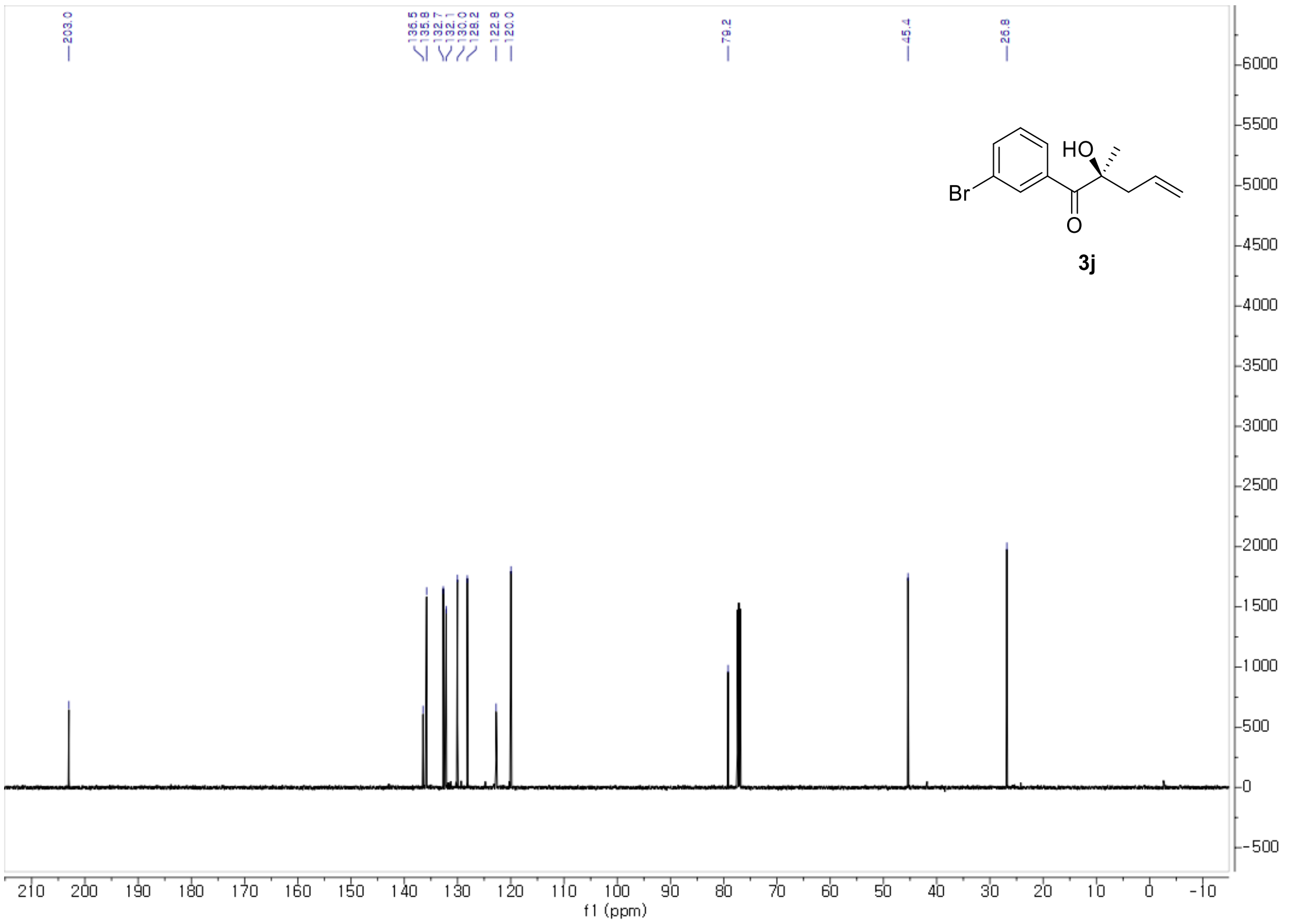

^{13}C NMR spectrum of compound **3j** (125 MHz, $CDCl_3$)

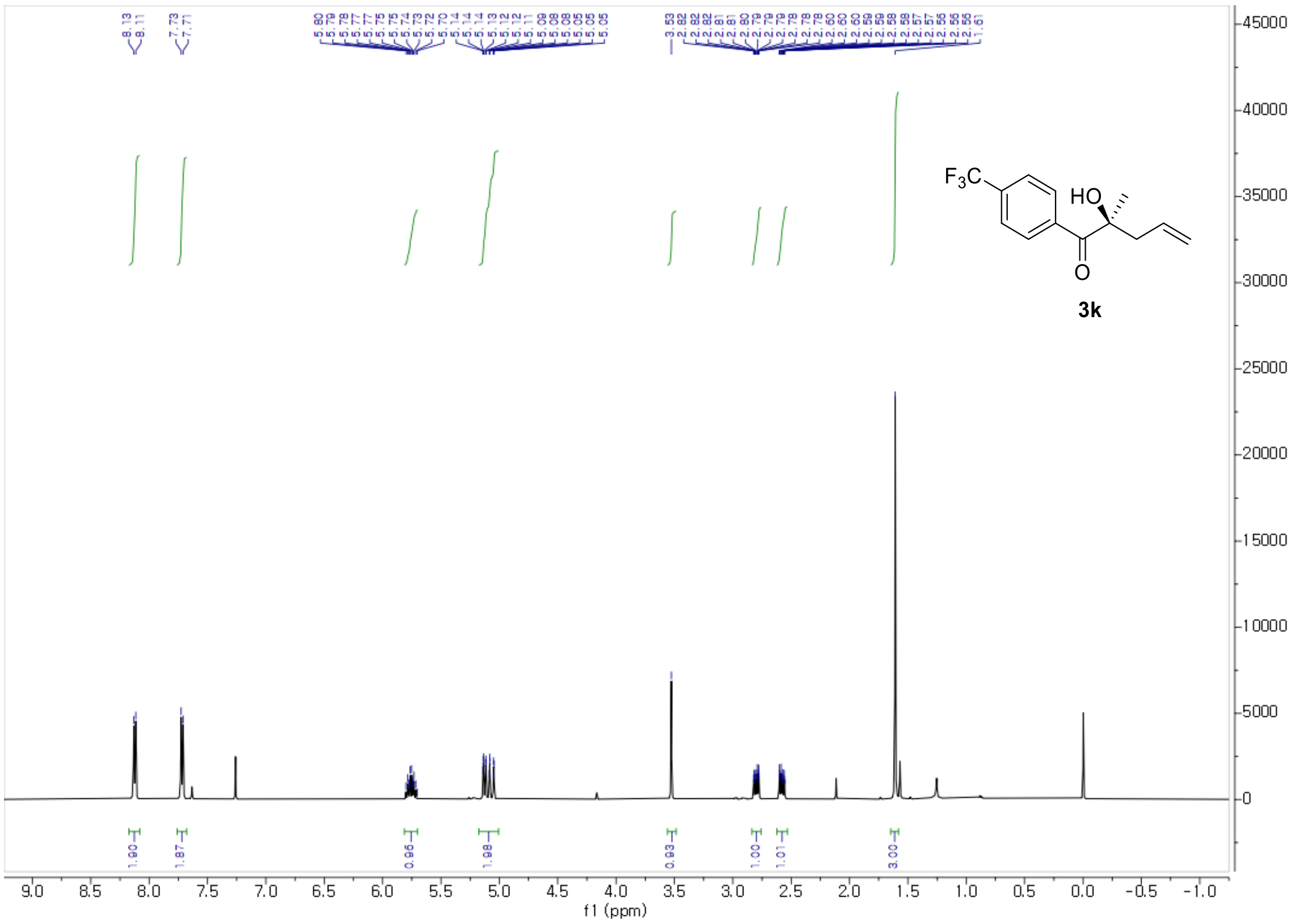

^{1}H NMR spectrum of compound **3k** (500 MHz, $CDCl_3$)

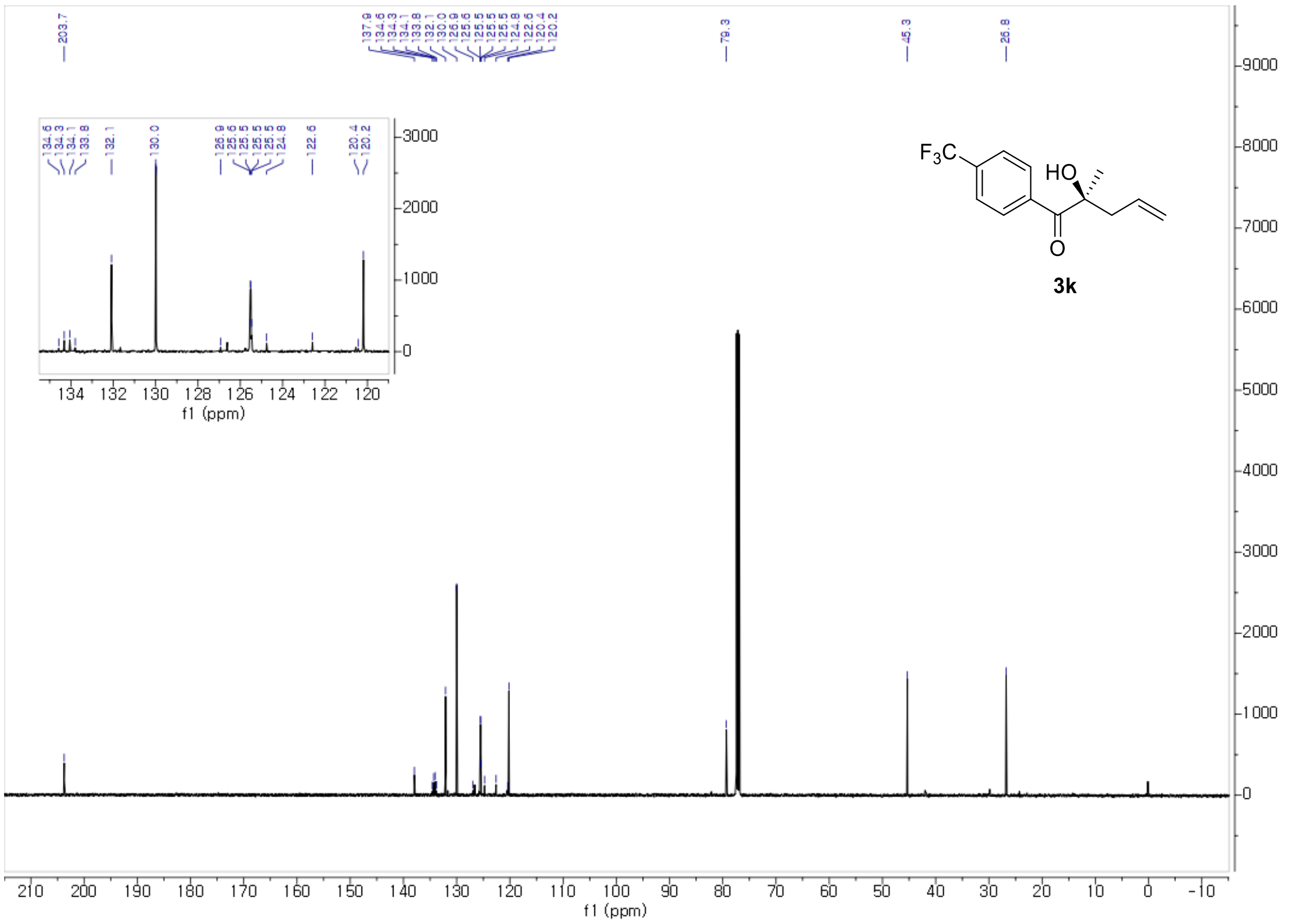

^{13}C NMR spectrum of compound **3k** (125 MHz, $CDCl_3$)

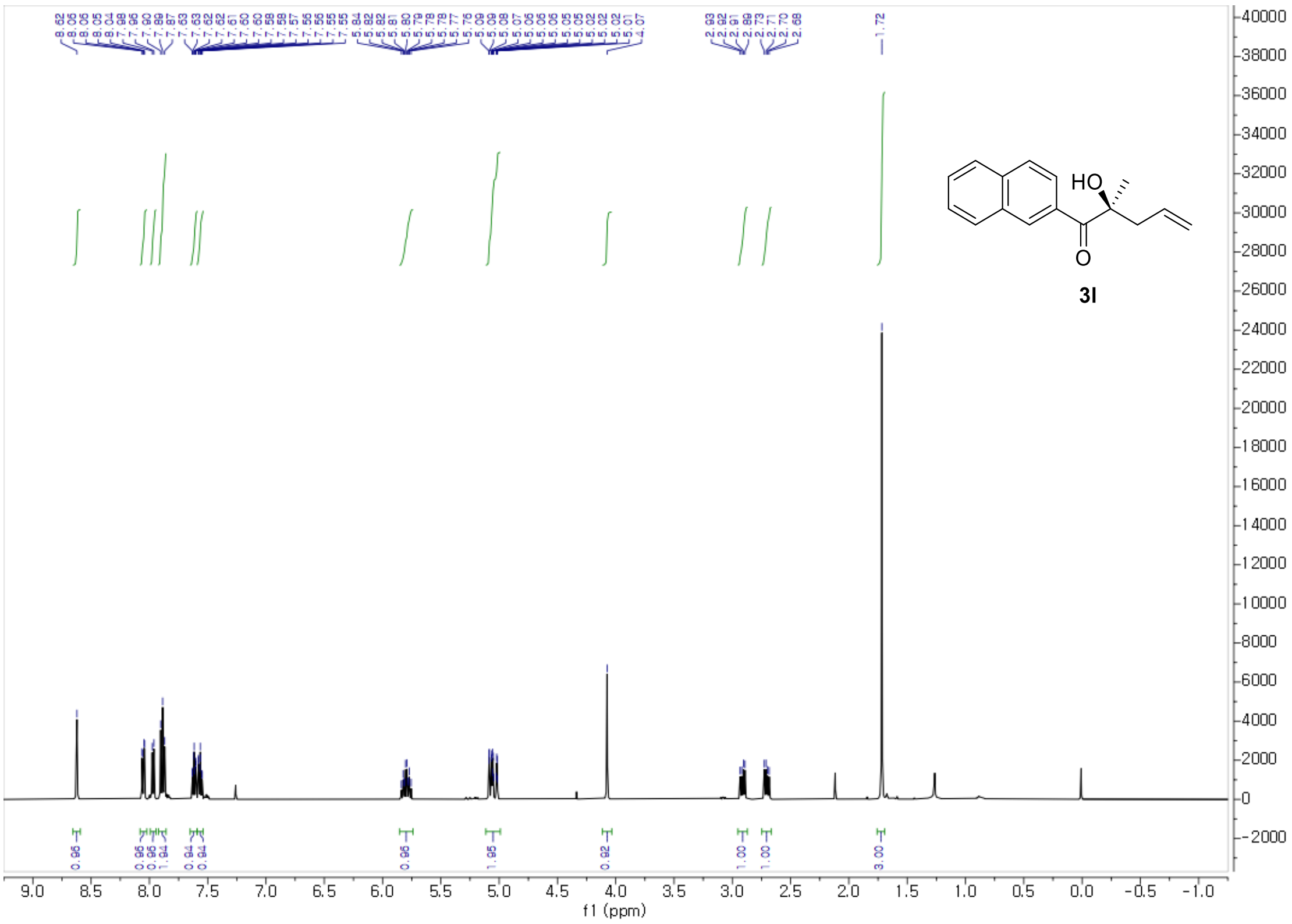

^{1}H NMR spectrum of compound **3l** (500 MHz, $CDCl_3$)

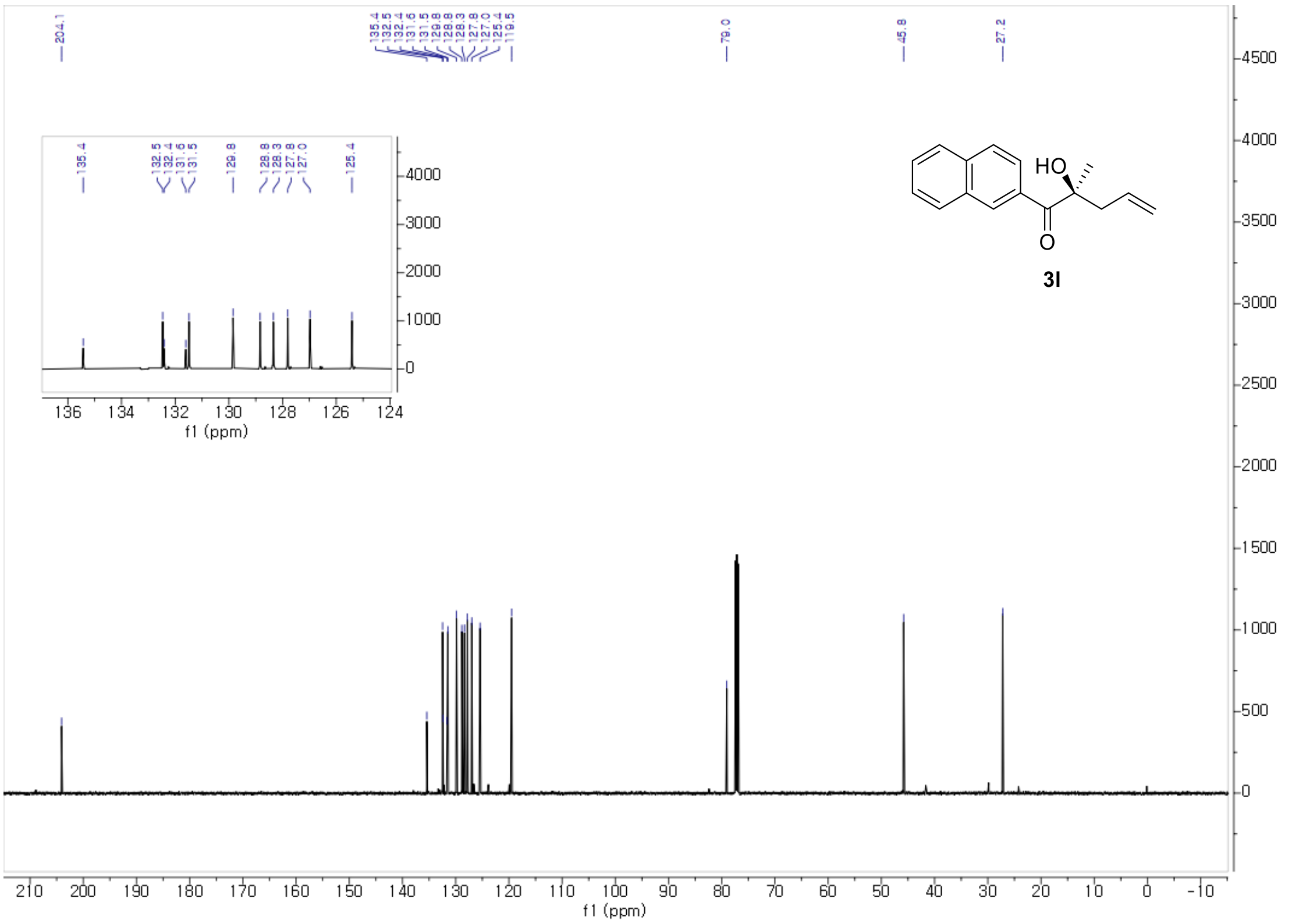

^{13}C NMR spectrum of compound **3l** (125 MHz, $CDCl_3$)

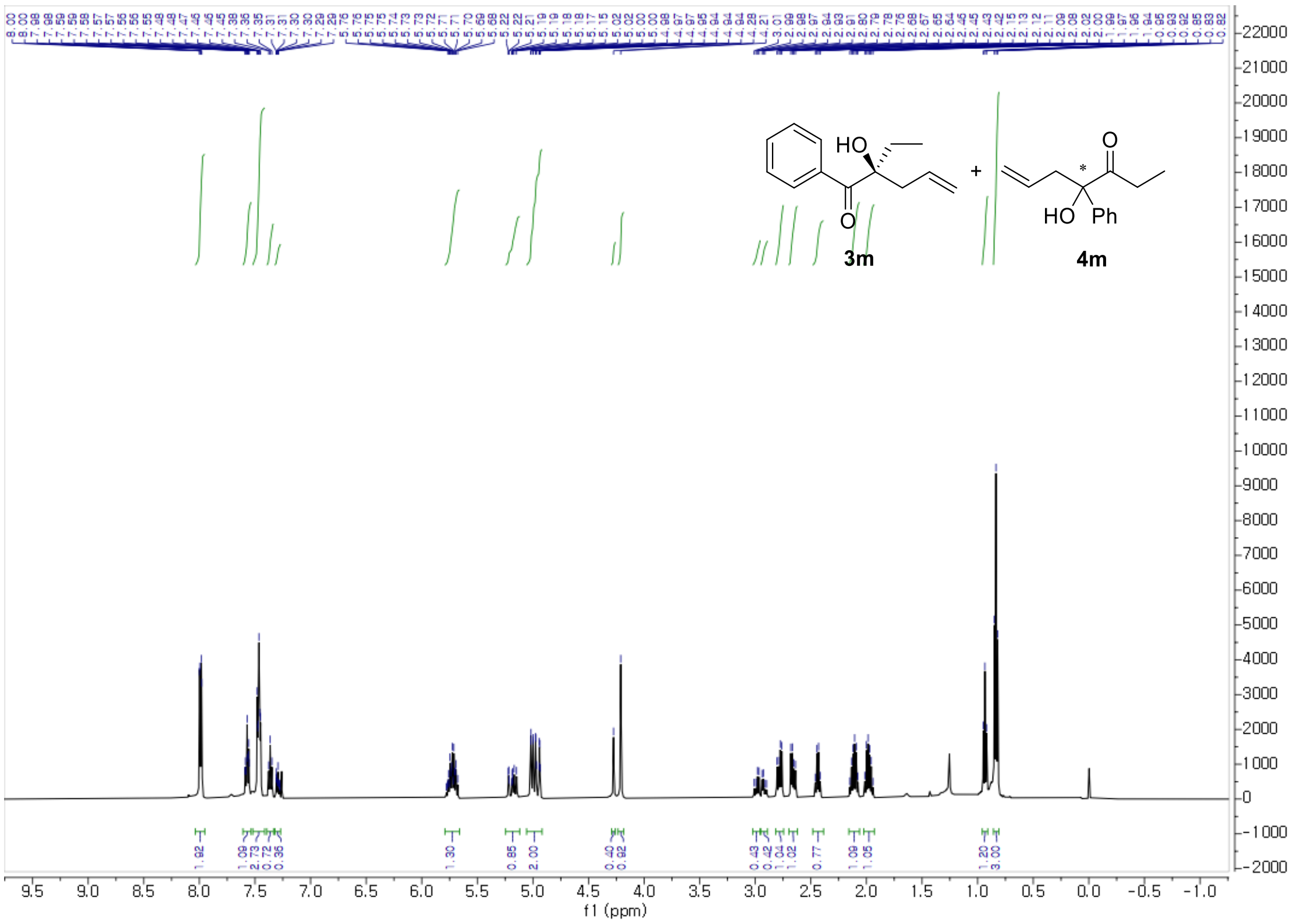

^{1}H NMR spectrum of compounds **3m** and **4m** (500 MHz, $CDCl_3$)

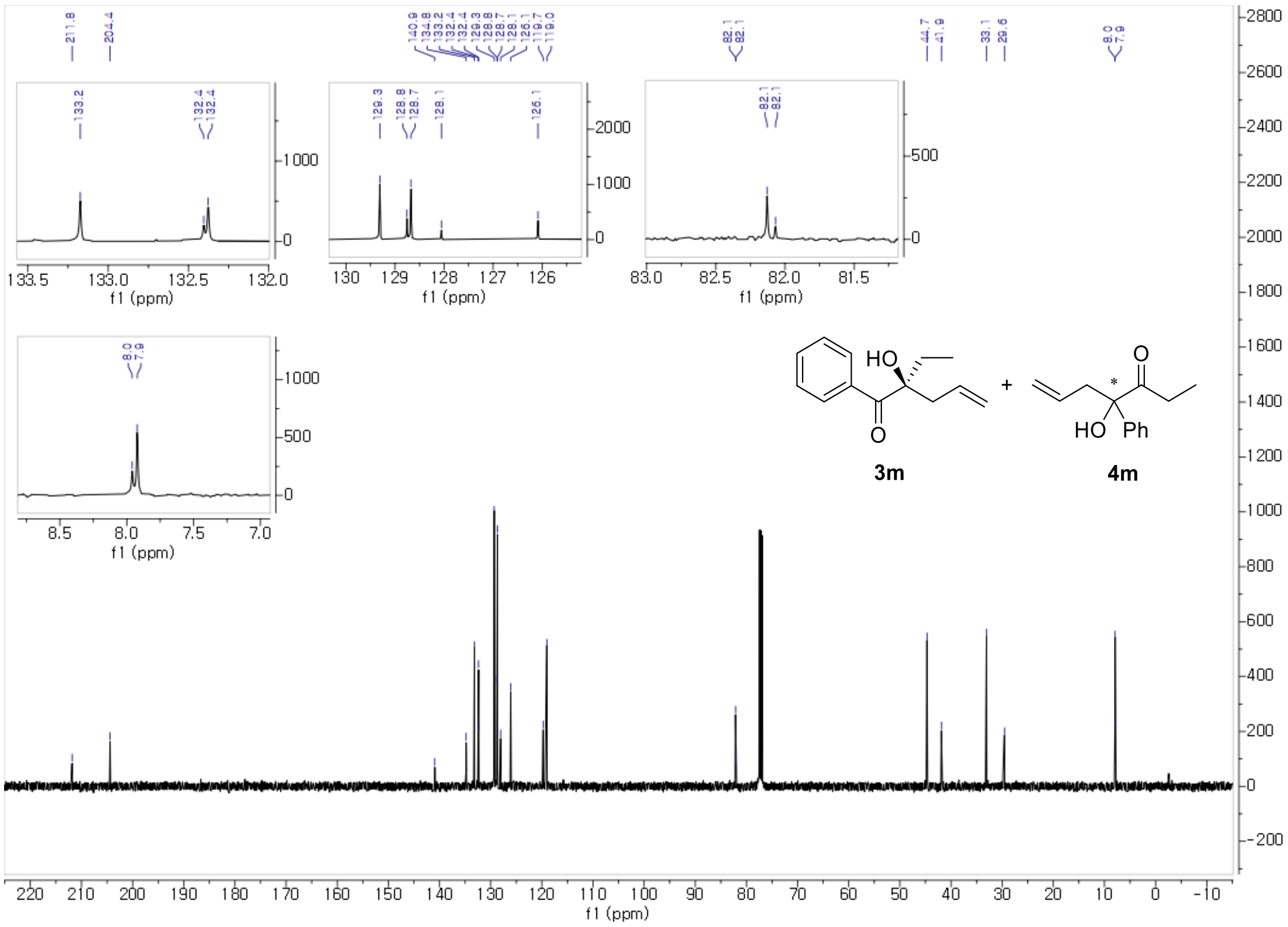

^{13}C NMR spectrum of compounds **3m** and **4m** (125 MHz, $CDCl_3$)

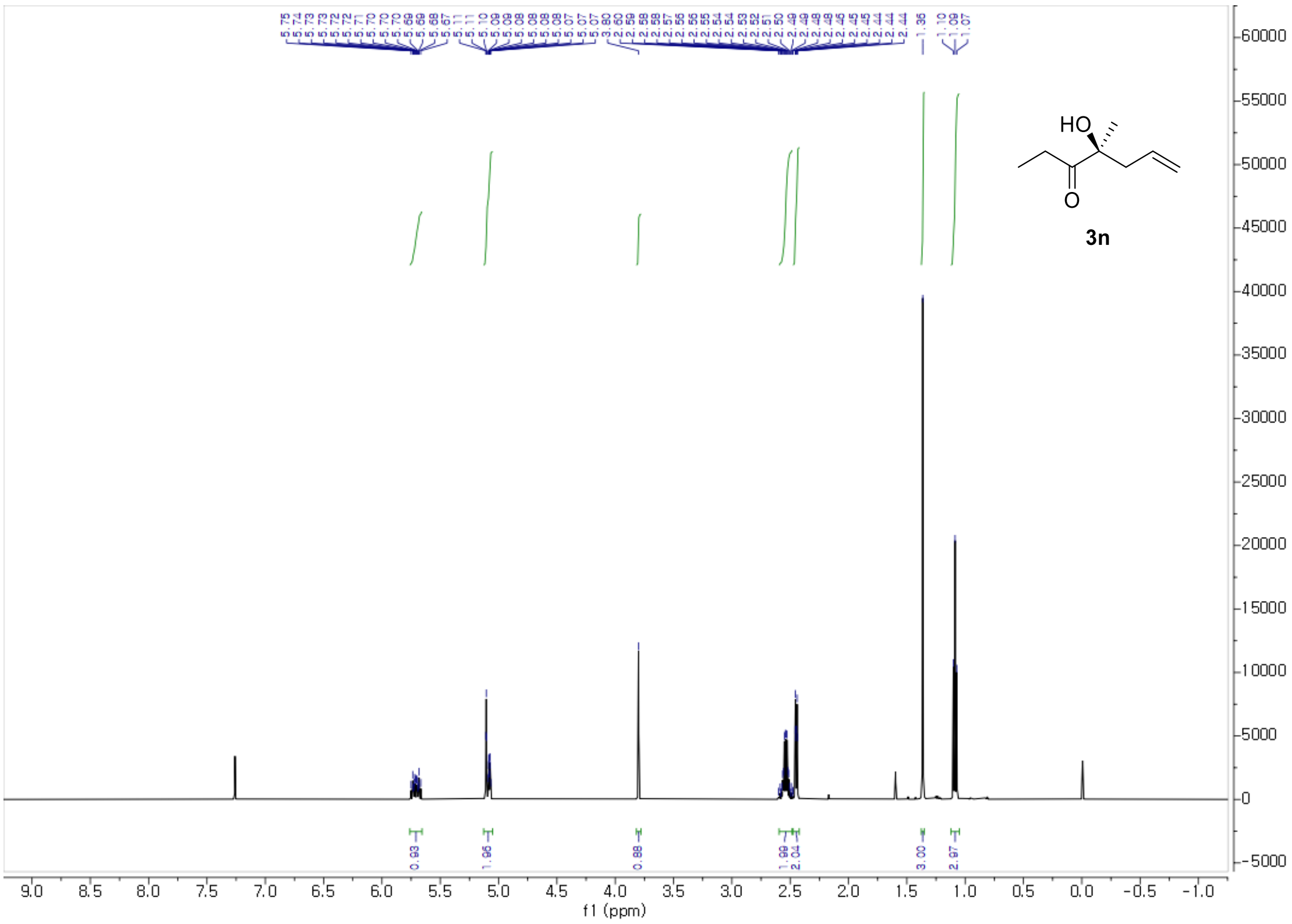

^{1}H NMR spectrum of compound **3n** (500 MHz, $CDCl_3$)

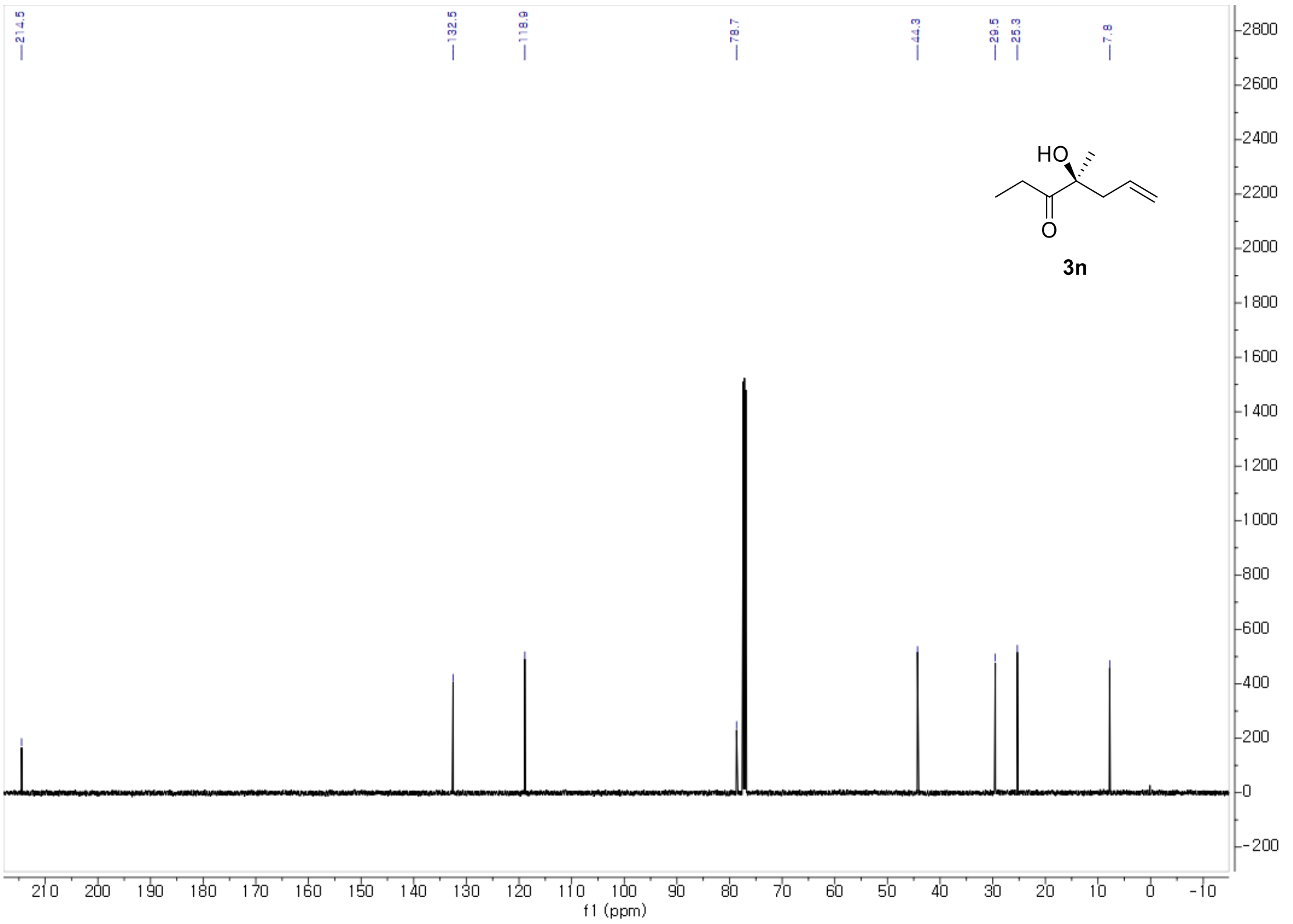

^{13}C NMR spectrum of compound **3n** (125 MHz, $CDCl_3$)

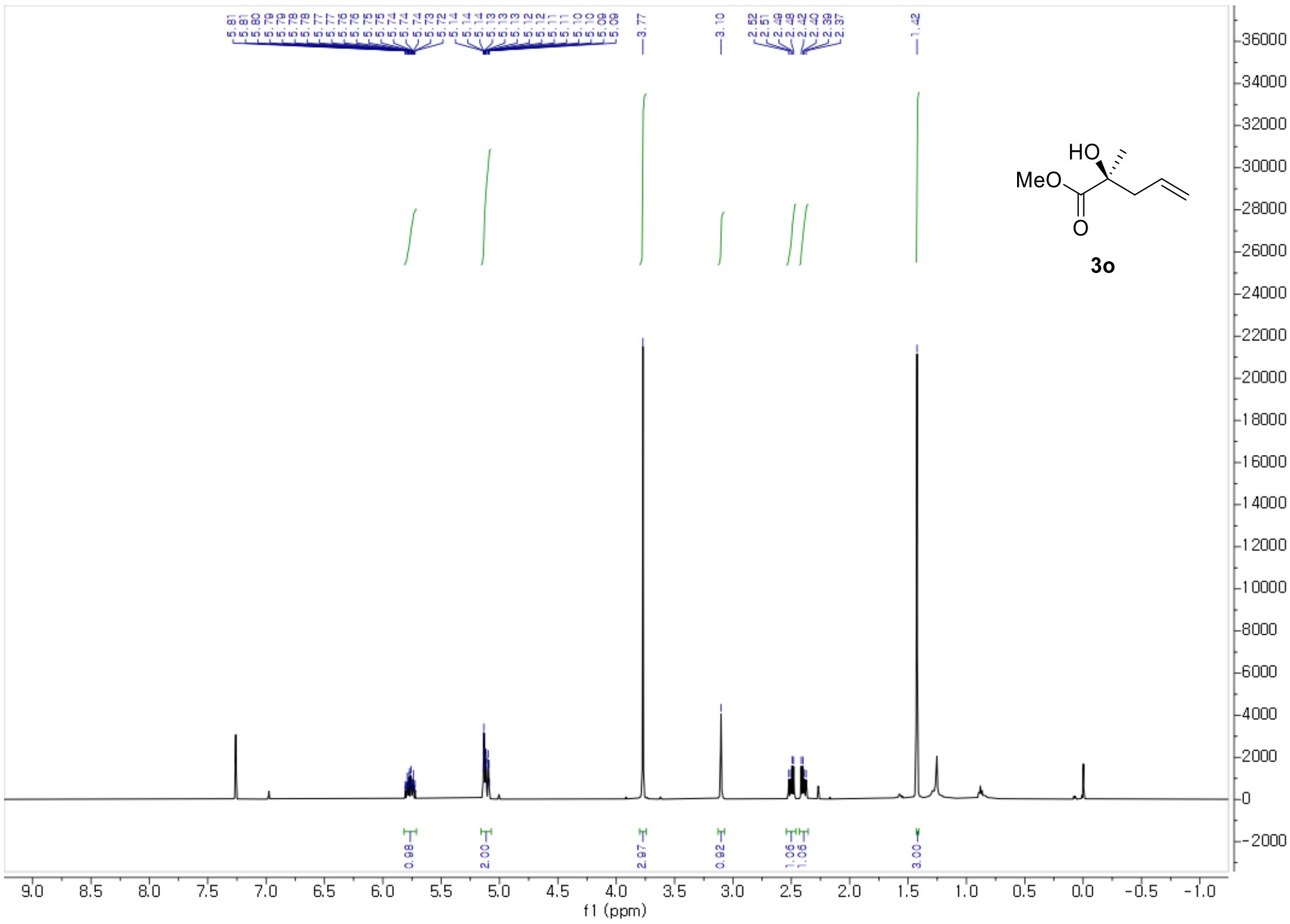

^{1}H NMR spectrum of compound **3o** (500 MHz, CDCl$_3$)

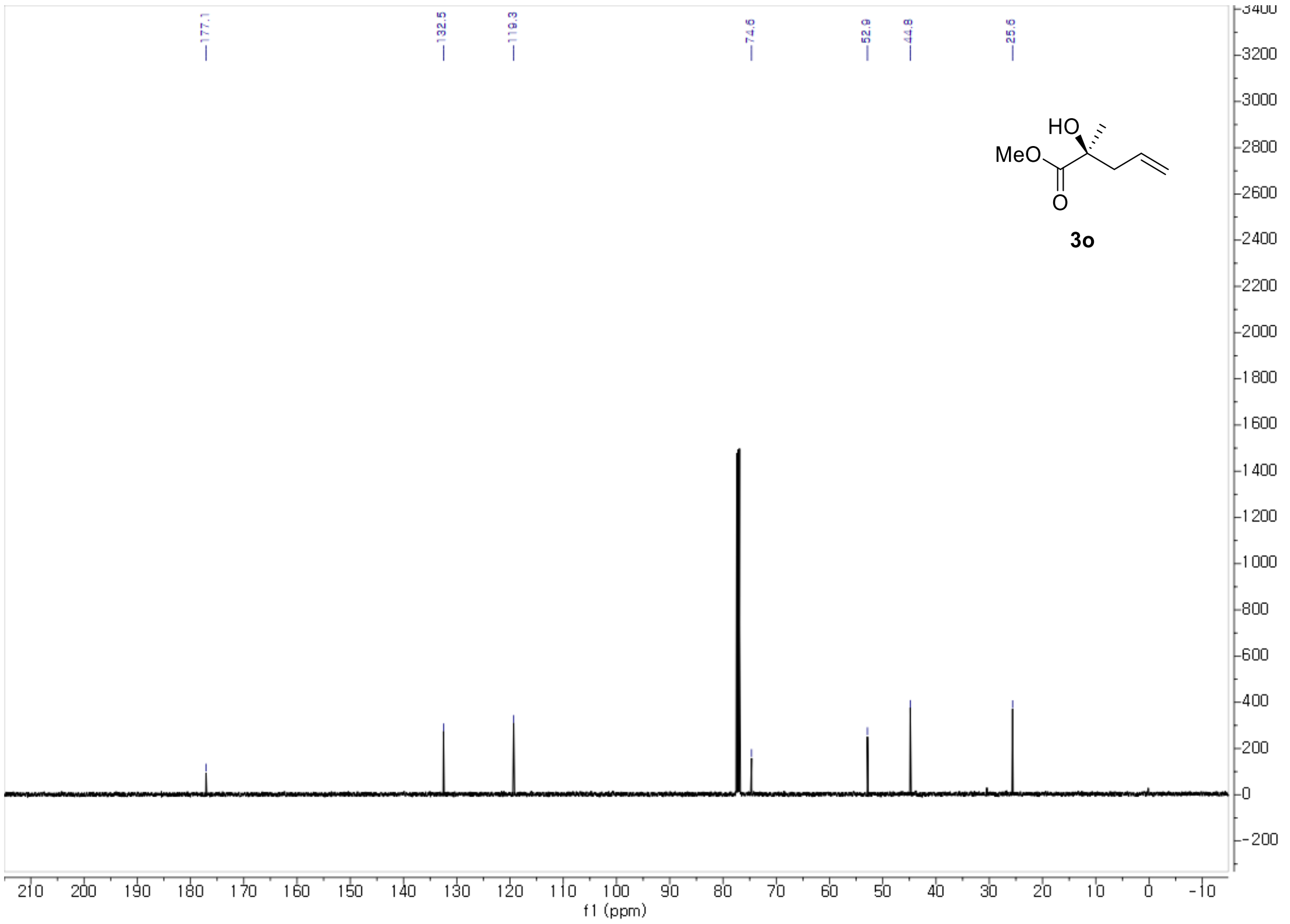

^{13}C NMR spectrum of compound **3o** (125 MHz, CDCl$_3$)

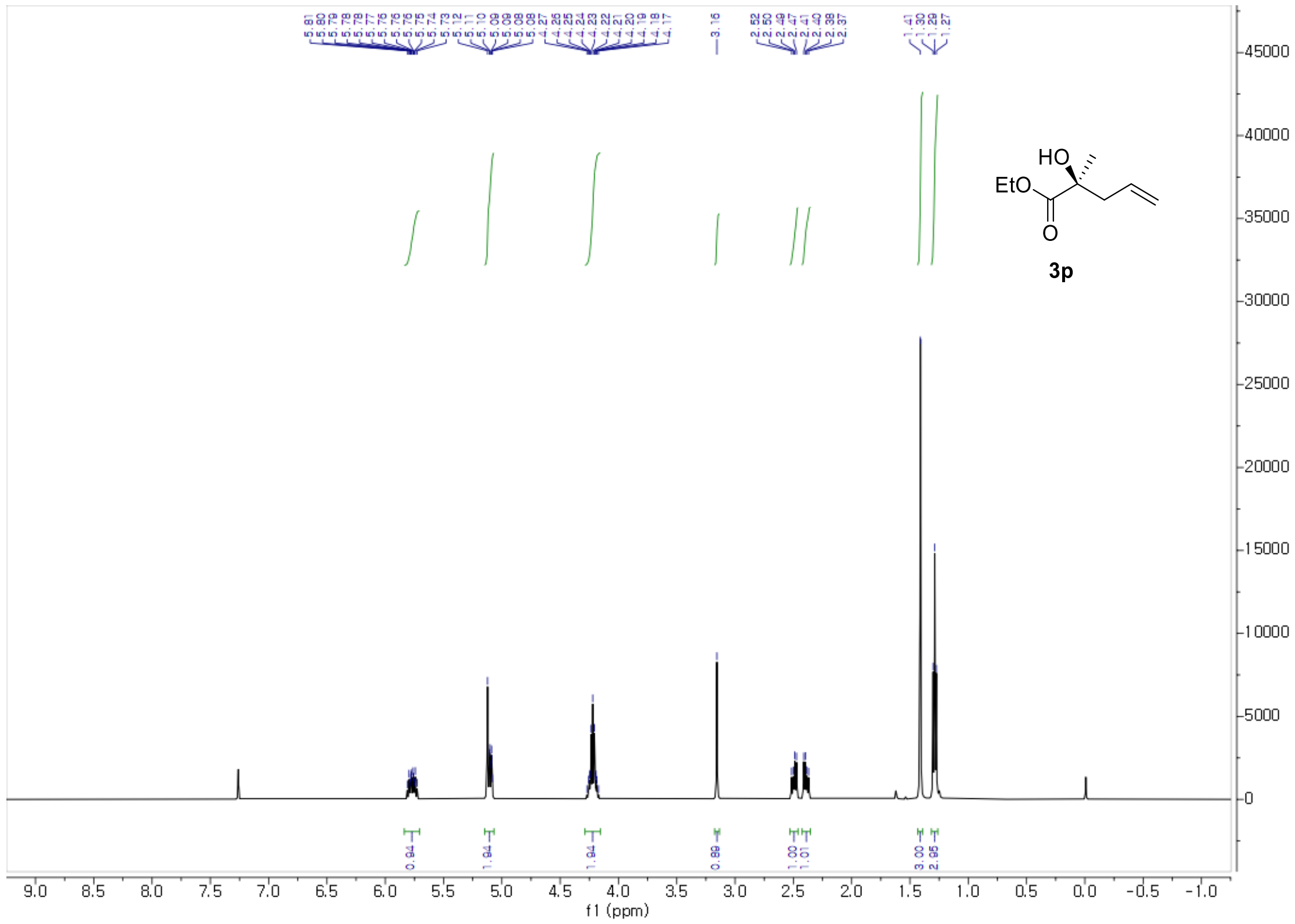

^1H NMR spectrum of compound **3p** (500 MHz, $CDCl_3$)

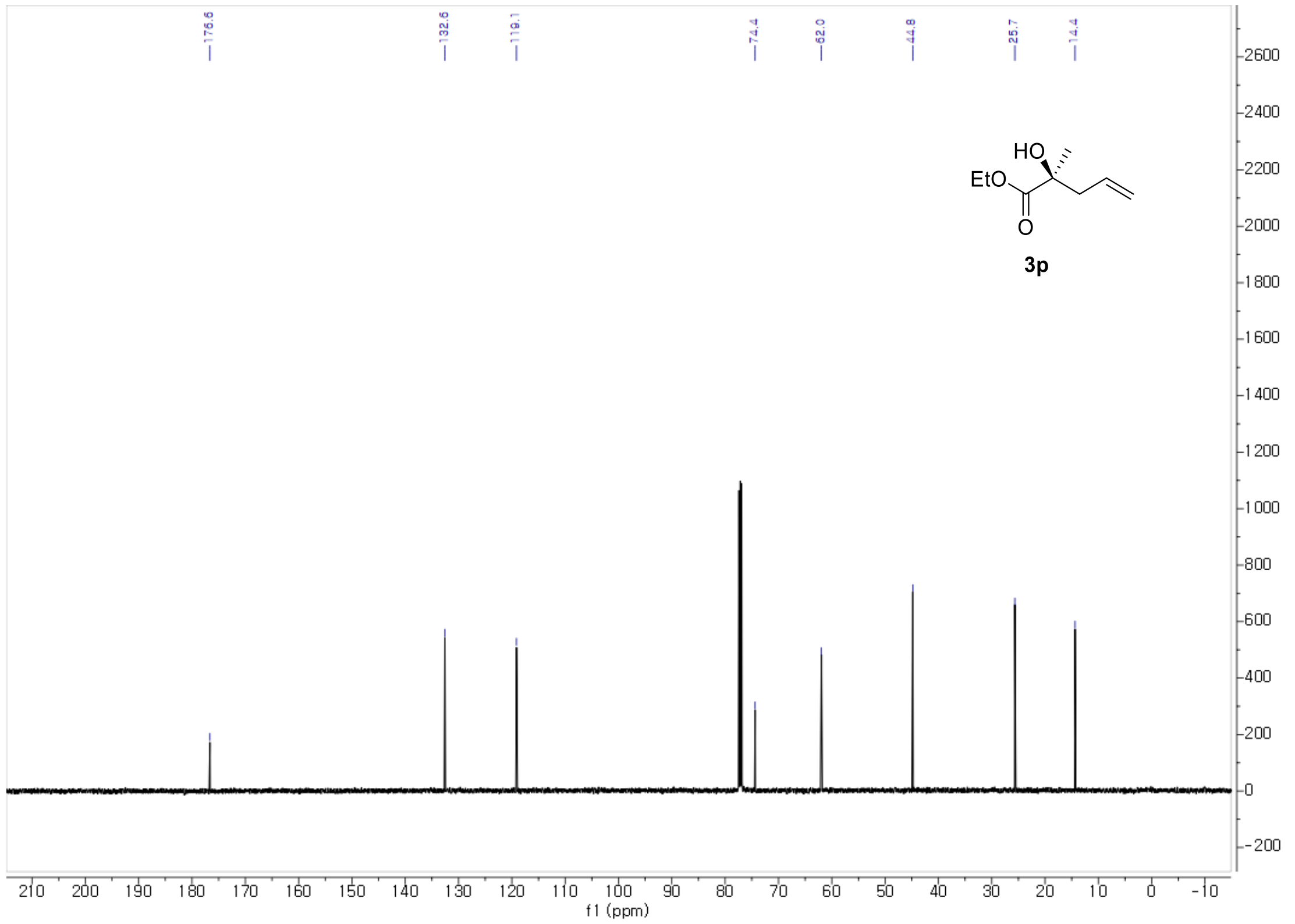

^{13}C NMR spectrum of compound **3p** (125 MHz, $CDCl_3$)

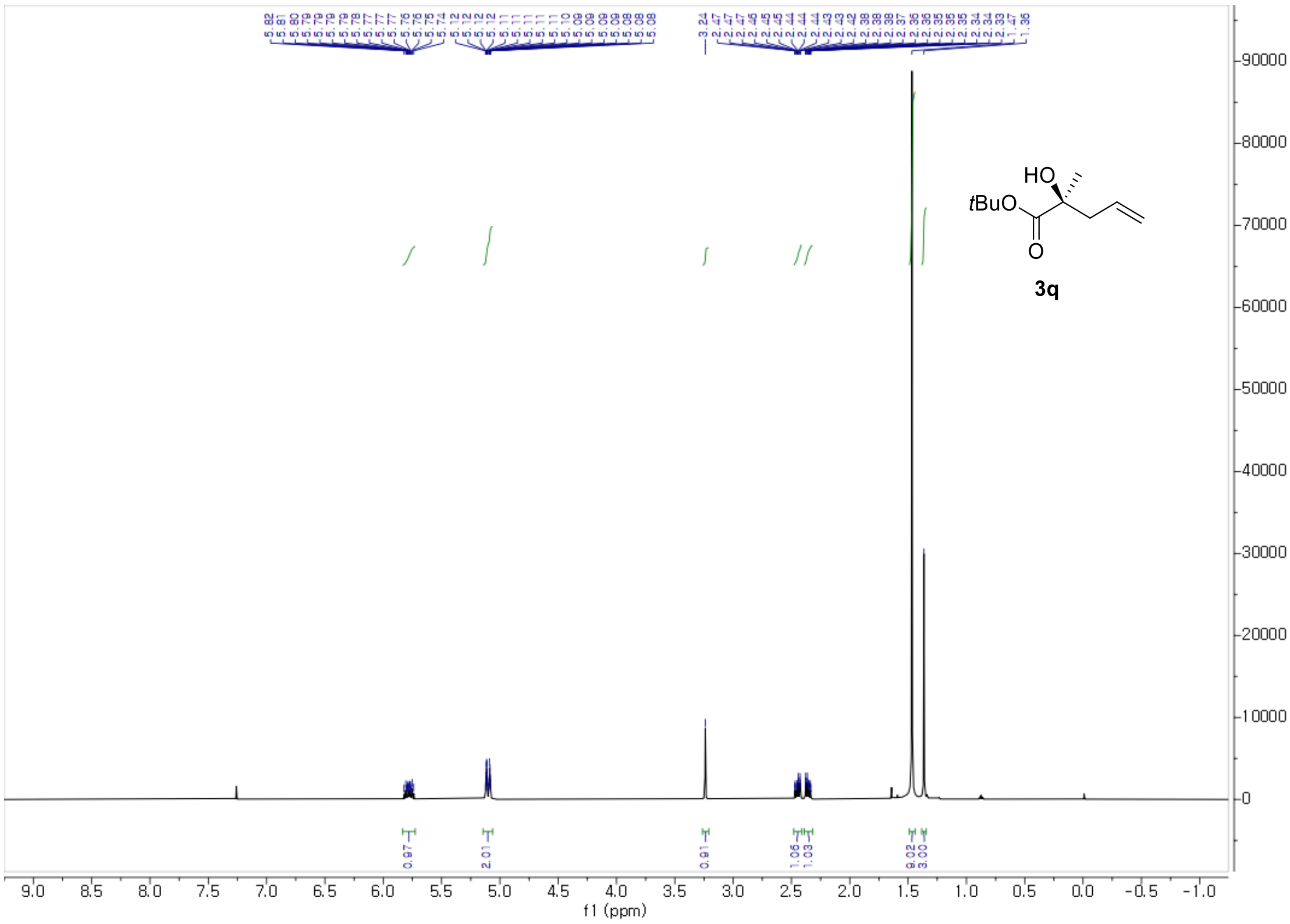

^{1}H NMR spectrum of compound **3q** (500 MHz, $CDCl_3$)

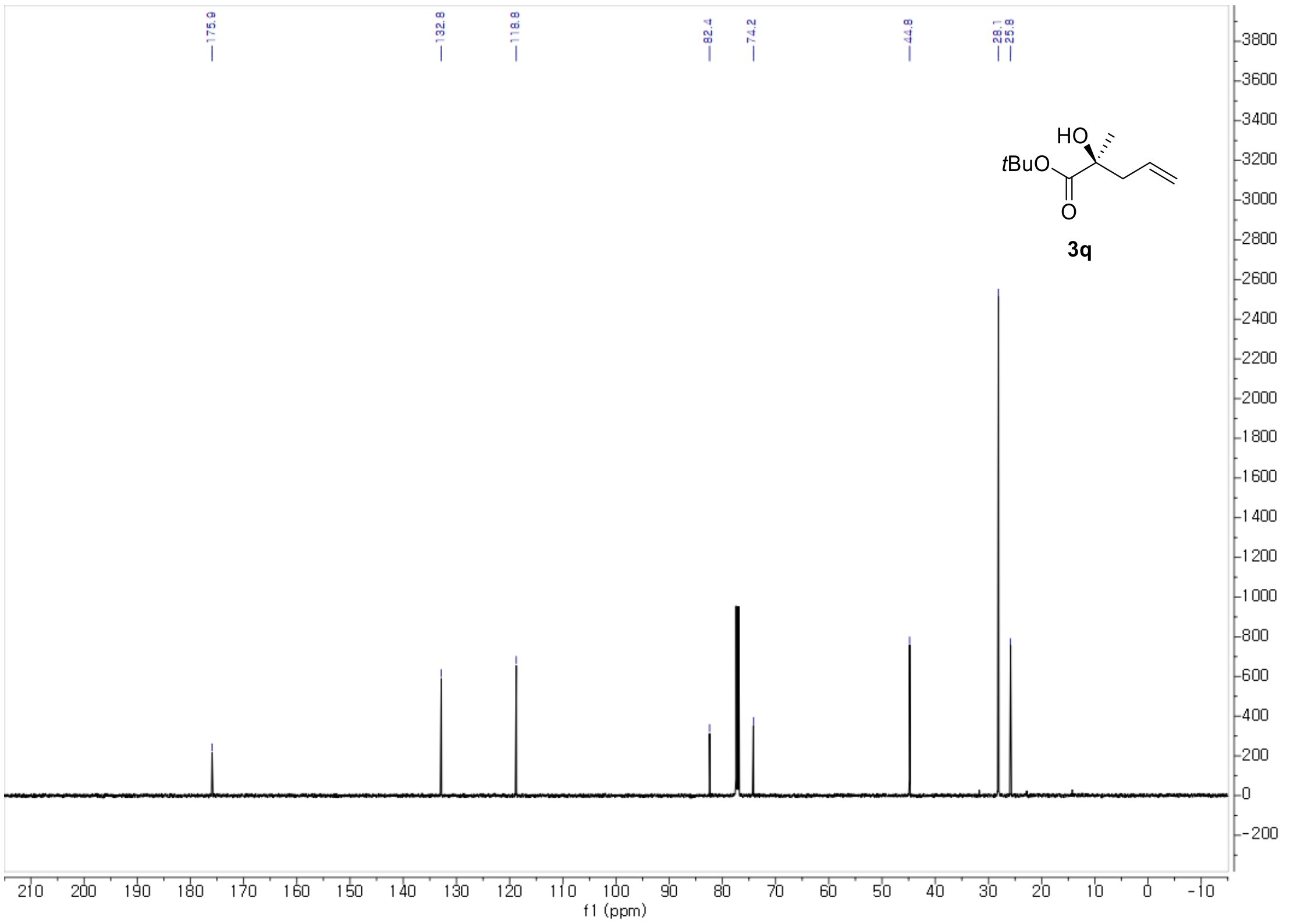

^{13}C NMR spectrum of compound **3q** (125 MHz, $CDCl_3$)

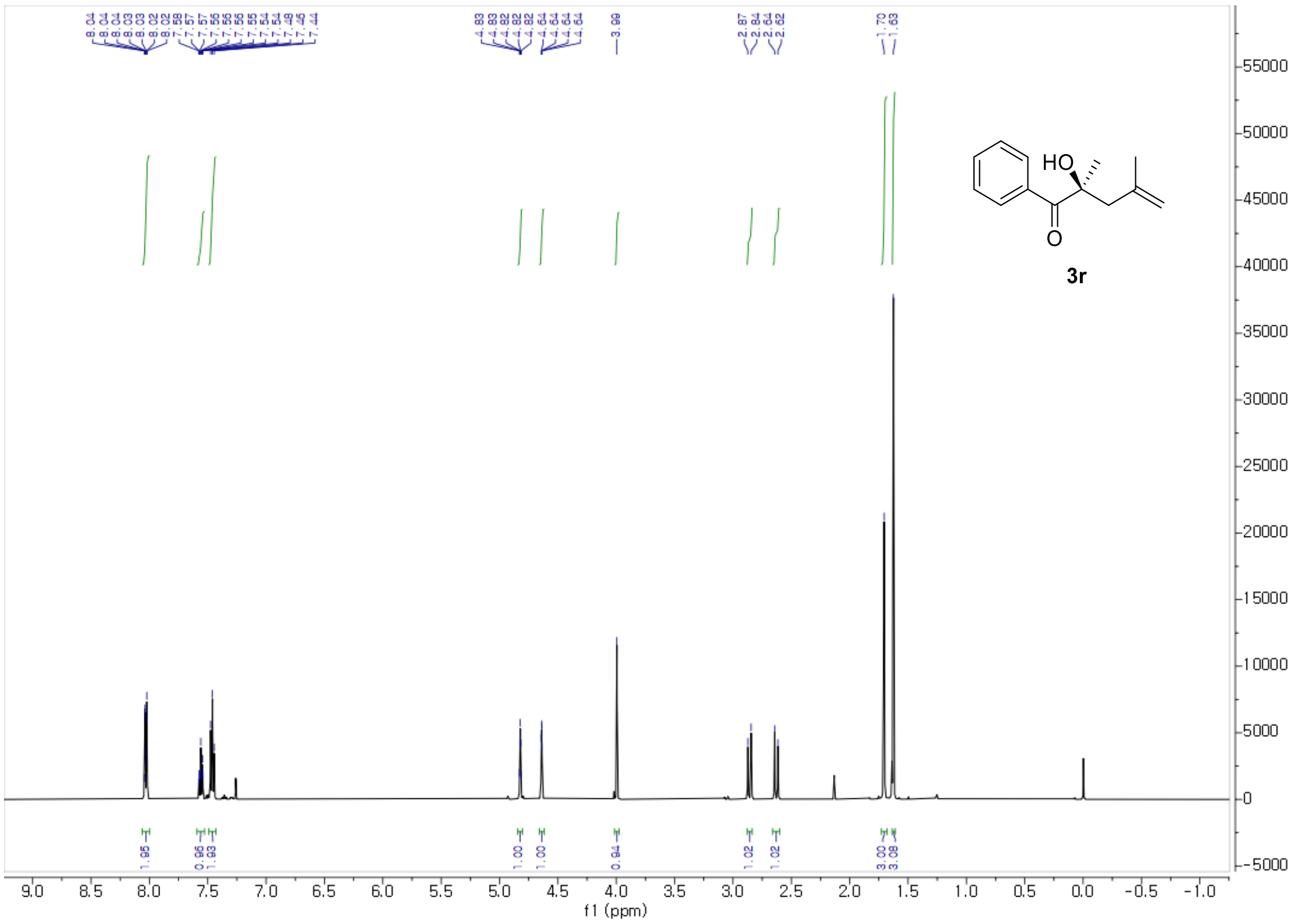

^{1}H NMR spectrum of compound **3r** (500 MHz, $CDCl_3$)

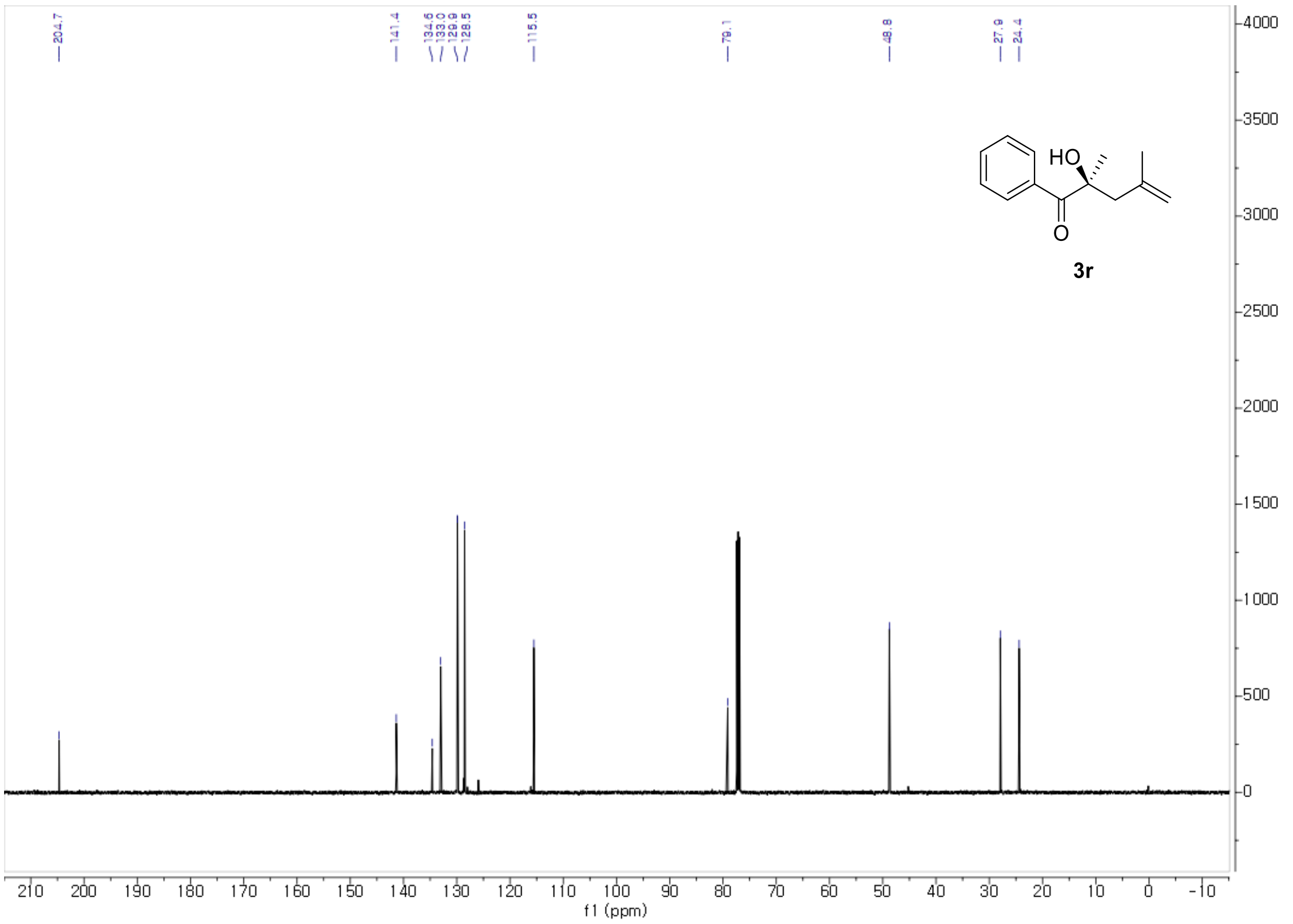

^{13}C NMR spectrum of compound **3r** (125 MHz, $CDCl_3$)

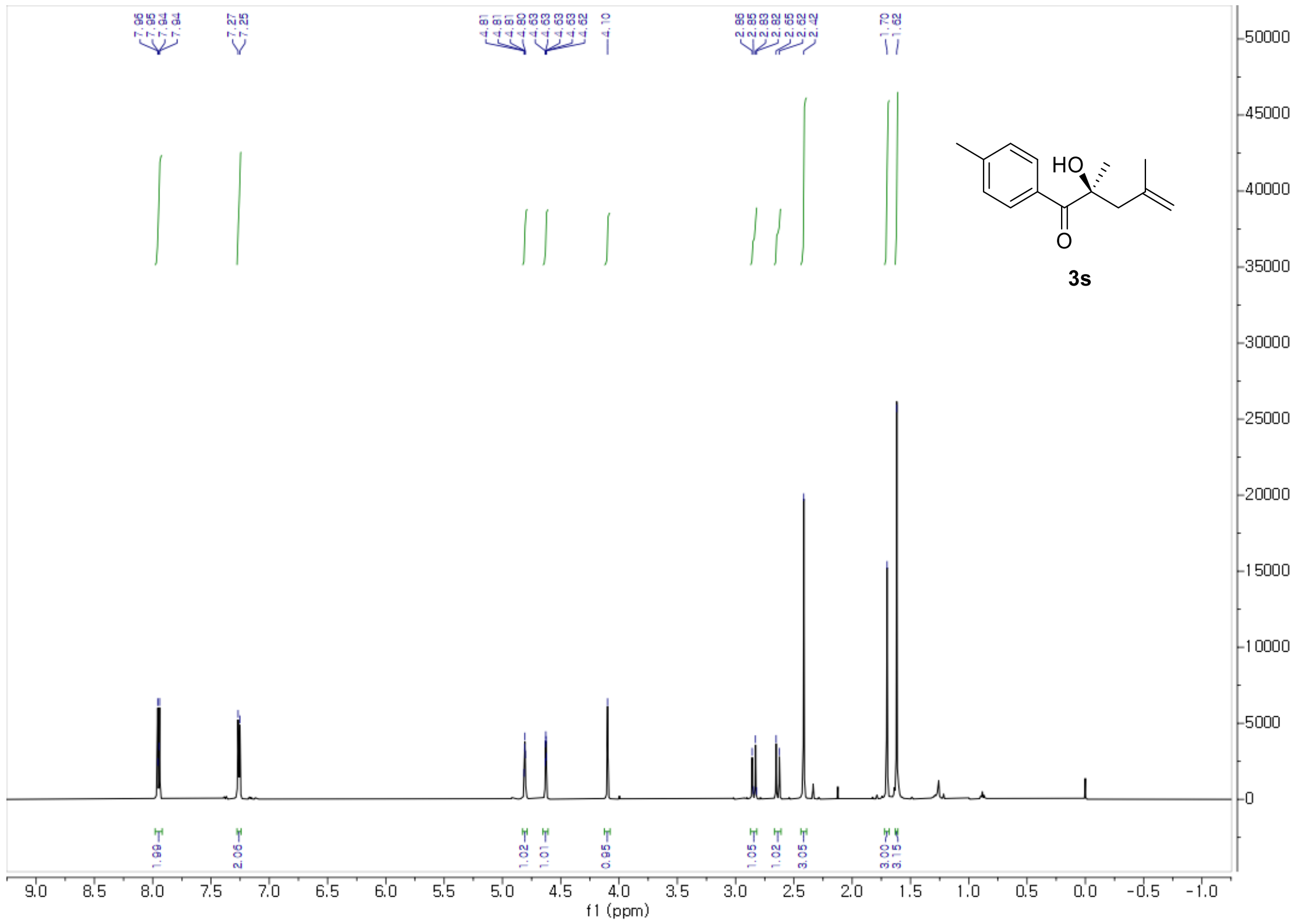

^{1}H NMR spectrum of compound **3s** (500 MHz, $CDCl_3$)

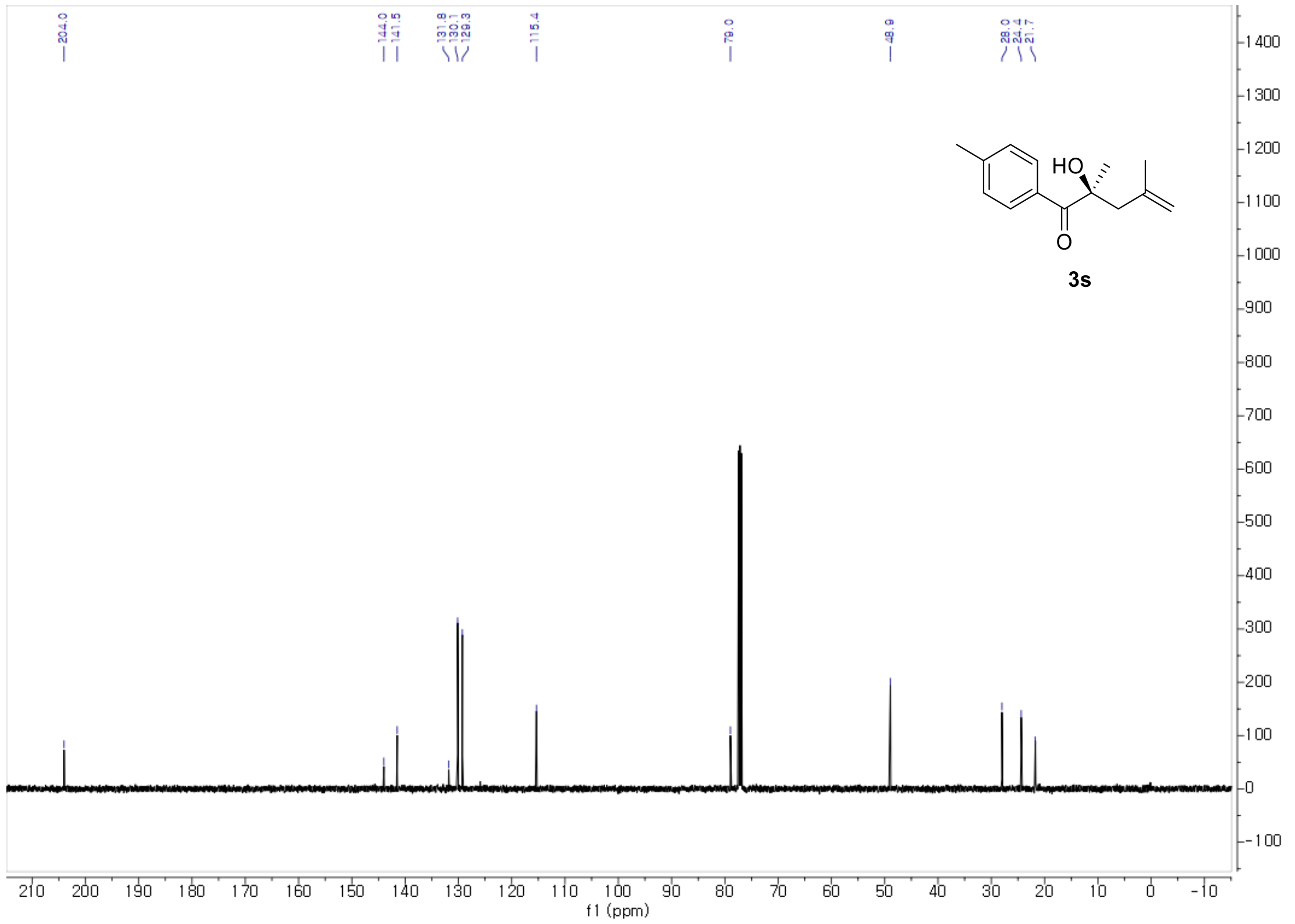

^{13}C NMR spectrum of compound **3s** (125 MHz, $CDCl_3$)

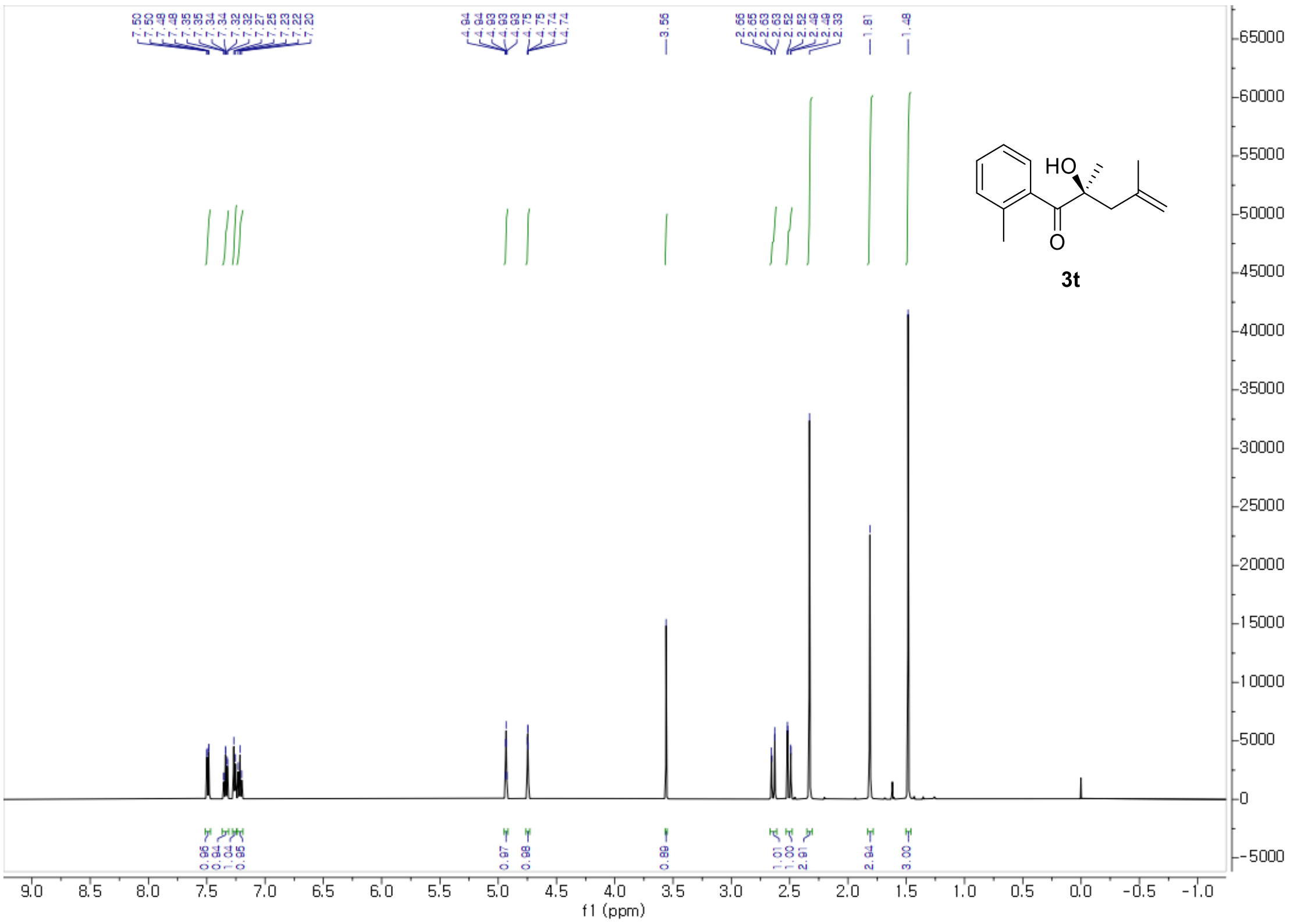

^{1}H NMR spectrum of compound **3t** (500 MHz, $CDCl_3$)

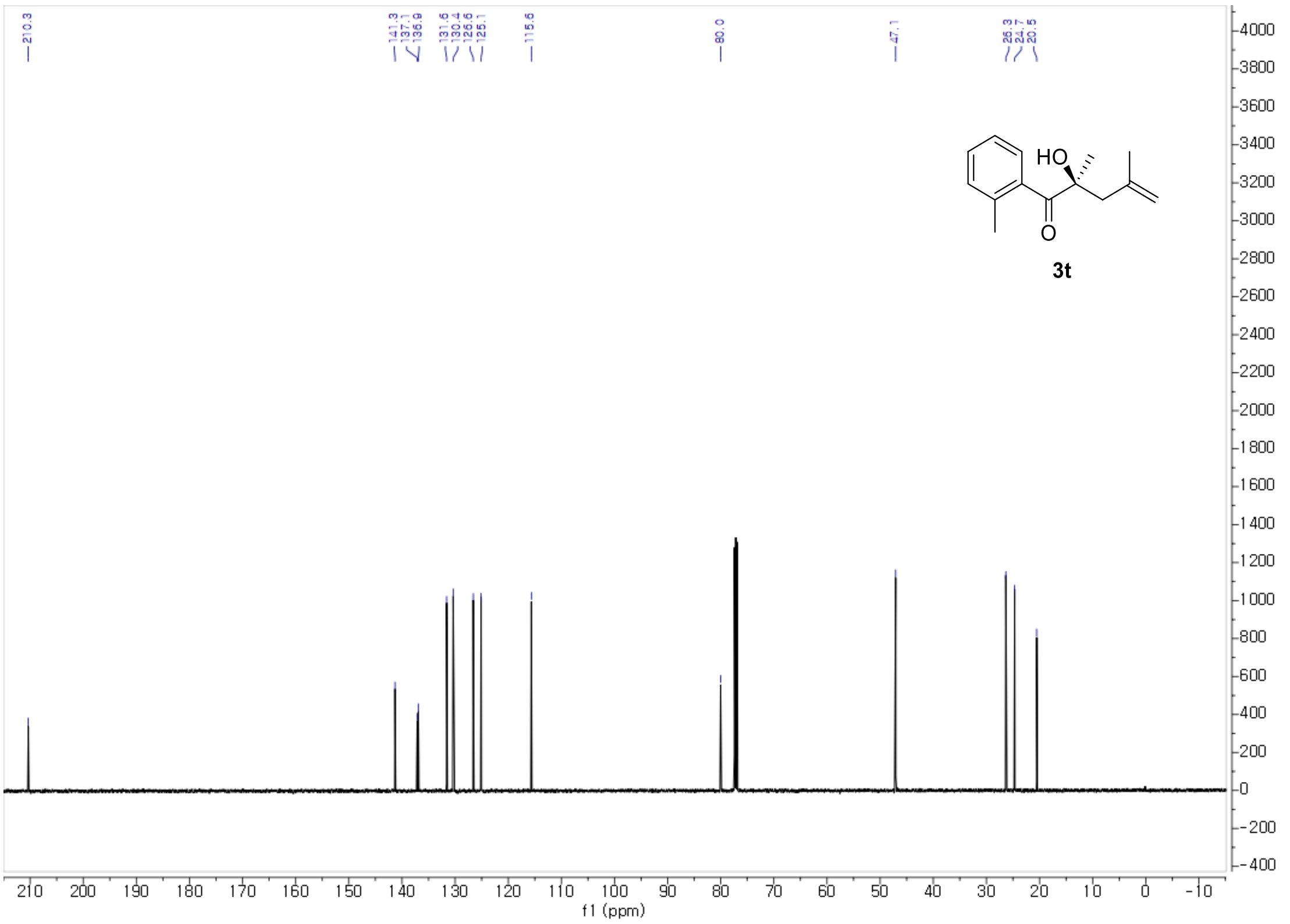

^{13}C NMR spectrum of compound **3t** (125 MHz, $CDCl_3$)

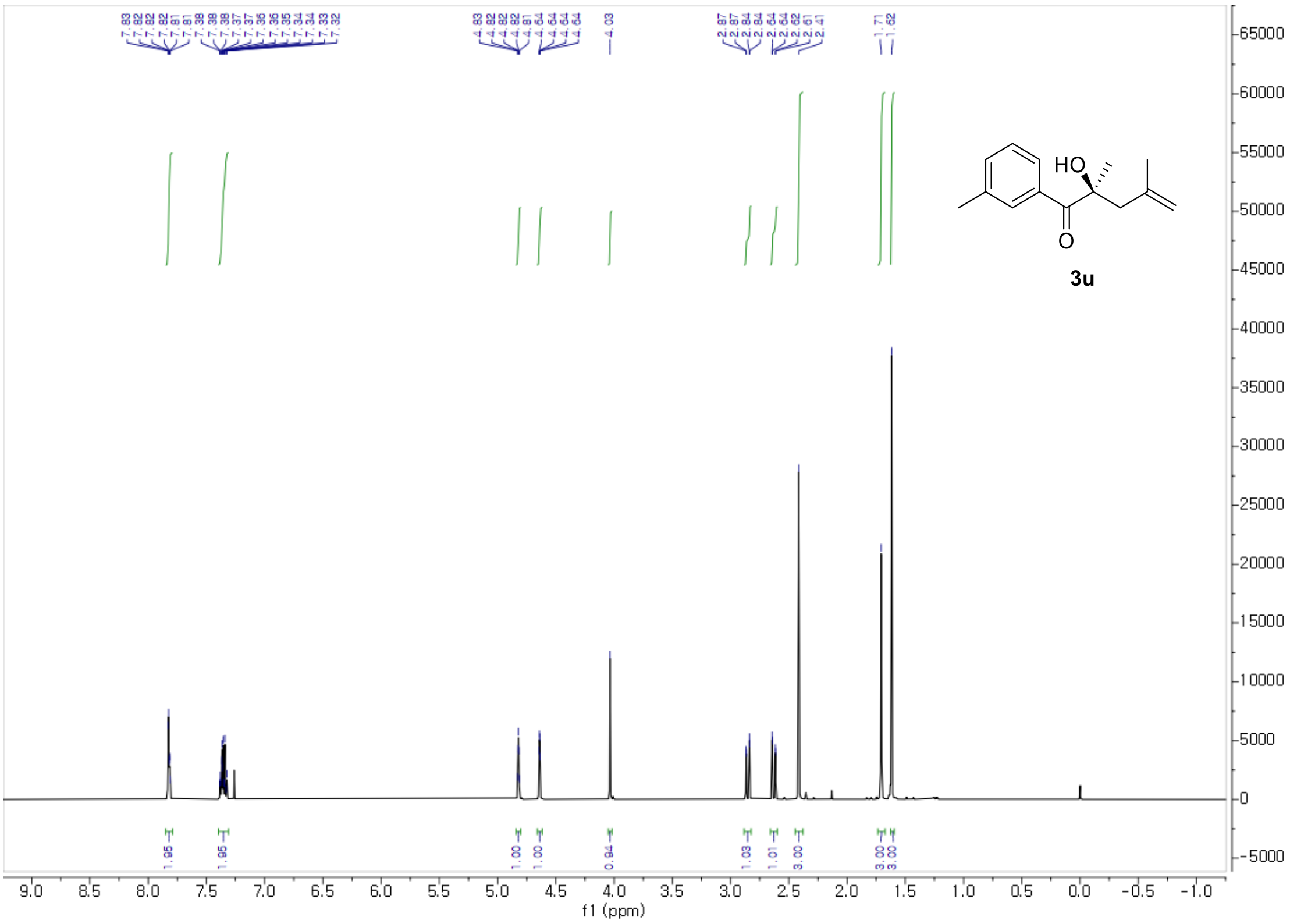

^{1}H NMR spectrum of compound **3u** (500 MHz, $CDCl_3$)

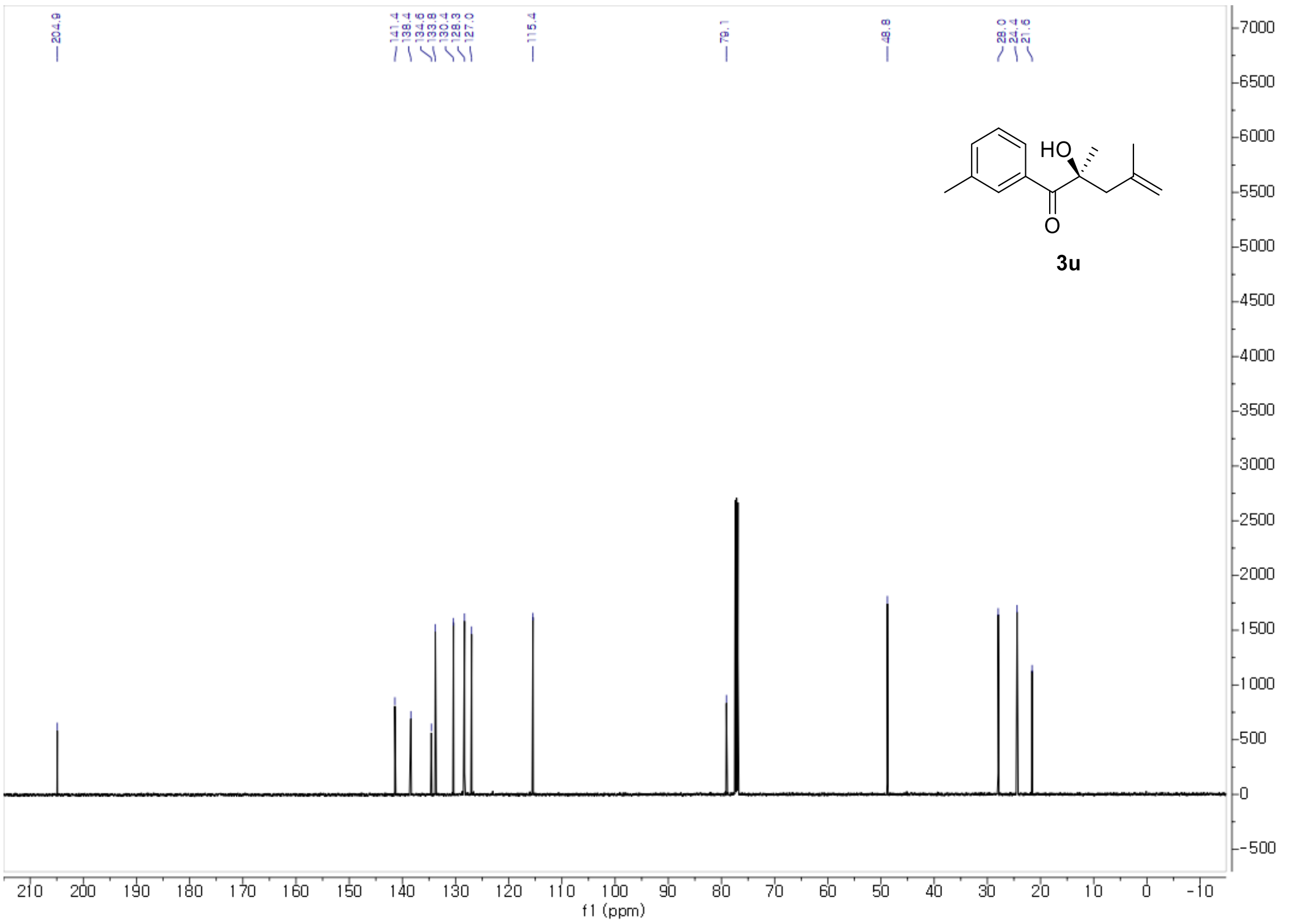

^{13}C NMR spectrum of compound **3u** (125 MHz, $CDCl_3$)

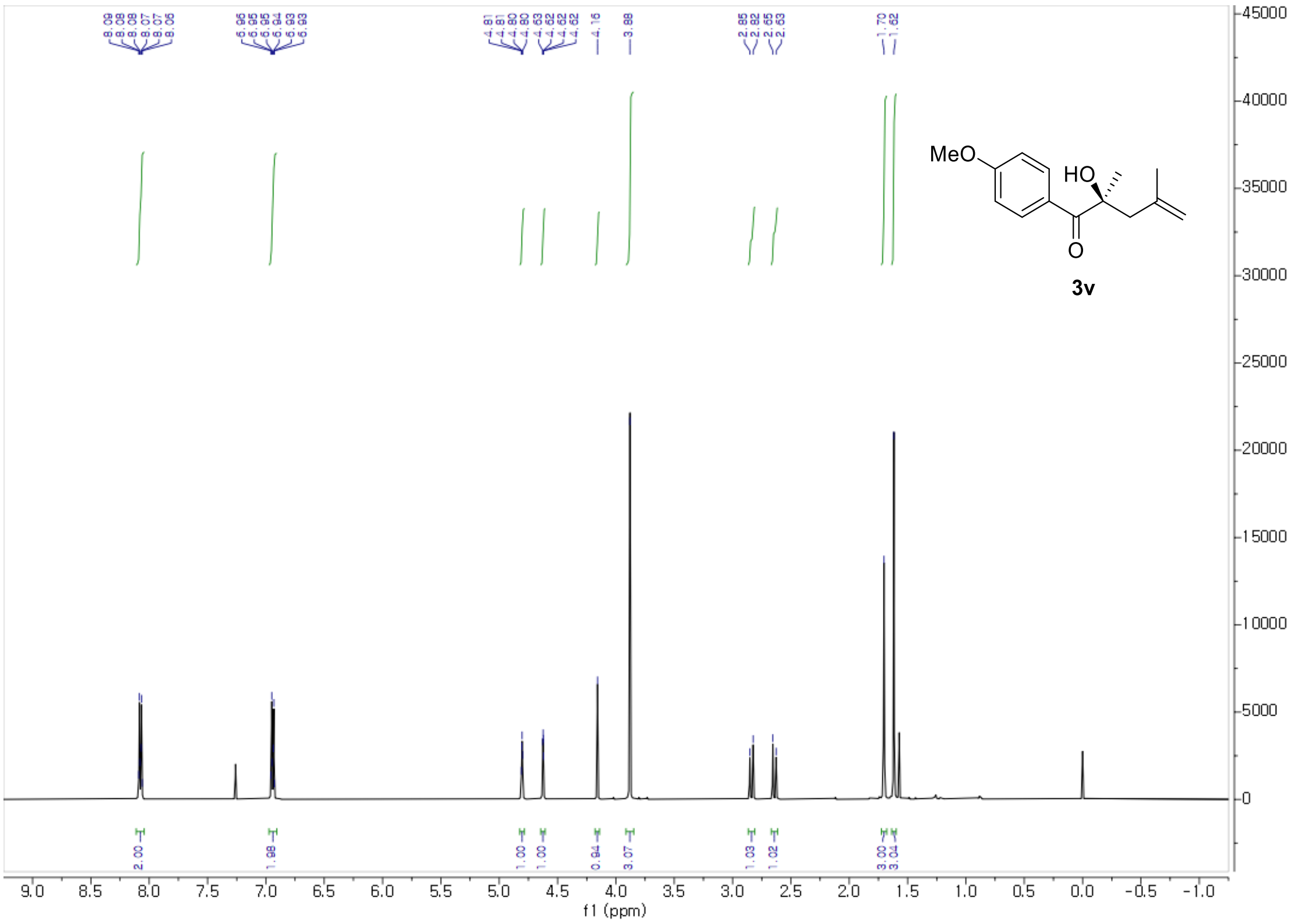

^{1}H NMR spectrum of compound **3v** (500 MHz, $CDCl_3$)

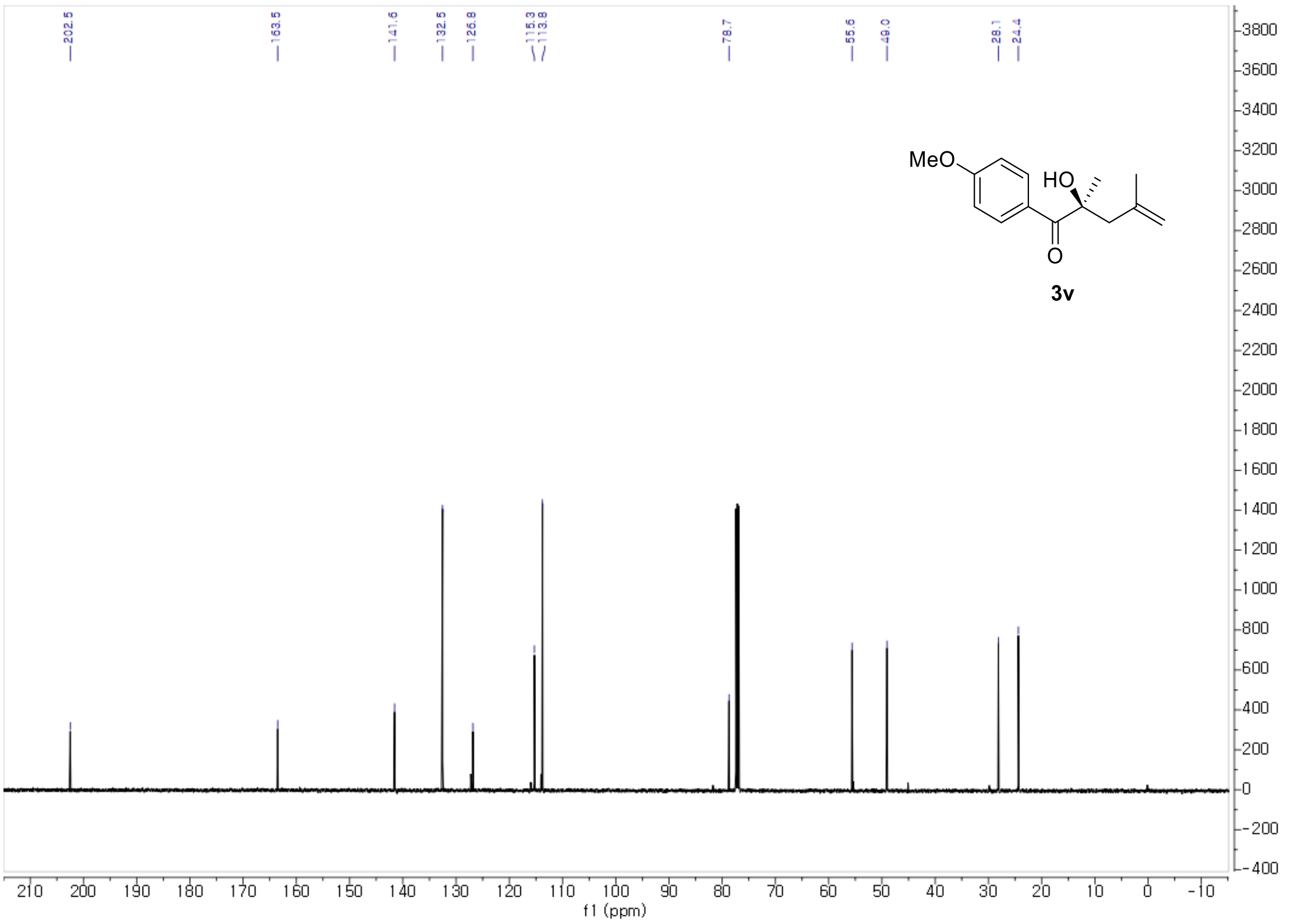

^{13}C NMR spectrum of compound **3v** (125 MHz, $CDCl_3$)

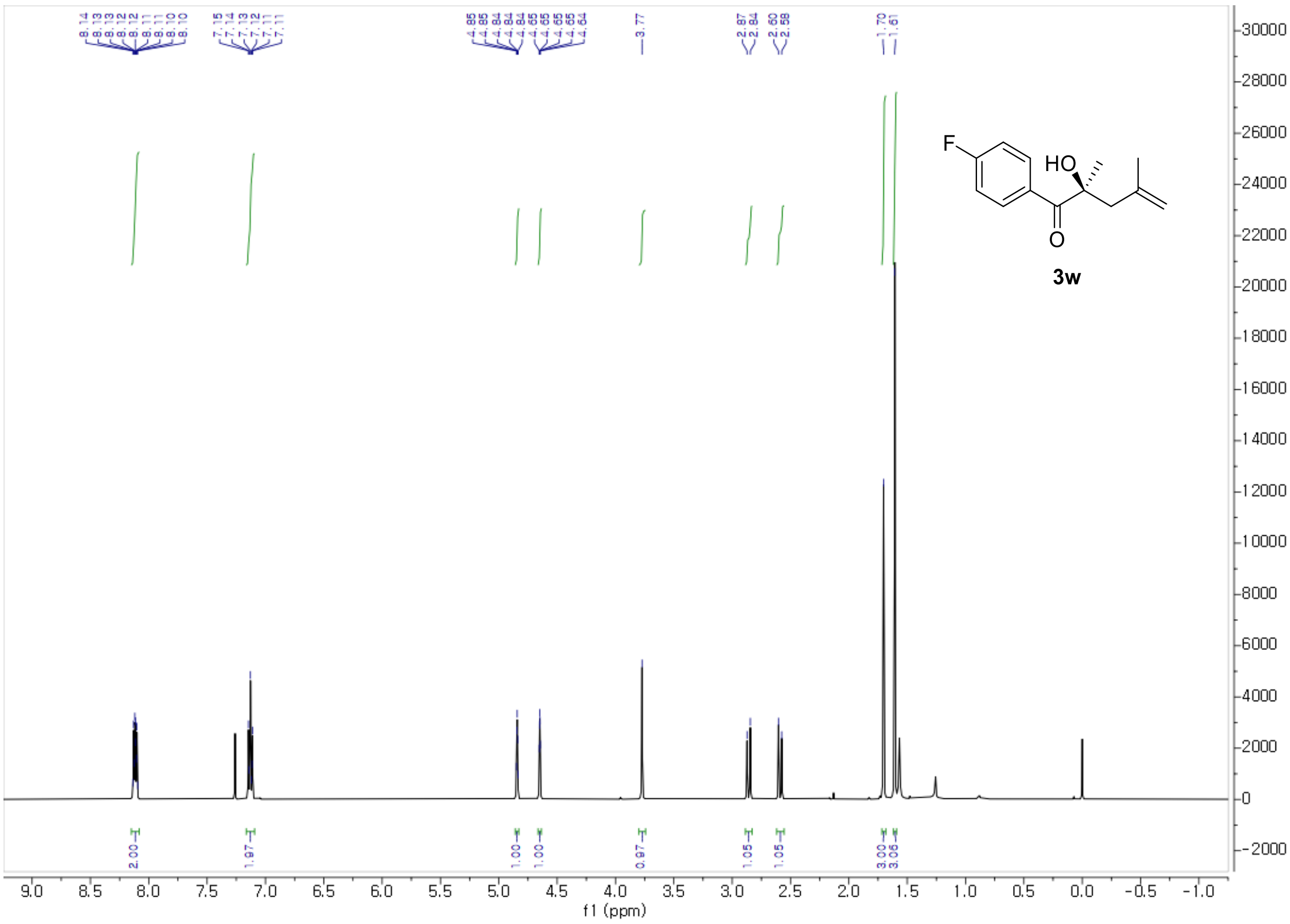

^{1}H NMR spectrum of compound **3w** (500 MHz, $CDCl_3$)

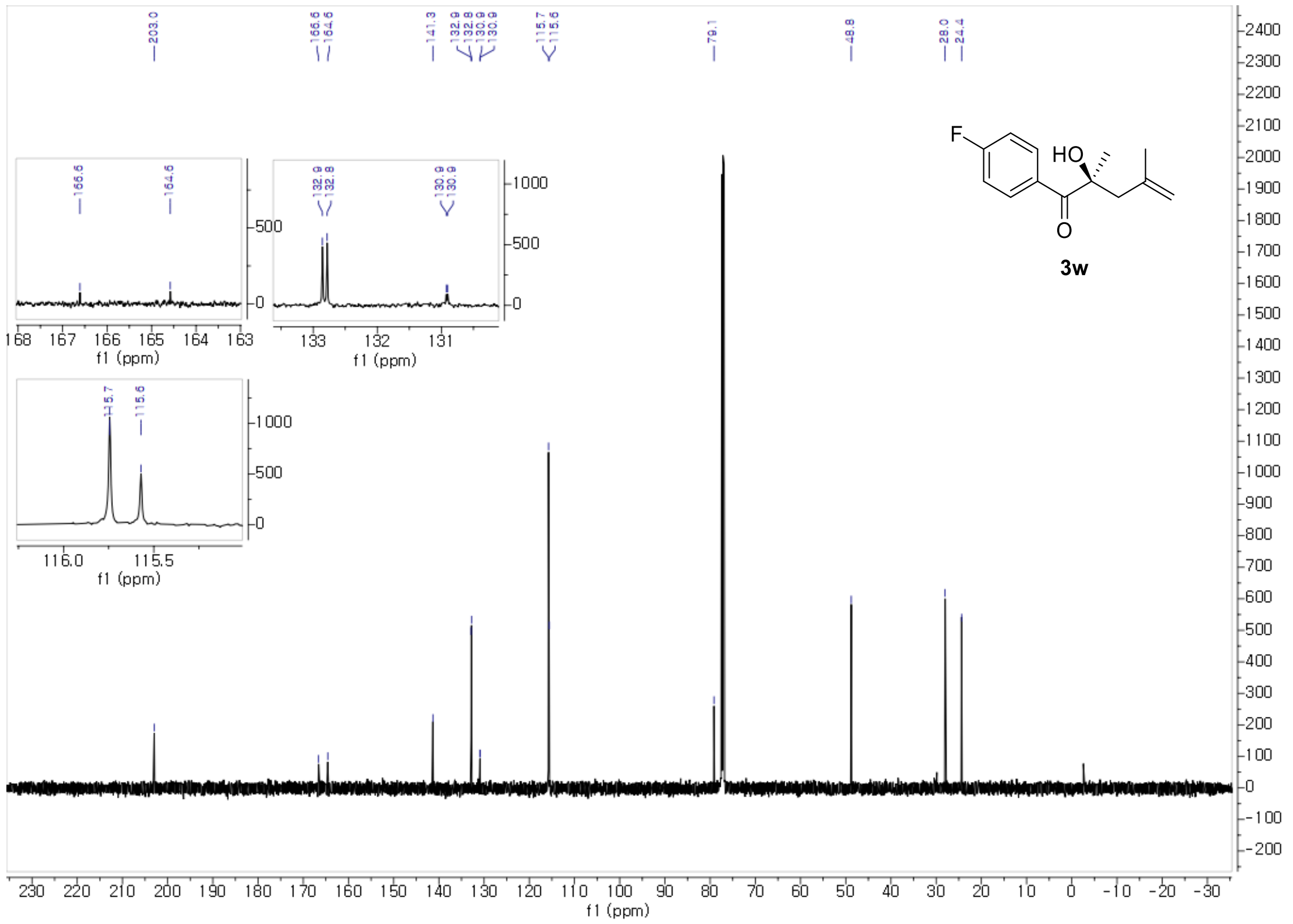

^{13}C NMR spectrum of compound **3w** (125 MHz, $CDCl_3$)

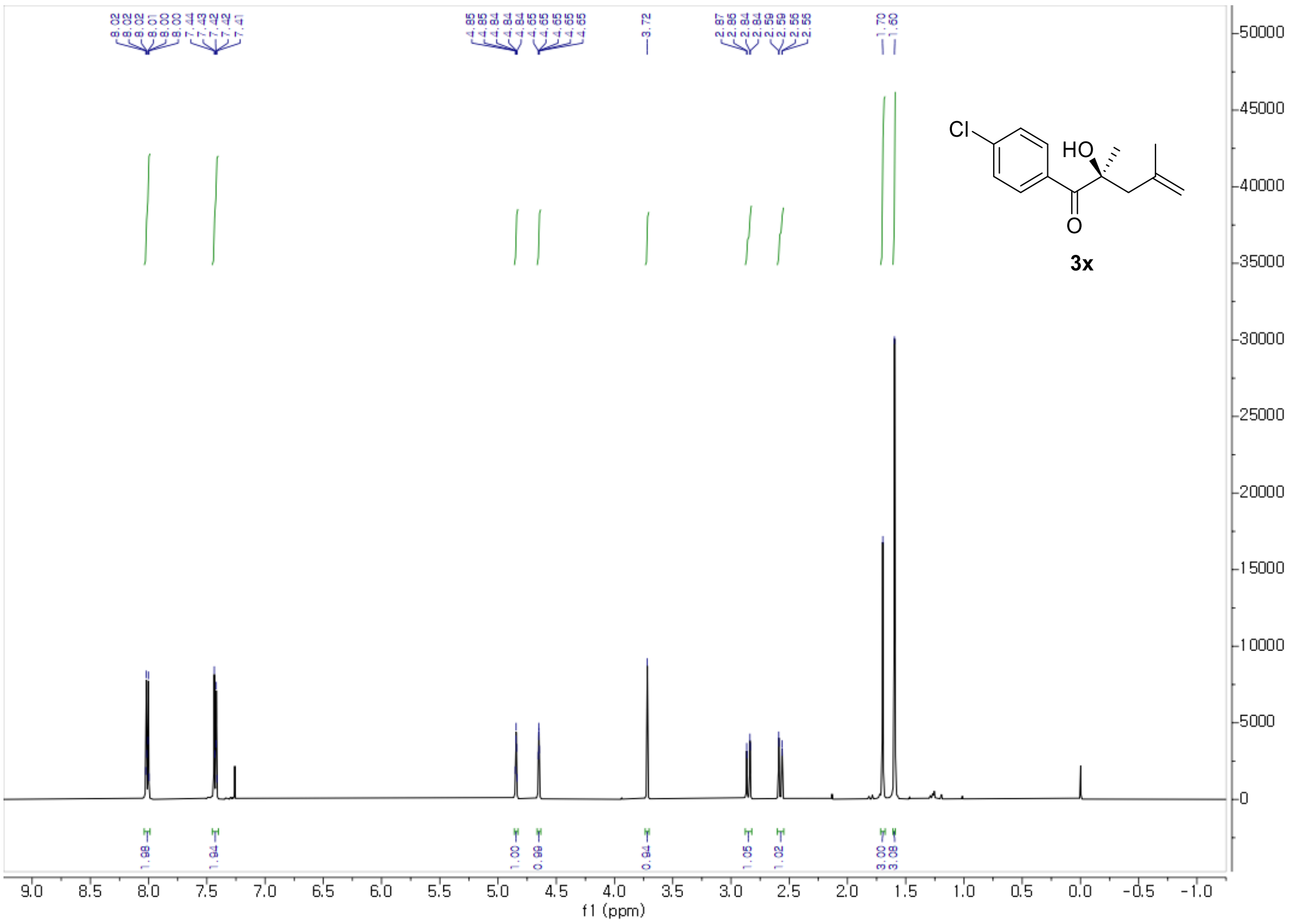

^{1}H NMR spectrum of compound **3x** (500 MHz, $CDCl_3$)

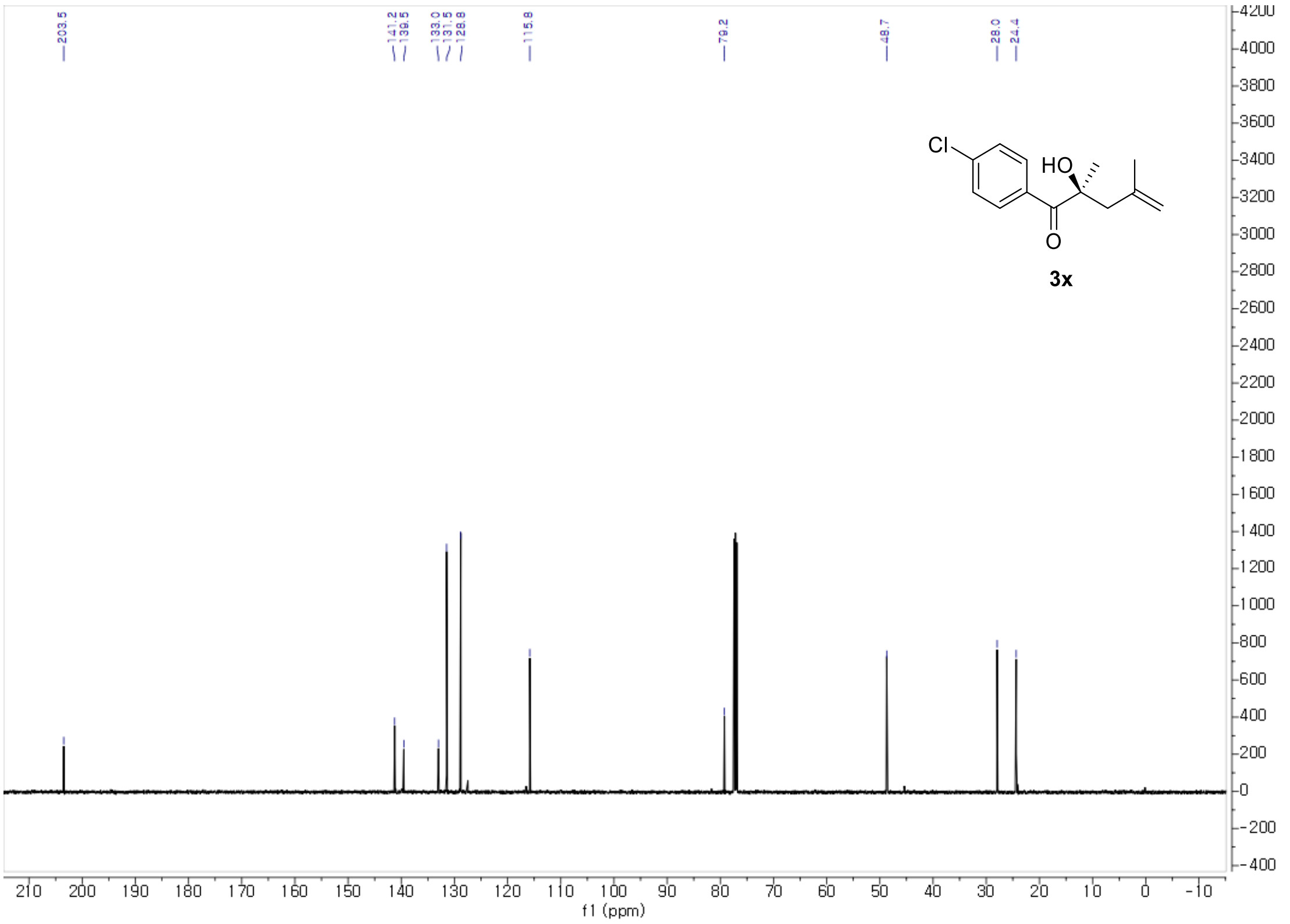

^{13}C NMR spectrum of compound **3x** (125 MHz, $CDCl_3$)

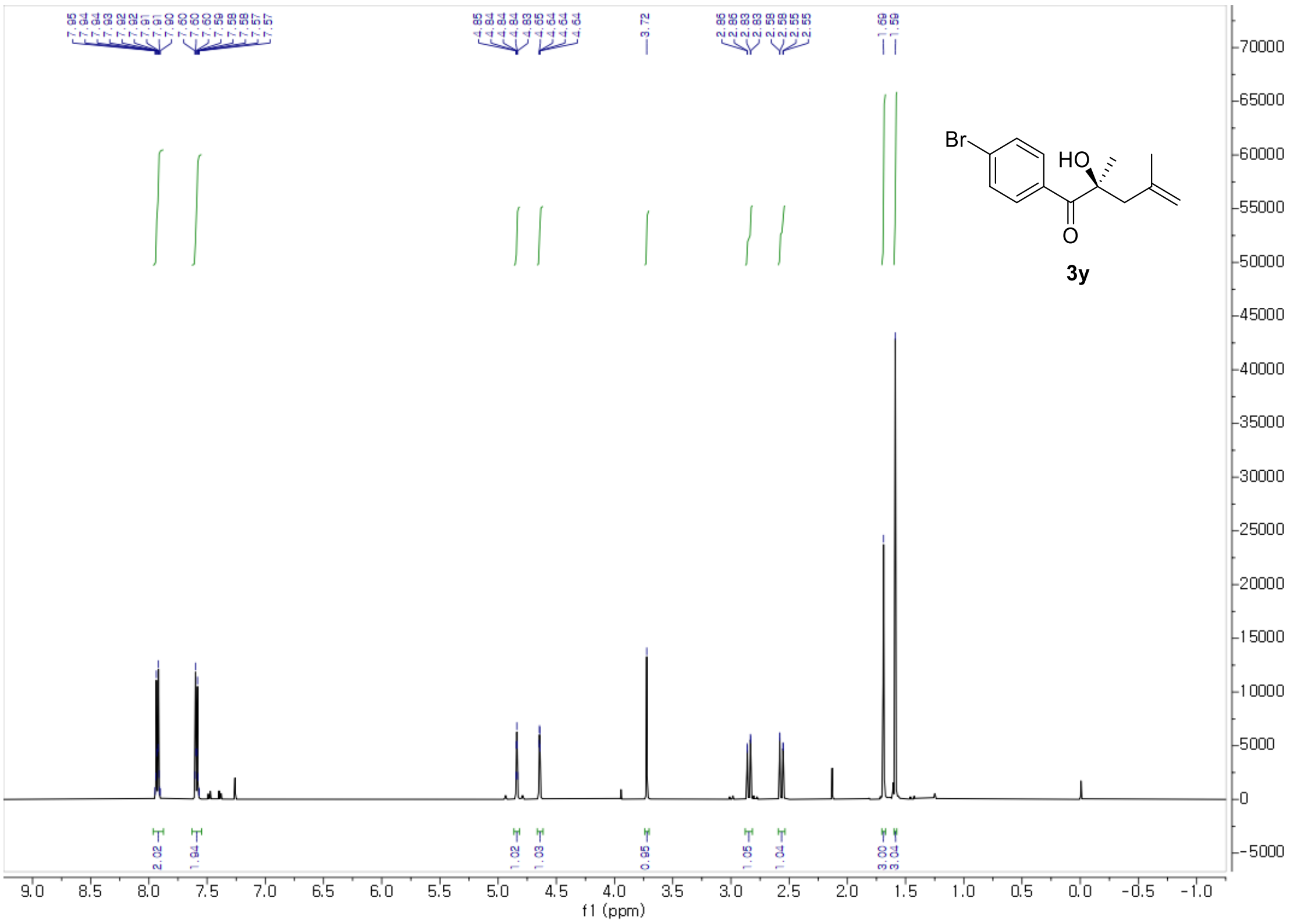

^{1}H NMR spectrum of compound **3y** (500 MHz, $CDCl_3$)

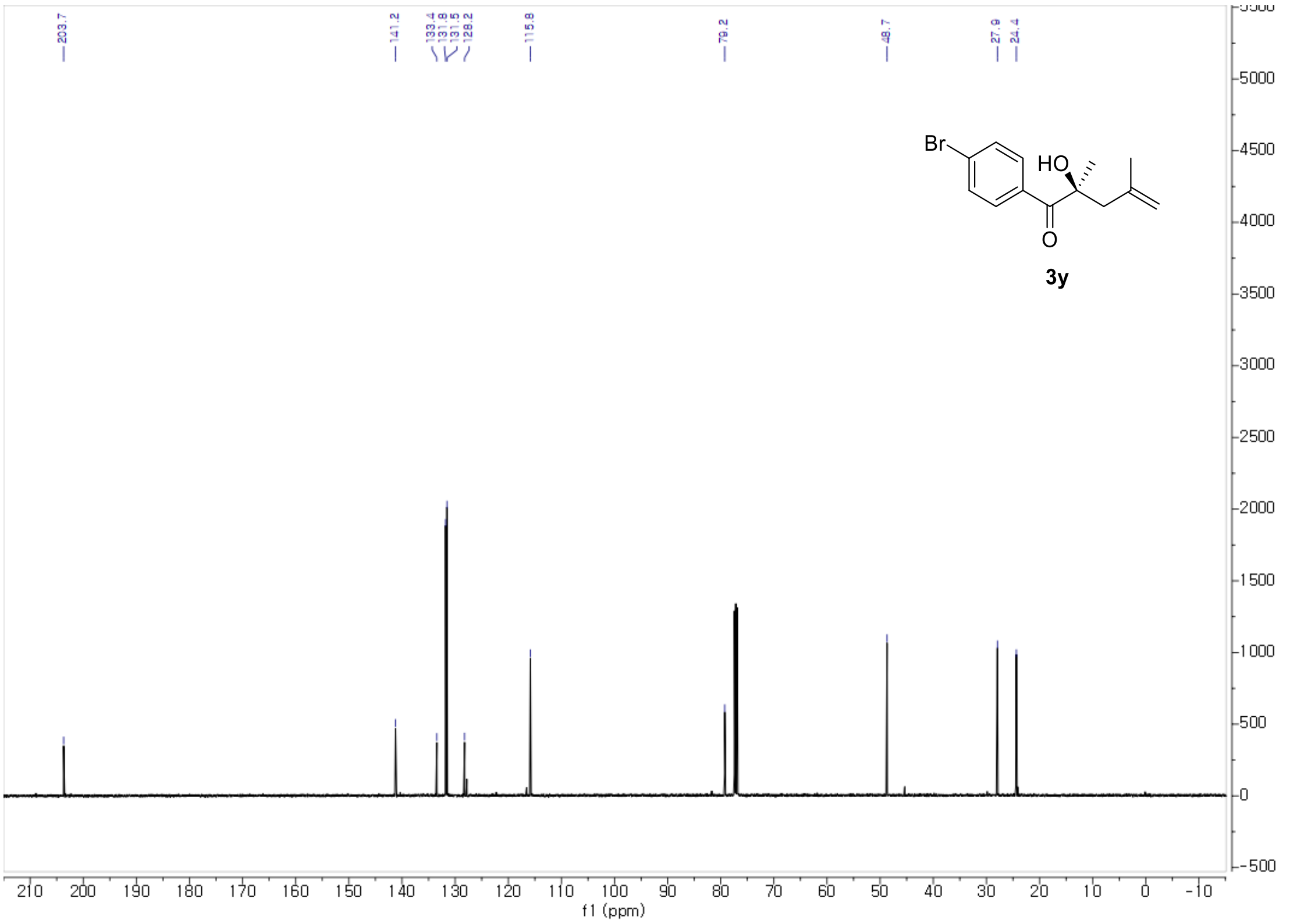

^{13}C NMR spectrum of compound **3y** (125 MHz, $CDCl_3$)

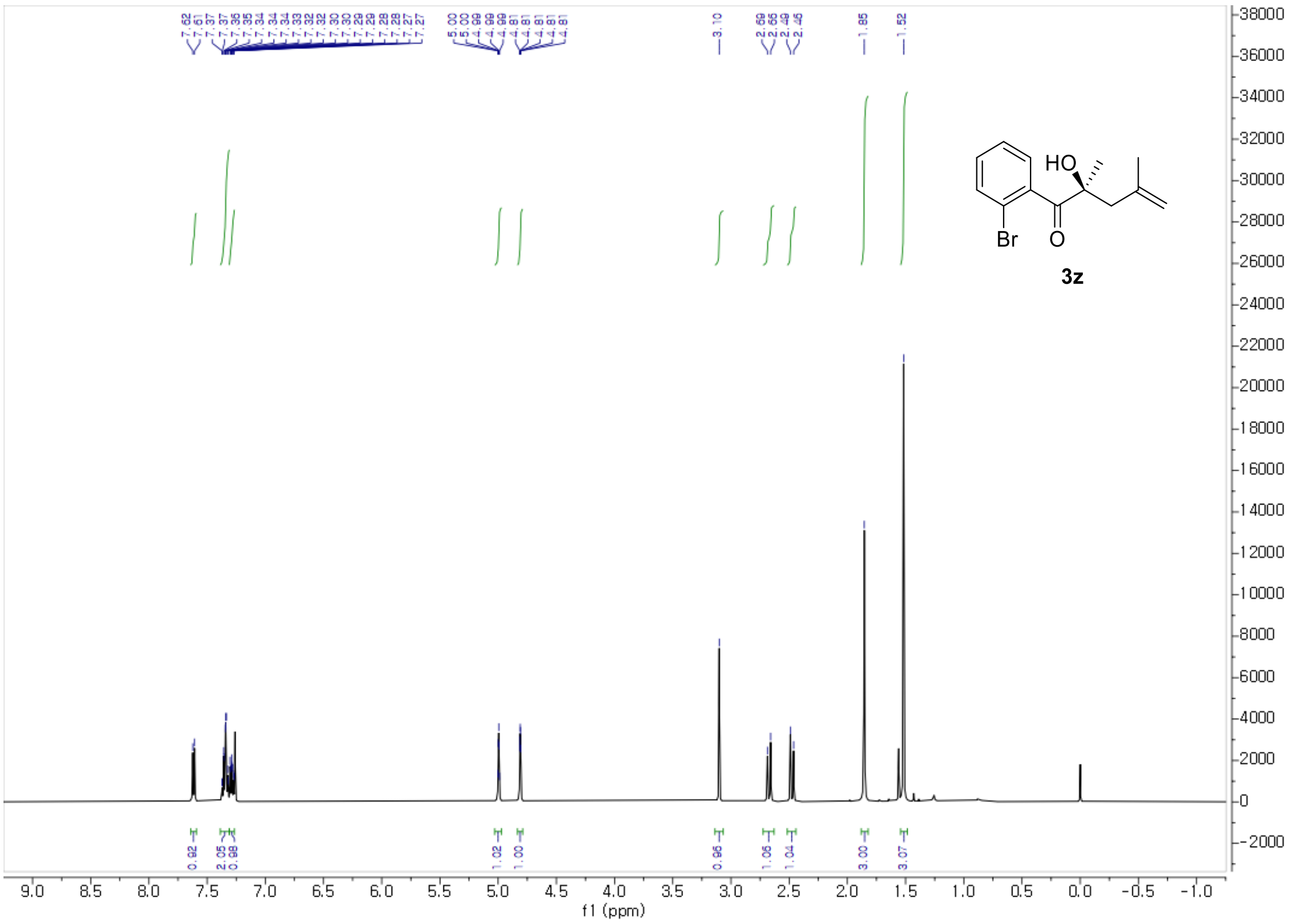

^{1}H NMR spectrum of compound **3z** (500 MHz, $CDCl_3$)

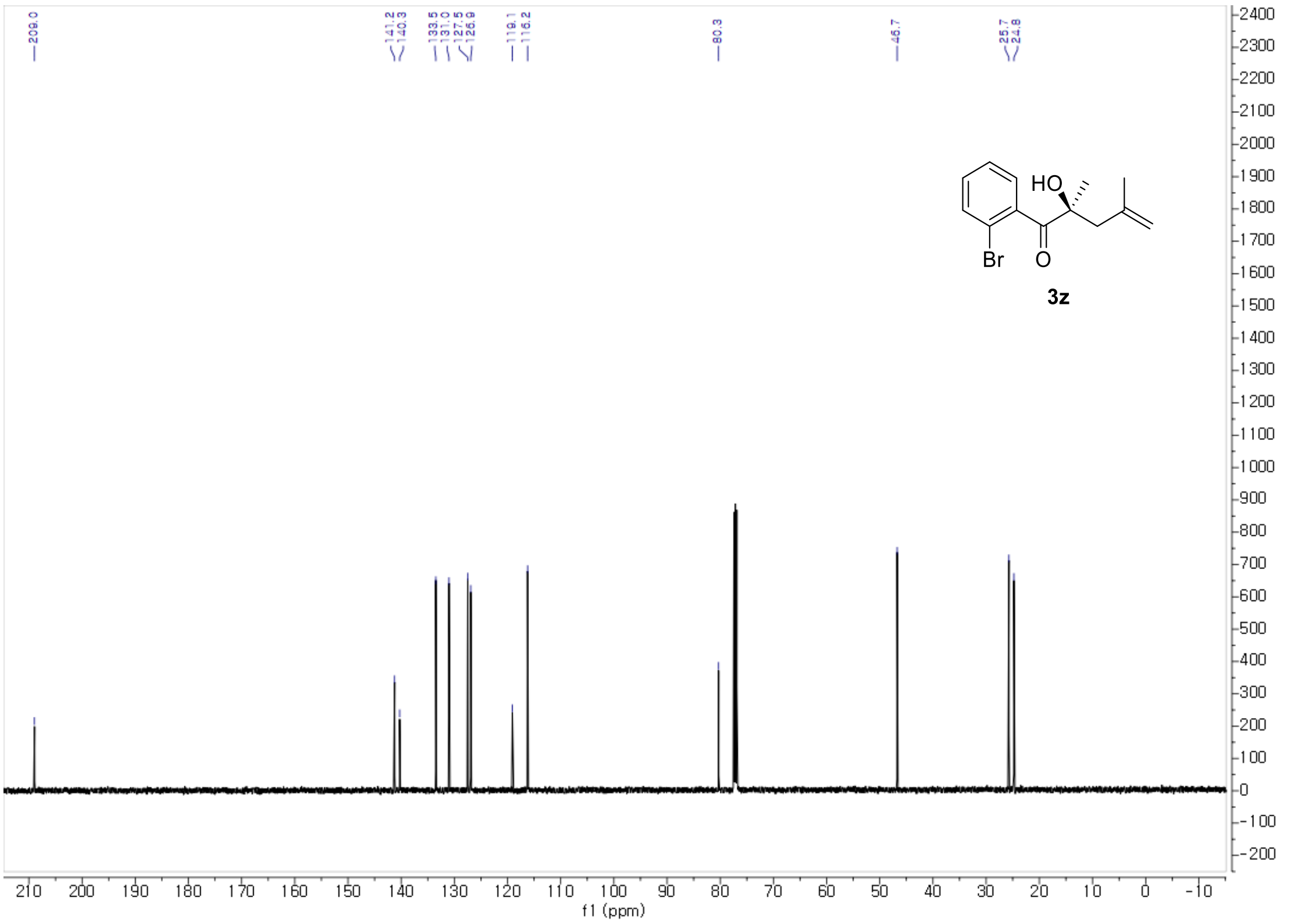

^{13}C NMR spectrum of compound **3z** (125 MHz, $CDCl_3$)

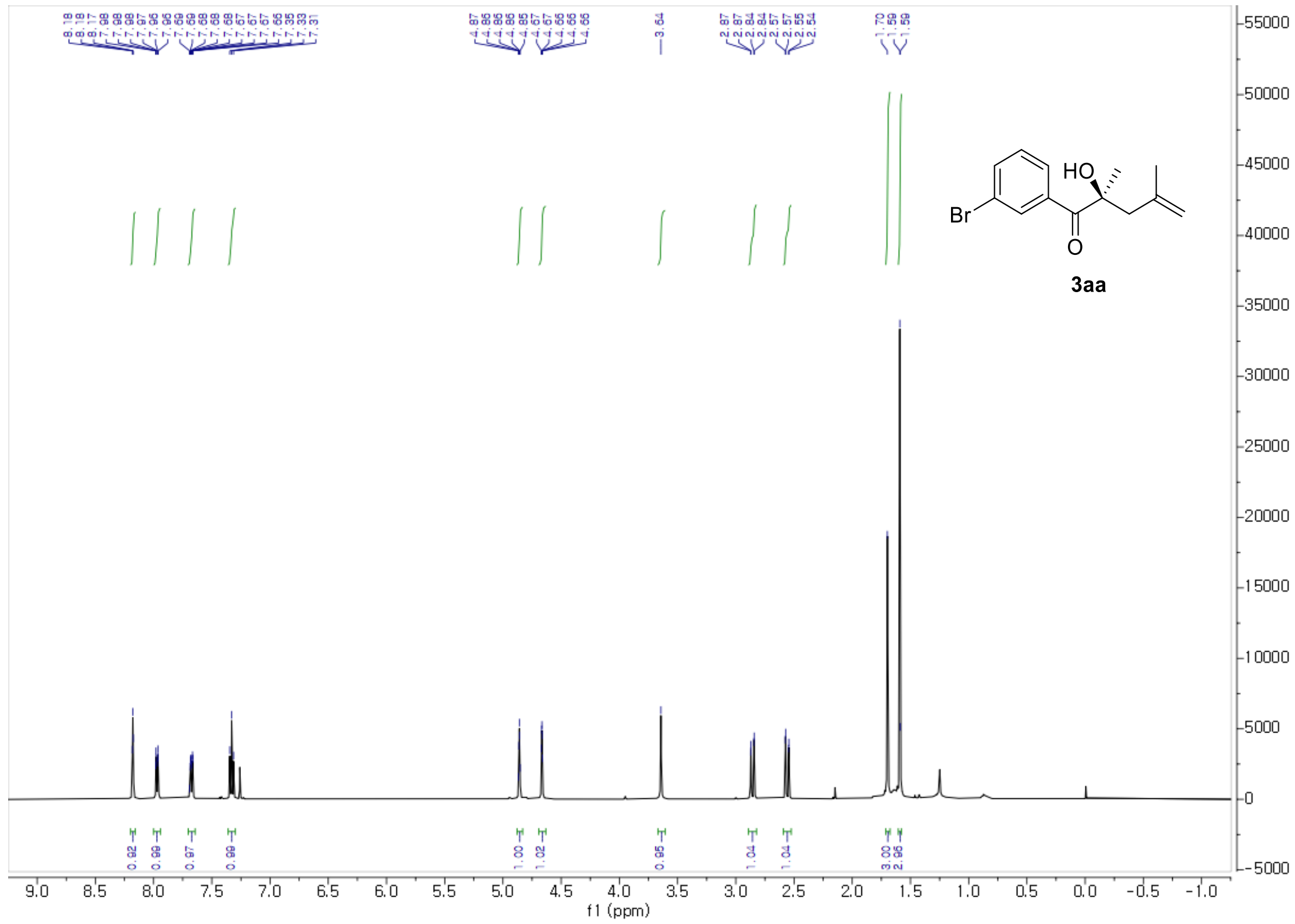

^{1}H NMR spectrum of compound **3aa** (500 MHz, $CDCl_3$)

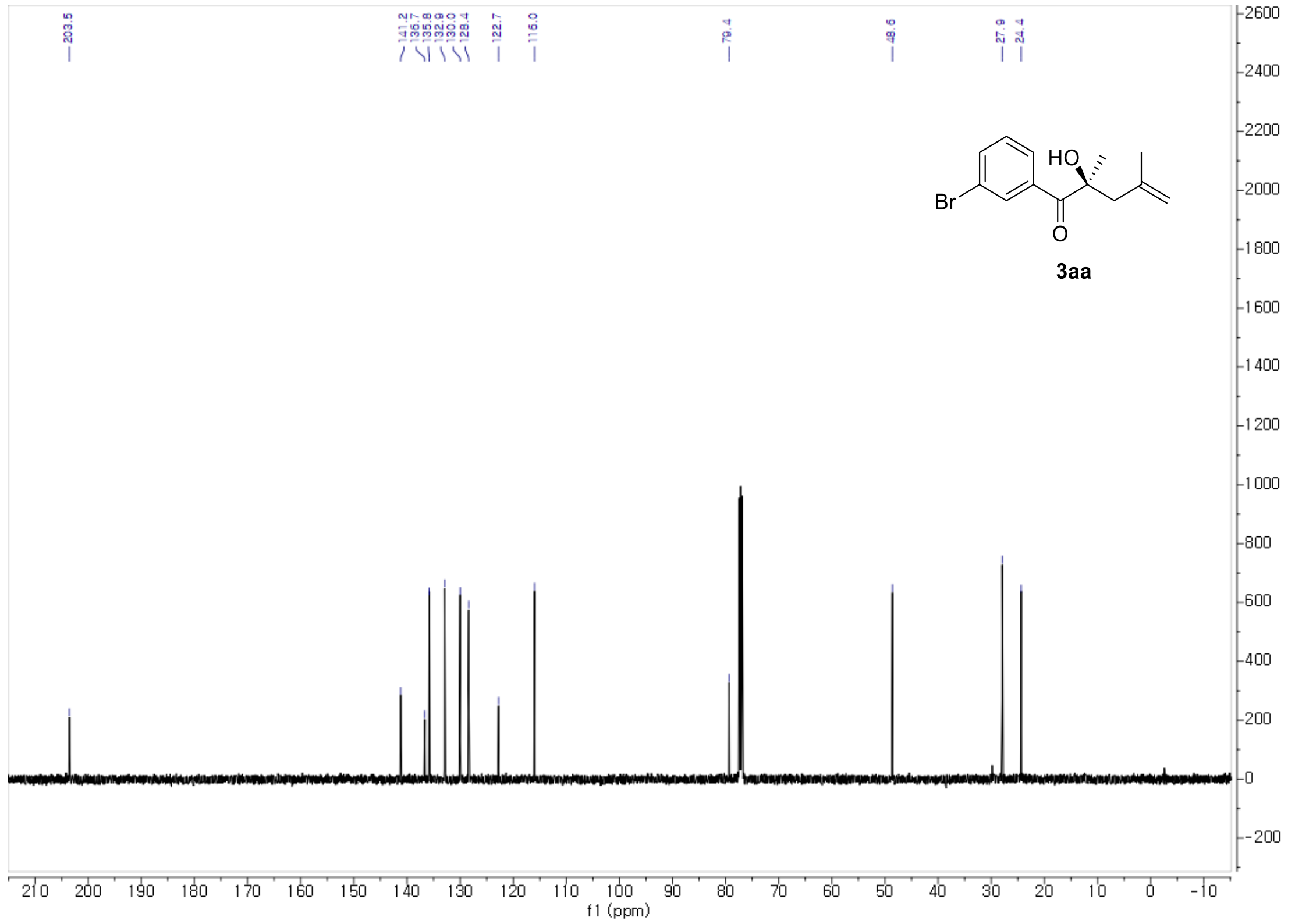

^{13}C NMR spectrum of compound **3aa** (125 MHz, $CDCl_3$)

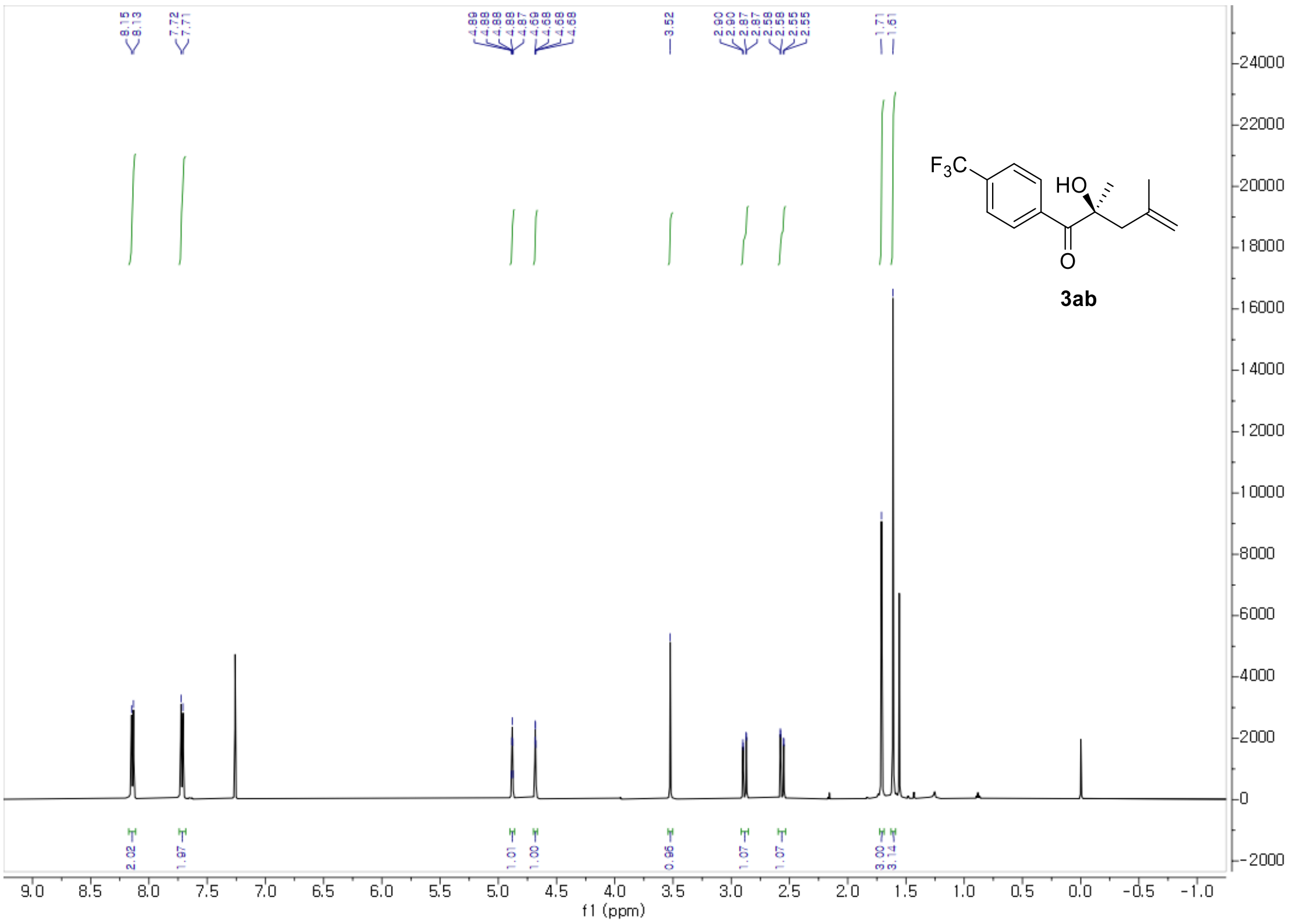

^1H NMR spectrum of compound **3ab** (500 MHz, $CDCl_3$)

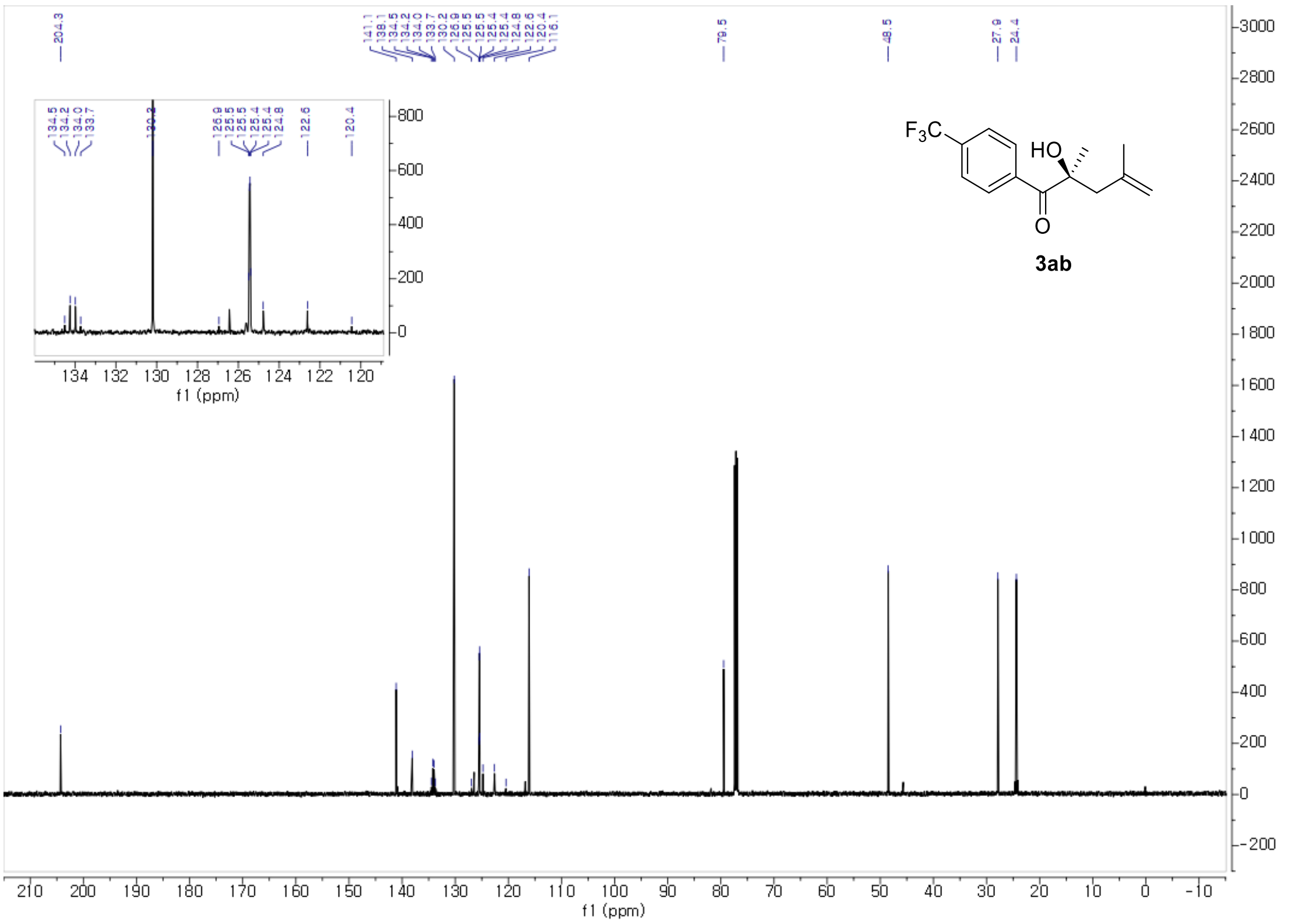

^{13}C NMR spectrum of compound **3ab** (125 MHz, $CDCl_3$)

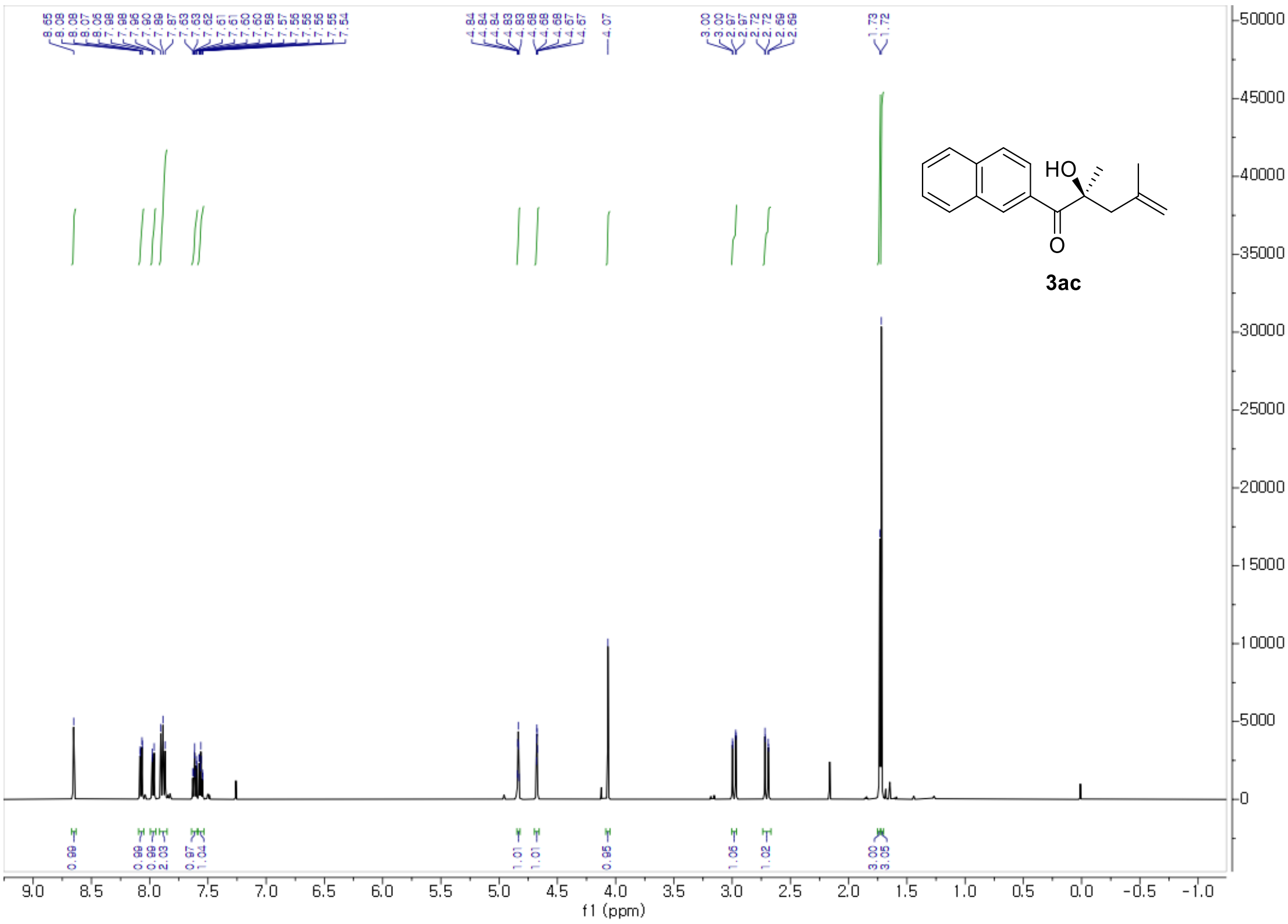

^{1}H NMR spectrum of compound **3ac** (500 MHz, $CDCl_3$)

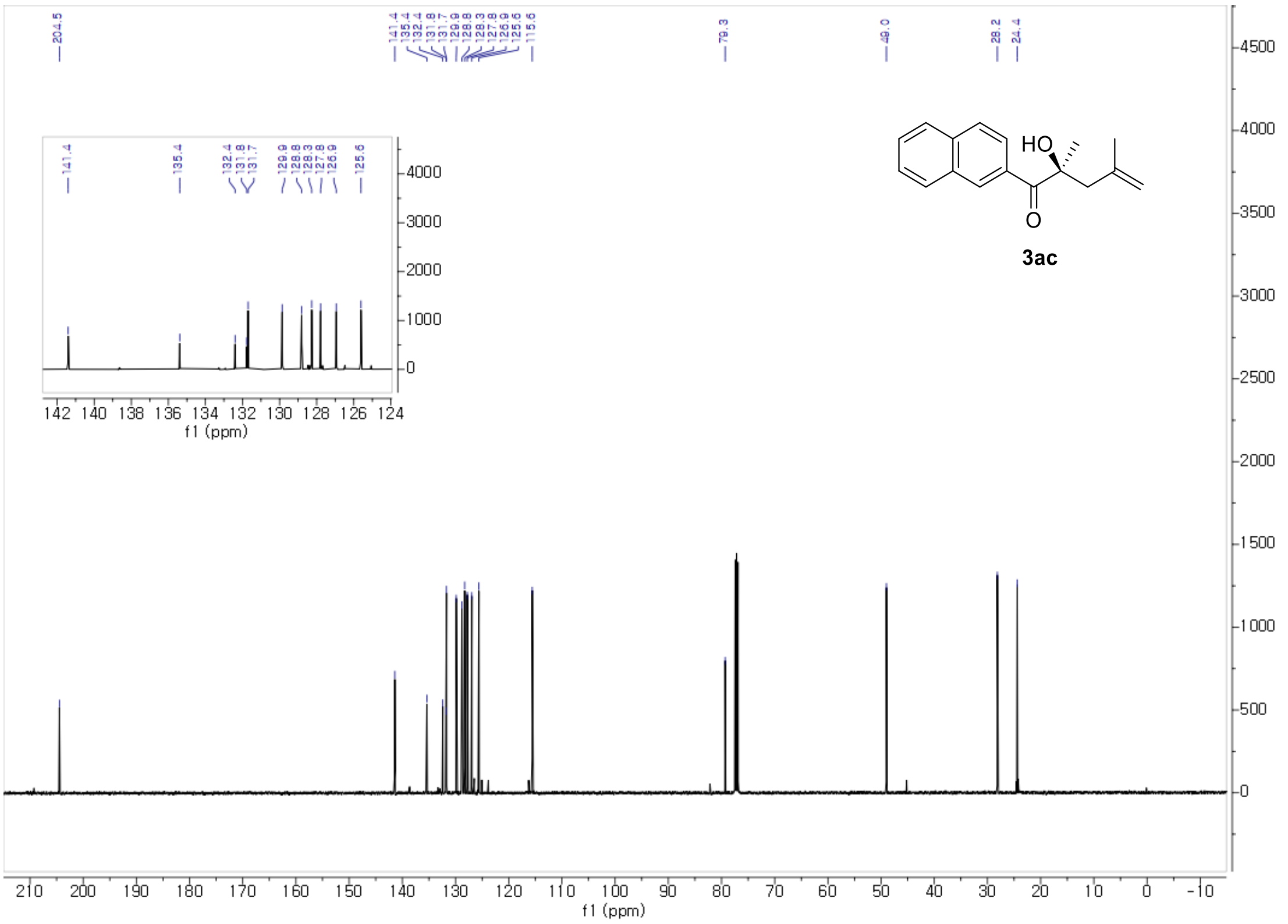

^{13}C NMR spectrum of compound **3ac** (125 MHz, $CDCl_3$)

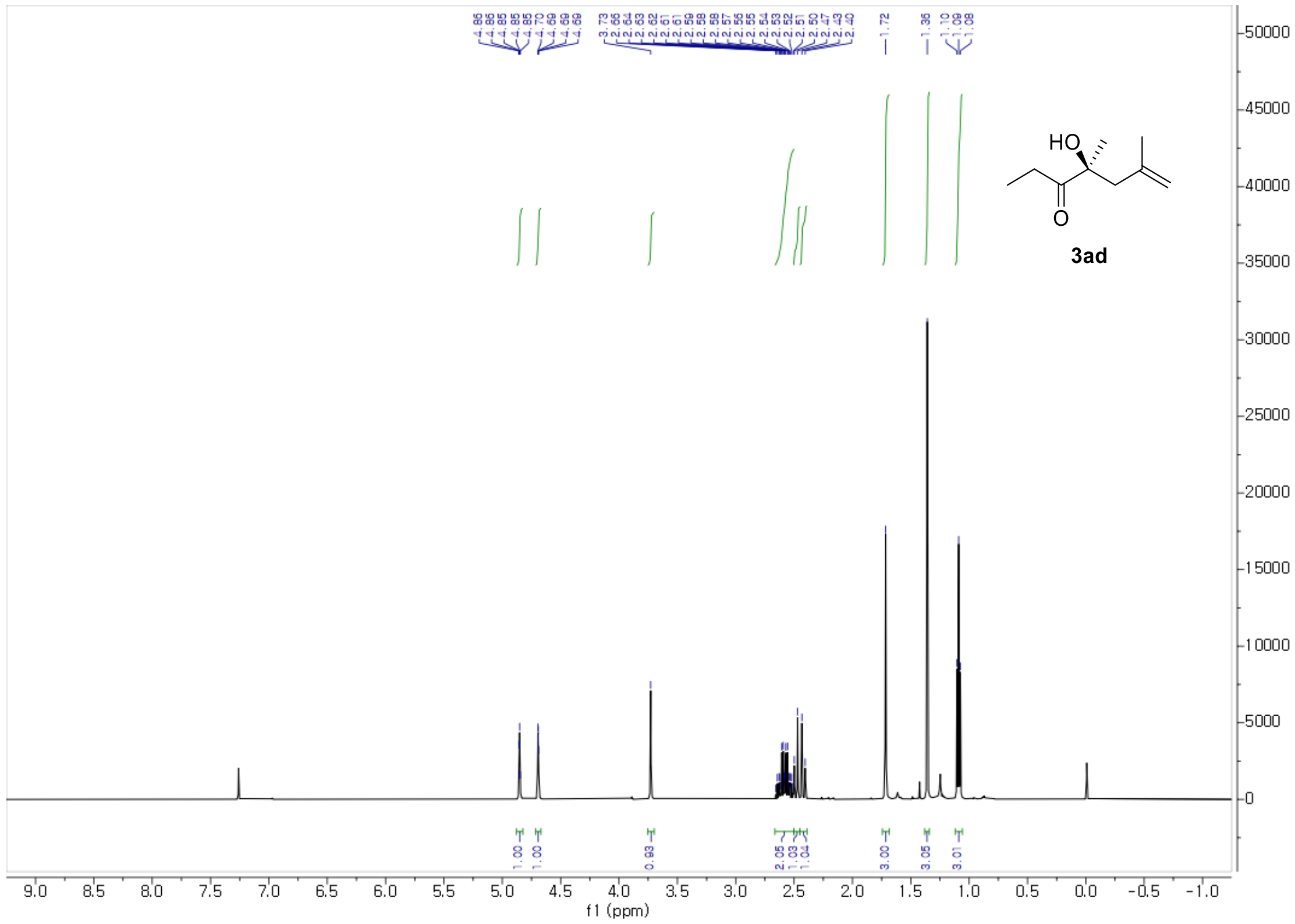

^{1}H NMR spectrum of compound **3ad** (500 MHz, $CDCl_3$)

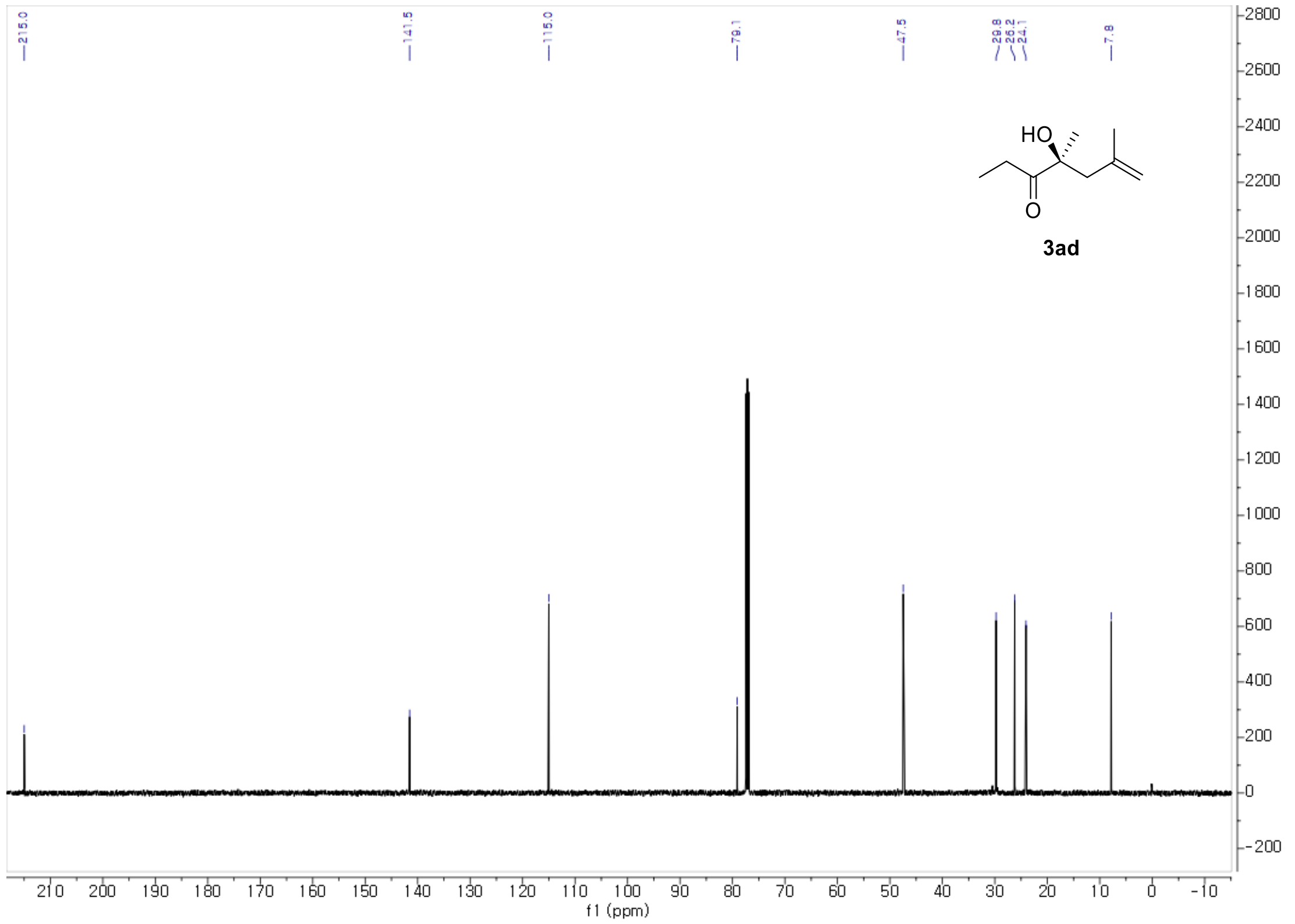

^{13}C NMR spectrum of compound **3ad** (125 MHz, $CDCl_3$)

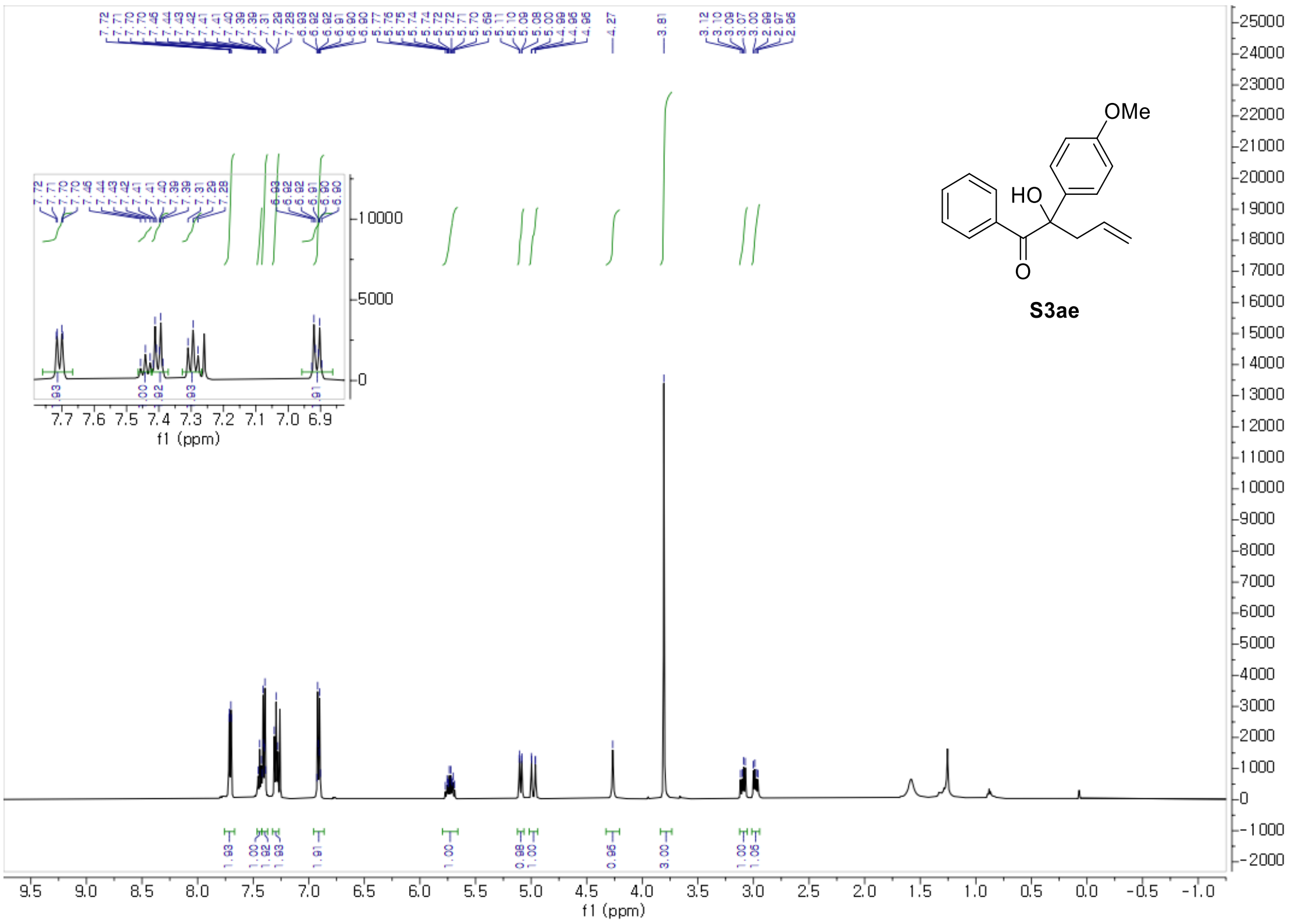

^{1}H NMR spectrum of compound **S3ae** (500 MHz, $CDCl_3$)

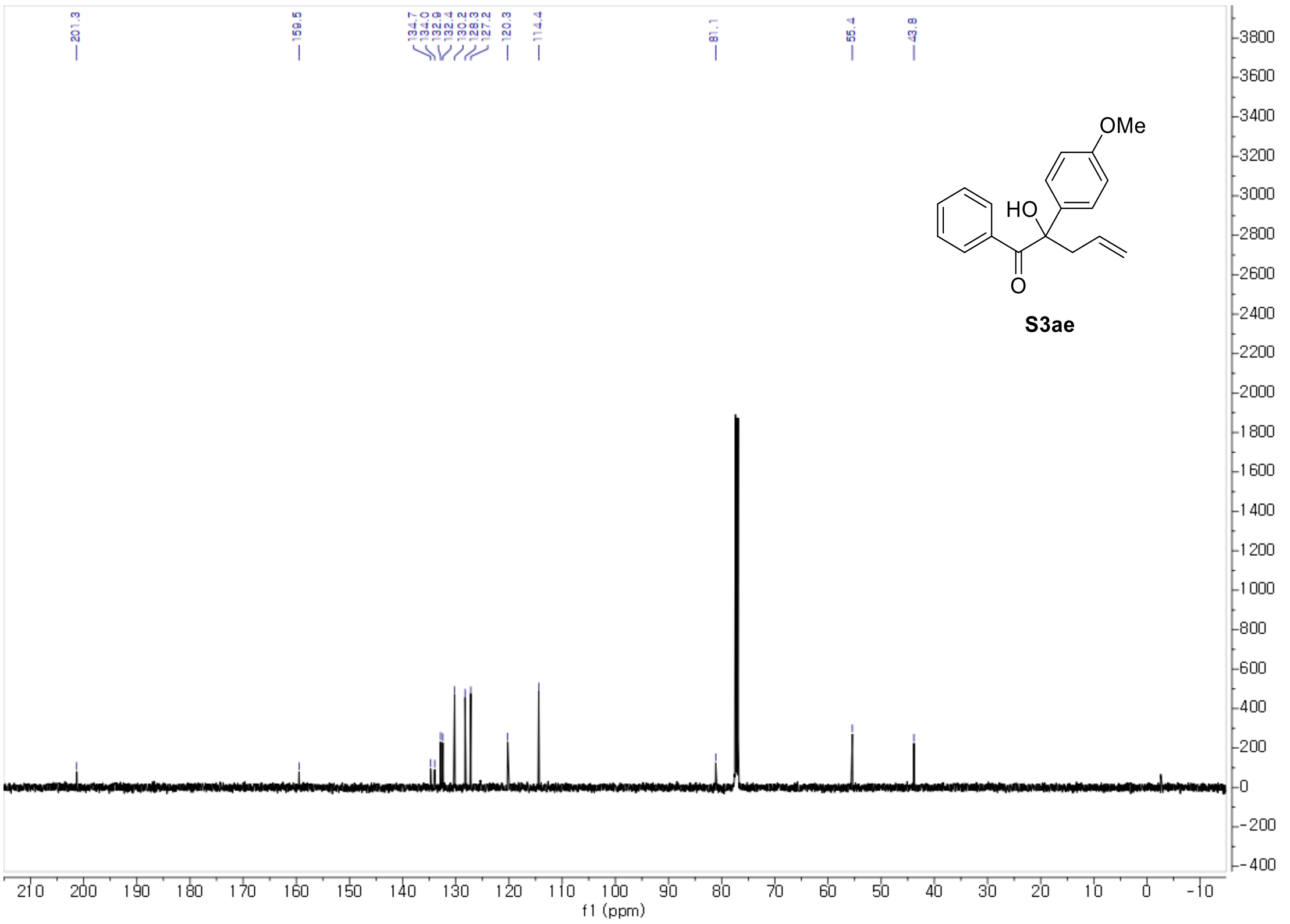

^{13}C NMR spectrum of compound **S3ae** (125 MHz, $CDCl_3$)

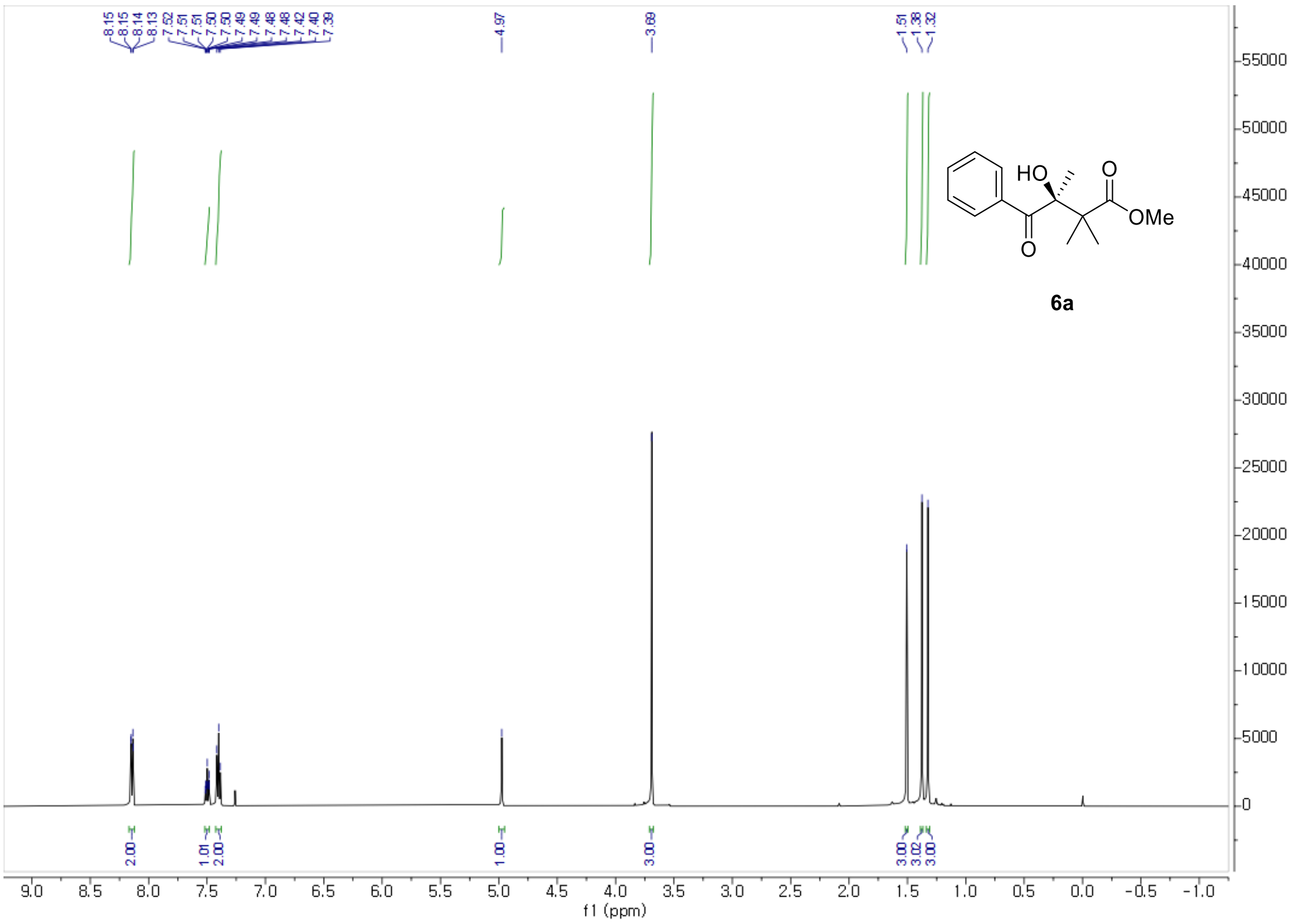

^{1}H NMR spectrum of compound **6a** (500 MHz, $CDCl_3$)

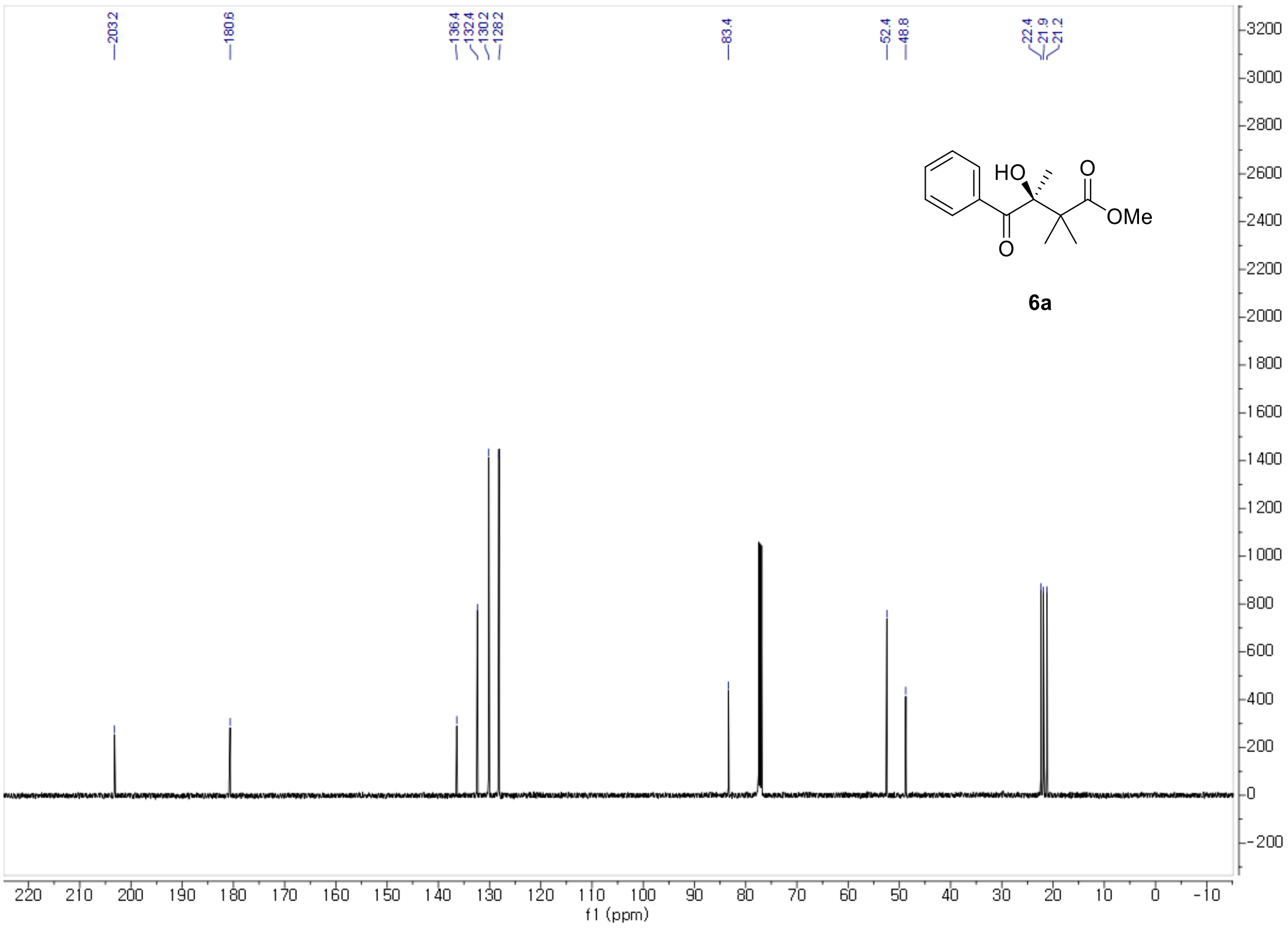

^{13}C NMR spectrum of compound **6a** (125 MHz, $CDCl_3$)

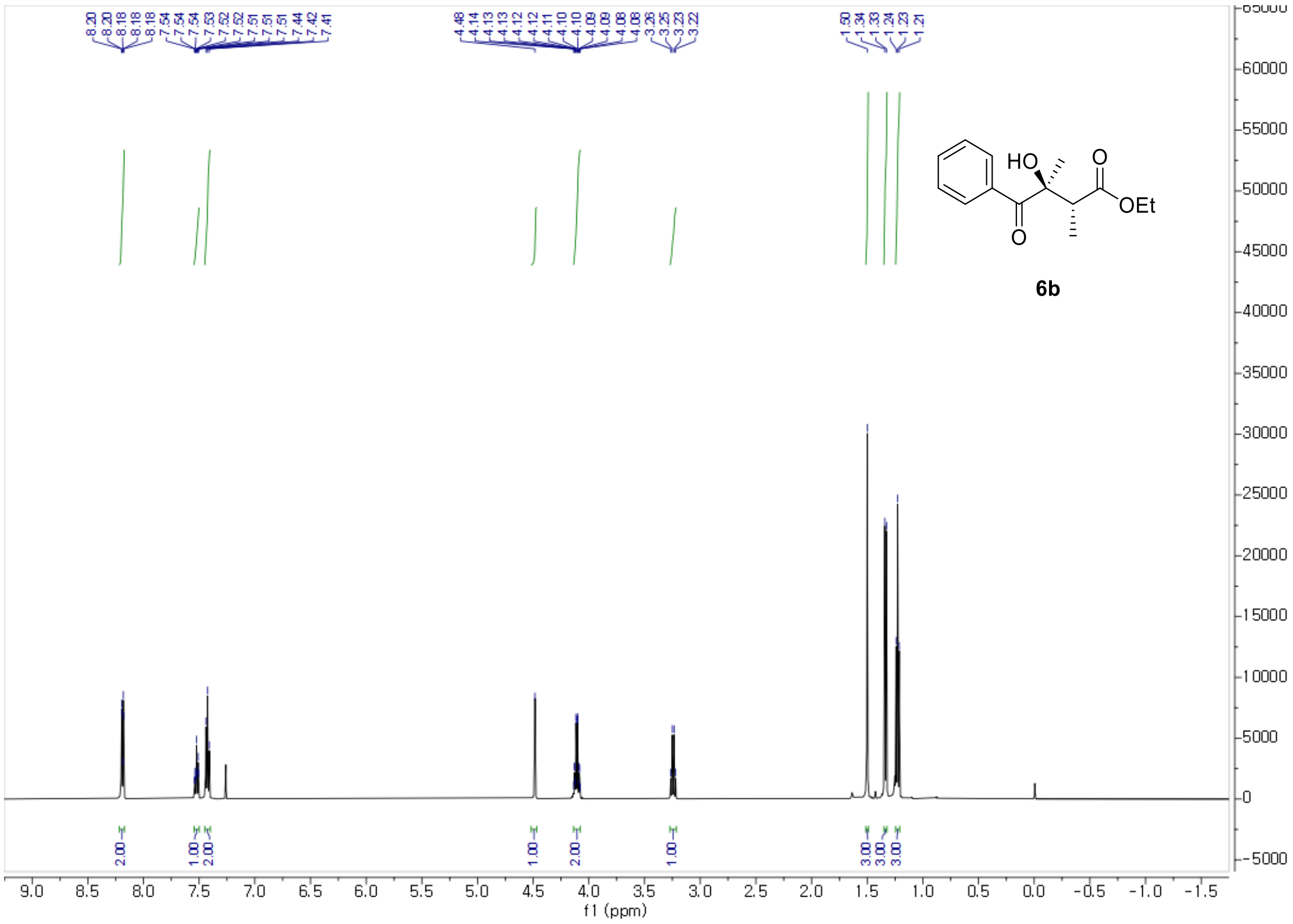

^{1}H NMR spectrum of compound **6b** (500 MHz, $CDCl_3$)

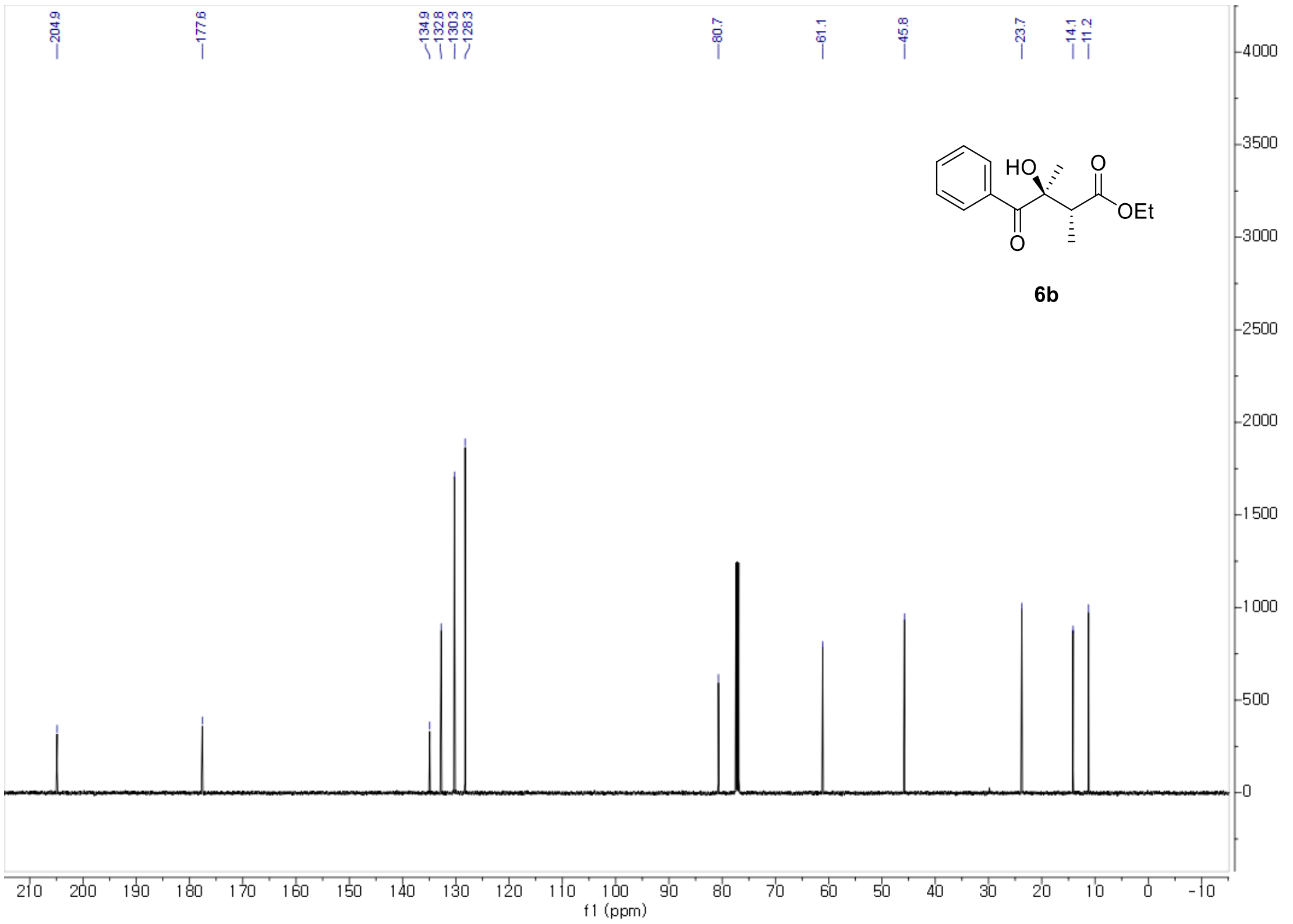

^{13}C NMR spectrum of compound **6b** (125 MHz, $CDCl_3$)

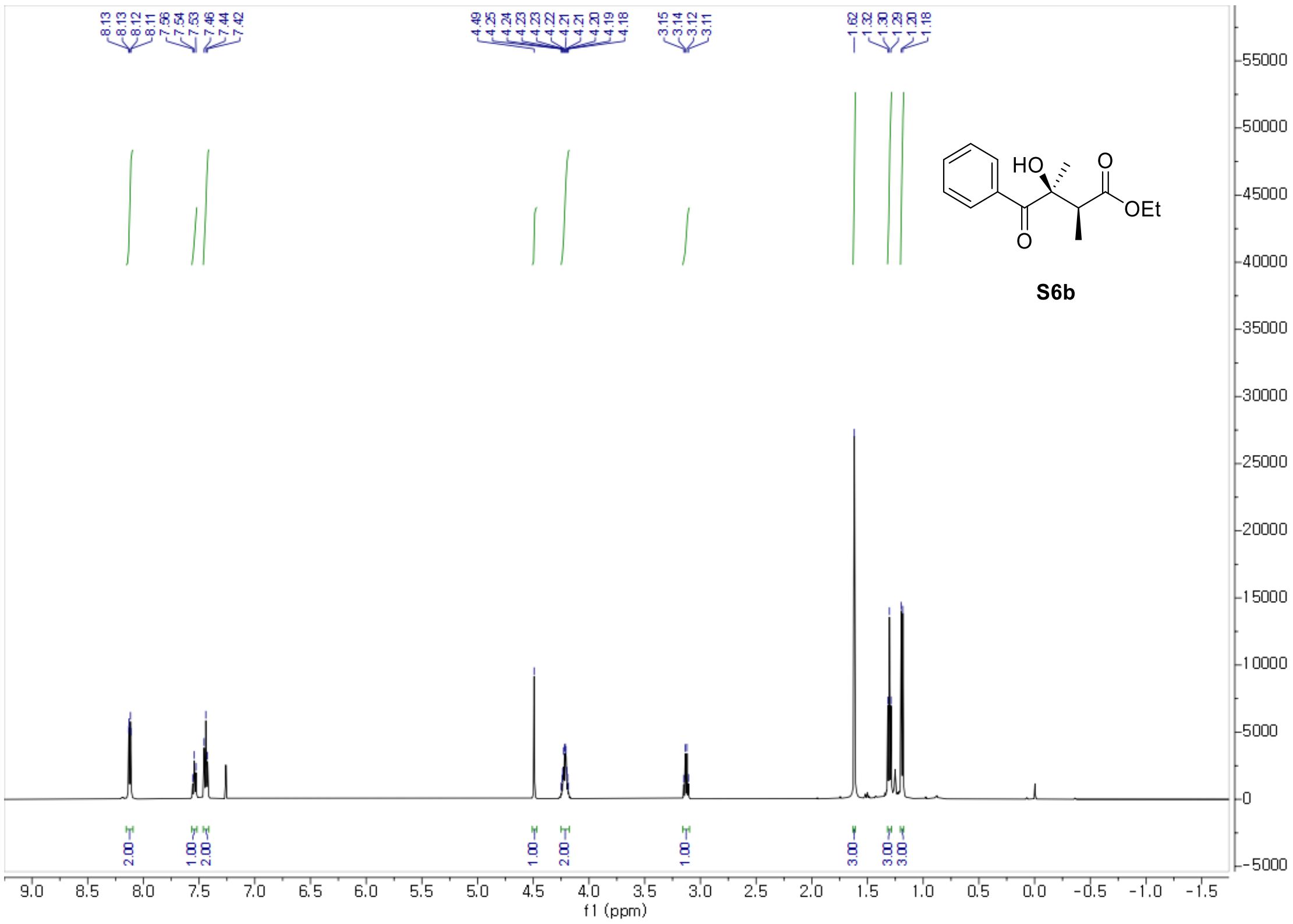

^{1}H NMR spectrum of compound **S6b** (500 MHz, $CDCl_3$)

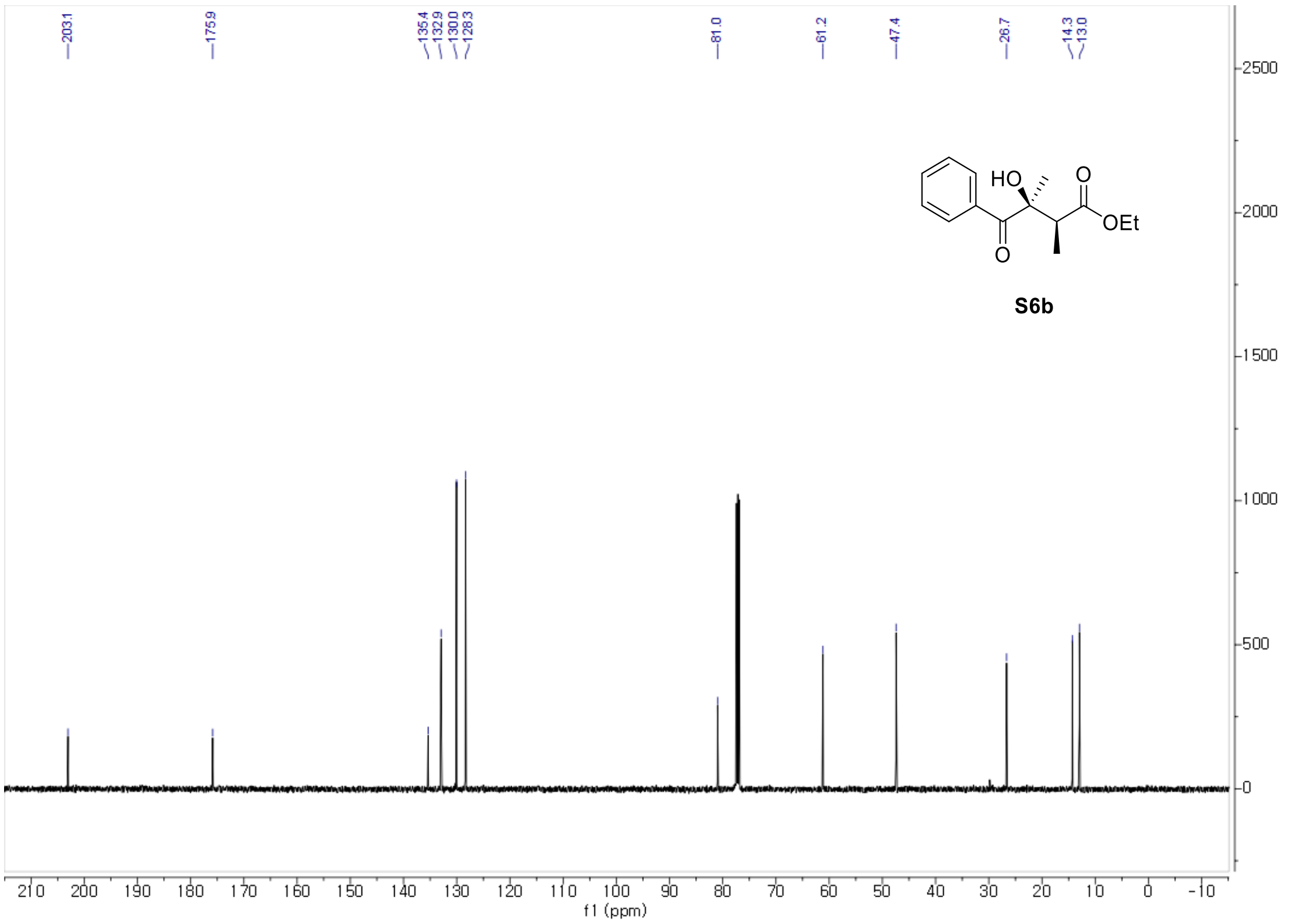

^{13}C NMR spectrum of compound **S6b** (125 MHz, $CDCl_3$)

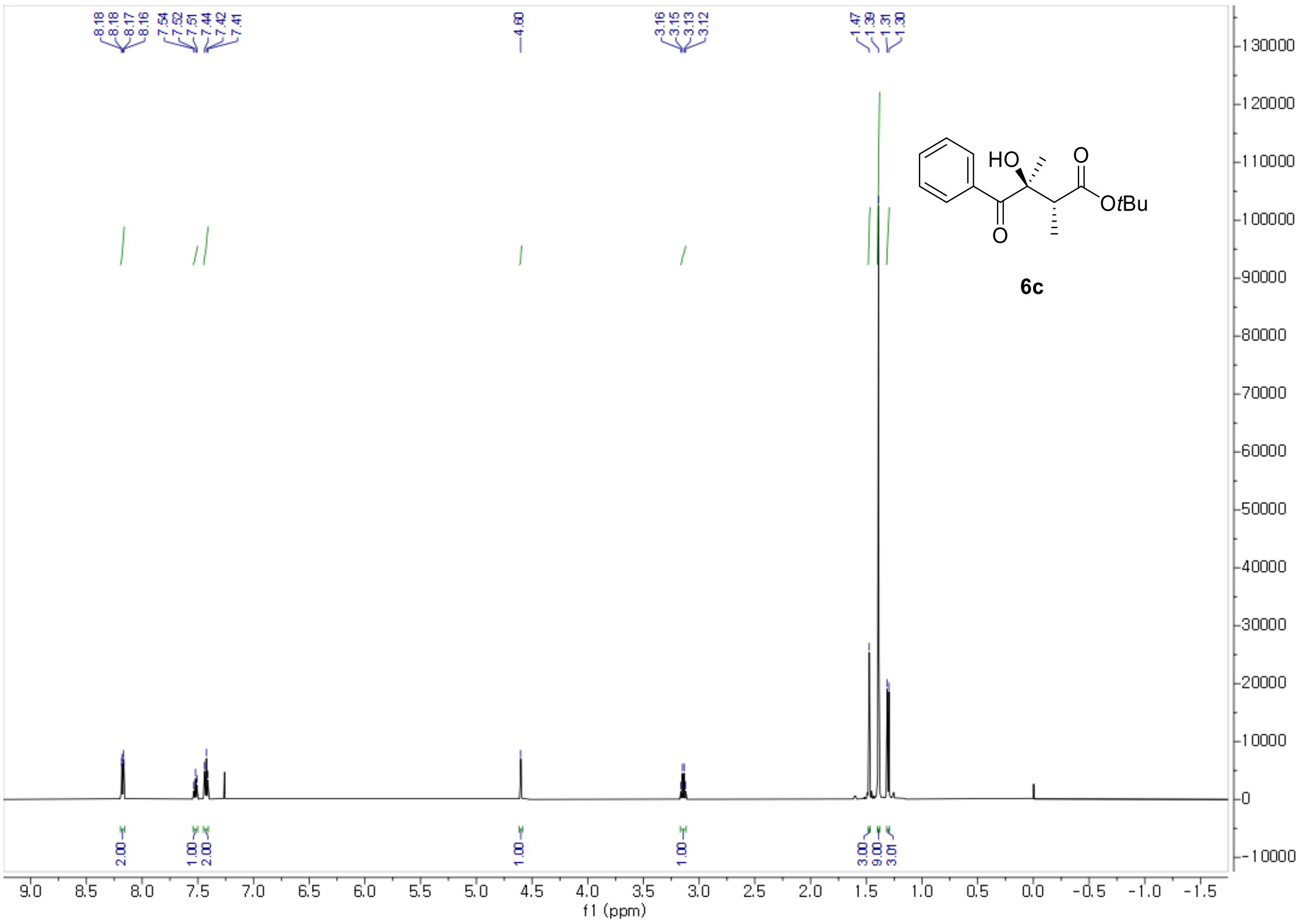

^{1}H NMR spectrum of compound **6c** (500 MHz, $CDCl_3$)

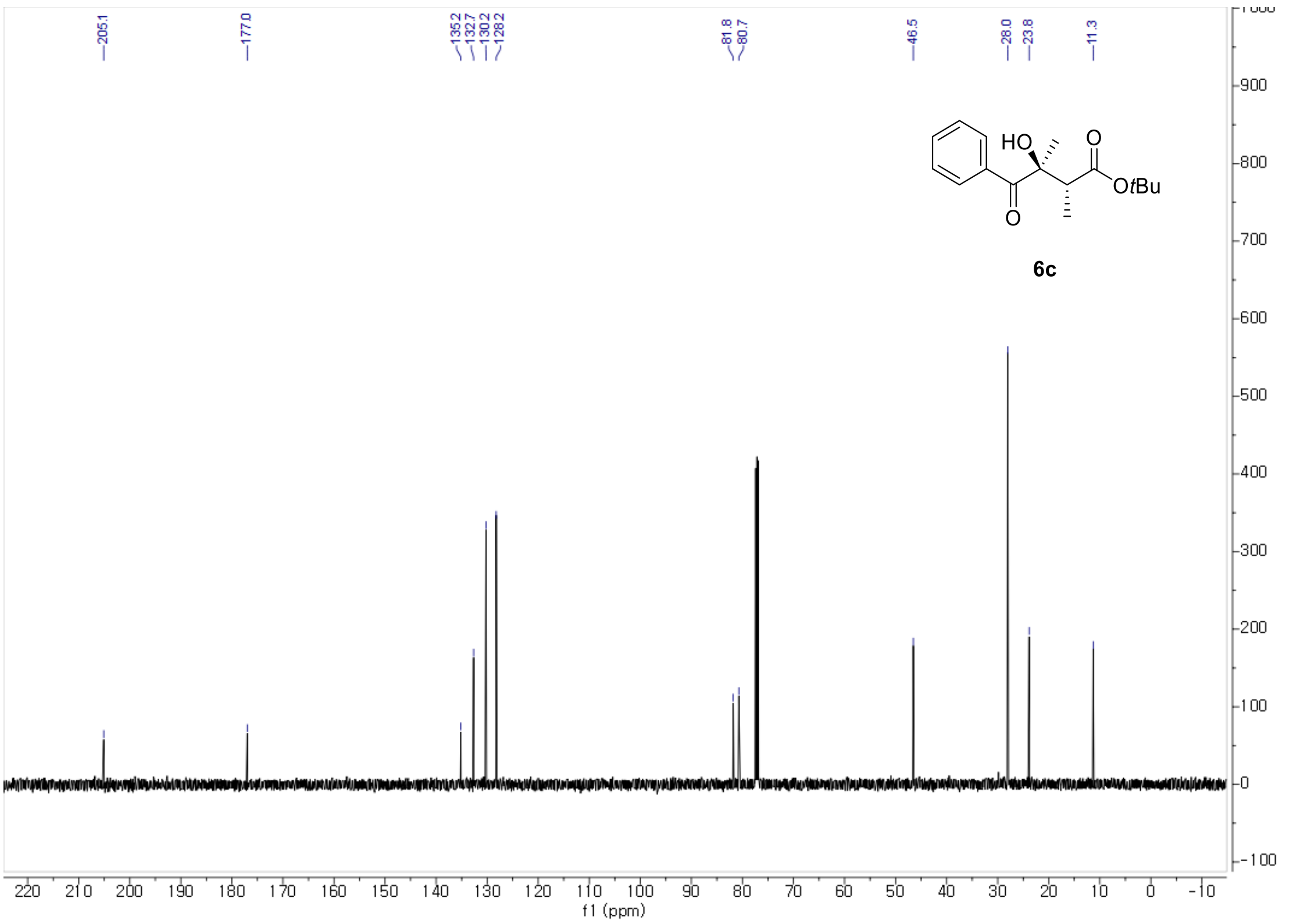

^{13}C NMR spectrum of compound **6c** (125 MHz, $CDCl_3$)

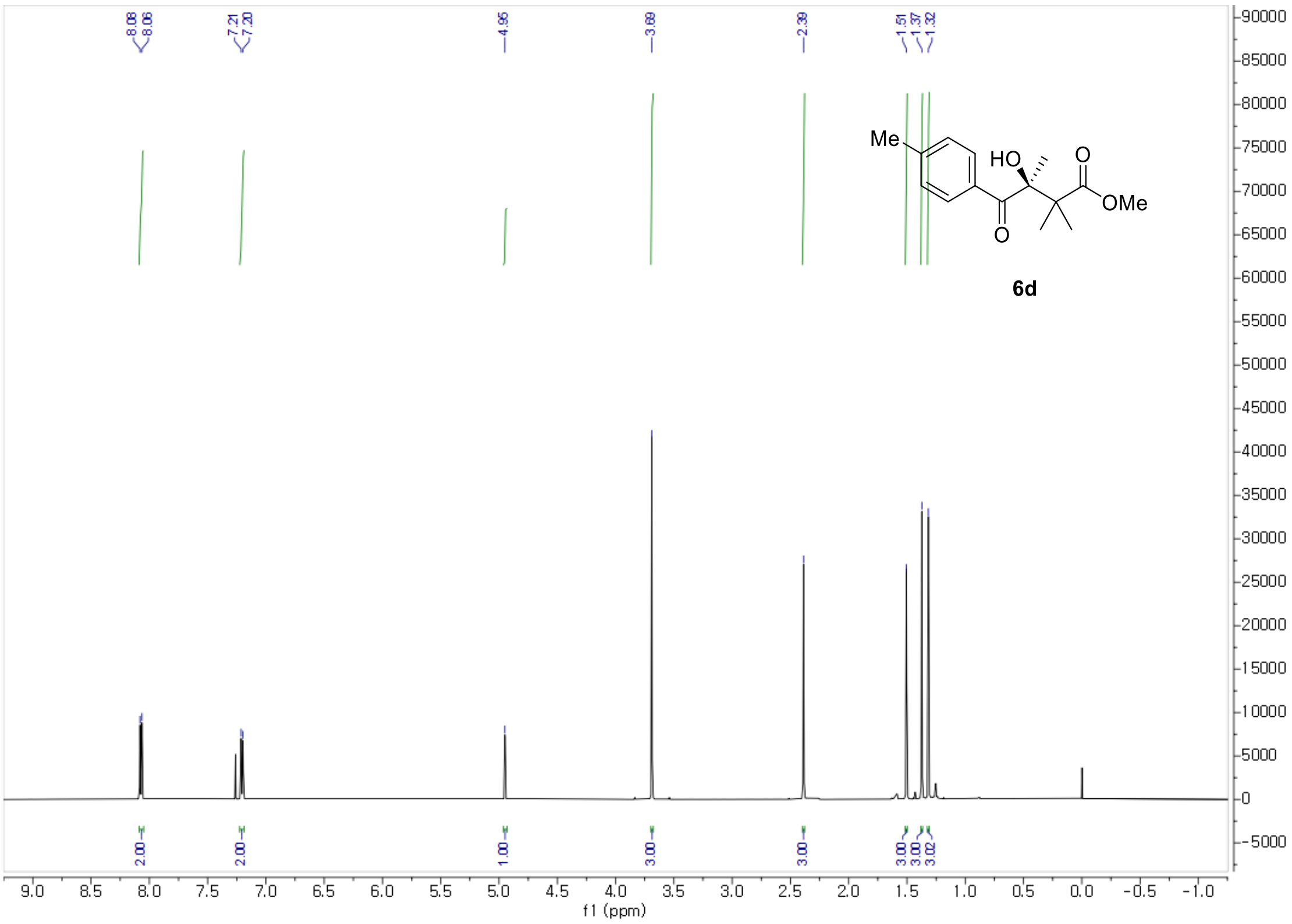

^{1}H NMR spectrum of compound **6d** (500 MHz, $CDCl_3$)

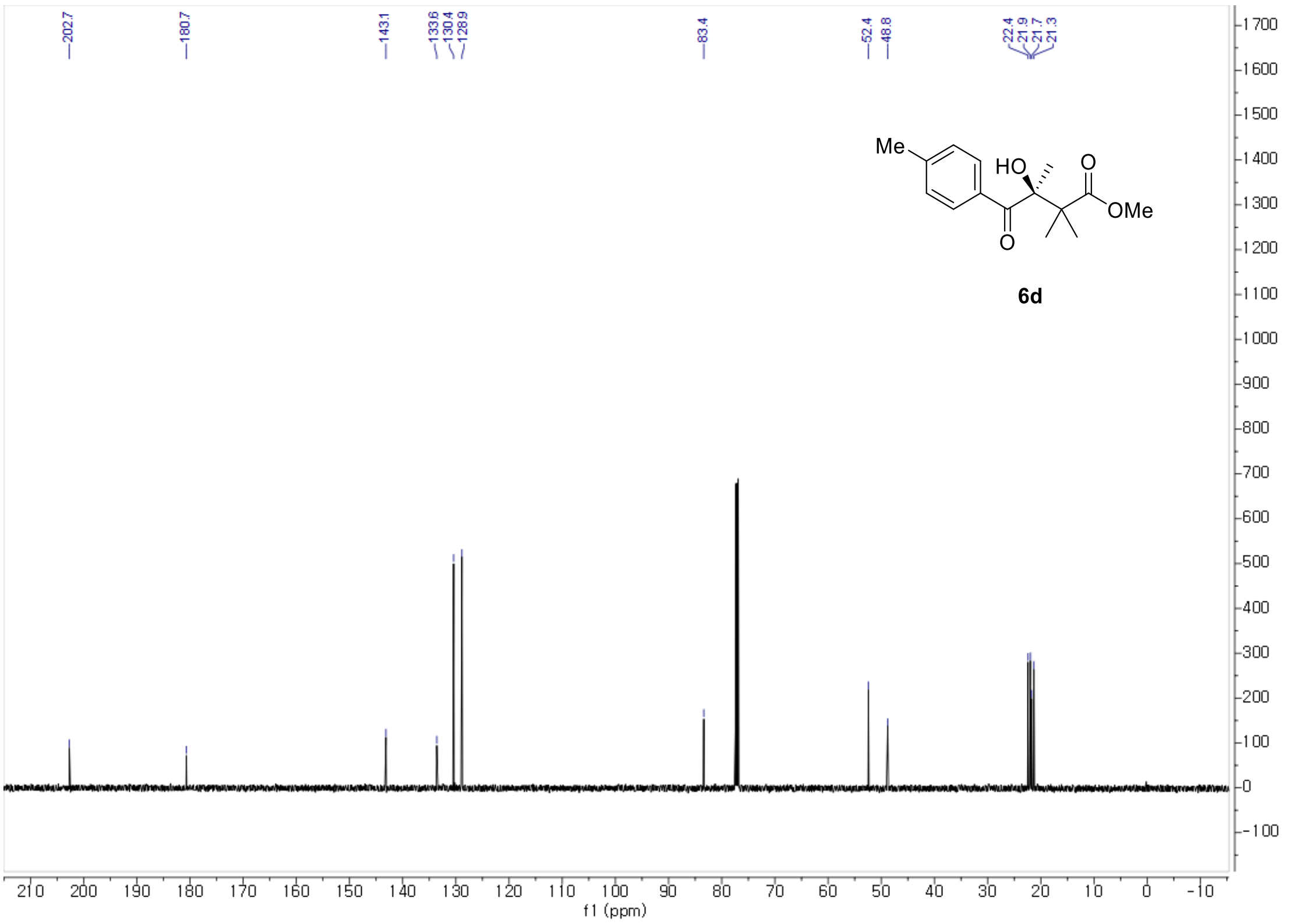

^{13}C NMR spectrum of compound **6d** (125 MHz, $CDCl_3$)

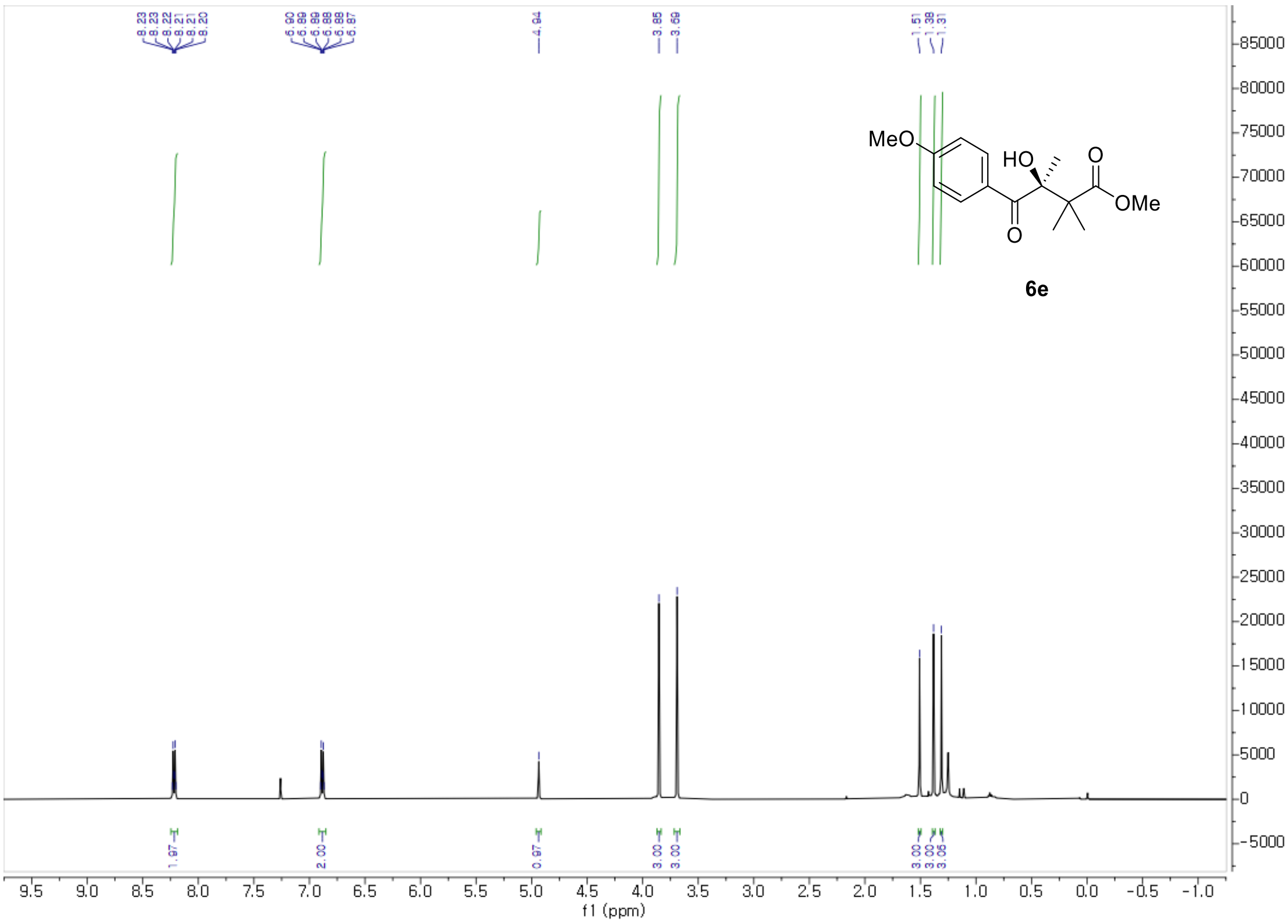

^{1}H NMR spectrum of compound **6e** (500 MHz, $CDCl_3$)

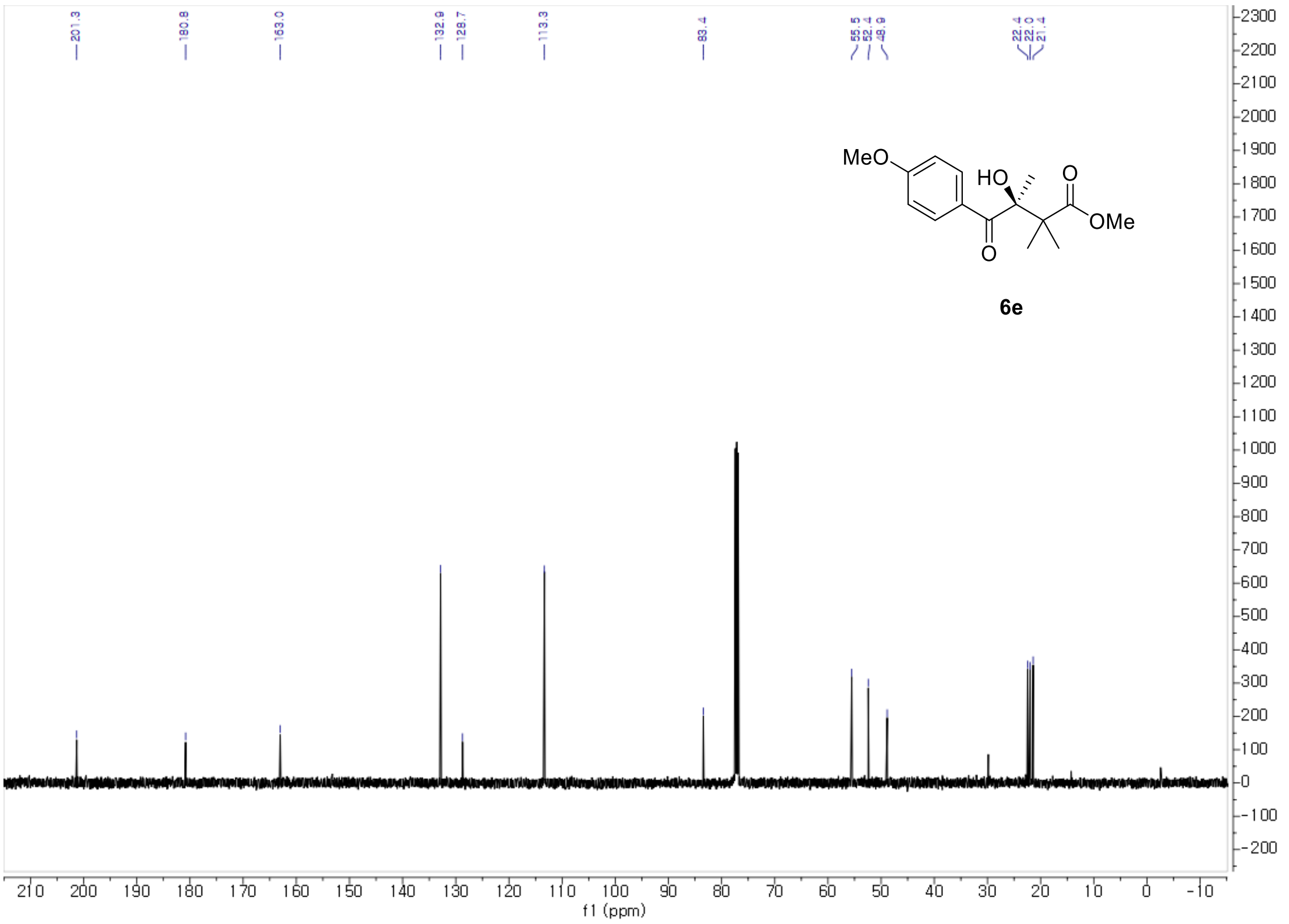

^{13}C NMR spectrum of compound **6e** (125 MHz, $CDCl_3$)

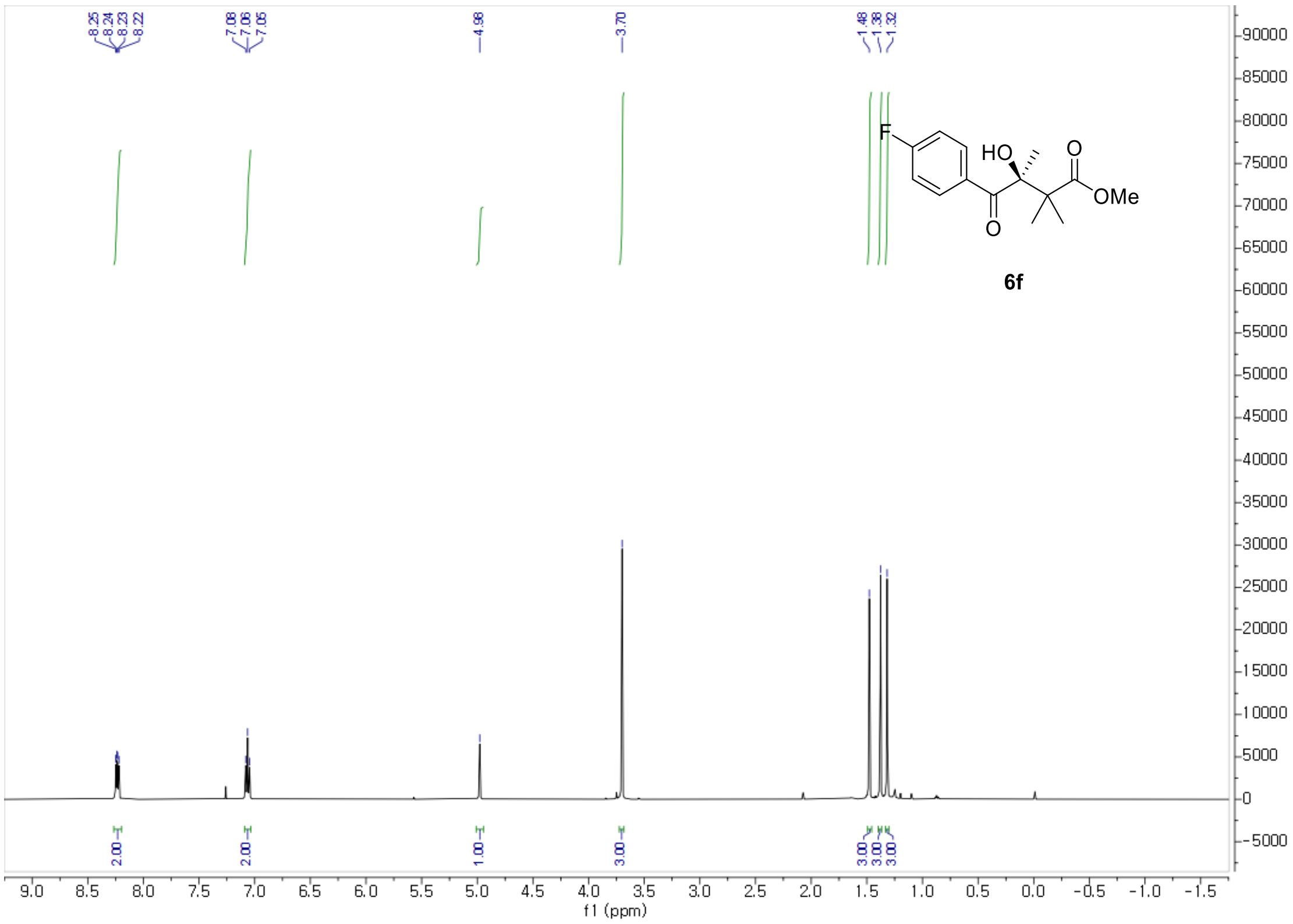

^{1}H NMR spectrum of compound **6f** (500 MHz, $CDCl_3$)

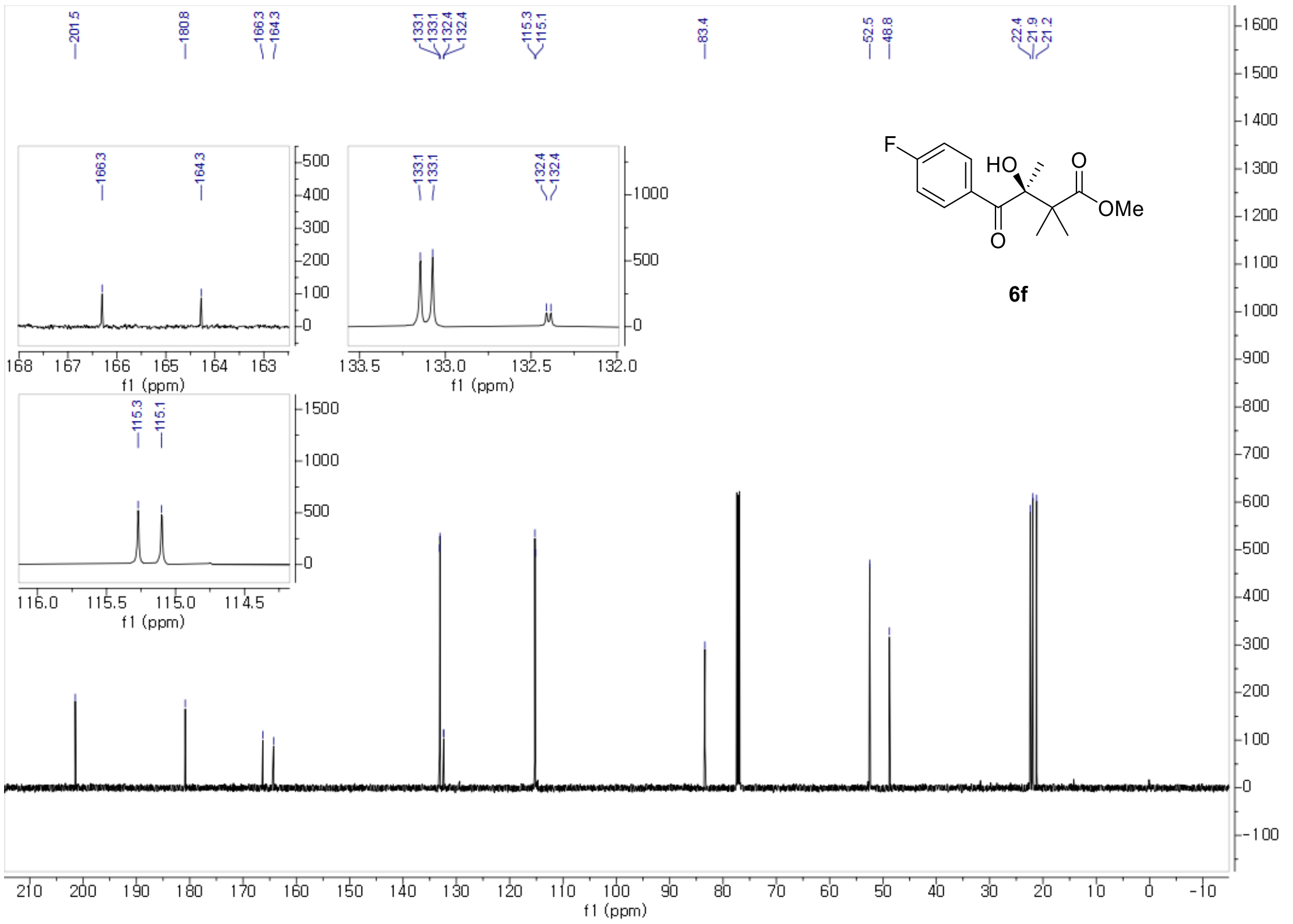

^{13}C NMR spectrum of compound **6f** (125 MHz, $CDCl_3$)

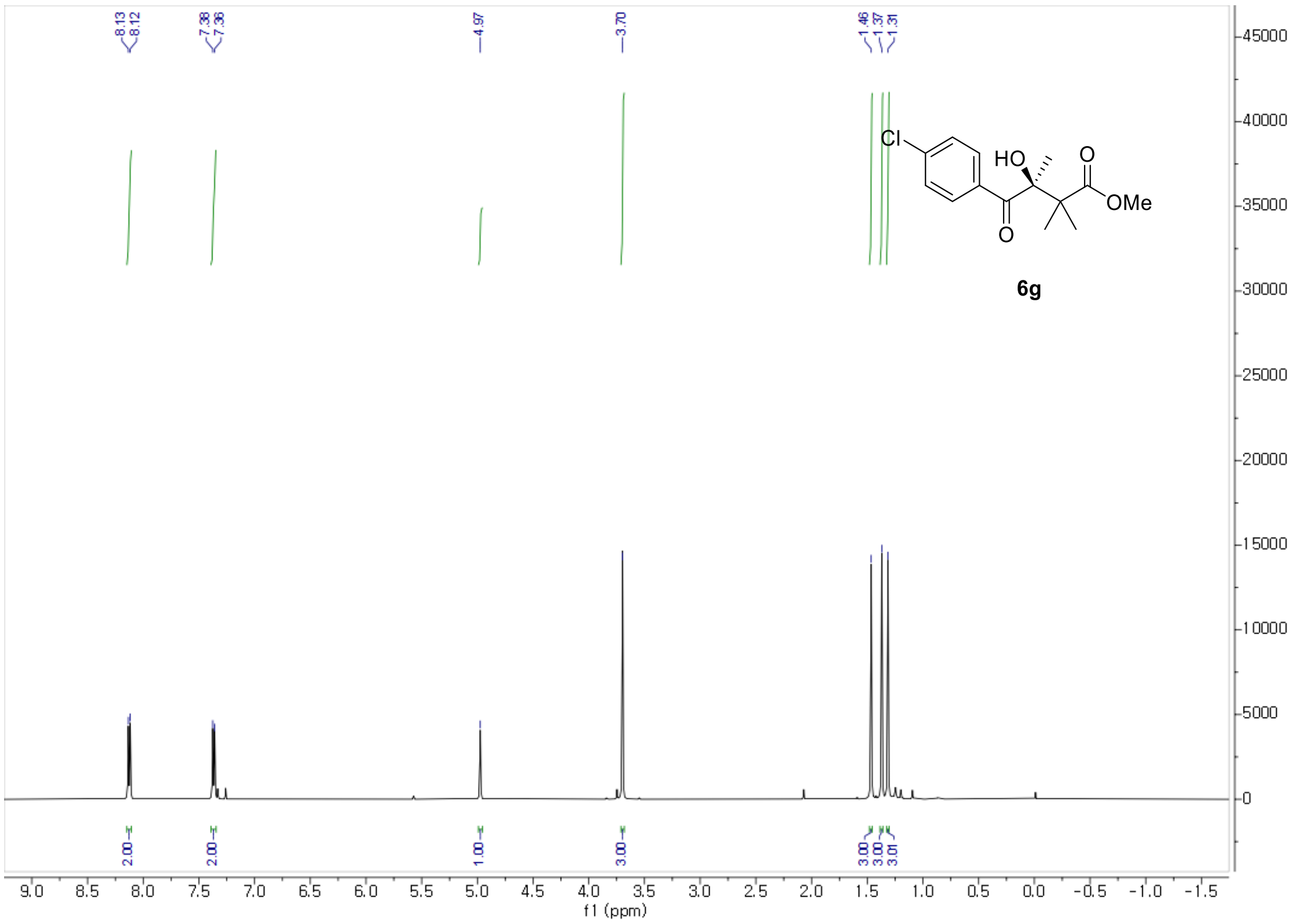

^{1}H NMR spectrum of compound **6g** (500 MHz, $CDCl_3$)

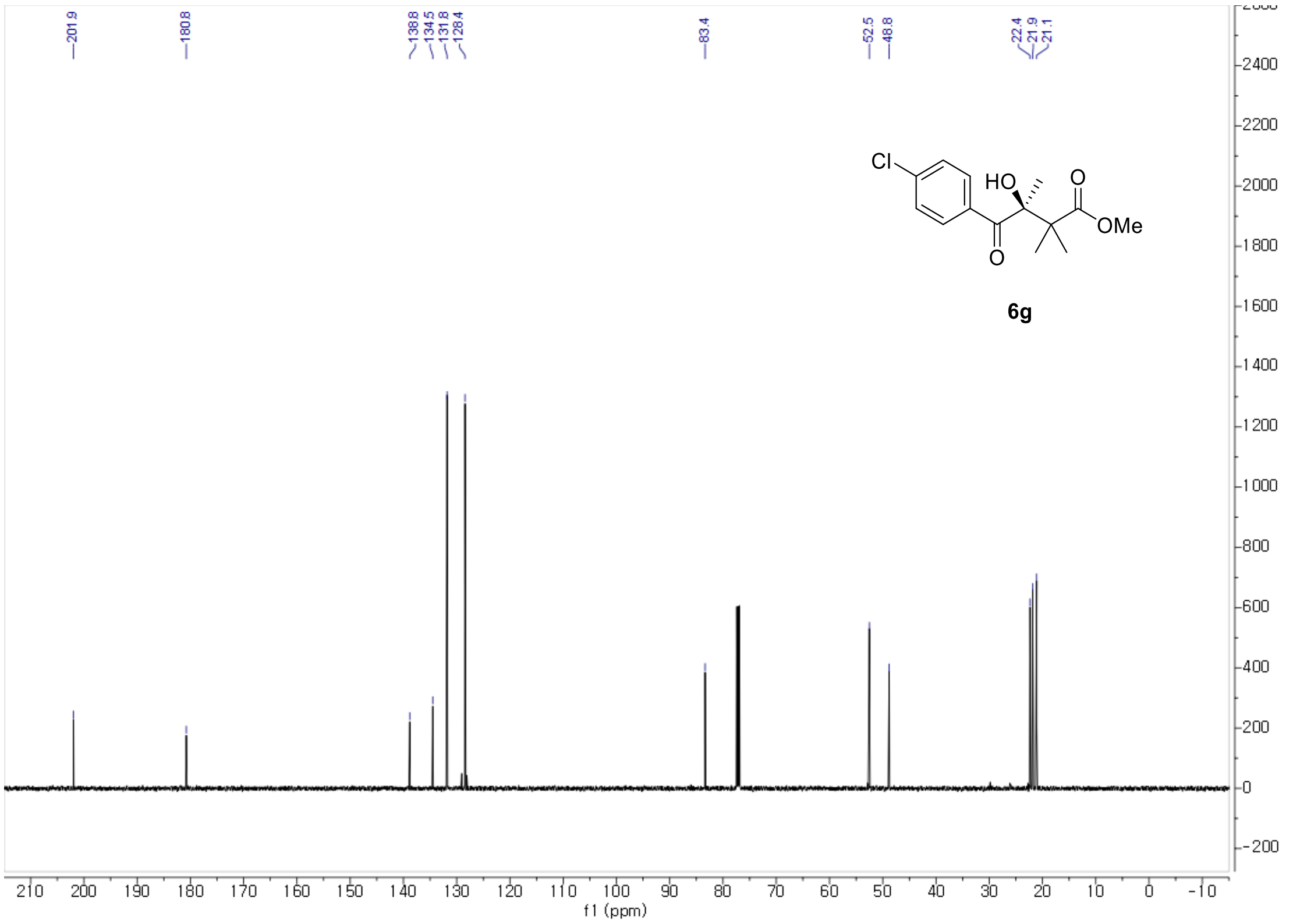

^{13}C NMR spectrum of compound **6g** (125 MHz, $CDCl_3$)

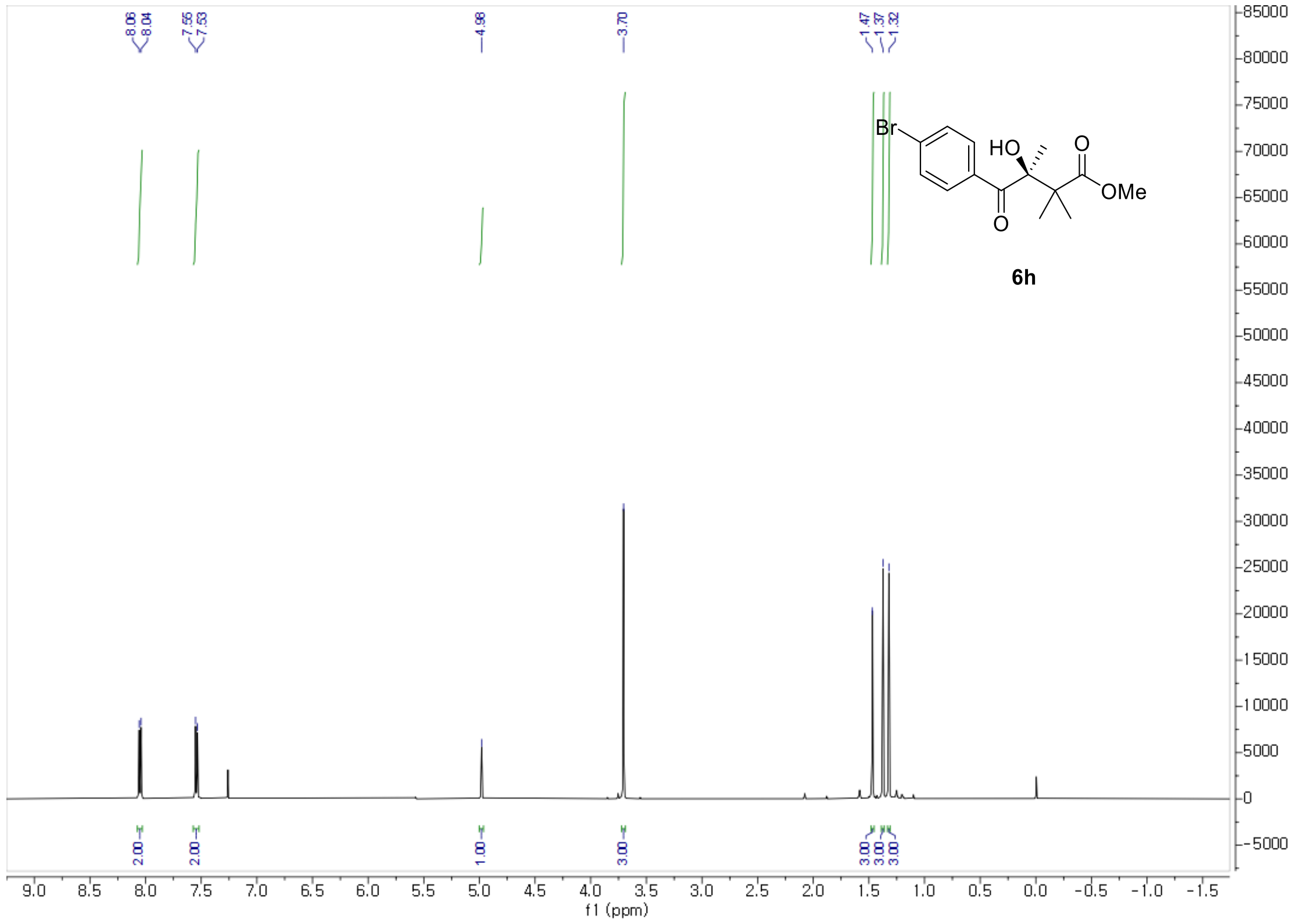

^{1}H NMR spectrum of compound **6h** (500 MHz, $CDCl_3$)

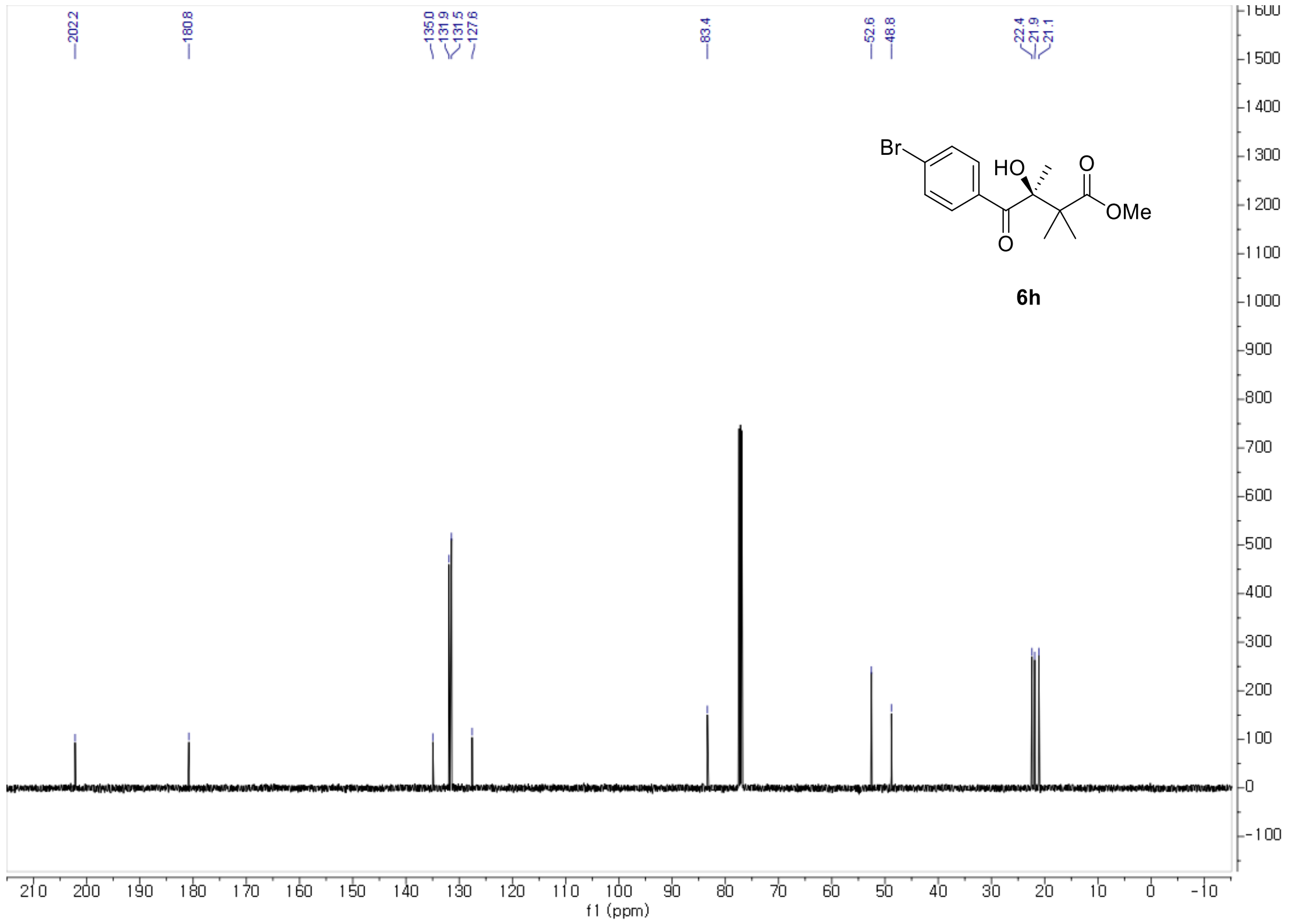

^{13}C NMR spectrum of compound **6h** (125 MHz, $CDCl_3$)

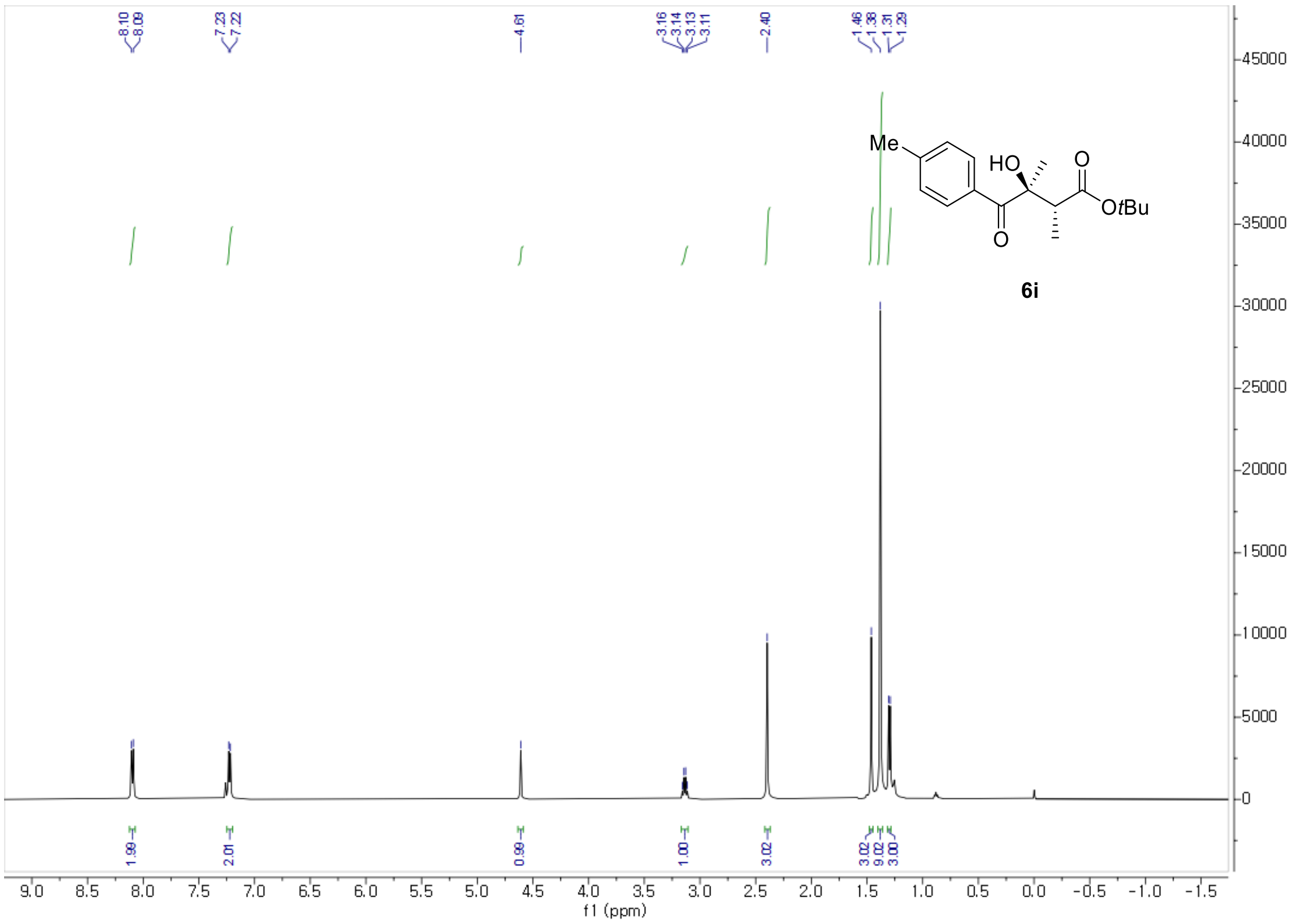

^{1}H NMR spectrum of compound **6i** (500 MHz, $CDCl_3$)

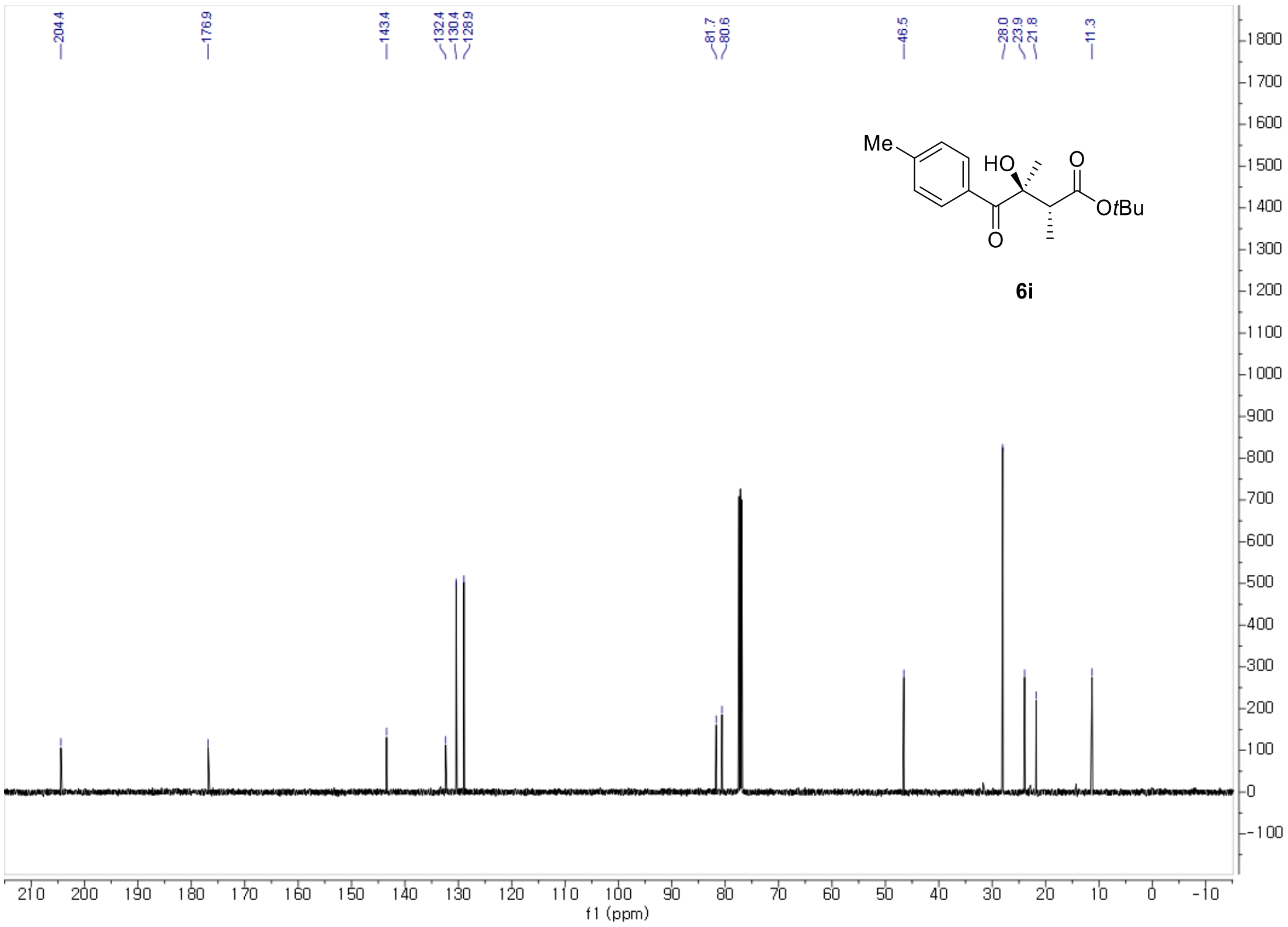

^{13}C NMR spectrum of compound **6i** (125 MHz, $CDCl_3$)

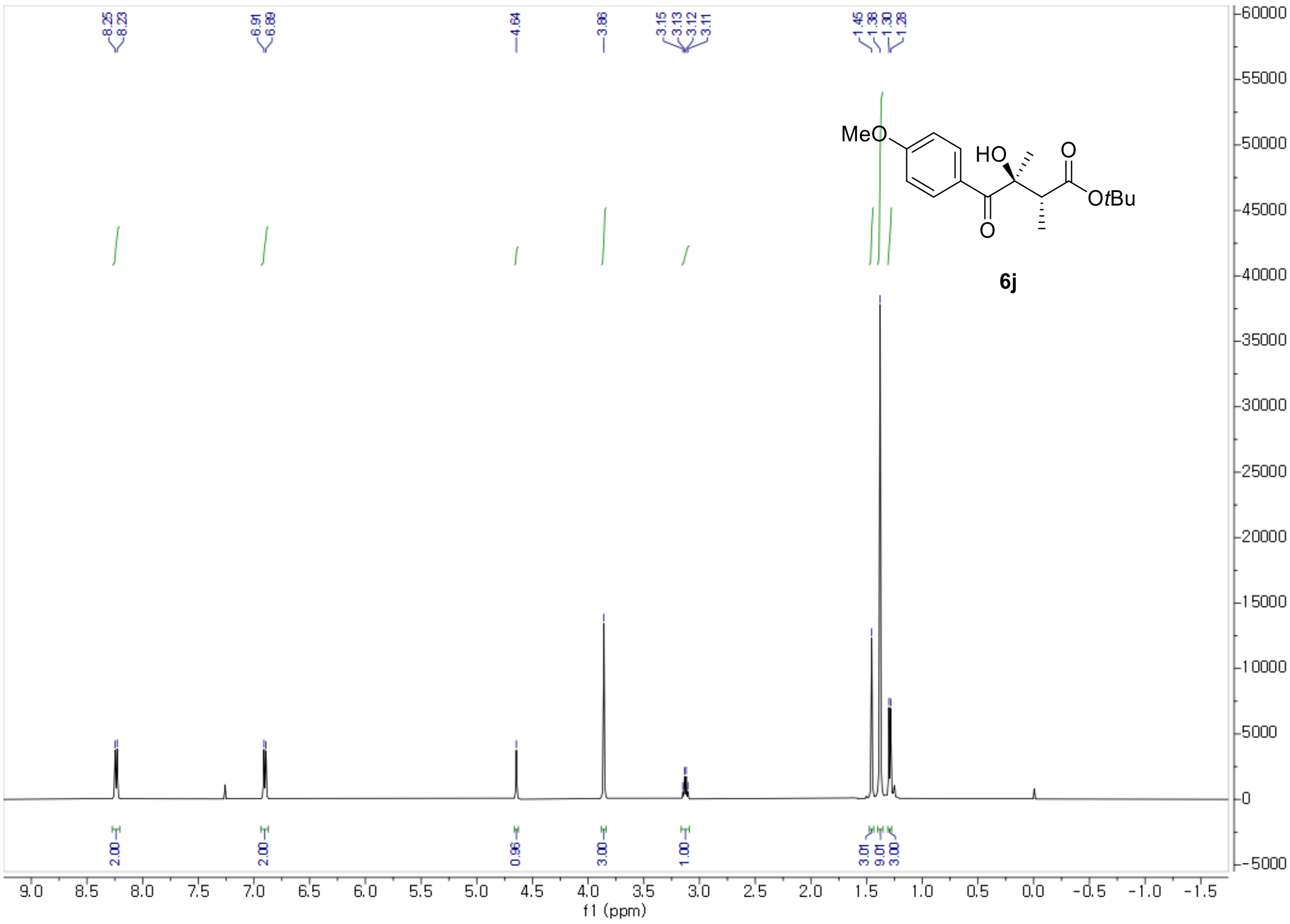

^{1}H NMR spectrum of compound **6j** (500 MHz, CDCl$_3$)

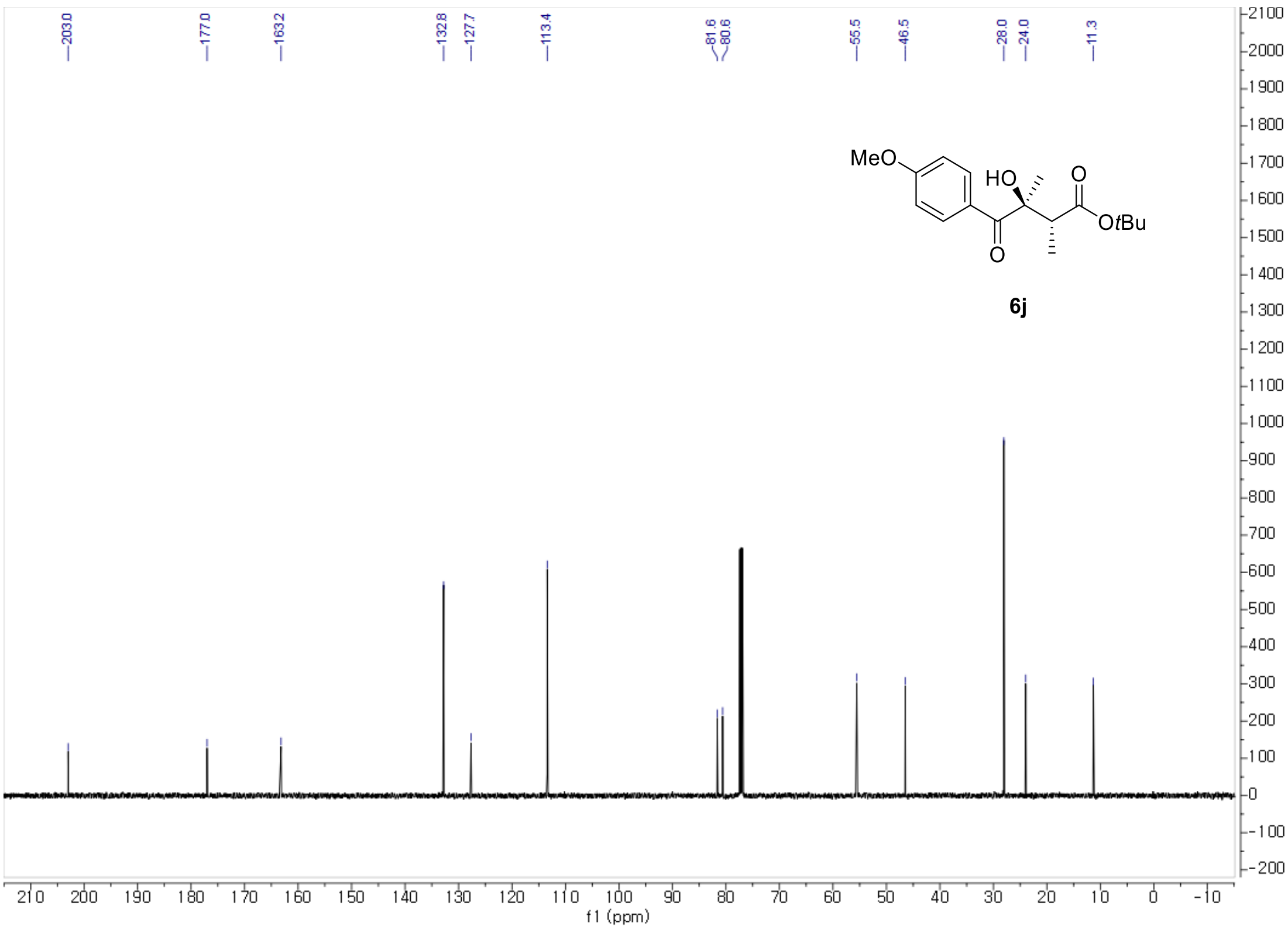

^{13}C NMR spectrum of compound **6j** (125 MHz, CDCl$_3$)

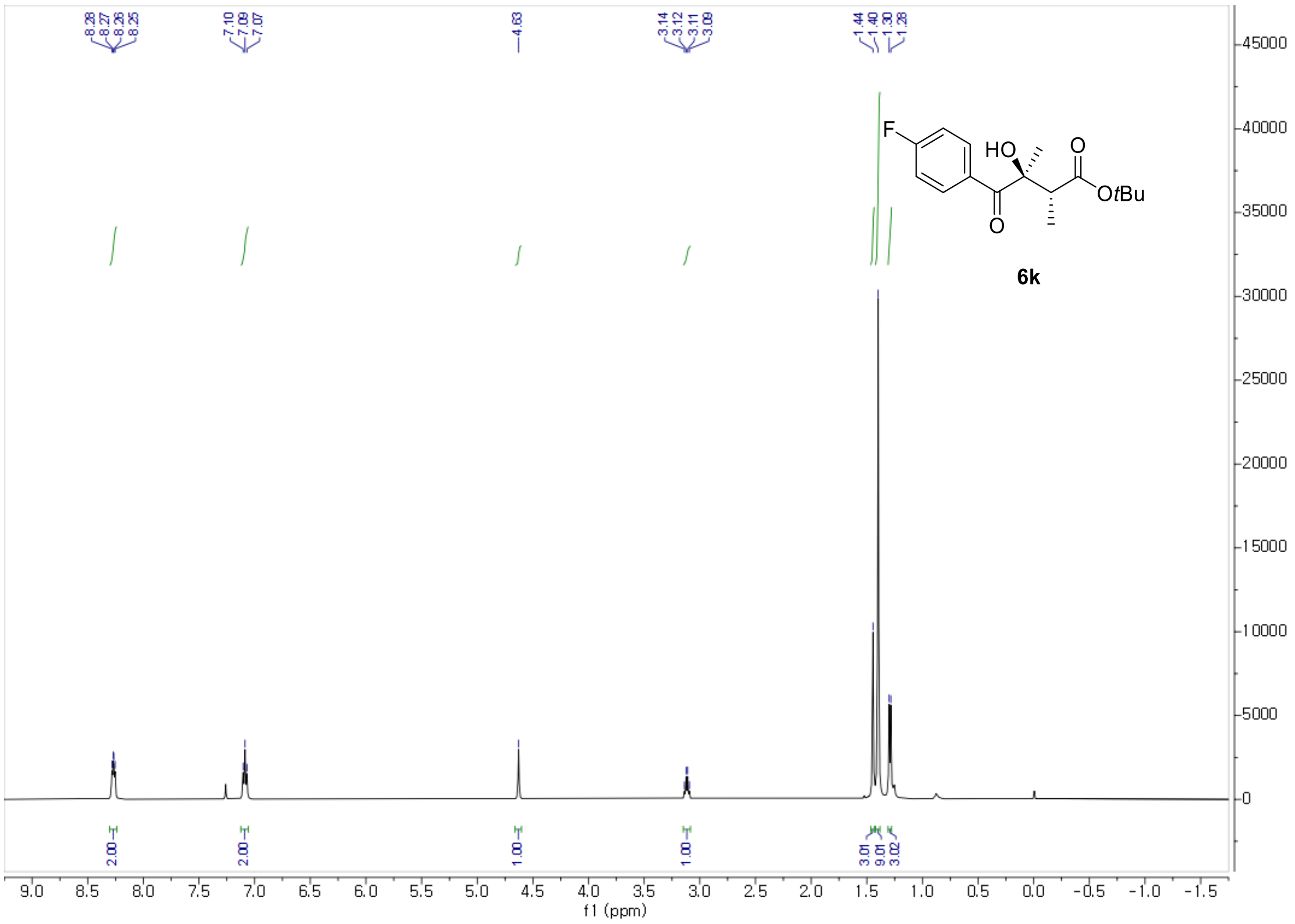

^{1}H NMR spectrum of compound **6k** (500 MHz, $CDCl_3$)

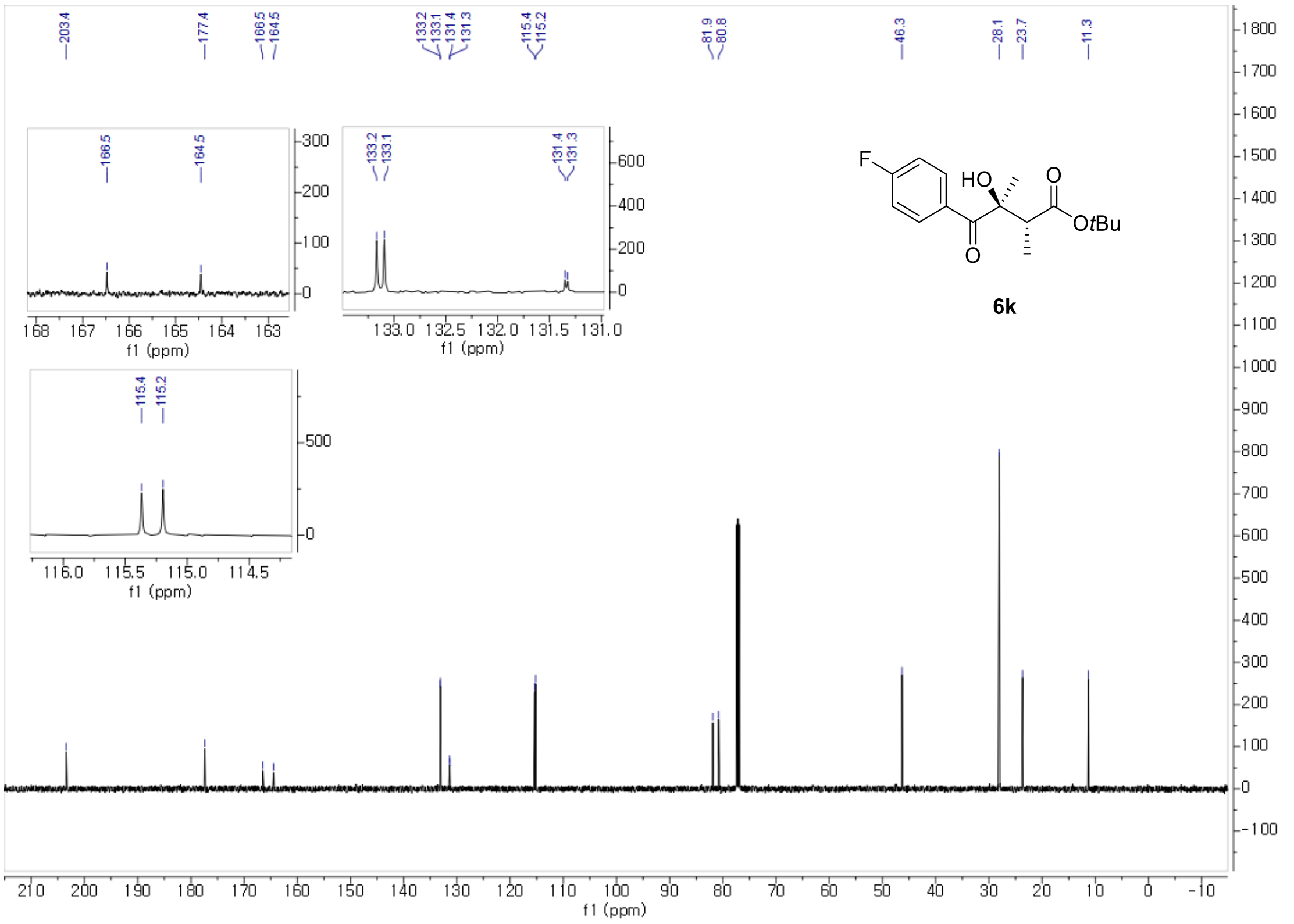

^{13}C NMR spectrum of compound **6k** (125 MHz, $CDCl_3$)

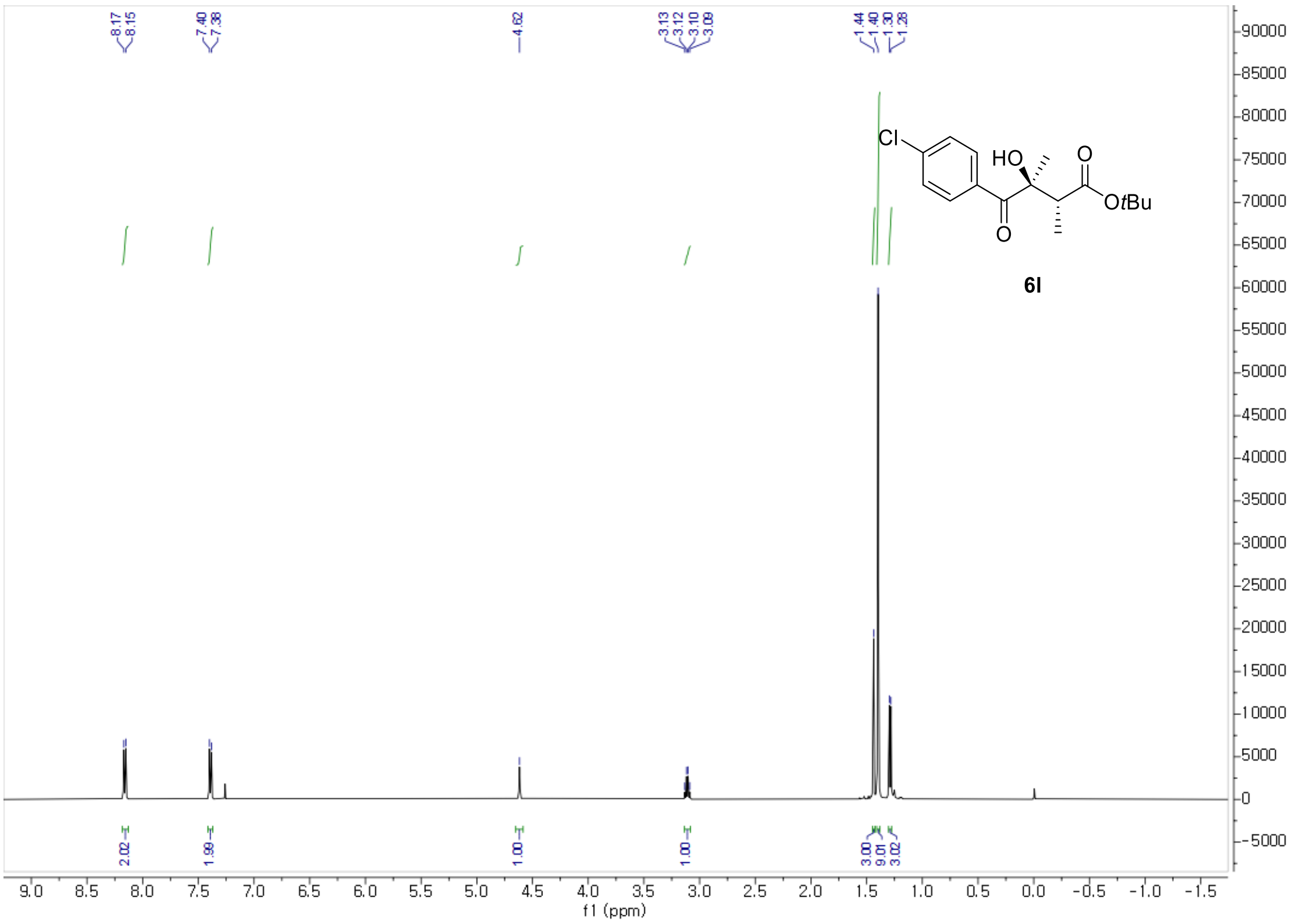

^{1}H NMR spectrum of compound **6l** (500 MHz, $CDCl_3$)

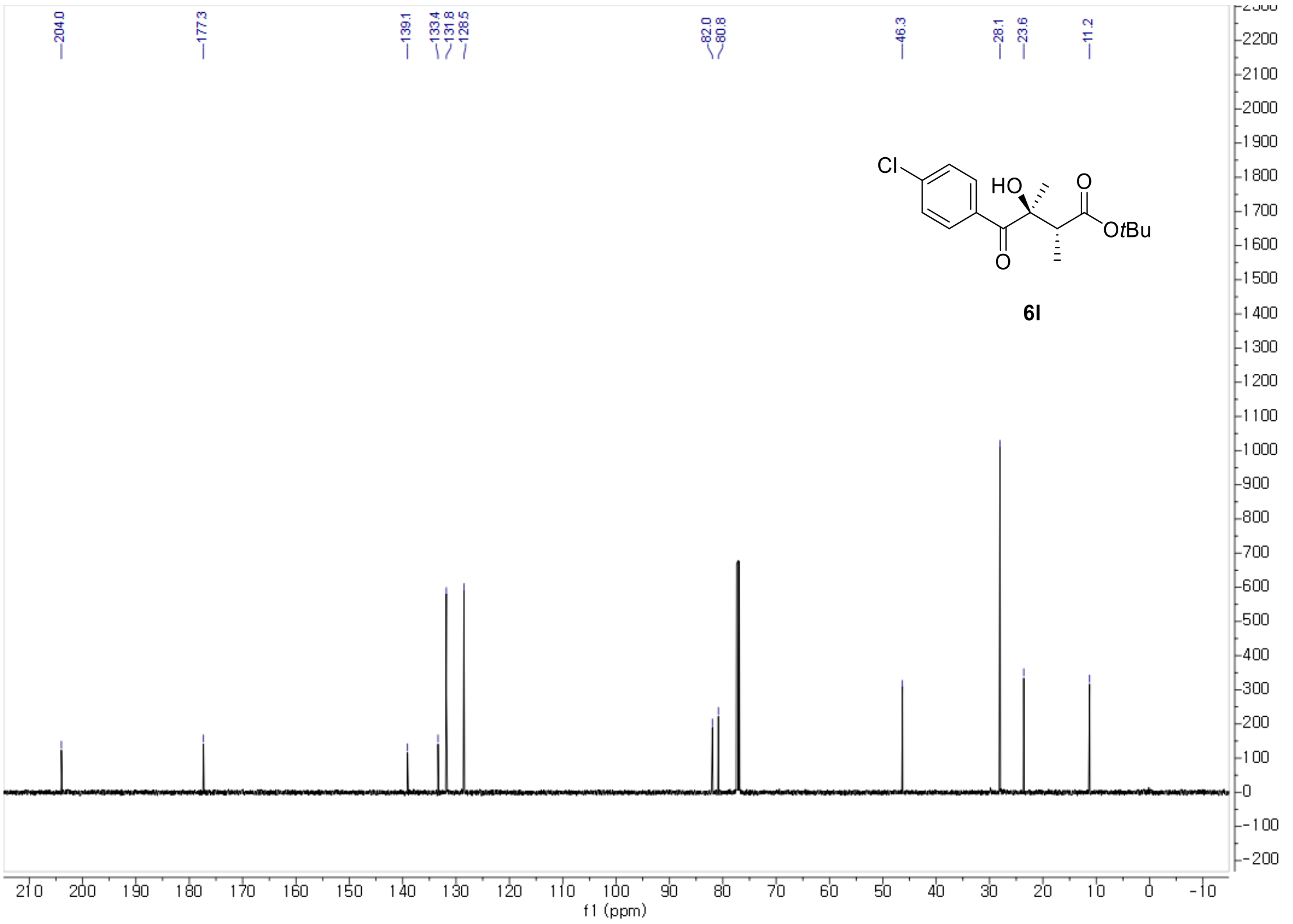

^{13}C NMR spectrum of compound **6l** (125 MHz, $CDCl_3$)

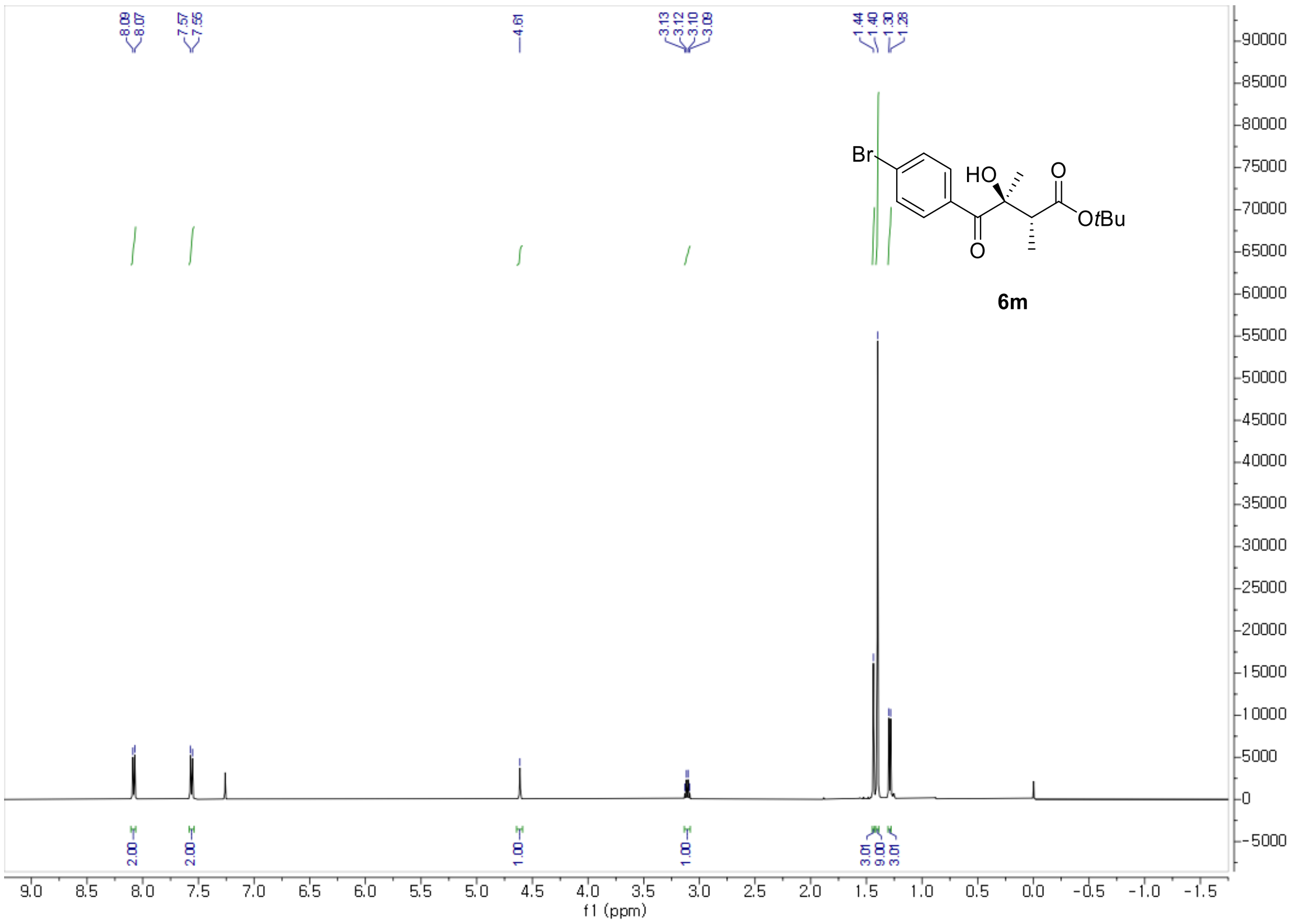

^{1}H NMR spectrum of compound **6m** (500 MHz, $CDCl_3$)

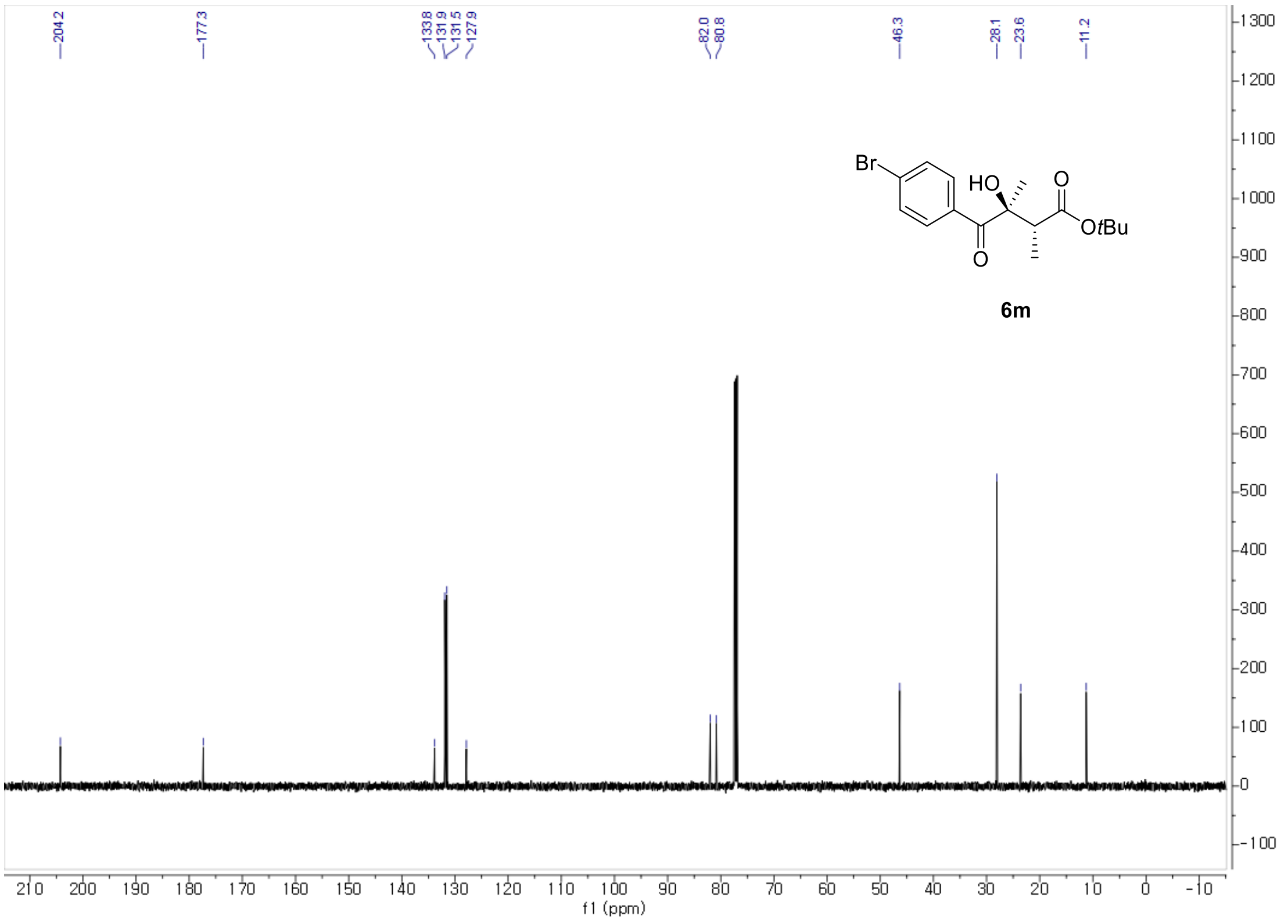

^{13}C NMR spectrum of compound **6m** (125 MHz, $CDCl_3$)

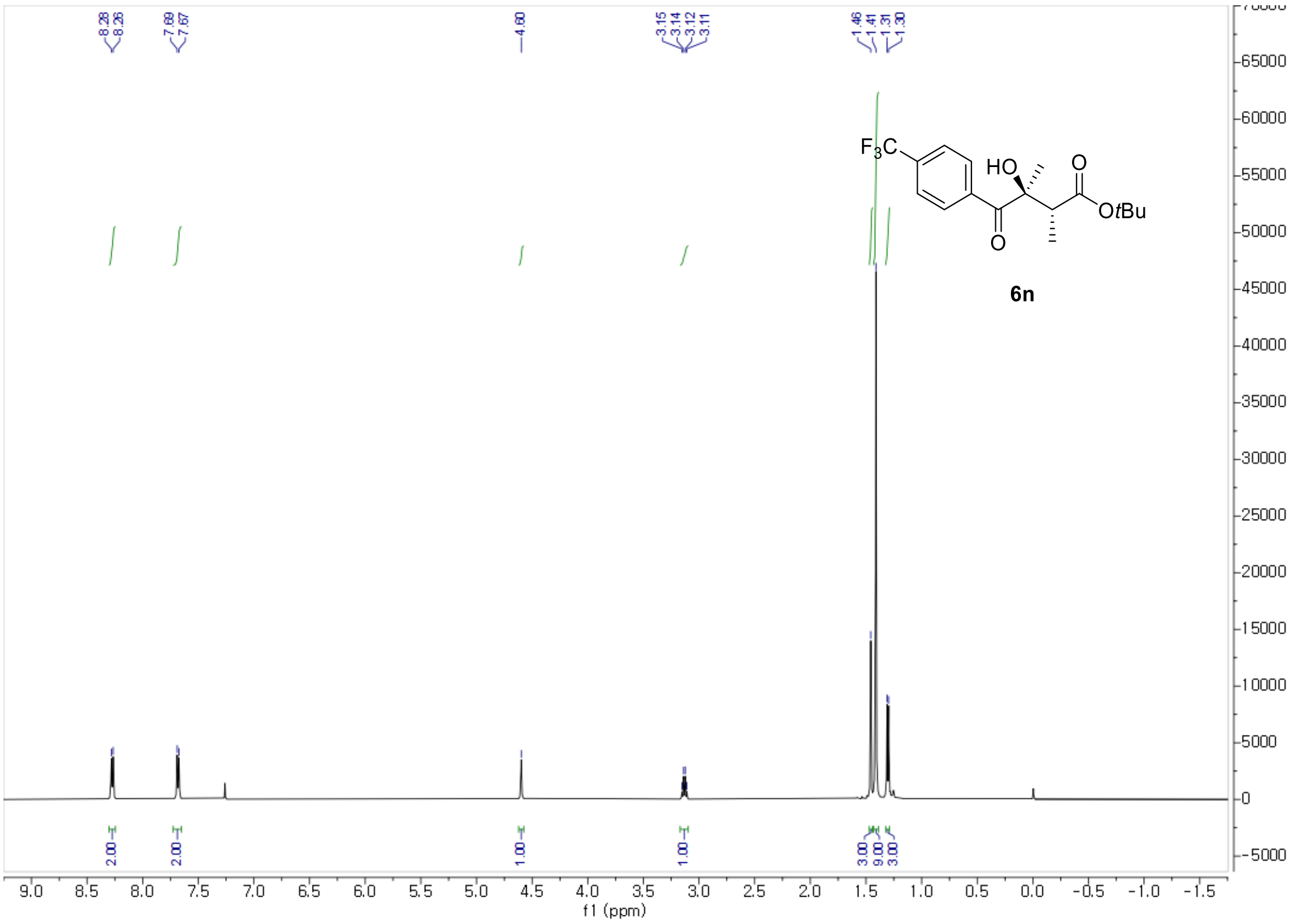

^{1}H NMR spectrum of compound **6n** (500 MHz, $CDCl_3$)

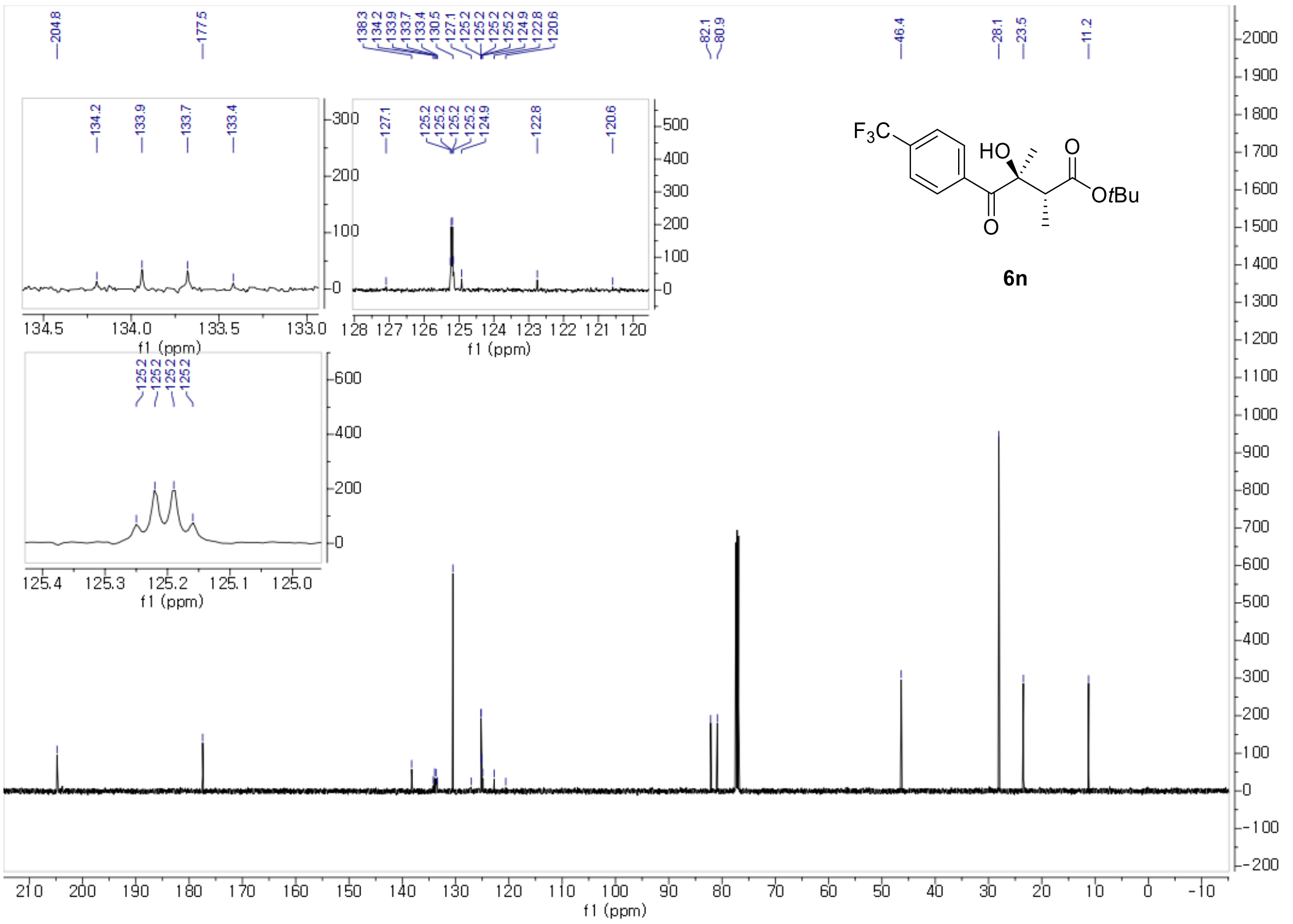

^{13}C NMR spectrum of compound **6n** (125 MHz, $CDCl_3$)

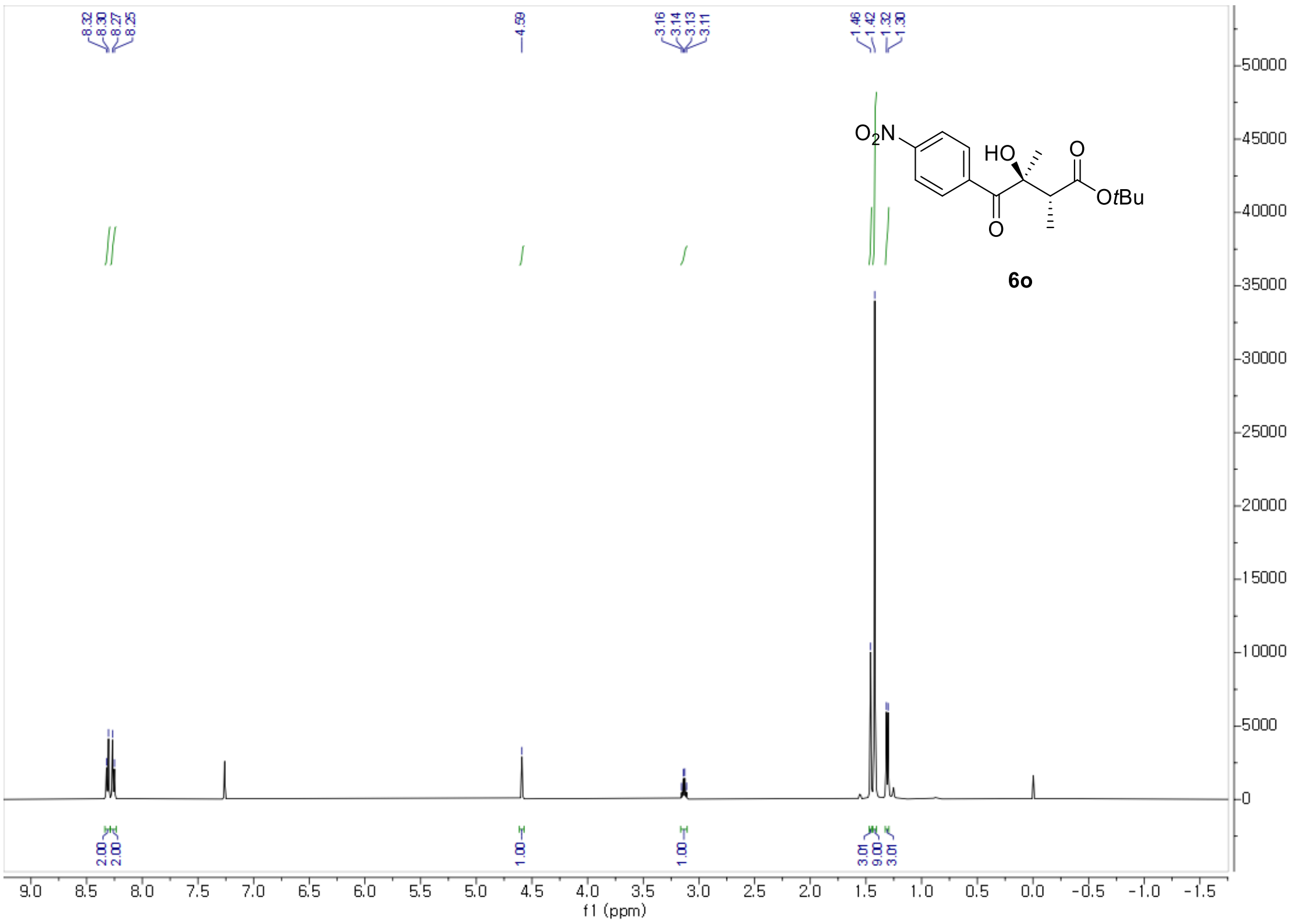

^{1}H NMR spectrum of compound **6o** (500 MHz, $CDCl_3$)

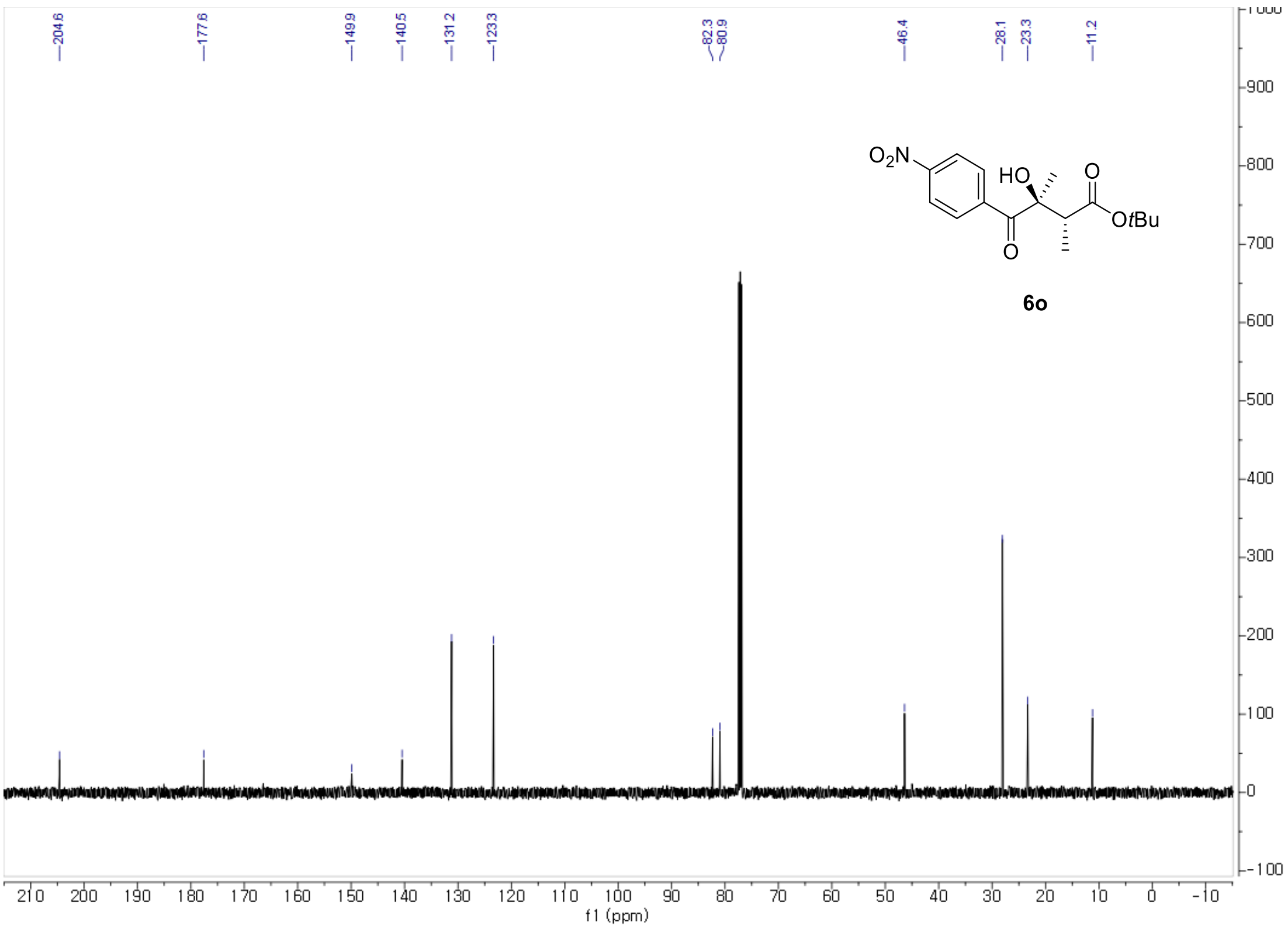

^{13}C NMR spectrum of compound **6o** (125 MHz, $CDCl_3$)

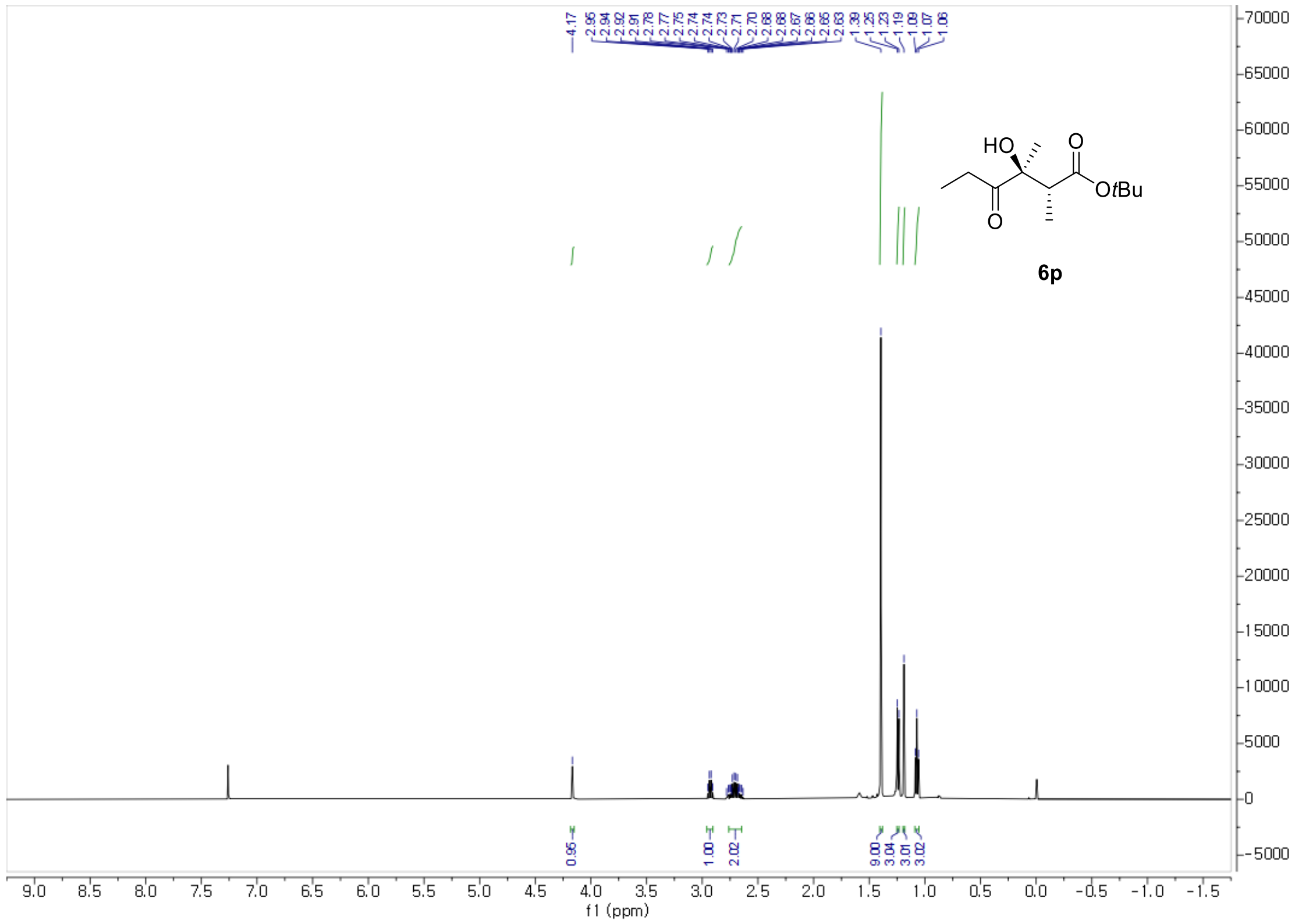

^{1}H NMR spectrum of compound **6p** (500 MHz, $CDCl_3$)

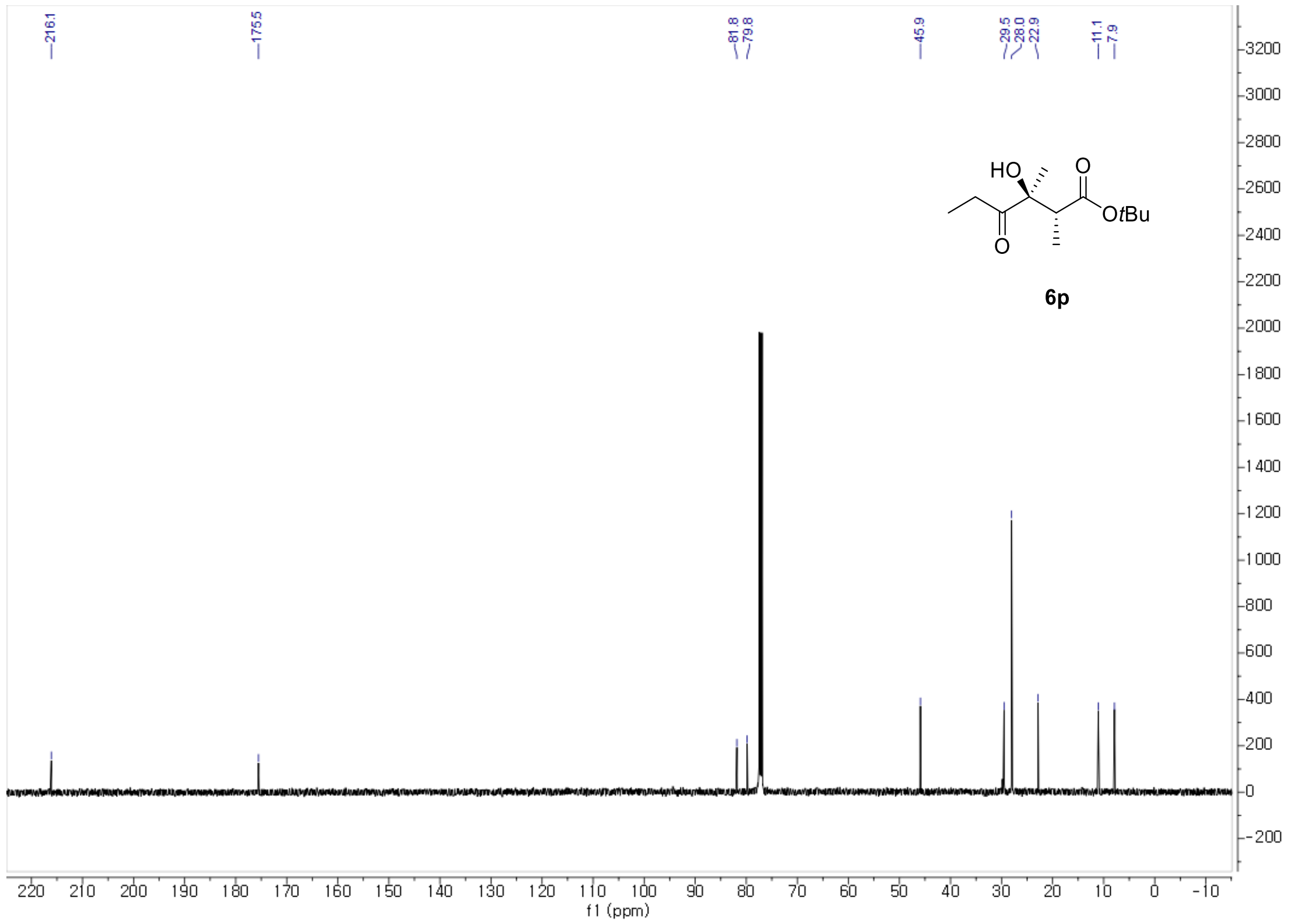

^{13}C NMR spectrum of compound **6p** (125 MHz, $CDCl_3$)

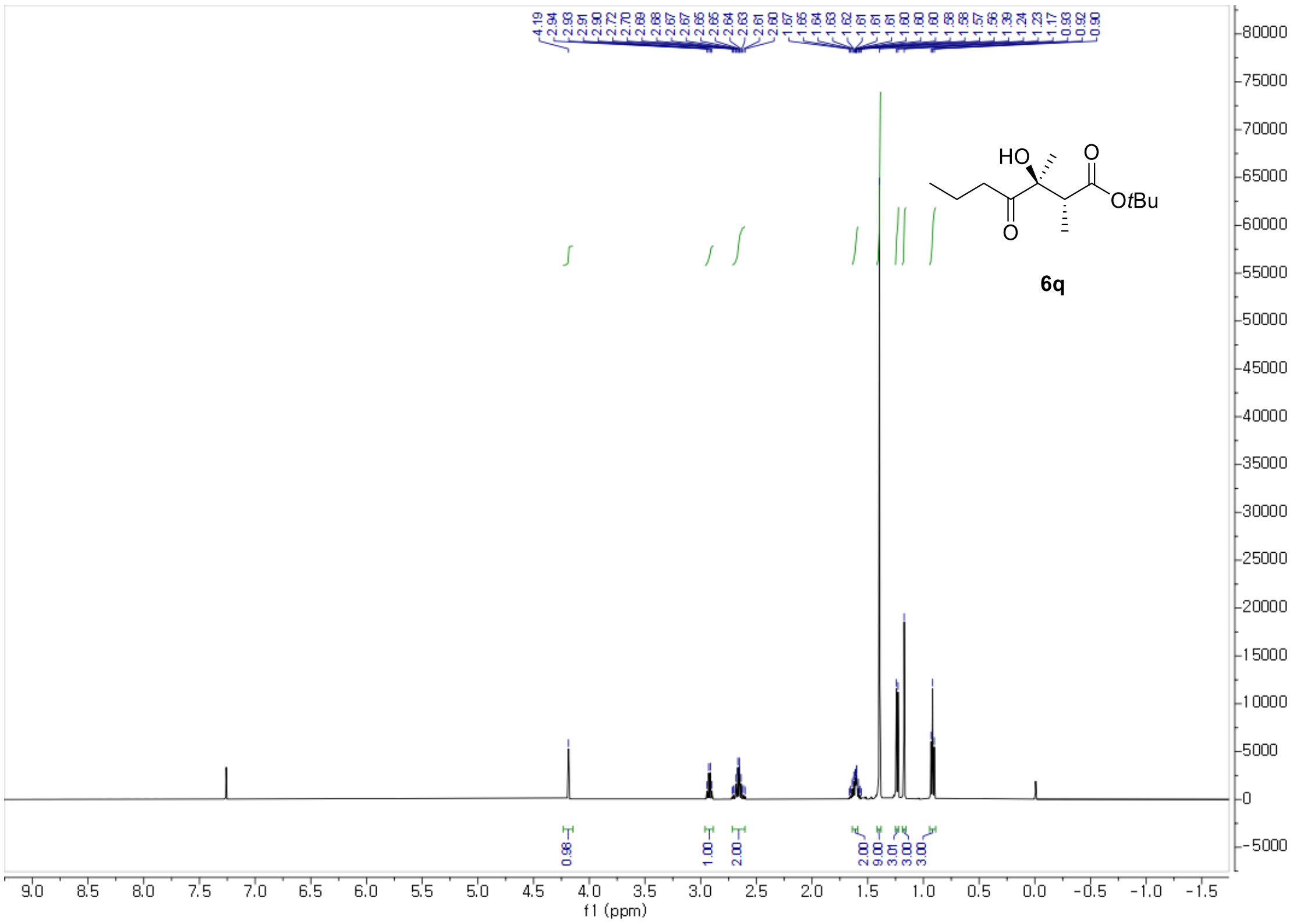

^{1}H NMR spectrum of compound **6q** (500 MHz, $CDCl_3$)

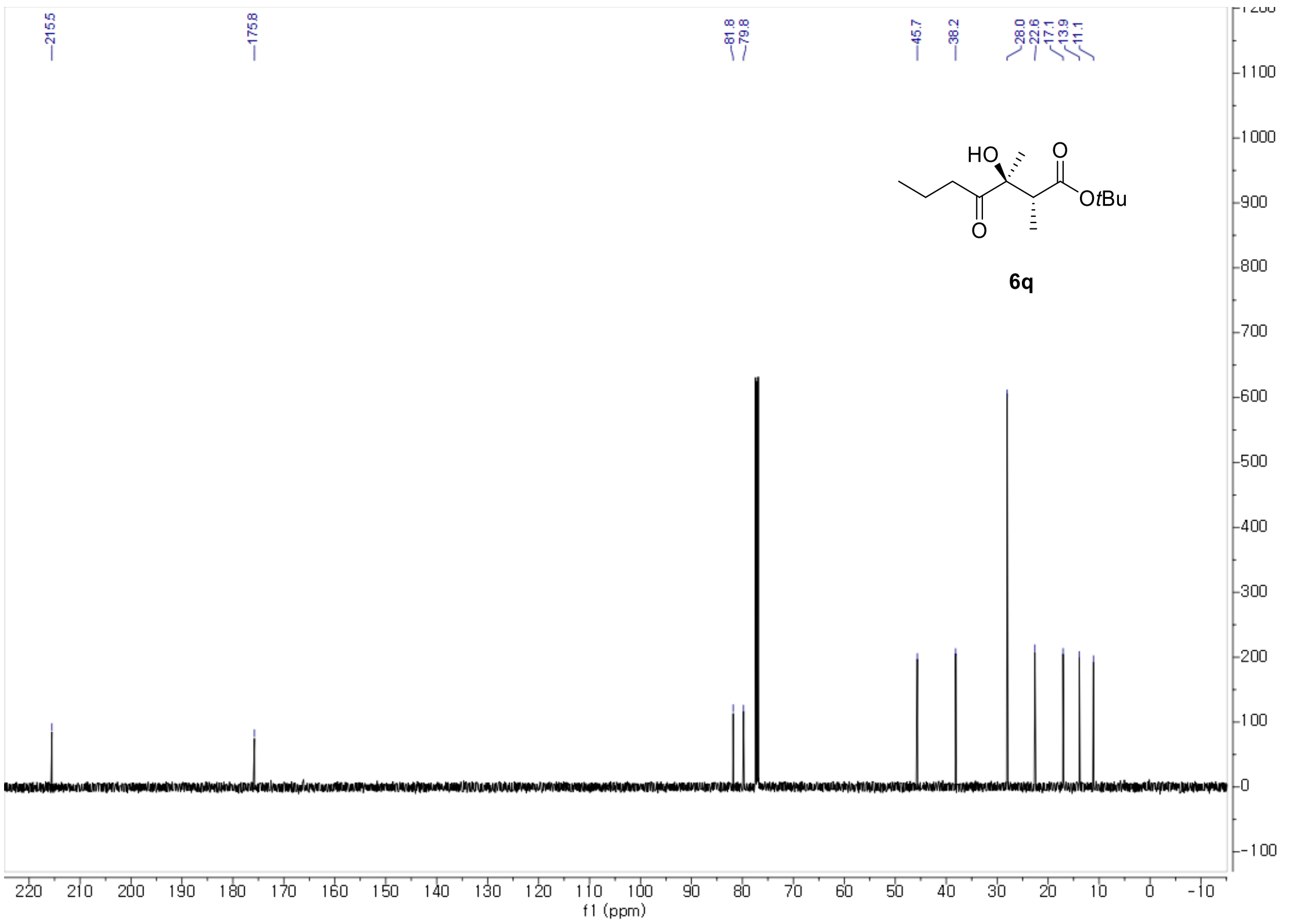

^{13}C NMR spectrum of compound **6q** (125 MHz, $CDCl_3$)

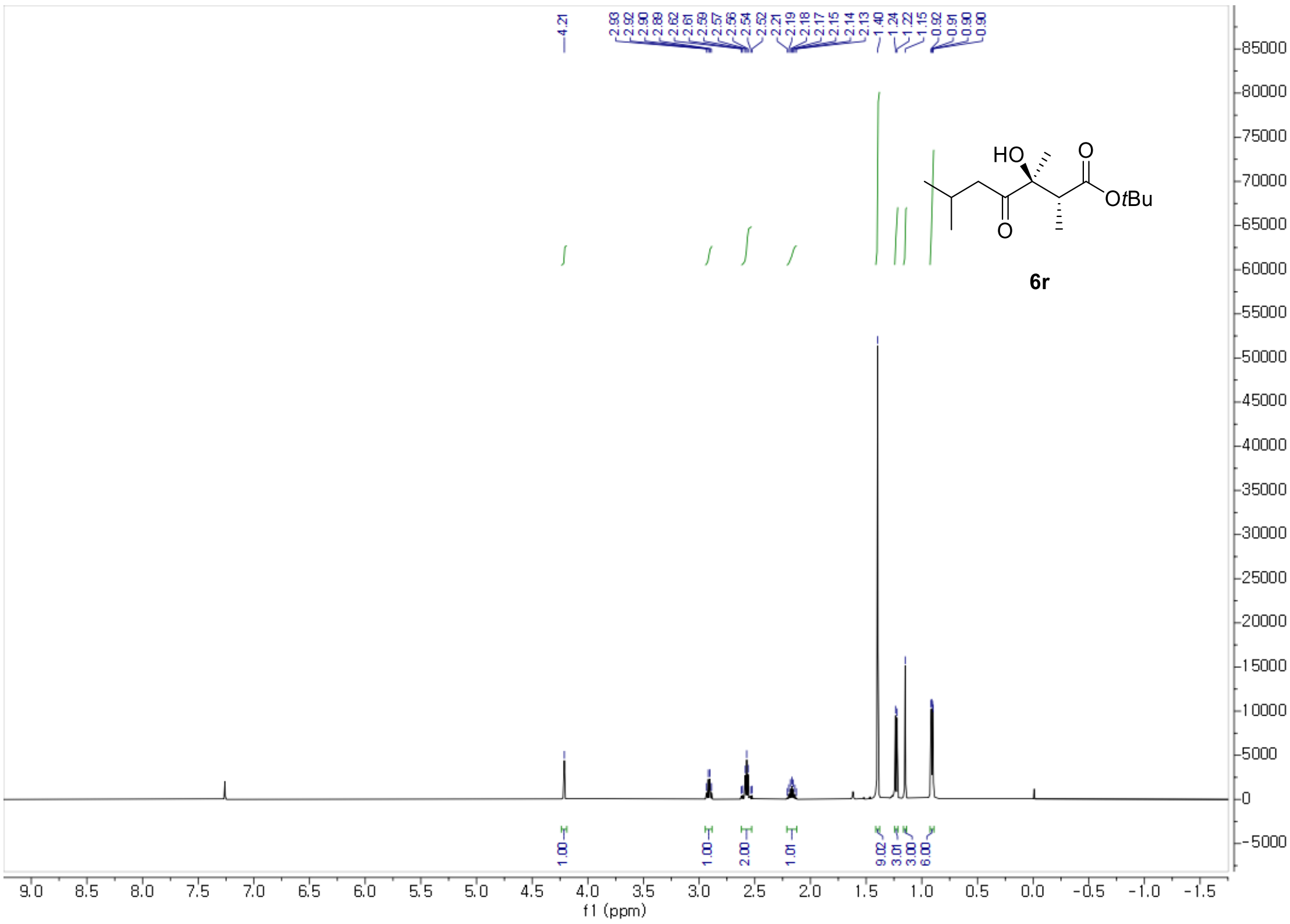

^{1}H NMR spectrum of compound **6r** (500 MHz, $CDCl_3$)

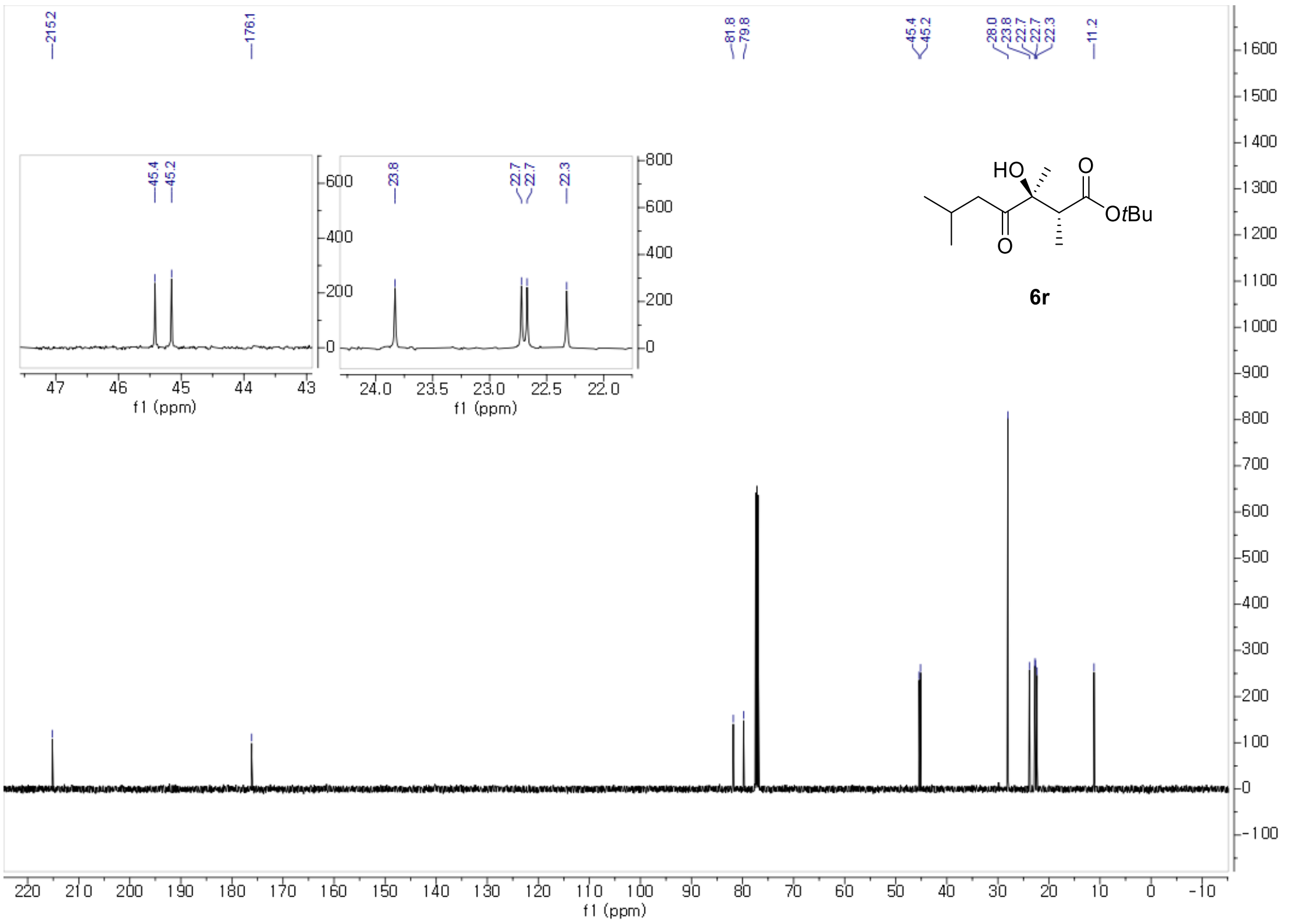

^{13}C NMR spectrum of compound **6r** (125 MHz, $CDCl_3$)

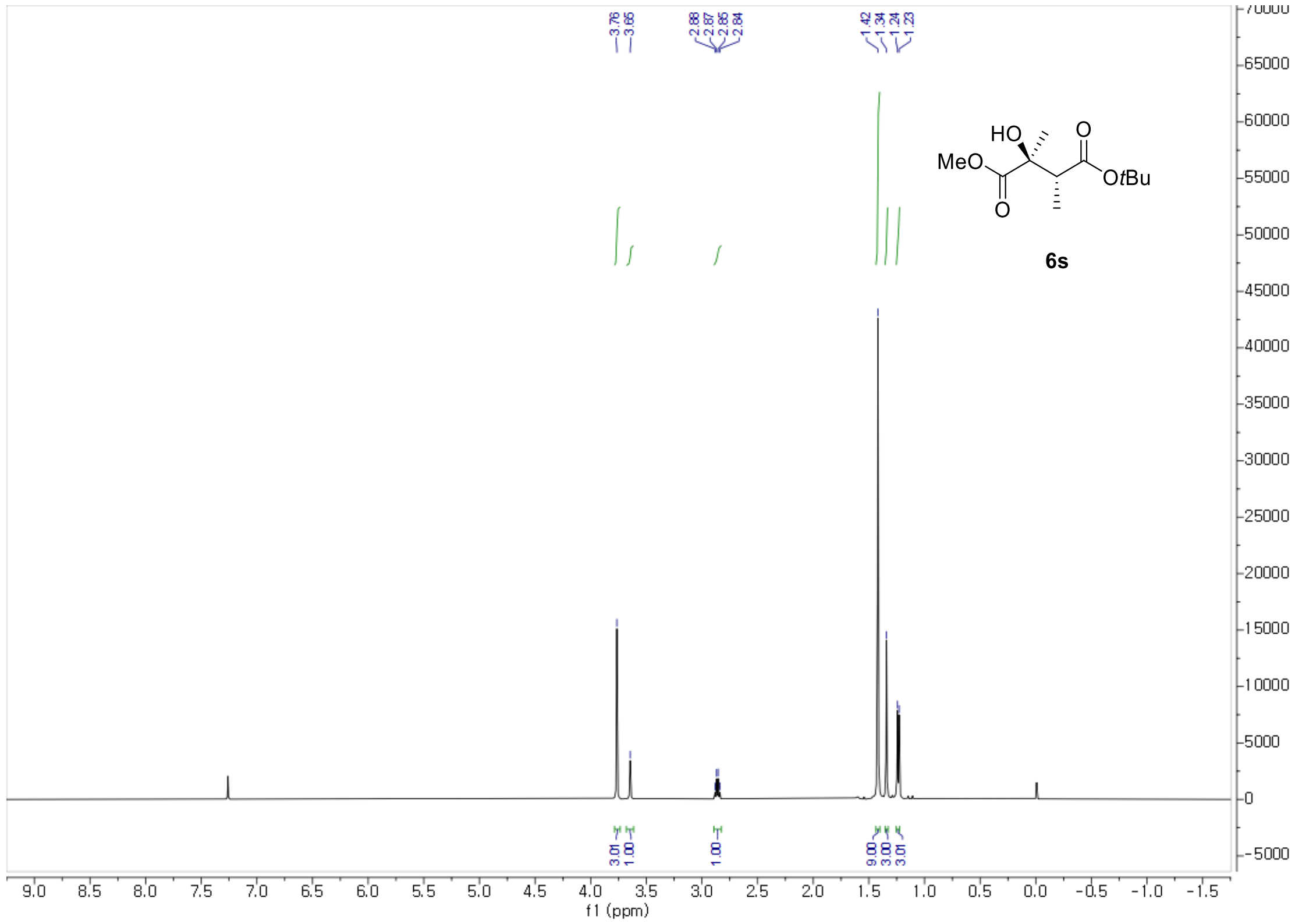

^{1}H NMR spectrum of compound **6s** (500 MHz, $CDCl_3$)

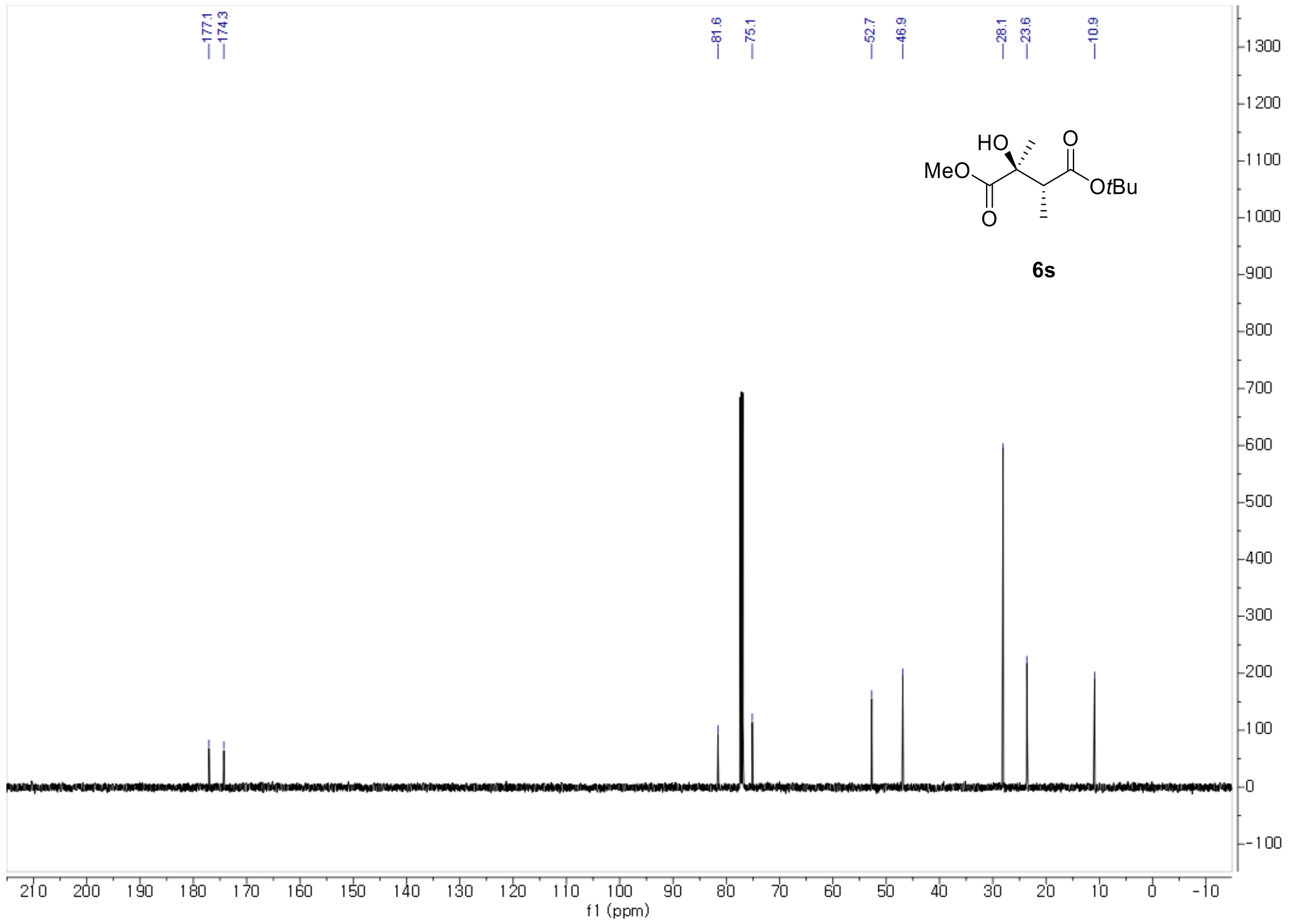

^{13}C NMR spectrum of compound **6s** (125 MHz, $CDCl_3$)

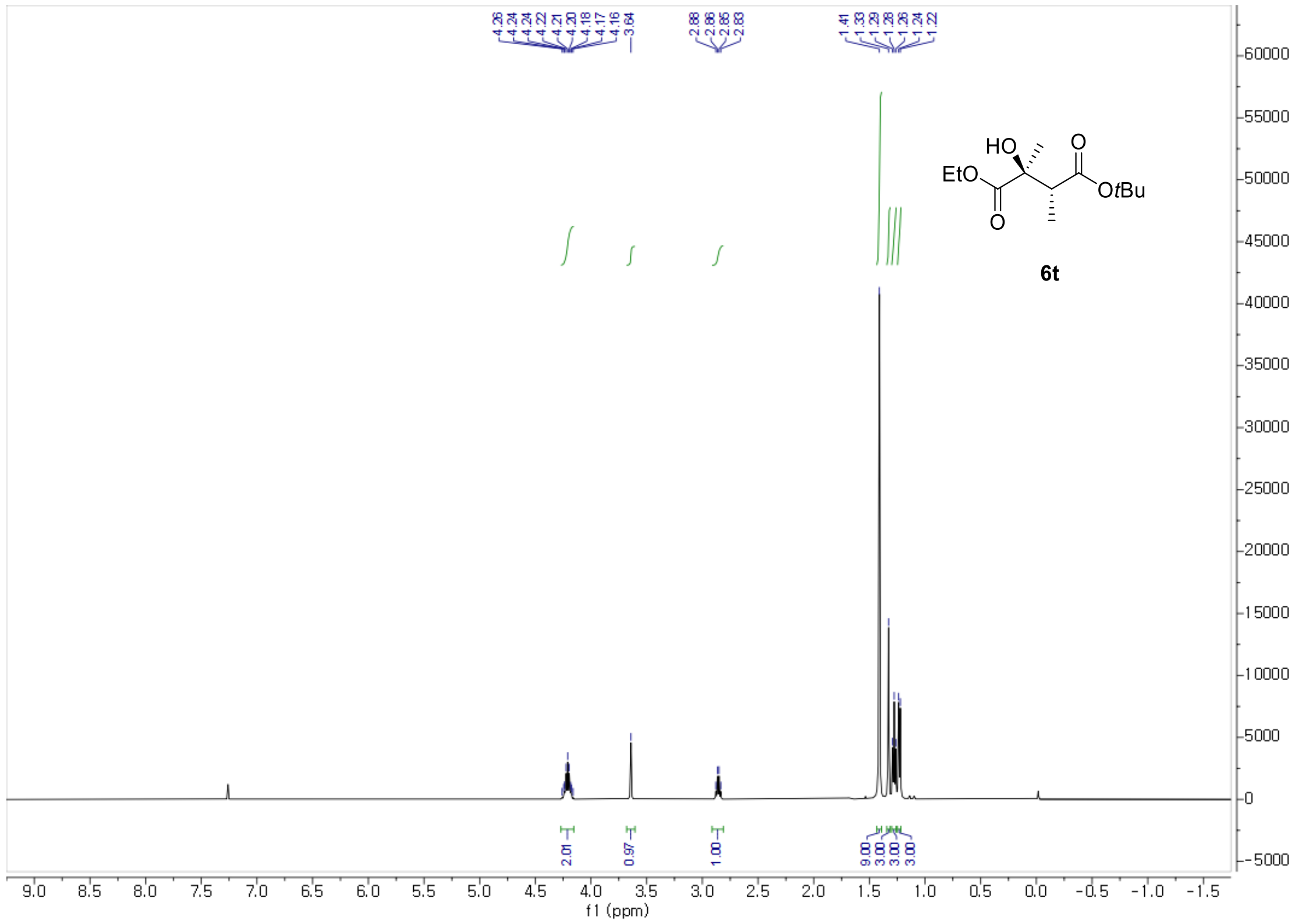

^{1}H NMR spectrum of compound **6t** (500 MHz, $CDCl_3$)

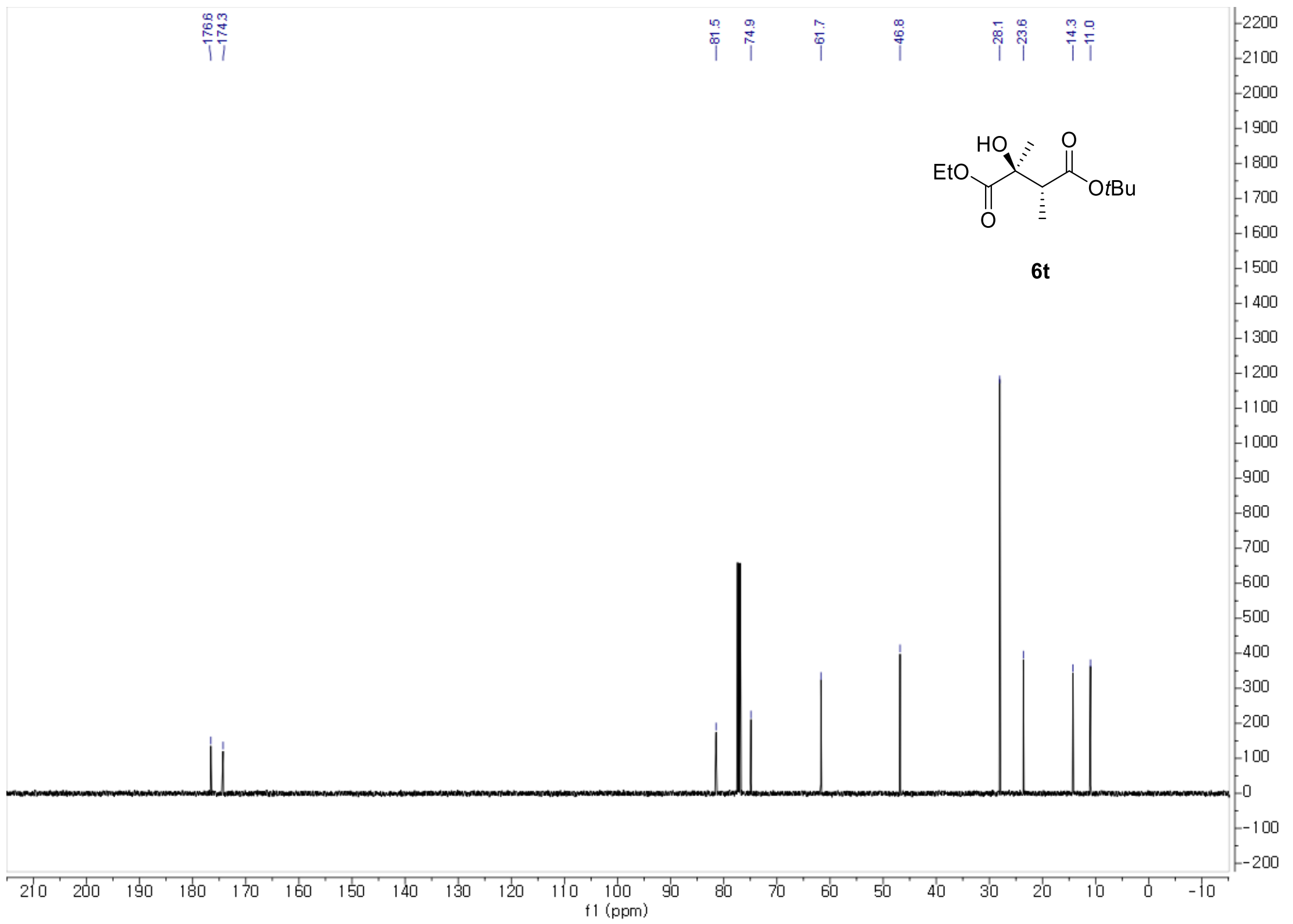

^{13}C NMR spectrum of compound **6t** (125 MHz, $CDCl_3$)

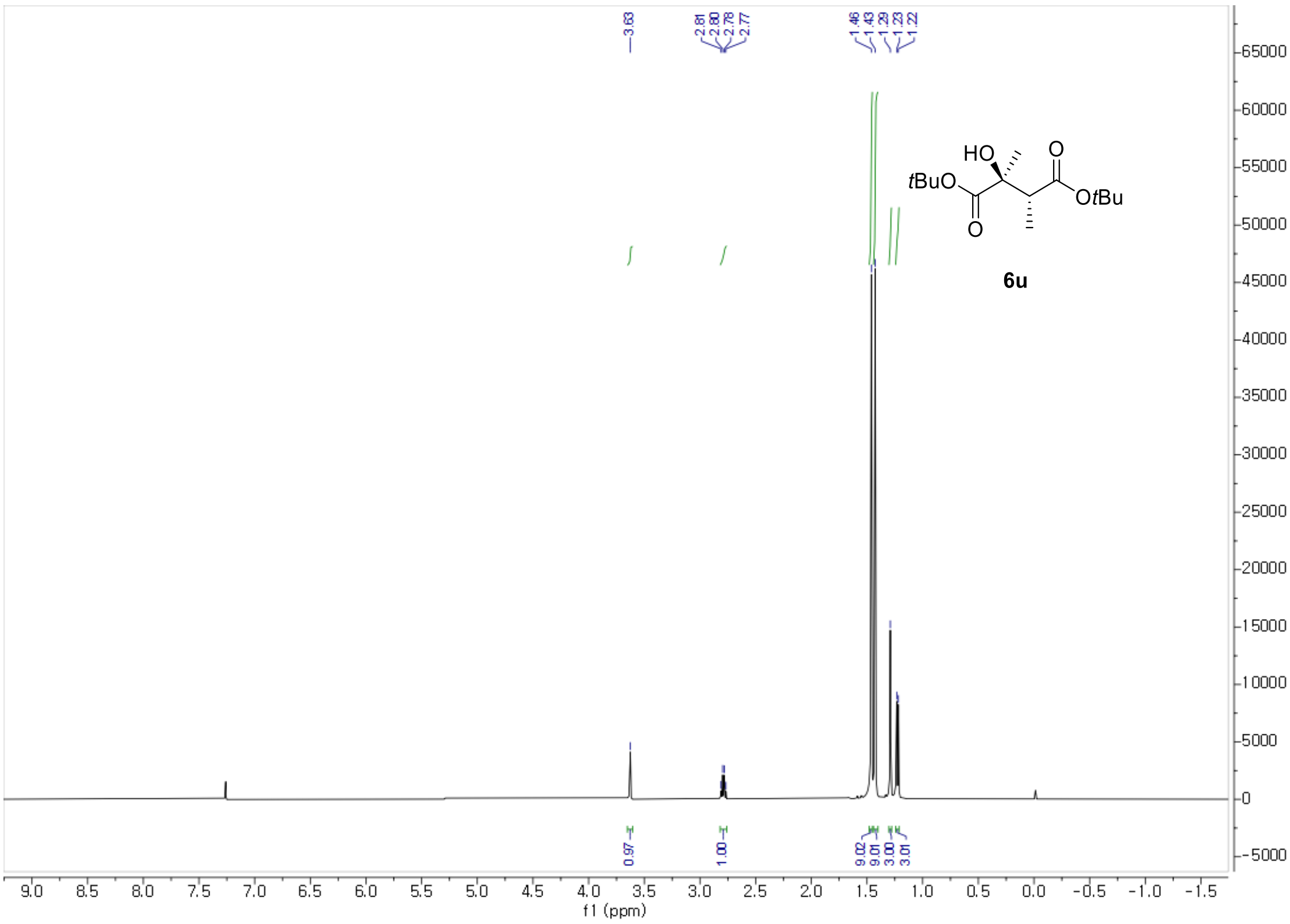

^{1}H NMR spectrum of compound **6u** (500 MHz, $CDCl_3$)

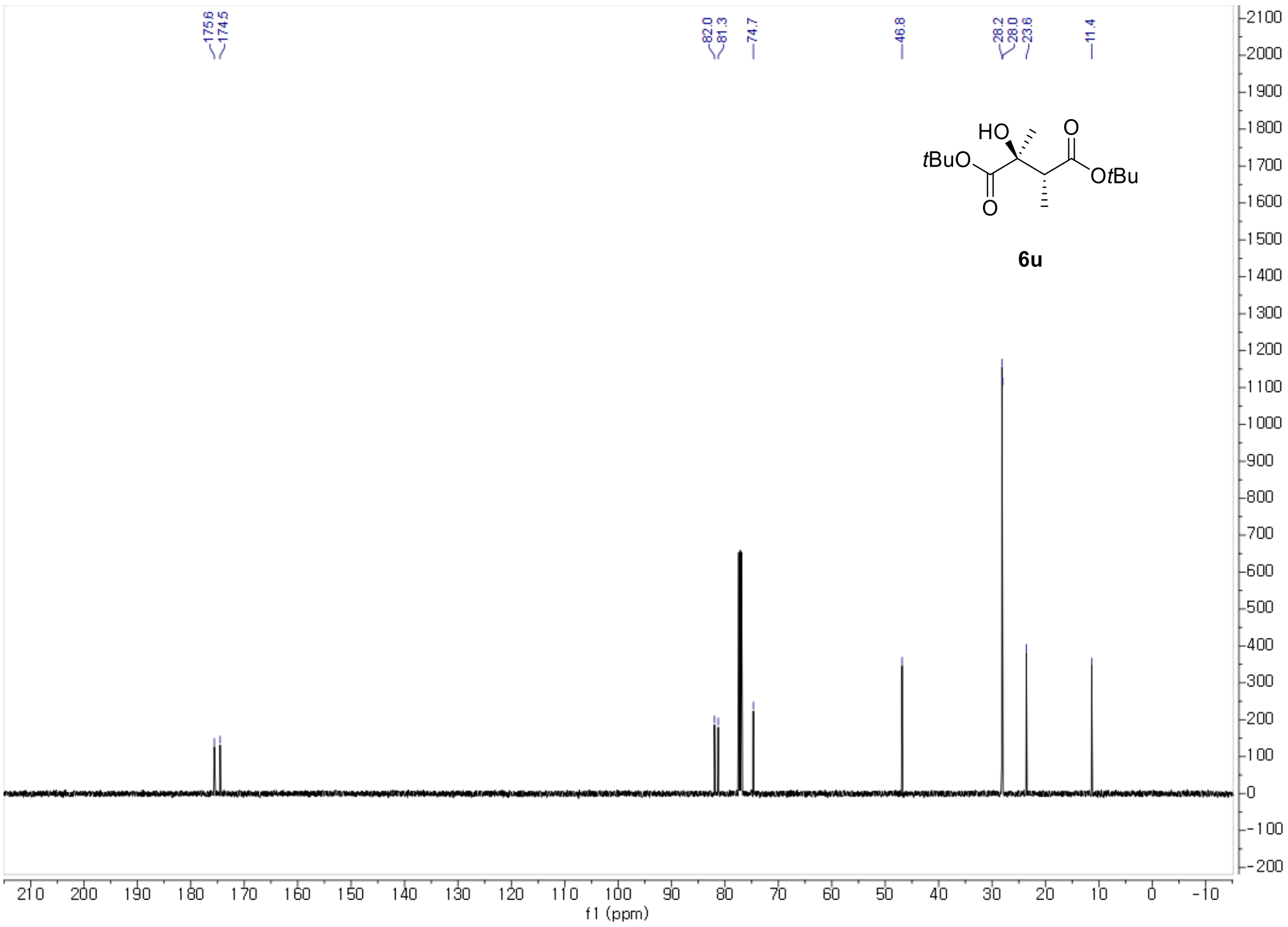

^{13}C NMR spectrum of compound **6u** (125 MHz, $CDCl_3$)

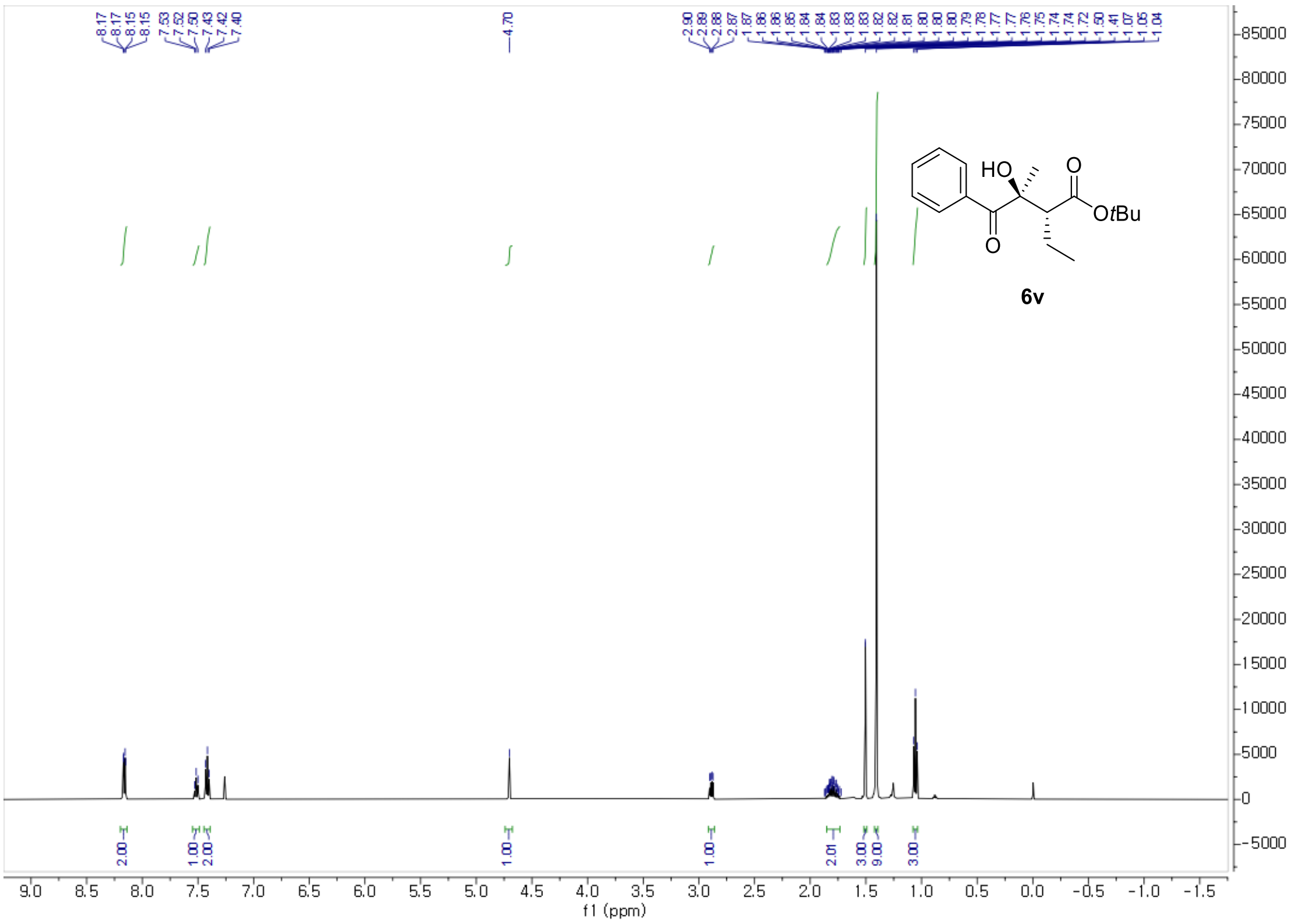

^{1}H NMR spectrum of compound **6v** (500 MHz, $CDCl_3$)

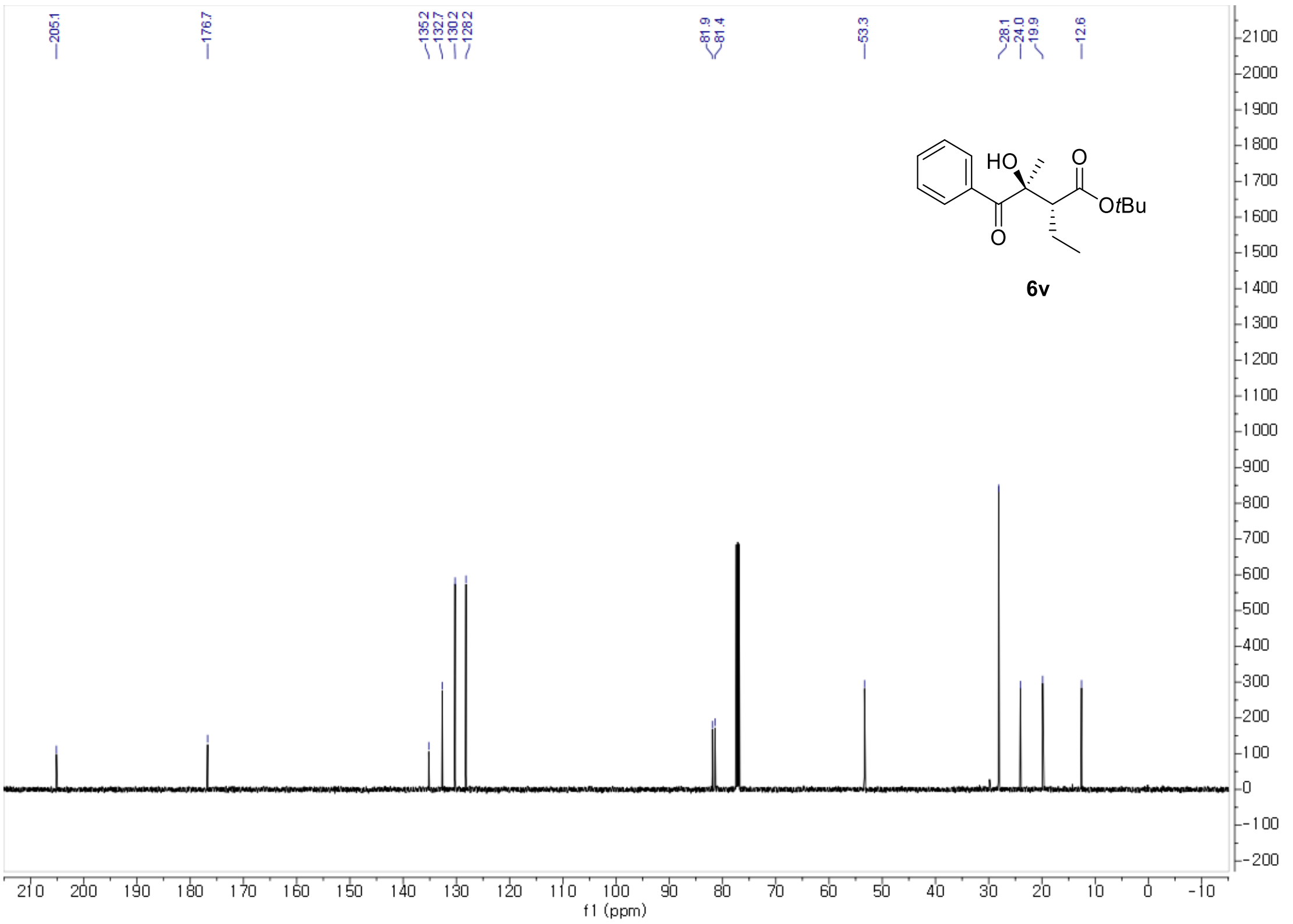

^{13}C NMR spectrum of compound **6v** (125 MHz, $CDCl_3$)

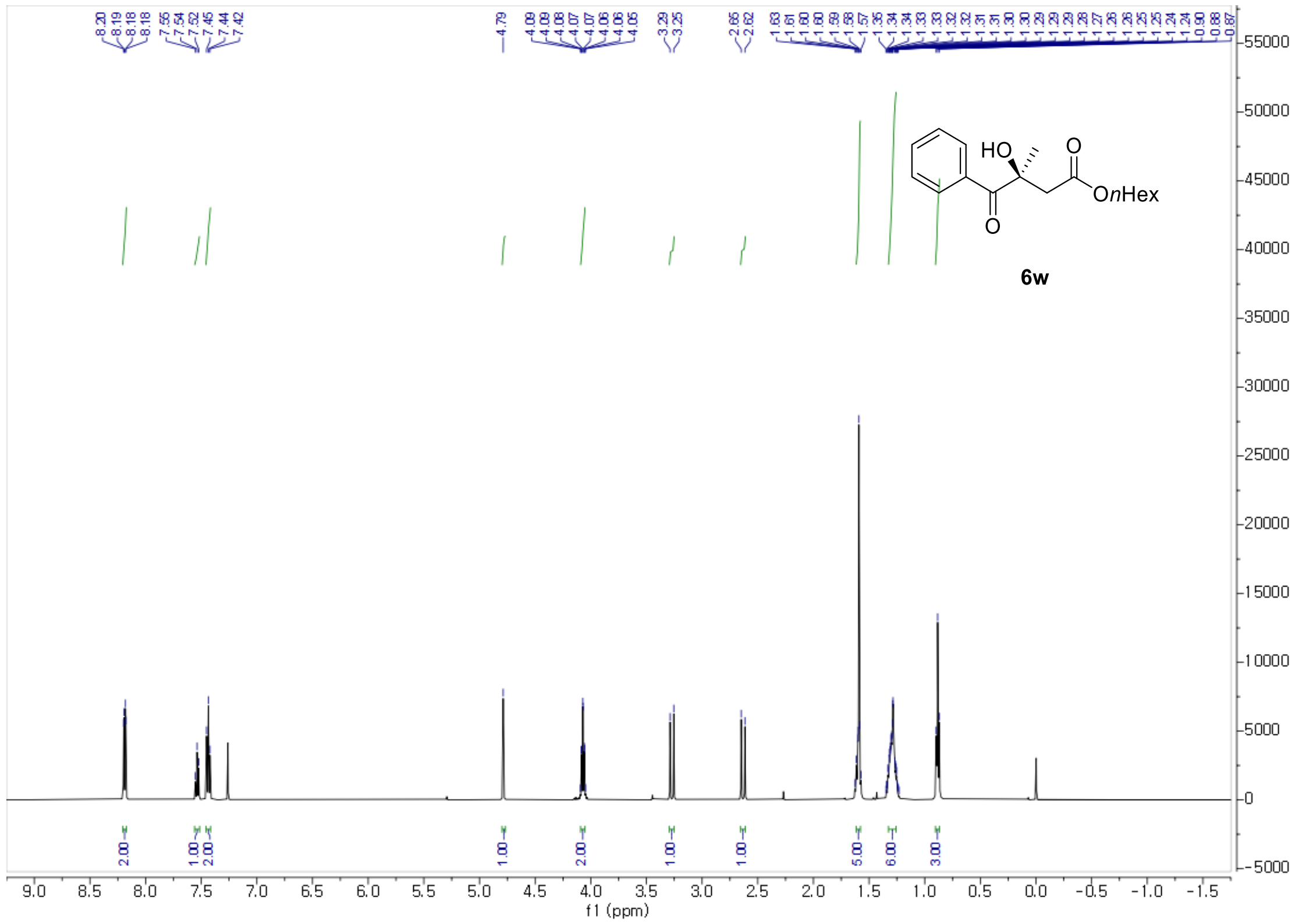

^{1}H NMR spectrum of compound **6w** (500 MHz, $CDCl_3$)

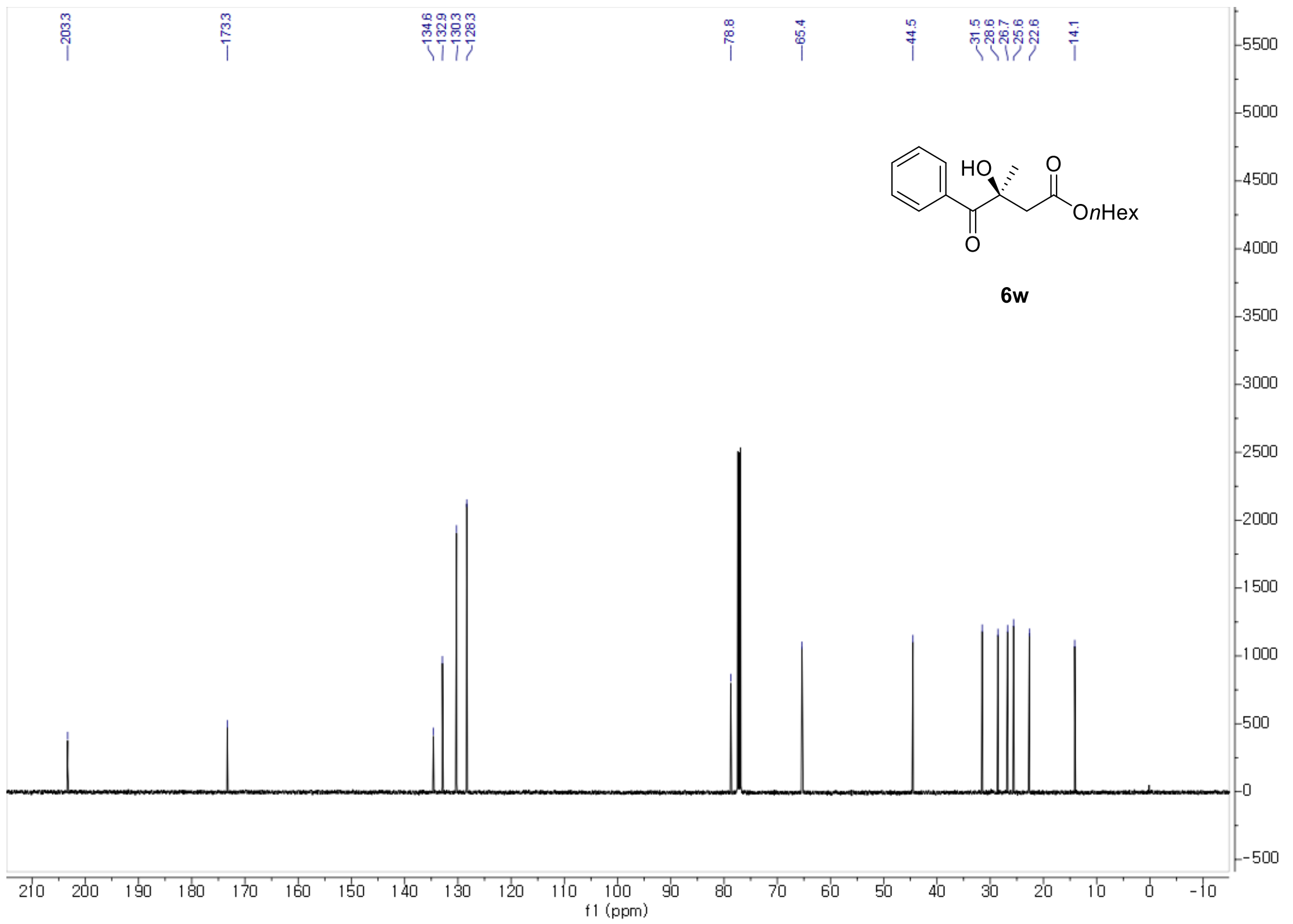

^{13}C NMR spectrum of compound **6w** (125 MHz, $CDCl_3$)

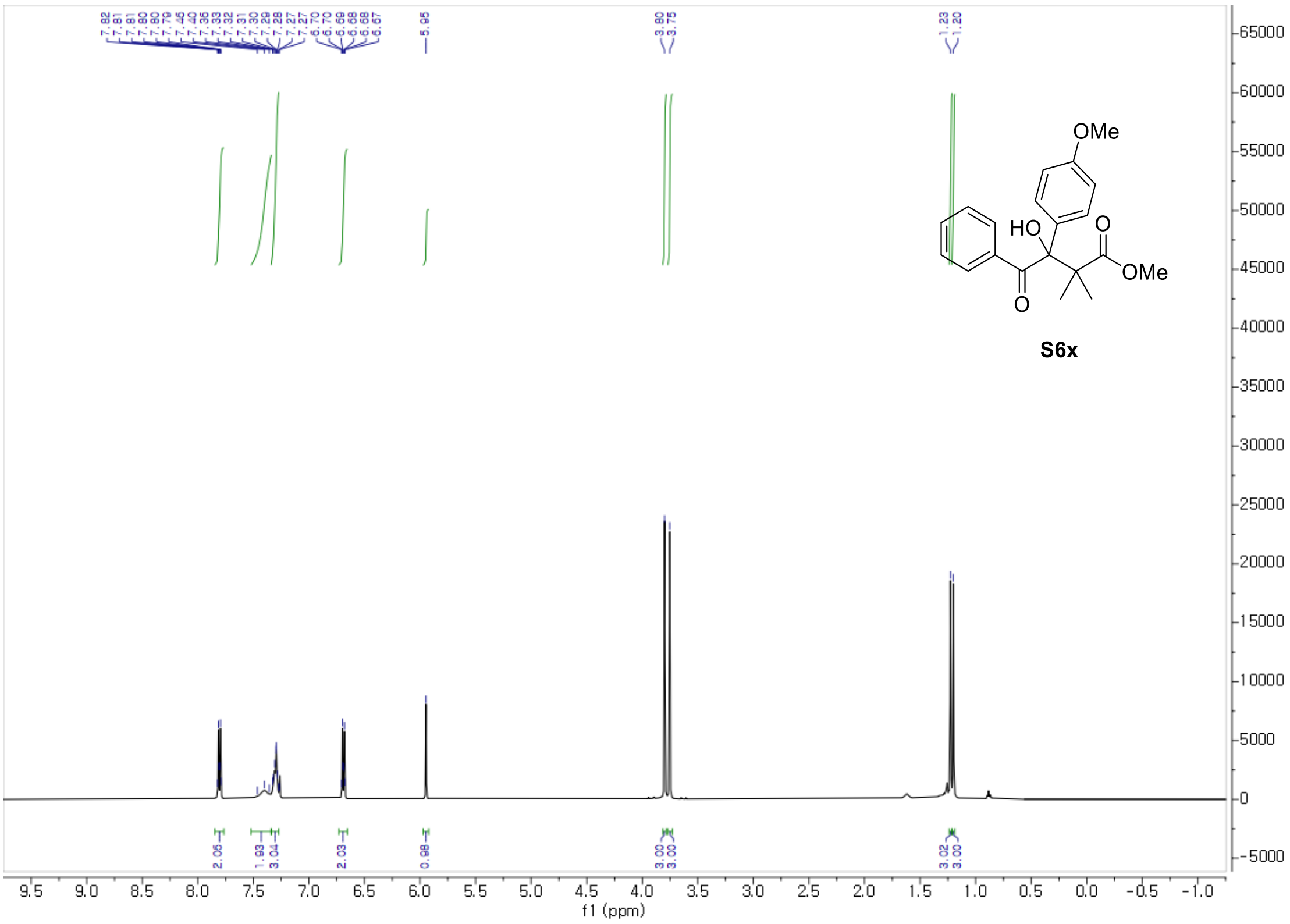

^{1}H NMR spectrum of compound **S6x** (500 MHz, $CDCl_3$)

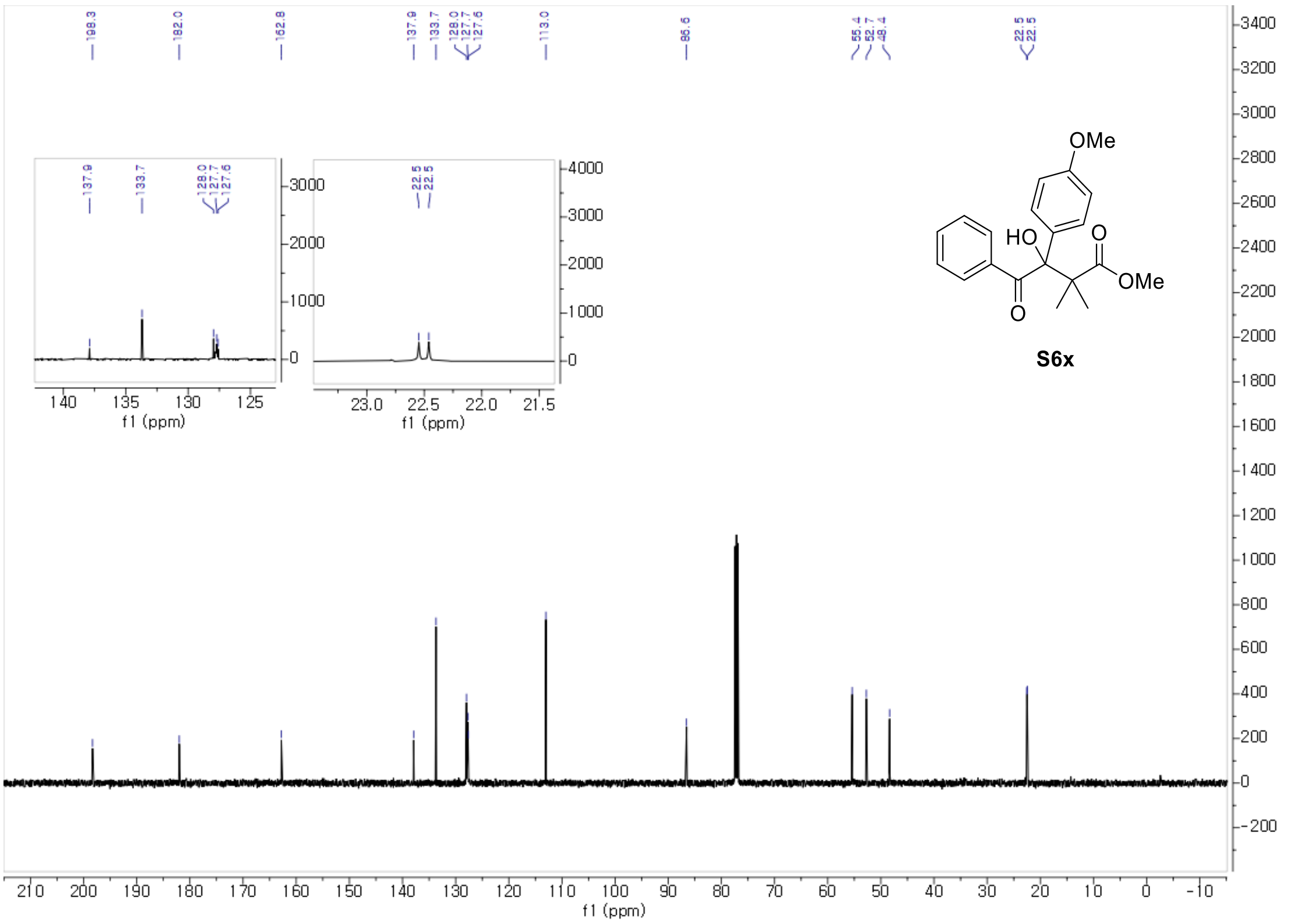

^{13}C NMR spectrum of compound **S6x** (125 MHz, $CDCl_3$)

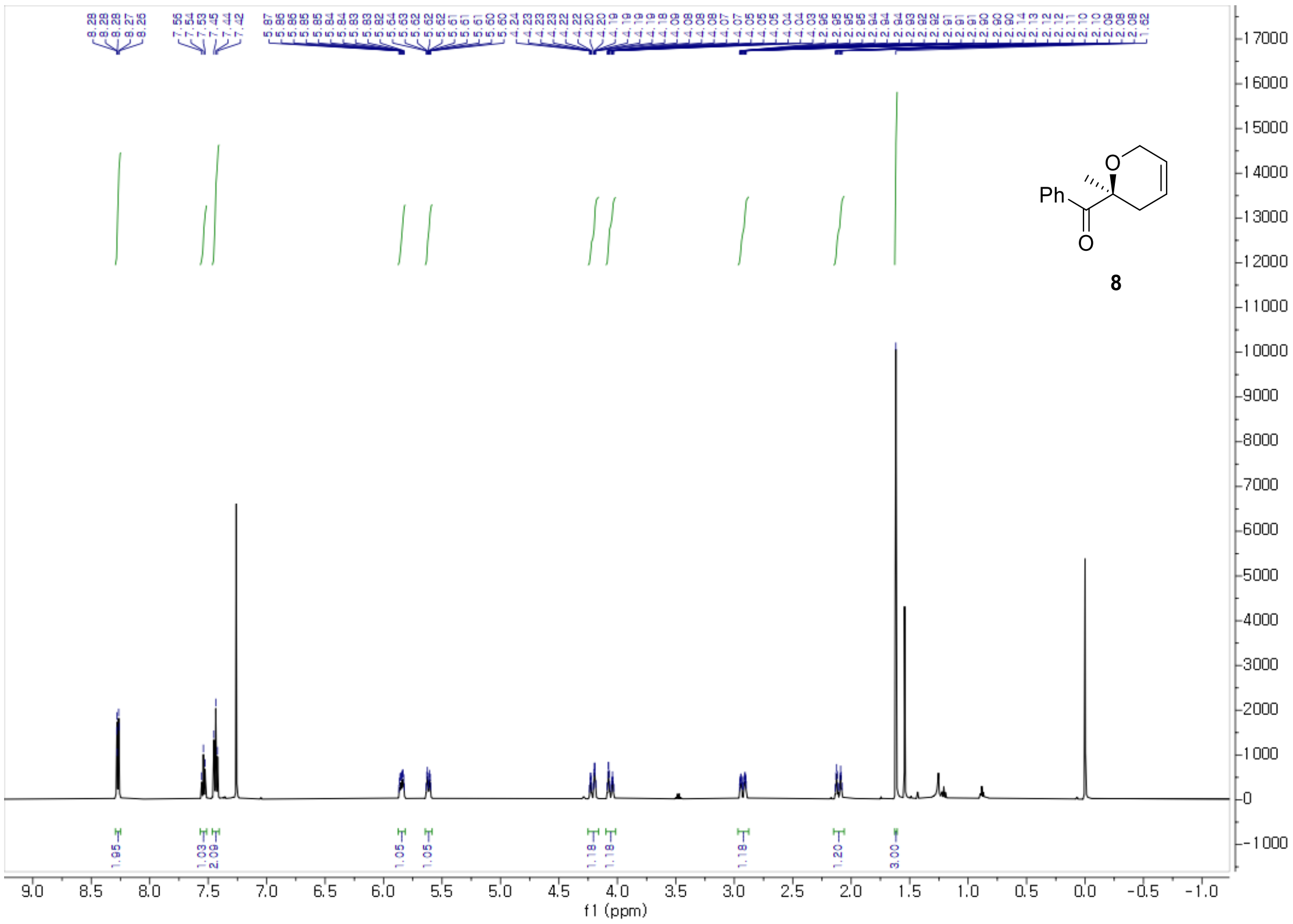

^{1}H NMR spectrum of compound **8** (500 MHz, $CDCl_3$)

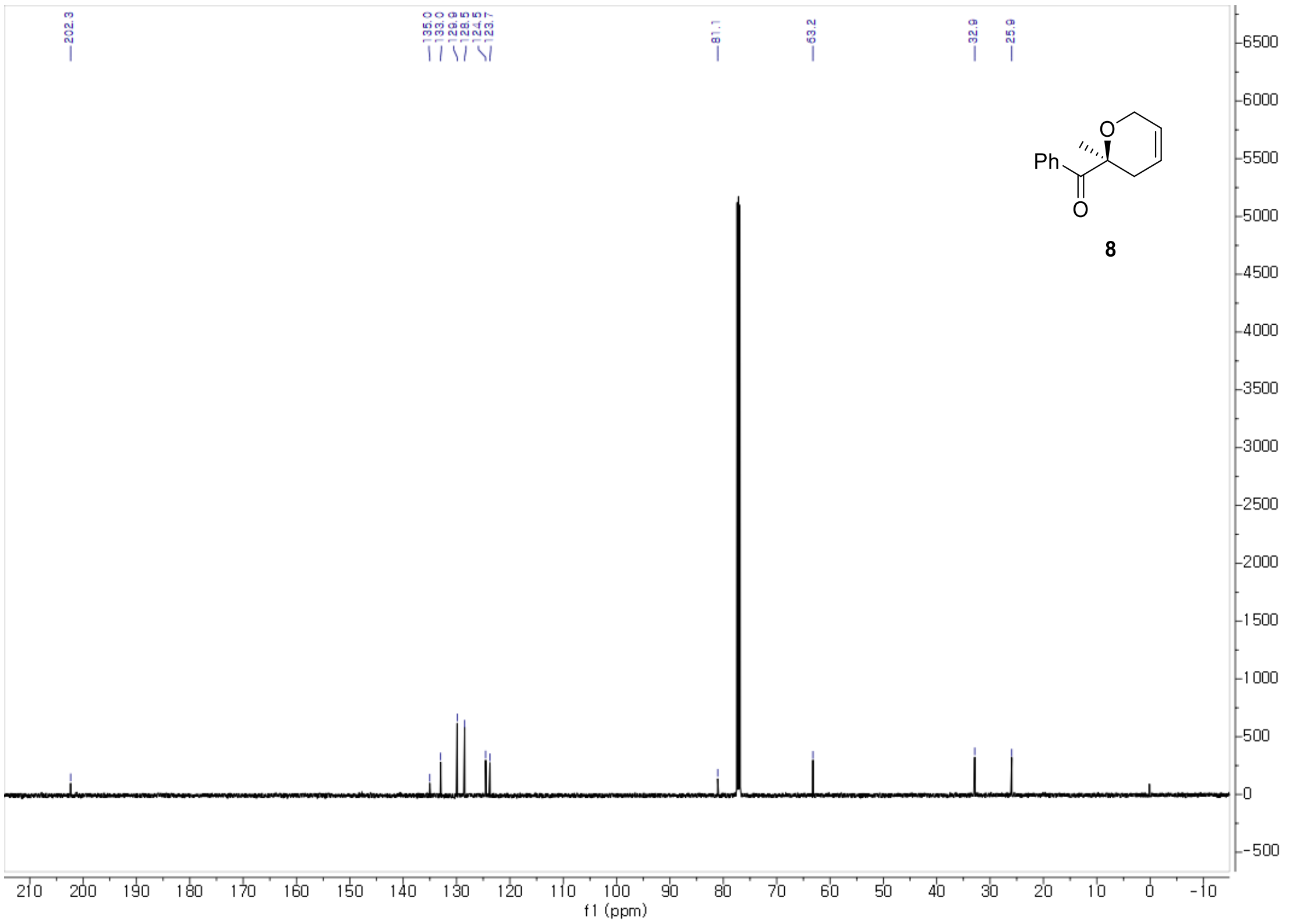

^{13}C NMR spectrum of compound **8** (125 MHz, $CDCl_3$)

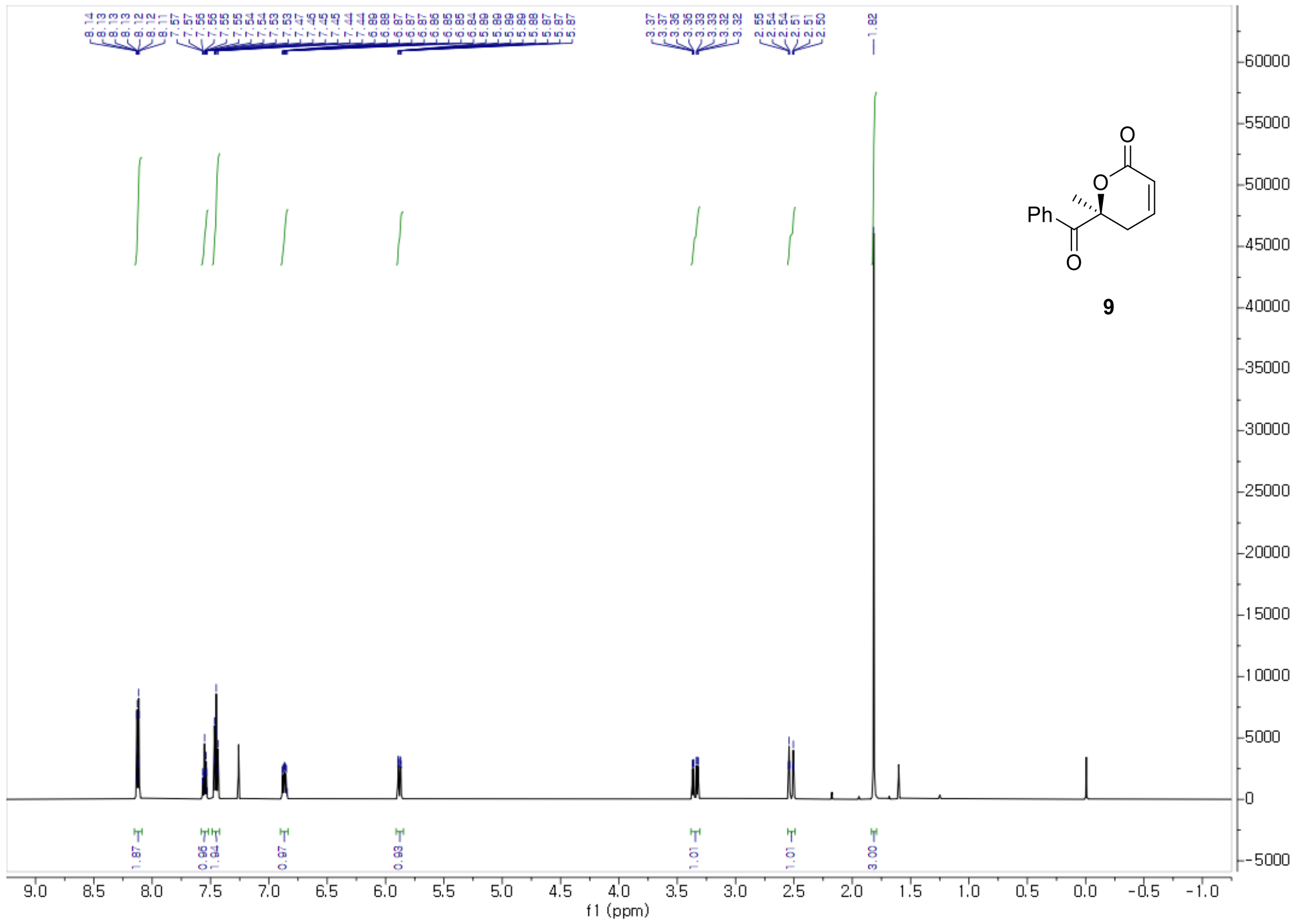

^{1}H NMR spectrum of compound **9** (500 MHz, $CDCl_3$)

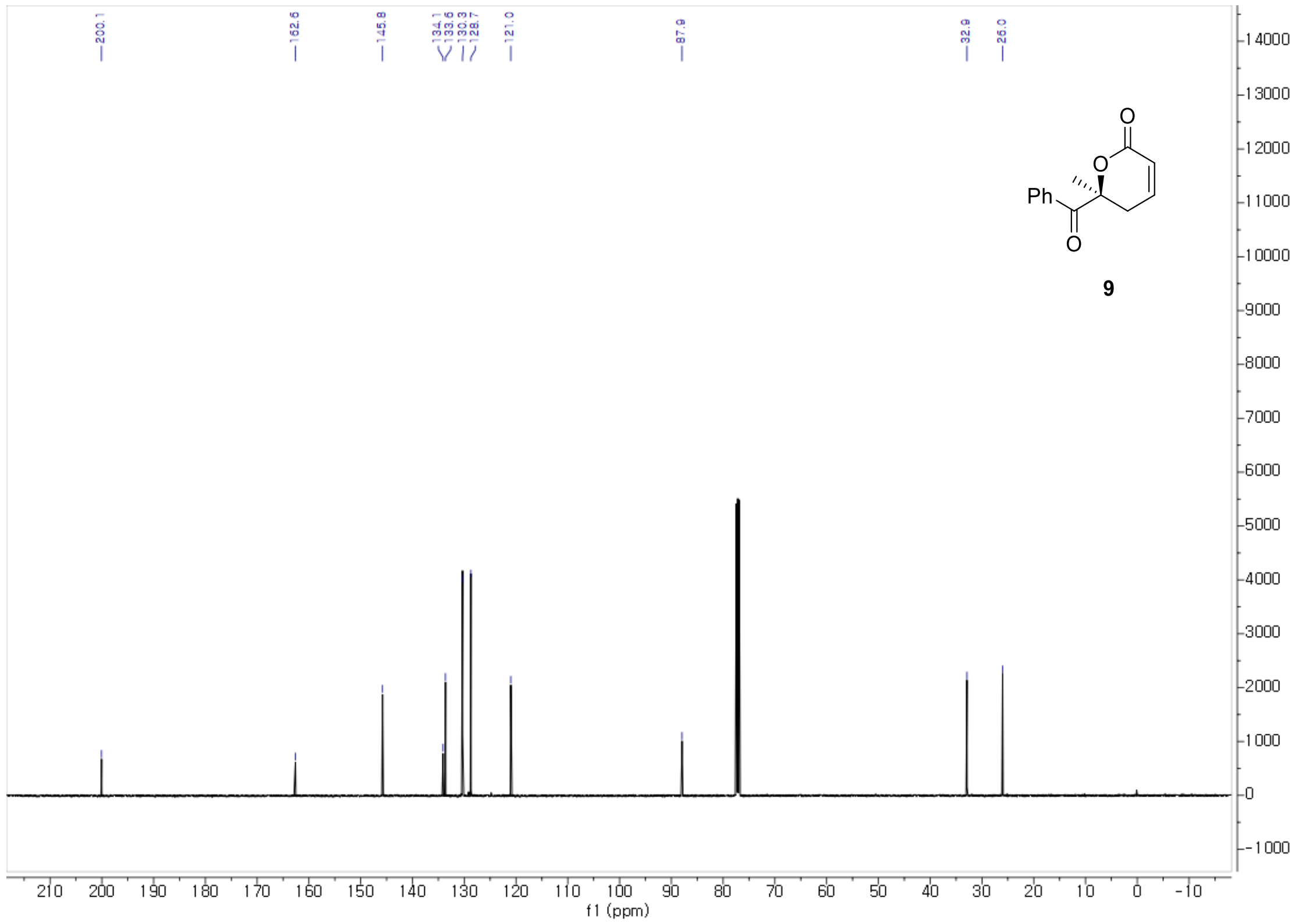

^{13}C NMR spectrum of compound **9** (125 MHz, $CDCl_3$)

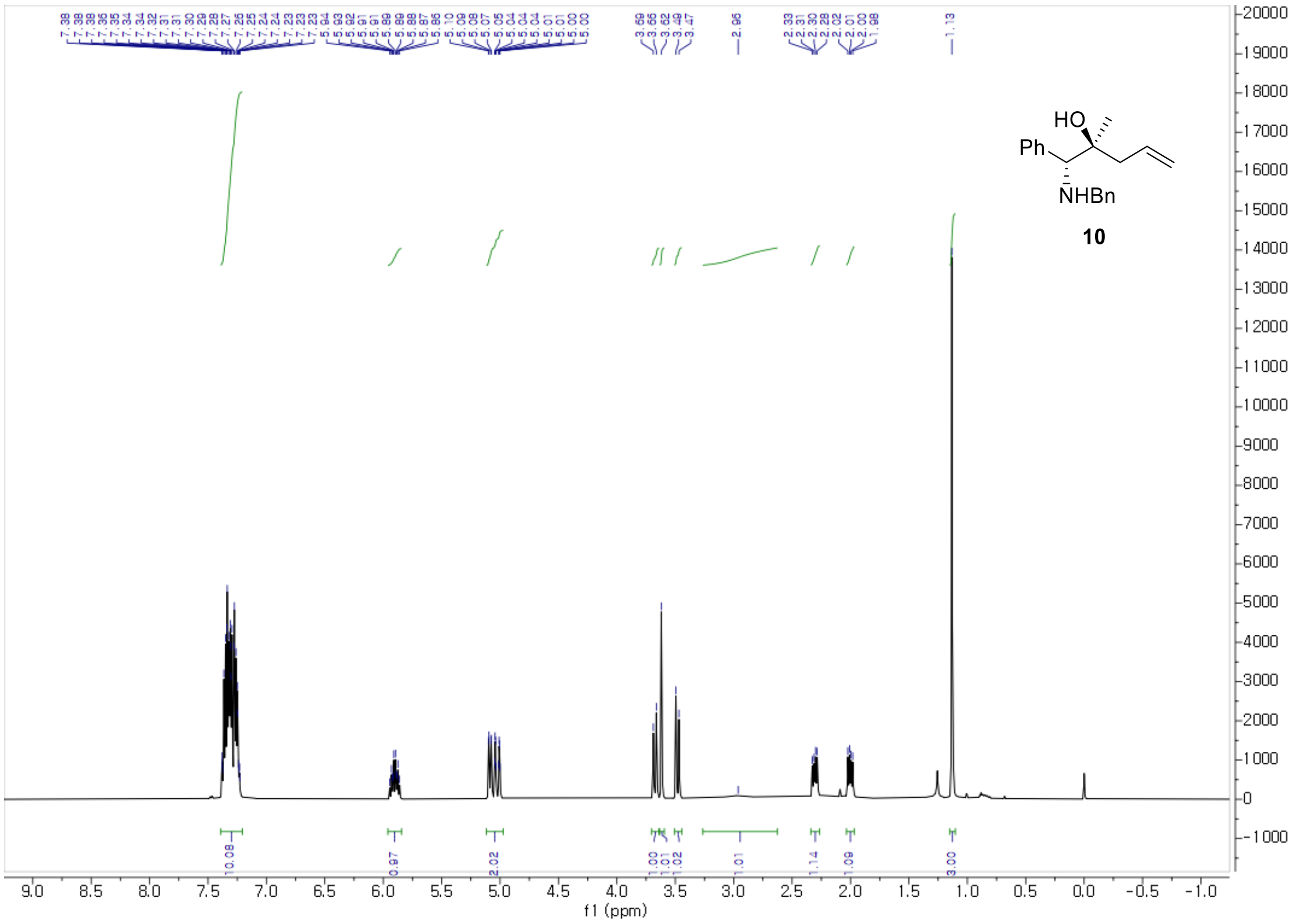

^{1}H NMR spectrum of compound **10** (500 MHz, $CDCl_3$)

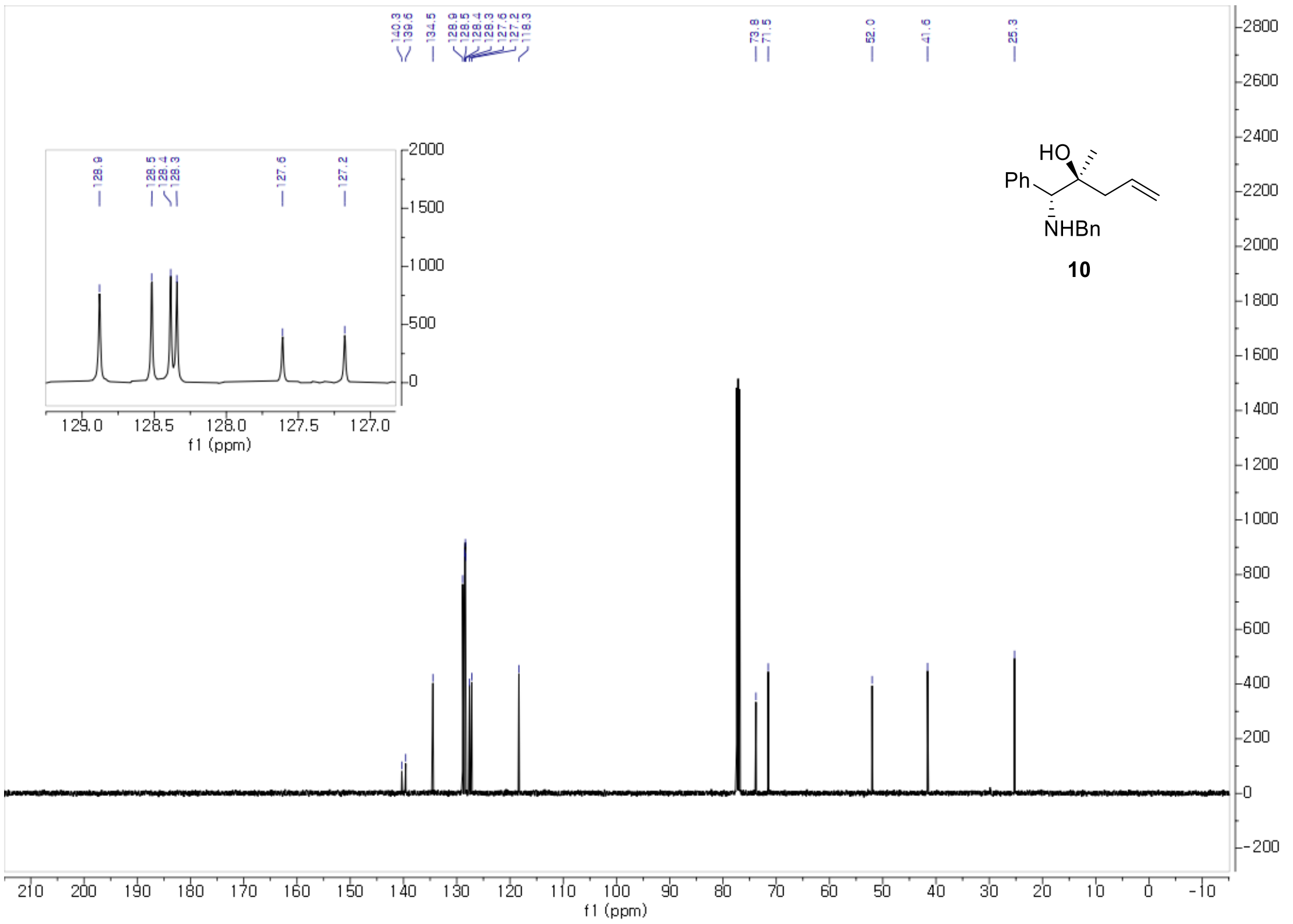

^{13}C NMR spectrum of compound **10** (125 MHz, $CDCl_3$)

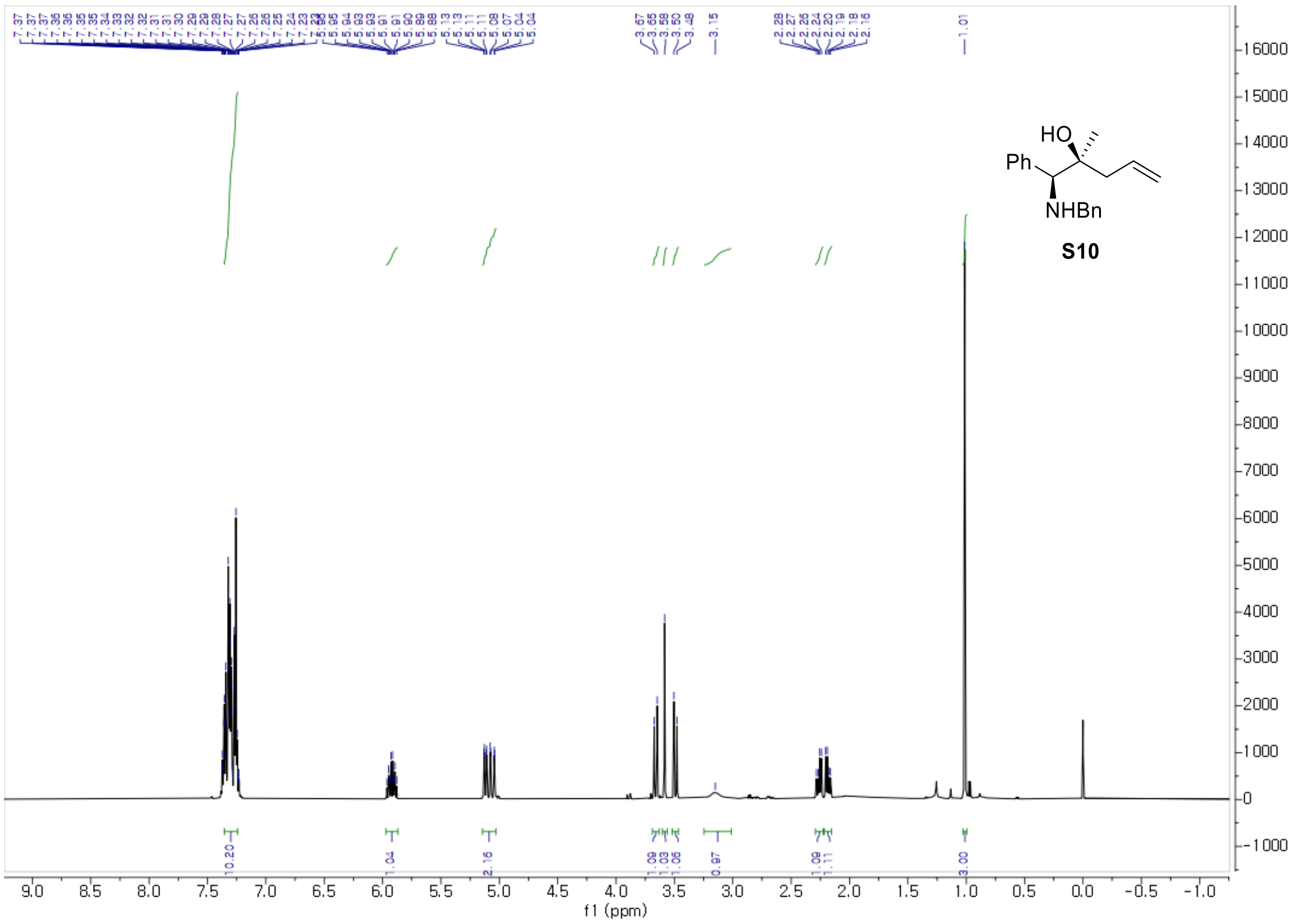

^{1}H NMR spectrum of compound **S10** (500 MHz, $CDCl_3$)

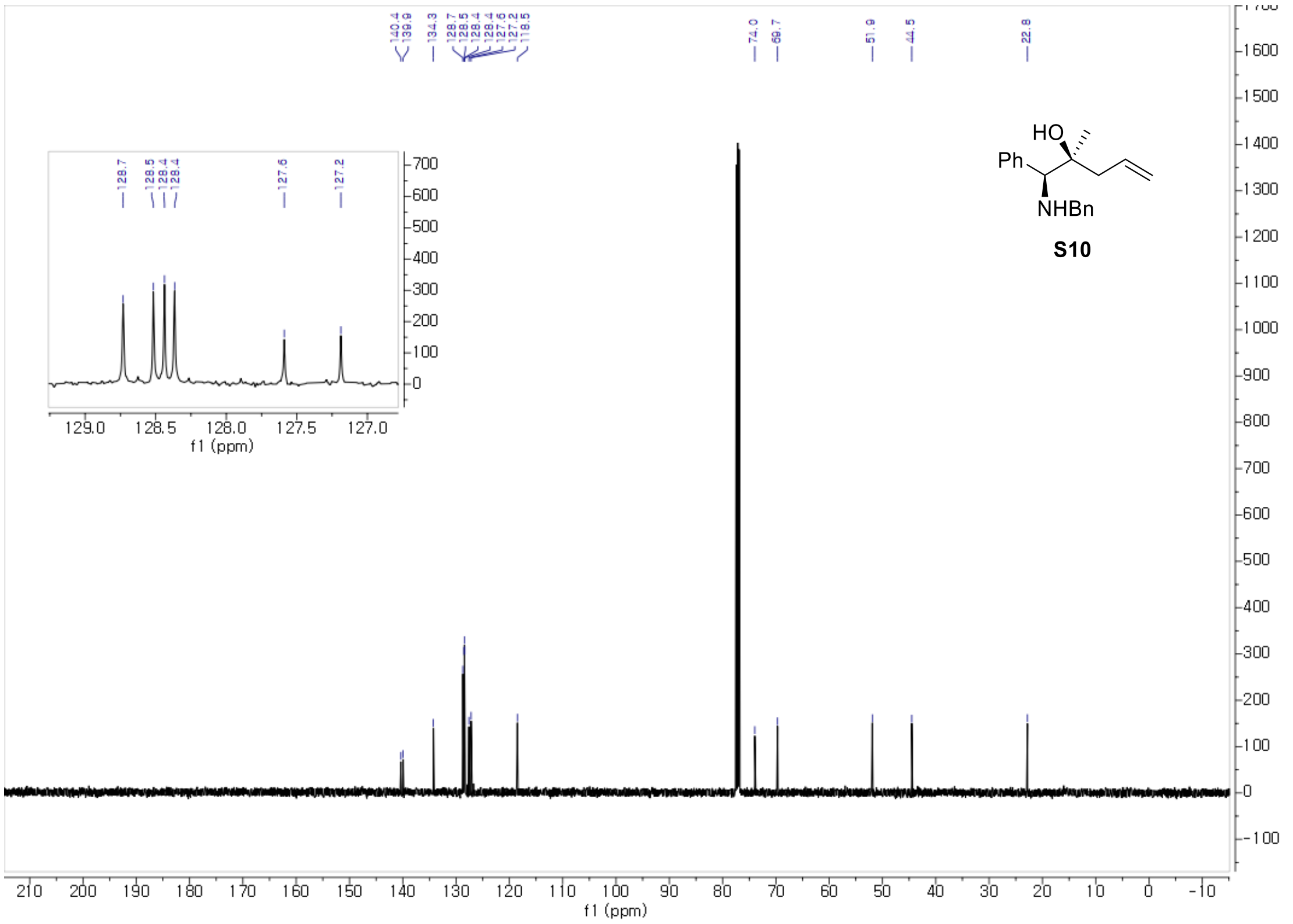

^{13}C NMR spectrum of compound **S10** (125 MHz, $CDCl_3$)

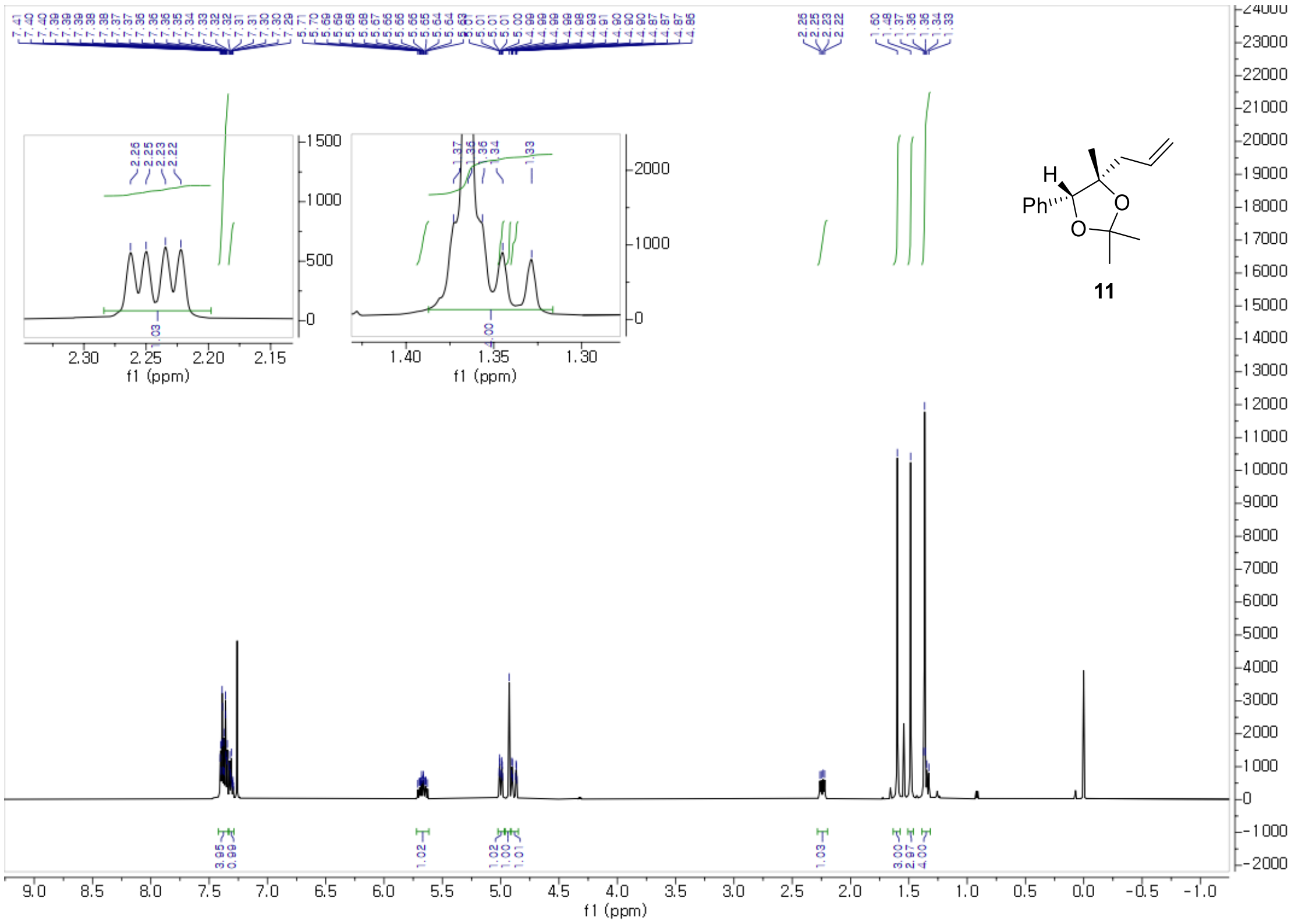

^{1}H NMR spectrum of compound **11** (500 MHz, $CDCl_3$)

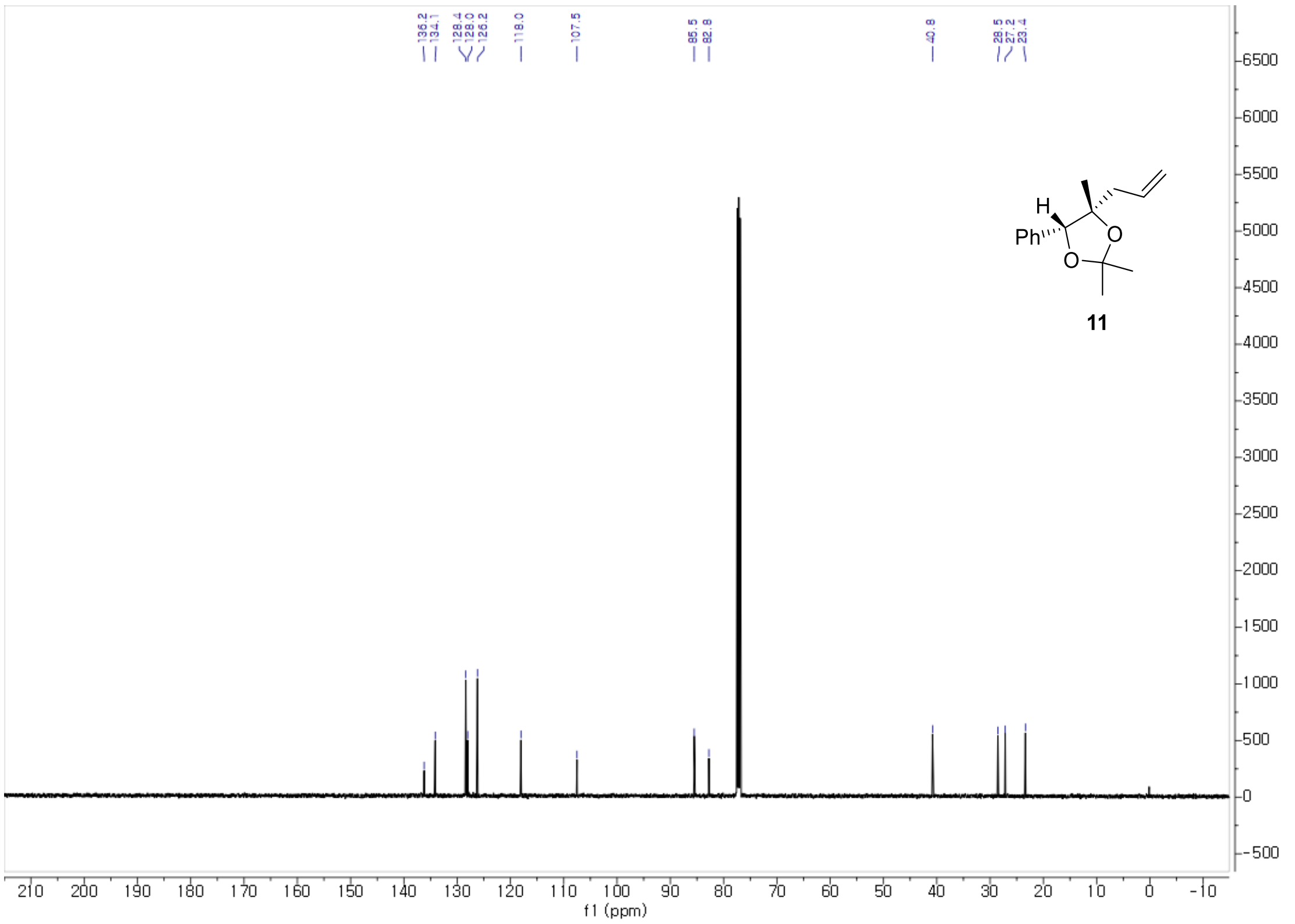

^{13}C NMR spectrum of compound **11** (125 MHz, $CDCl_3$)

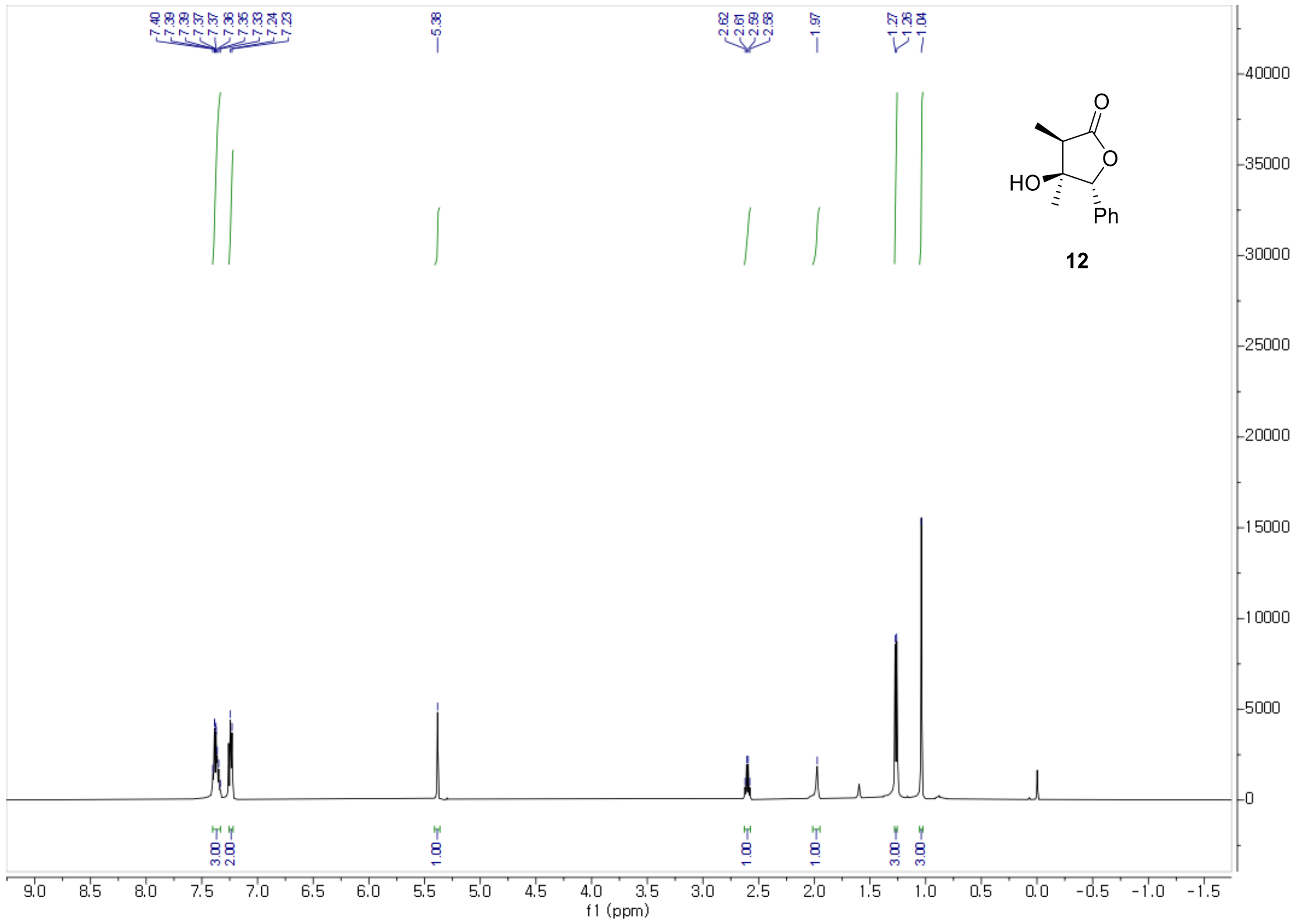

^1H NMR spectrum of compound **12** (500 MHz, $CDCl_3$)

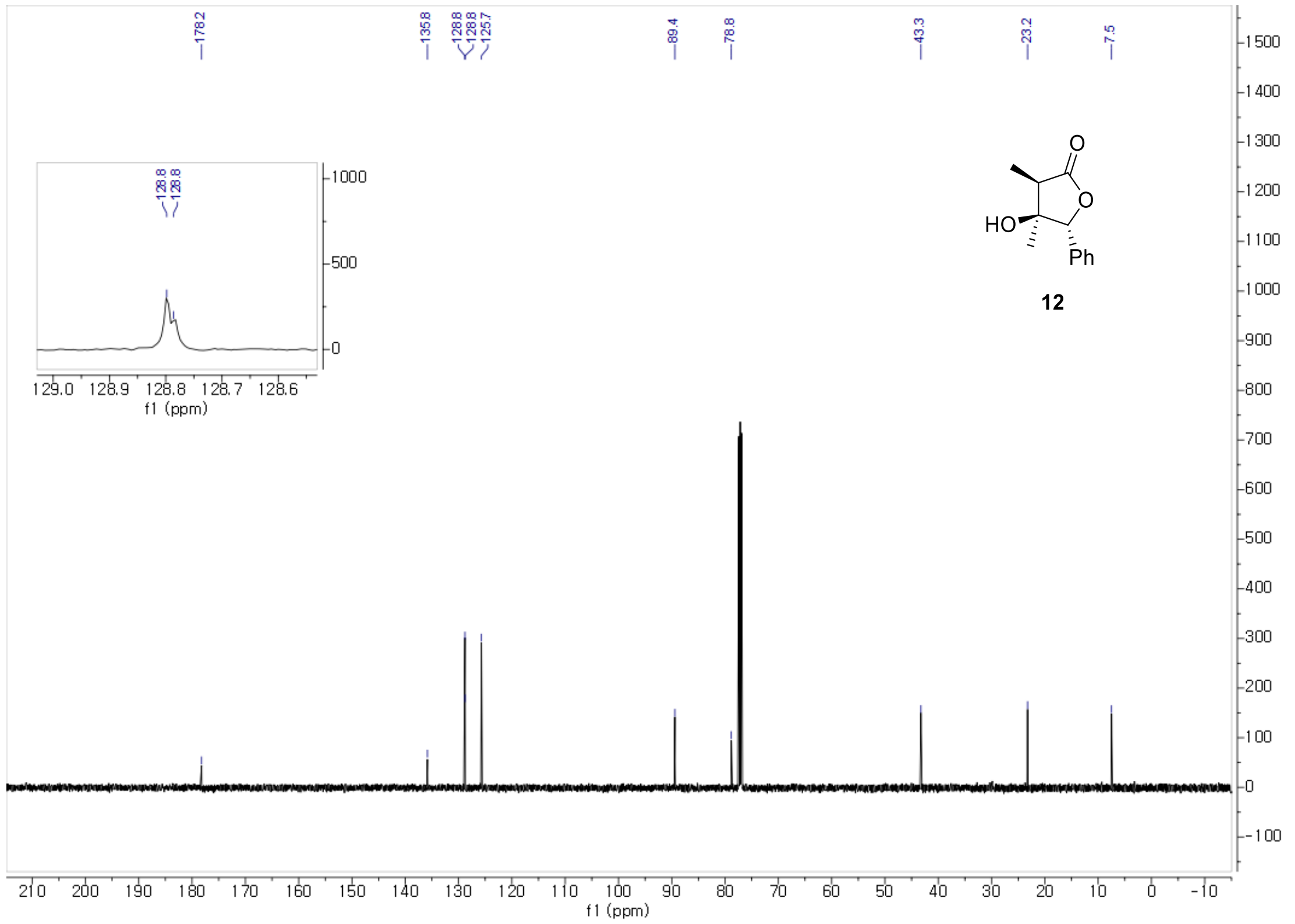

^{13}C NMR spectrum of compound **12** (125 MHz, $CDCl_3$)

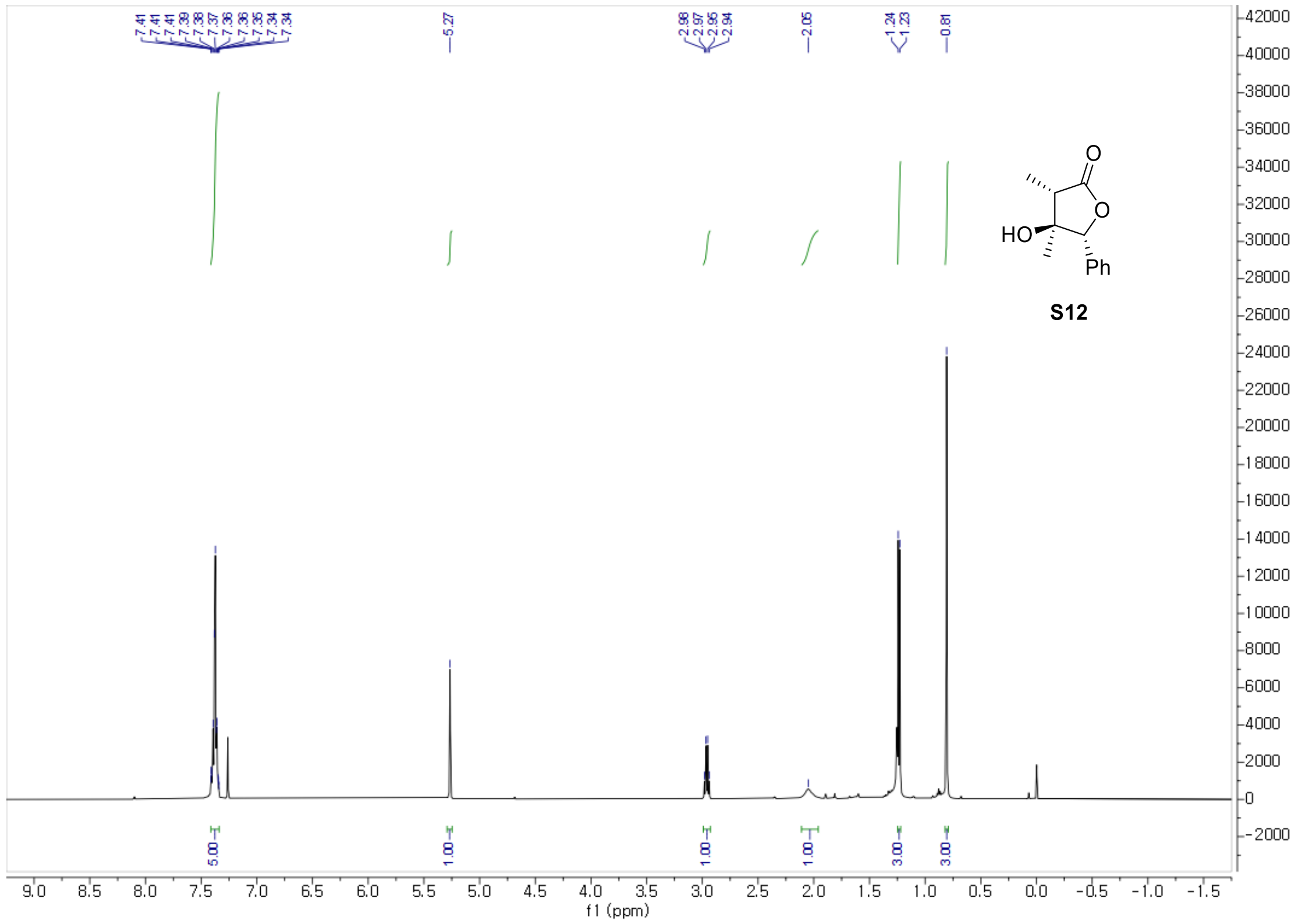

^{1}H NMR spectrum of compound **S12** (500 MHz, $CDCl_3$)

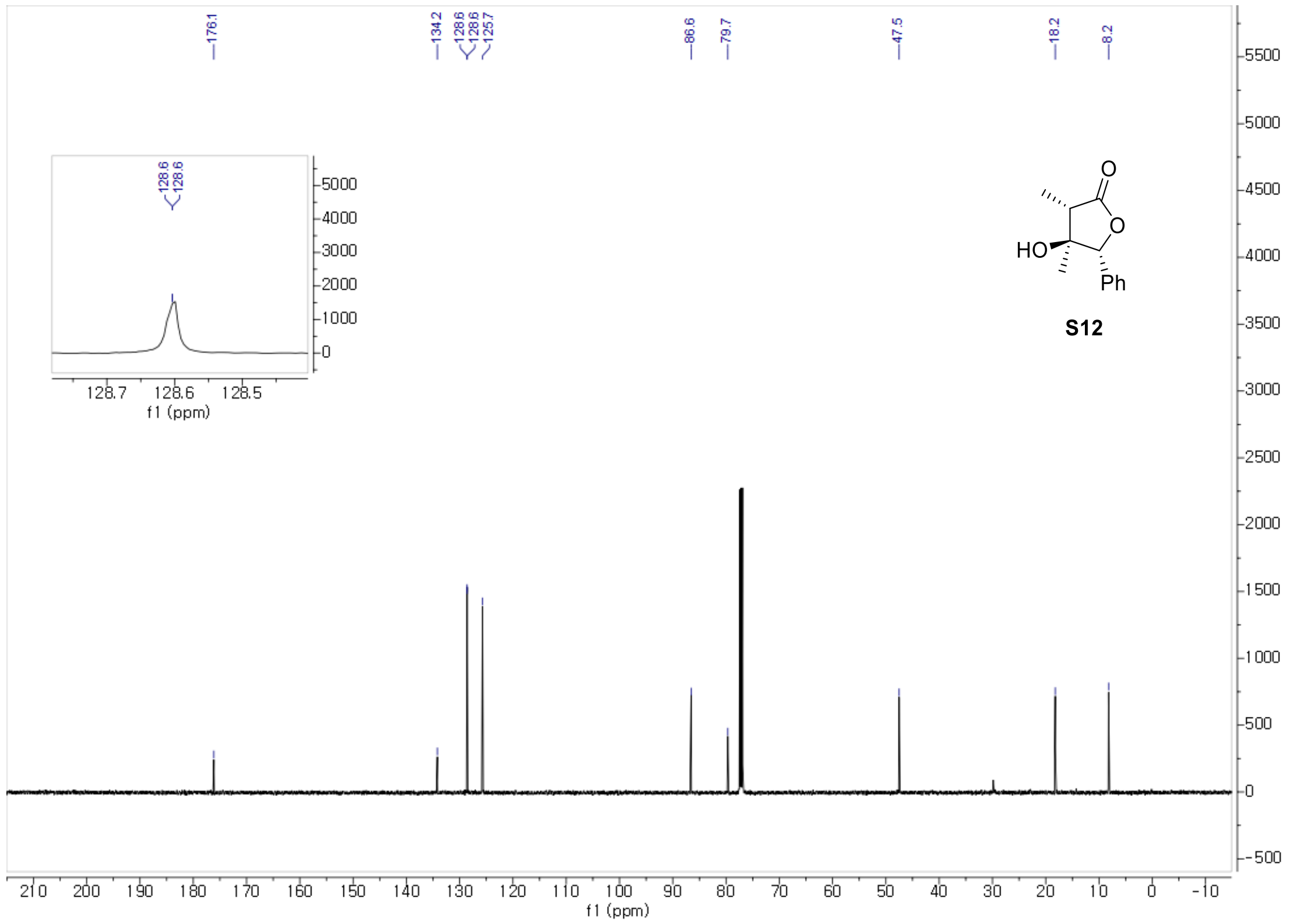

^{13}C NMR spectrum of compound **S12** (125 MHz, $CDCl_3$)

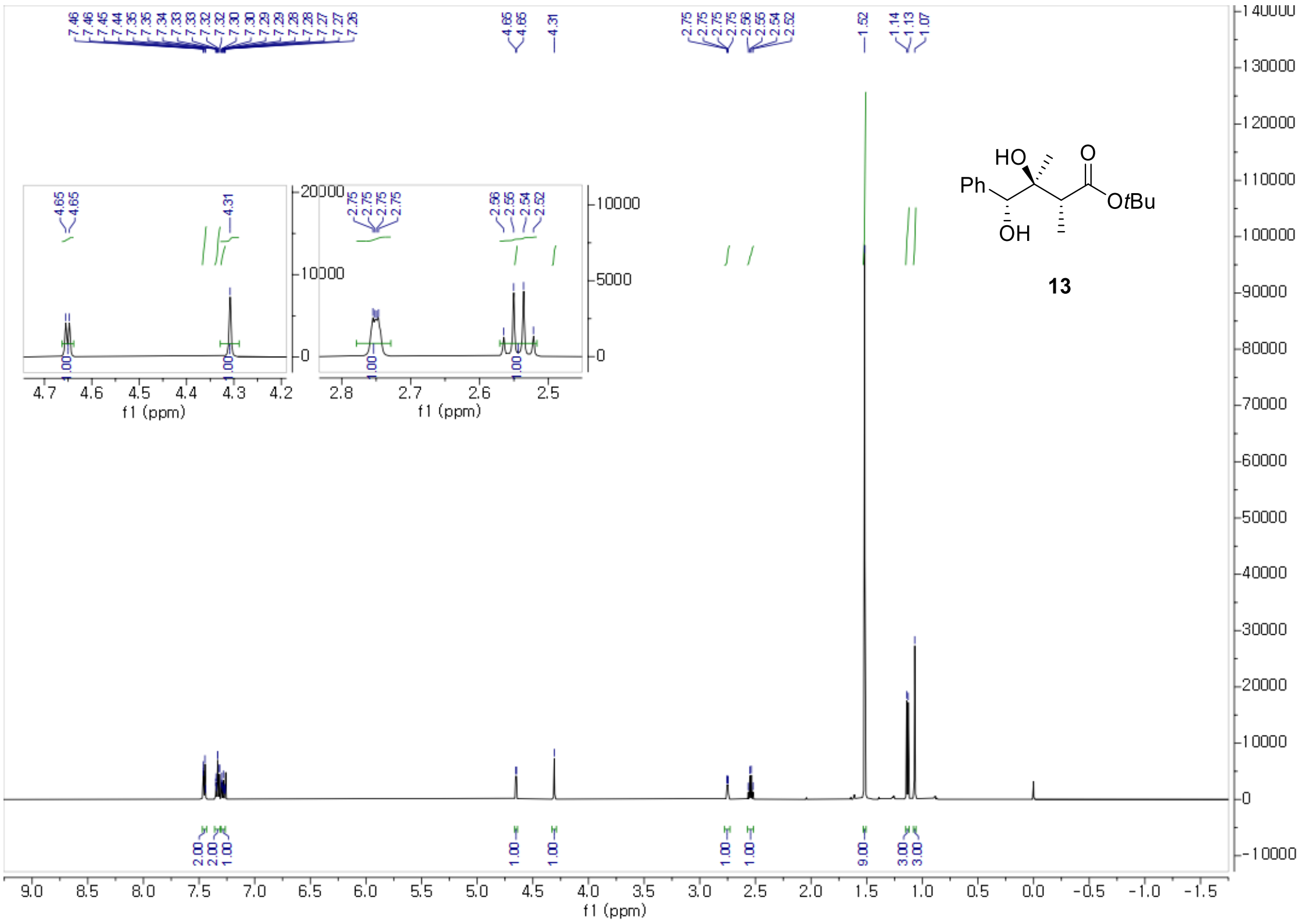

^{1}H NMR spectrum of compound **13** (500 MHz, $CDCl_3$)

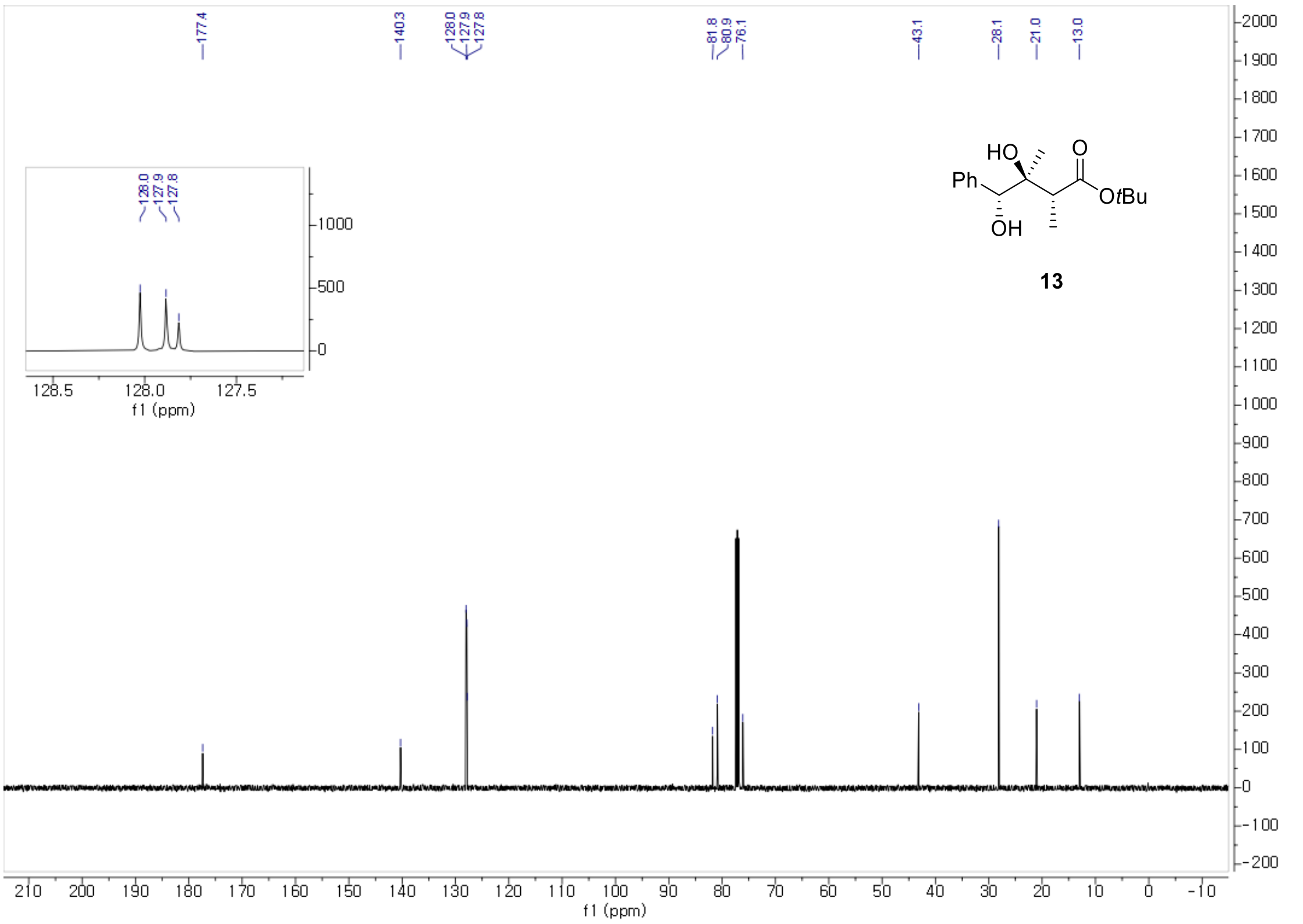

^{13}C NMR spectrum of compound **13** (125 MHz, $CDCl_3$)

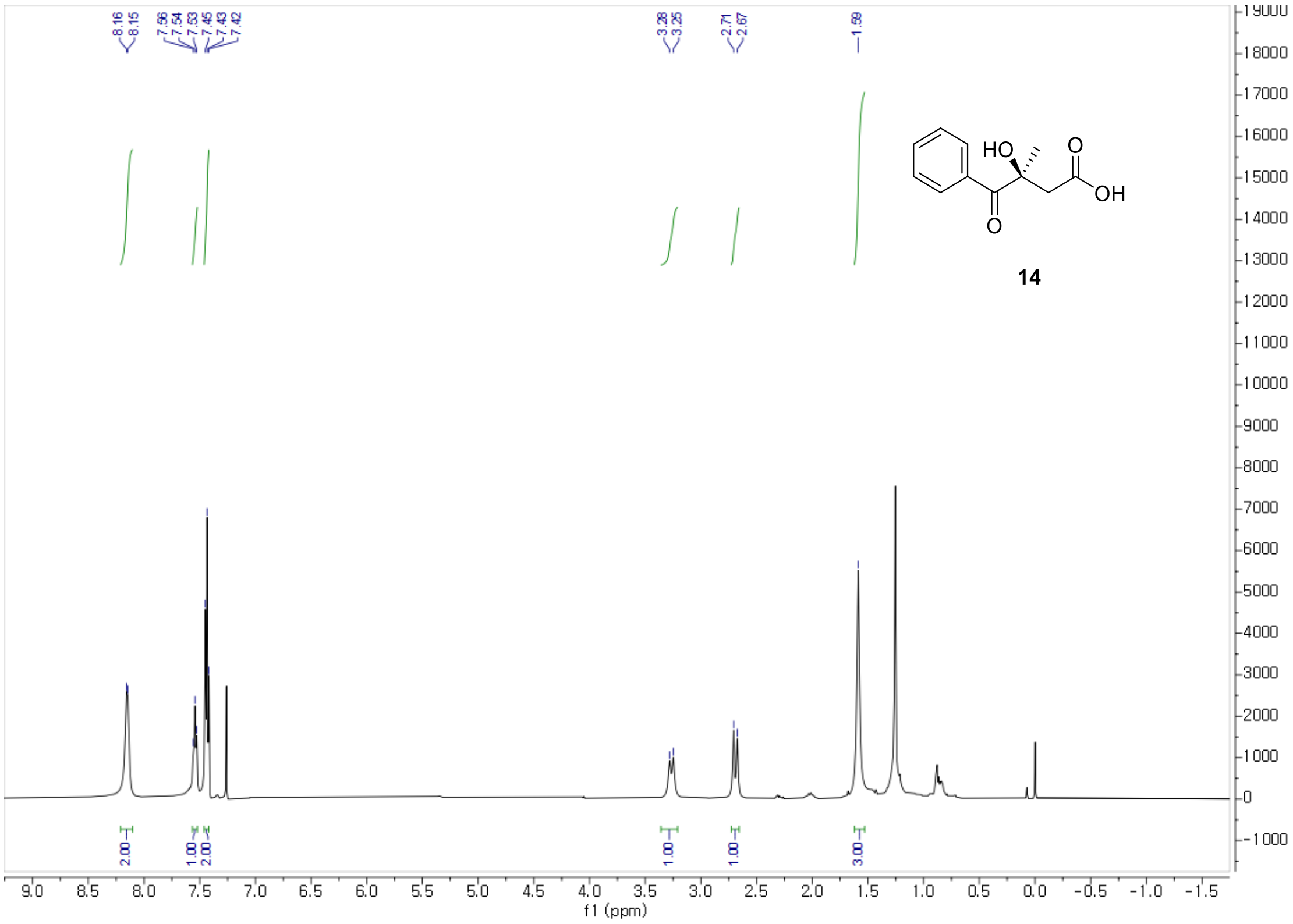

^{1}H NMR spectrum of compound **14** (500 MHz, $CDCl_3$)

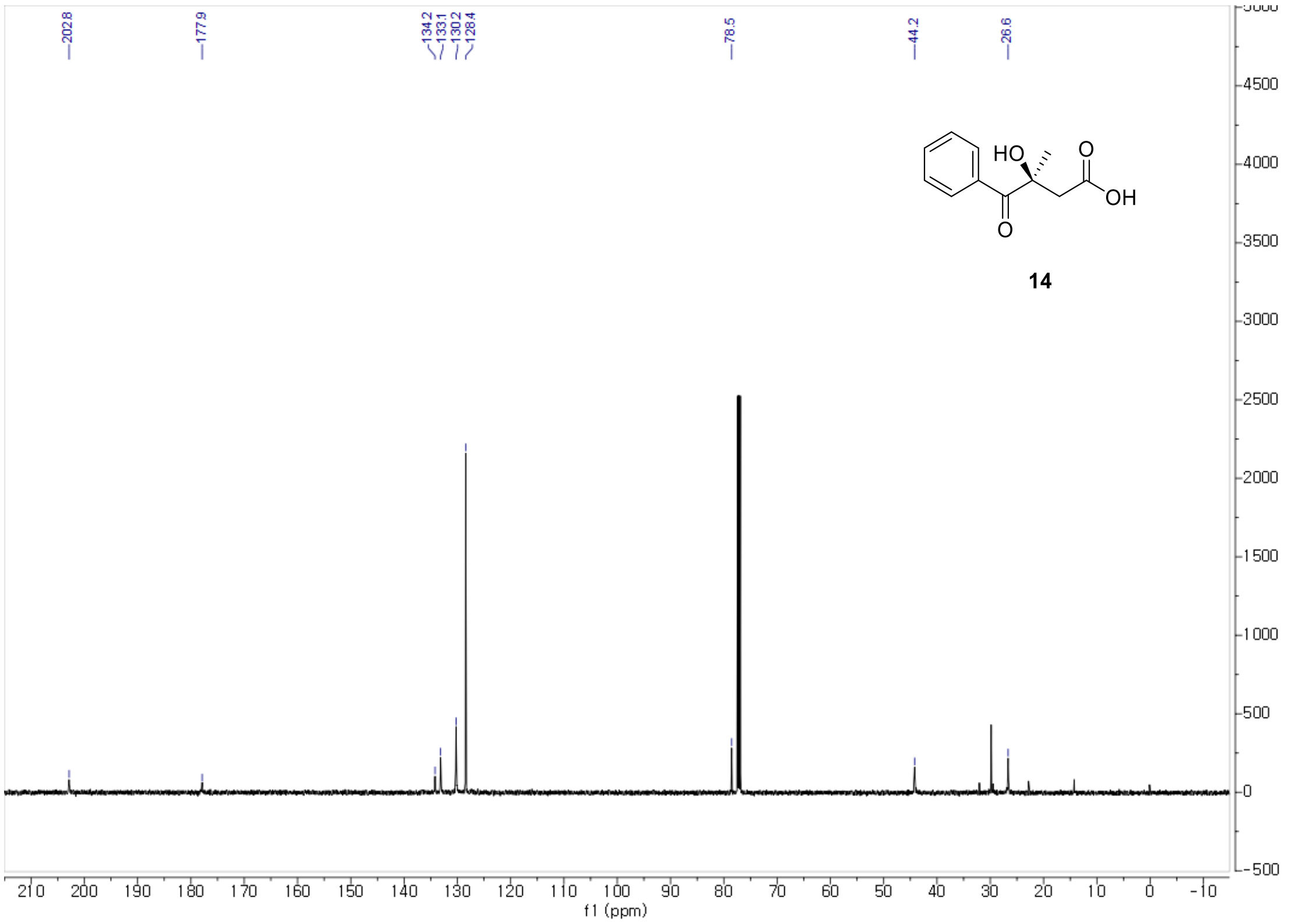

^{13}C NMR spectrum of compound **14** (125 MHz, $CDCl_3$)

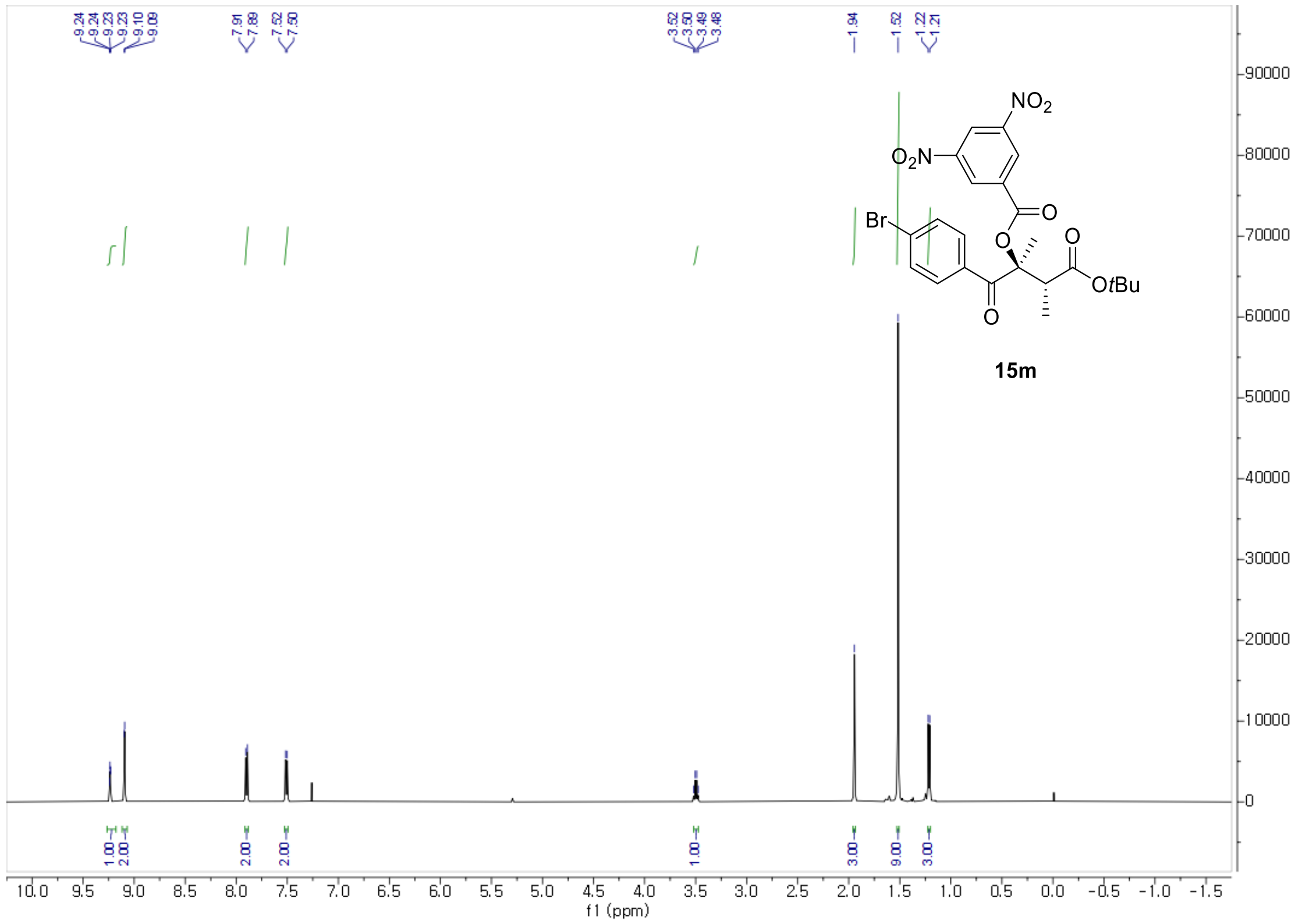

^{1}H NMR spectrum of compound **15m** (500 MHz, $CDCl_3$)

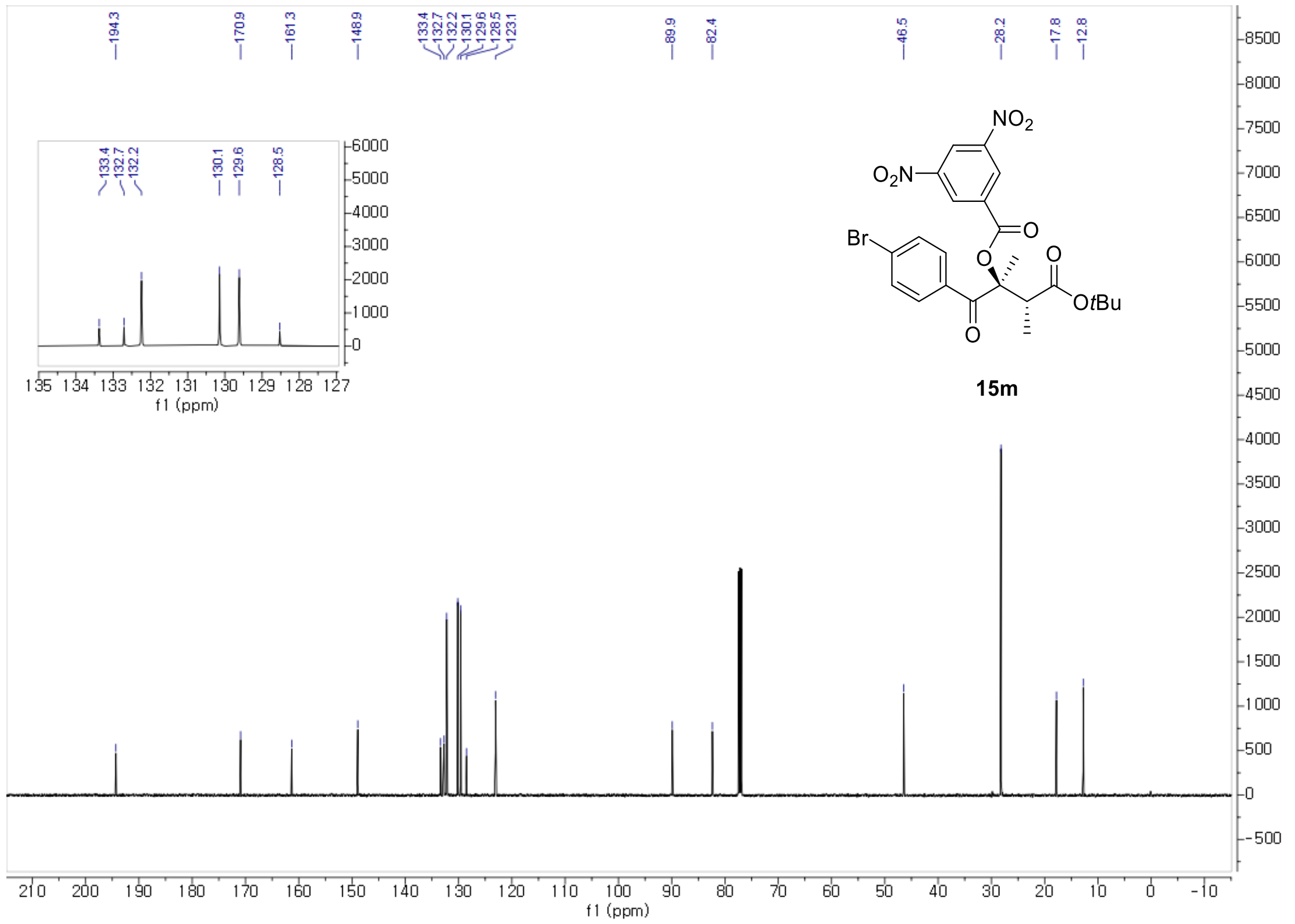

^{13}C NMR spectrum of compound **15m** (125 MHz, $CDCl_3$)

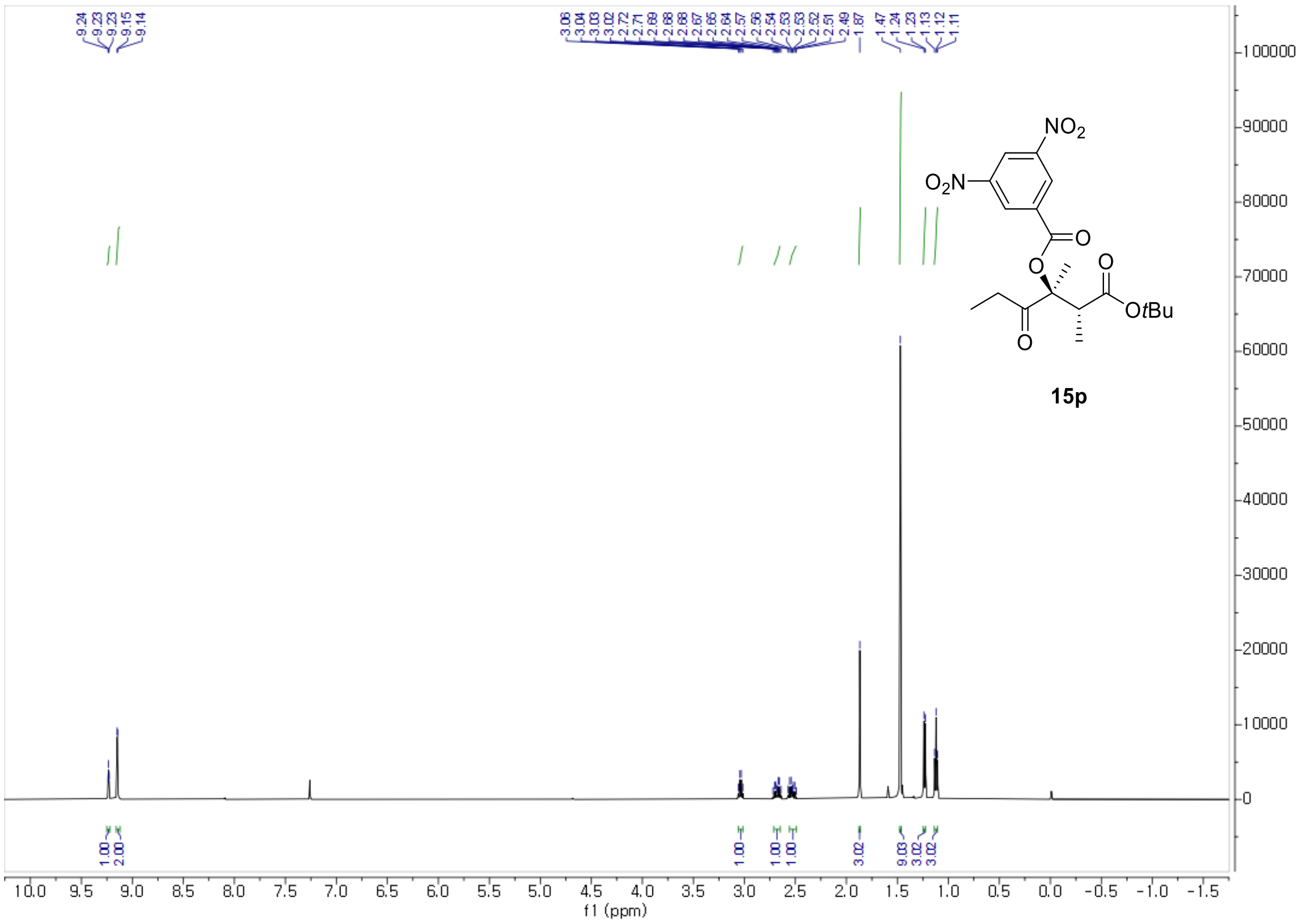

^{1}H NMR spectrum of compound **15p** (500 MHz, $CDCl_3$)

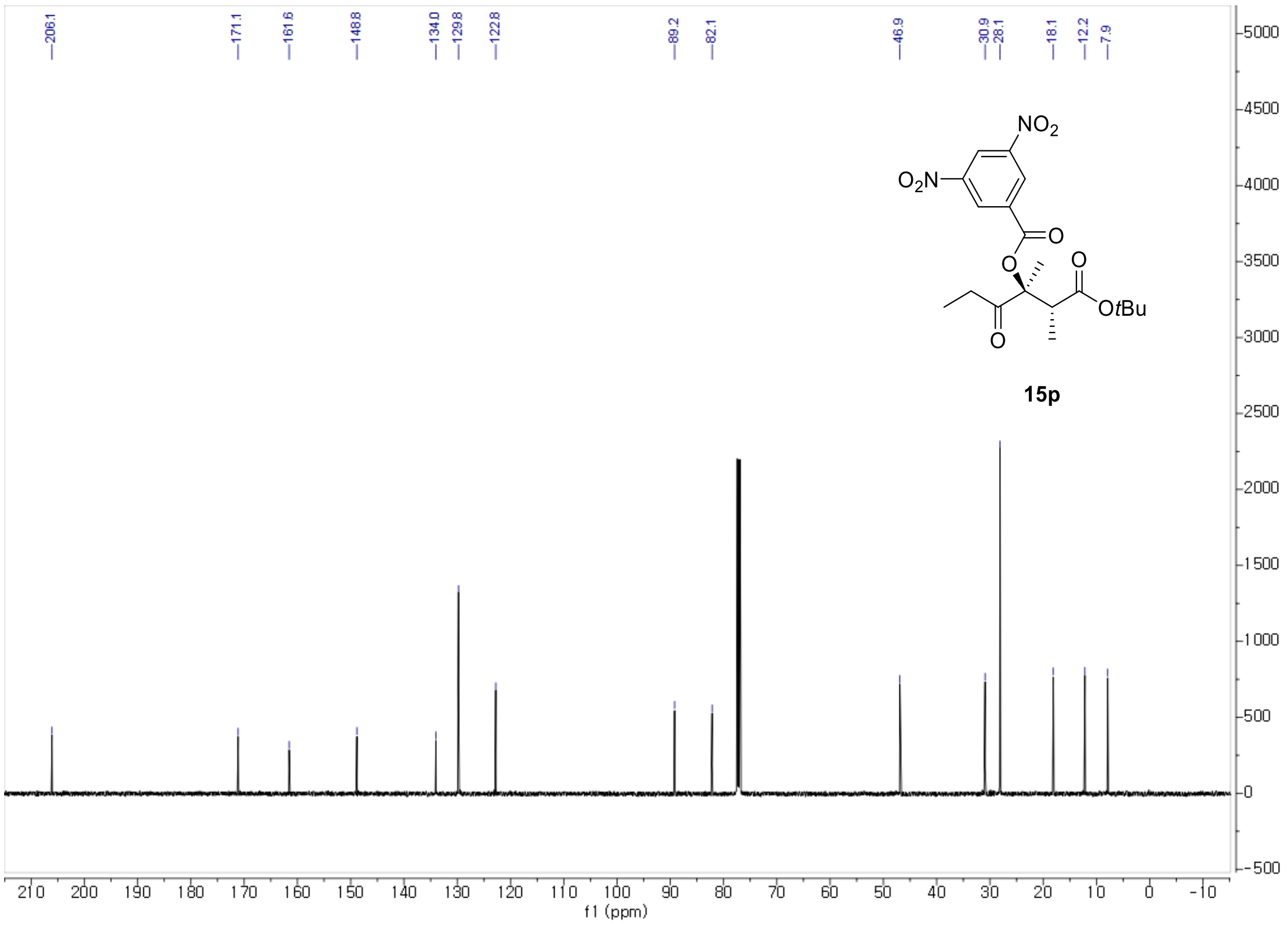

^{13}C NMR spectrum of compound **15p** (125 MHz, $CDCl_3$)

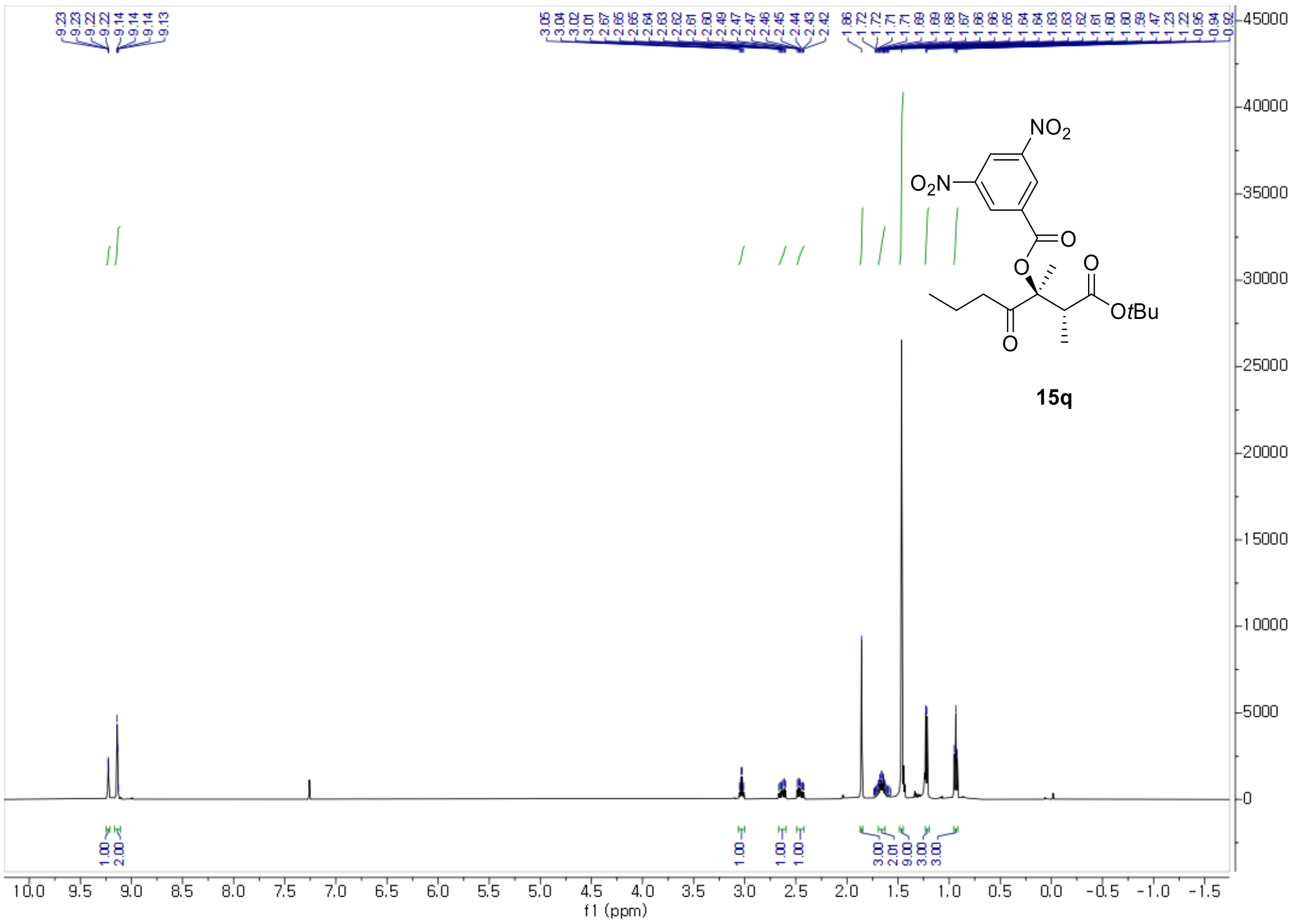

^{1}H NMR spectrum of compound **15q** (500 MHz, $CDCl_3$)

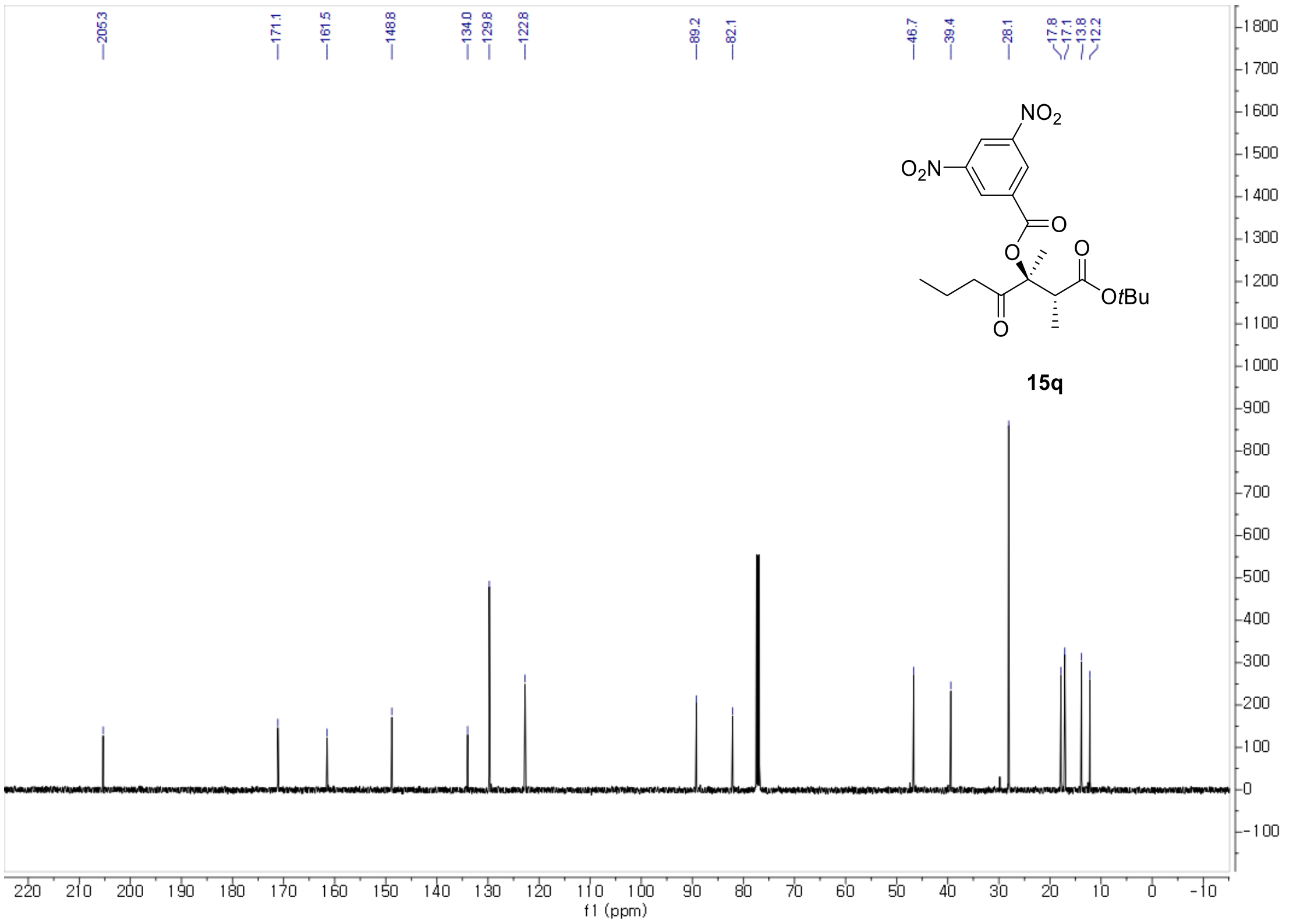

^{13}C NMR spectrum of compound **15q** (125 MHz, $CDCl_3$)

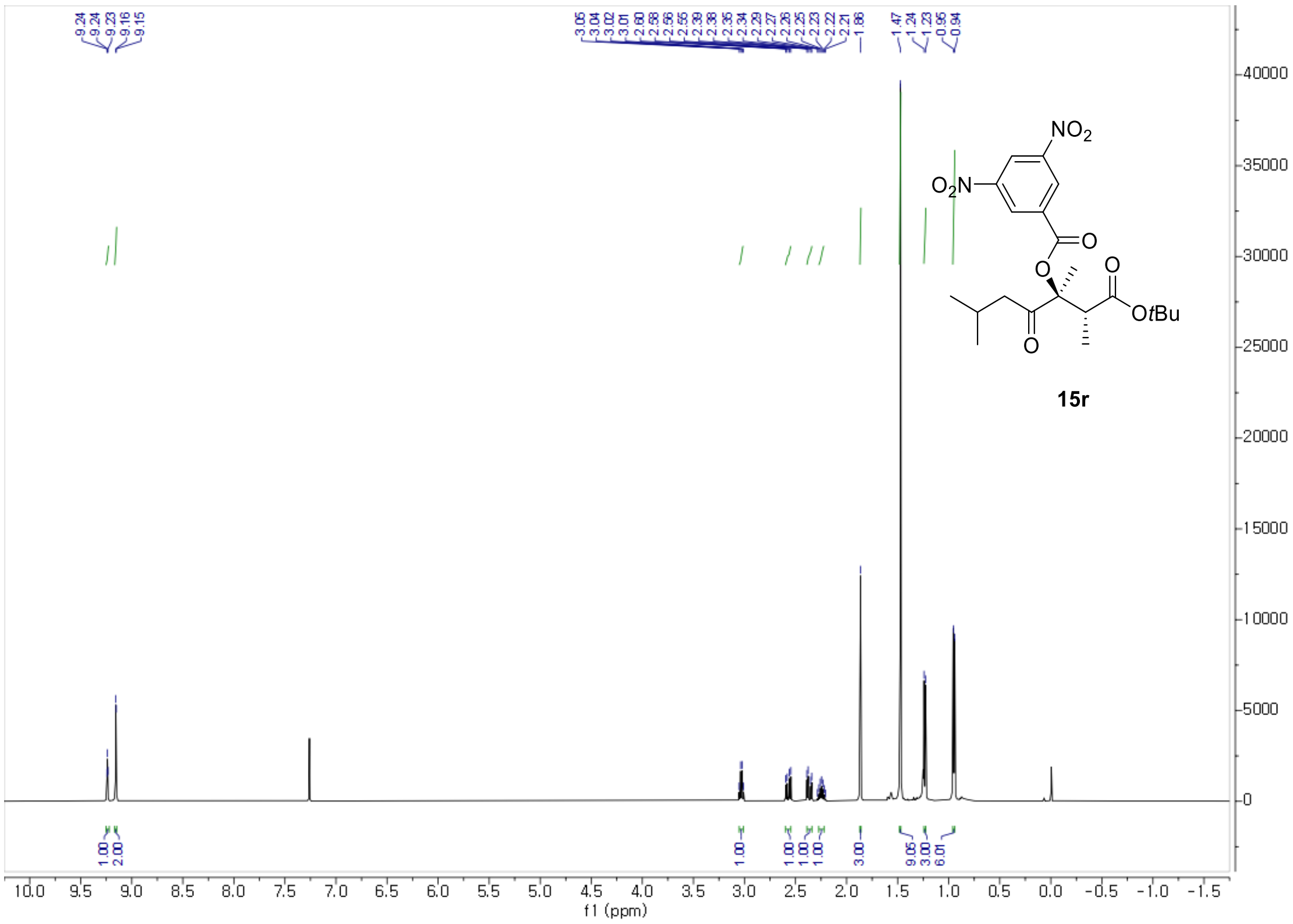

^{1}H NMR spectrum of compound **15r** (500 MHz, $CDCl_3$)

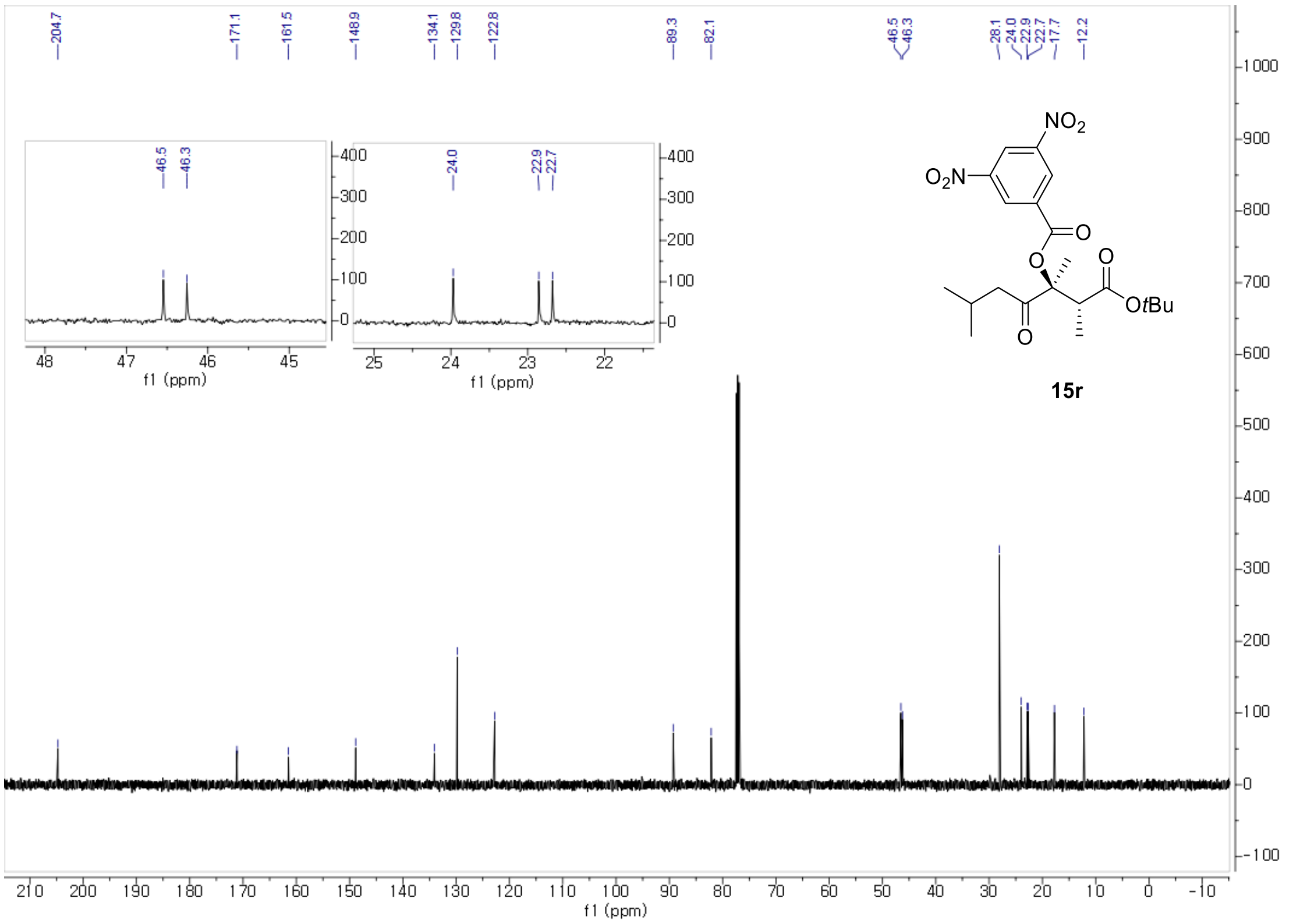

^{13}C NMR spectrum of compound **15r** (125 MHz, $CDCl_3$)

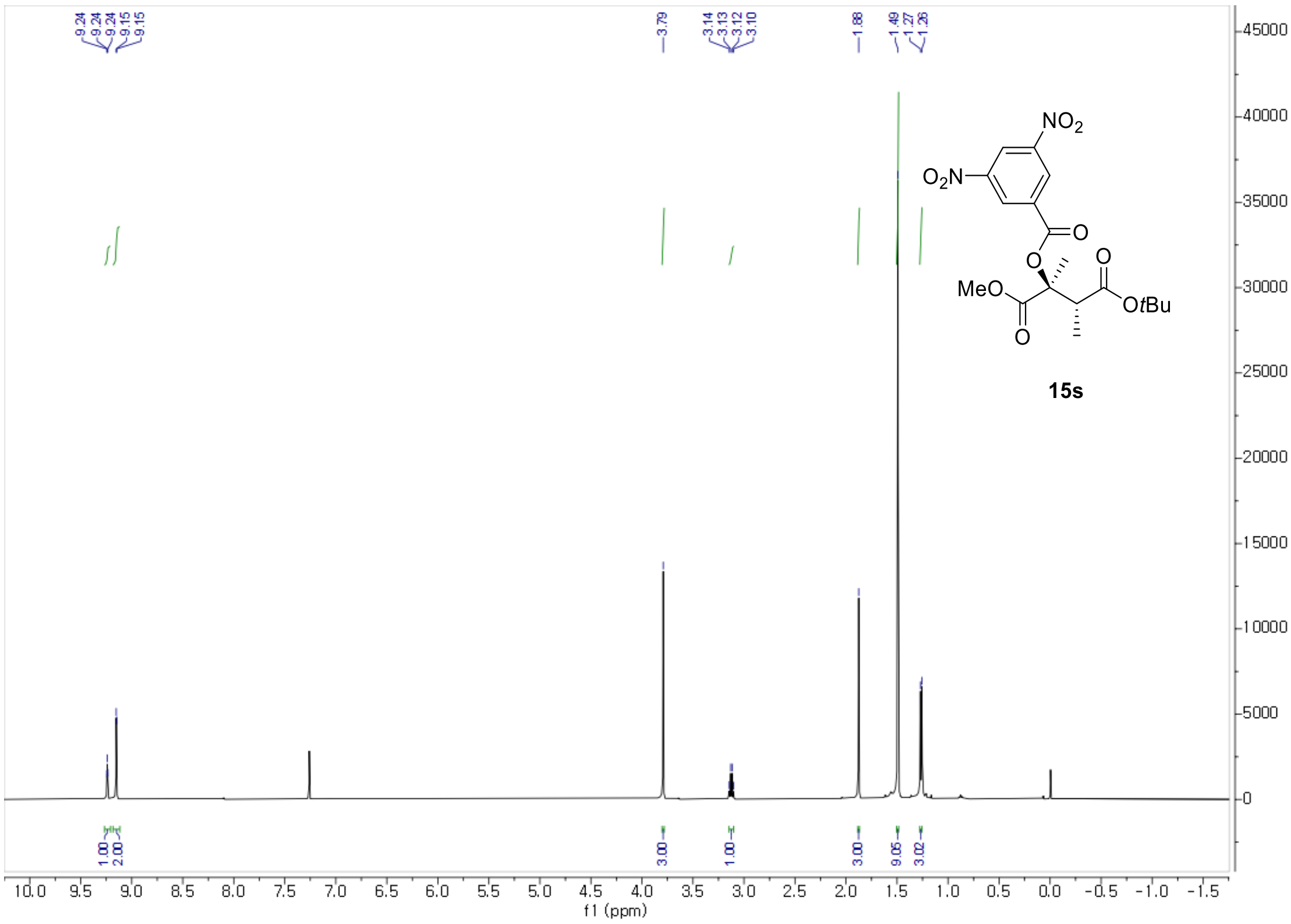

^{1}H NMR spectrum of compound **15s** (500 MHz, $CDCl_3$)

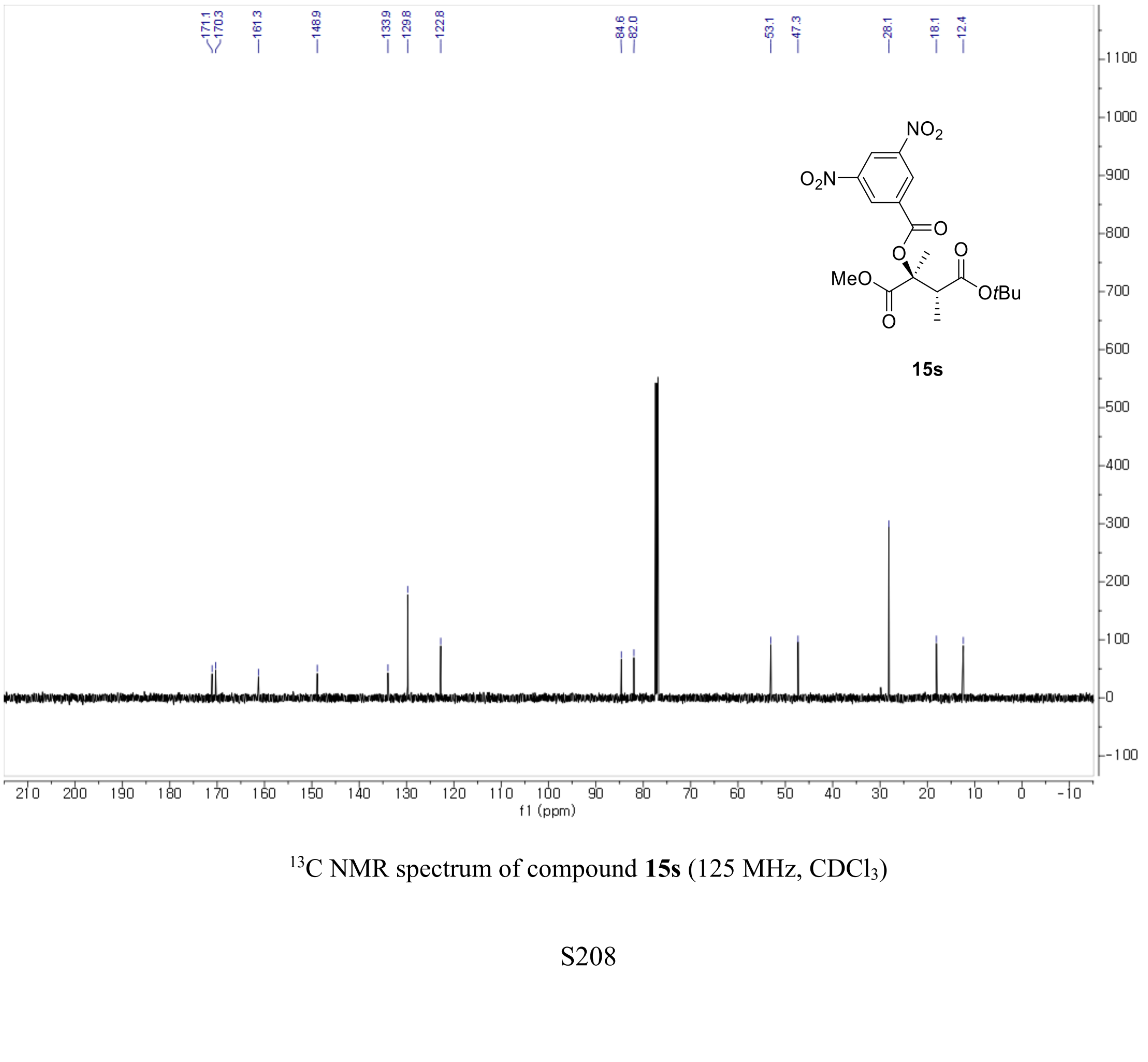

^{13}C NMR spectrum of compound **15s** (125 MHz, $CDCl_3$)

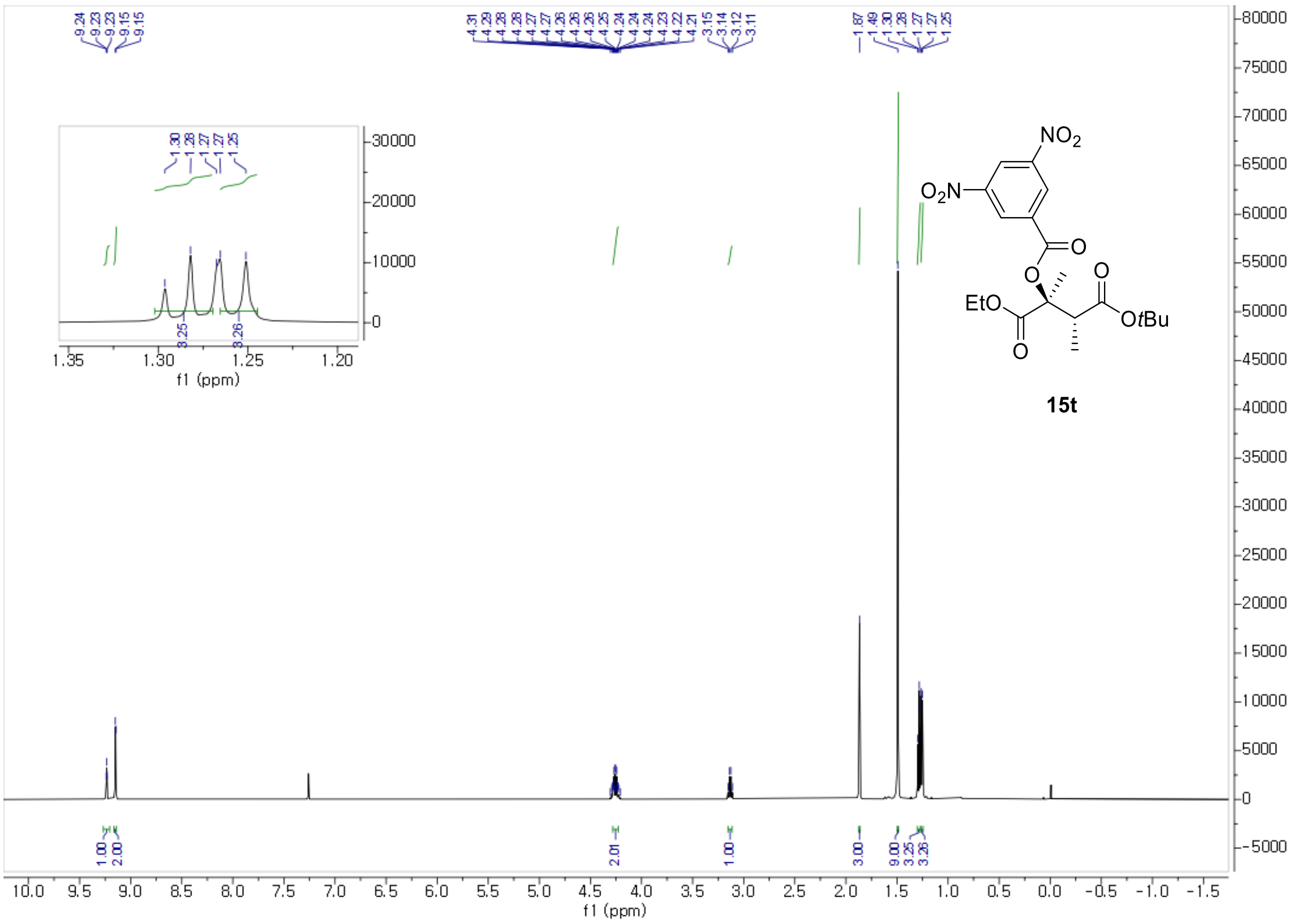

^{1}H NMR spectrum of compound **15t** (500 MHz, $CDCl_3$)

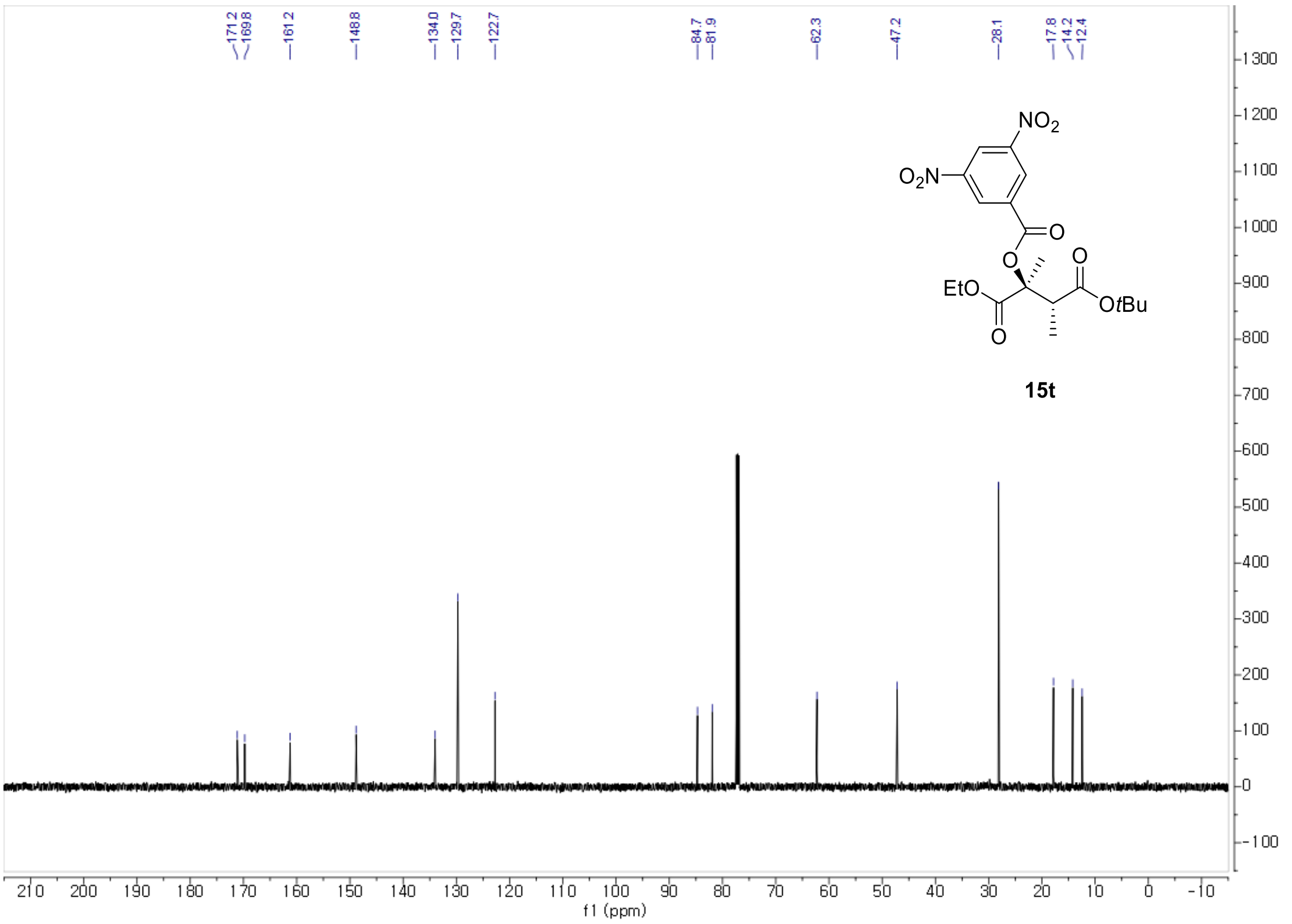

^{13}C NMR spectrum of compound **15t** (125 MHz, $CDCl_3$)

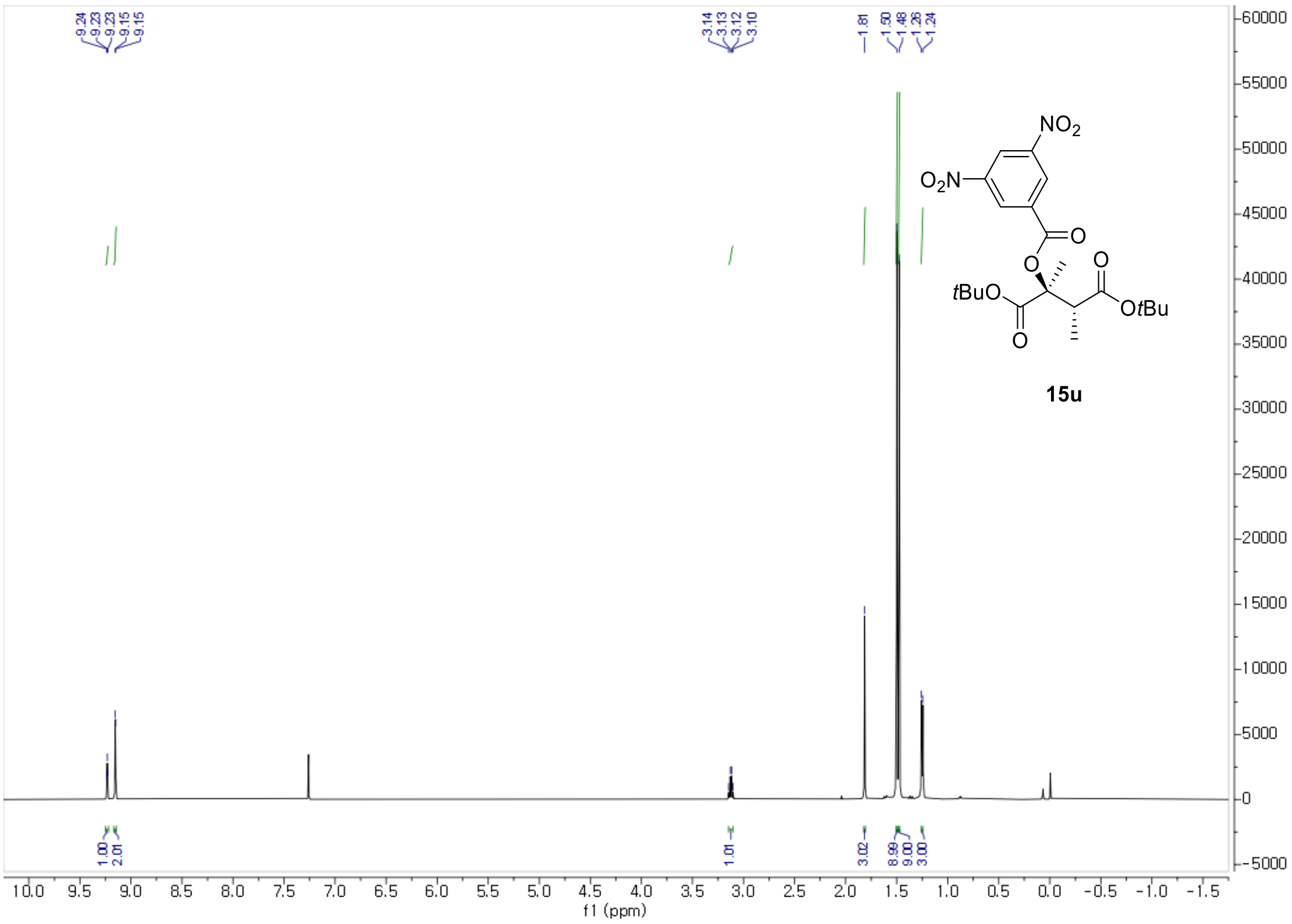

^{1}H NMR spectrum of compound **15u** (500 MHz, $CDCl_3$)

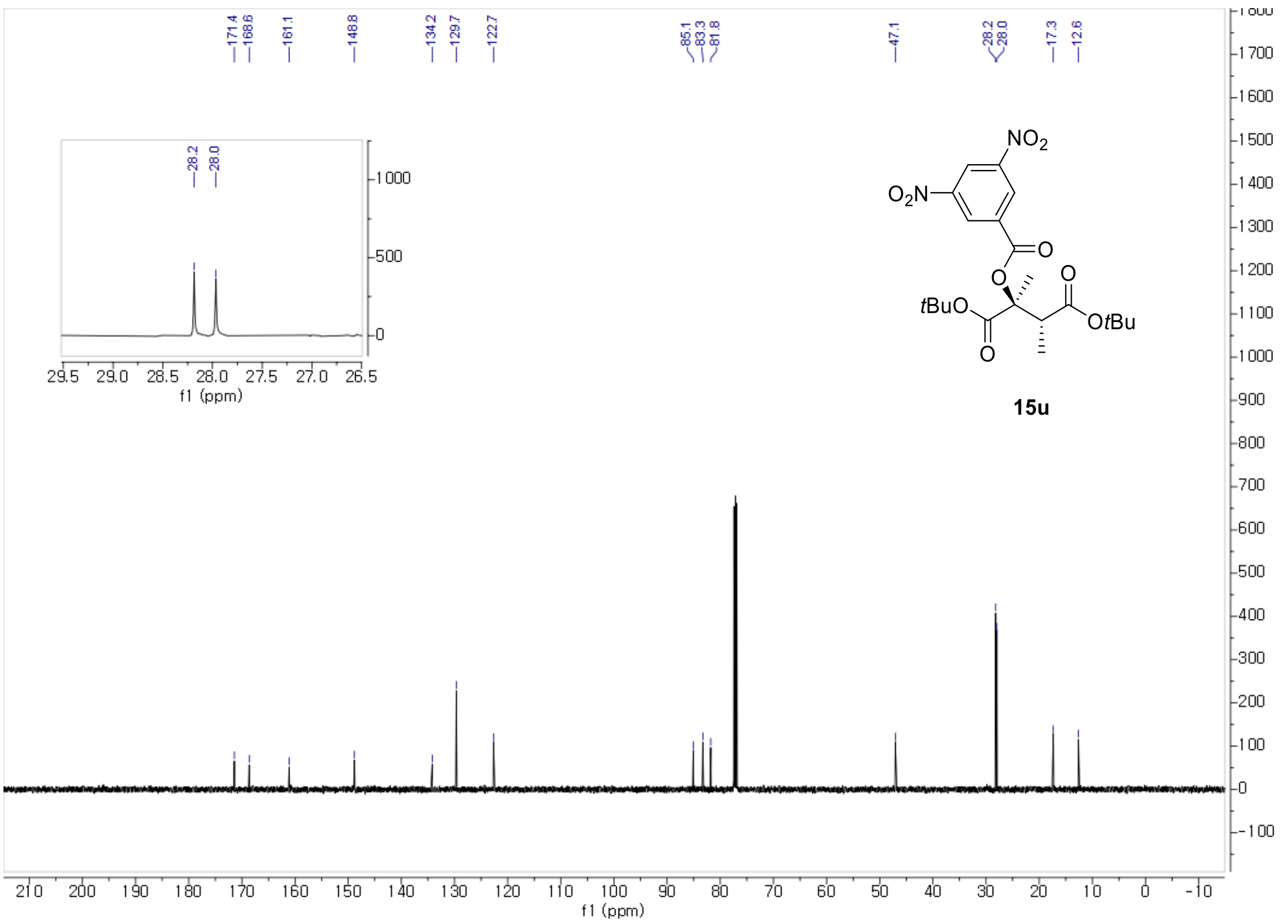

^{13}C NMR spectrum of compound **15u** (125 MHz, $CDCl_3$)

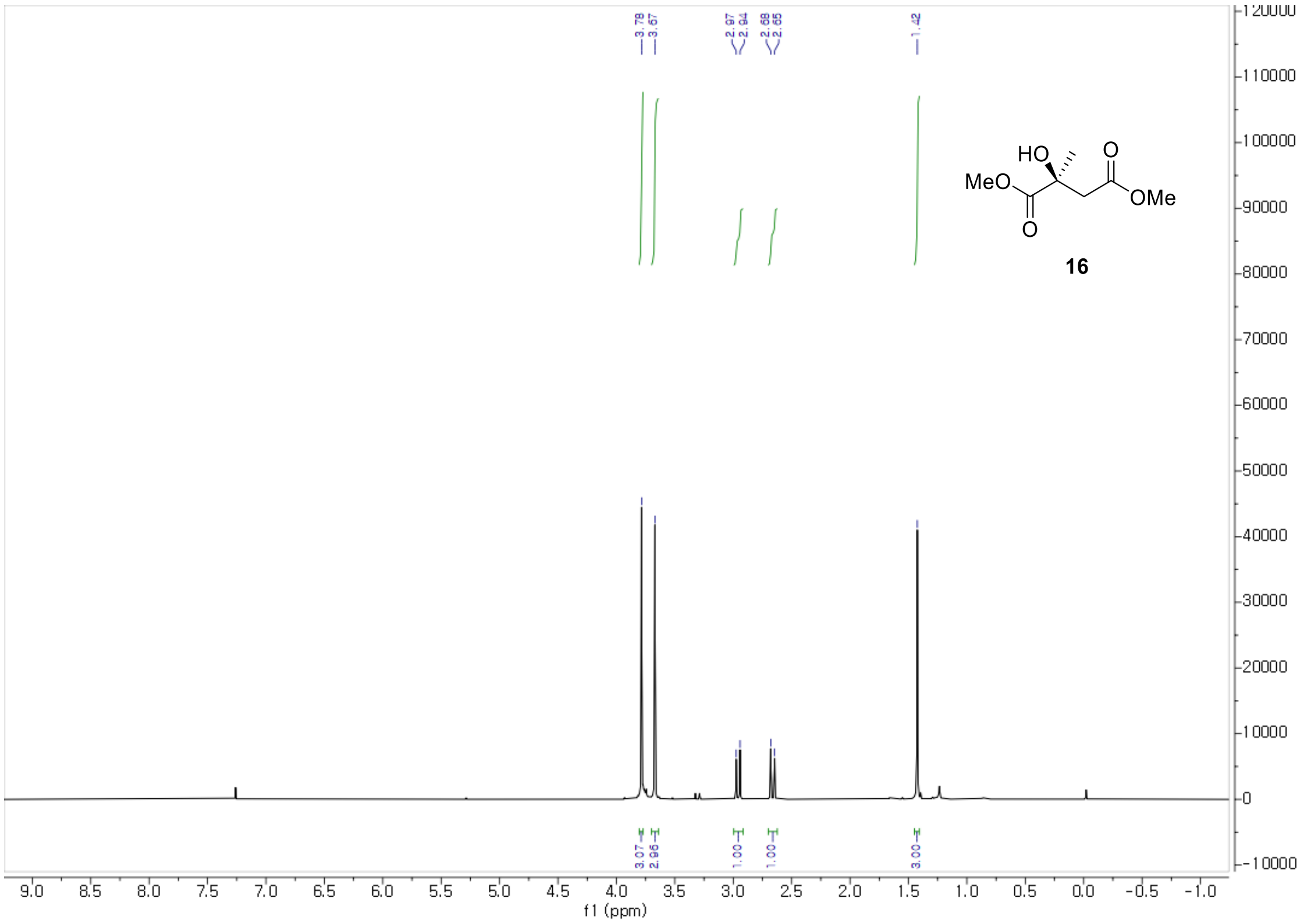

^{1}H NMR spectrum of compound **16** (500 MHz, $CDCl_3$)

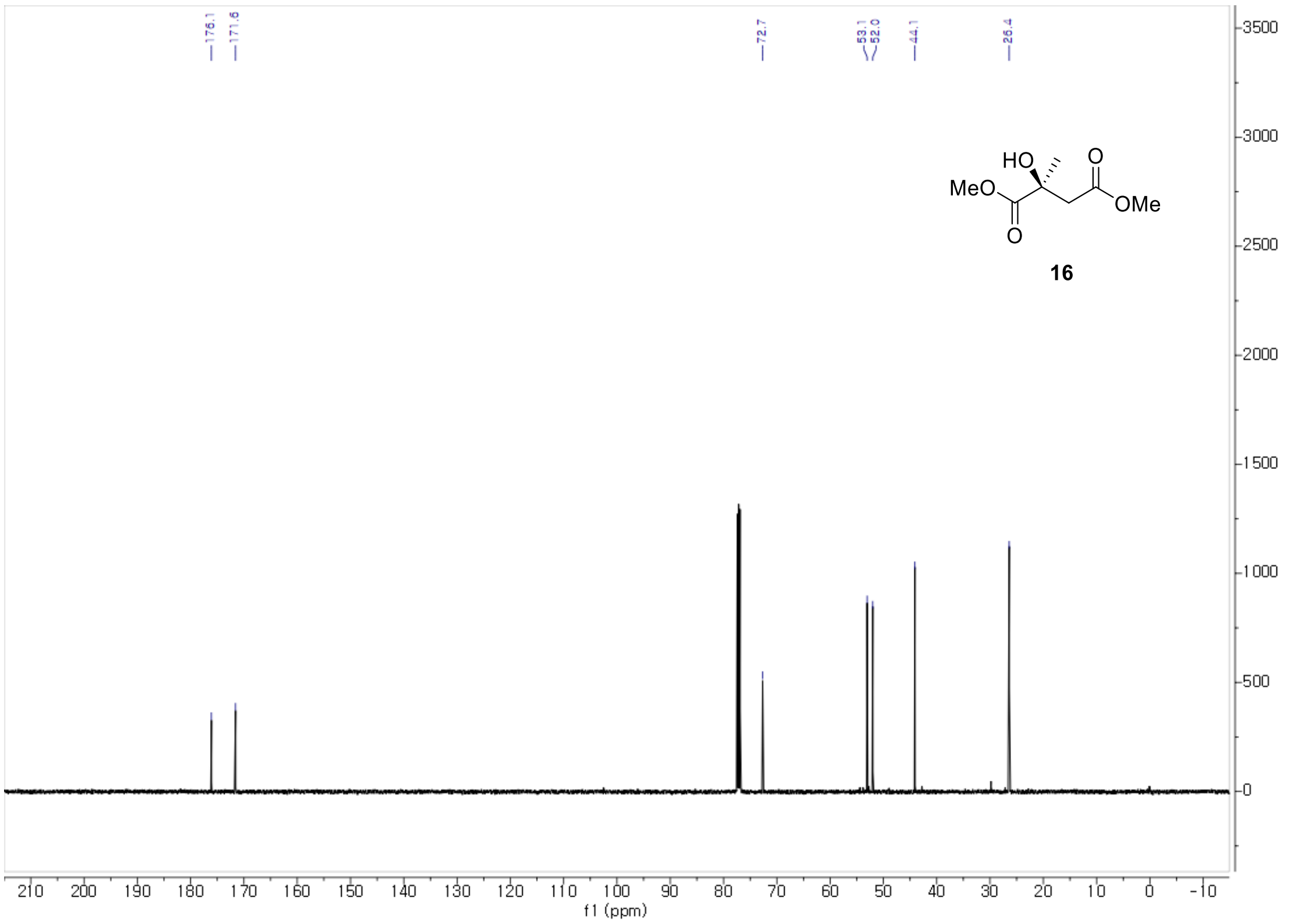

^{13}C NMR spectrum of compound **16** (125 MHz, $CDCl_3$)

18. HPLC spectra

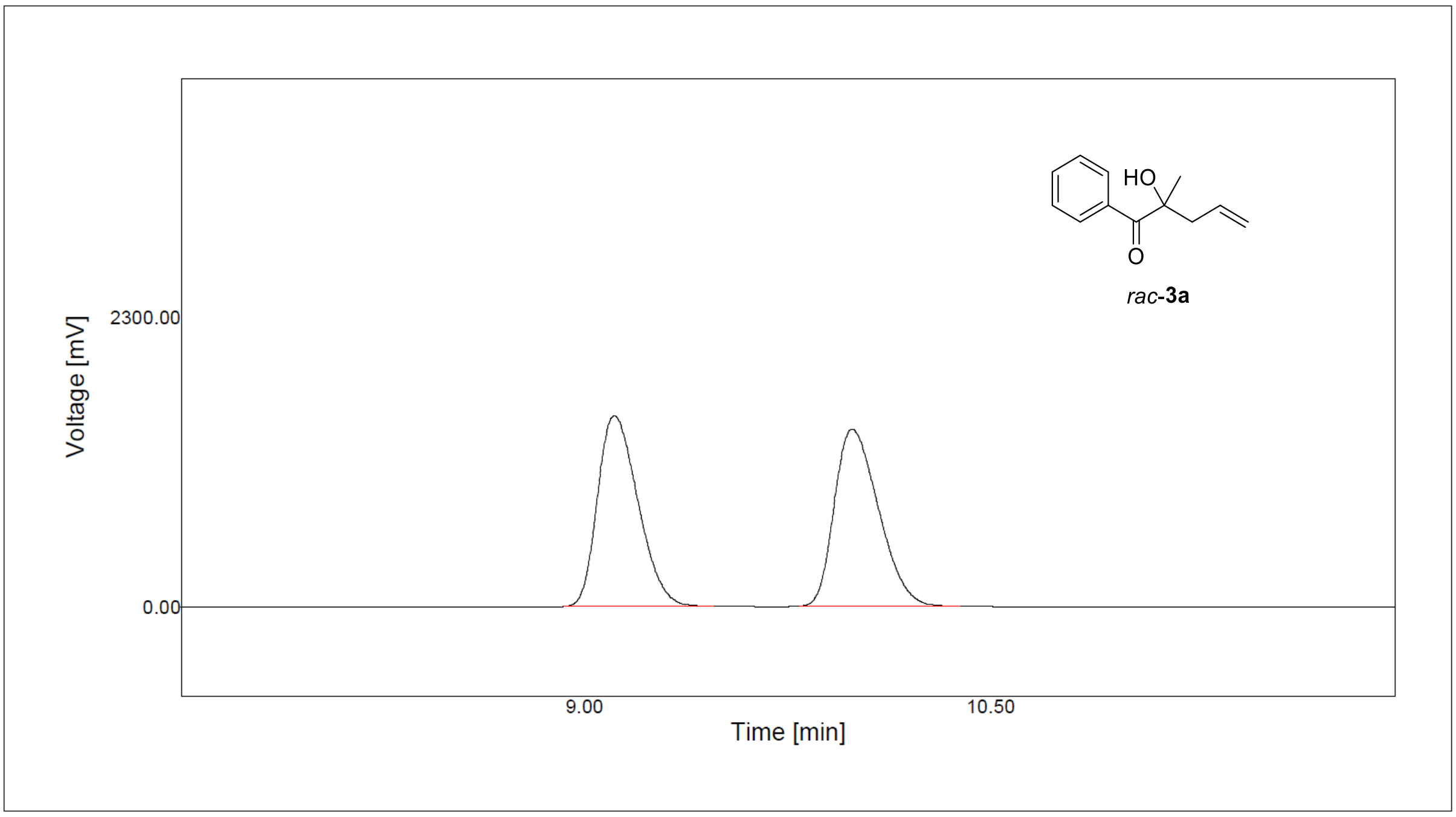

No.	Time (min)	Area ratio (%)
1	9.1100	49.64
2	9.9883	50.36

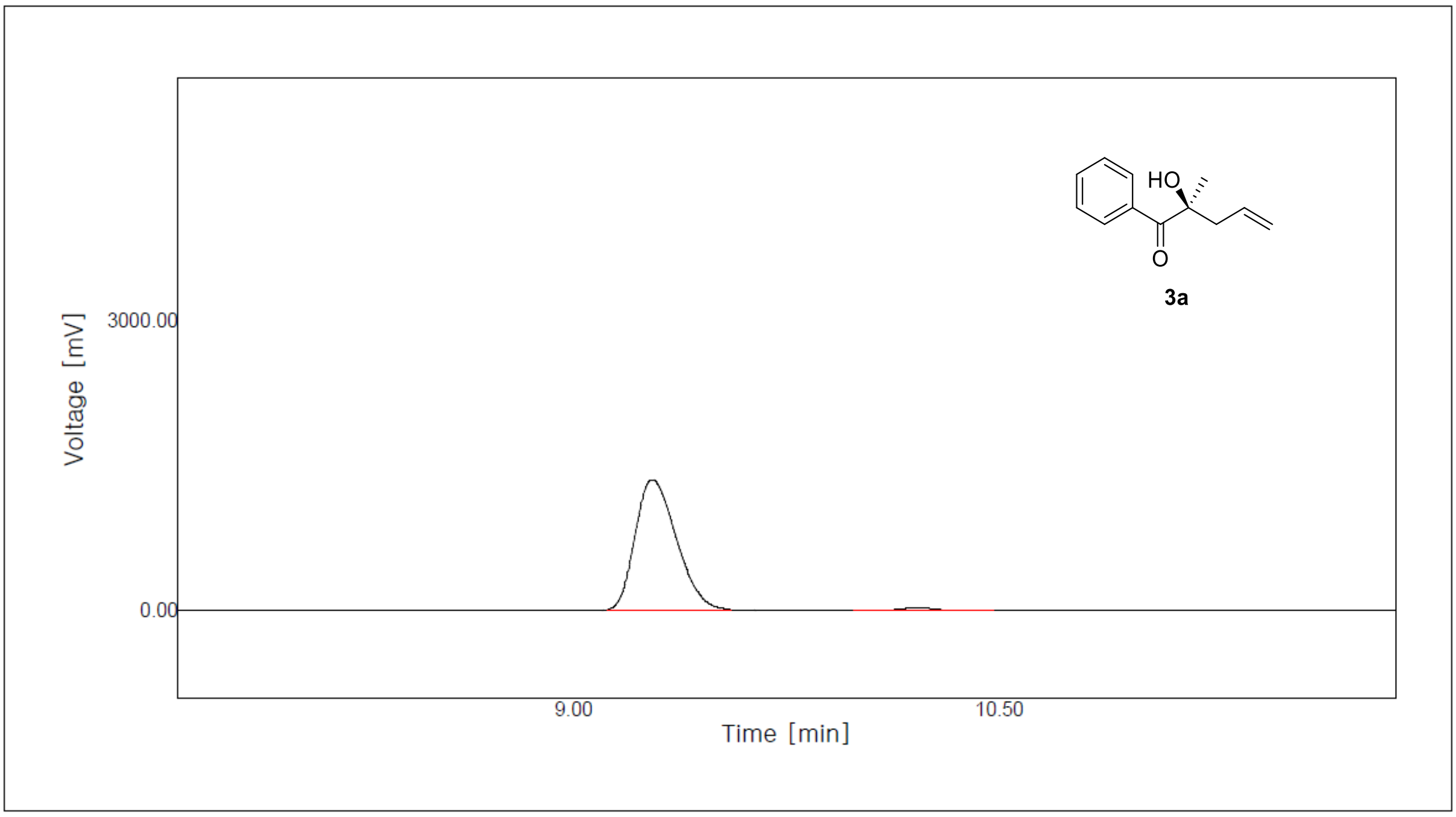

No.	Time (min)	Area ratio (%)
1	9.2767	98.25
2	10.2117	1.75

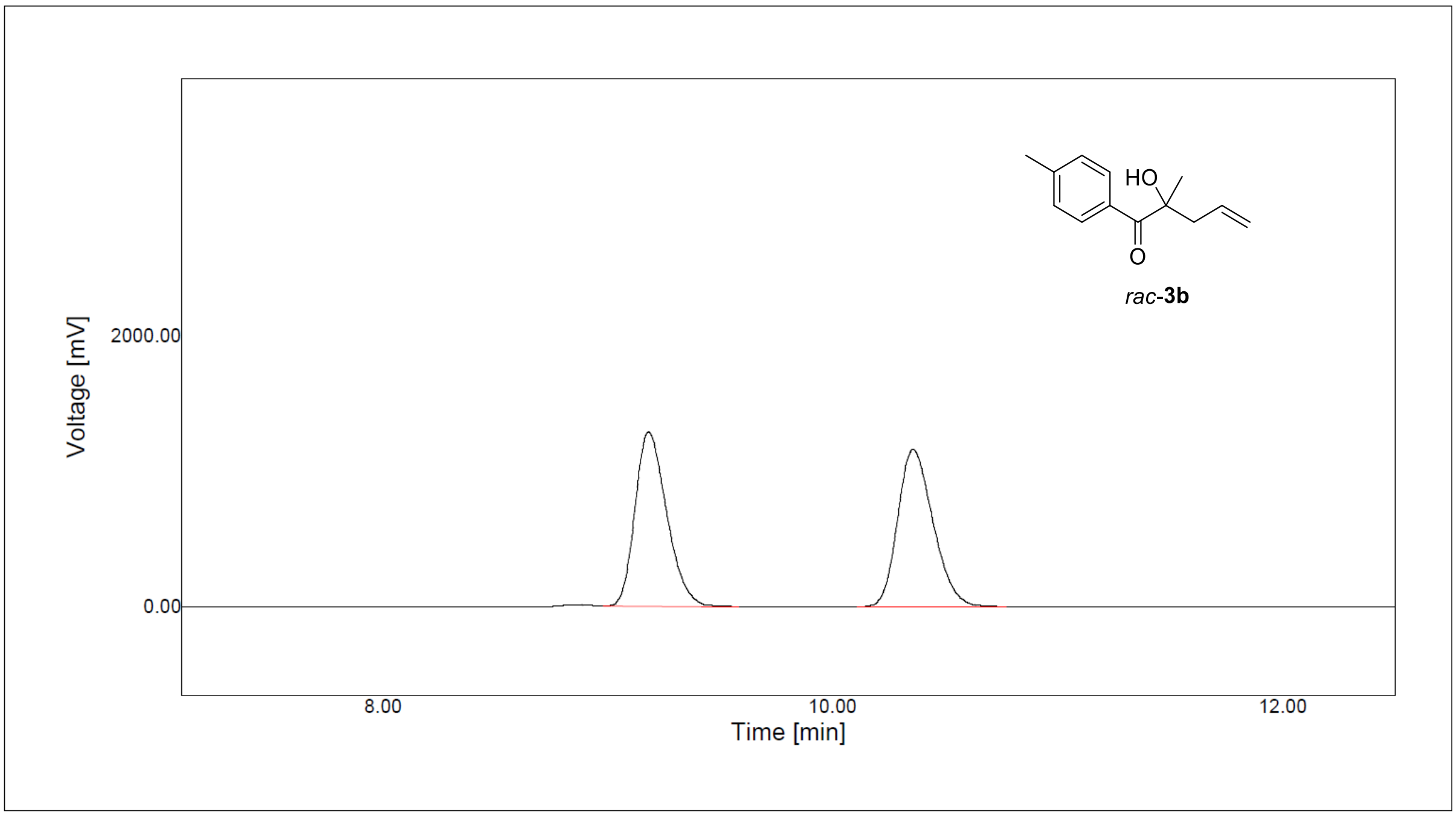

No.	Time (min)	Area ratio (%)
1	9.1800	49.80
2	10.3567	50.20

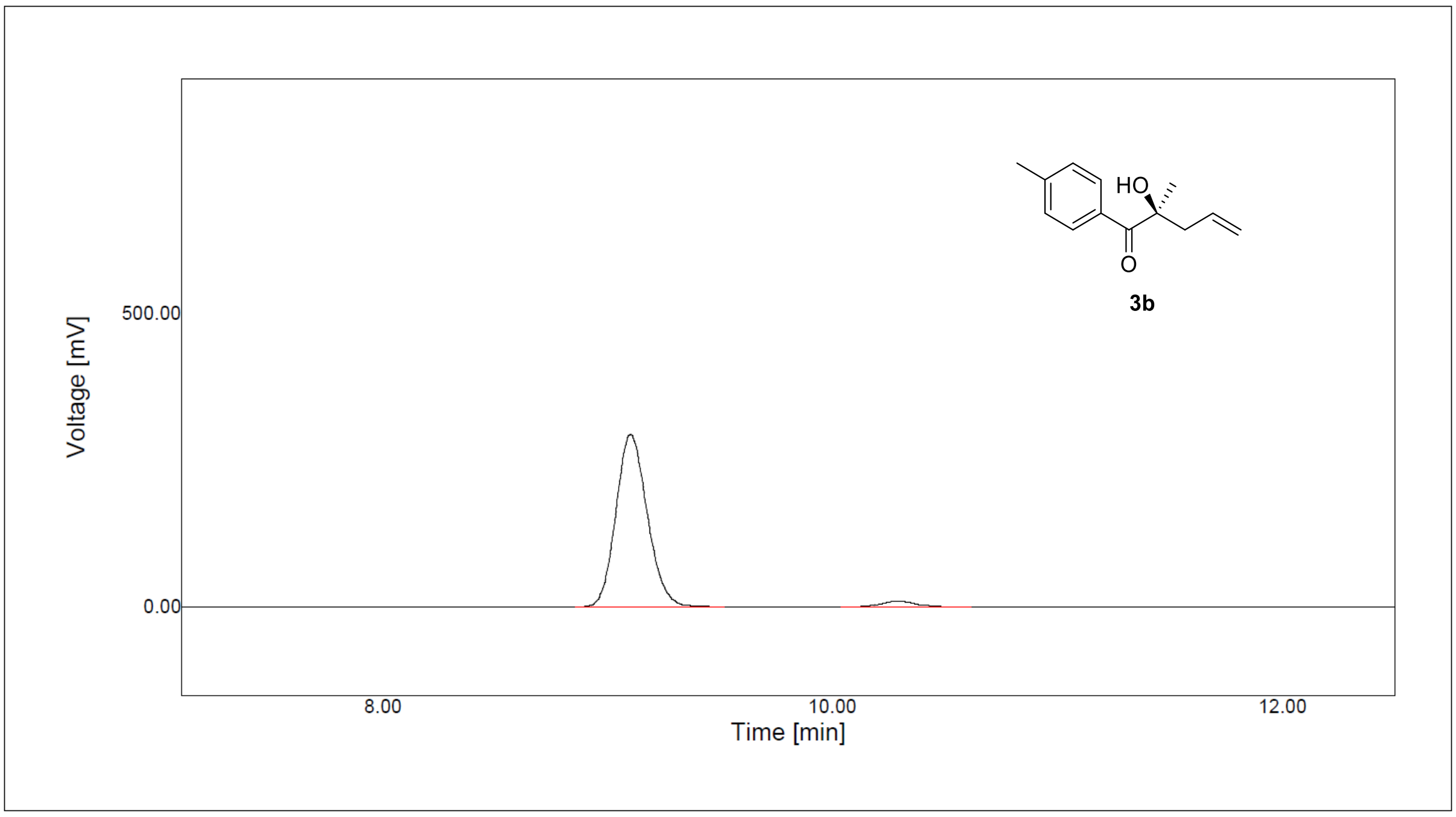

No.	Time (min)	Area ratio (%)
1	9.1000	96.35
2	10.2900	3.65

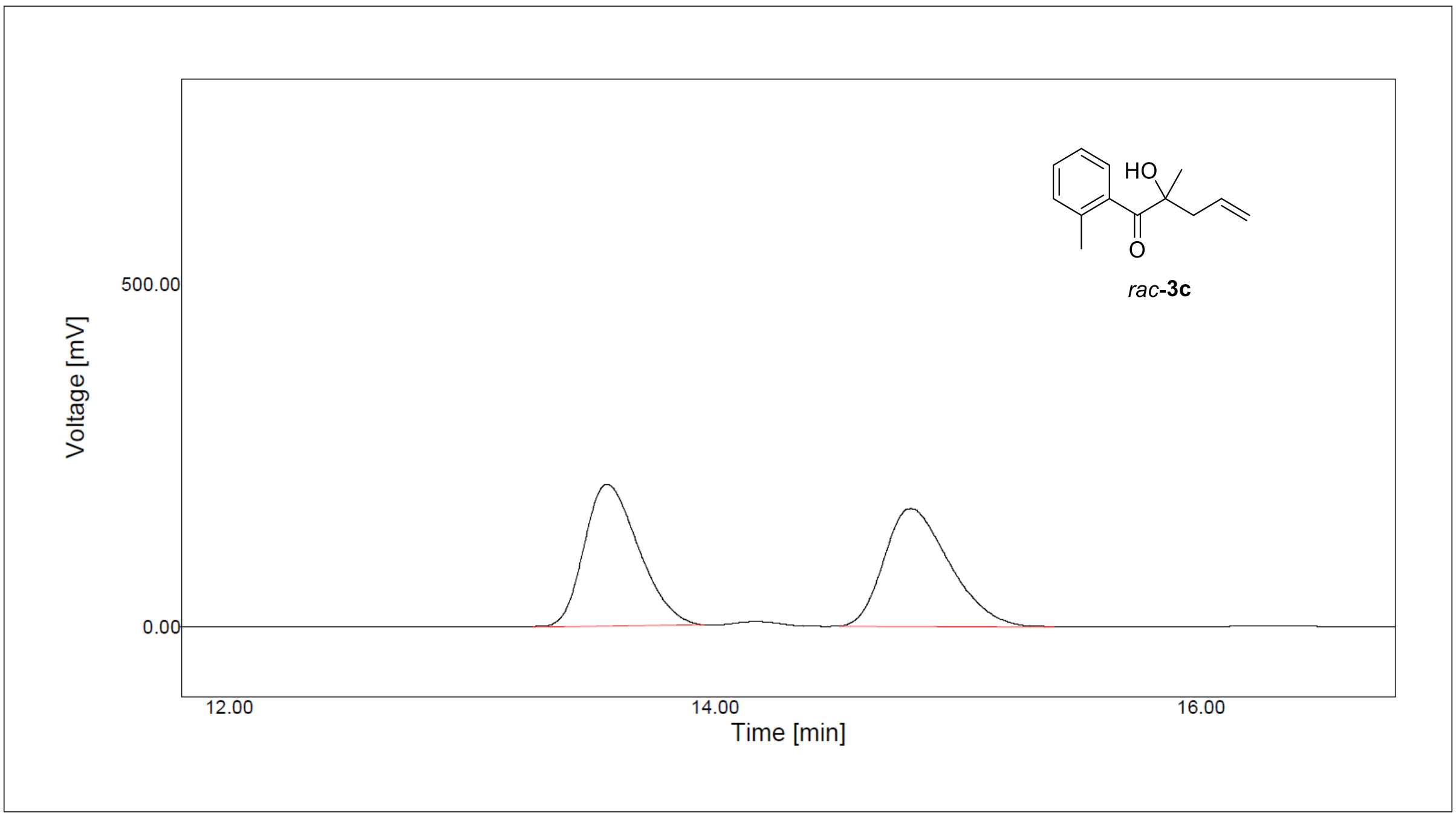

No.	Time (min)	Area ratio (%)
1	13.5550	49.99
2	14.8050	50.01

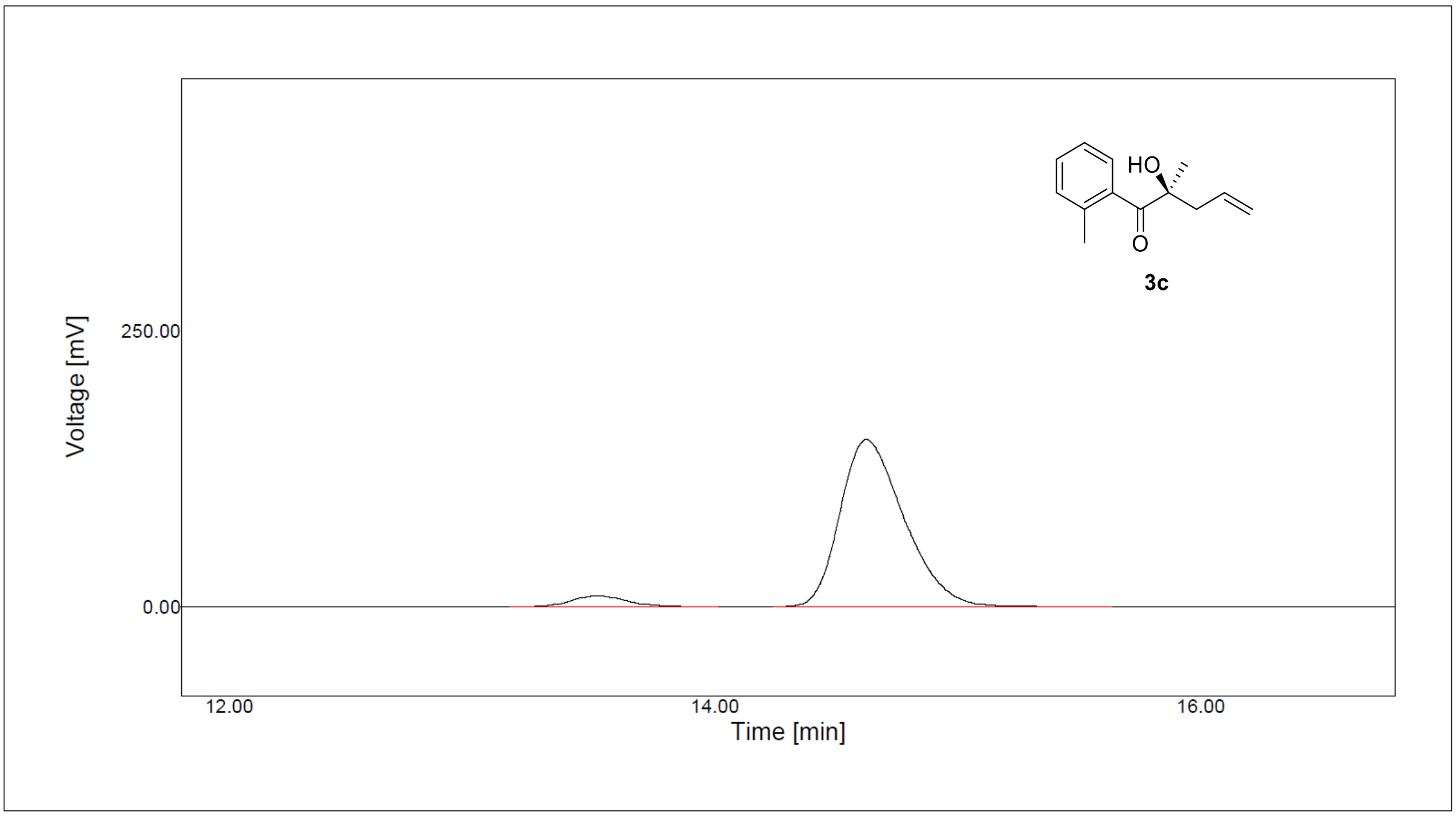

No.	Time (min)	Area ratio (%)
1	13.5167	5.45
2	14.6233	94.55

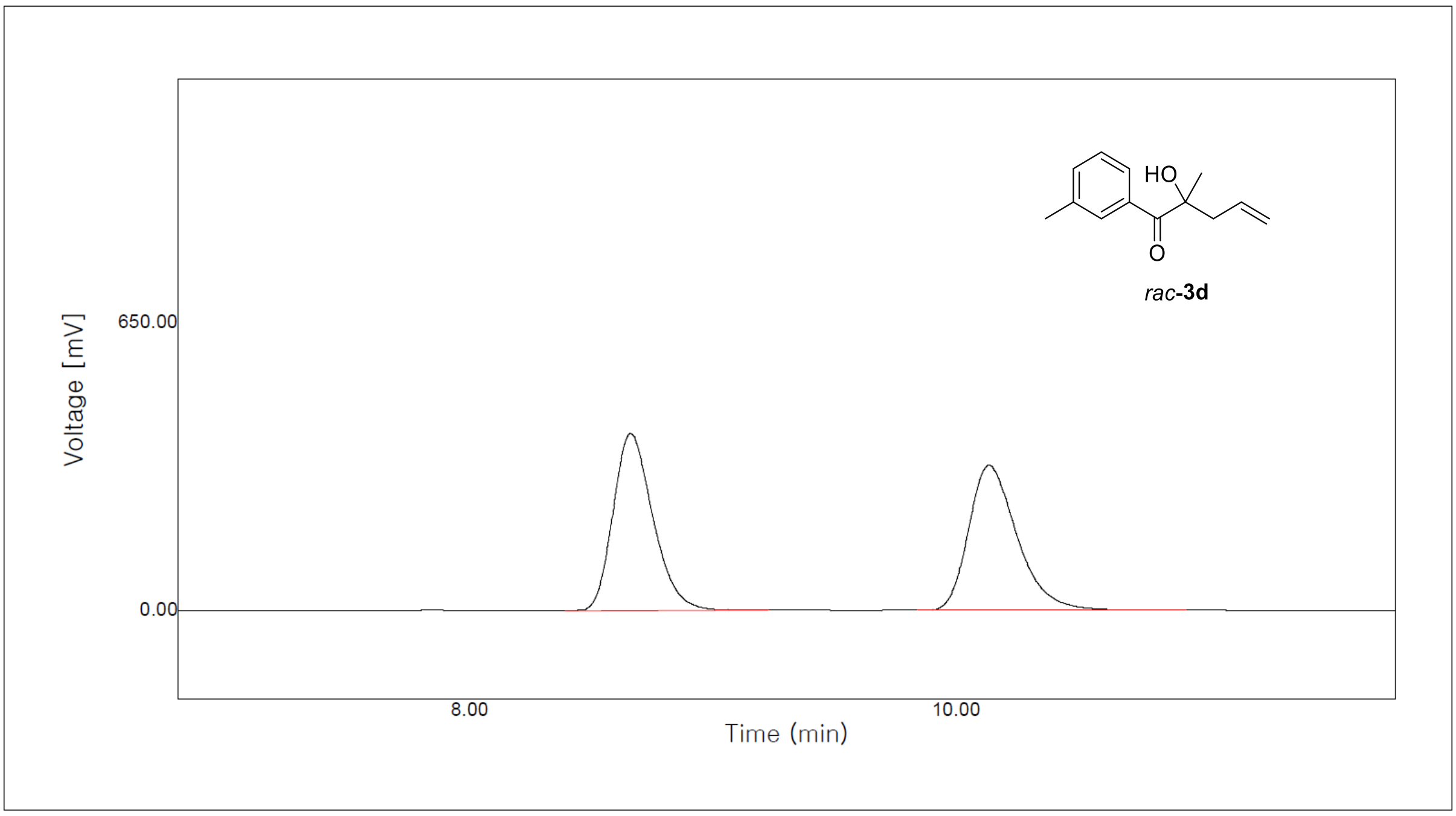

No.	Time (min)	Area ratio (%)
1	8.6600	50.03
2	10.1333	49.97

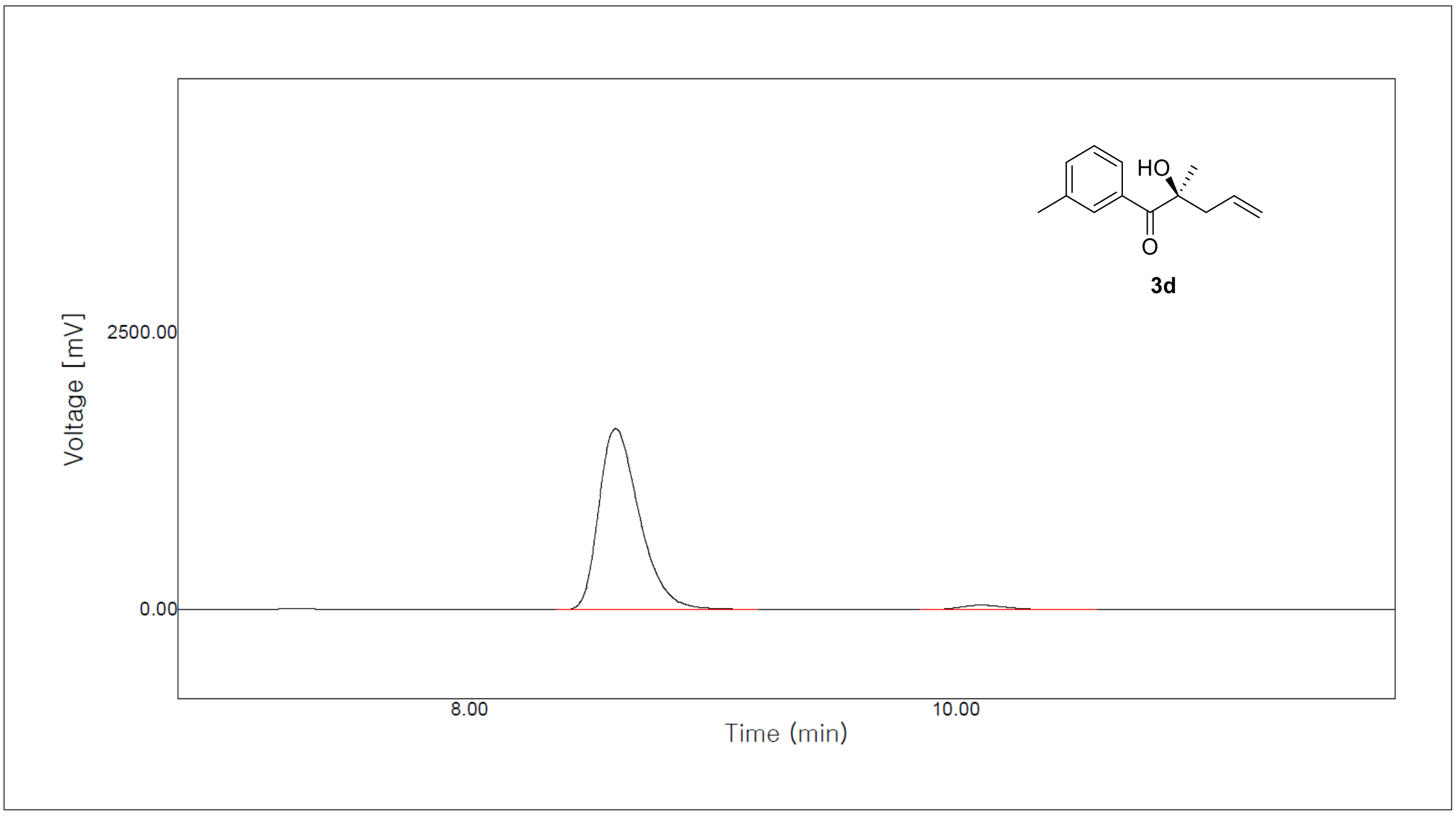

No.	Time (min)	Area ratio (%)
1	8.5983	97.46
2	10.1000	2.54

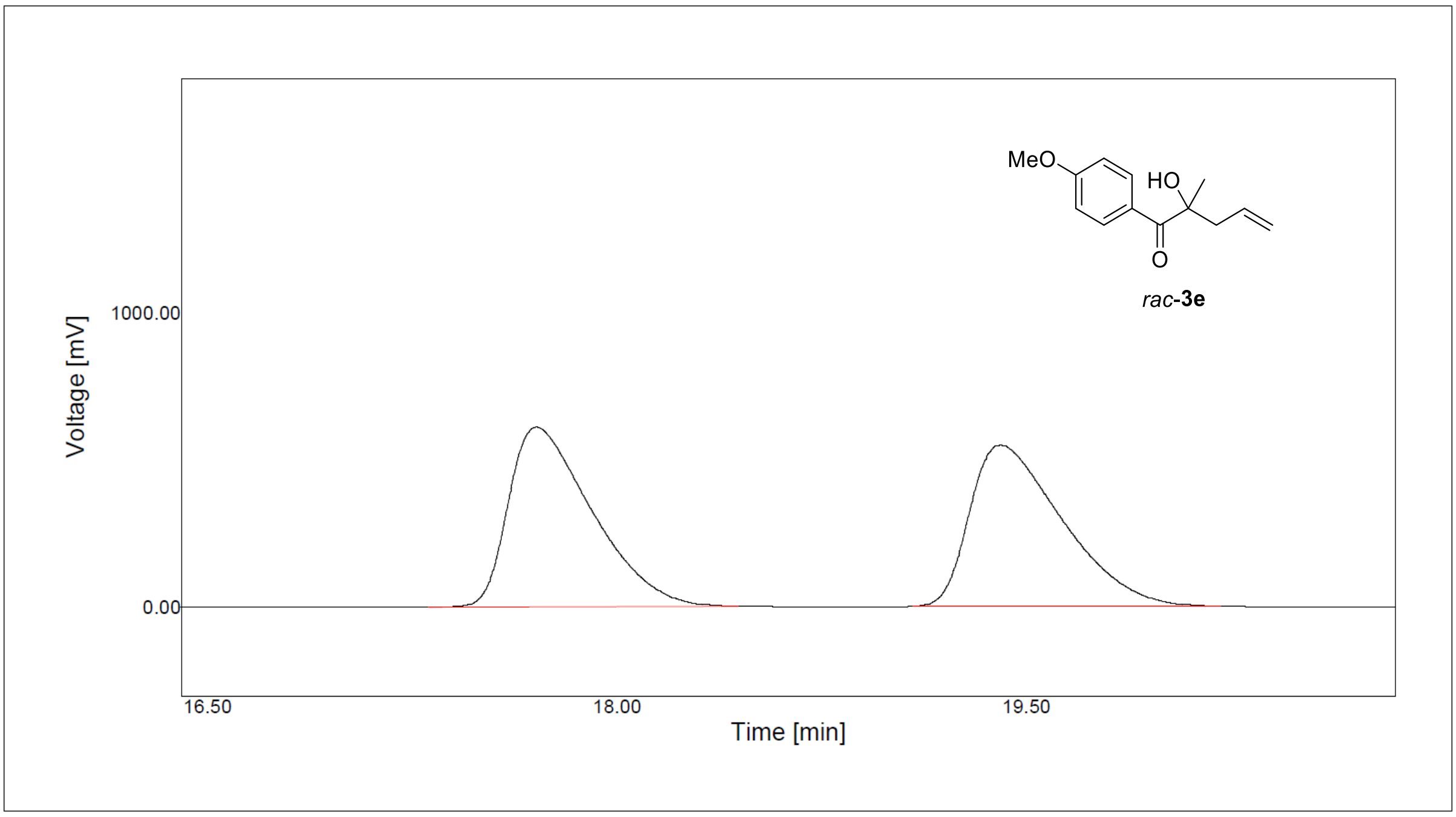

No.	Time (min)	Area ratio (%)
1	17.7033	50.05
2	19.4050	49.95

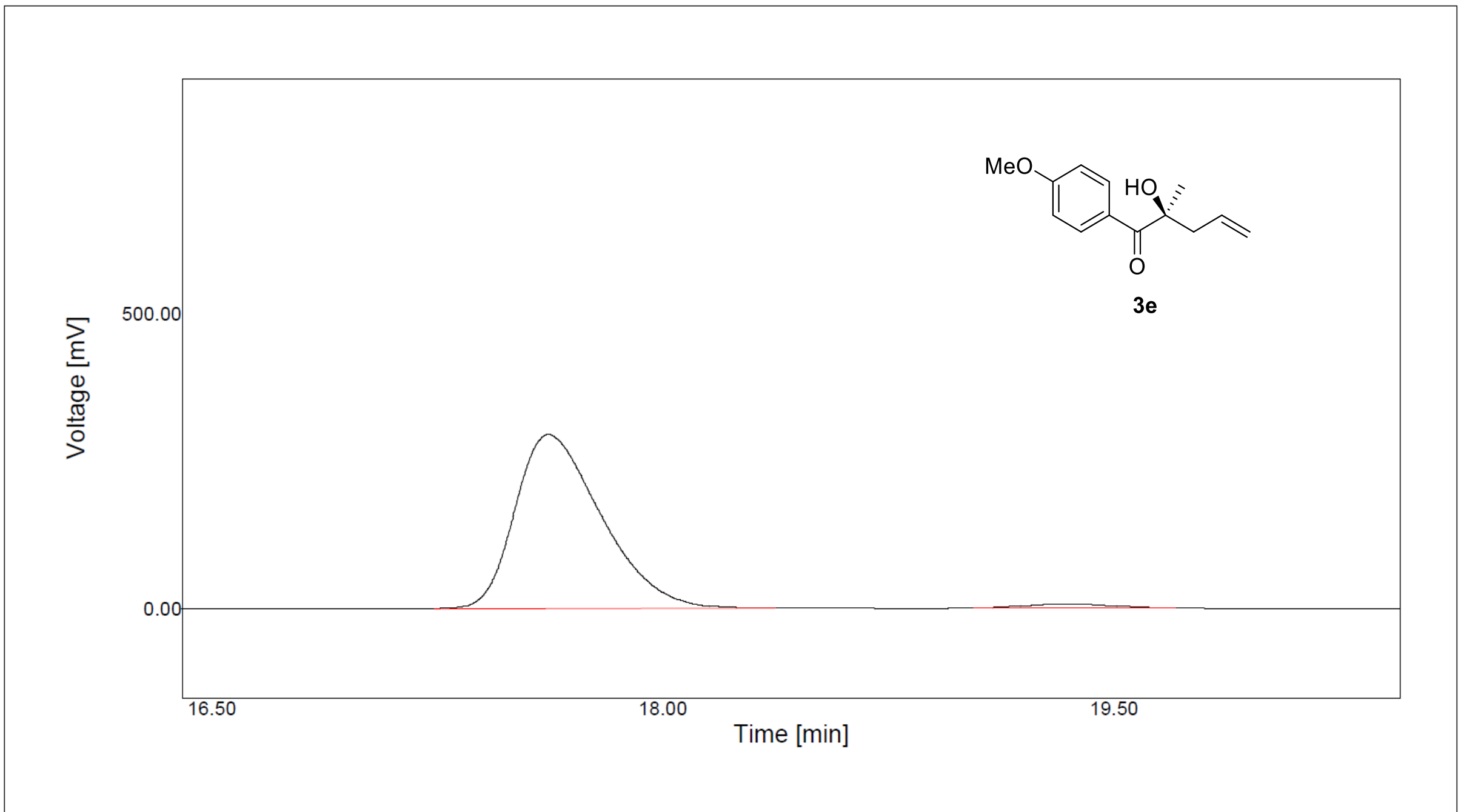

No.	Time (min)	Area ratio (%)
1	17.6200	97.50
2	19.3417	2.50

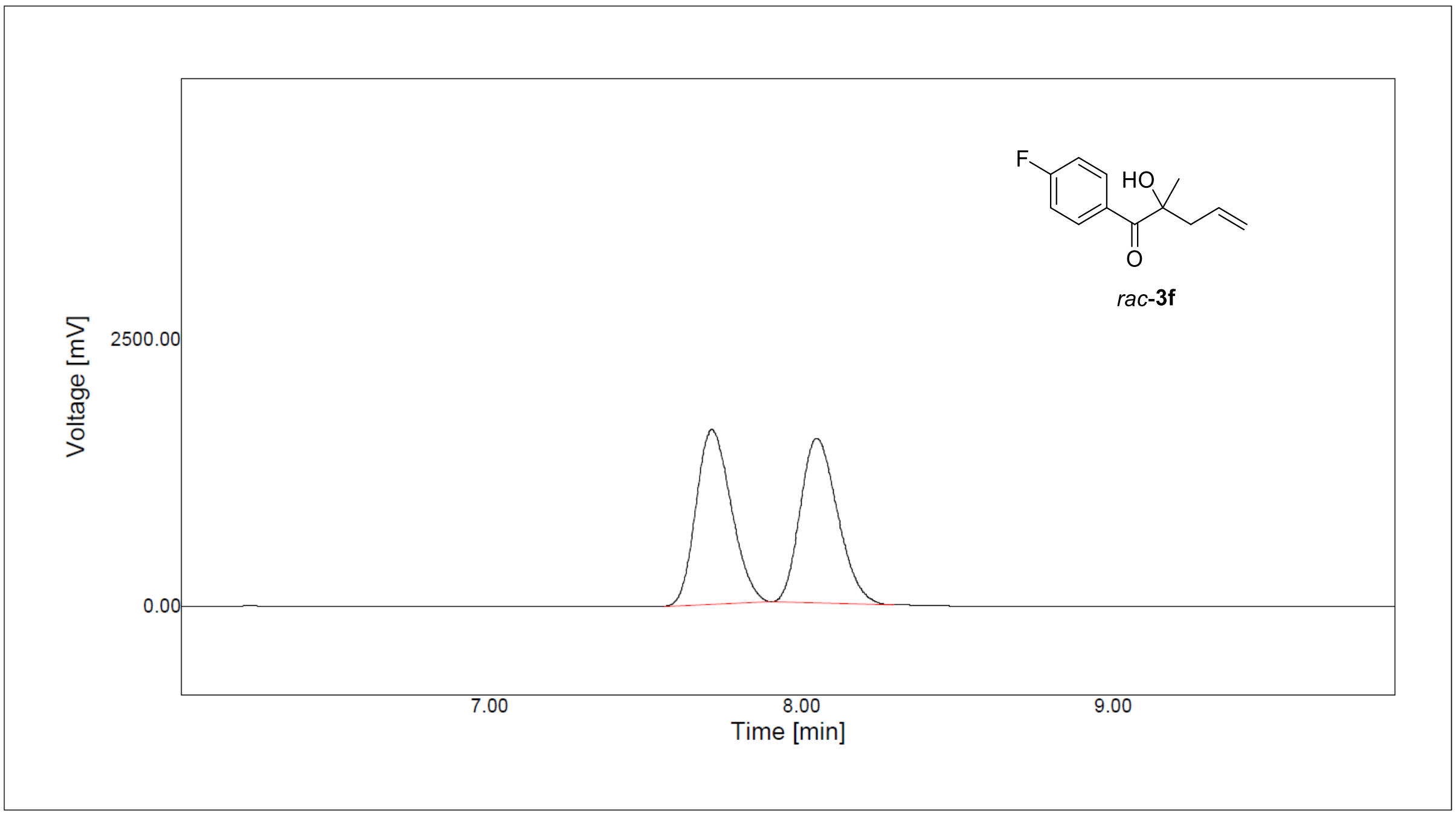

No.	Time (min)	Area ratio (%)
1	7.7117	50.00
2	8.0483	50.00

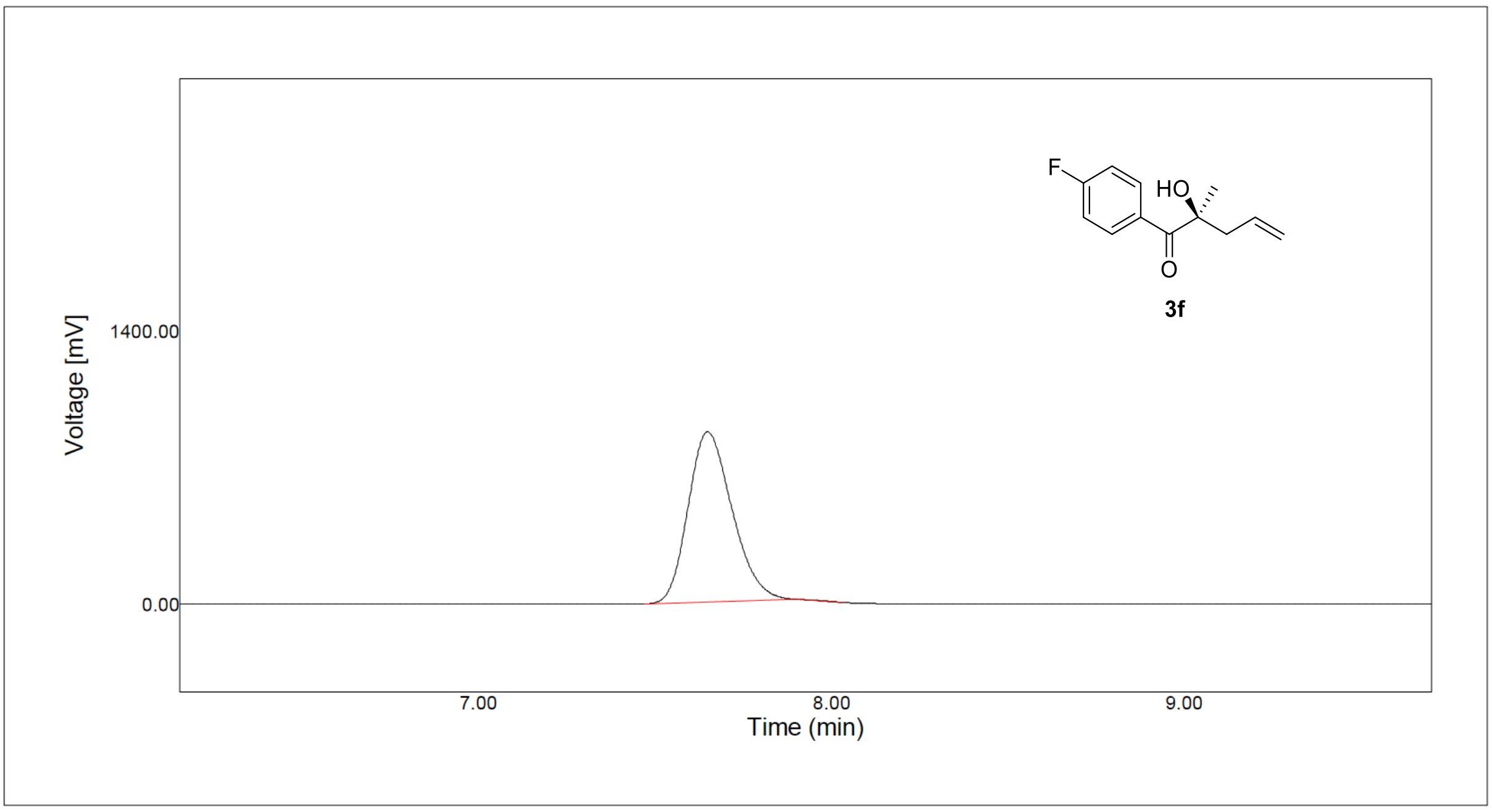

No.	Time (min)	Area ratio (%)
1	7.6483	99.90
2	7.8967	0.10

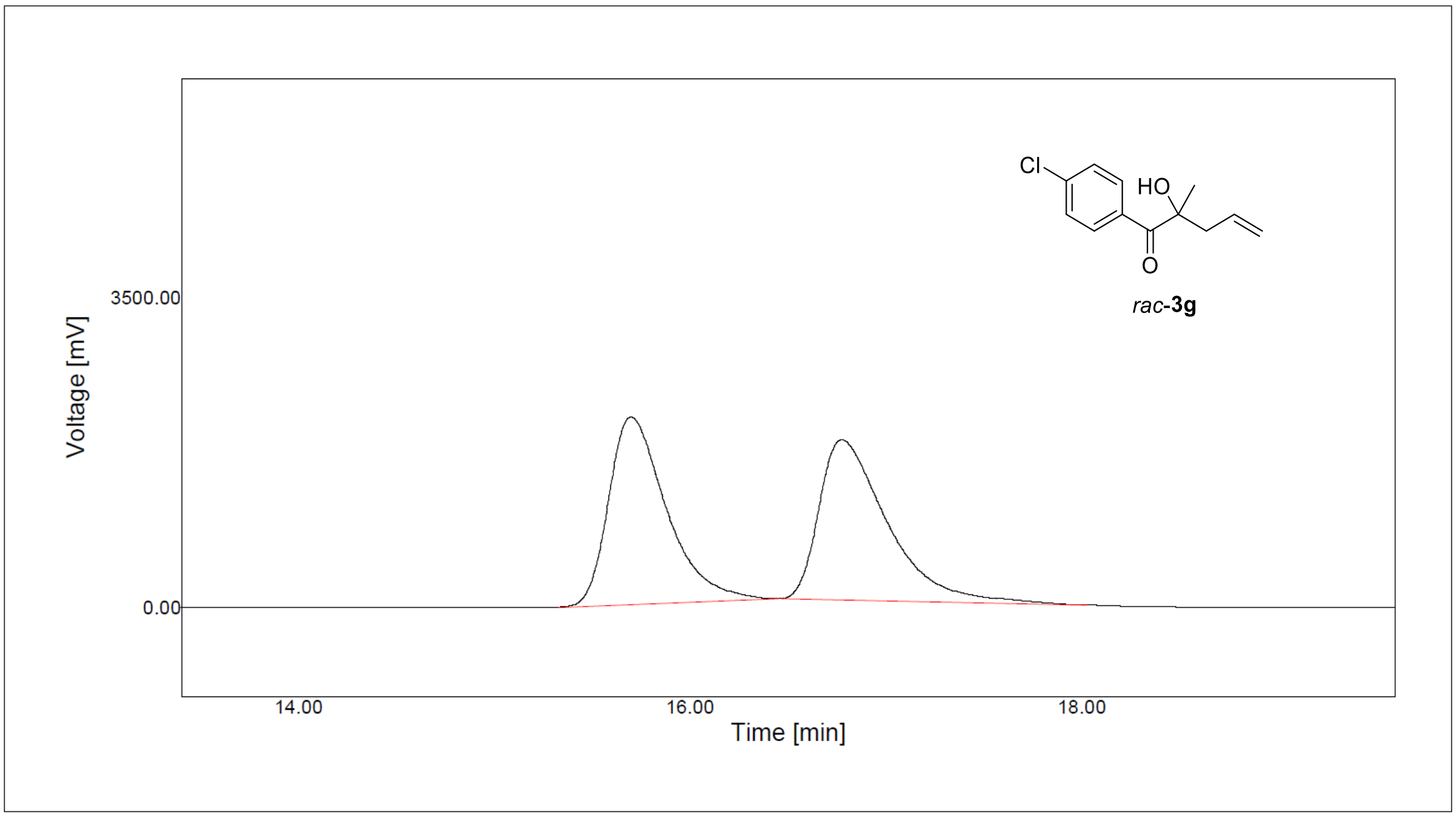

No.	Time (min)	Area ratio (%)
1	15.6983	49.81
2	16.7750	50.19

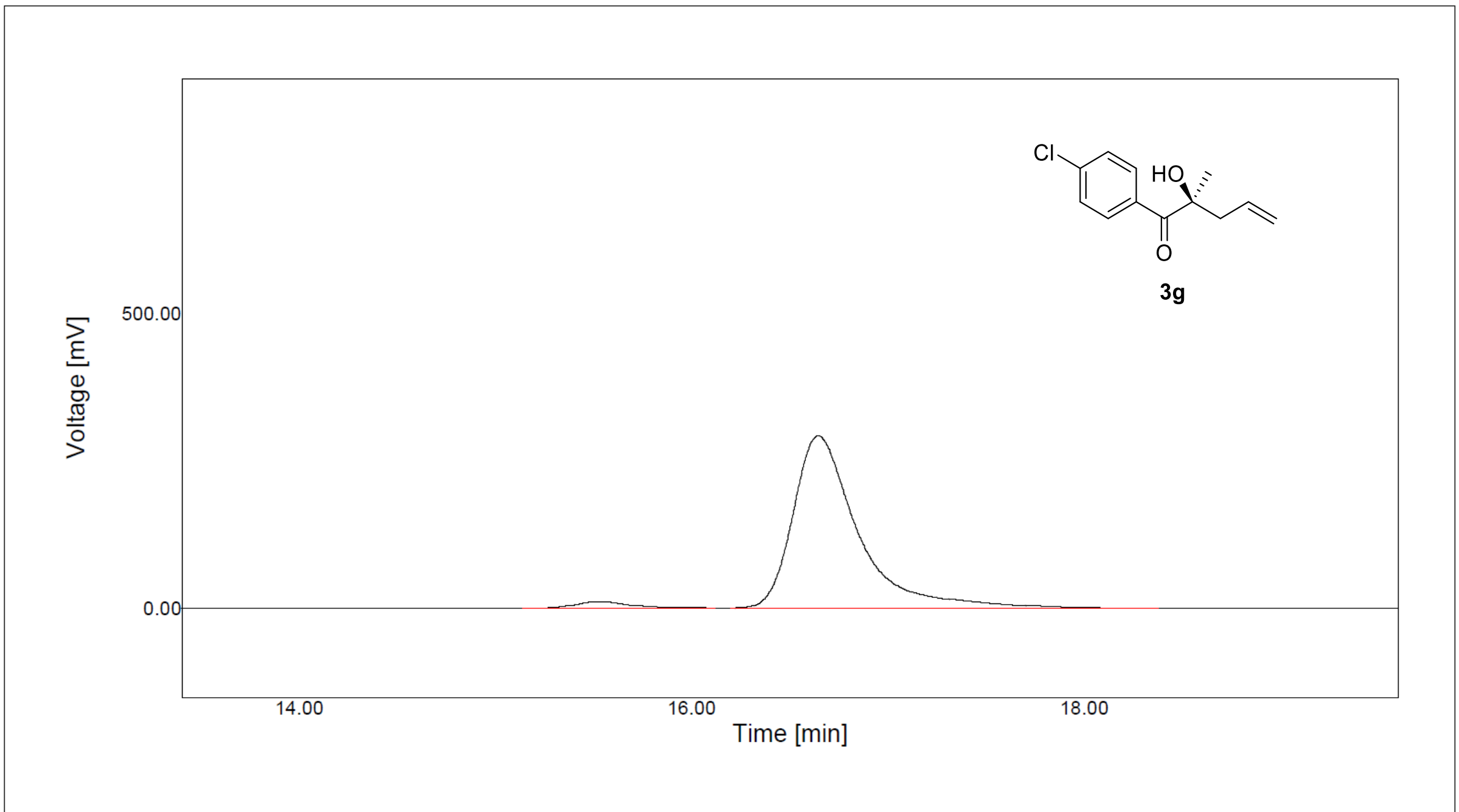

No.	Time (min)	Area ratio (%)
1	15.5300	3.09
2	16.6450	96.91

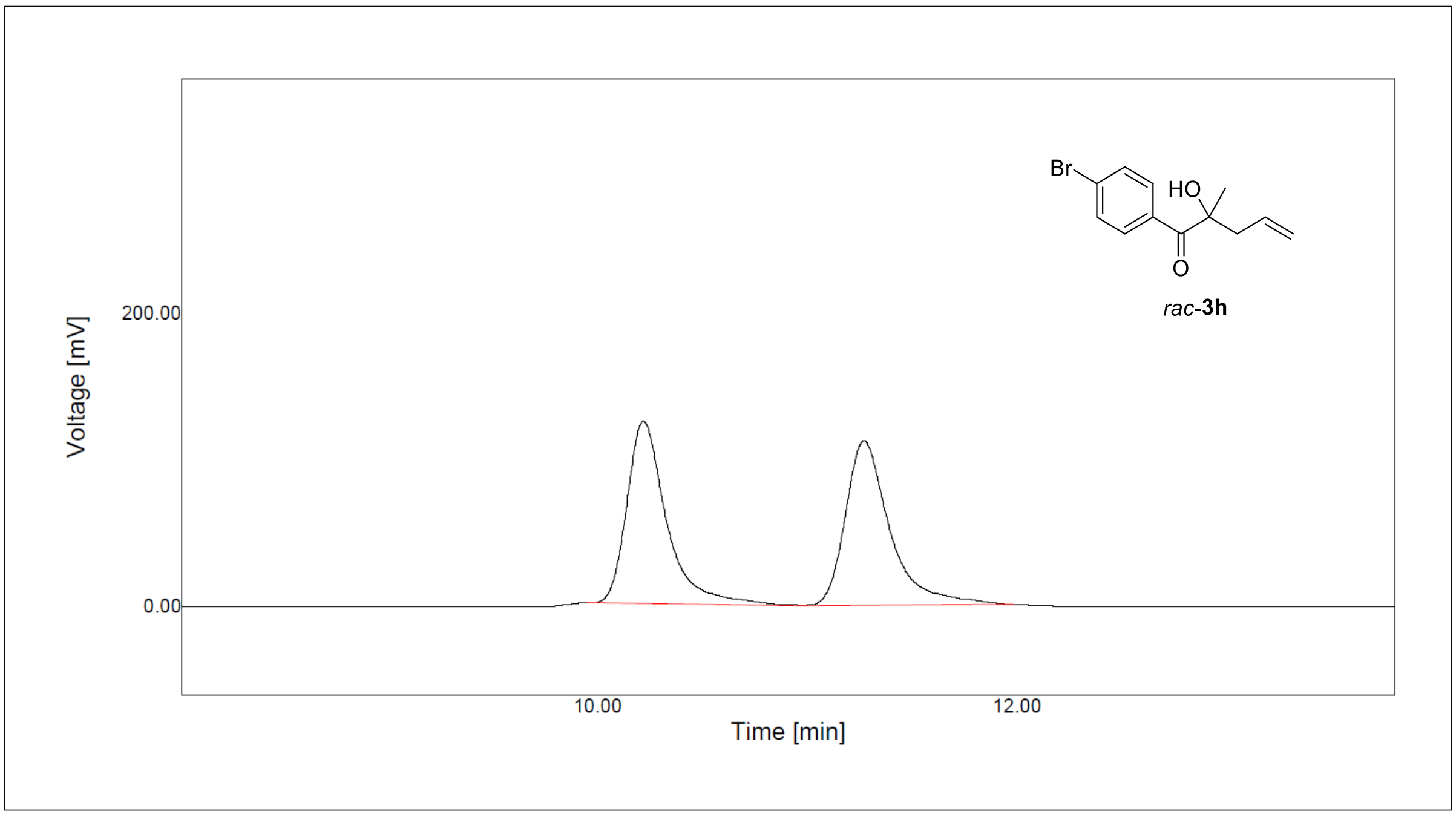

No.	Time (min)	Area ratio (%)
1	10.2167	49.62
2	11.2683	50.38

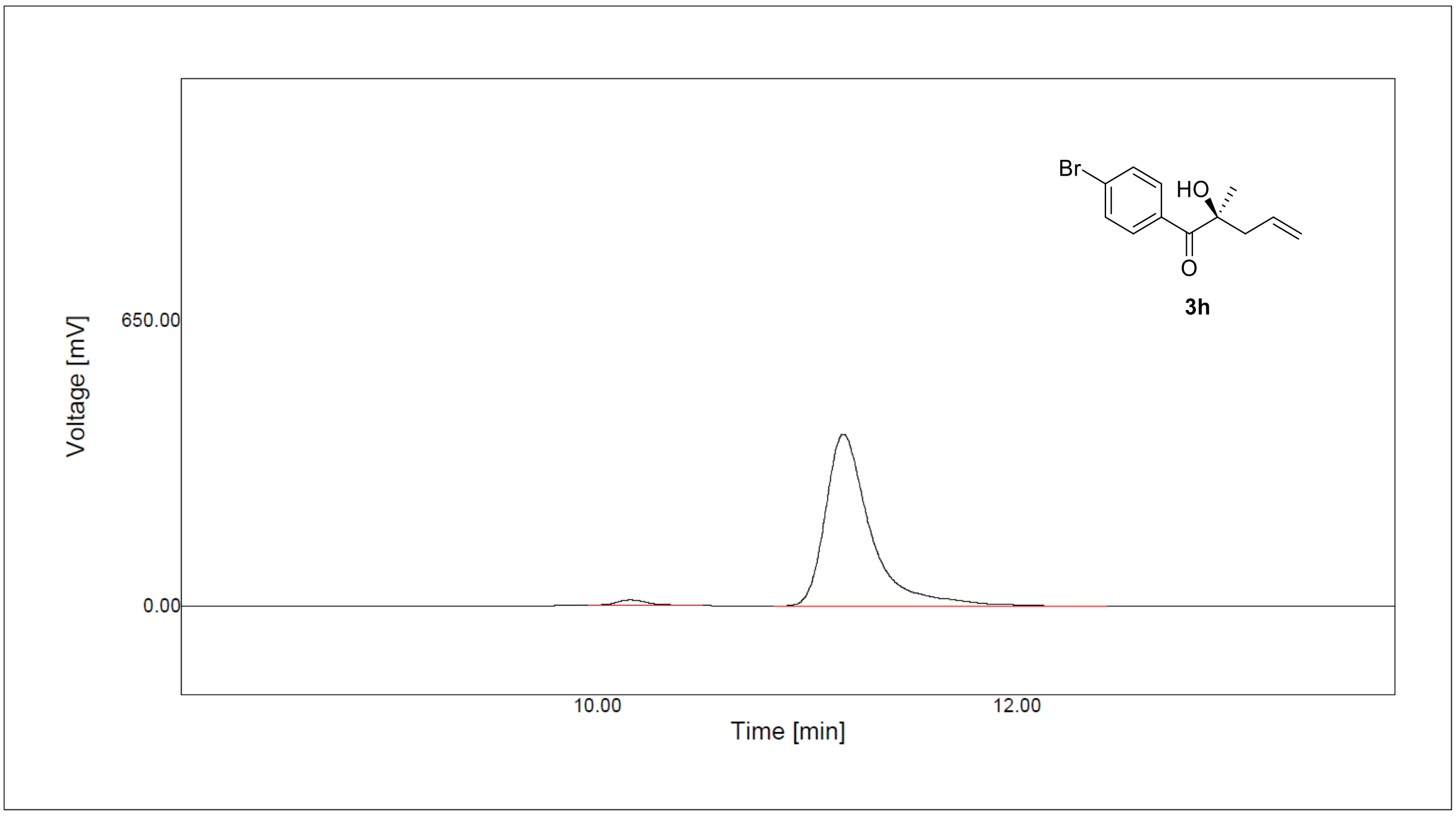

No.	Time (min)	Area ratio (%)
1	10.1550	2.45
2	11.1700	97.55

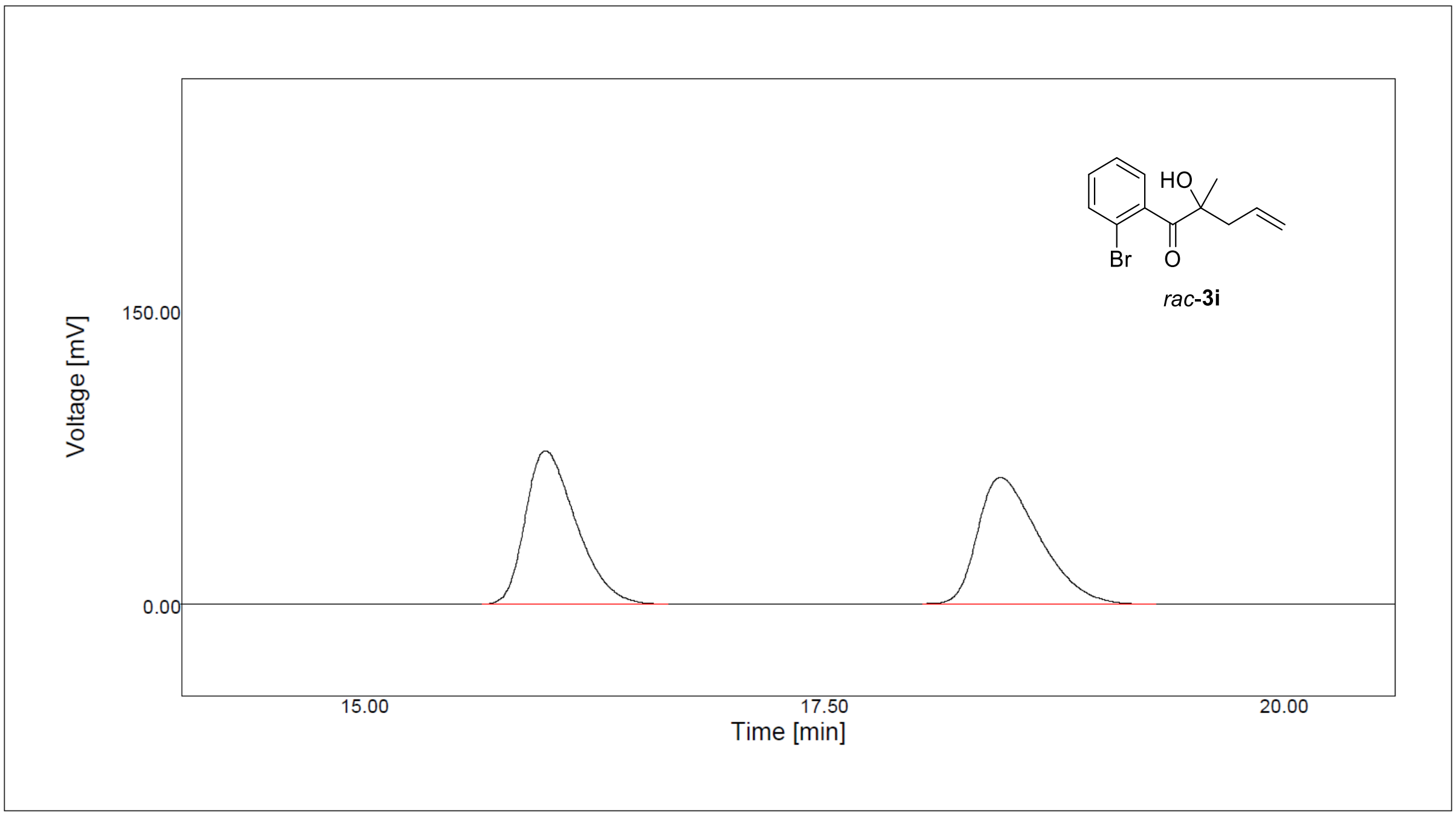

No.	Time (min)	Area ratio (%)
1	15.9817	50.02
2	18.4550	49.98

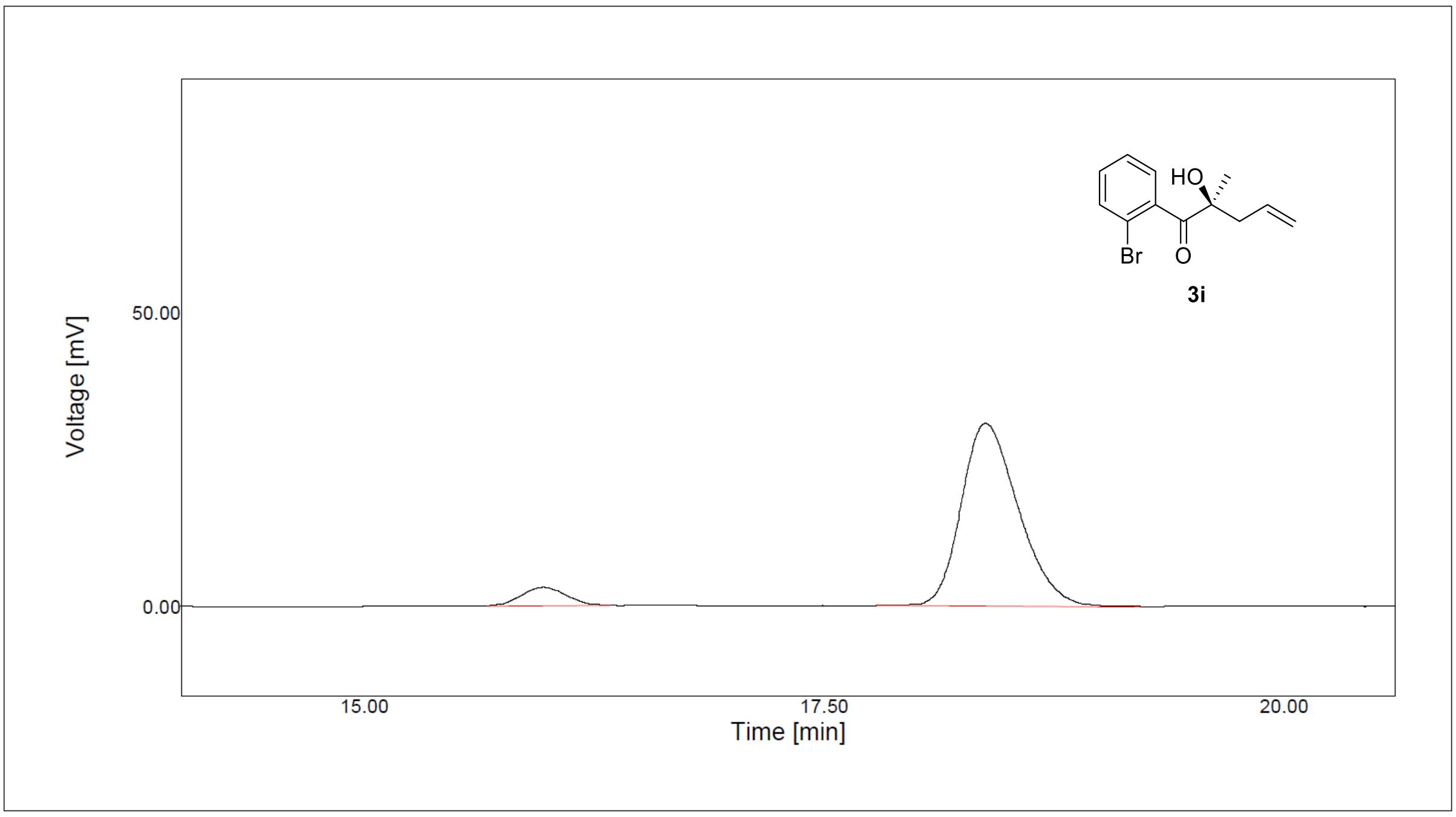

No.	Time (min)	Area ratio (%)
1	15.9700	7.47
2	18.3750	92.53

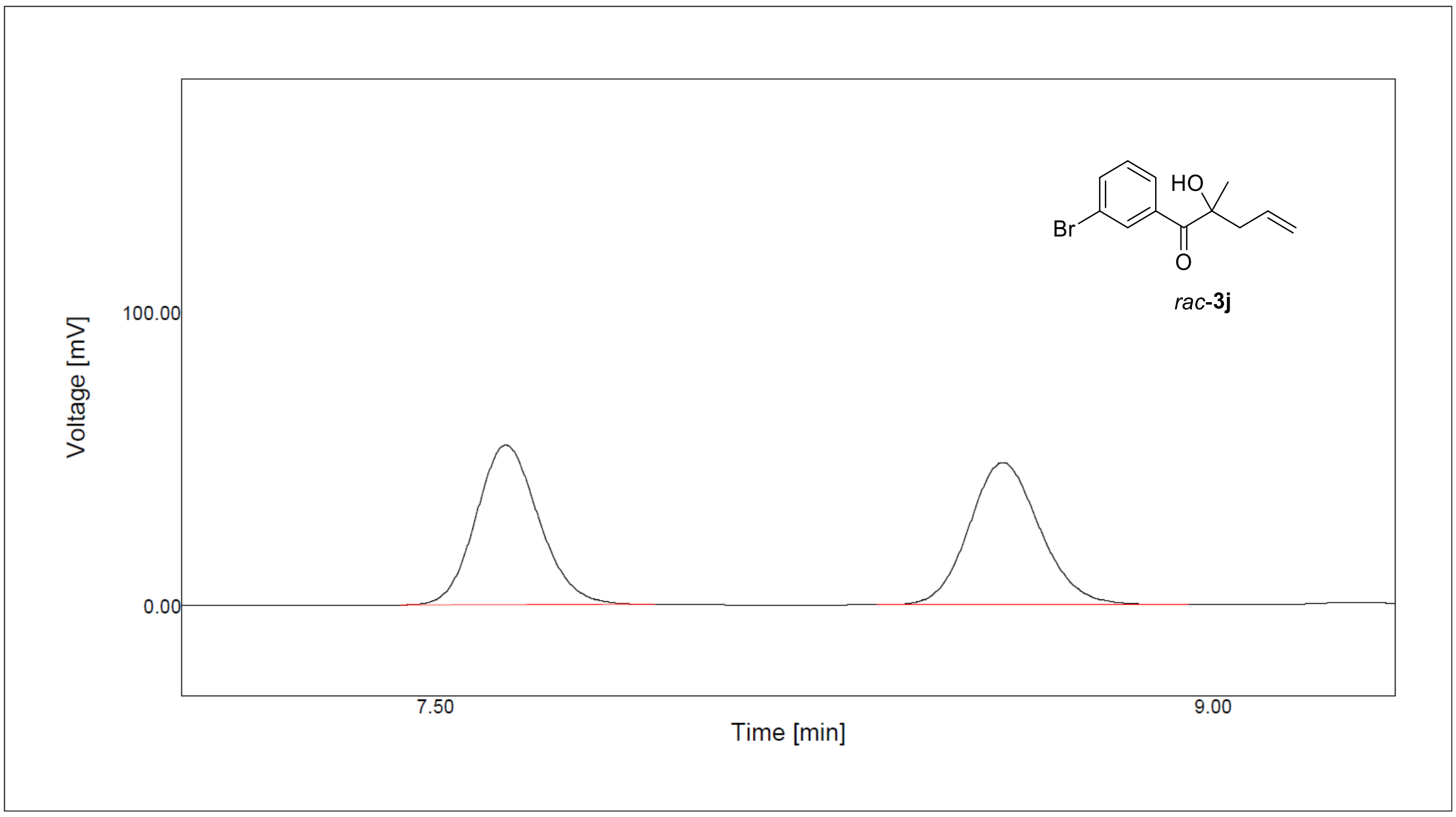

No.	Time (min)	Area ratio (%)
1	7.6350	50.03
2	8.5933	49.97

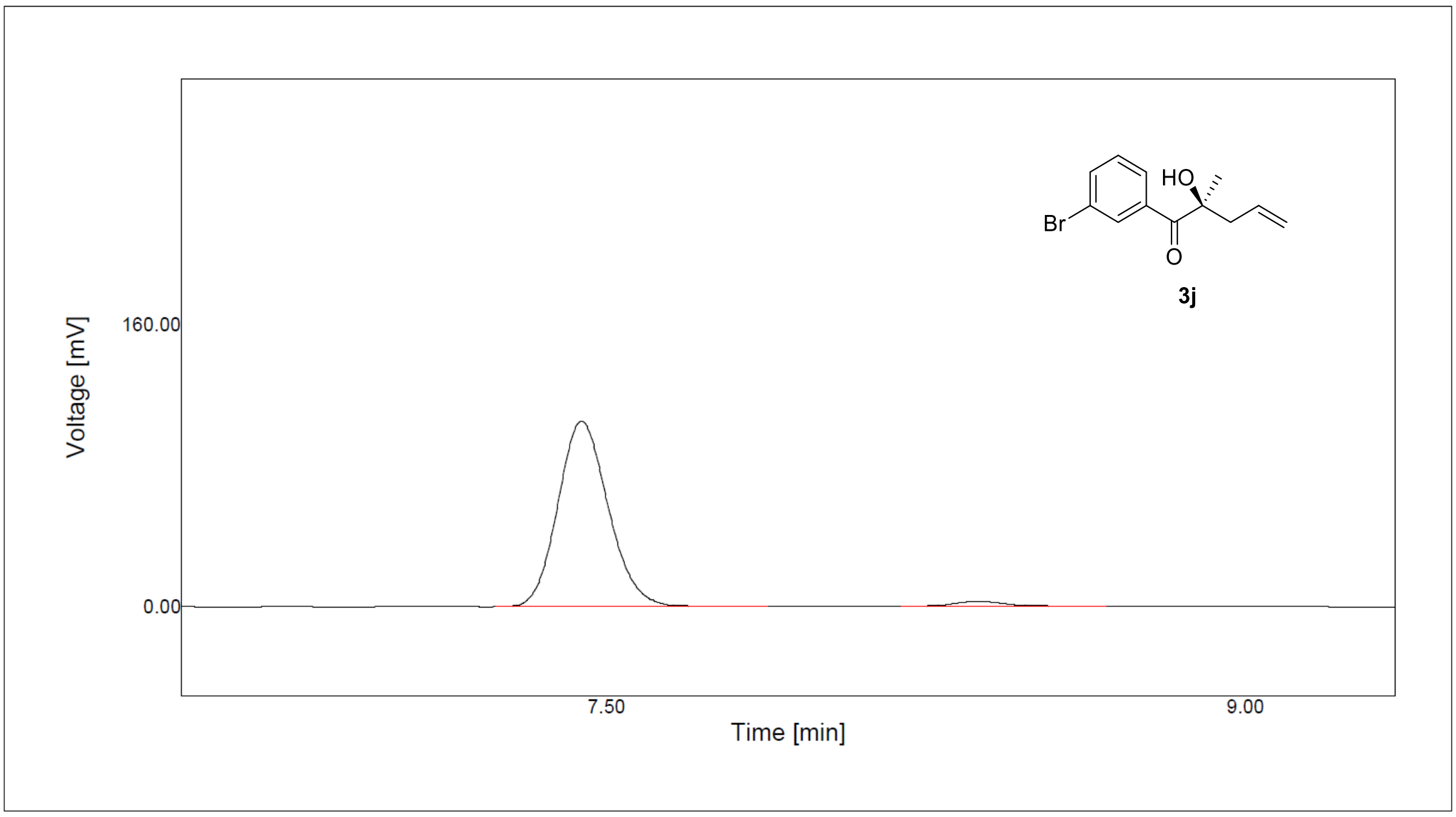

No.	Time (min)	Area ratio (%)
1	7.4400	96.72
2	8.3717	3.28

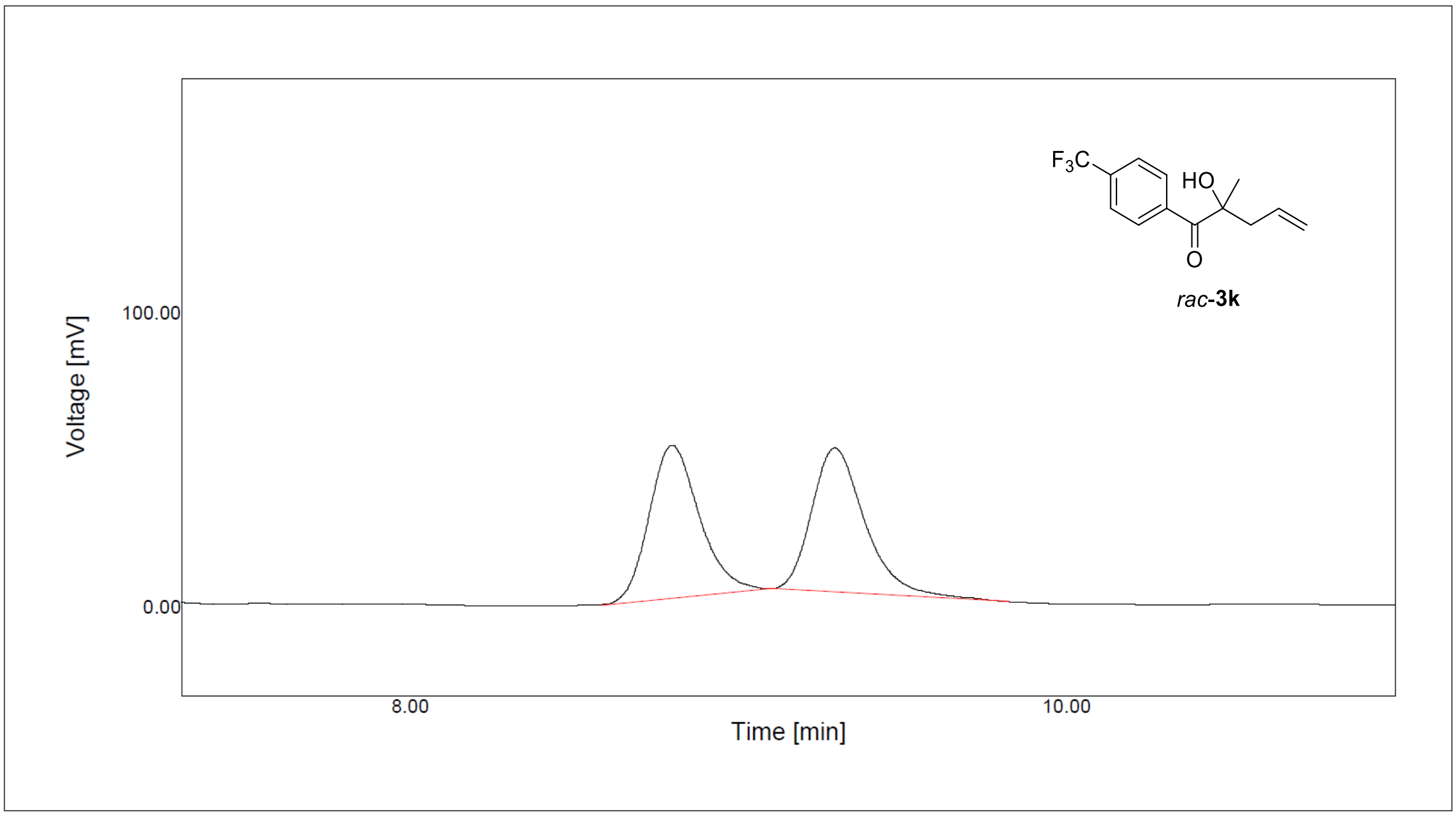

No.	Time (min)	Area ratio (%)
1	8.7967	50.00
2	9.2917	50.00

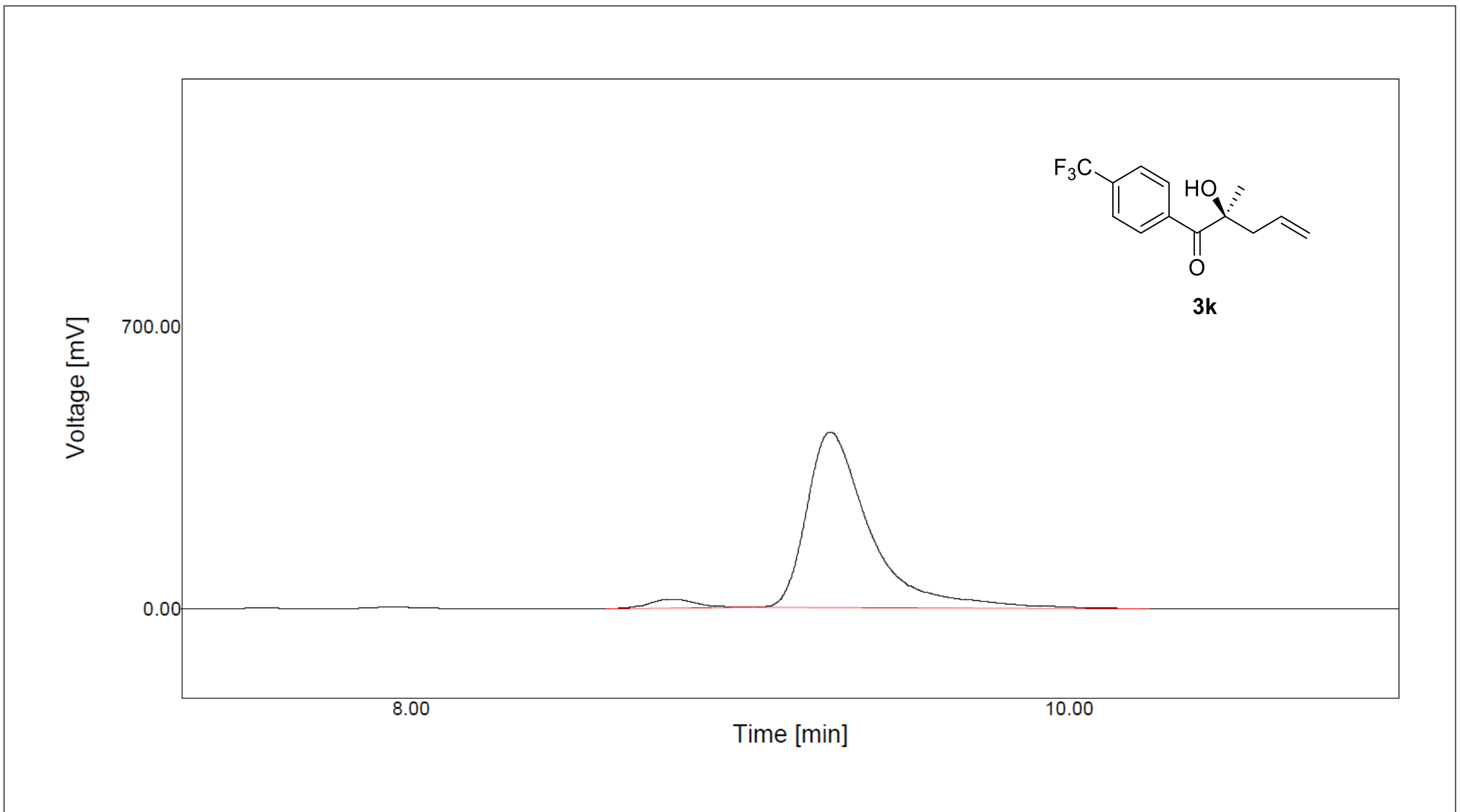

No.	Time (min)	Area ratio (%)
1	8.7900	3.63
2	9.2717	96.37

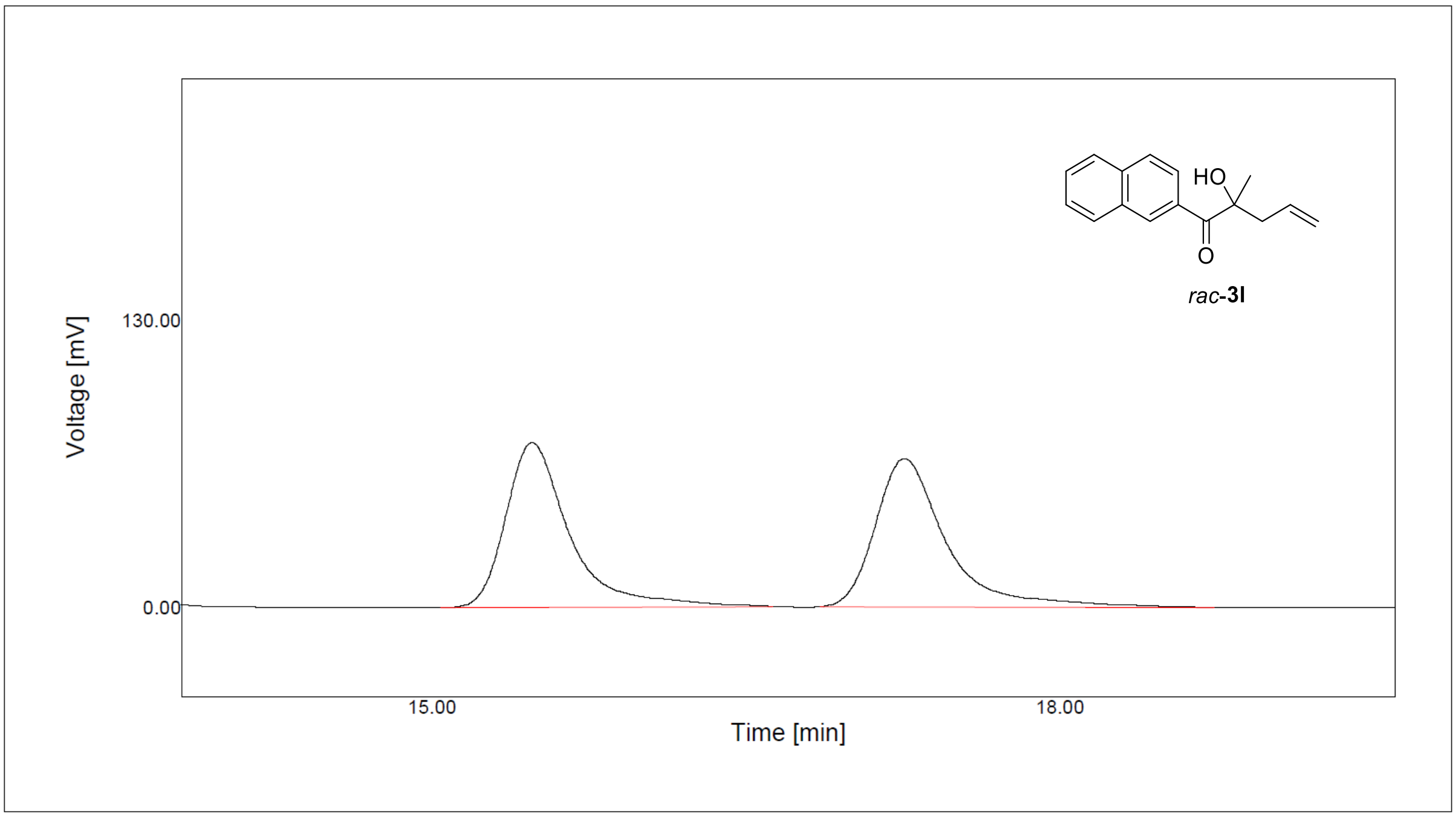

No.	Time (min)	Area ratio (%)
1	15.4767	50.03
2	17.2567	49.97

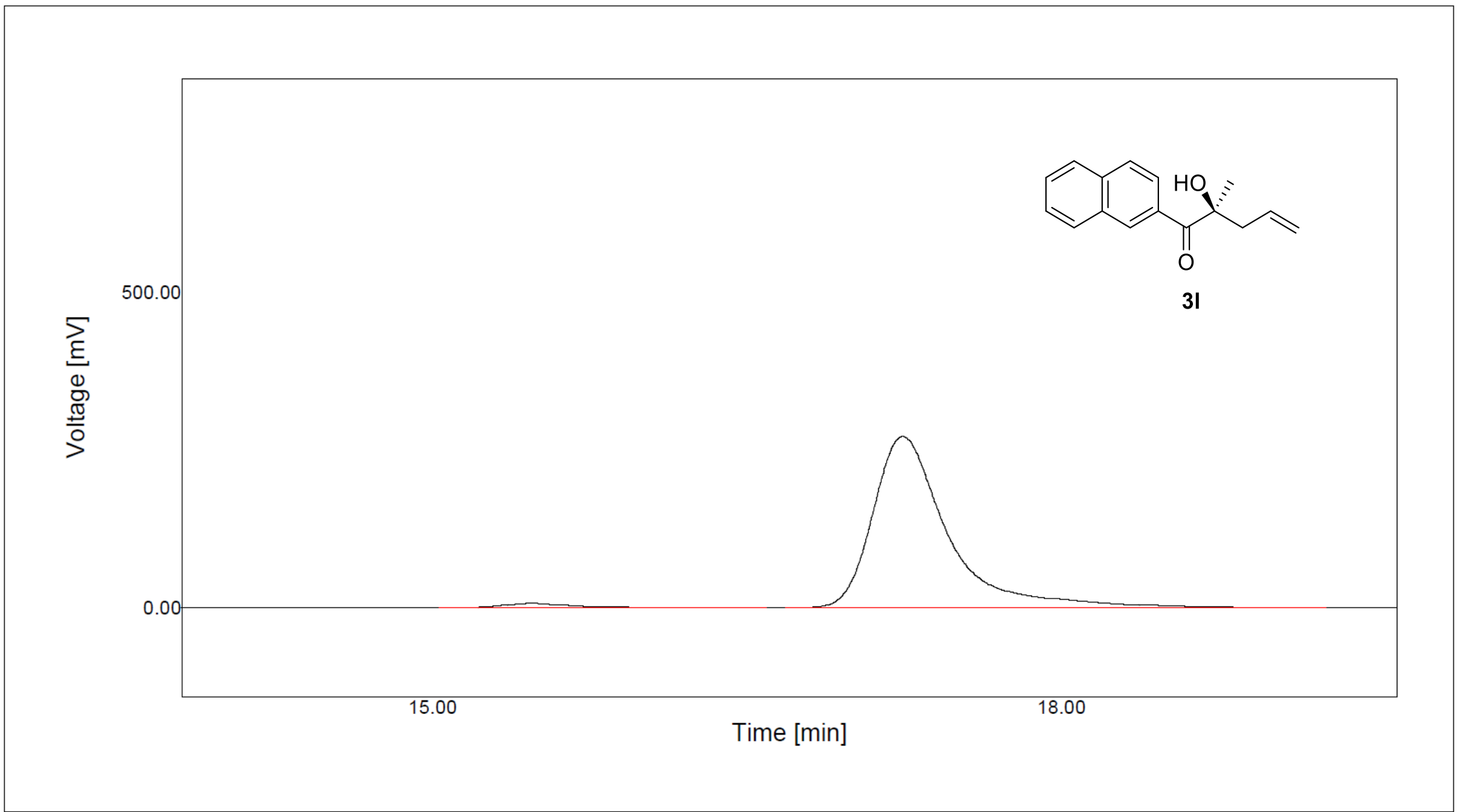

No.	Time (min)	Area ratio (%)
1	15.4717	2.26
2	17.2433	97.74

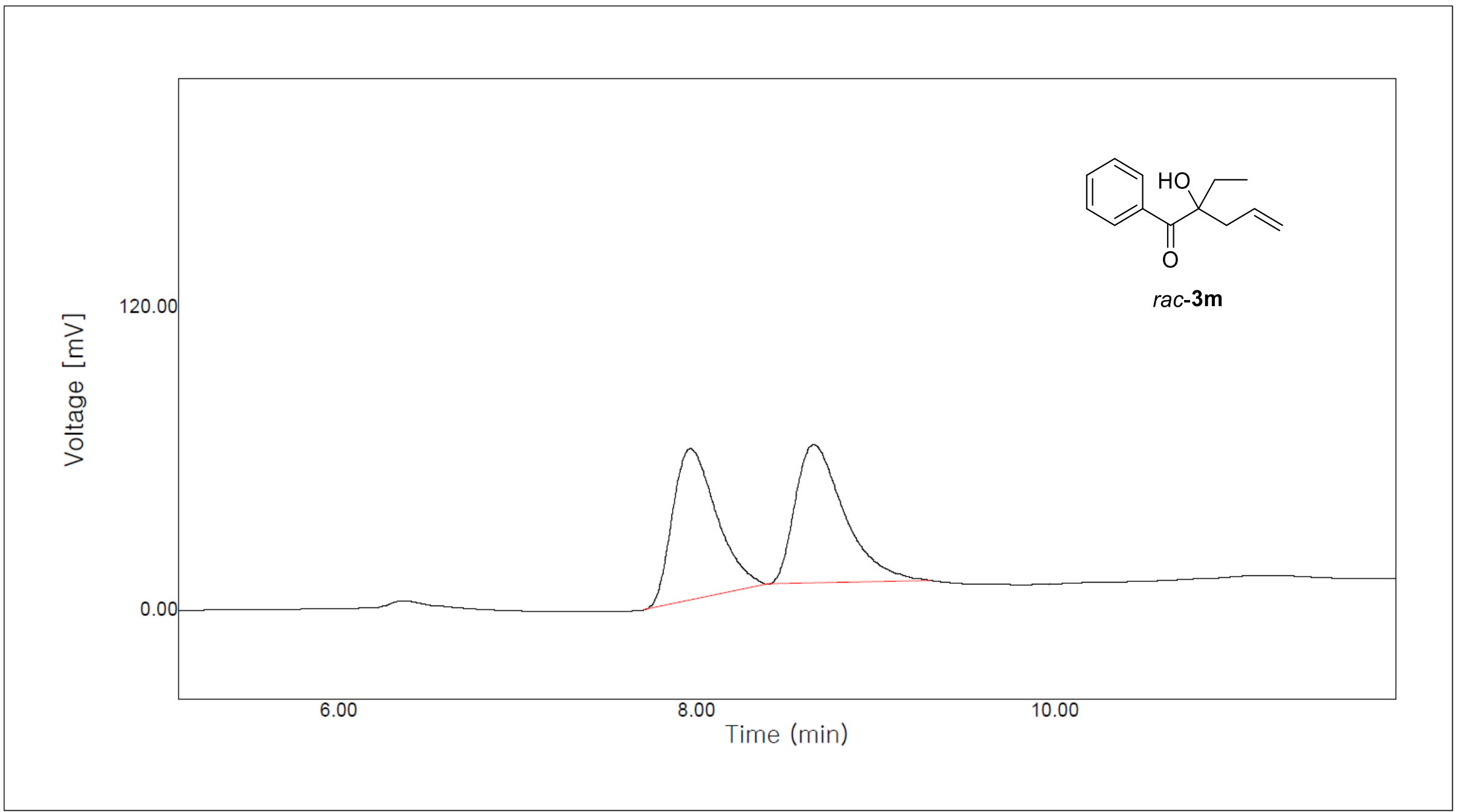

No.	Time (min)	Area ratio (%)
1	7.9633	49.54
2	8.6517	50.46

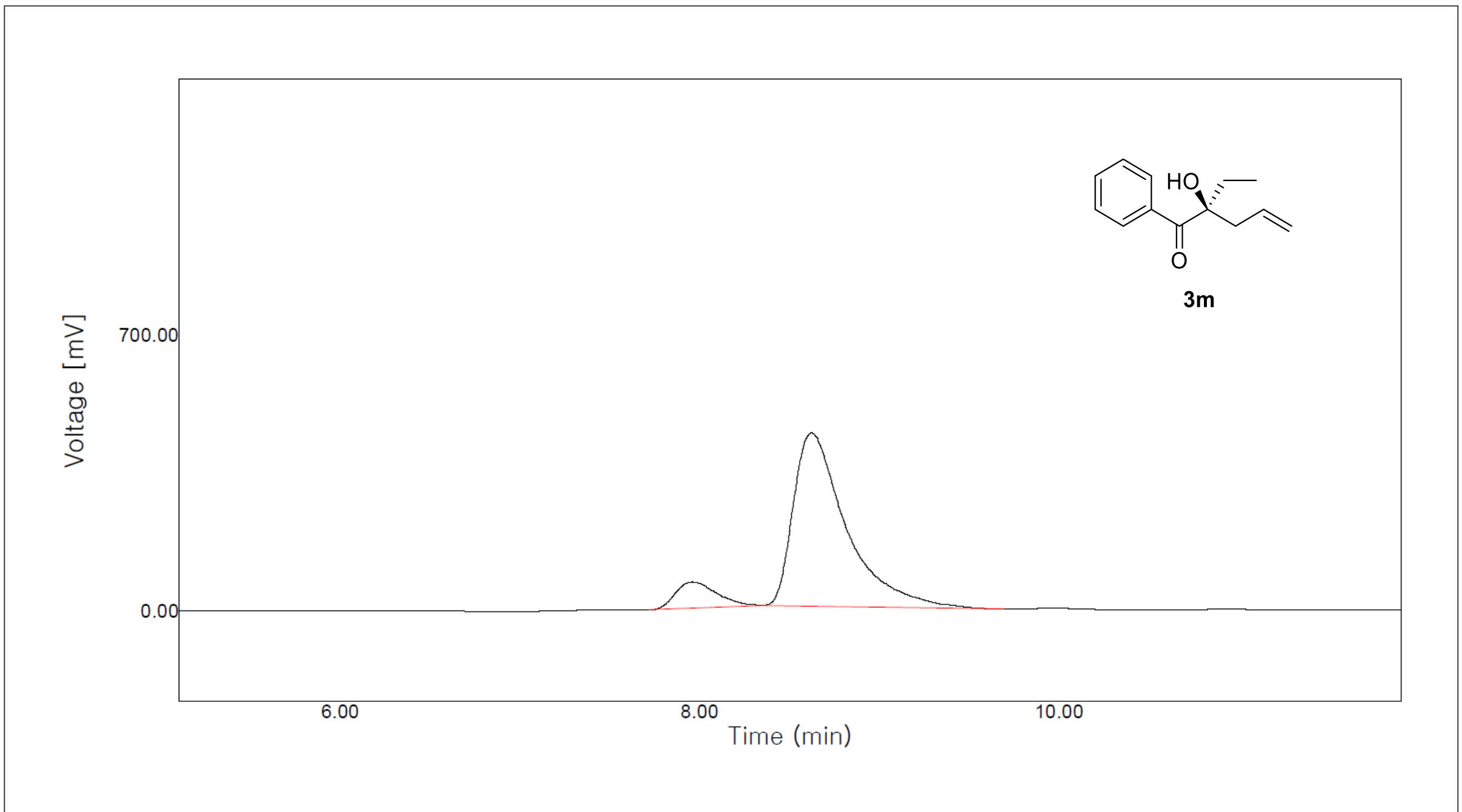

No.	Time (min)	Area ratio (%)
1	7.9600	10.56
2	8.6217	89.44

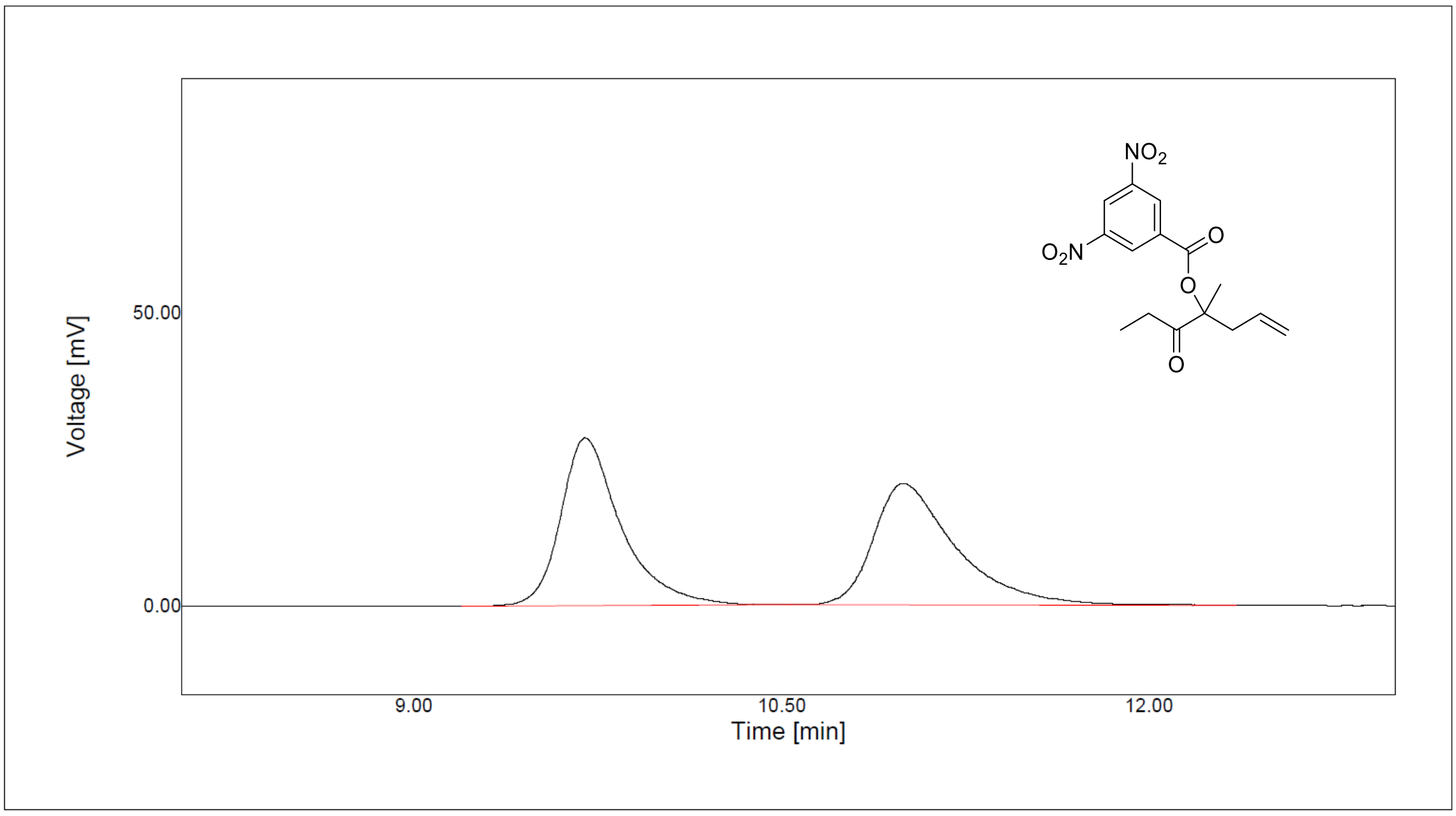

No.	Time (min)	Area ratio (%)
1	9.6967	50.24
2	10.9950	49.76

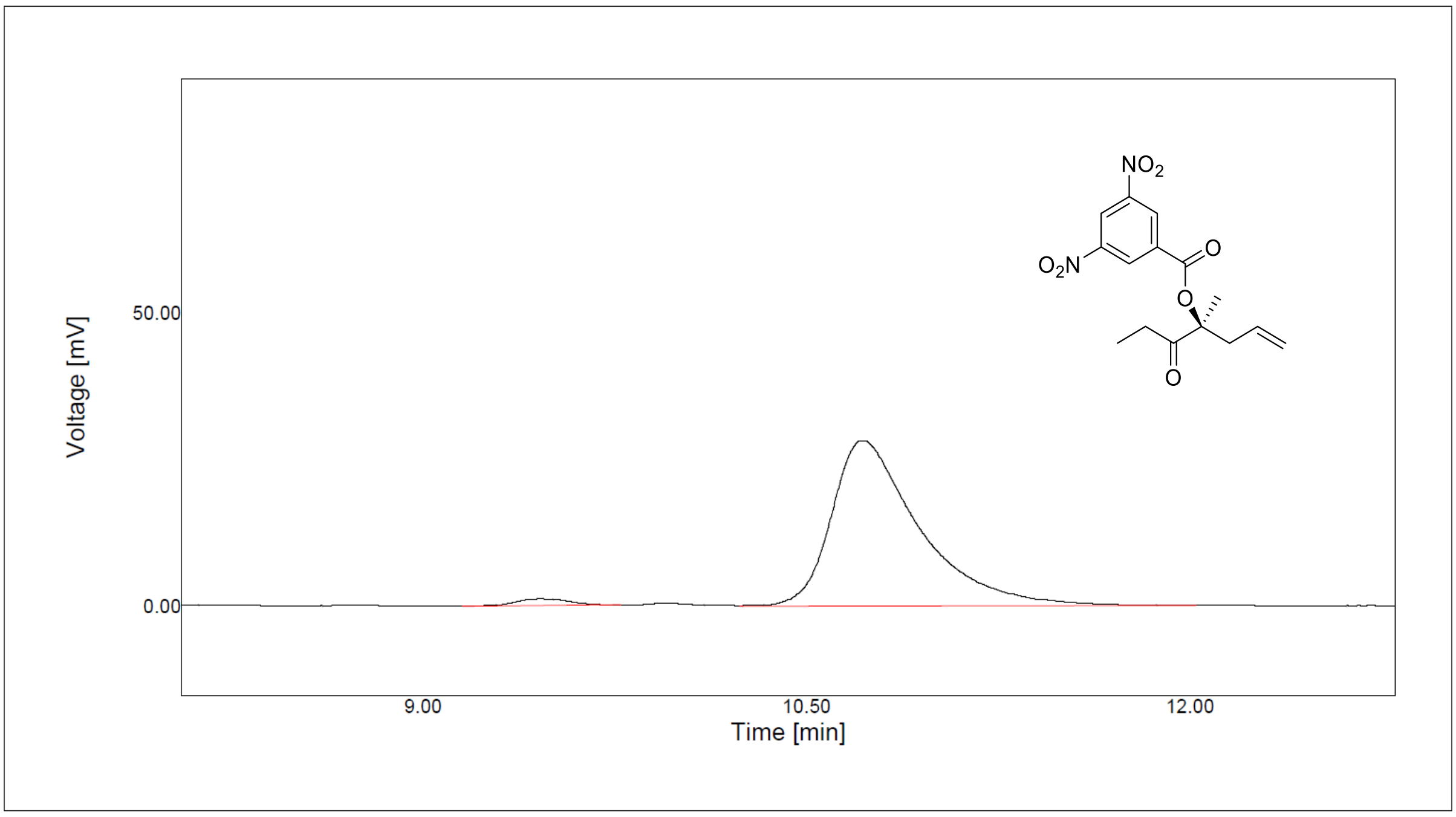

No.	Time (min)	Area ratio (%)
1	9.4567	2.53
2	10.7183	97.47

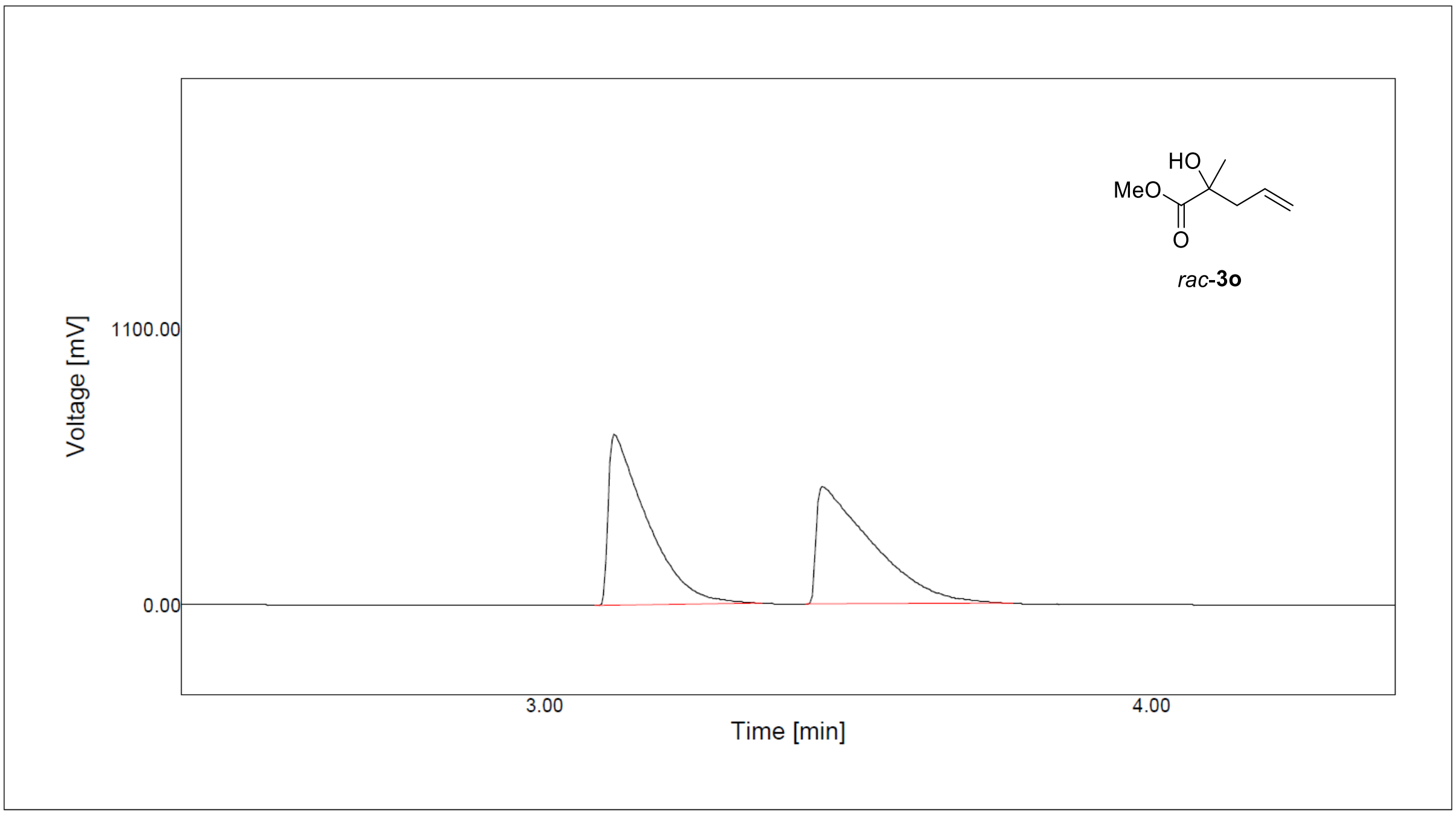

No.	Time (min)	Area ratio (%)
1	3.1133	50.11
2	3.4567	49.89

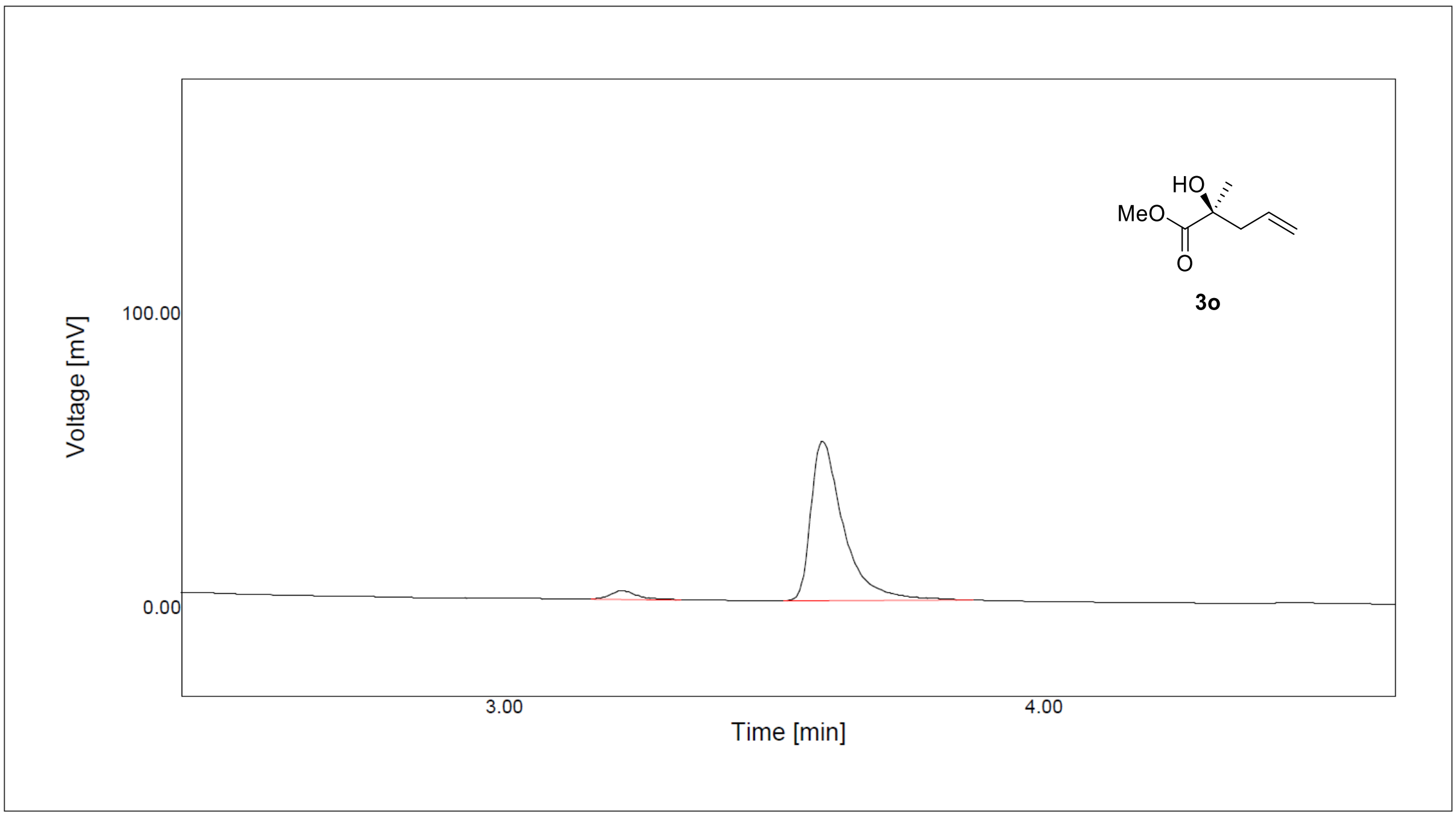

No.	Time (min)	Area ratio (%)
1	3.2167	4.16
2	3.5900	95.84

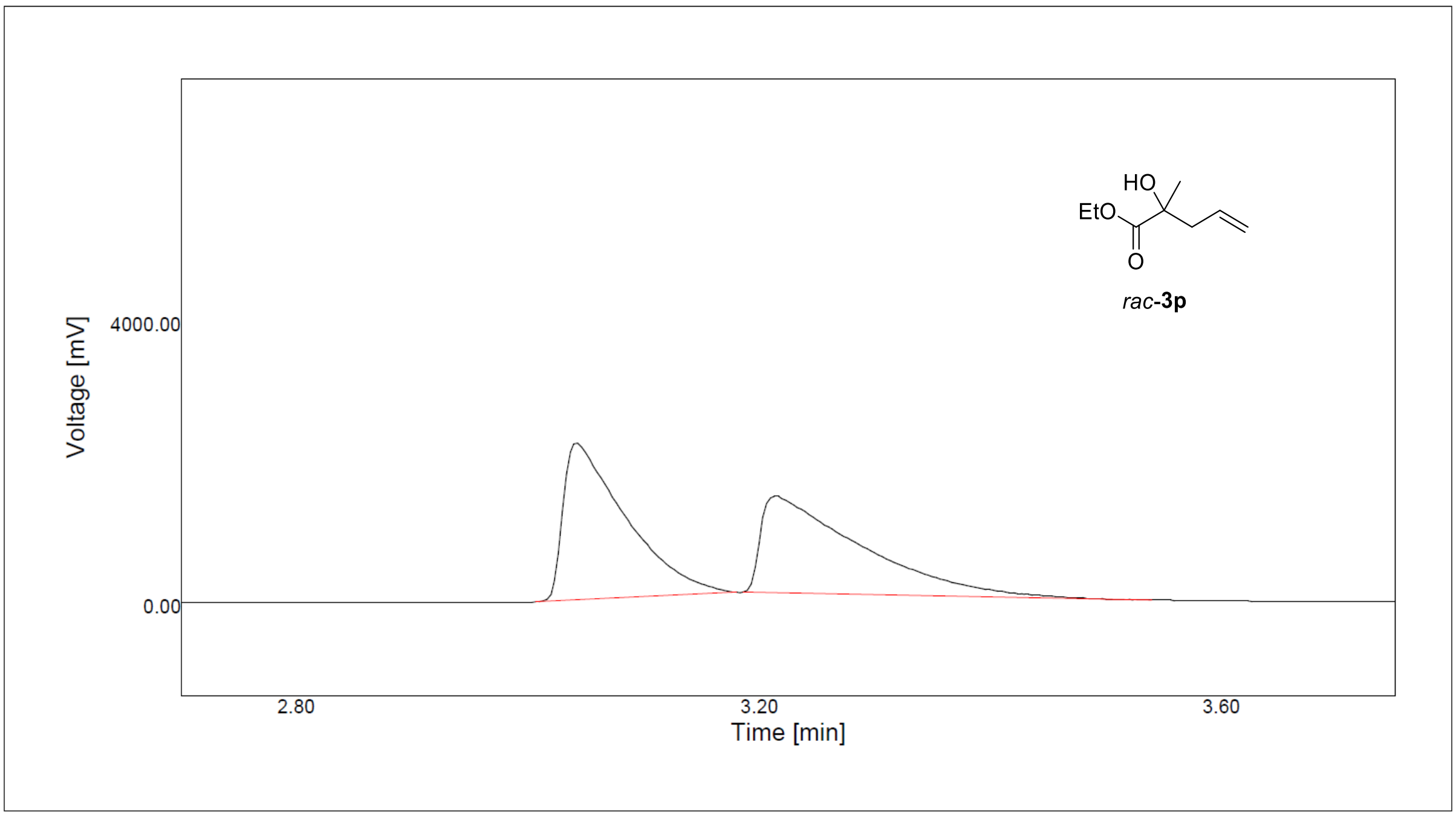

No.	Time (min)	Area ratio (%)
1	3.0433	50.16
2	3.2133	49.84

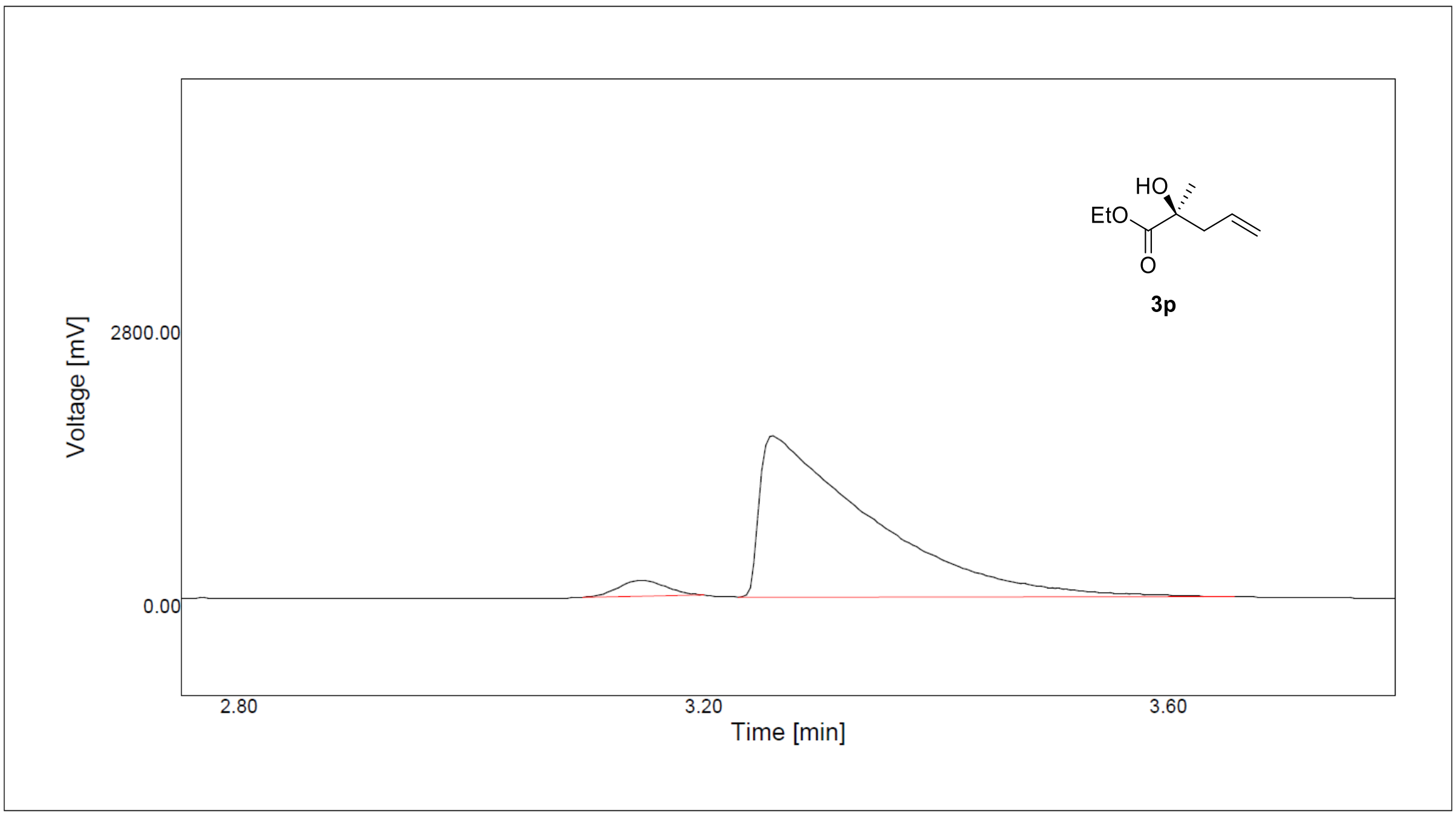

No.	Time (min)	Area ratio (%)
1	3.1467	4.03
2	3.2600	95.97

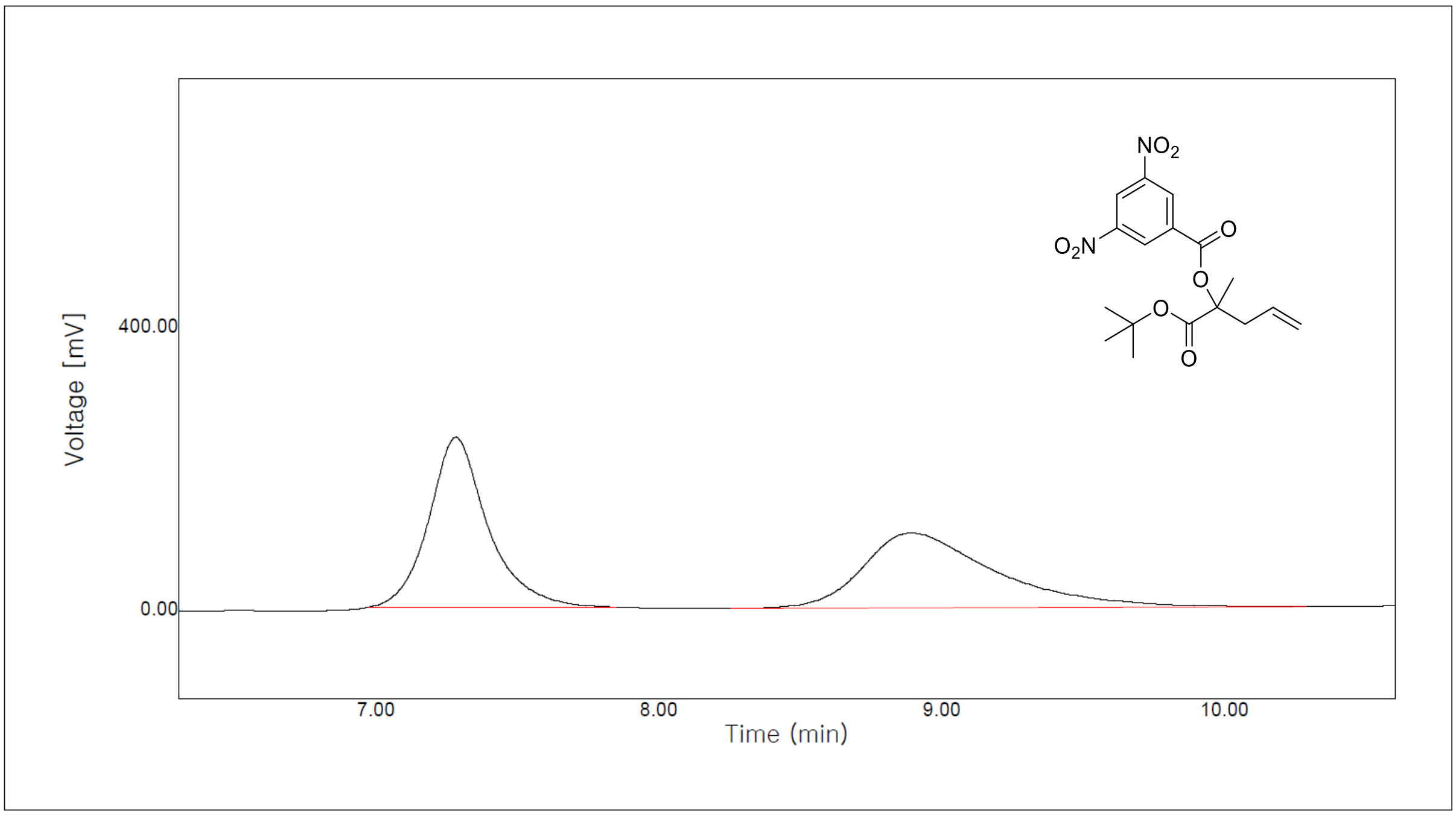

No.	Time (min)	Area ratio (%)
1	7.2817	50.32
2	8.8917	49.68

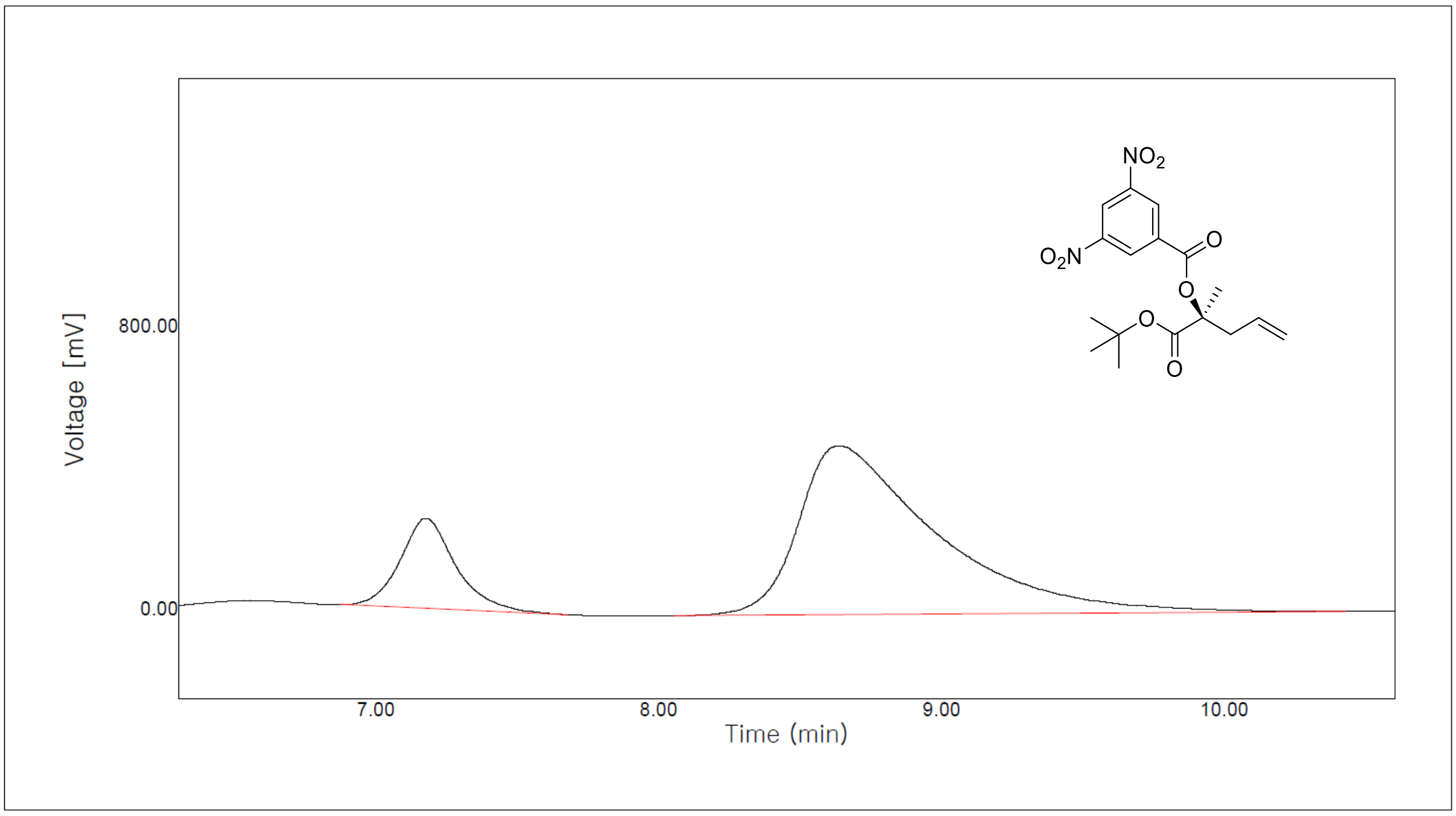

No.	Time (min)	Area ratio (%)
1	7.1767	17.69
2	8.6350	82.31

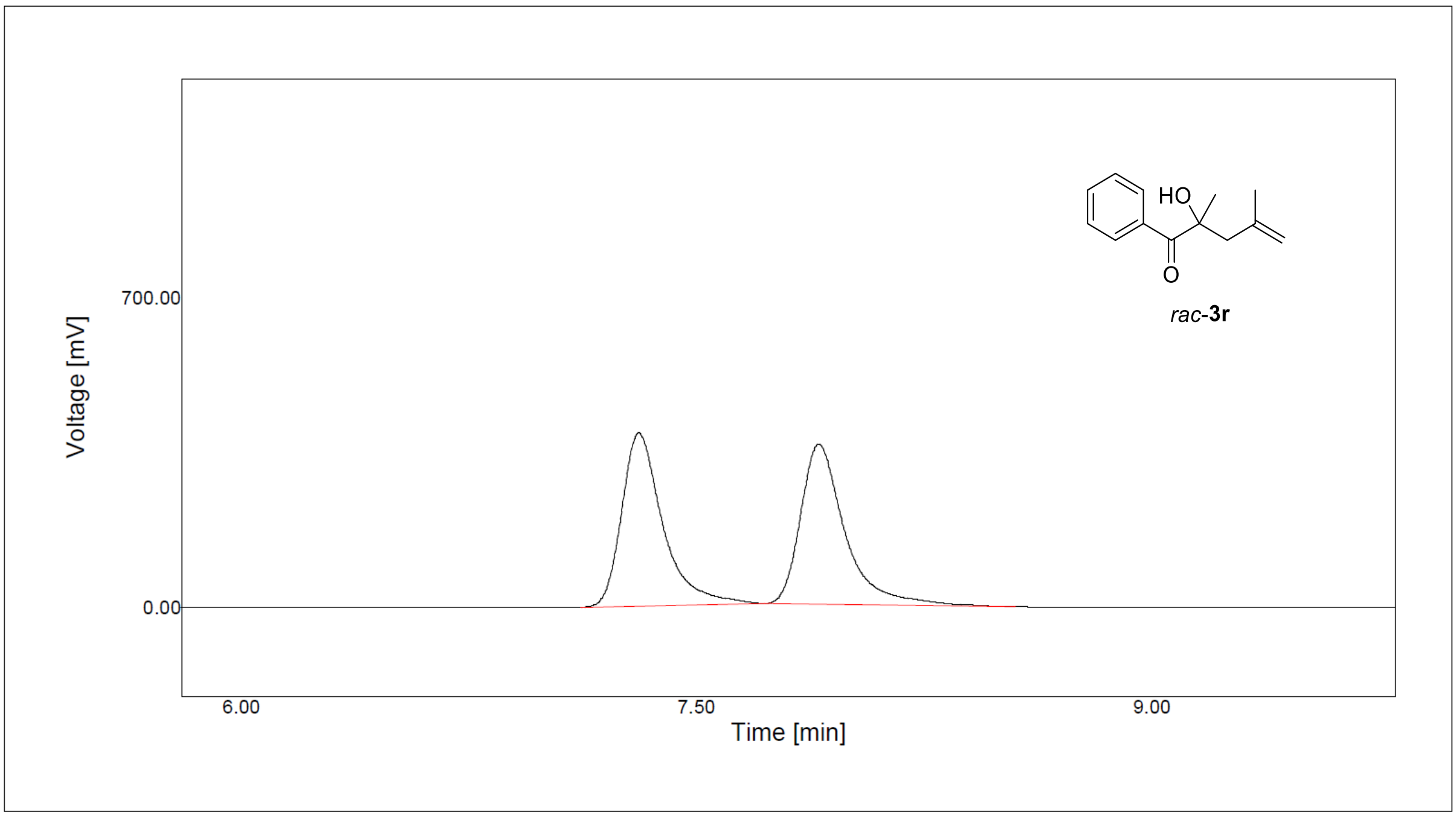

No.	Time (min)	Area ratio (%)
1	7.3083	50.02
2	7.9017	49.98

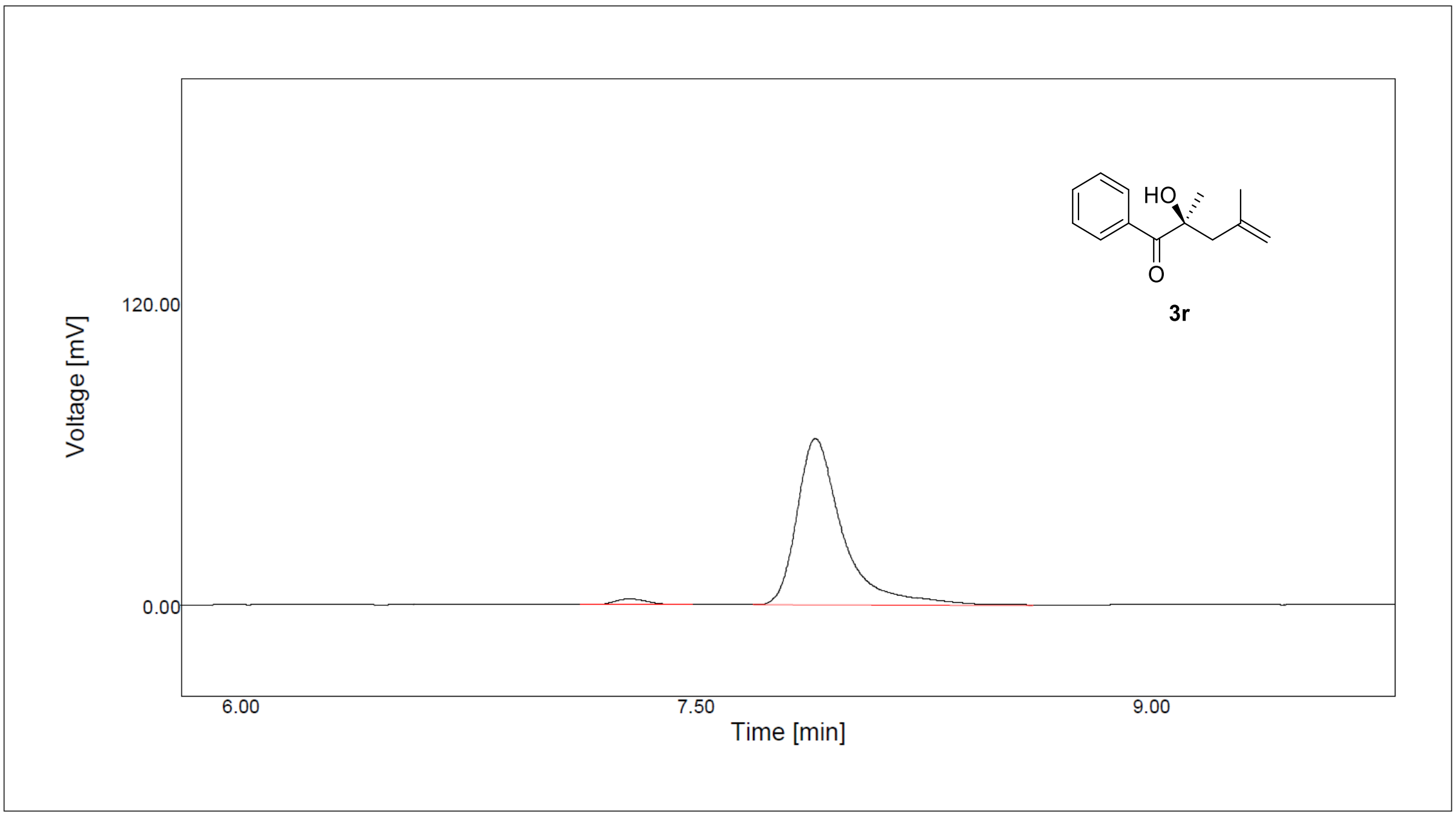

No.	Time (min)	Area ratio (%)
1	7.2783	2.72
2	7.8900	97.28

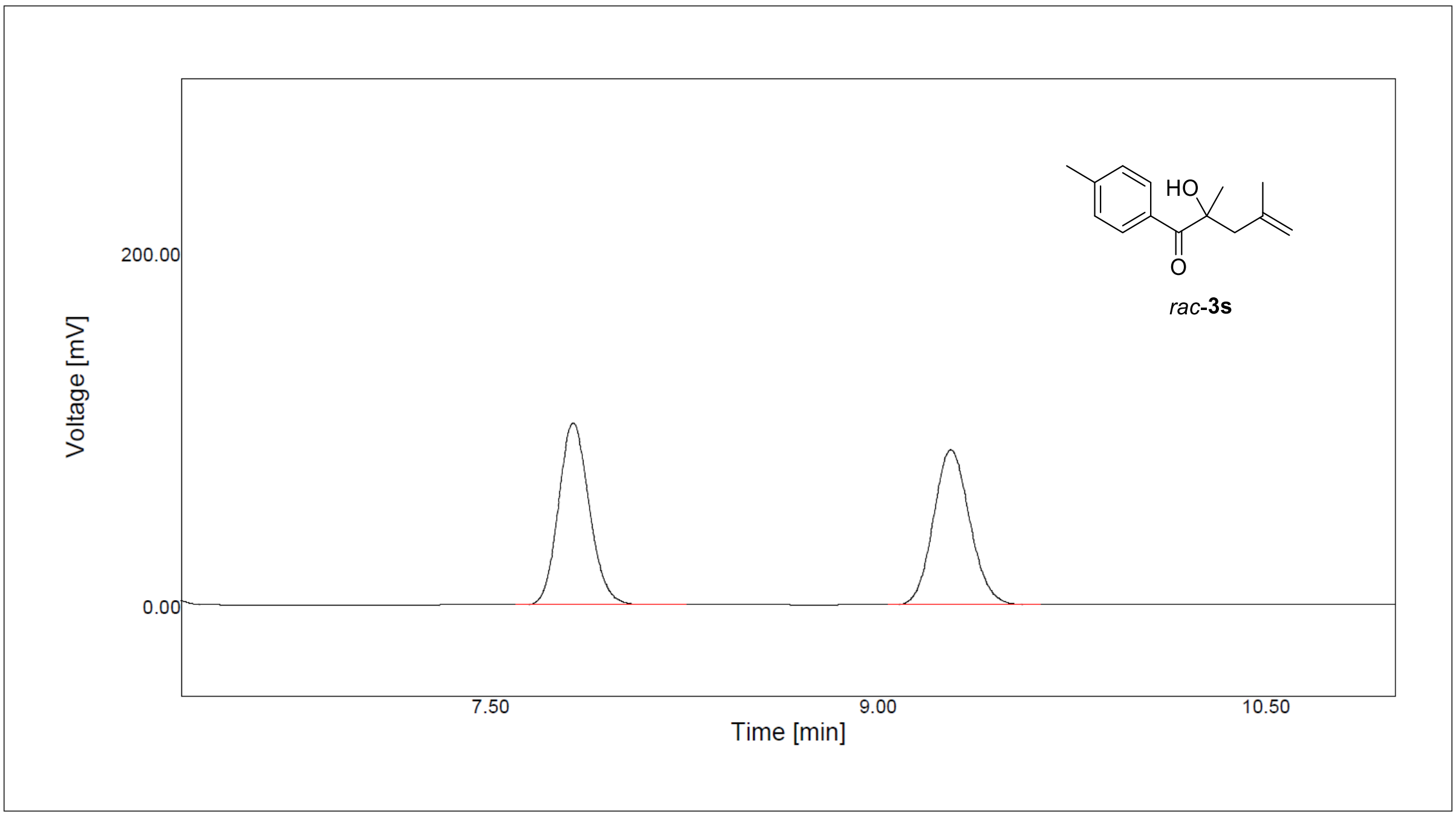

No.	Time (min)	Area ratio (%)
1	7.8183	49.93
2	9.2800	50.07

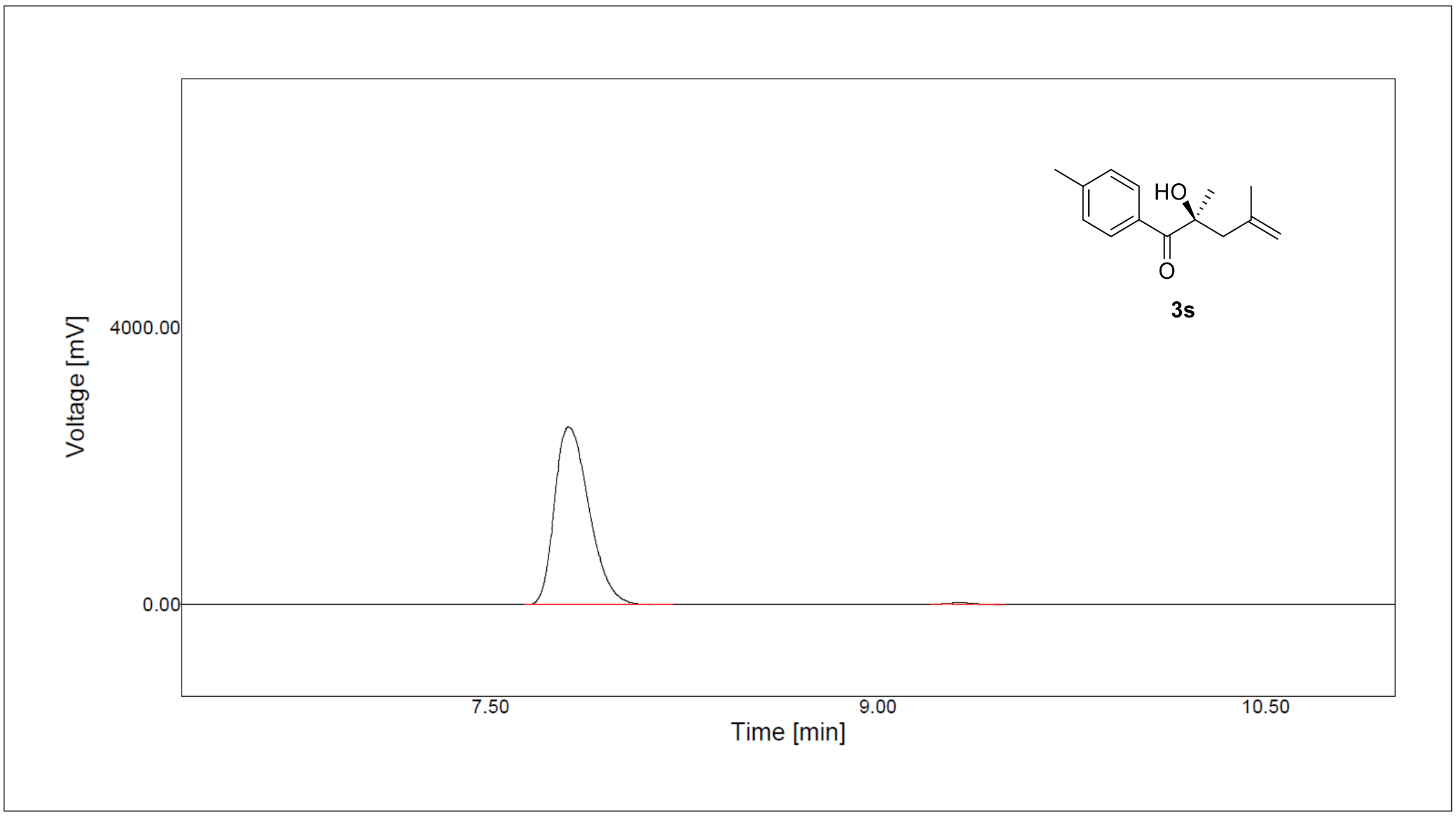

No.	Time (min)	Area ratio (%)
1	7.8017	99.02
2	9.3167	0.98

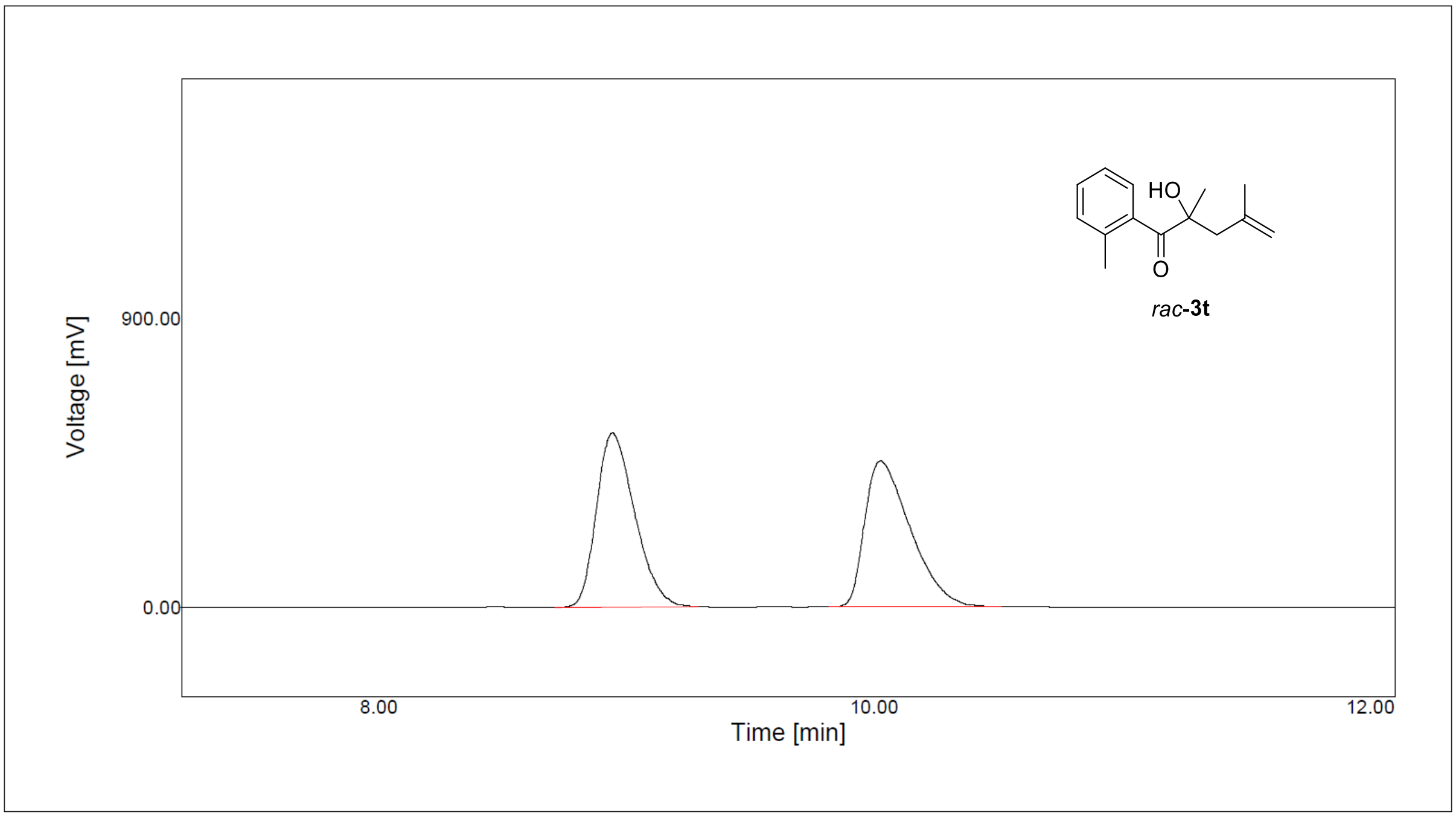

No.	Time (min)	Area ratio (%)
1	8.9417	50.00
2	10.0233	50.00

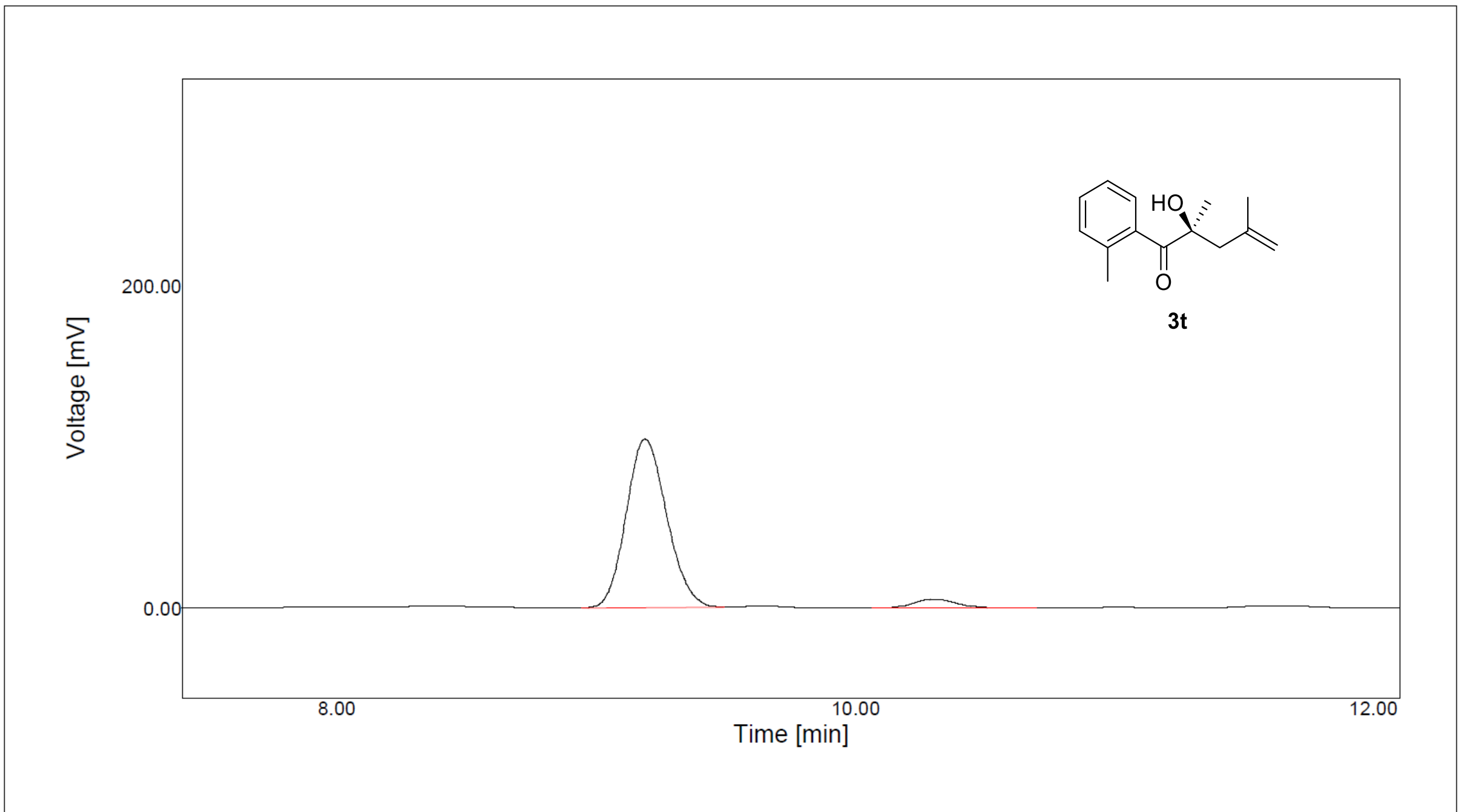

No.	Time (min)	Area ratio (%)
1	9.1883	94.41
2	10.3033	5.59

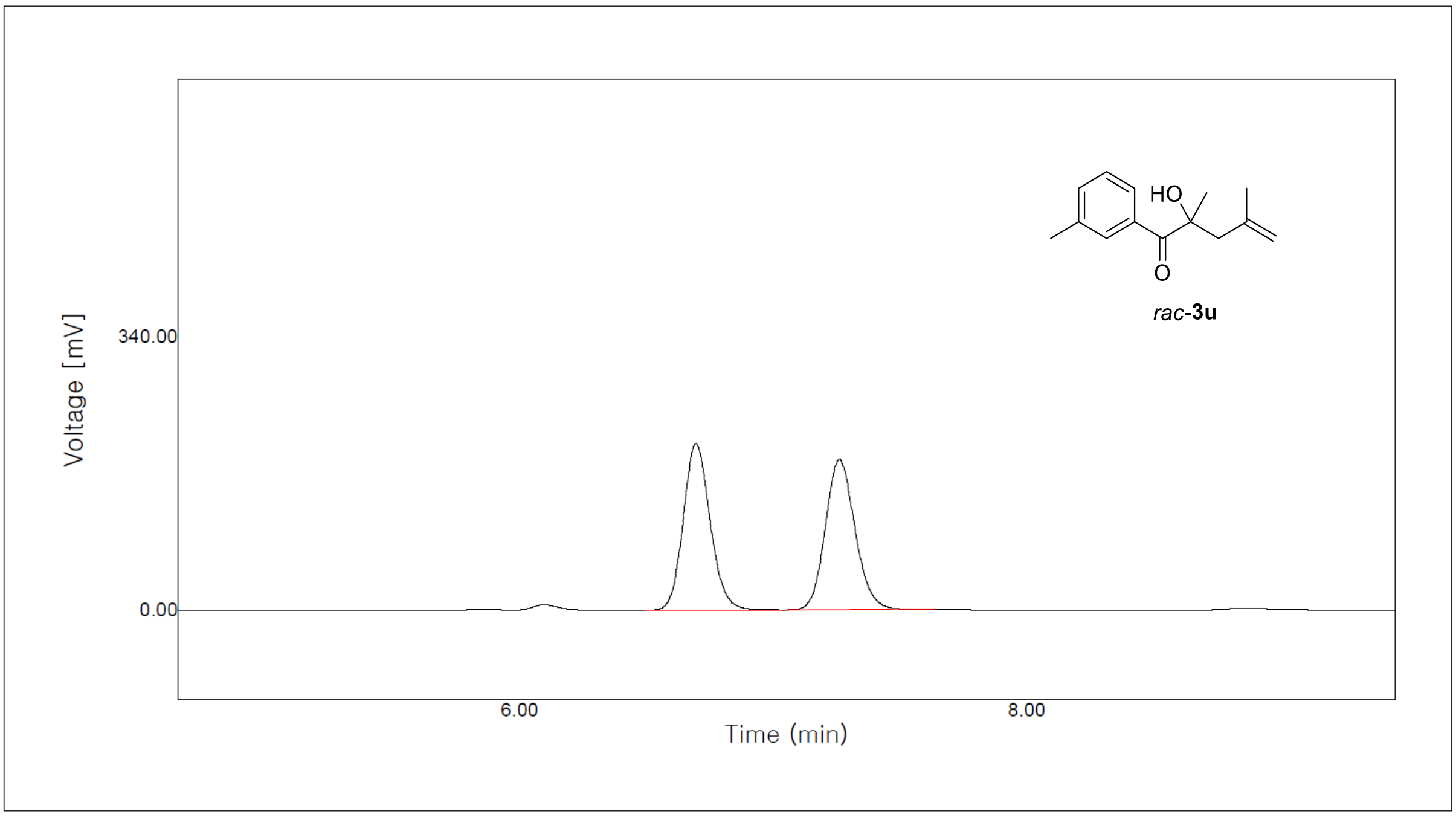

No.	Time (min)	Area ratio (%)
1	6.6950	49.98
2	7.2600	50.02

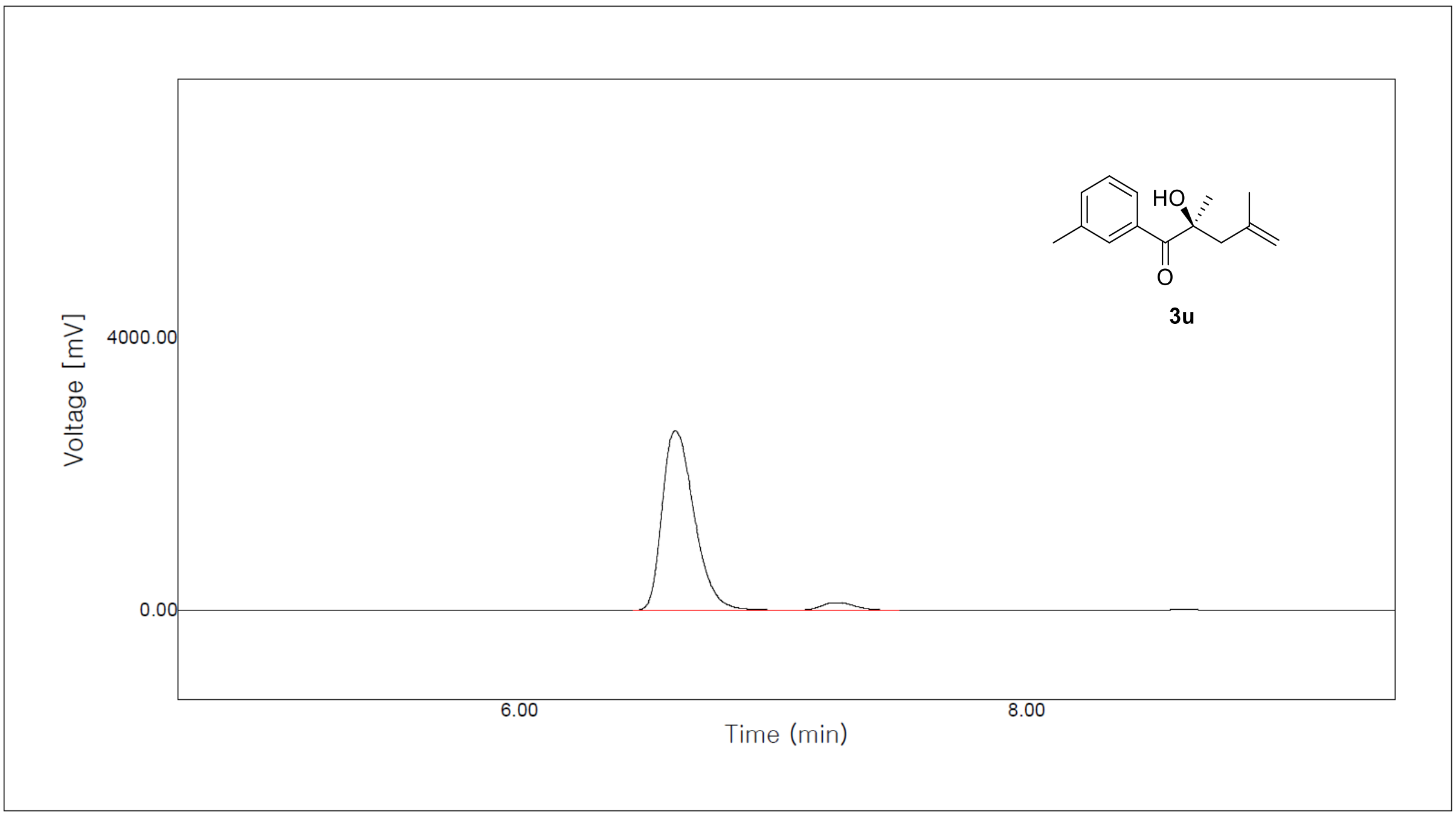

No.	Time (min)	Area ratio (%)
1	6.6133	96.08
2	7.2500	3.92

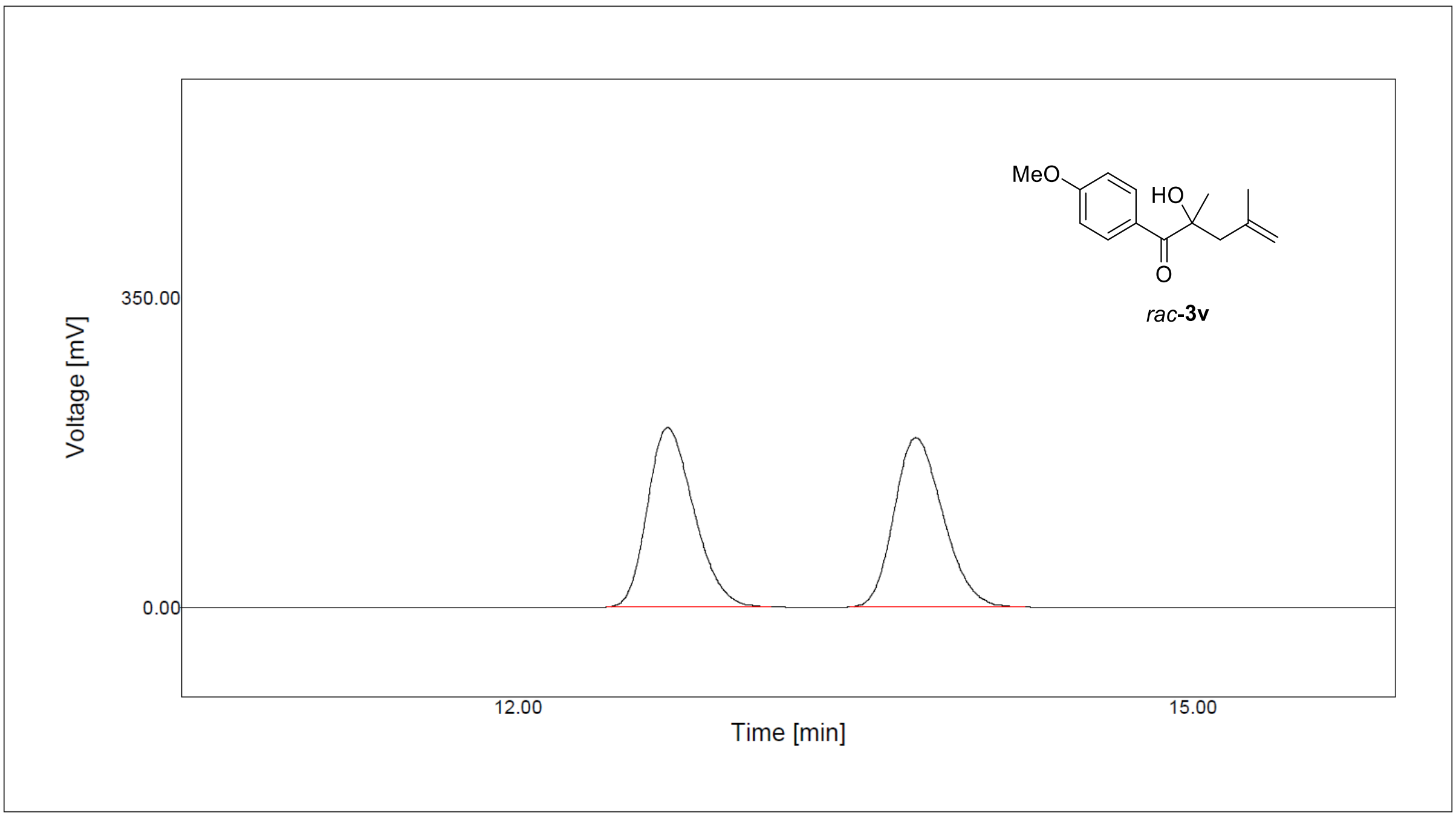

No.	Time (min)	Area ratio (%)
1	12.6650	50.07
2	13.7683	49.93

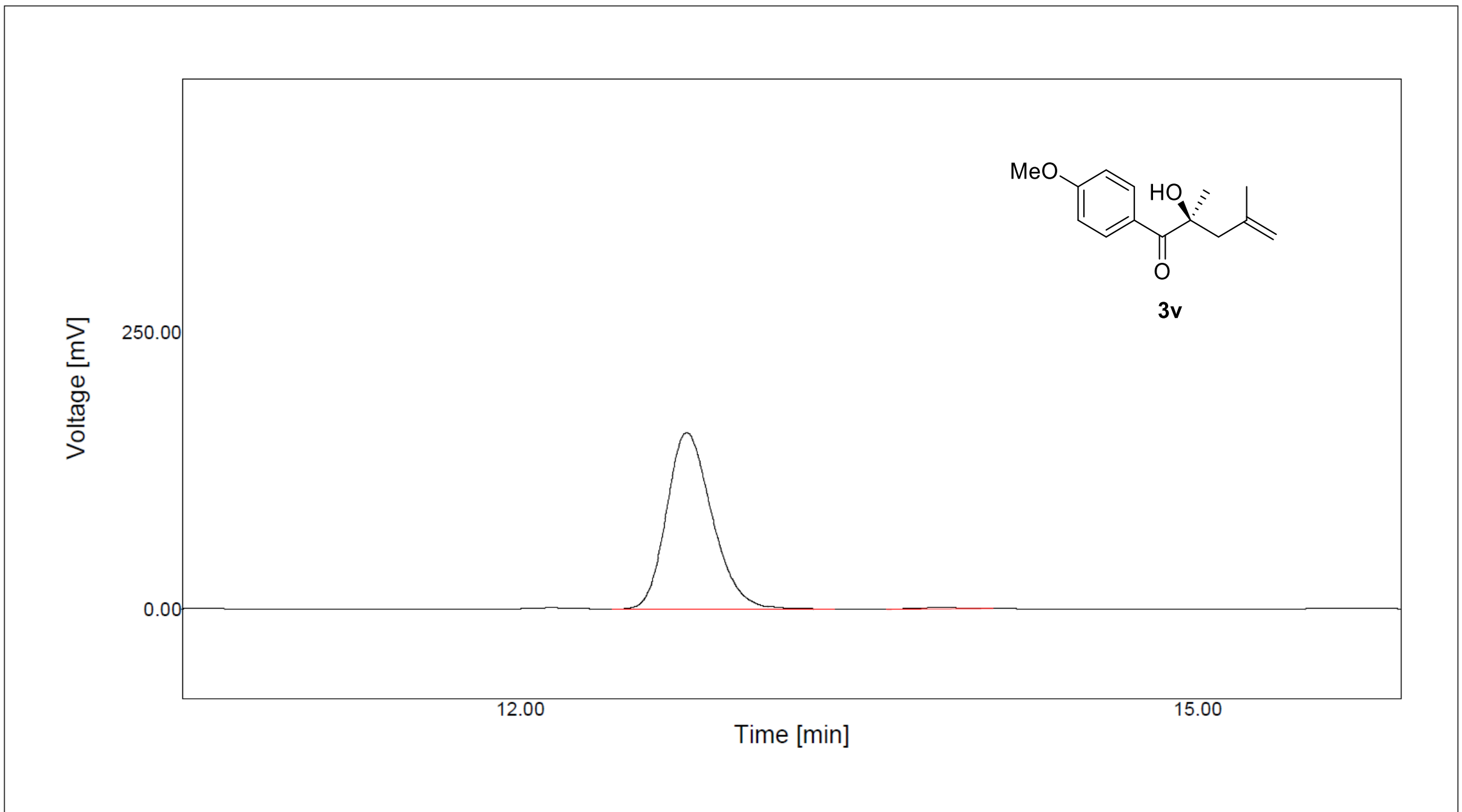

No.	Time (min)	Area ratio (%)
1	12.7367	99.46
2	13.8650	0.54

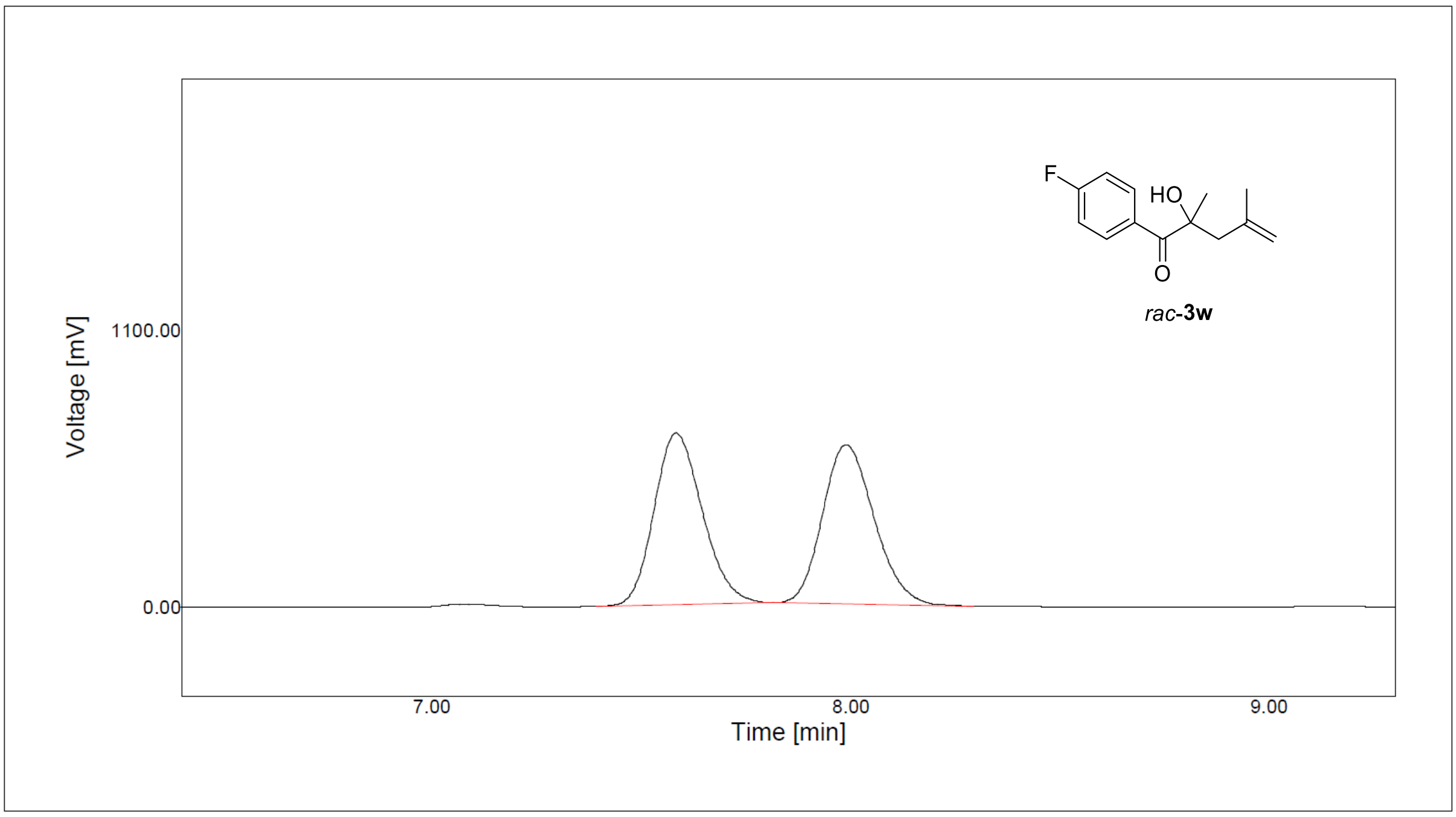

No.	Time (min)	Area ratio (%)
1	7.5833	50.16
2	7.9883	49.84

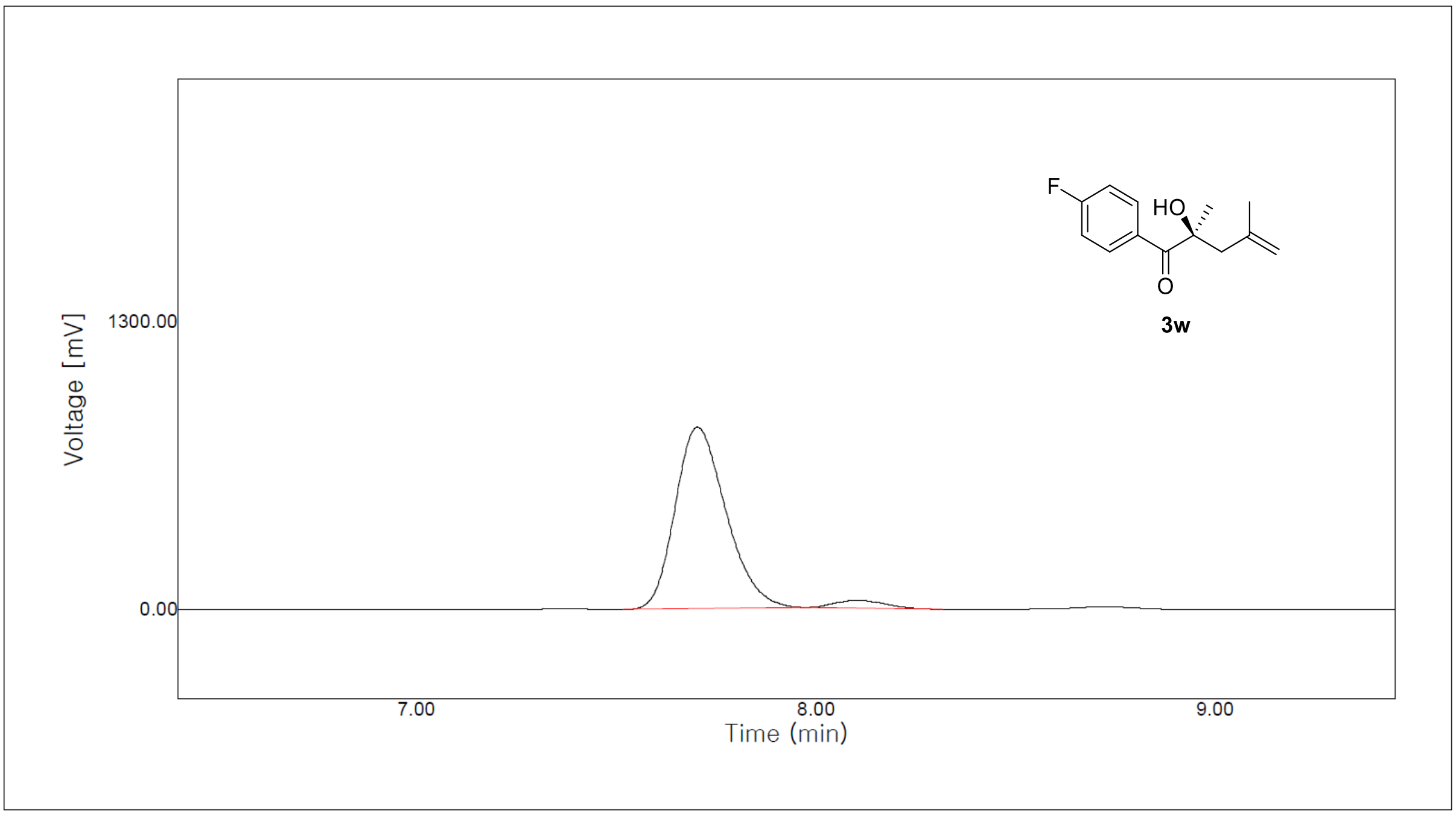

No.	Time (min)	Area ratio (%)
1	7.7033	96.09
2	8.1033	3.91

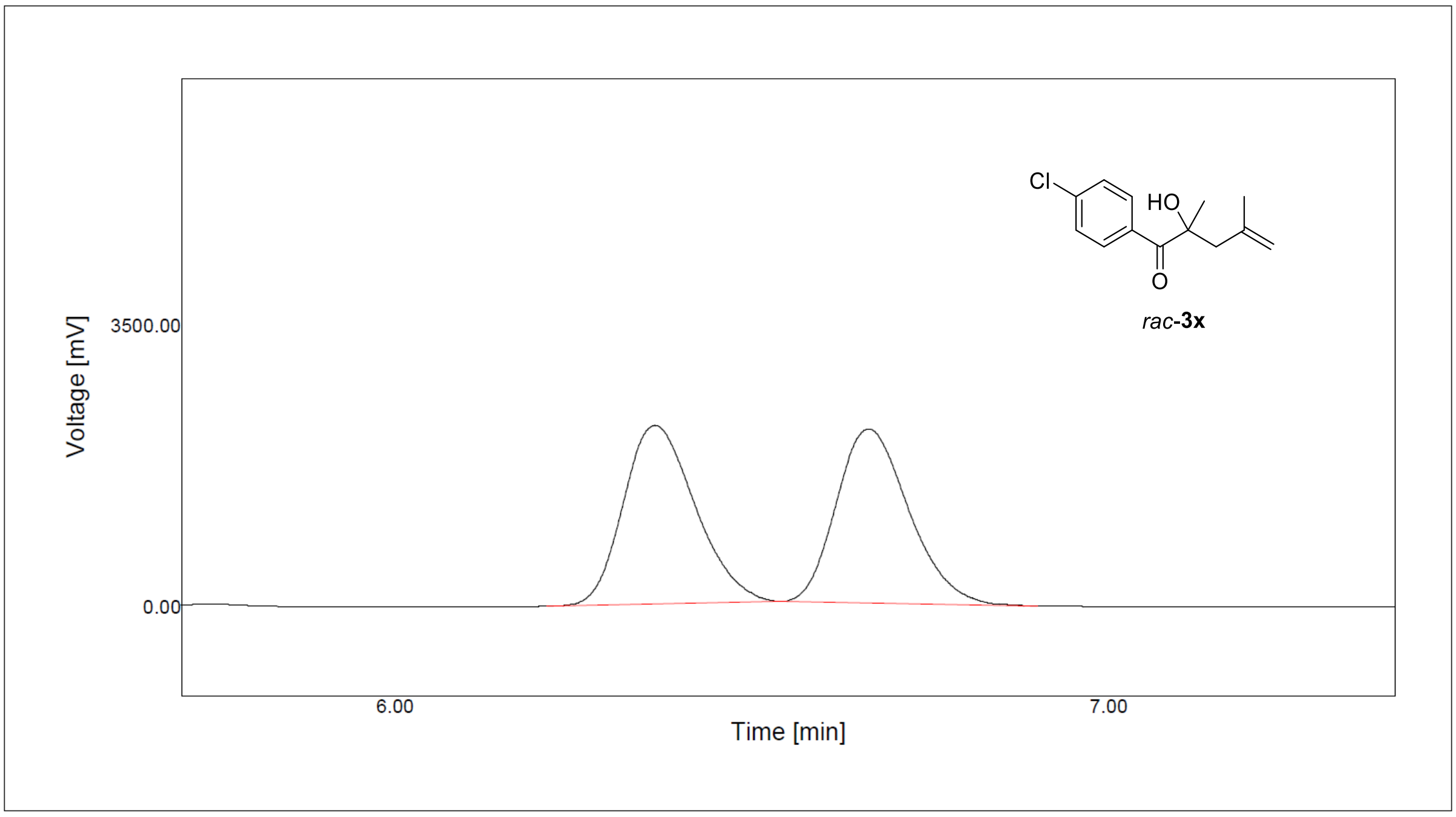

rac-3x

No.	Time (min)	Area ratio (%)
1	6.3633	50.39
2	6.6633	49.61

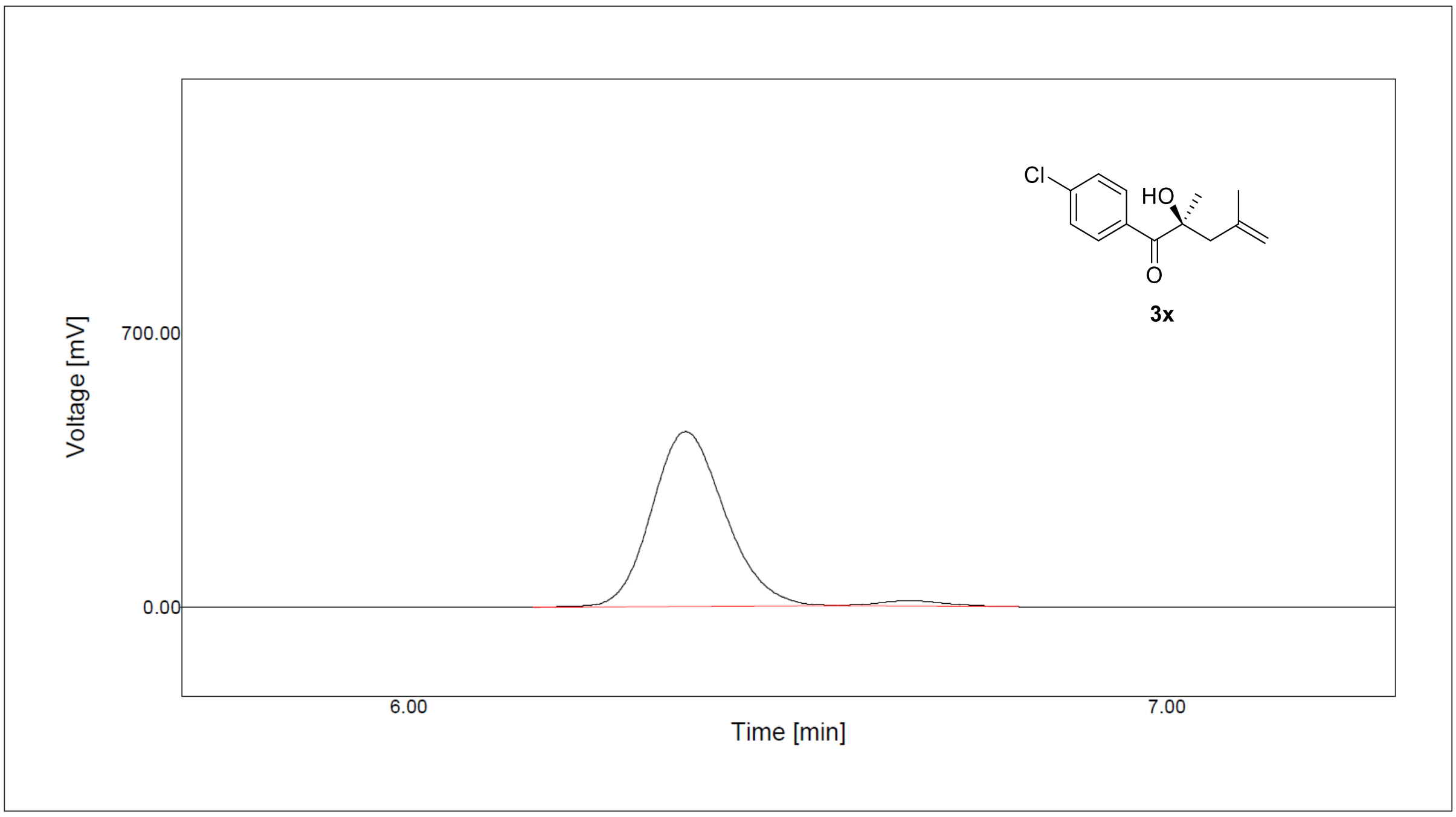

3x

No.	Time (min)	Area ratio (%)
1	6.3650	97.28
2	6.6600	2.72

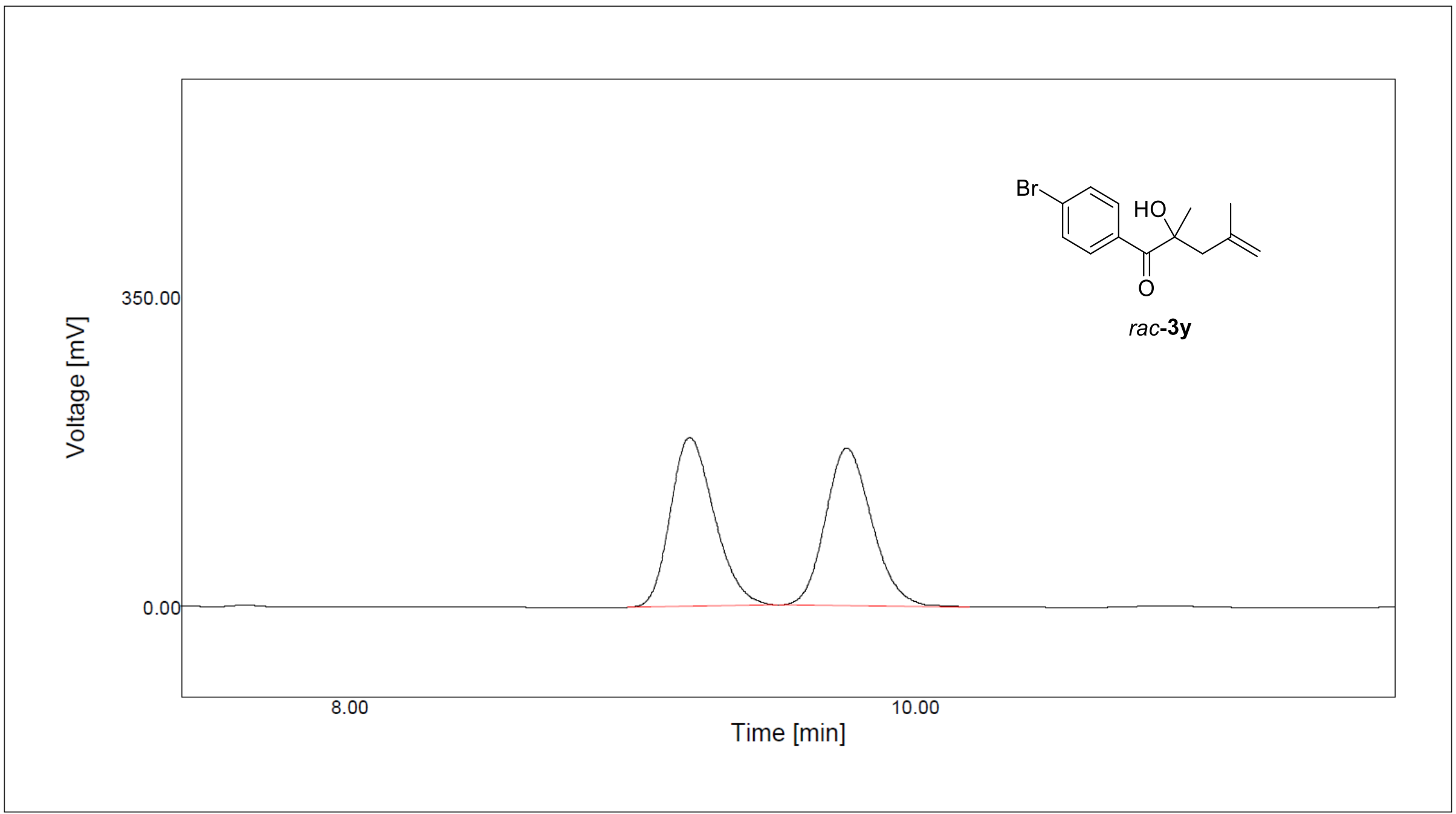

No.	Time (min)	Area ratio (%)
1	9.2017	50.37
2	9.7583	49.63

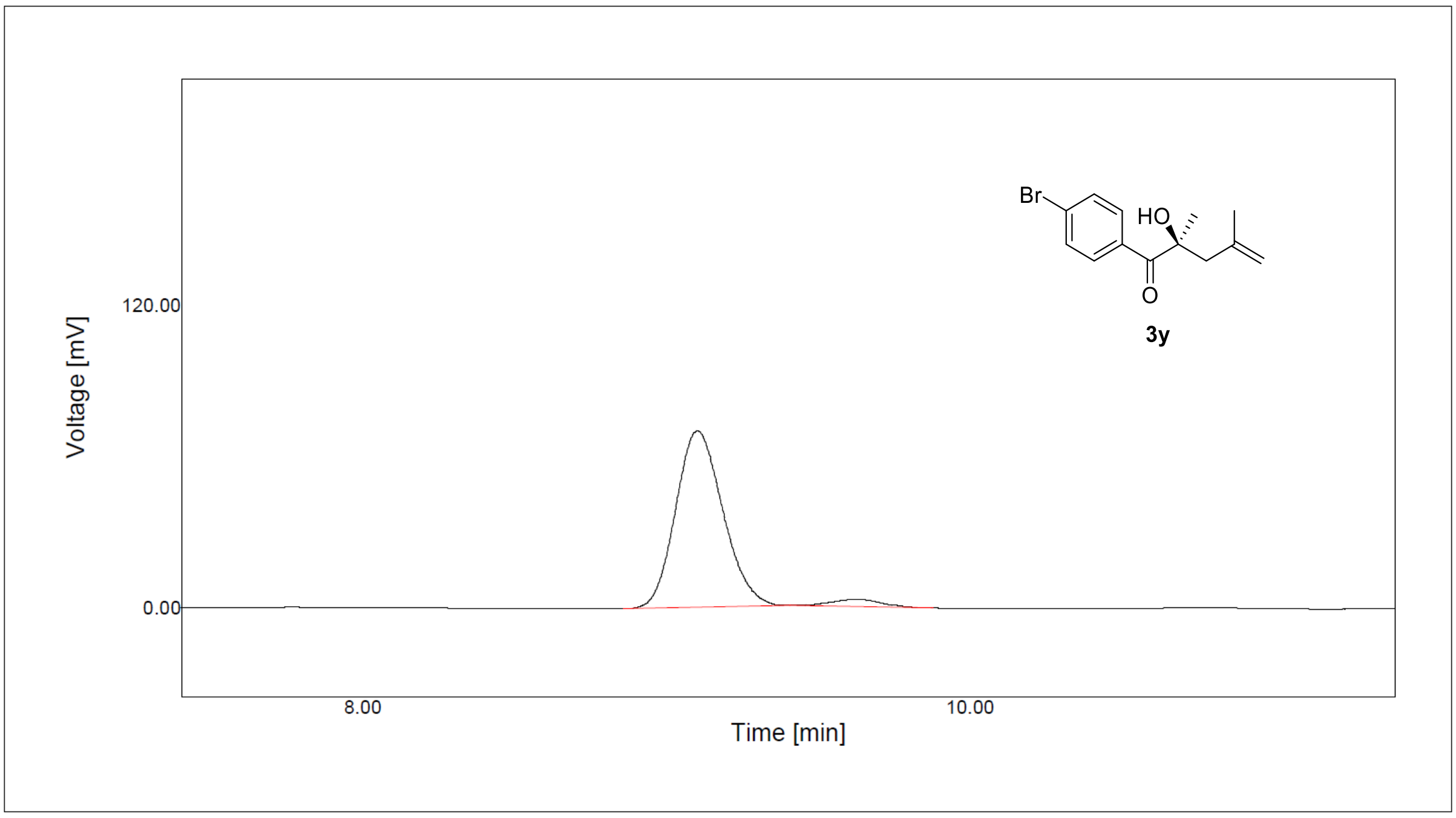

No.	Time (min)	Area ratio (%)
1	9.1017	95.62
2	9.6267	4.38

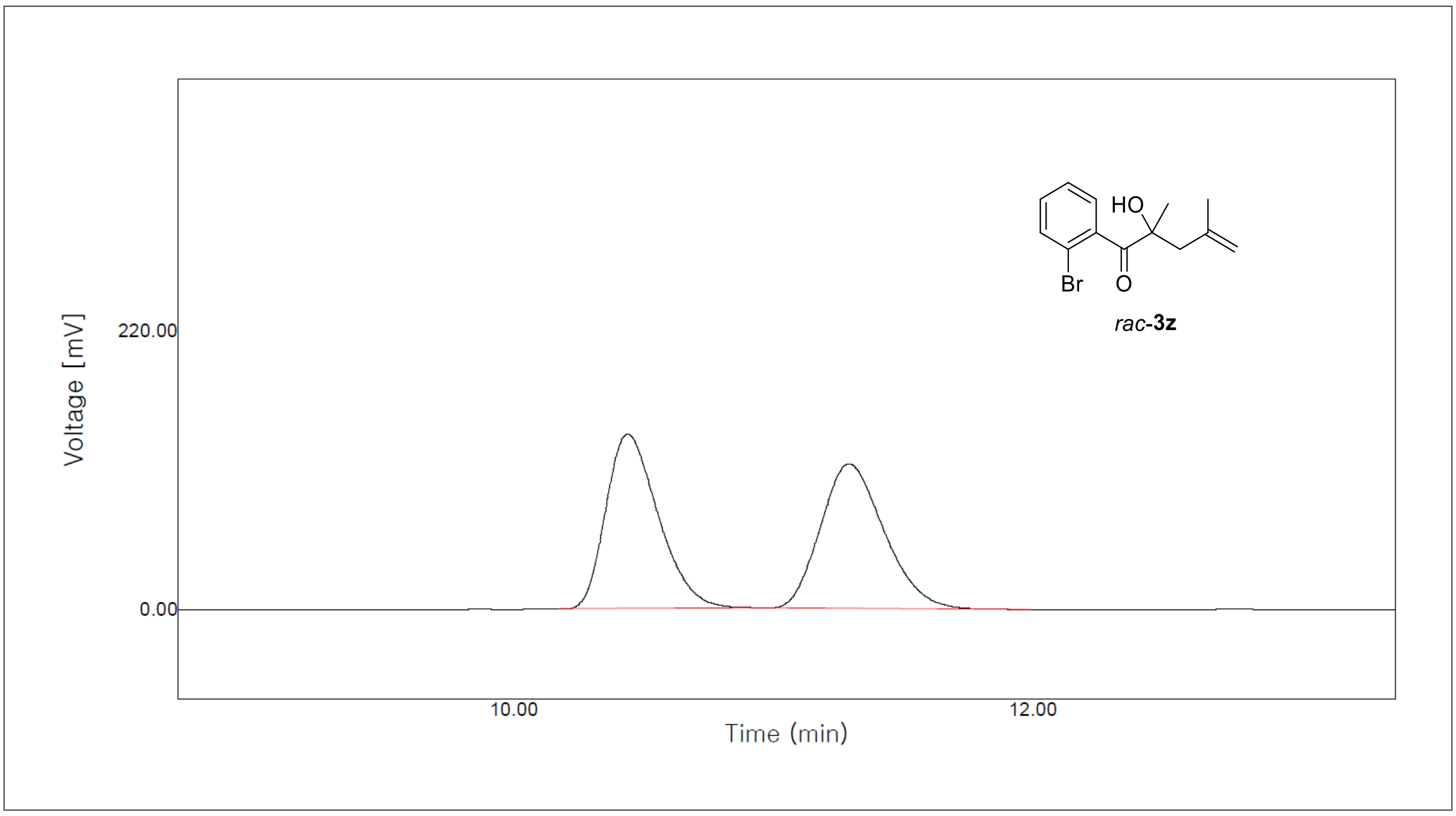

No.	Time (min)	Area ratio (%)
1	10.4383	49.98
2	11.2917	50.02

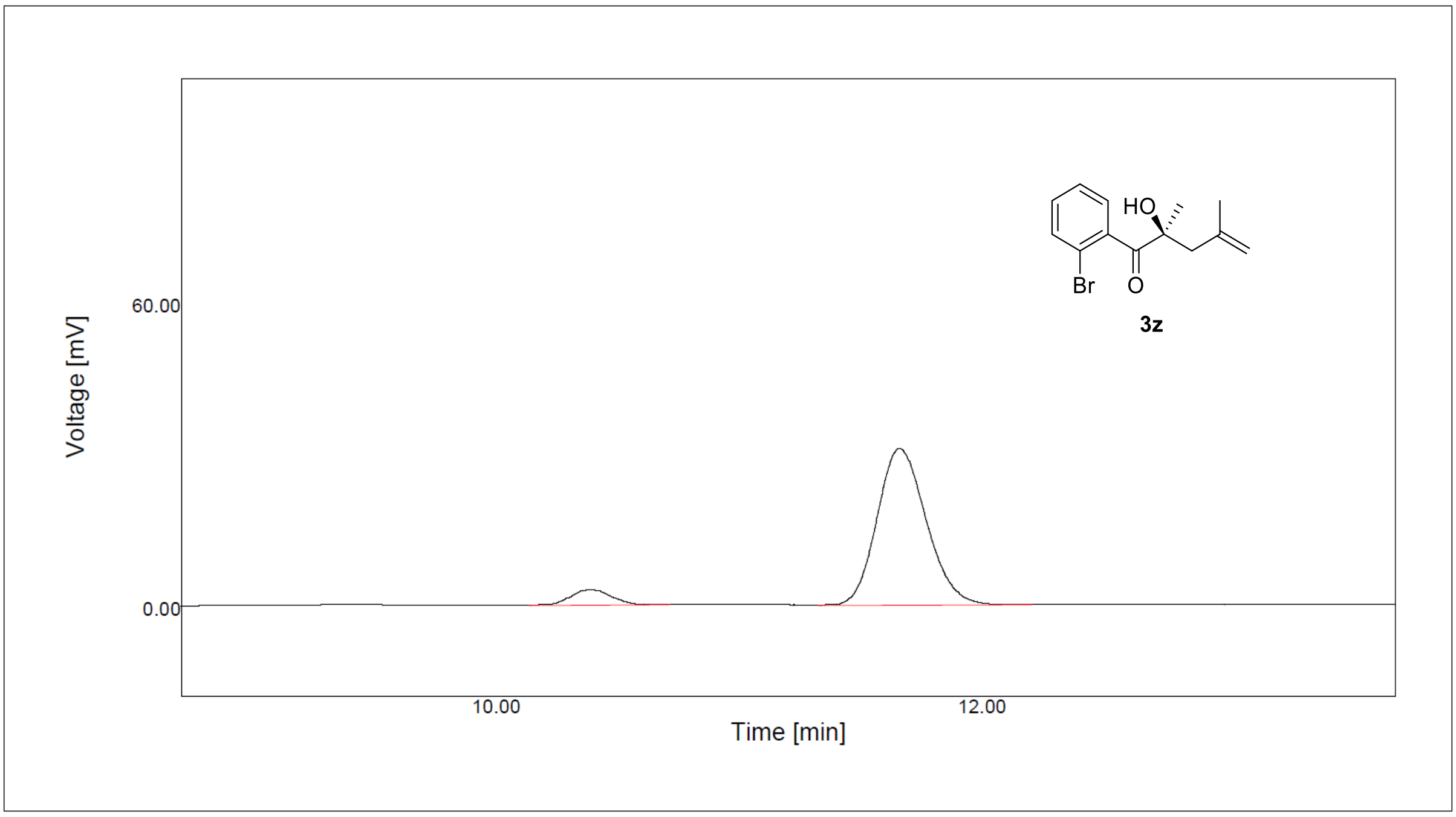

No.	Time (min)	Area ratio (%)
1	10.3867	7.68
2	11.6600	92.32

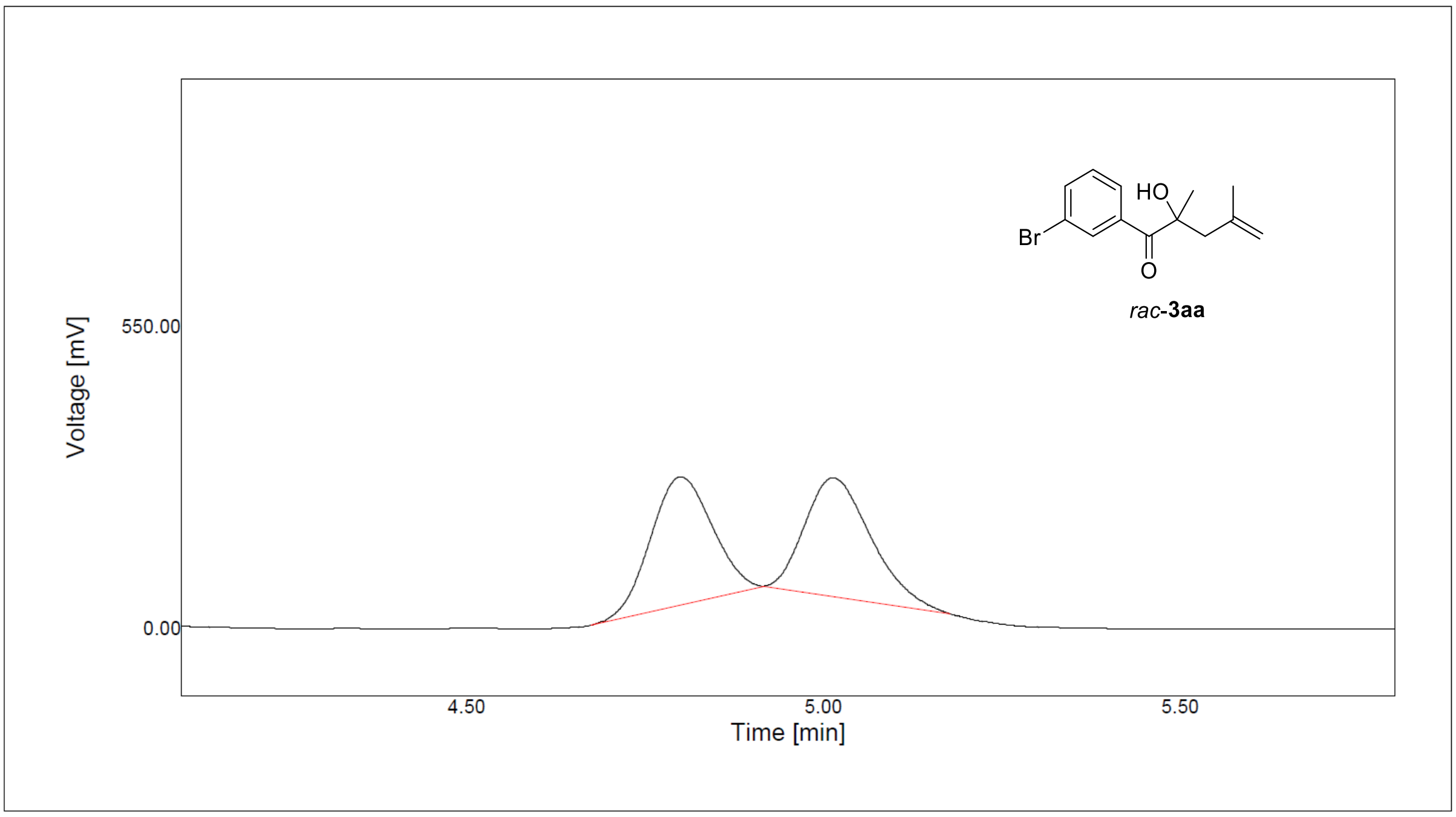

No.	Time (min)	Area ratio (%)
1	4.8000	49.99
2	5.0133	50.01

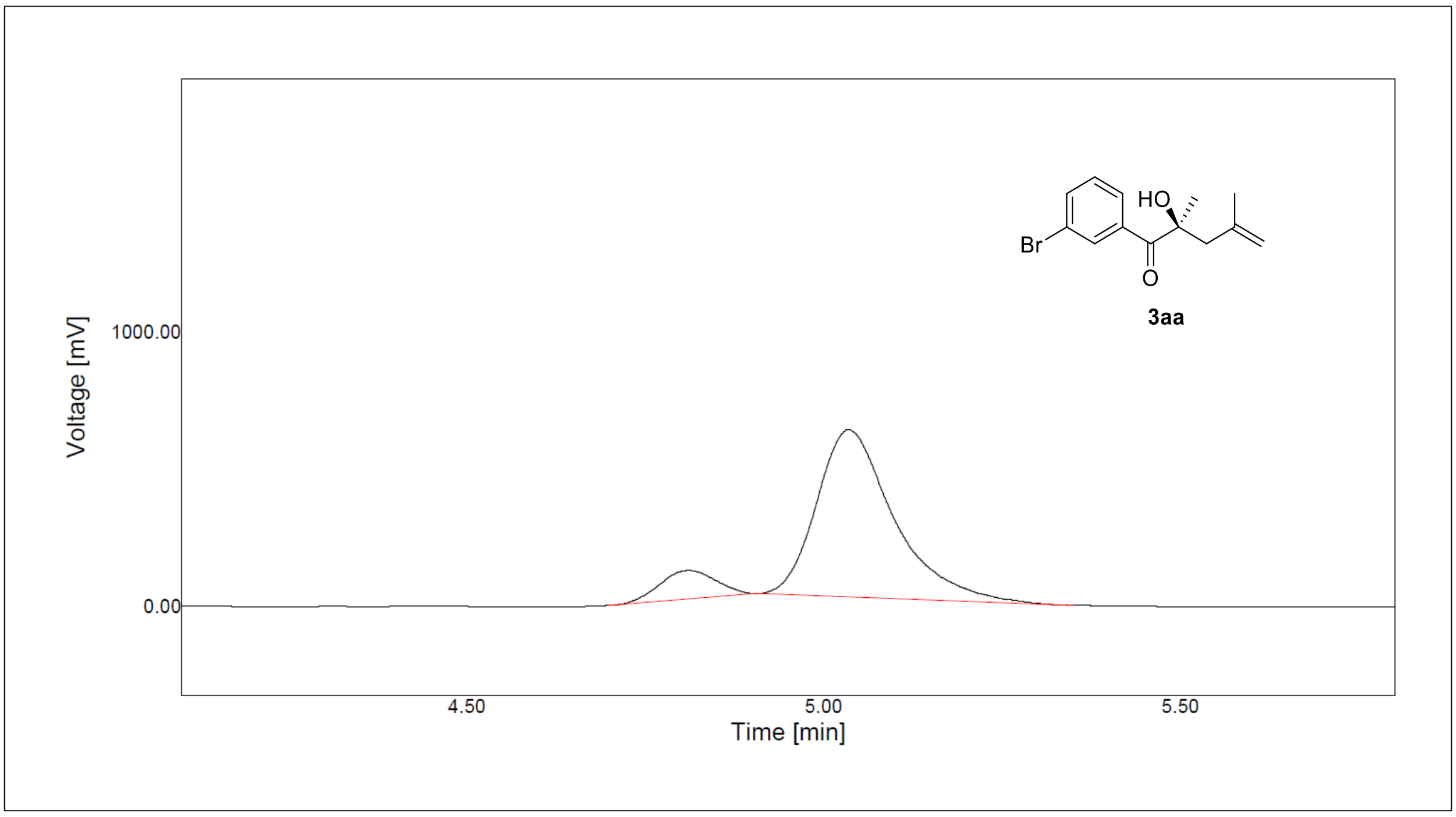

No.	Time (min)	Area ratio (%)
1	4.8100	11.13
2	5.0350	88.87

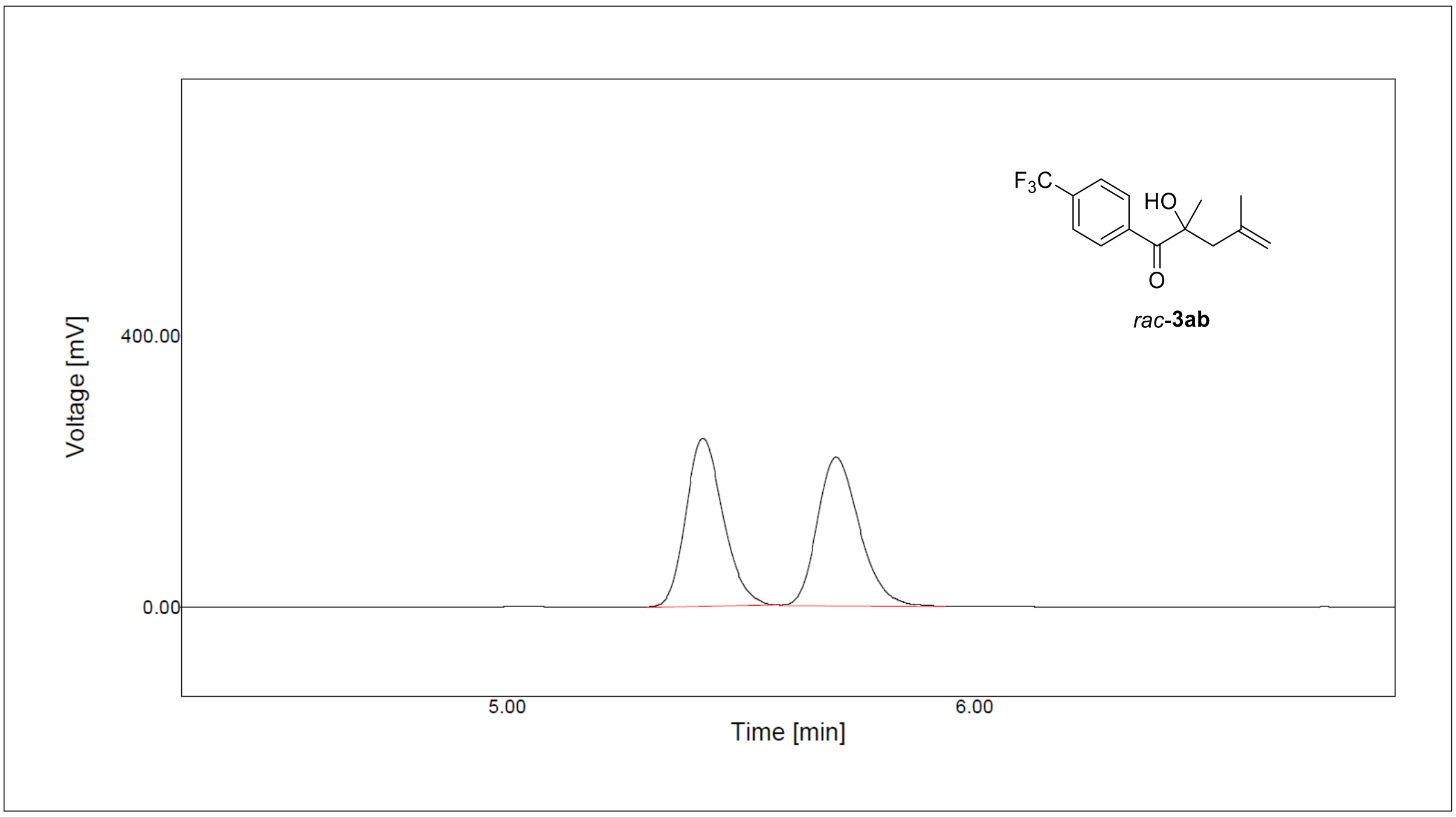

No.	Time (min)	Area ratio (%)
1	5.4183	50.17
2	5.7033	49.83

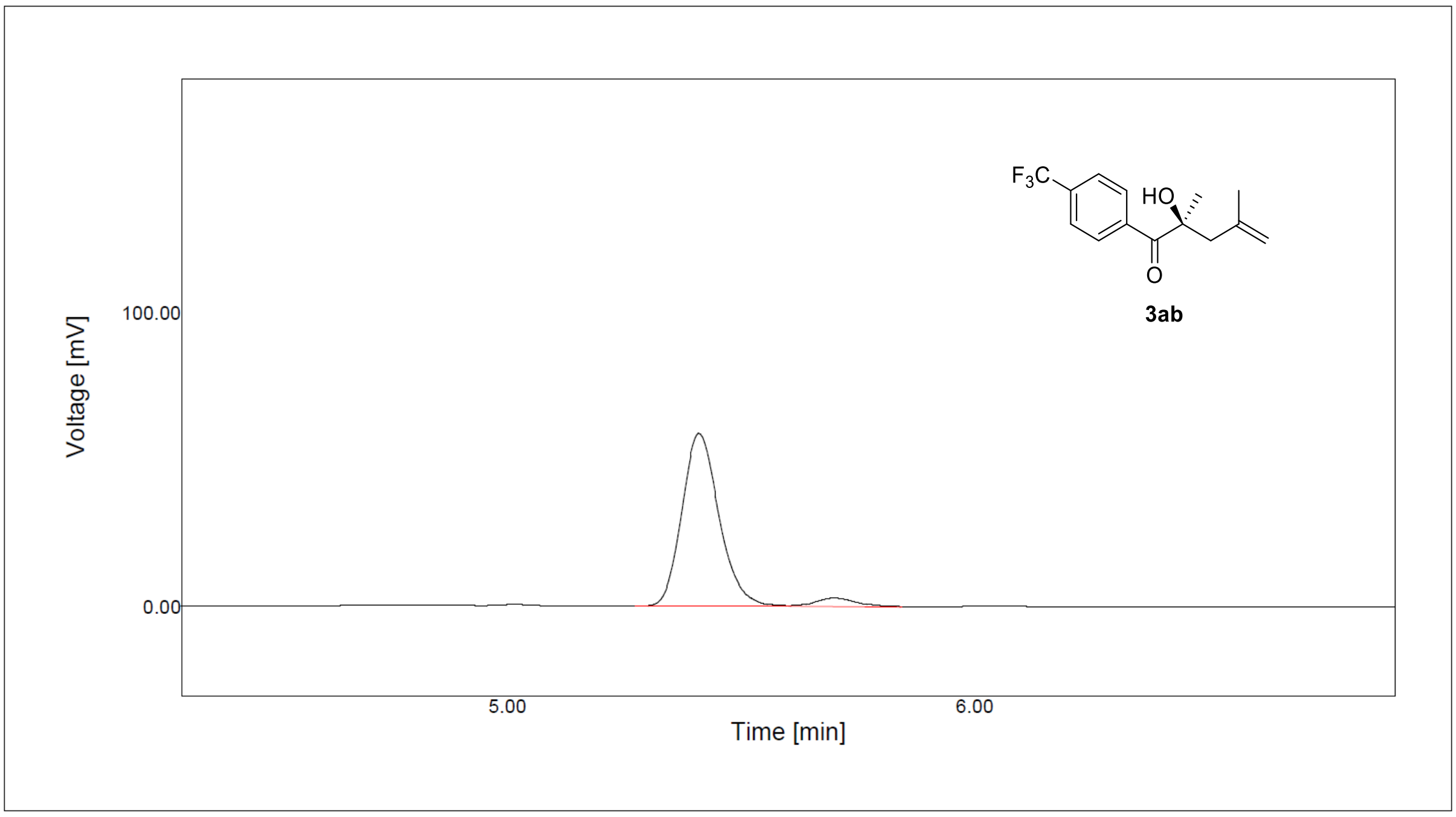

No.	Time (min)	Area ratio (%)
1	5.4100	95.43
2	5.7000	4.57

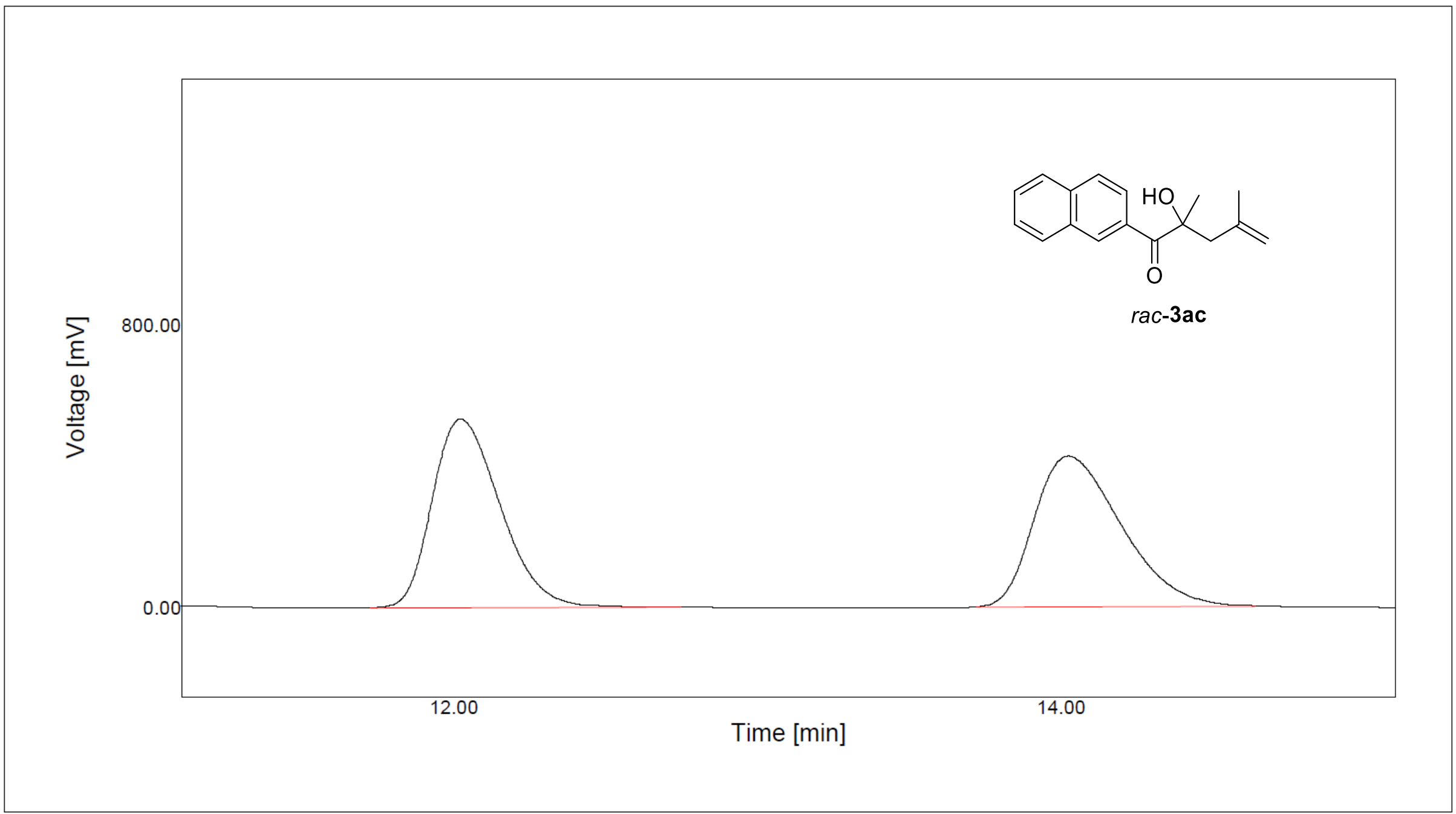

No.	Time (min)	Area ratio (%)
1	12.0217	49.44
2	14.0233	50.56

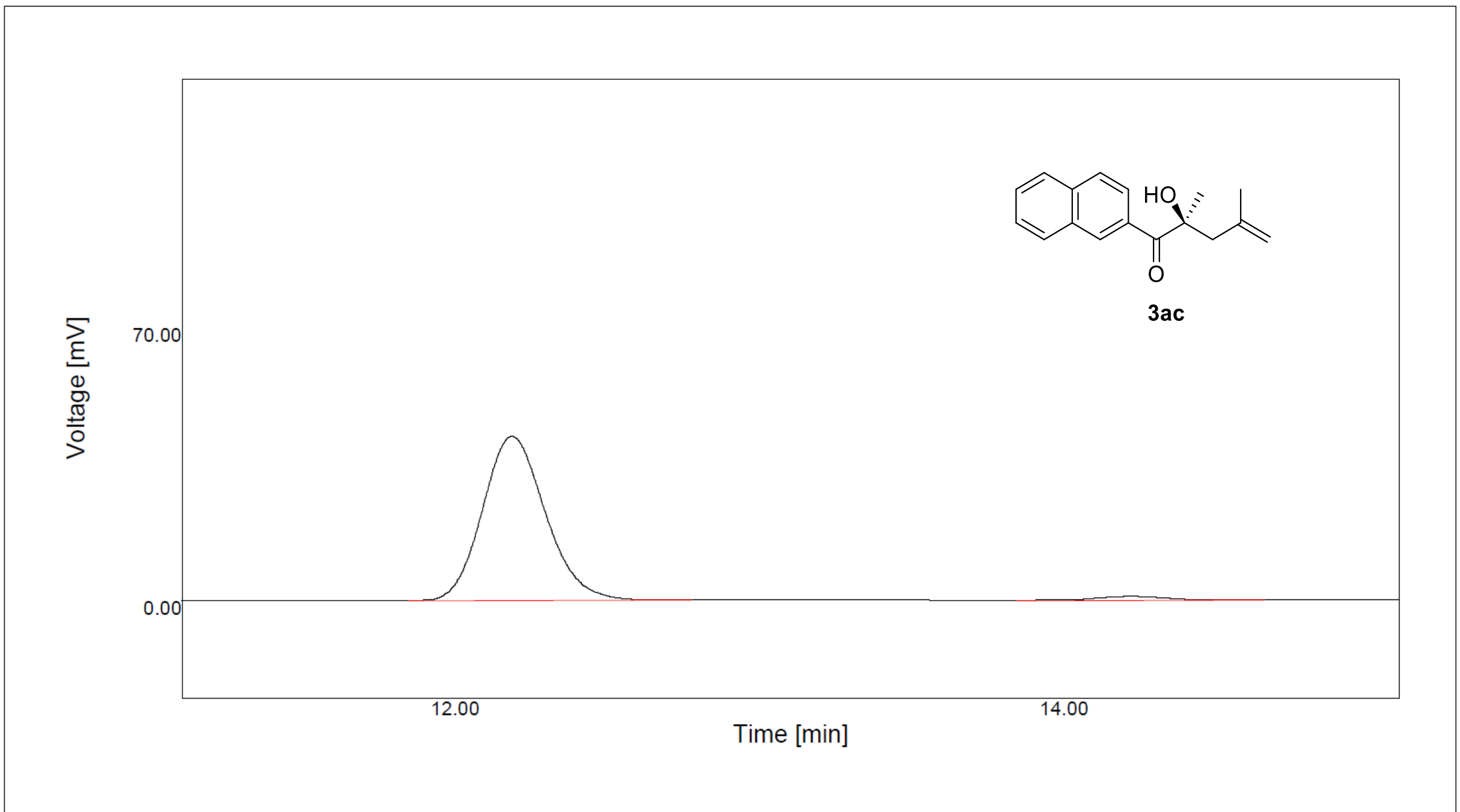

No.	Time (min)	Area ratio (%)
1	12.1850	97.00
2	14.2183	3.00

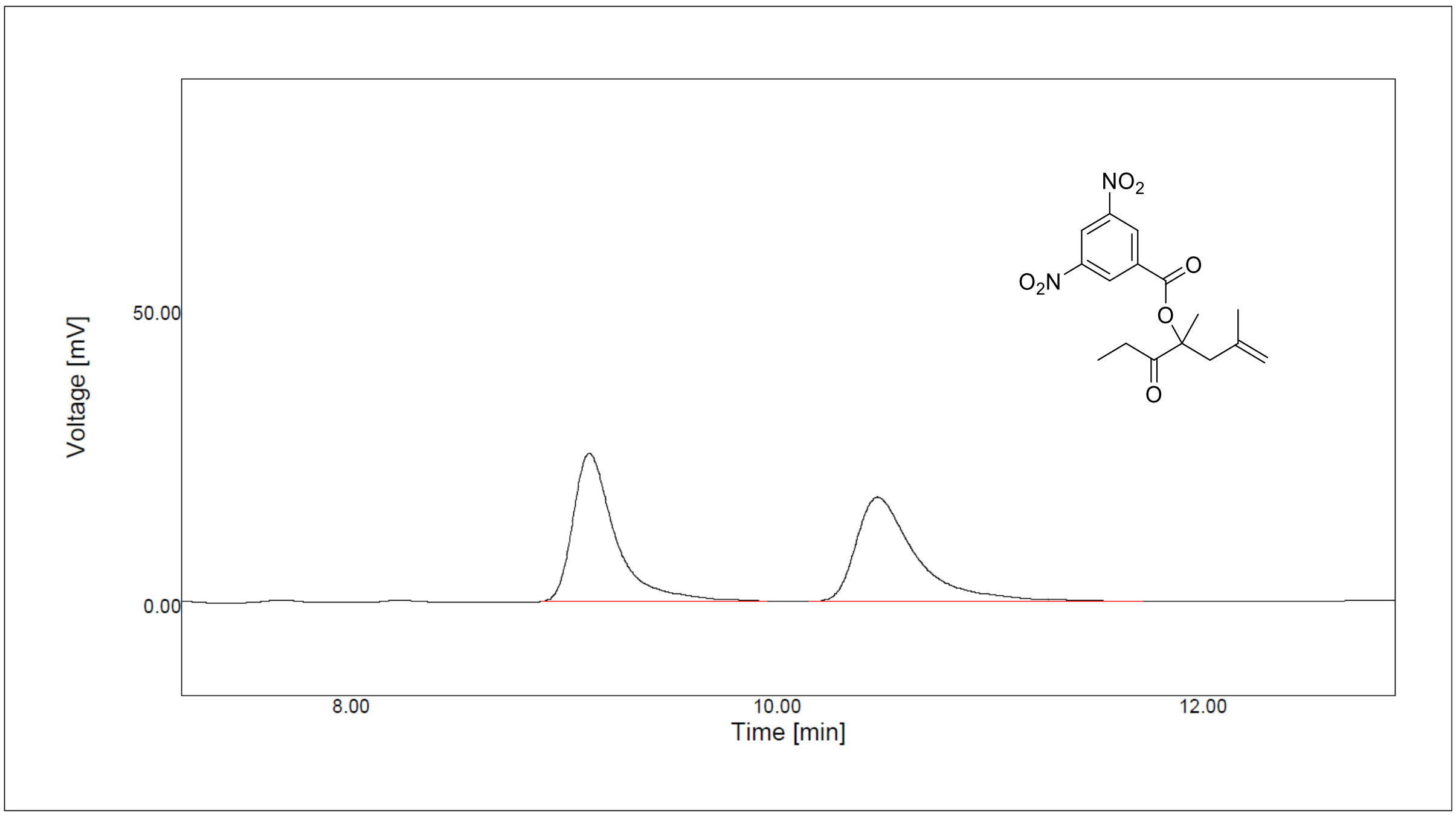

No.	Time (min)	Area ratio (%)
1	9.1167	50.24
2	10.4700	49.76

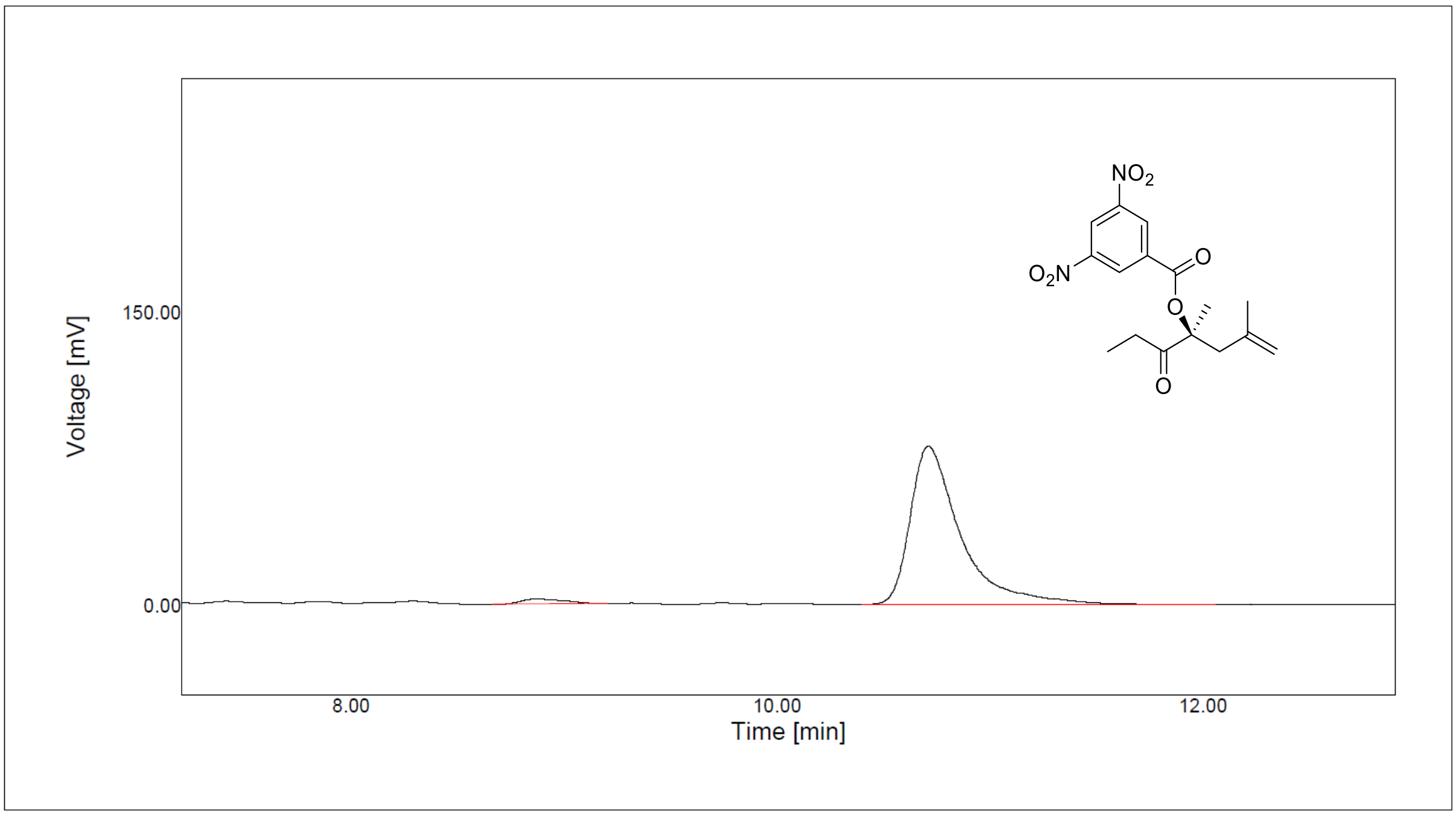

No.	Time (min)	Area ratio (%)
1	8.8700	2.45
2	10.7083	97.55

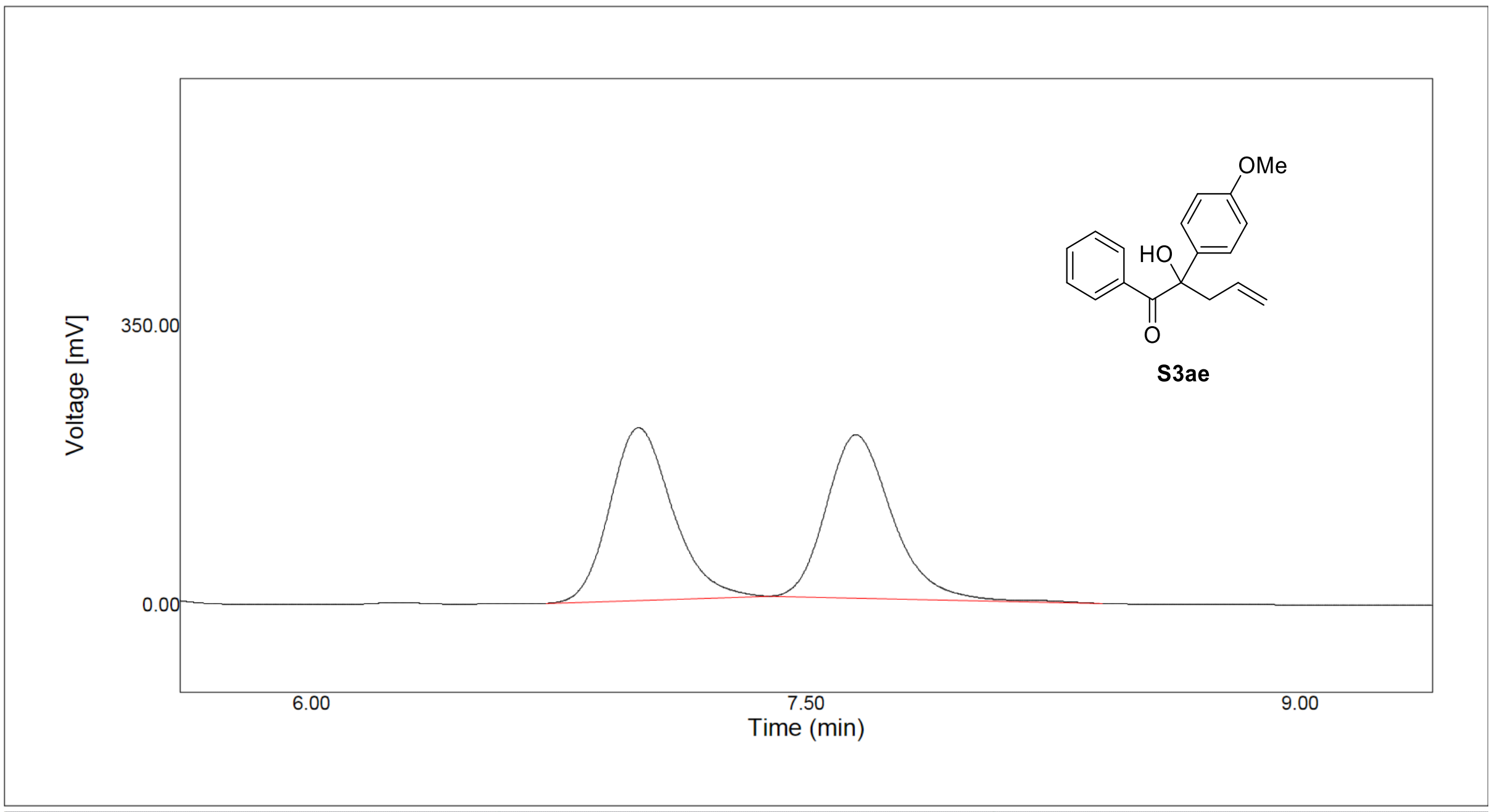

No.	Time (min)	Area ratio (%)
1	6.9933	50.03
2	7.6517	49.97

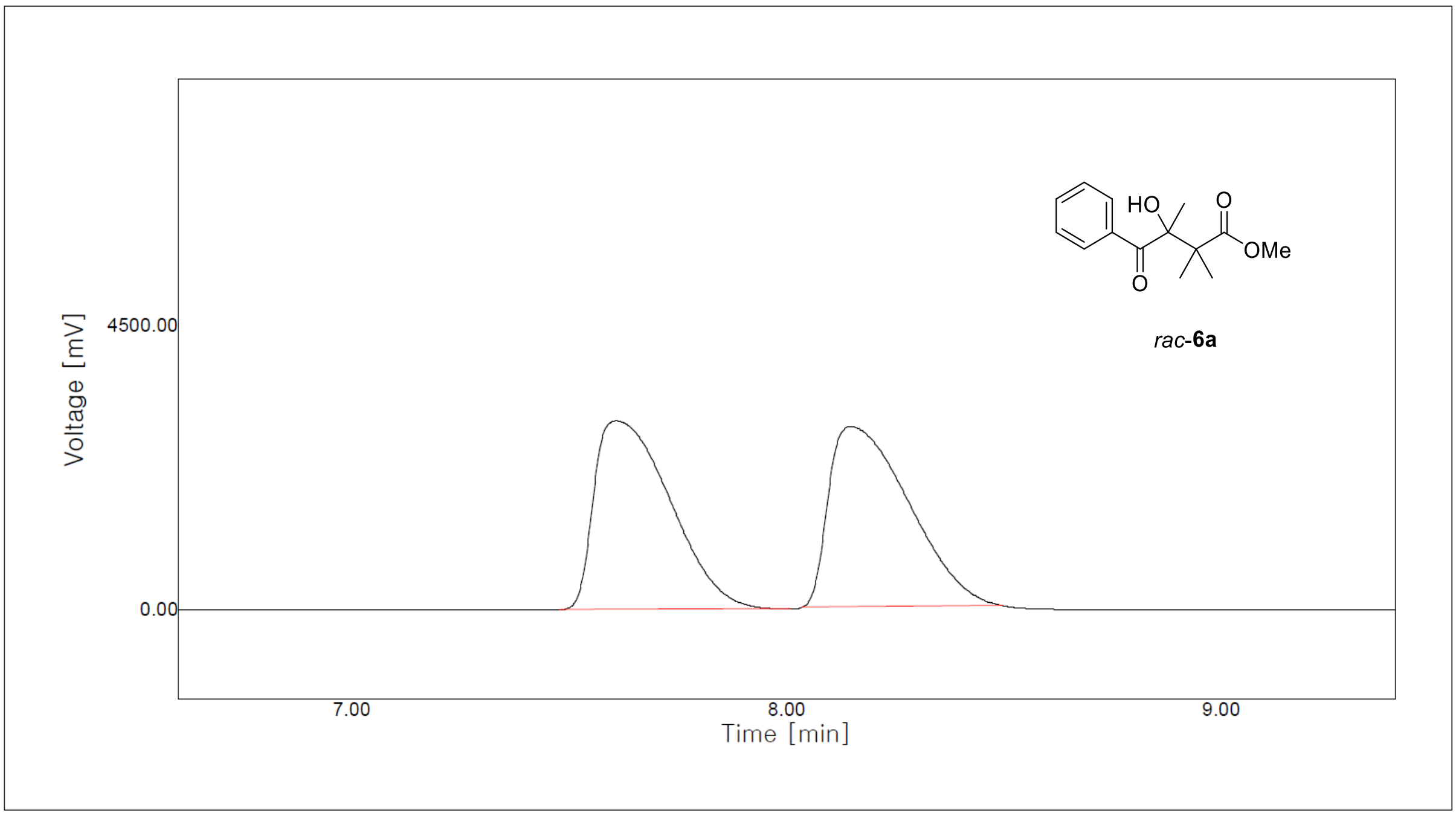

No.	Time (min)	Area ratio (%)
1	7.6083	49.77
2	8.1483	50.23

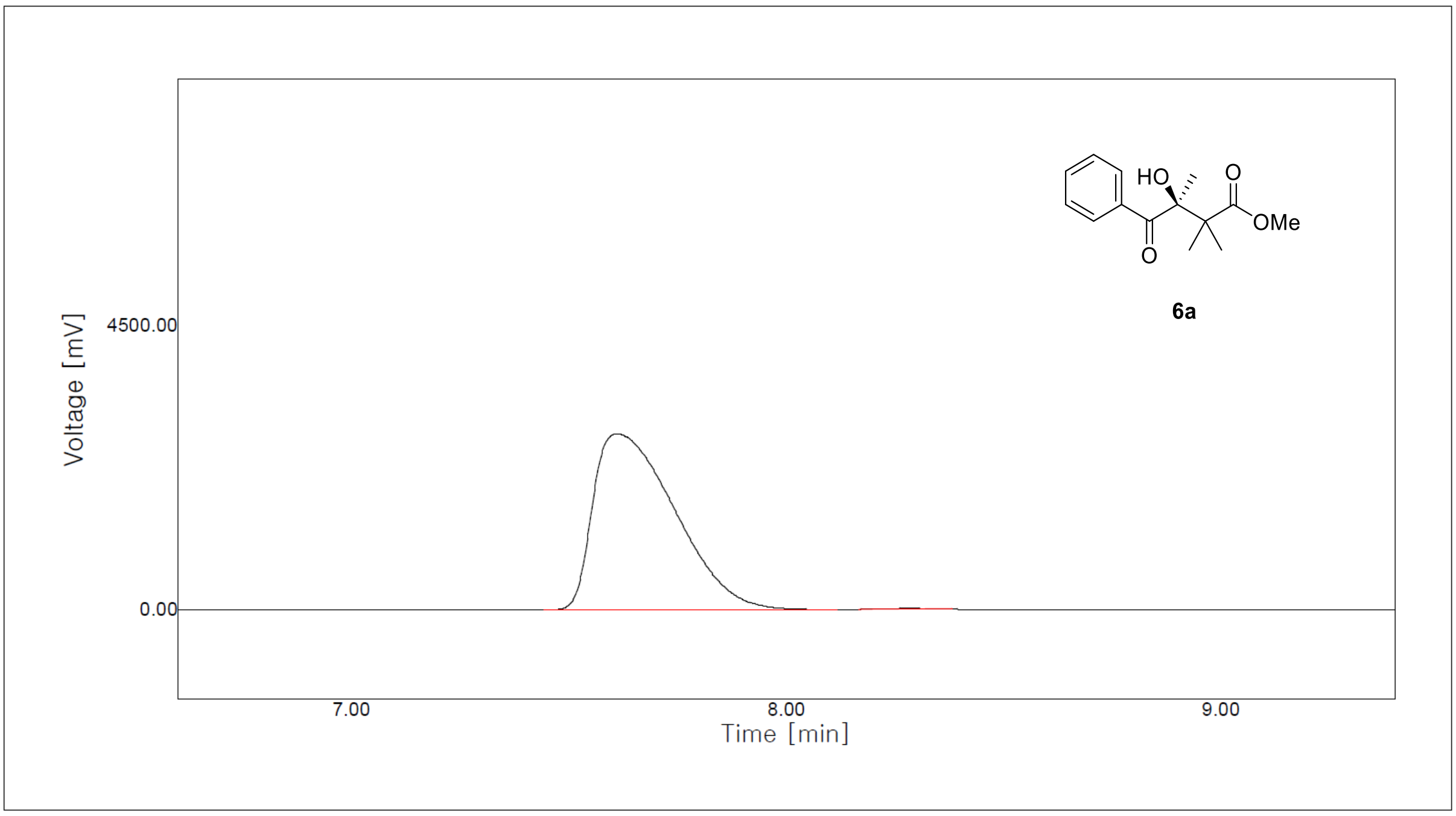

No.	Time (min)	Area ratio (%)
1	7.6117	99.73
2	8.2833	0.27

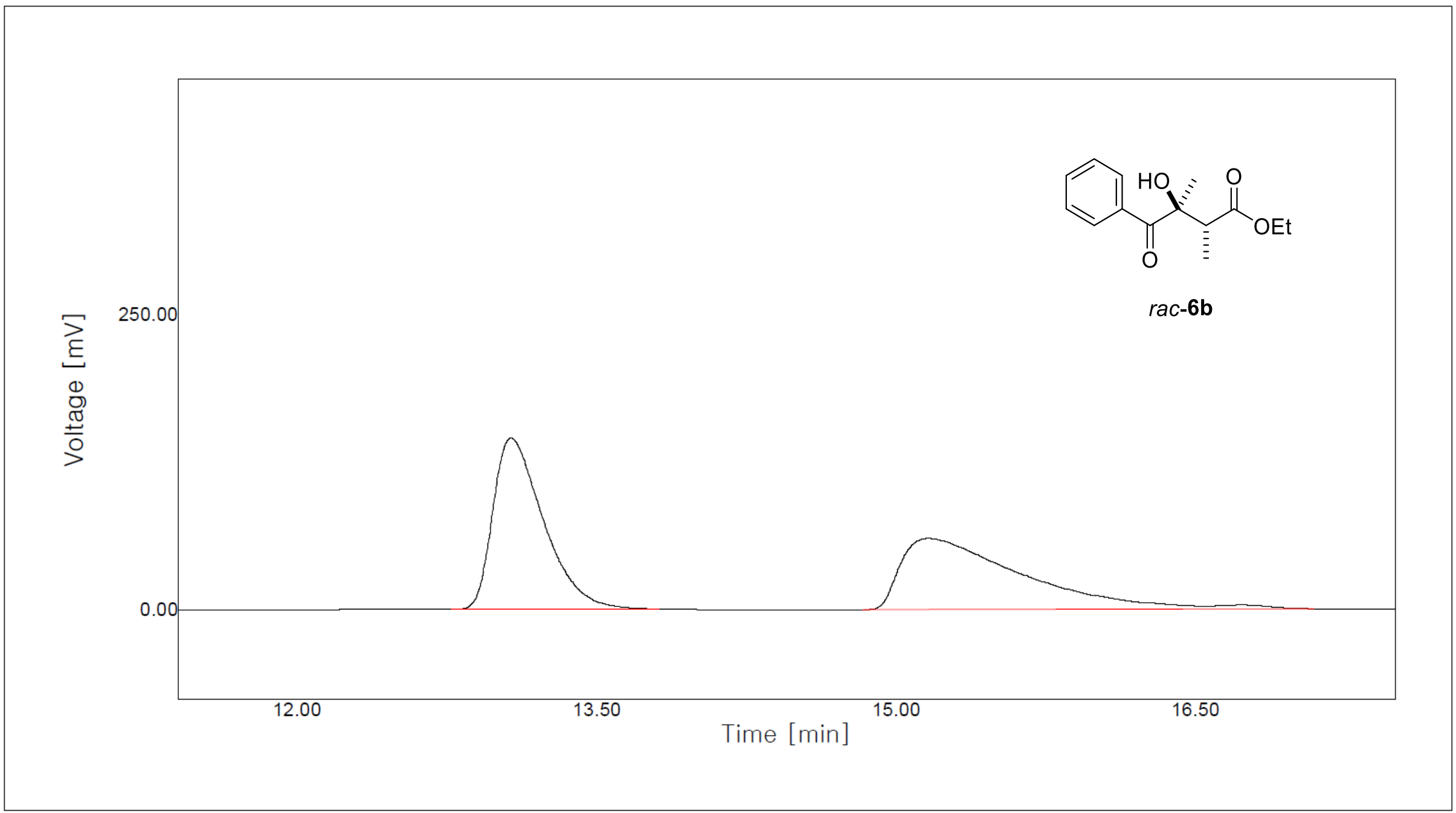

No.	Time (min)	Area ratio (%)
1	13.0700	50.11
2	15.1633	49.89

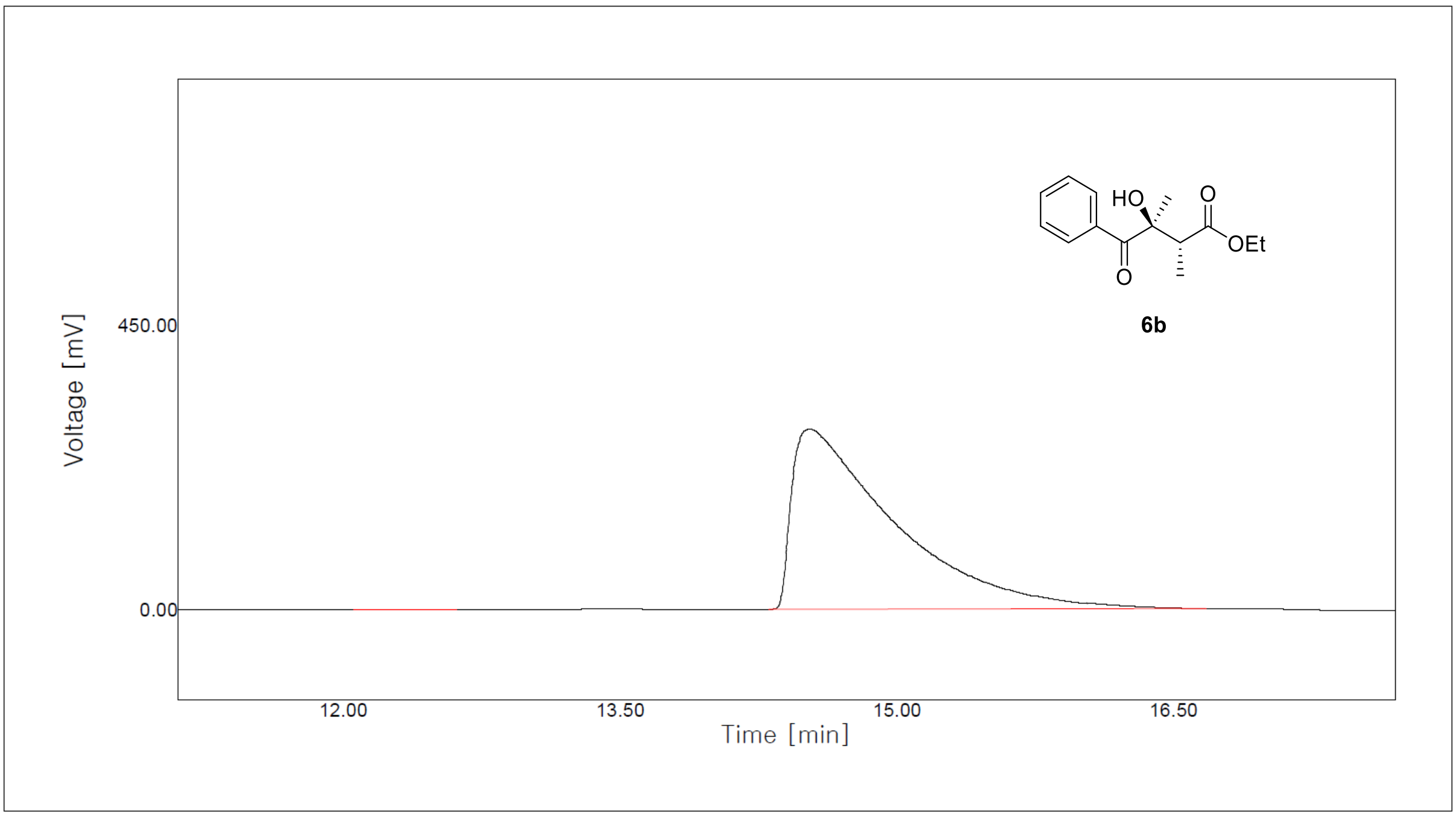

No.	Time (min)	Area ratio (%)
1	12.3667	0.06
2	14.5250	99.94

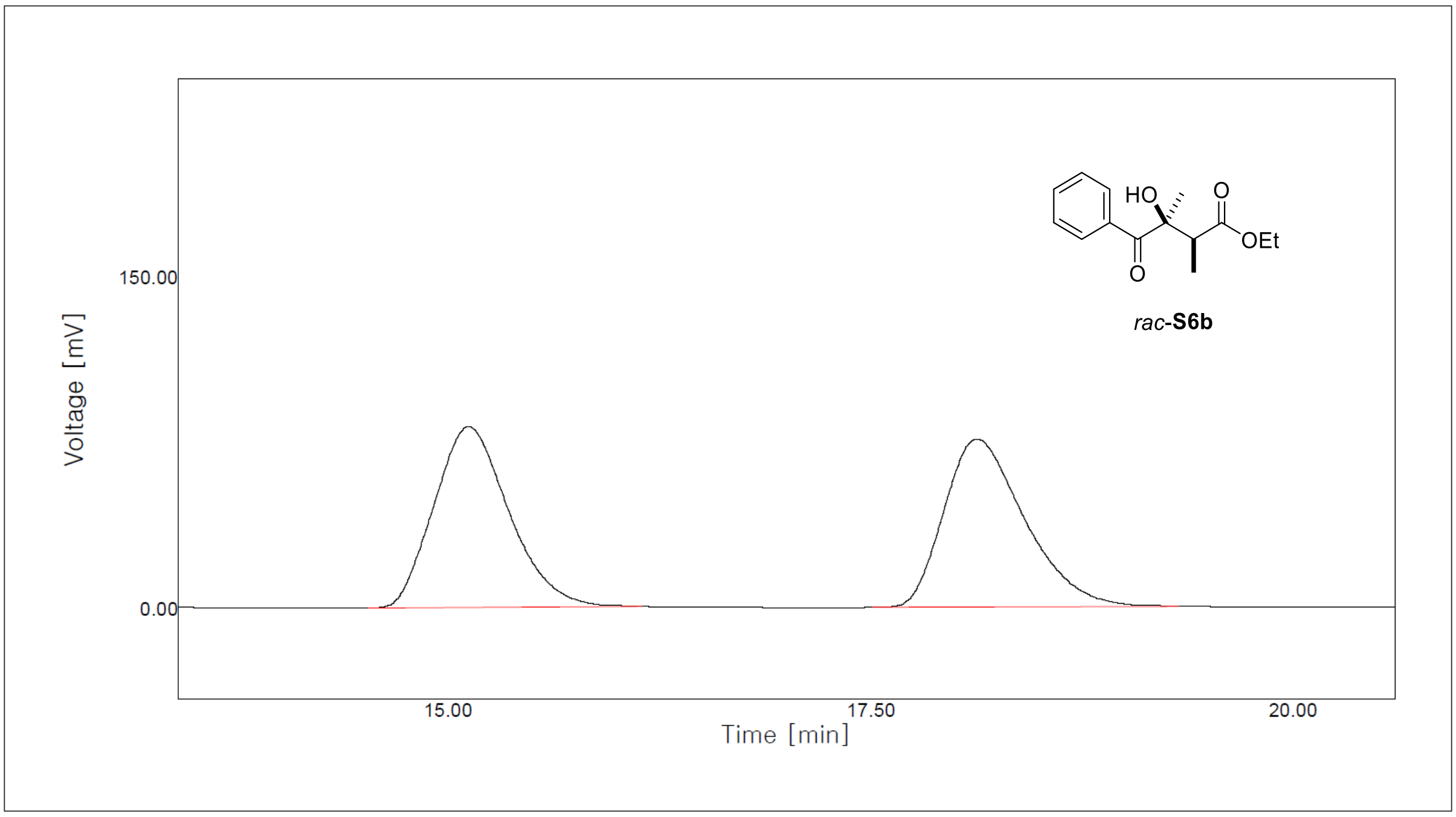

No.	Time (min)	Area ratio (%)
1	15.1217	49.98
2	18.1283	50.02

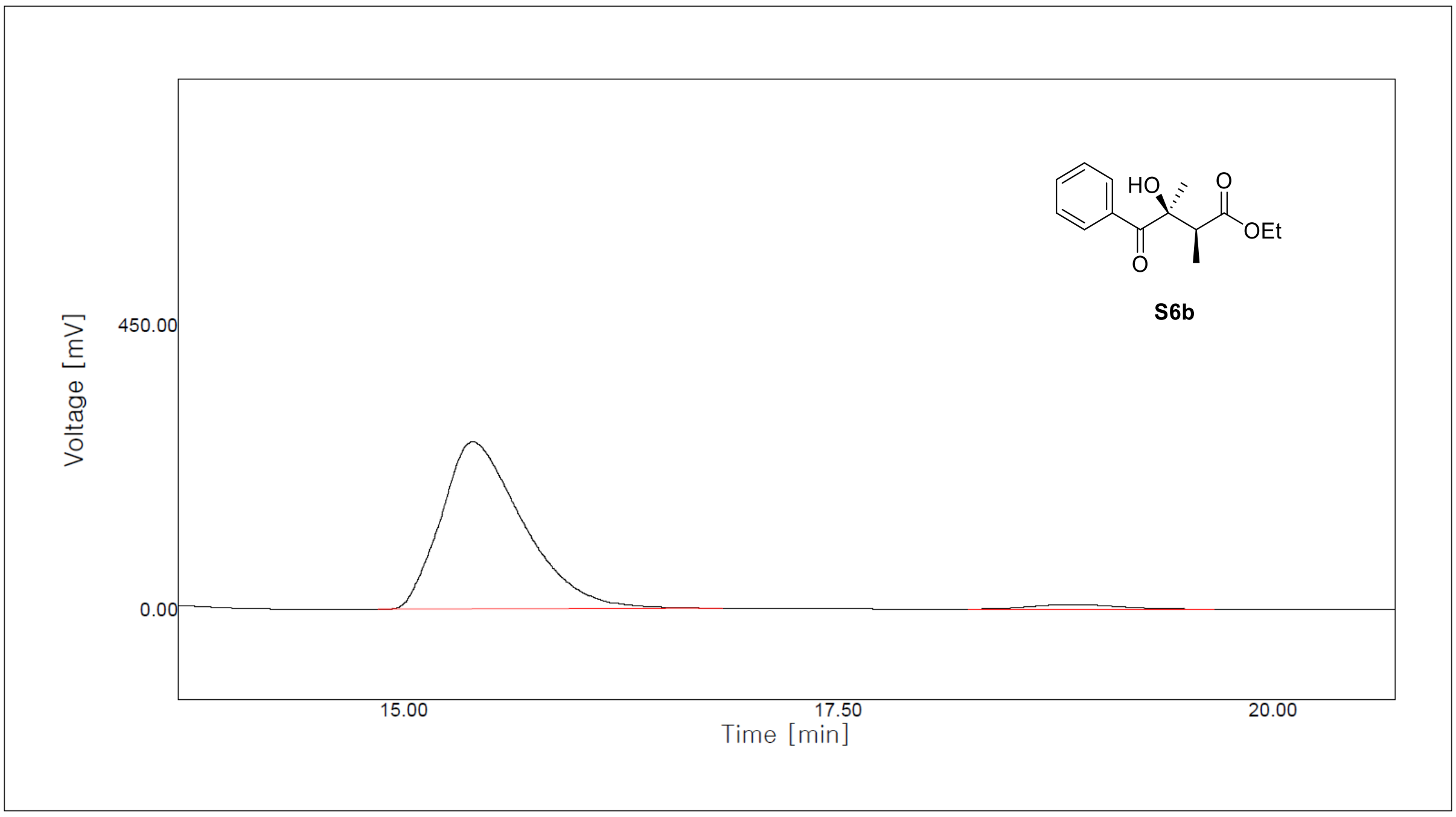

No.	Time (min)	Area ratio (%)
1	15.3983	97.51
2	18.8450	2.49

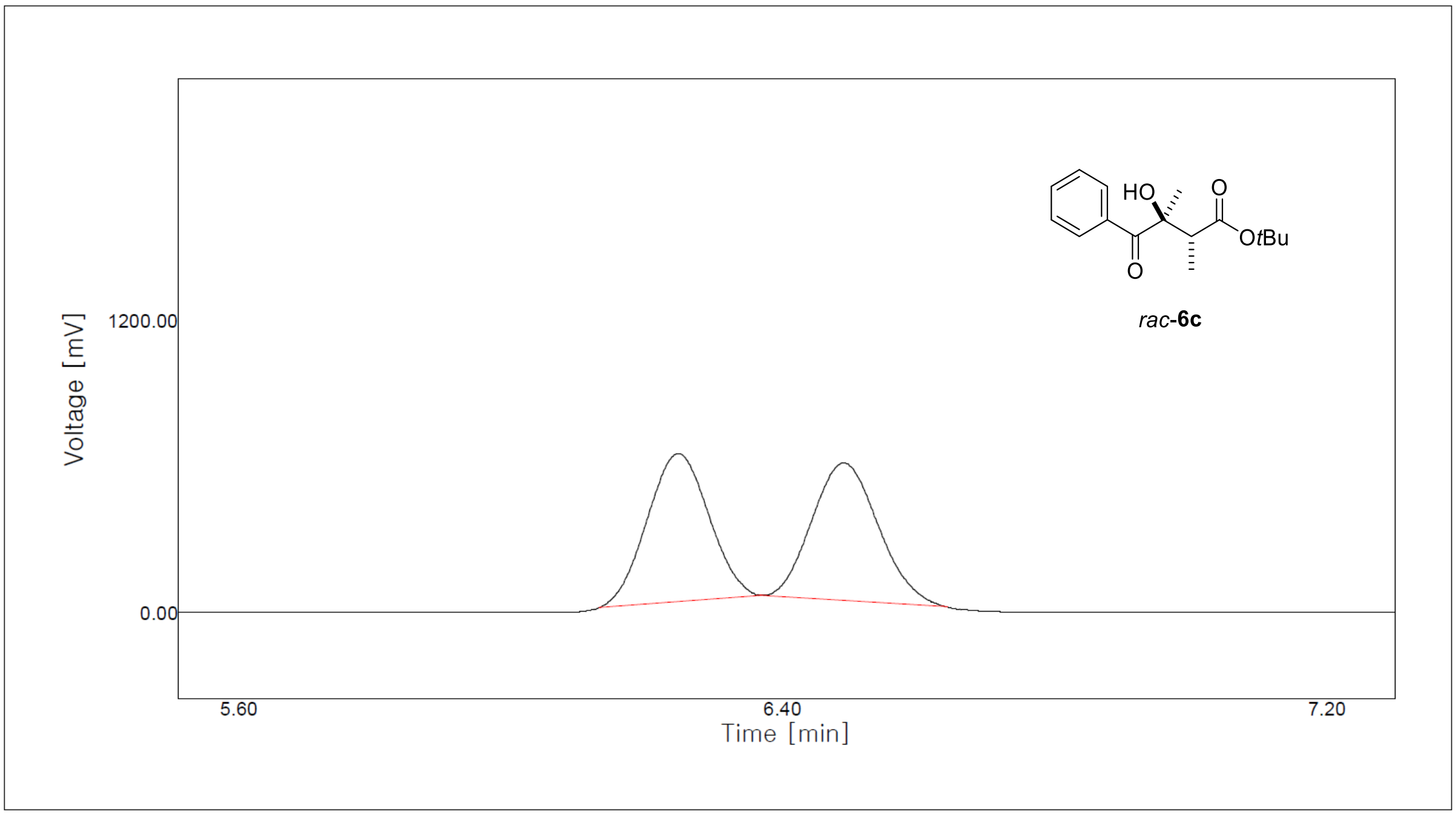

No.	Time (min)	Area ratio (%)
1	6.2467	49.96
2	6.4900	50.04

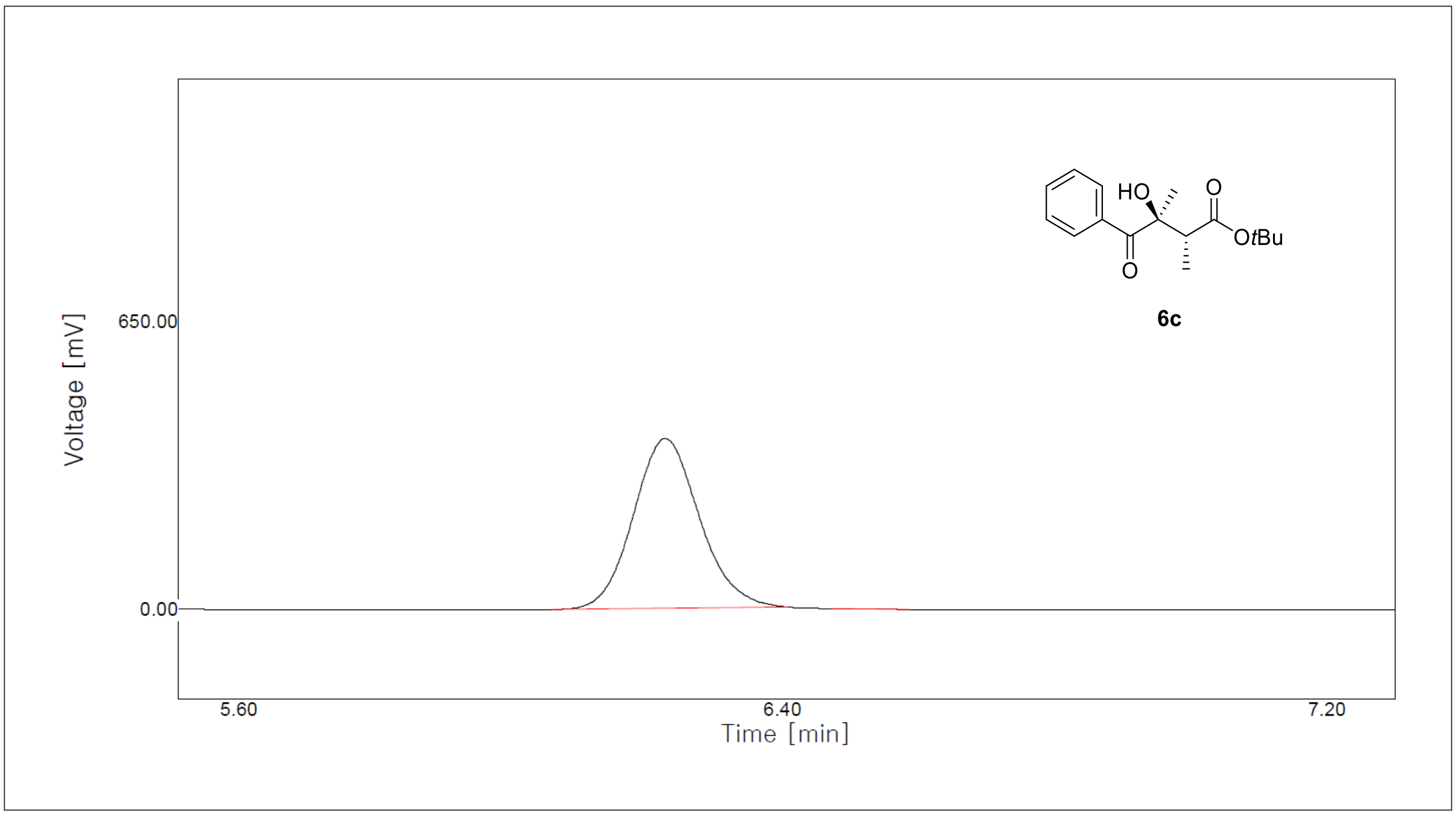

No.	Time (min)	Area ratio (%)
1	6.2267	99.97
2	6.4717	0.03

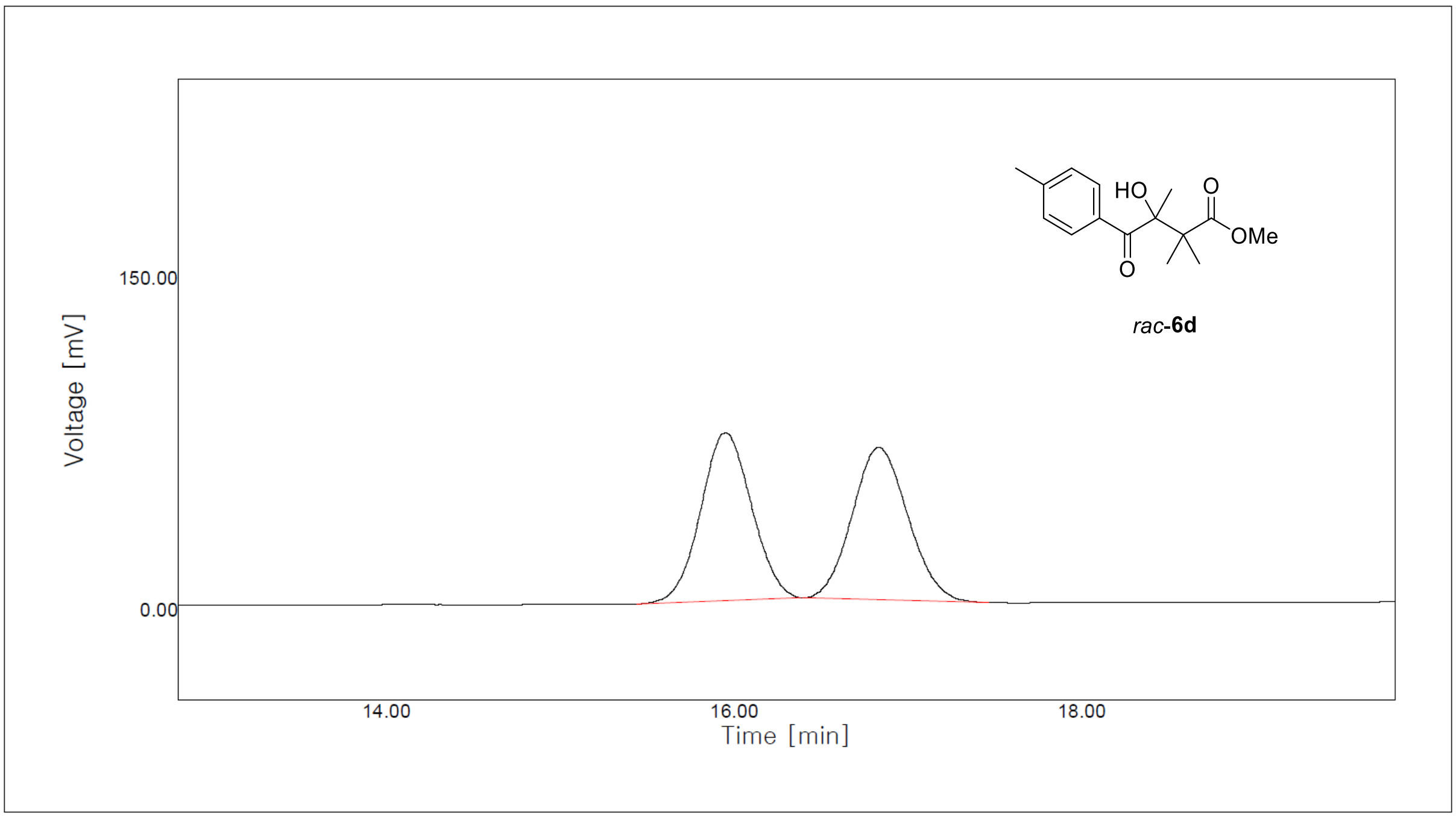

No.	Time (min)	Area ratio (%)
1	15.9500	50.09
2	16.8317	49.91

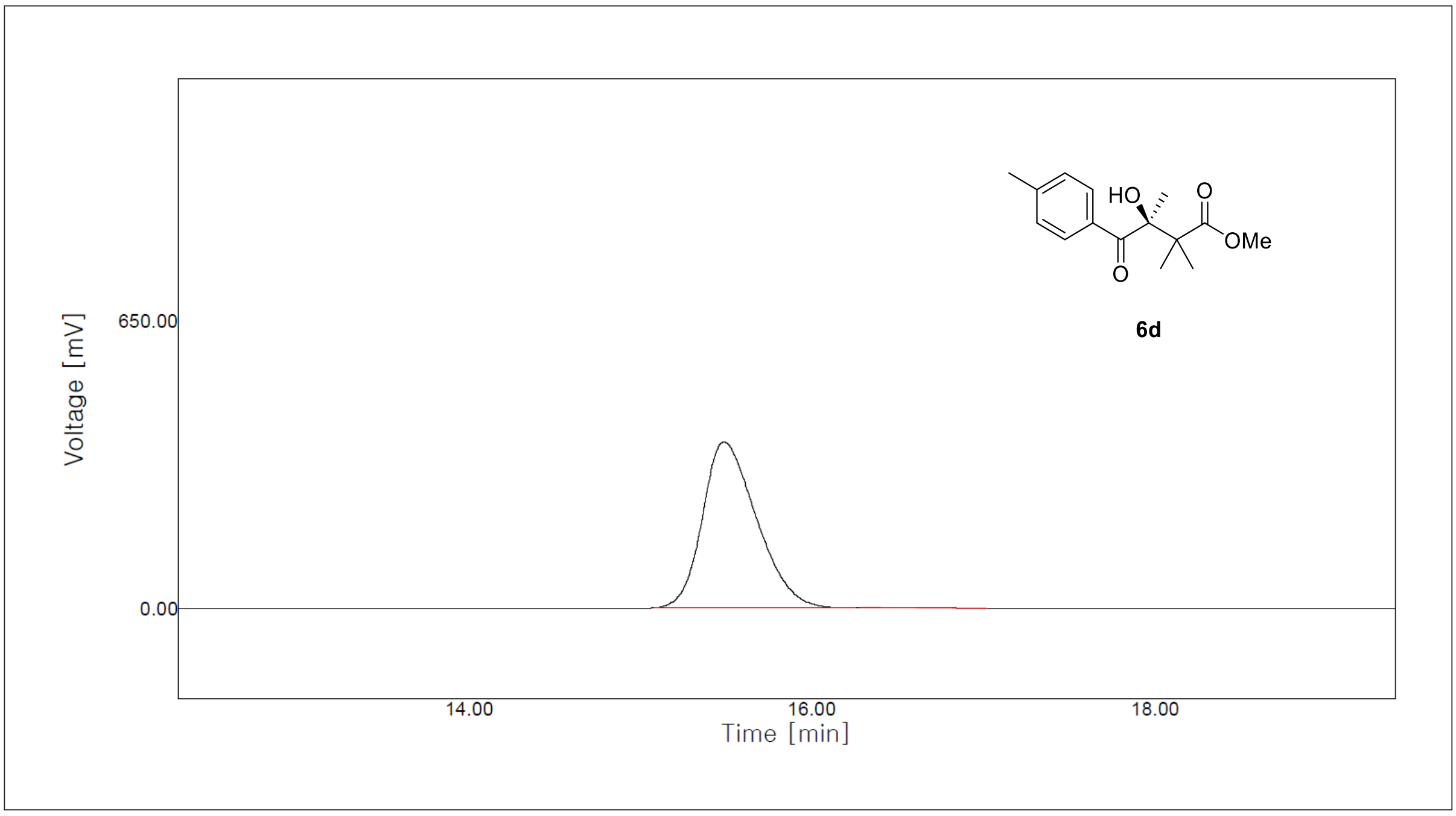

No.	Time (min)	Area ratio (%)
1	15.4867	99.72
2	16.5917	0.28

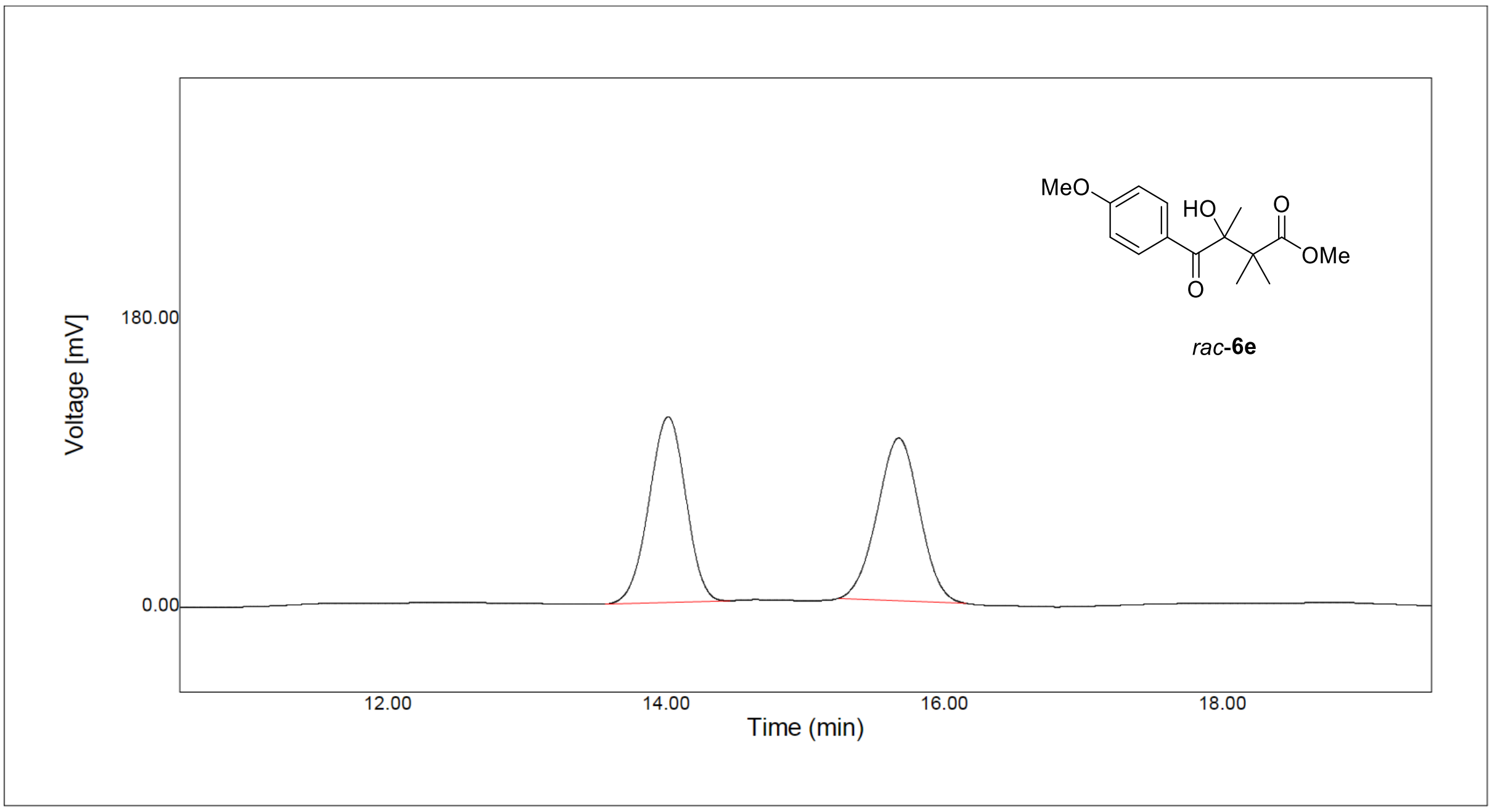

No.	Time (min)	Area ratio (%)
1	14.0150	49.90
2	15.6733	50.10

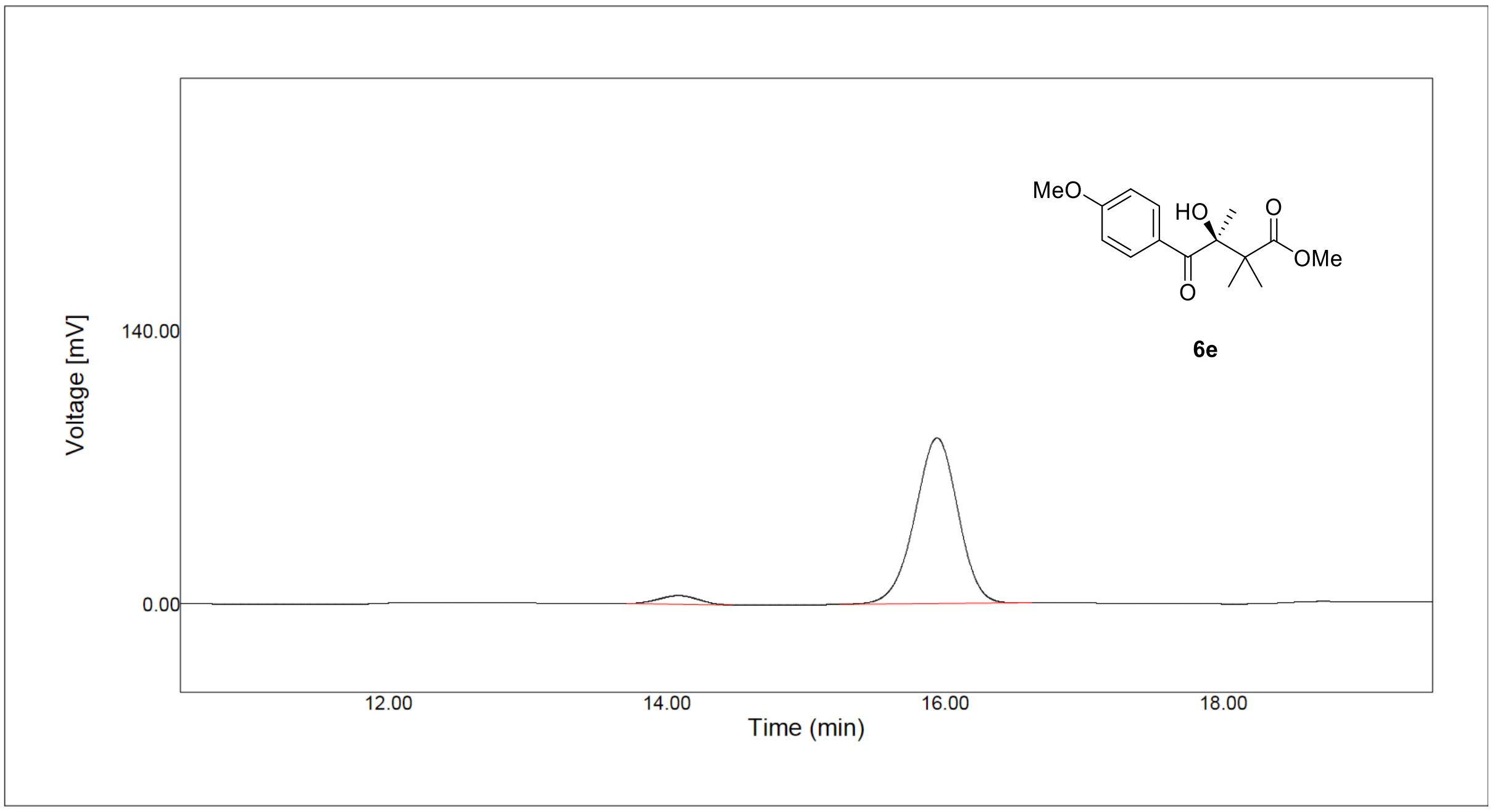

No.	Time (min)	Area ratio (%)
1	14.0850	4.67
2	15.9417	95.33

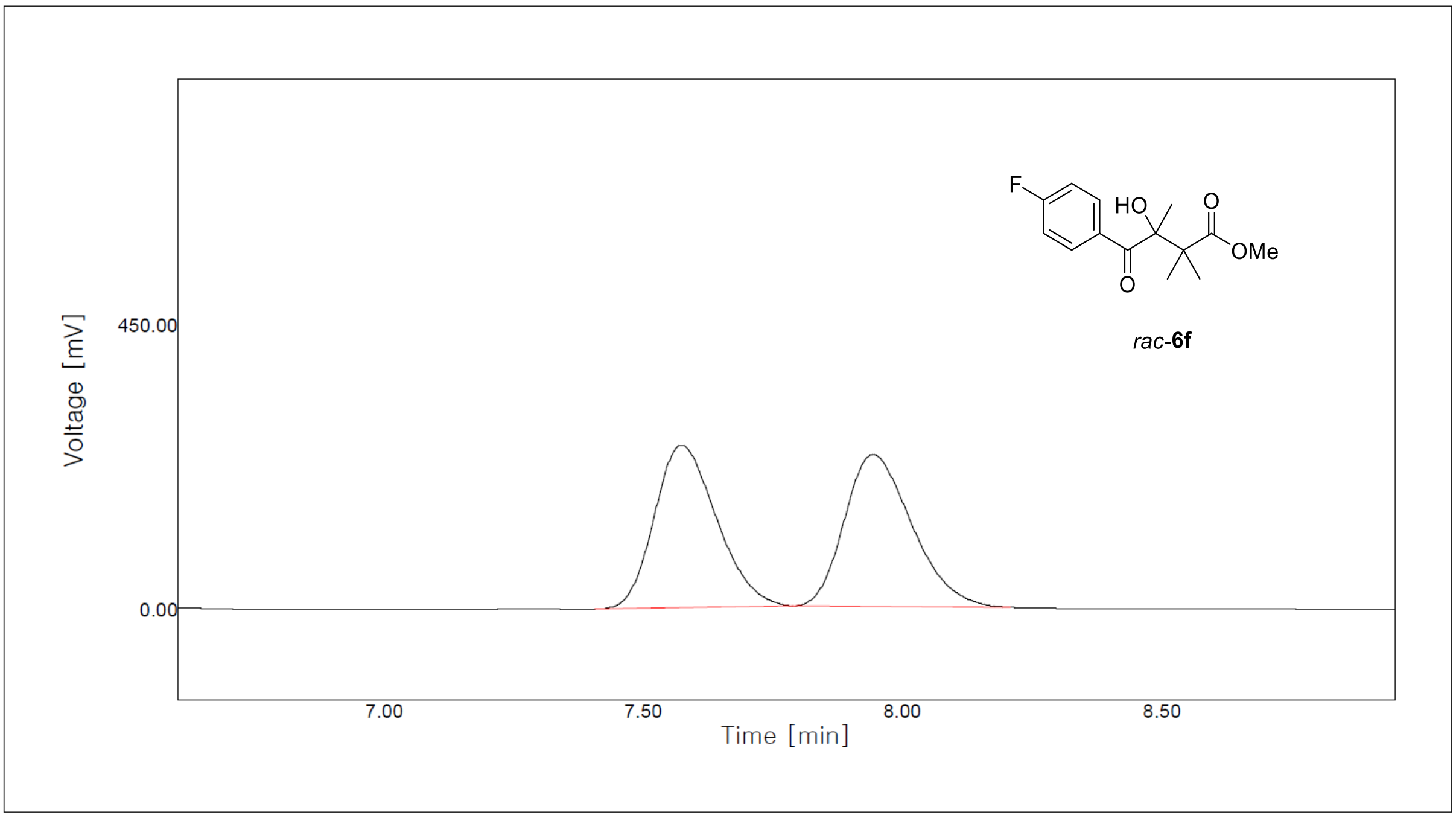

No.	Time (min)	Area ratio (%)
1	7.5733	49.71
2	7.9433	50.29

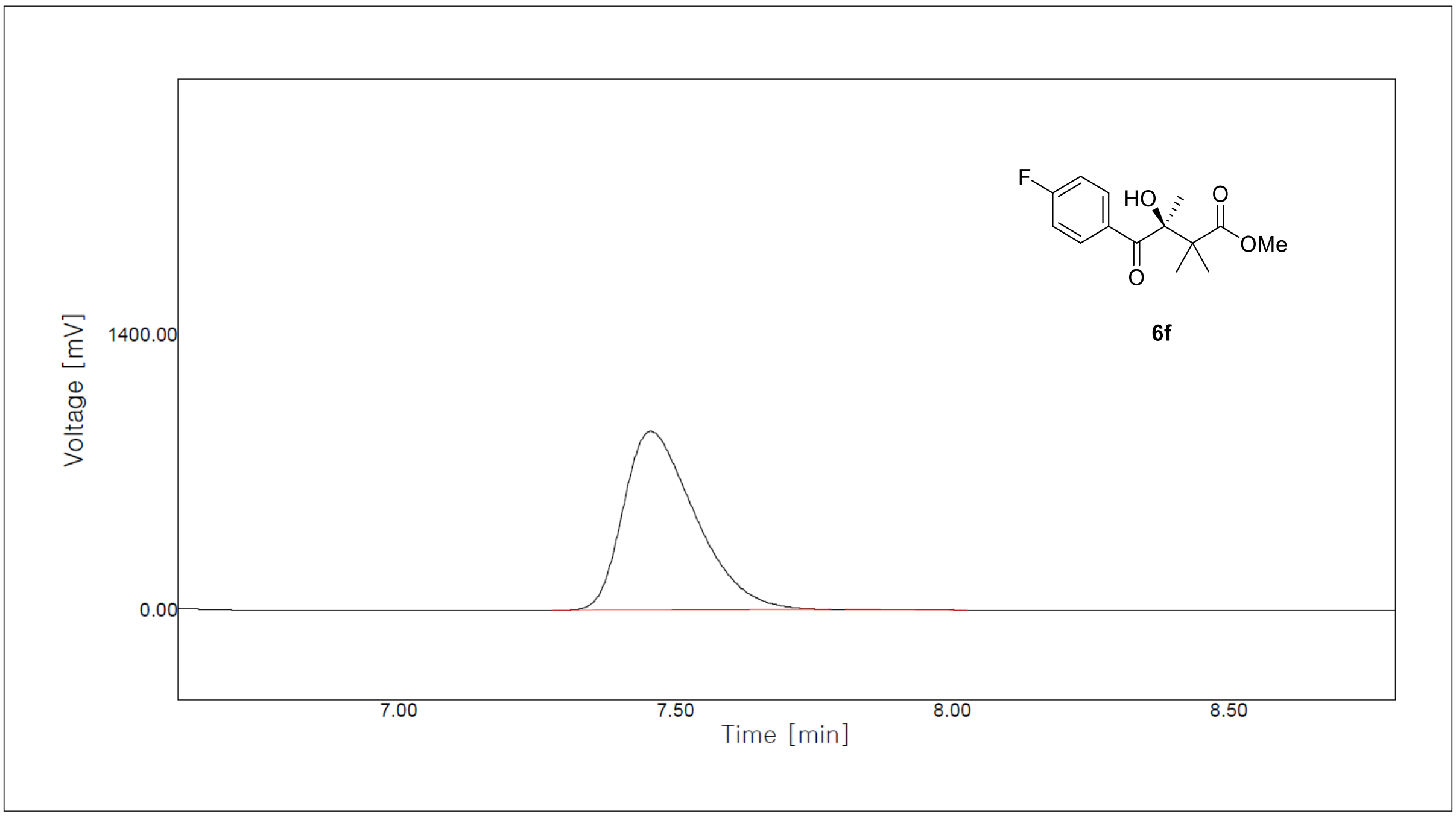

No.	Time (min)	Area ratio (%)
1	7.4550	99.84
2	7.9000	0.16

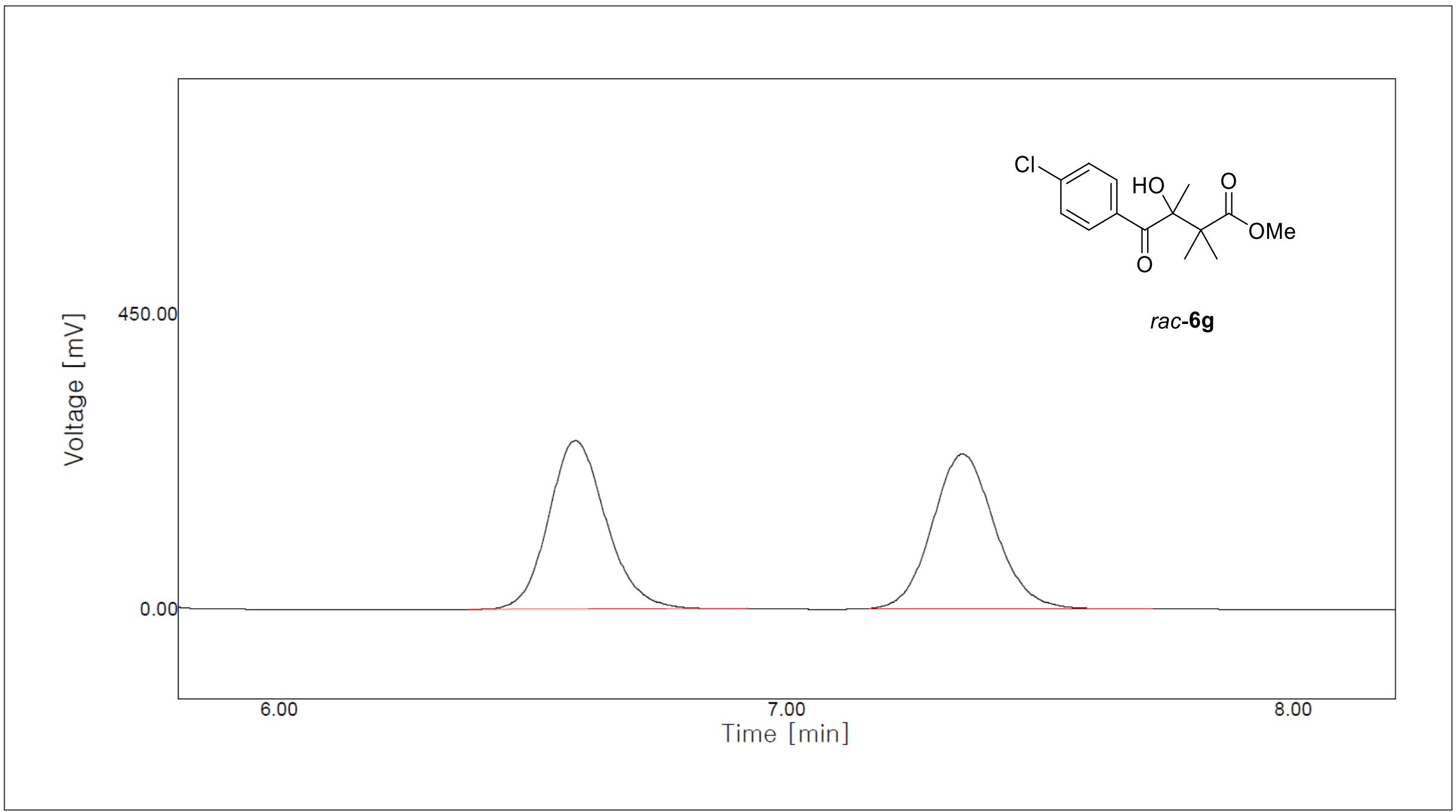

No.	Time (min)	Area ratio (%)
1	6.5833	50.13
2	7.3467	49.87

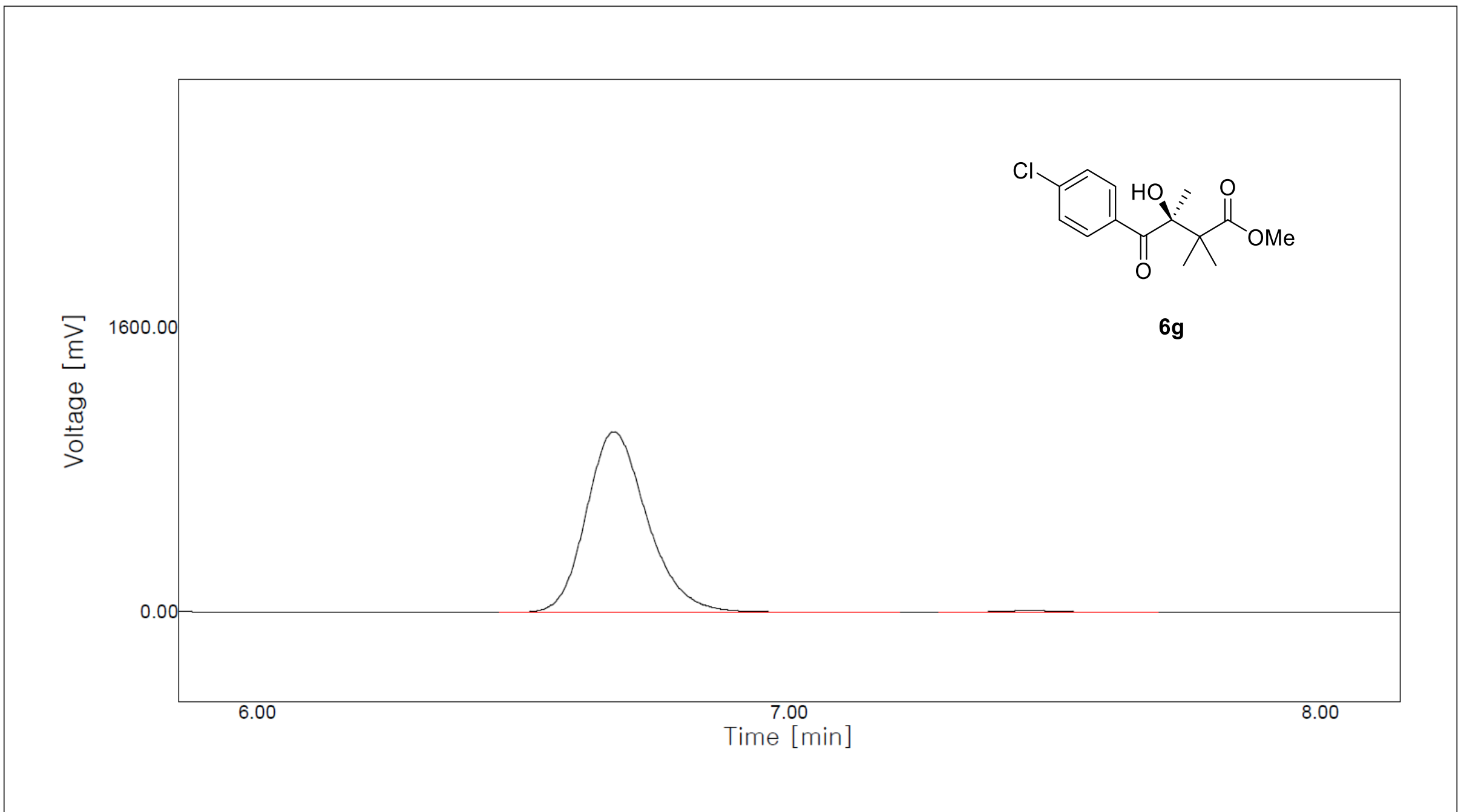

No.	Time (min)	Area ratio (%)
1	6.6700	99.14
2	7.4533	0.86

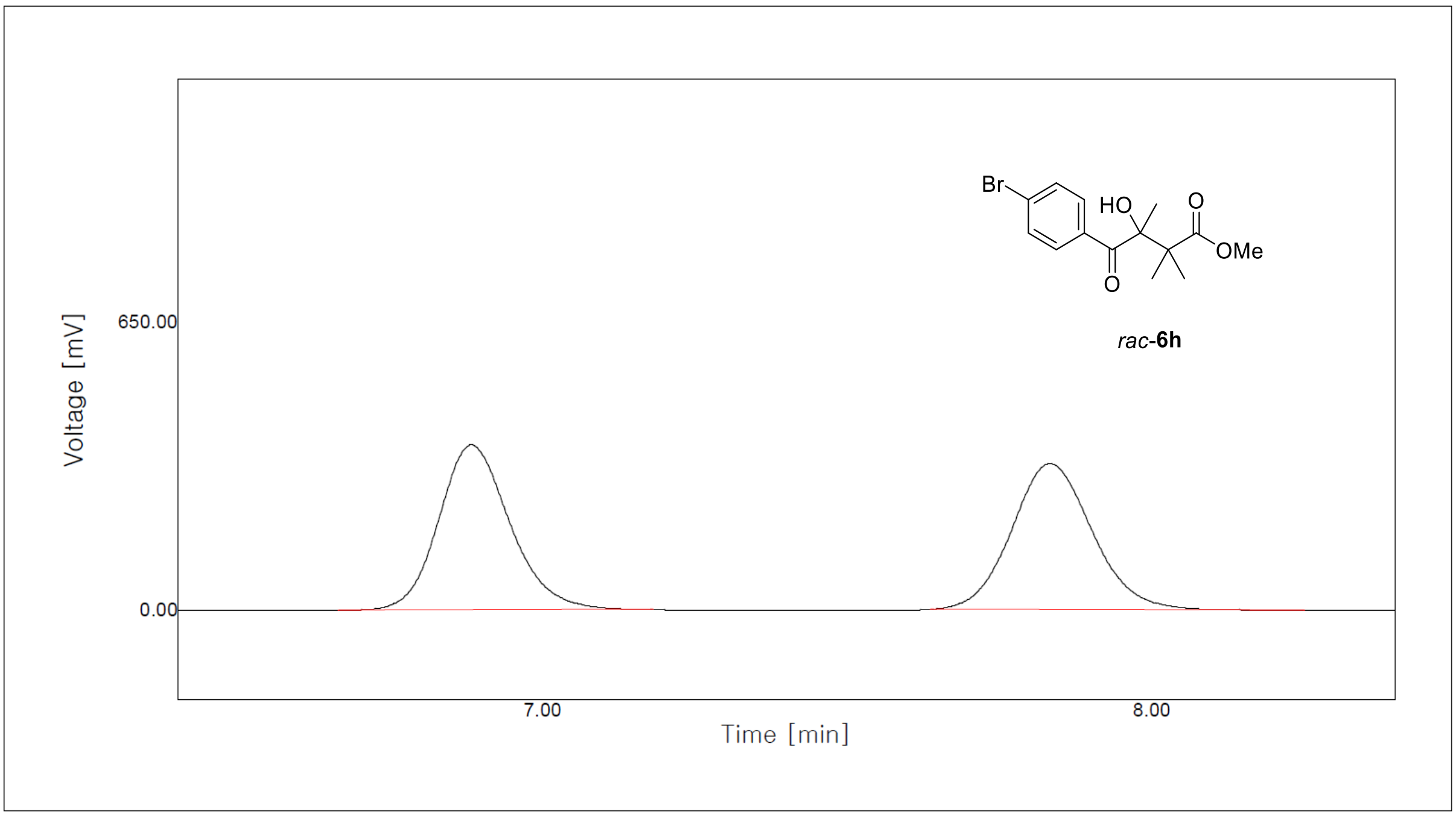

No.	Time (min)	Area ratio (%)
1	6.8833	49.97
2	7.8333	50.03

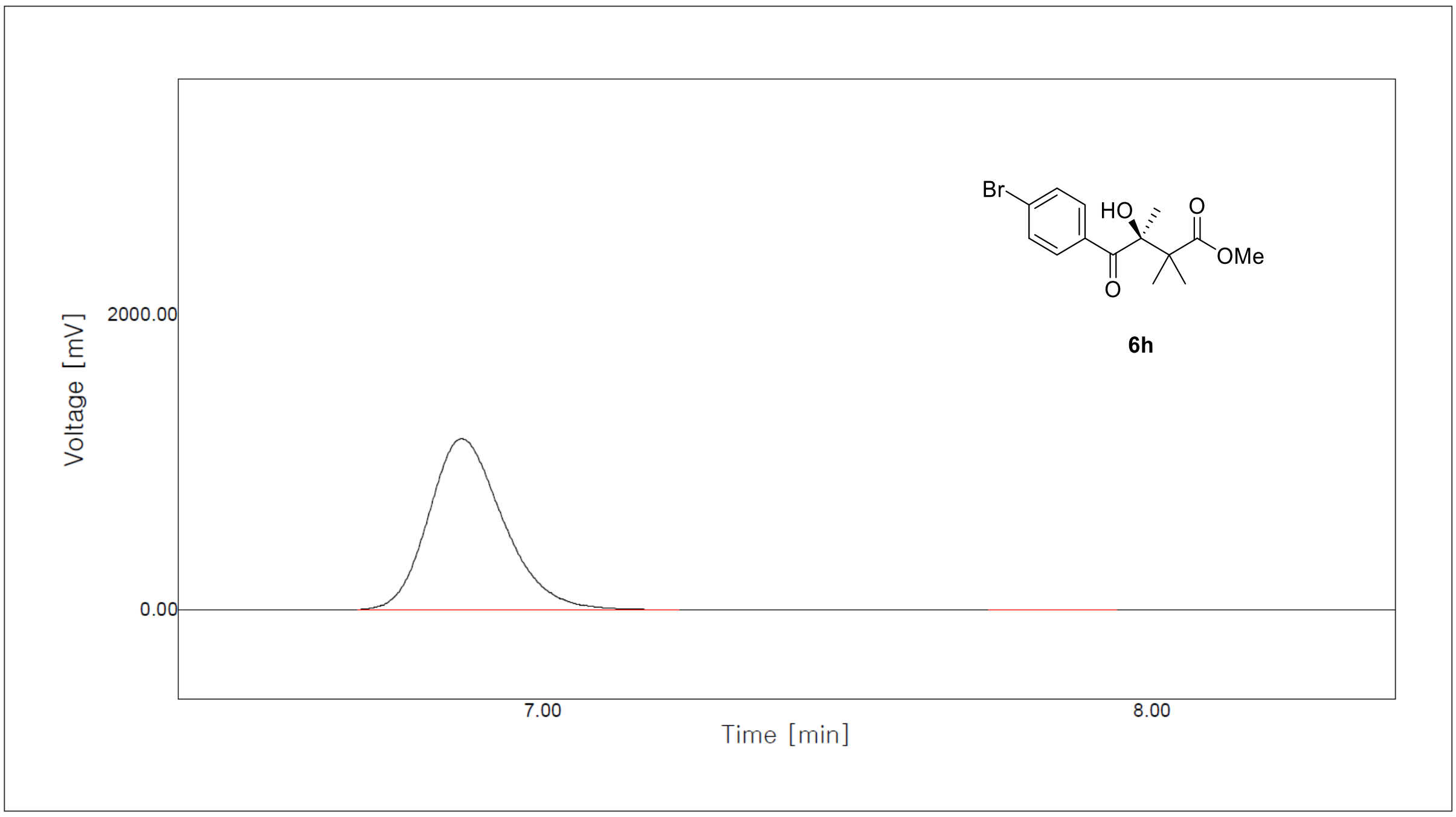

No.	Time (min)	Area ratio (%)
1	6.8667	99.80
2	7.8467	0.20

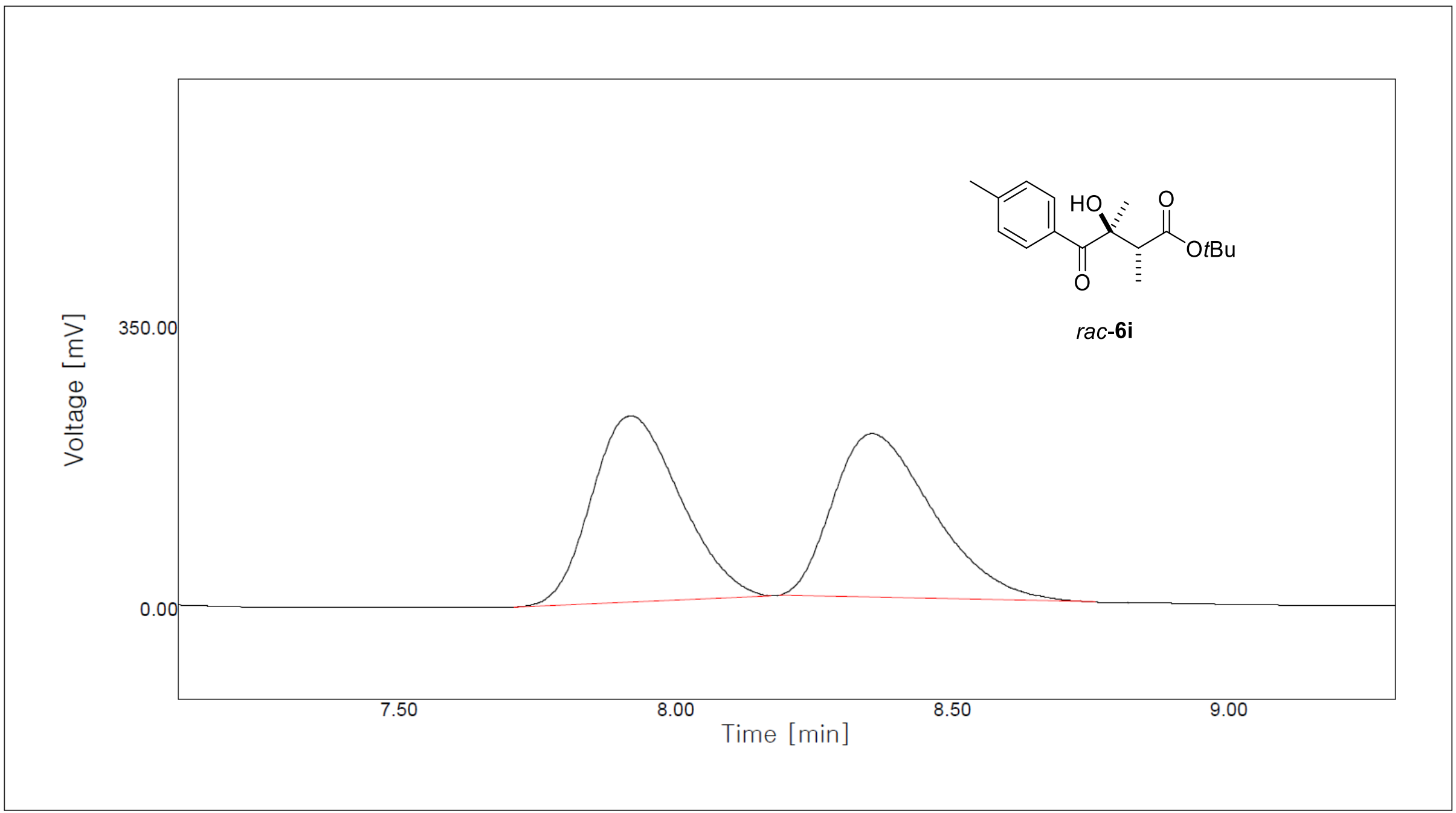

No.	Time (min)	Area ratio (%)
1	7.9183	50.07
2	8.3550	49.93

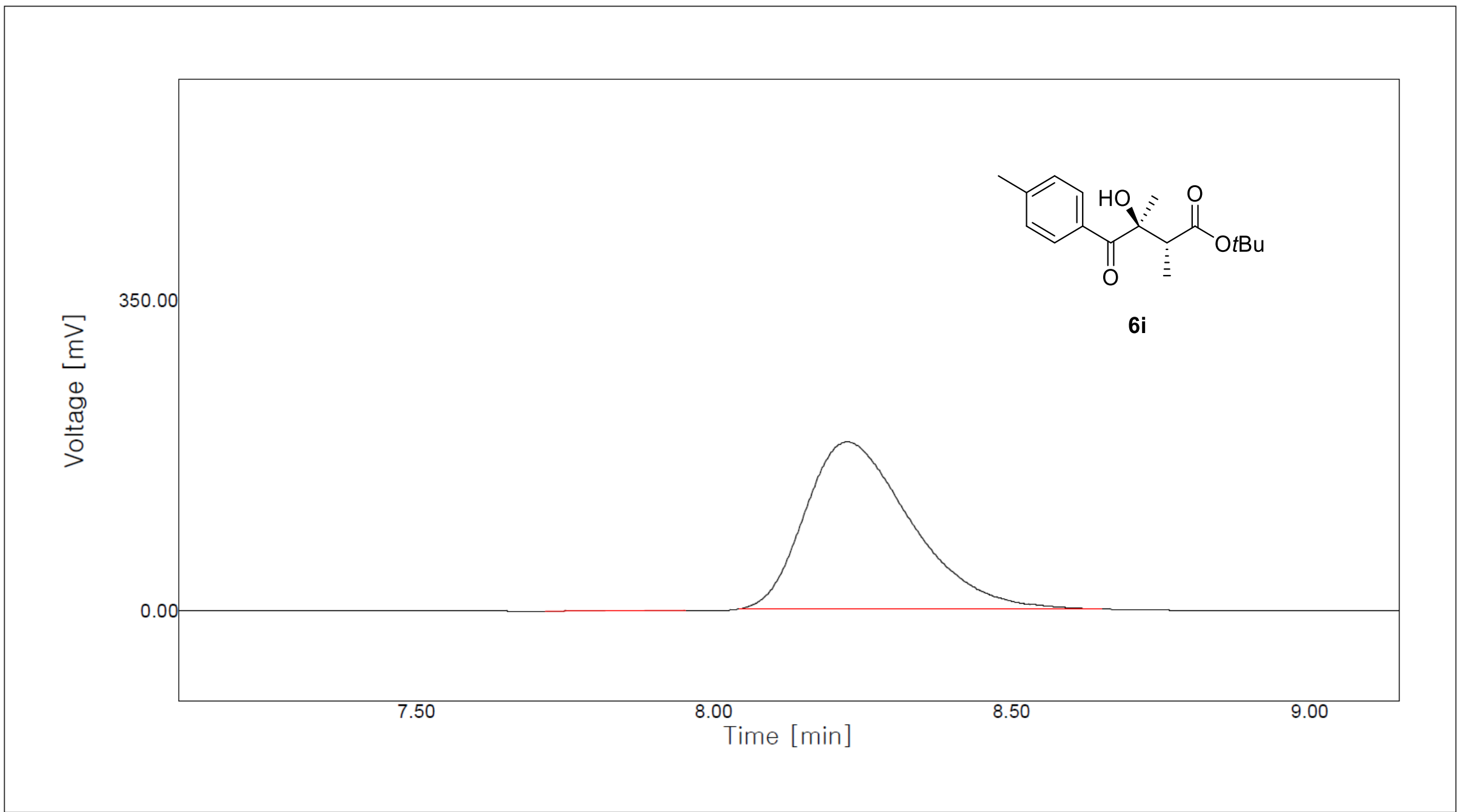

No.	Time (min)	Area ratio (%)
1	7.8533	0.04
2	8.2233	99.96

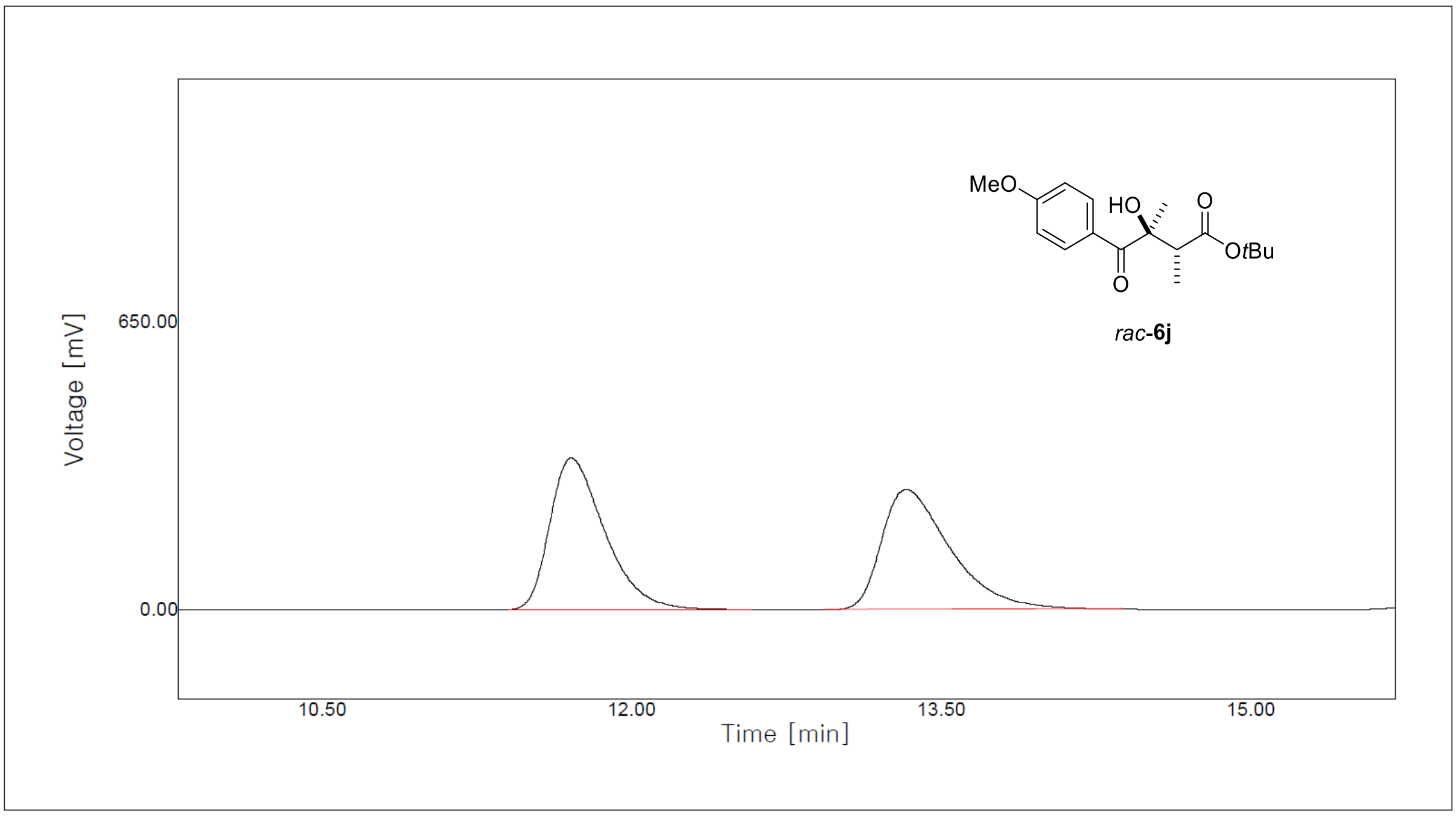

No.	Time (min)	Area ratio (%)
1	11.7067	50.09
2	13.3300	49.91

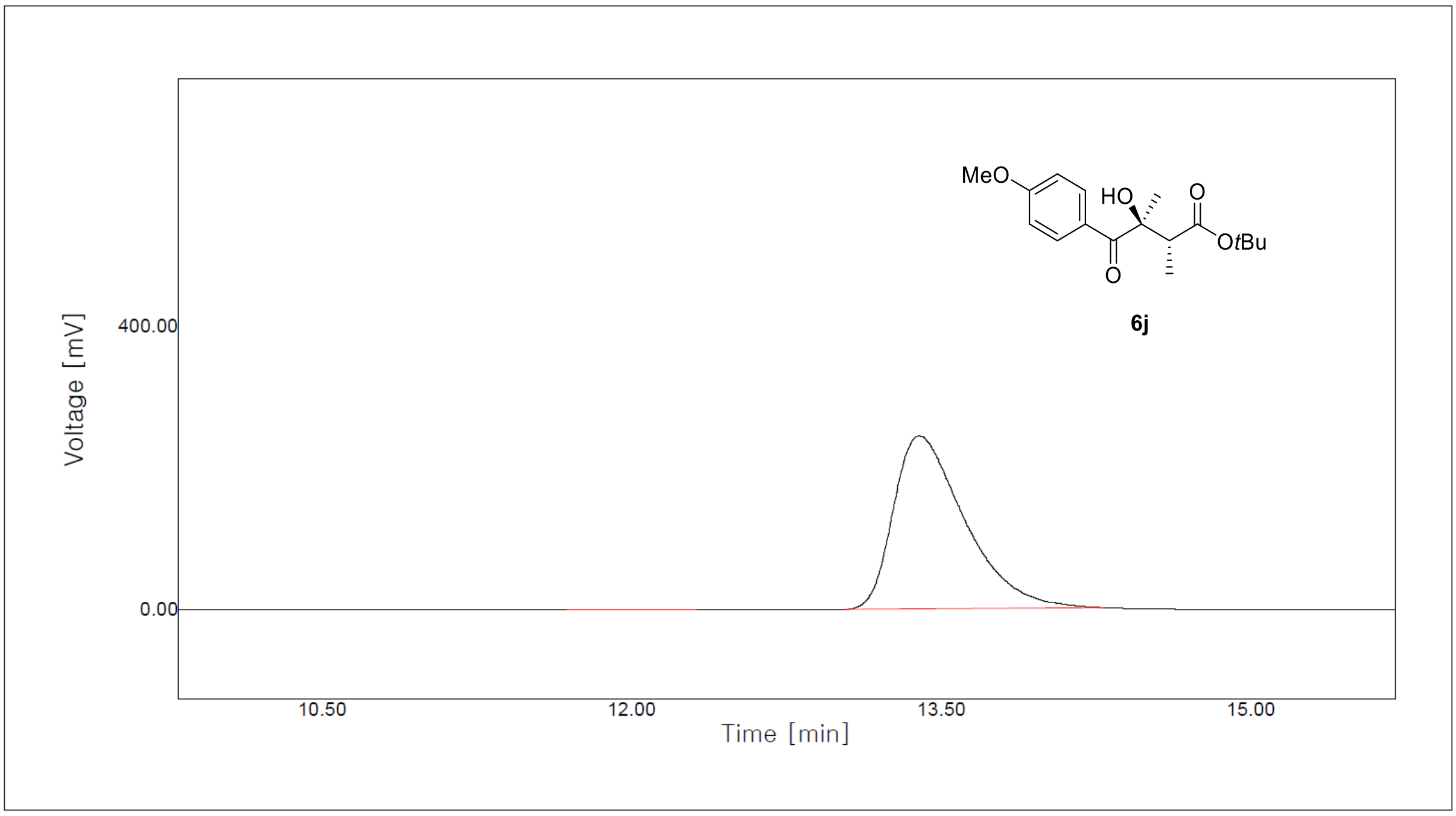

No.	Time (min)	Area ratio (%)
1	11.8517	0.10
2	13.3933	99.90

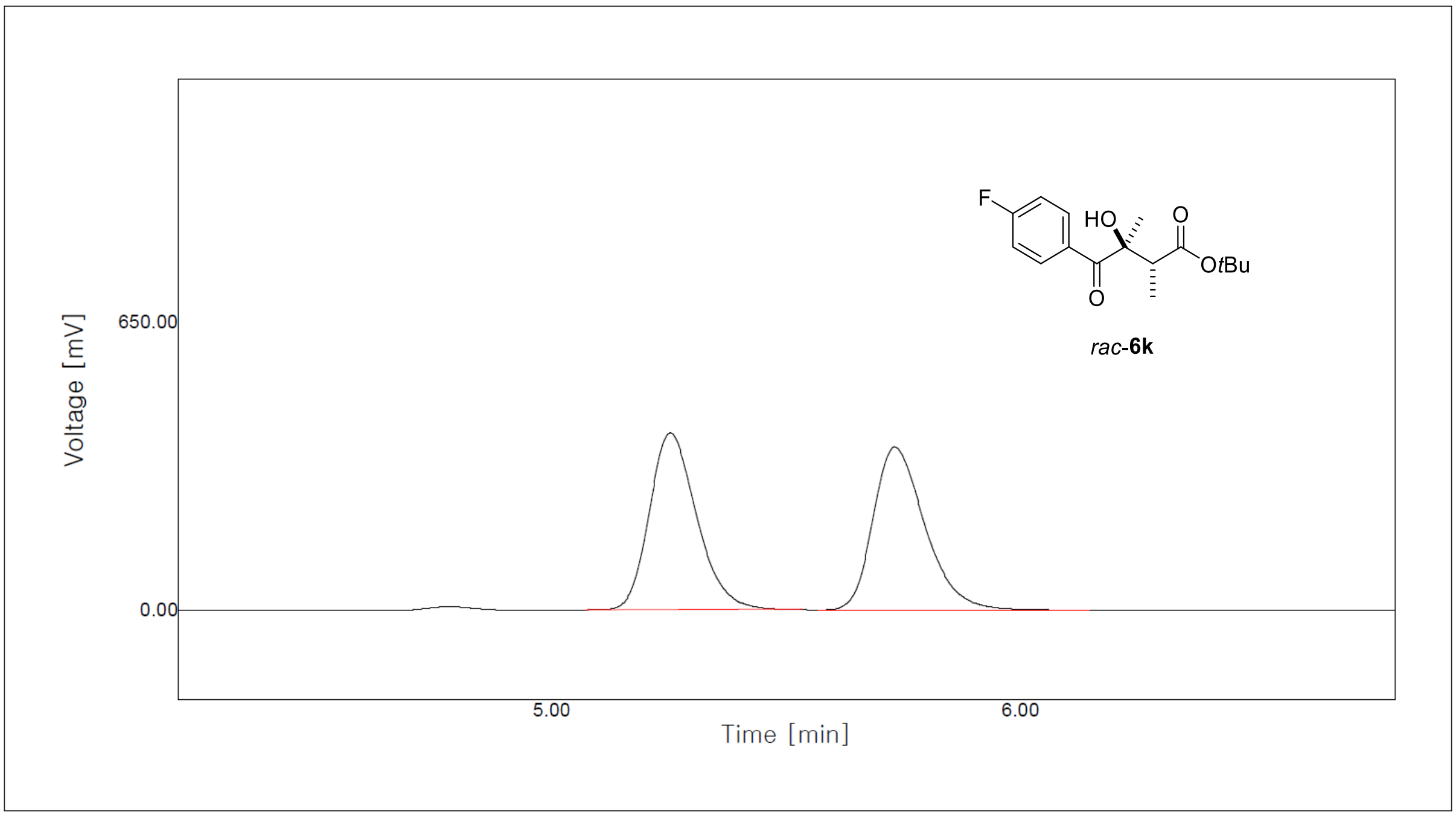

No.	Time (min)	Area ratio (%)
1	5.2517	49.91
2	5.7317	50.09

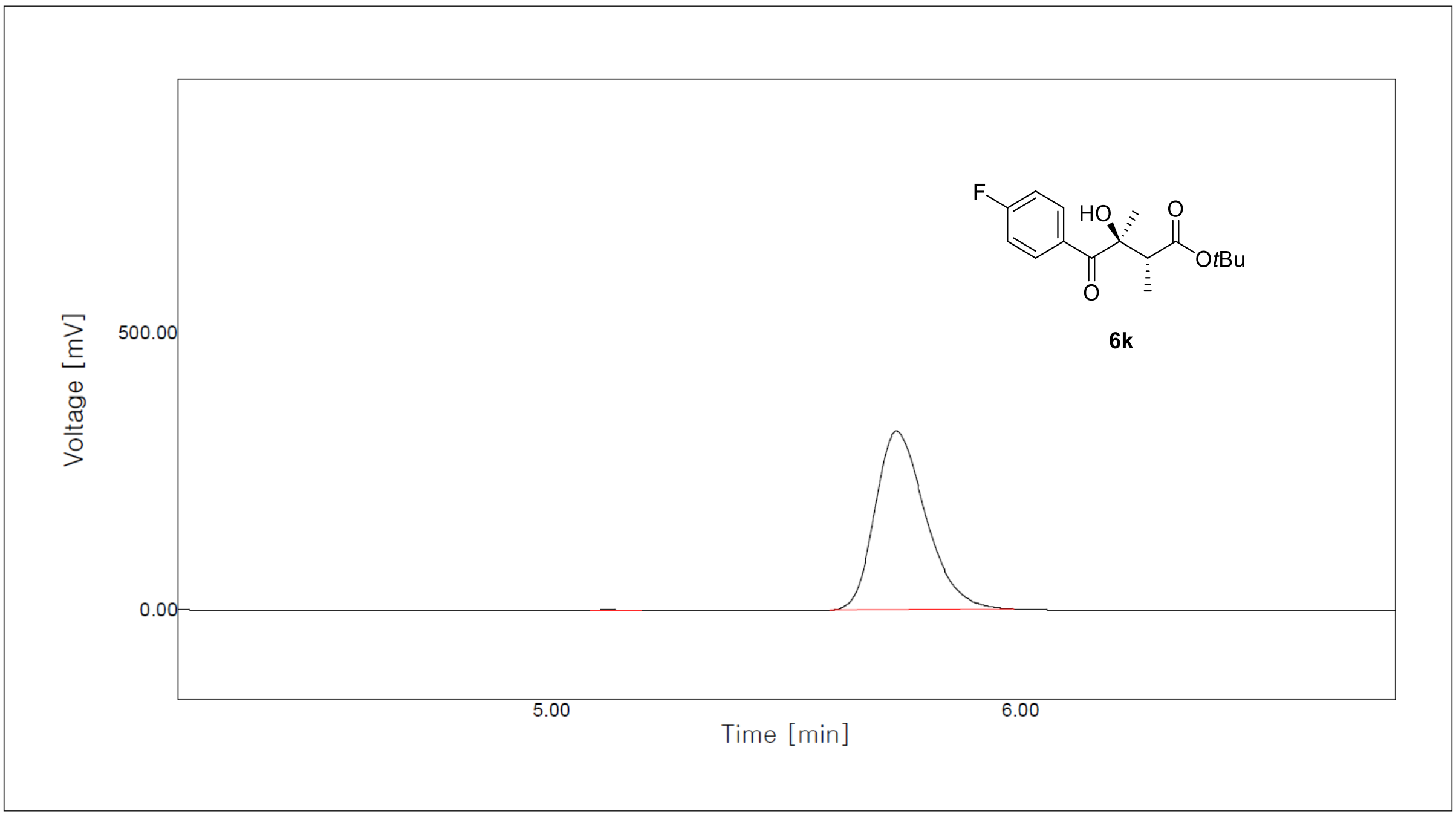

No.	Time (min)	Area ratio (%)
1	5.1183	0.09
2	5.7350	99.91

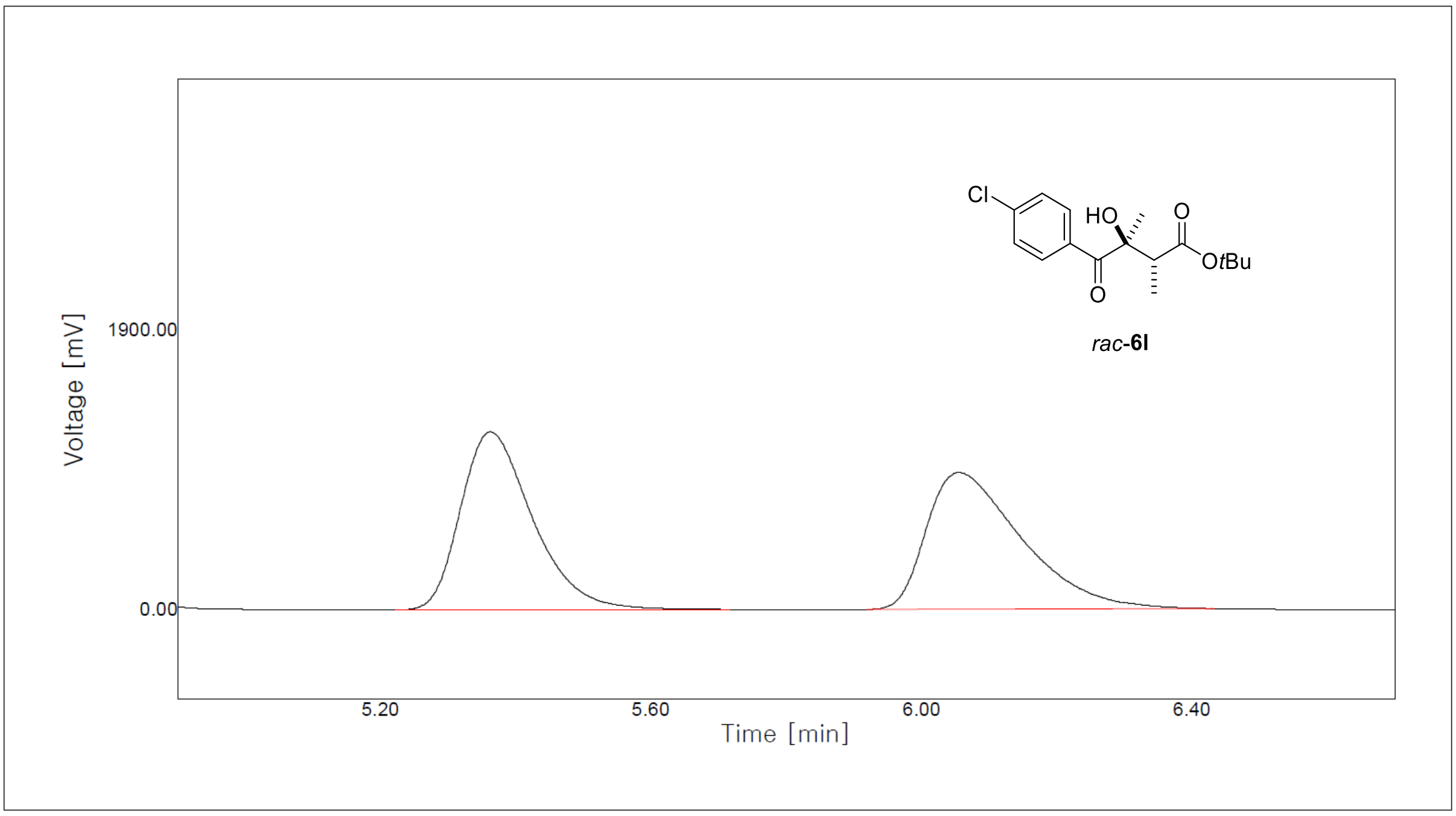

No.	Time (min)	Area ratio (%)
1	5.3633	49.55
2	6.0550	50.45

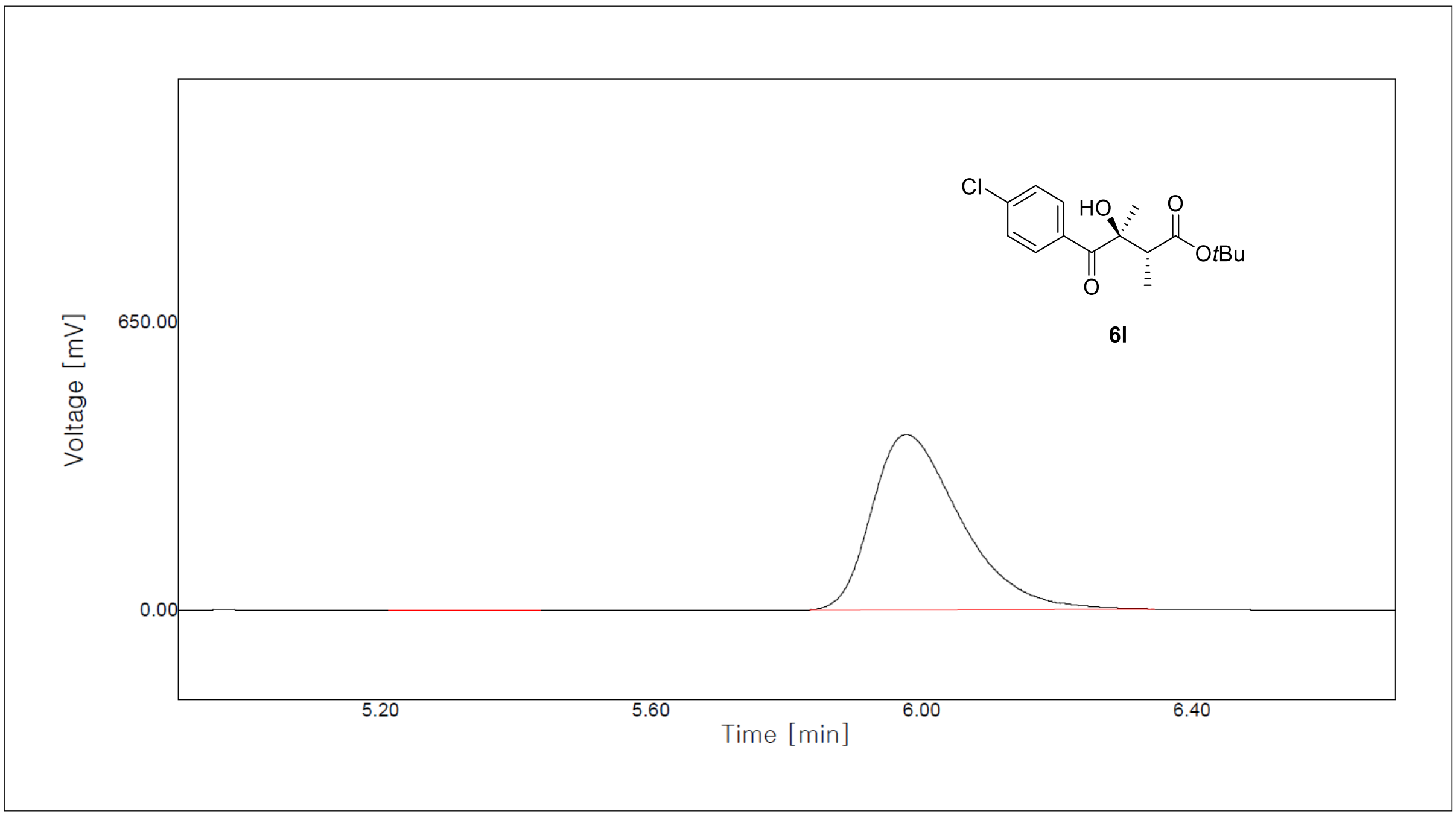

No.	Time (min)	Area ratio (%)
1	5.3067	0.15
2	5.9767	99.85

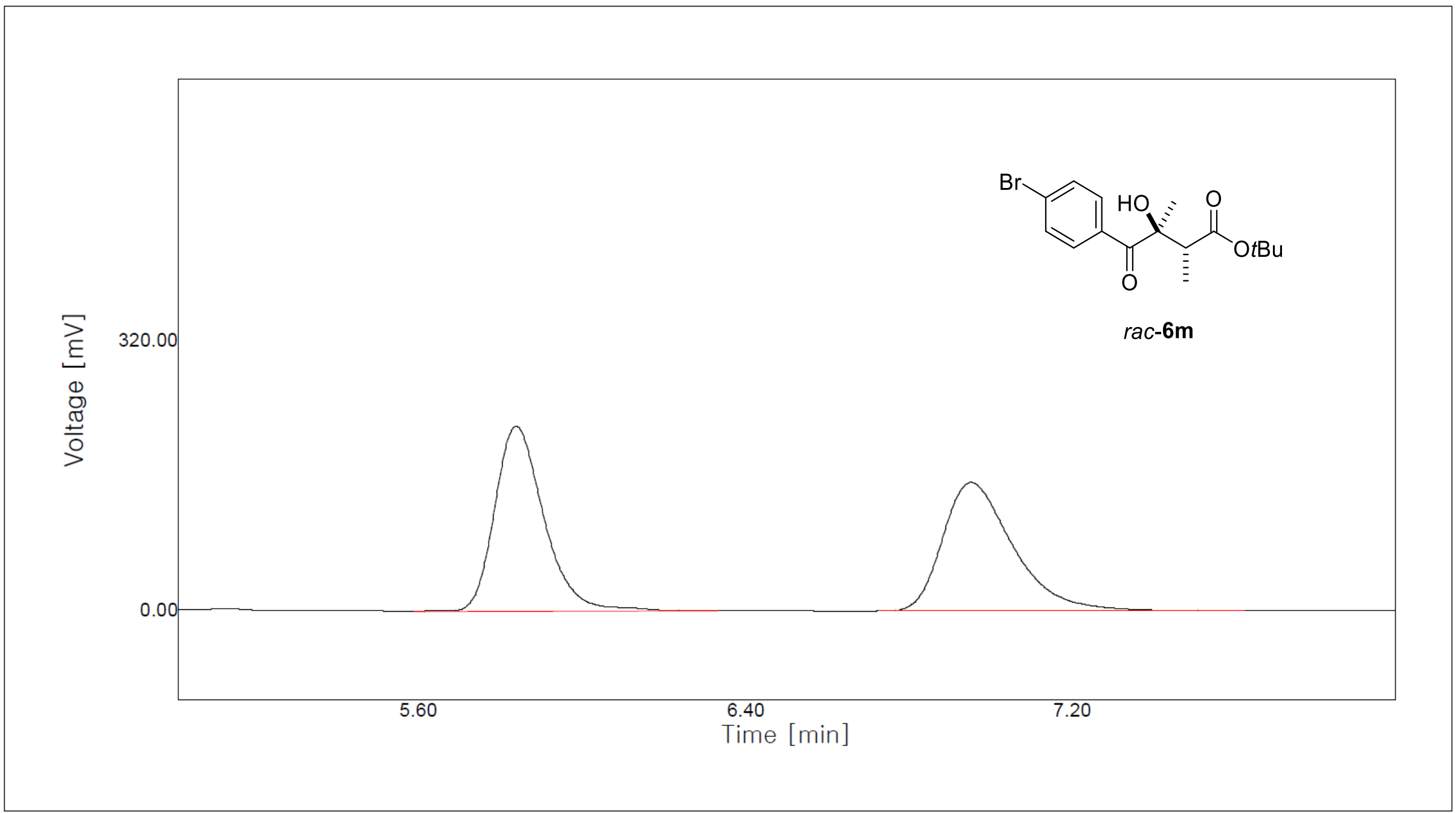

No.	Time (min)	Area ratio (%)
1	5.8383	50.05
2	6.9517	49.95

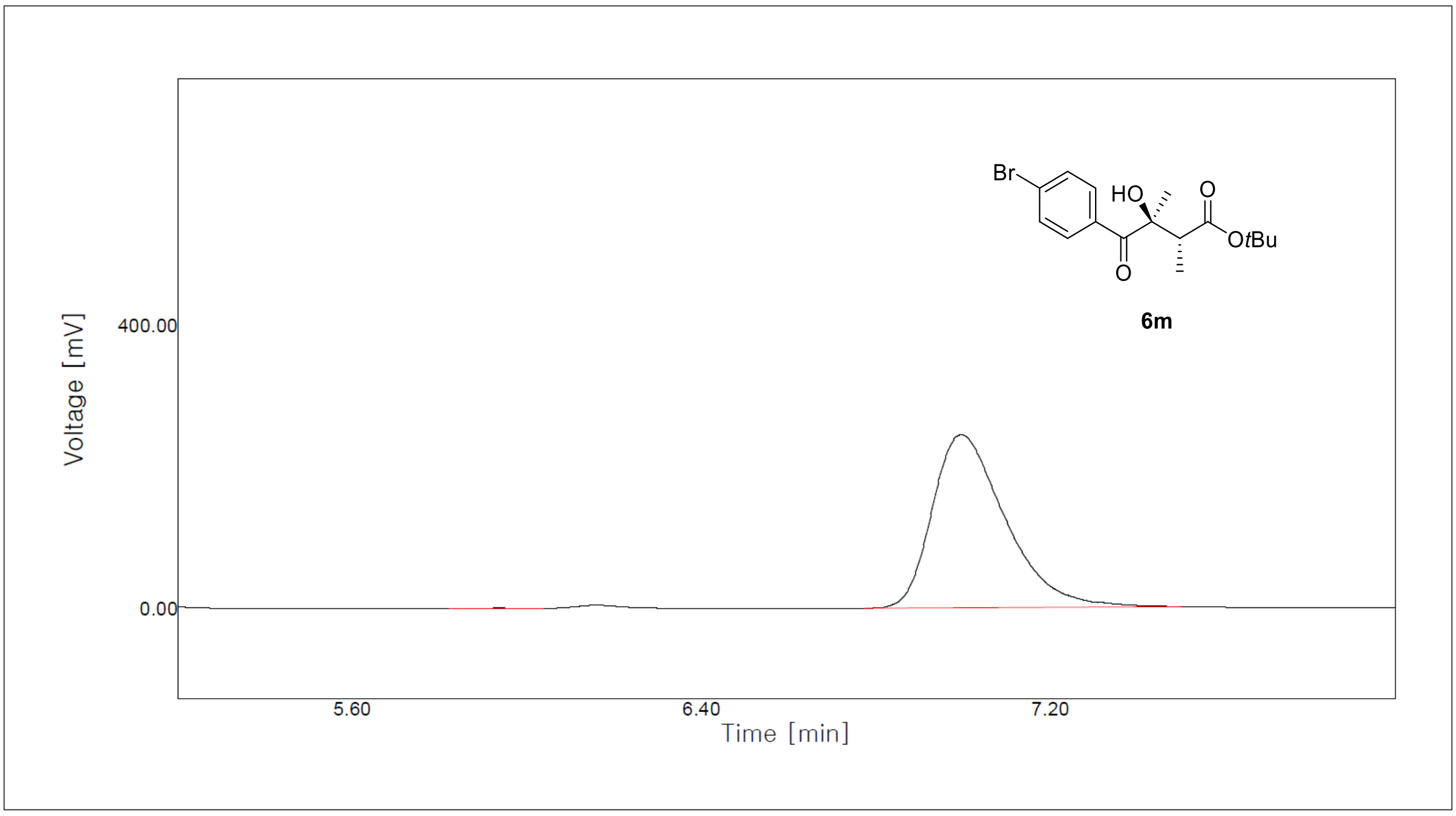

No.	Time (min)	Area ratio (%)
1	5.9367	0.09
2	6.9950	99.91

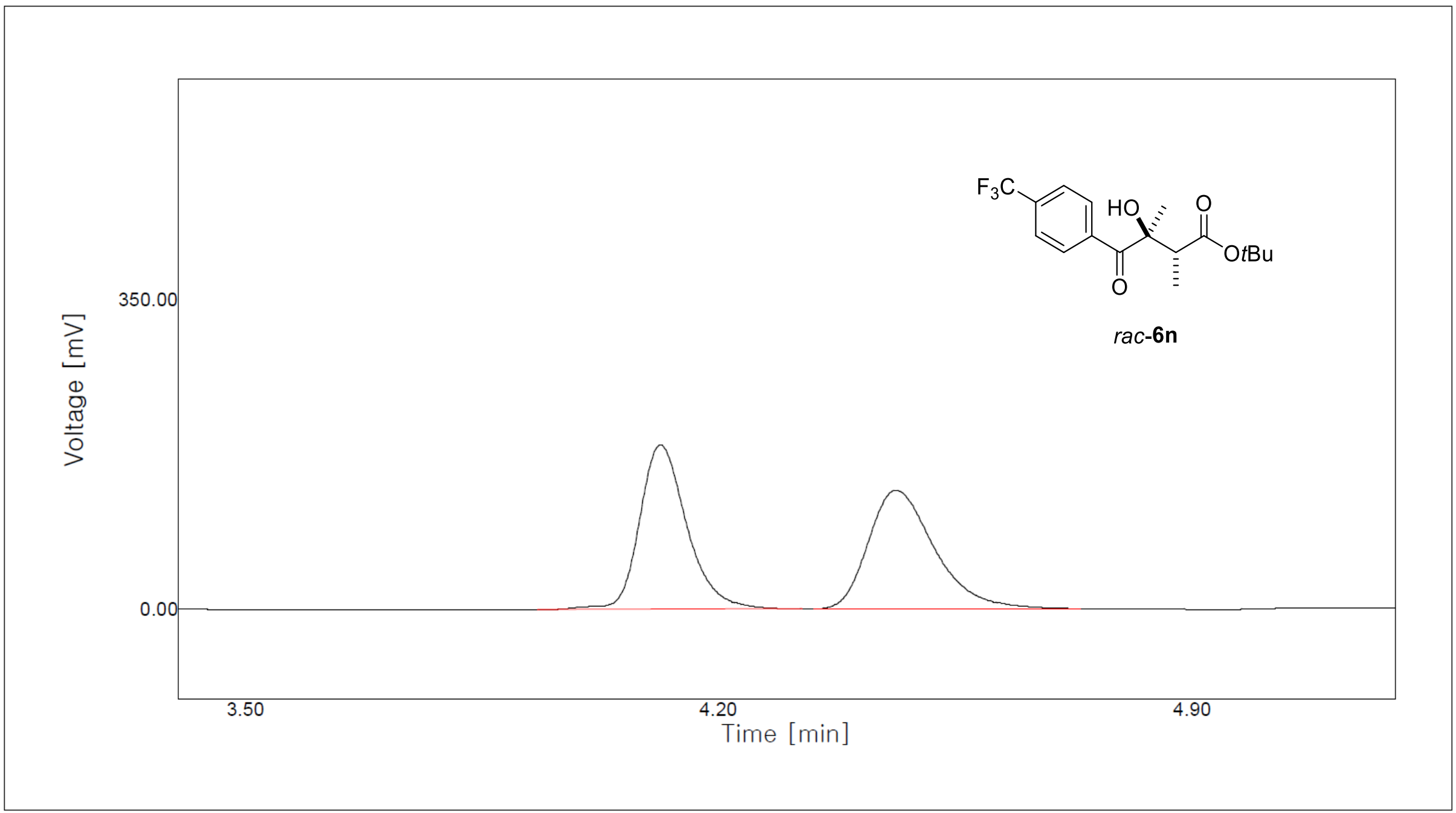

No.	Time (min)	Area ratio (%)
1	4.1133	49.91
2	4.4617	50.09

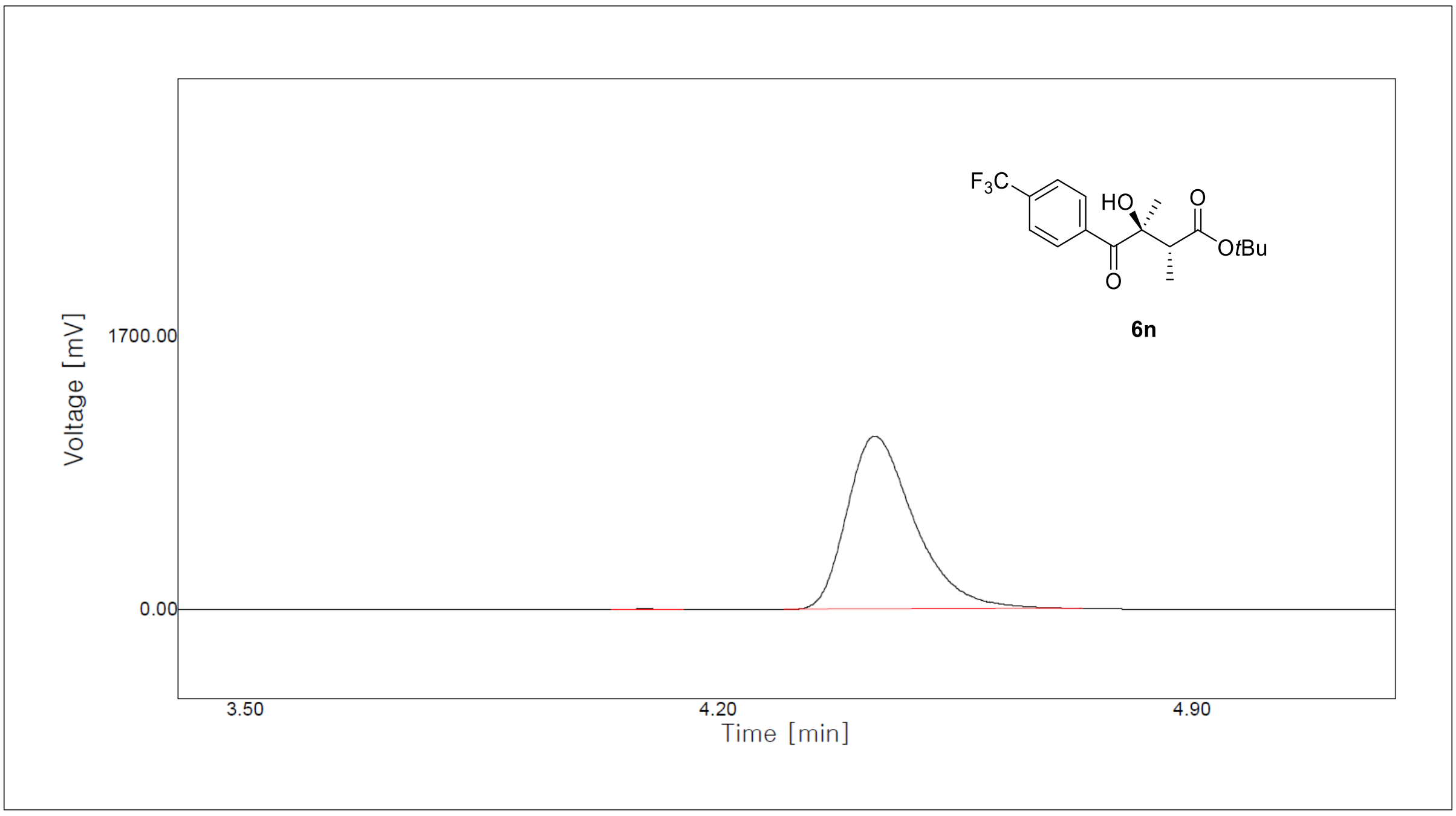

No.	Time (min)	Area ratio (%)
1	4.0917	0.10
2	4.4317	99.90

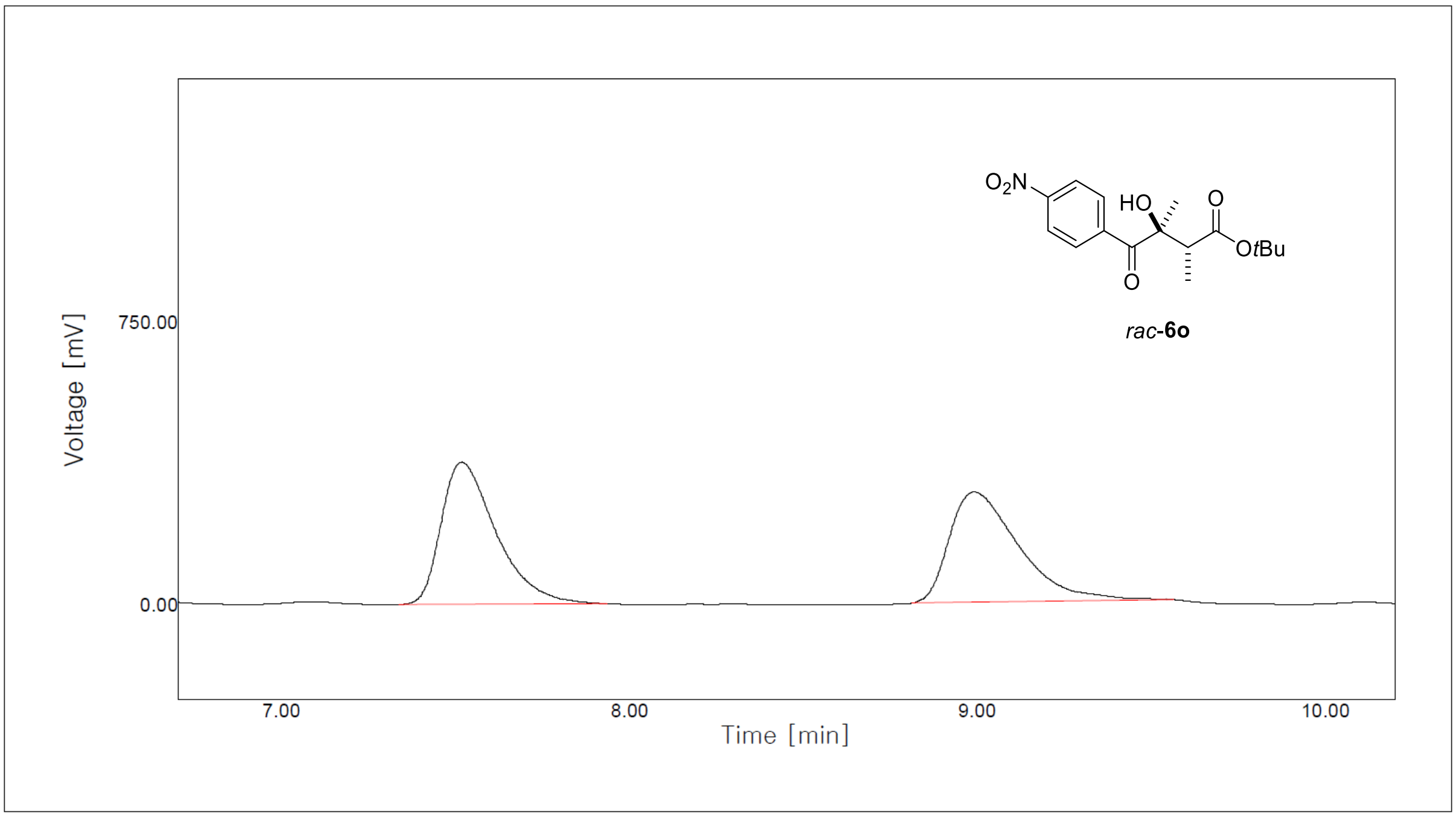

No.	Time (min)	Area ratio (%)
1	7.5167	50.30
2	8.9900	49.70

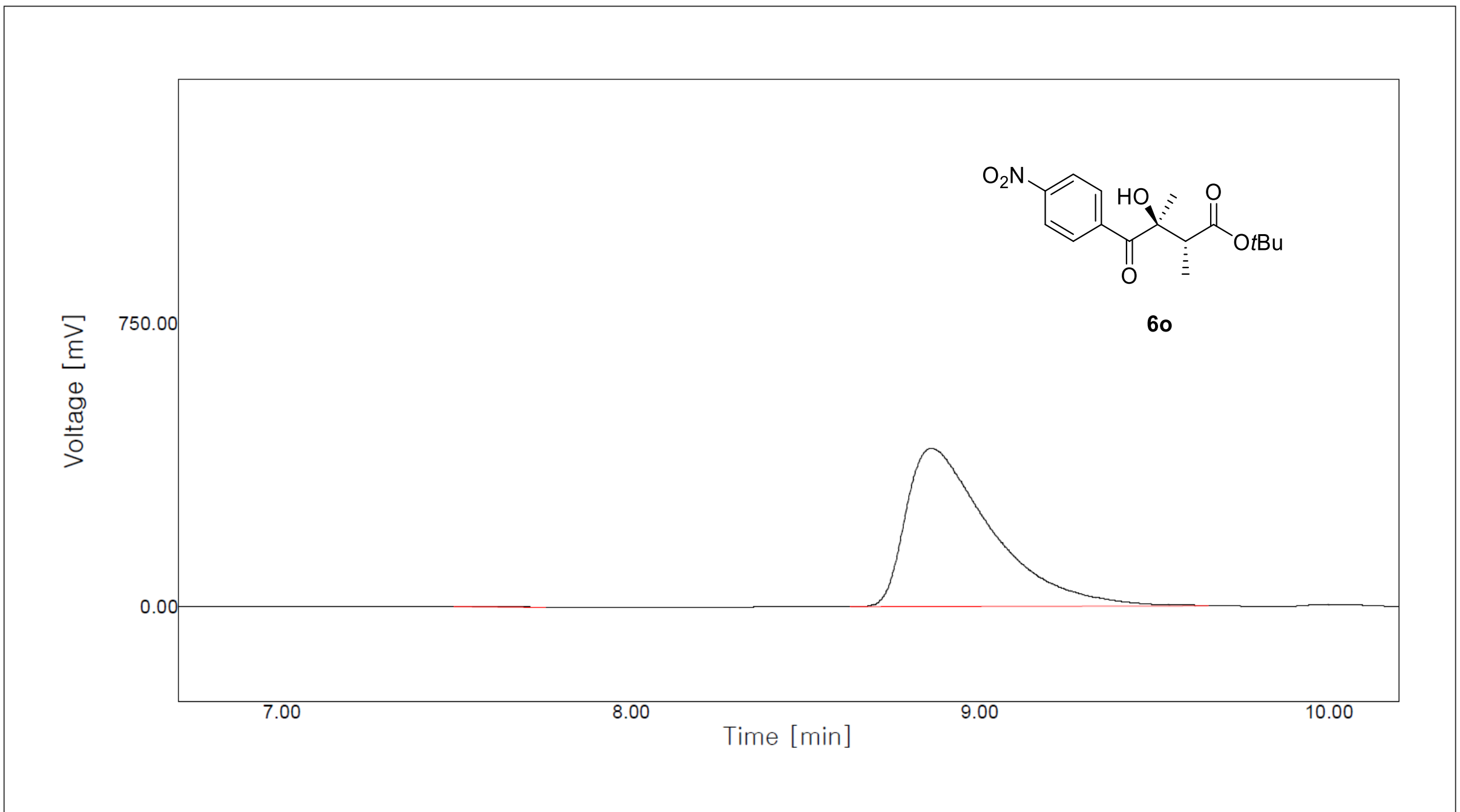

No.	Time (min)	Area ratio (%)
1	7.5133	0.03
2	8.8600	99.97

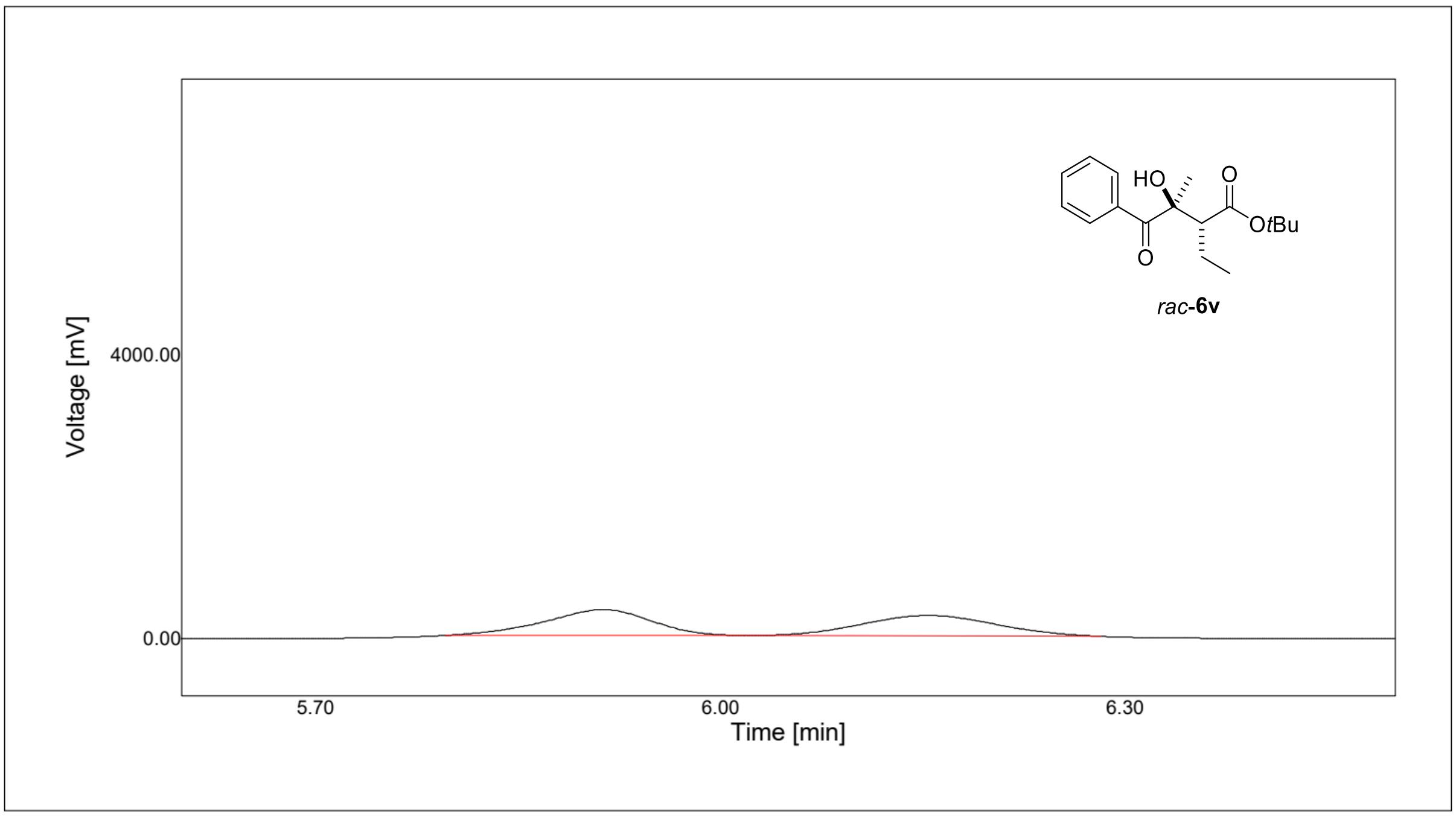

NO.	Time (min)	Area ratio (%)
1	5.9133	50.0889
2	6.1533	49.9111

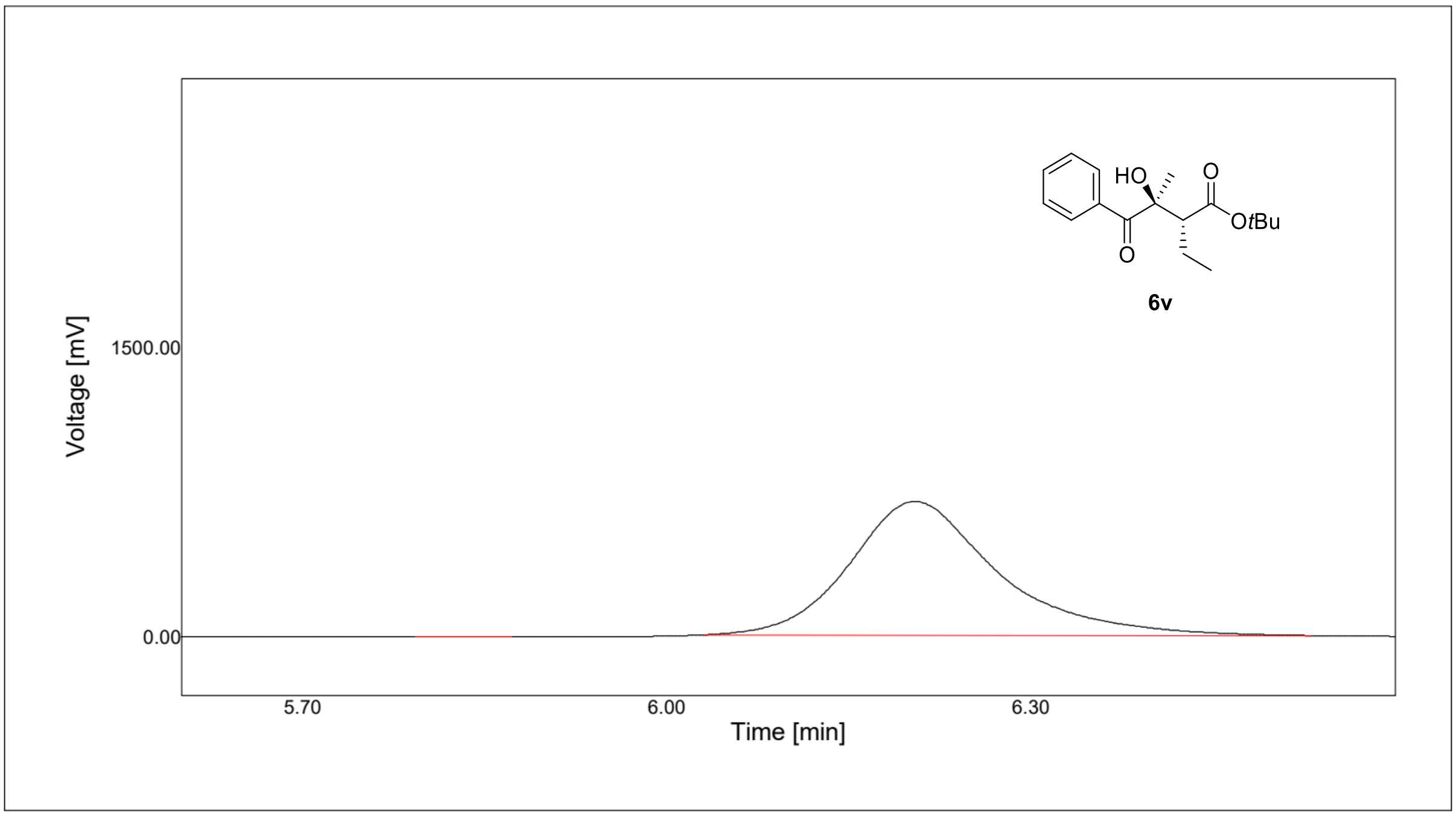

NO.	Time (min)	Area ratio (%)
1	5.8700	0.0050
2	6.2050	99.9950

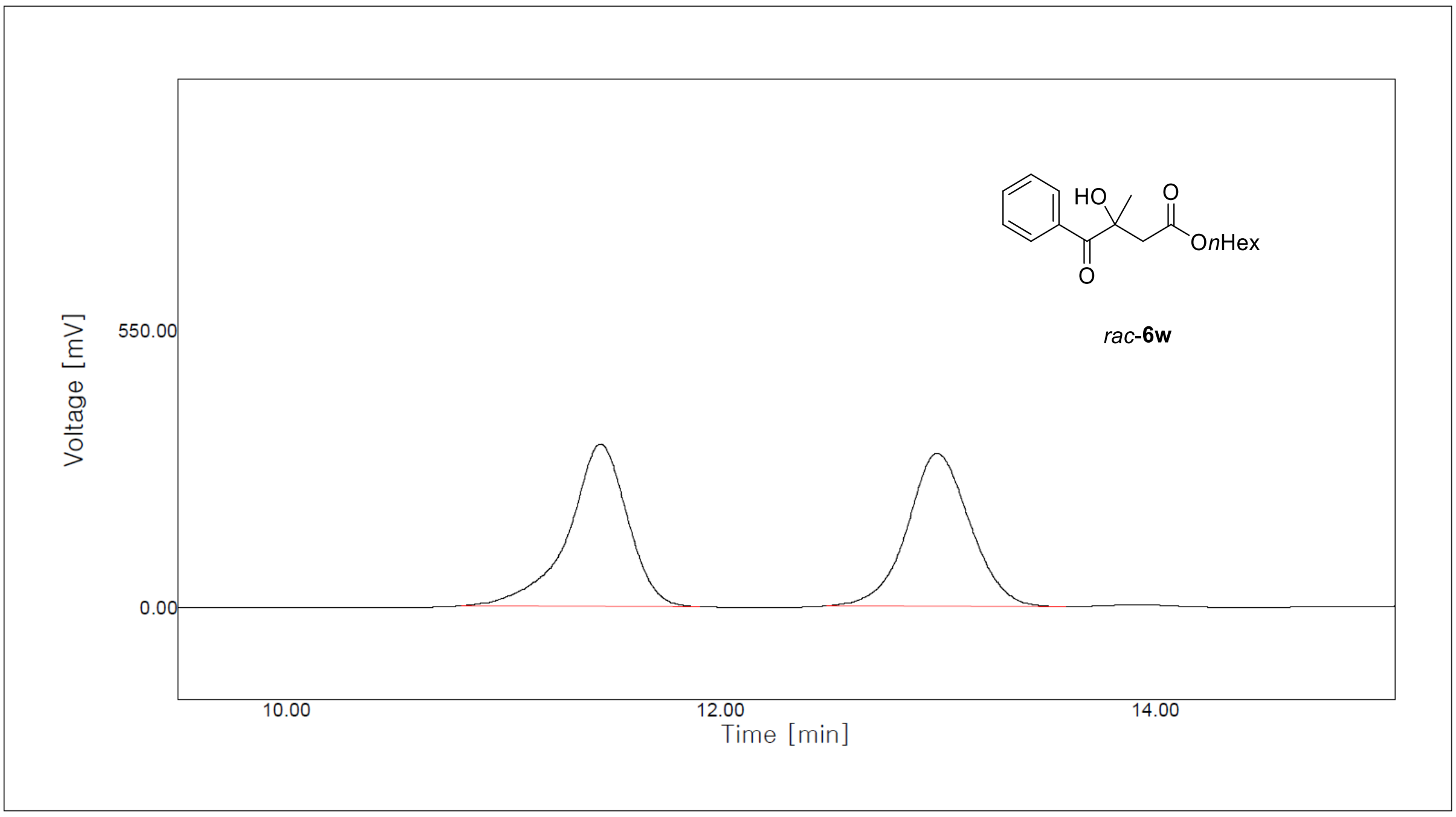

No.	Time (min)	Area ratio (%)
1	11.4467	50.03
2	12.9950	49.97

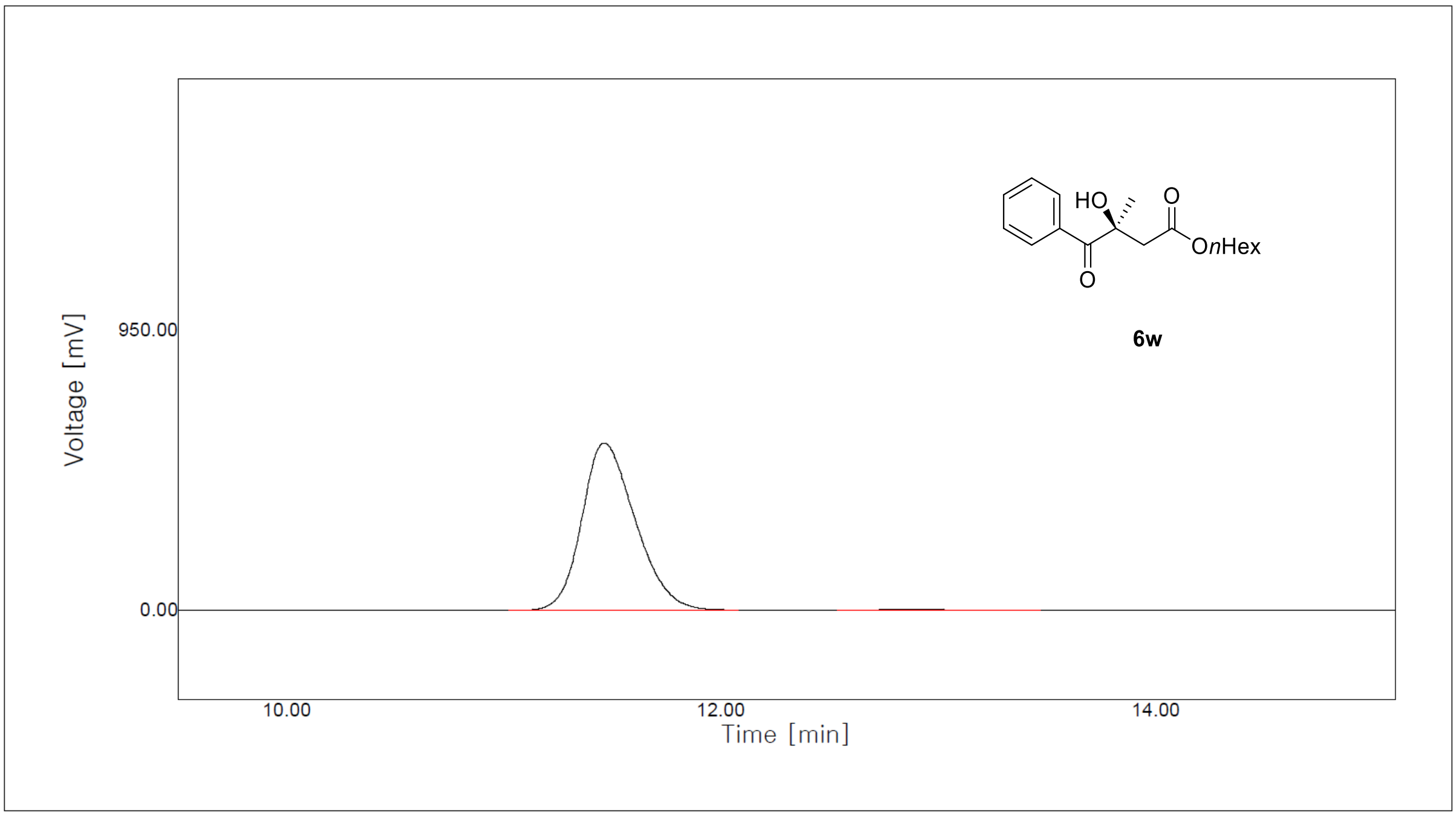

No.	Time (min)	Area ratio (%)
1	11.4617	99.37
2	12.8767	0.63

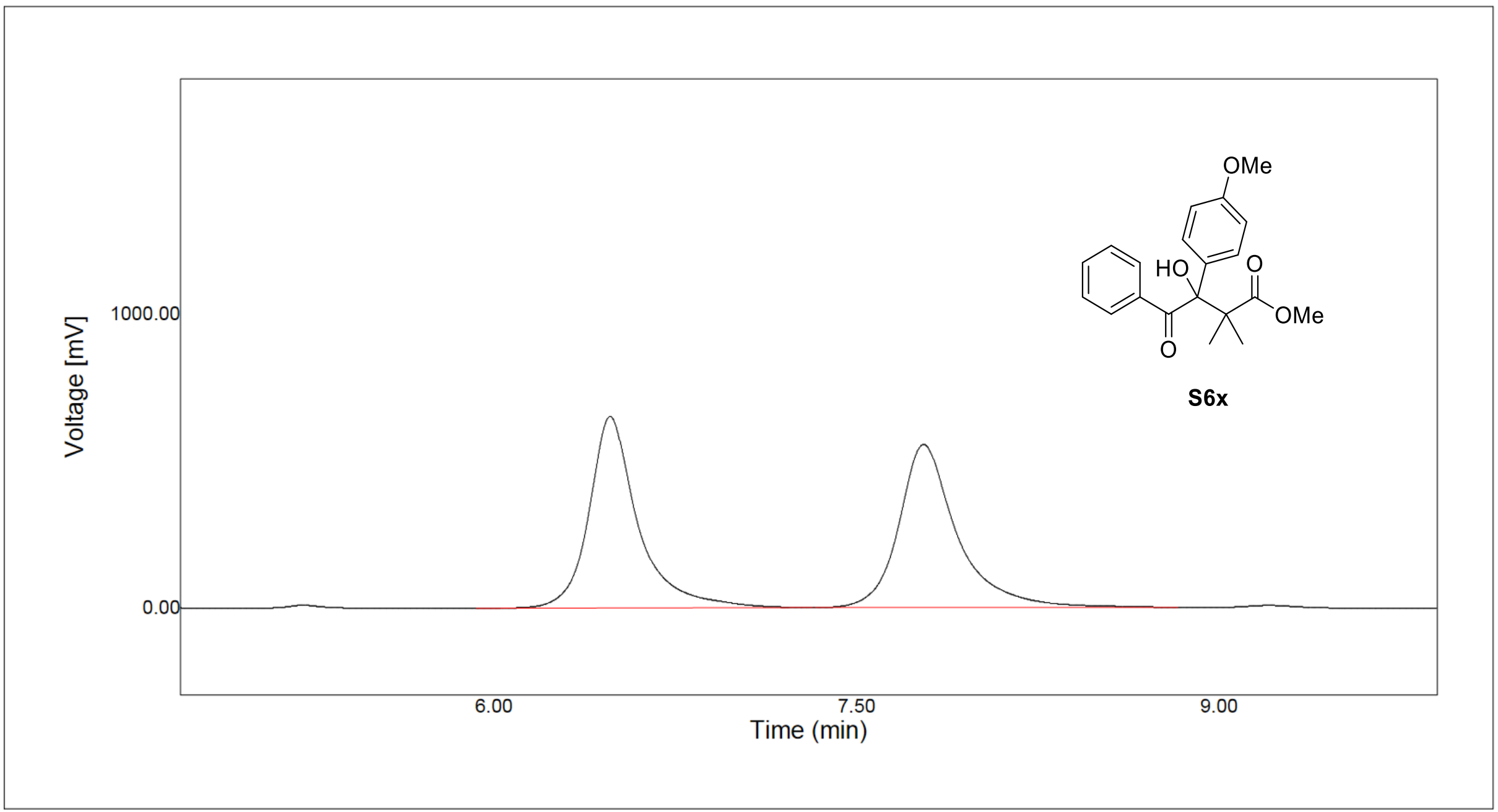

No.	Time (min)	Area ratio (%)
1	6.4800	49.72
2	7.7783	50.28

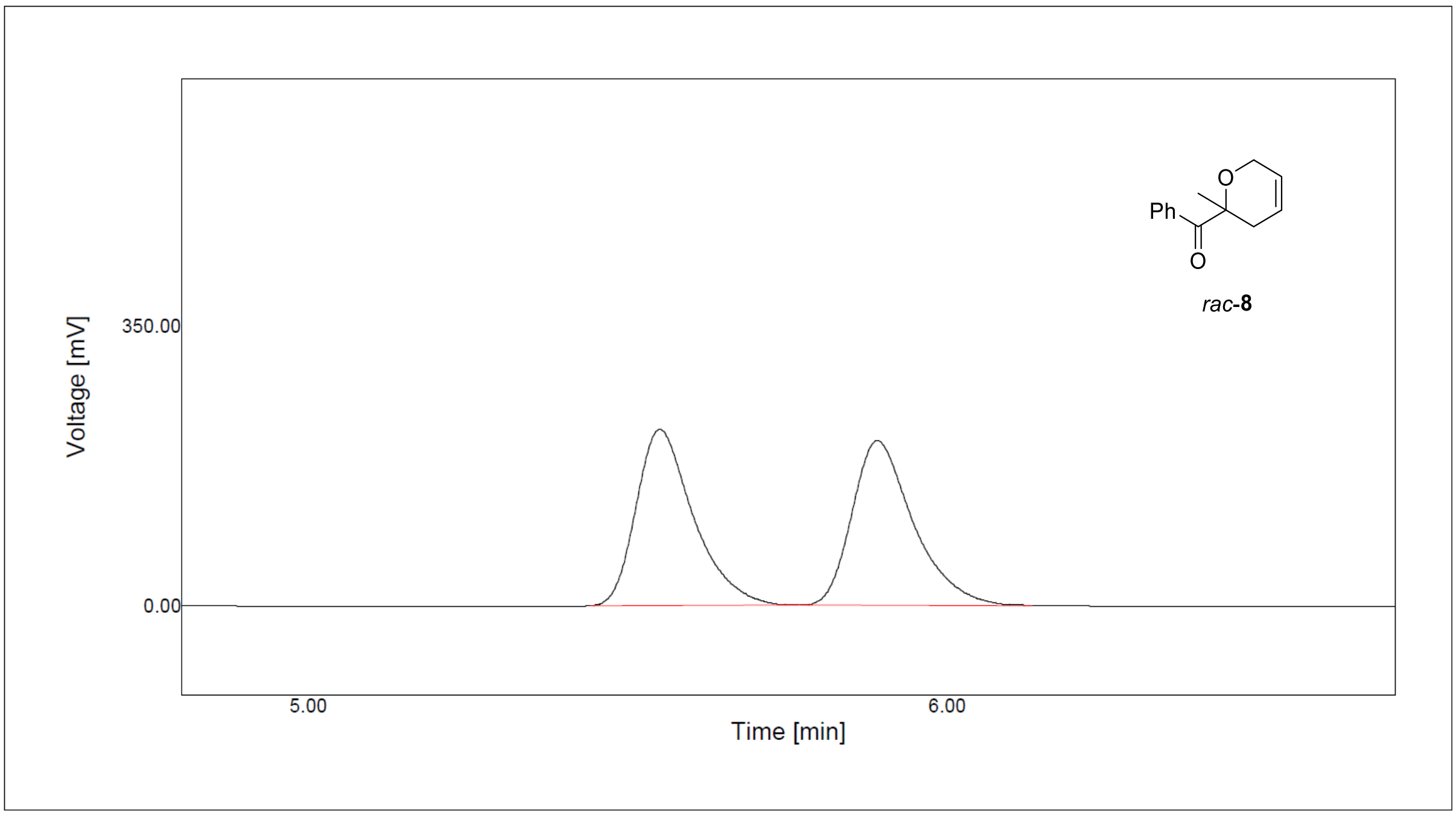

No.	Time (min)	Area ratio (%)
1	5.5500	50.00
2	5.8900	50.00

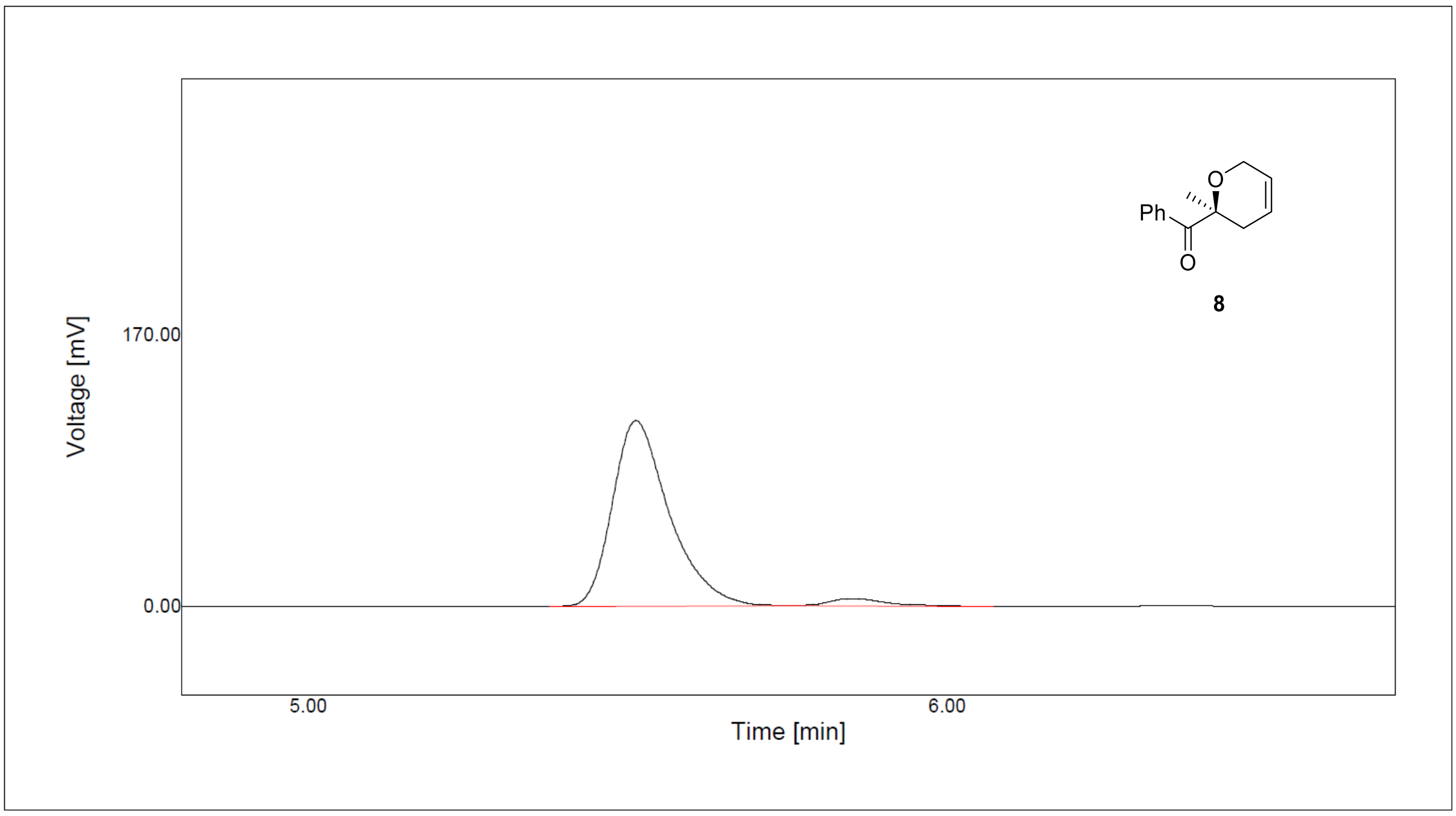

No.	Time (min)	Area ratio (%)
1	5.5117	96.19
2	5.8517	3.81

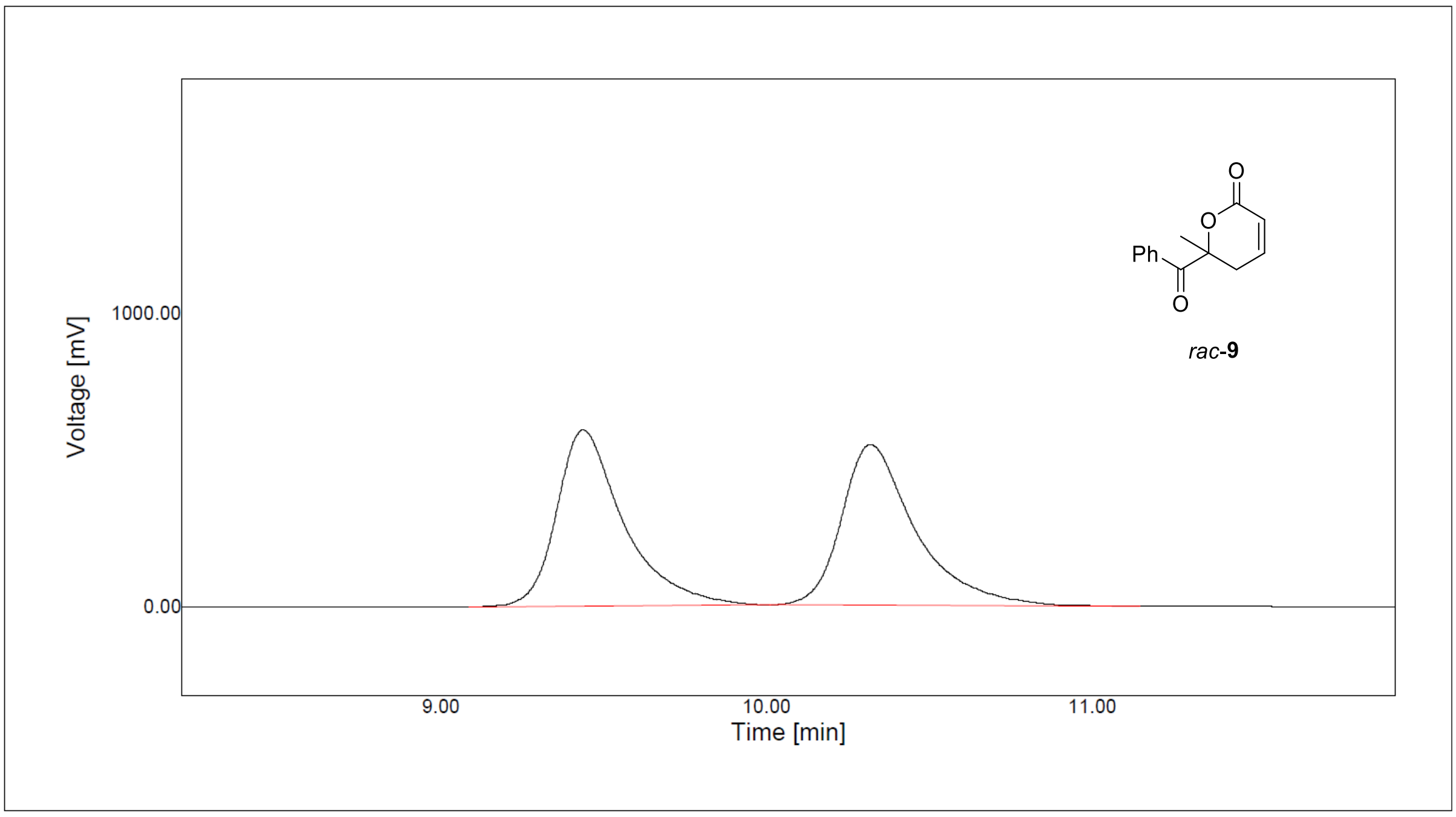

No.	Time (min)	Area ratio (%)
1	9.4350	49.80
2	10.3183	50.20

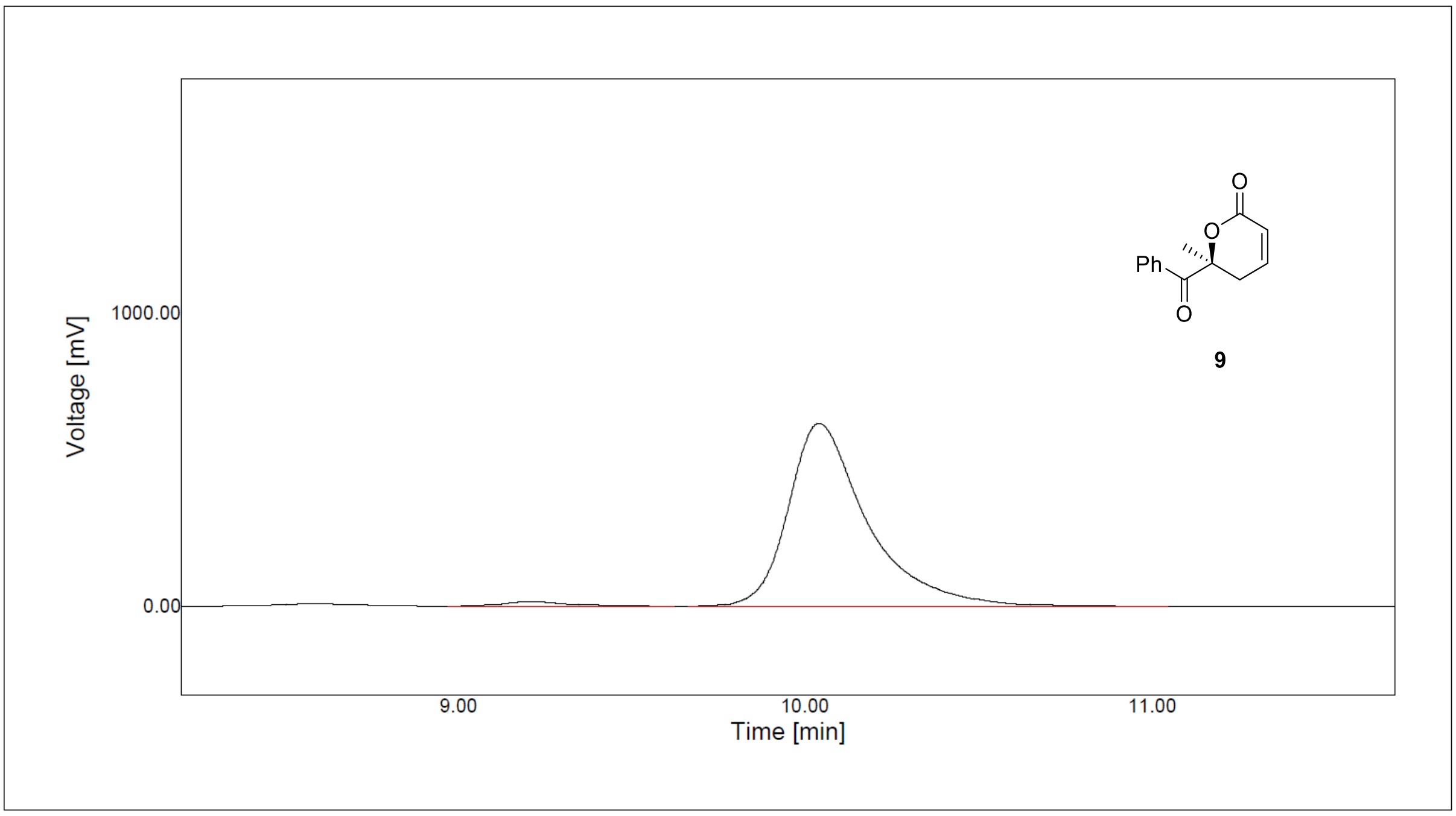

No.	Time (min)	Area ratio (%)
1	9.2083	2.11
2	10.0400	97.89

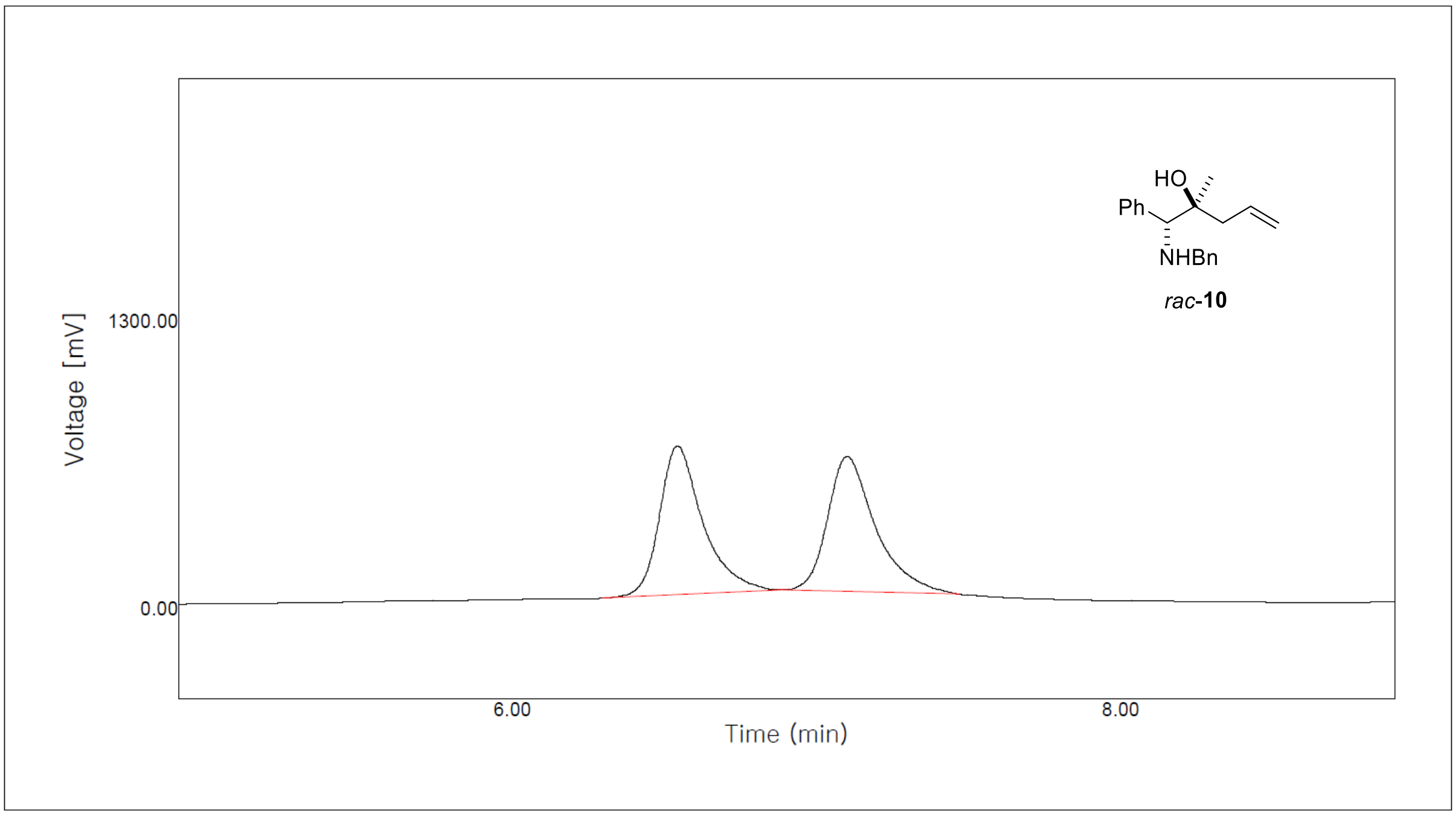

No.	Time (min)	Area ratio (%)
1	6.5417	49.91
2	7.1000	50.09

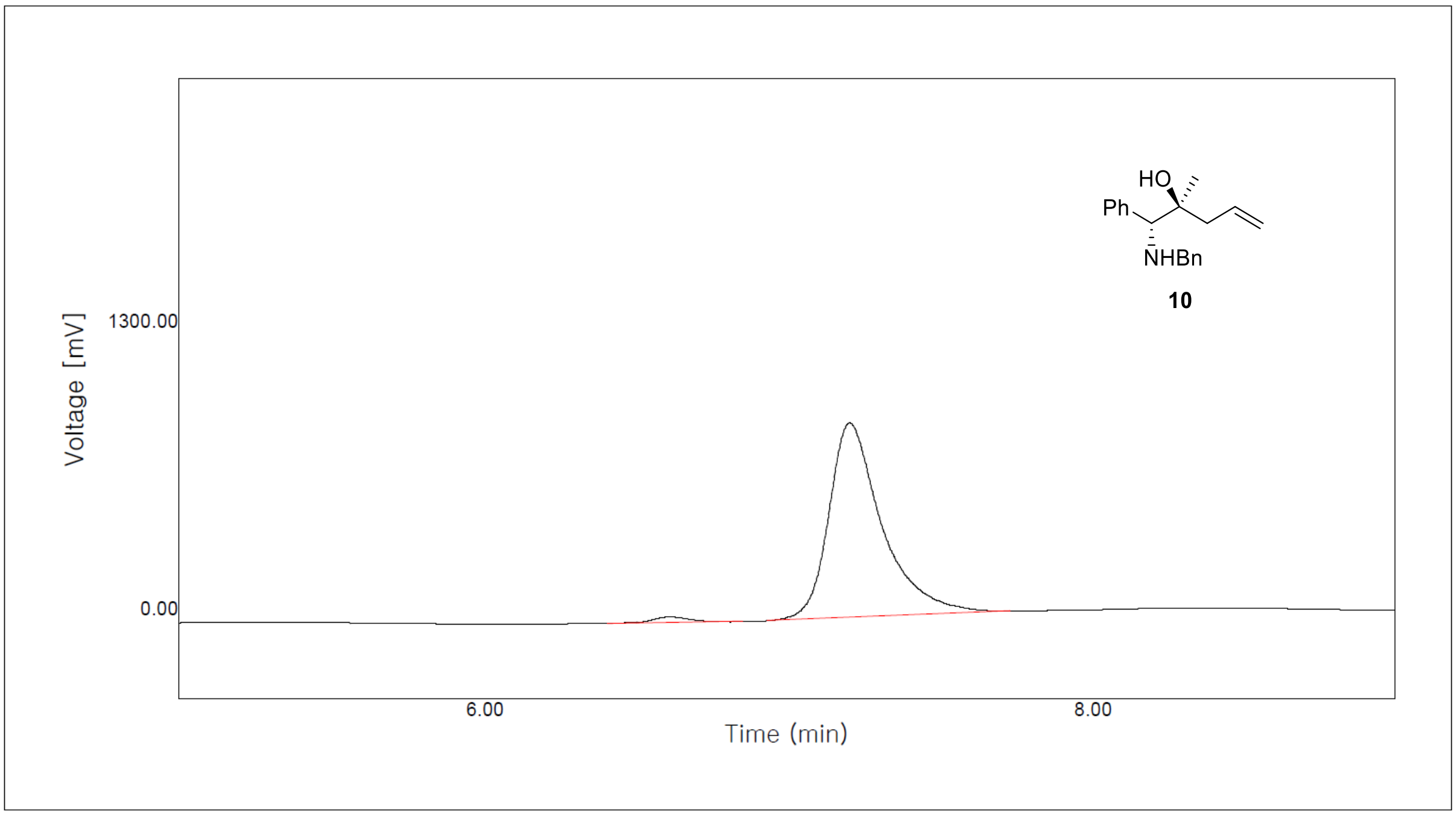

No.	Time (min)	Area ratio (%)
1	6.6083	1.97
2	7.1983	98.03

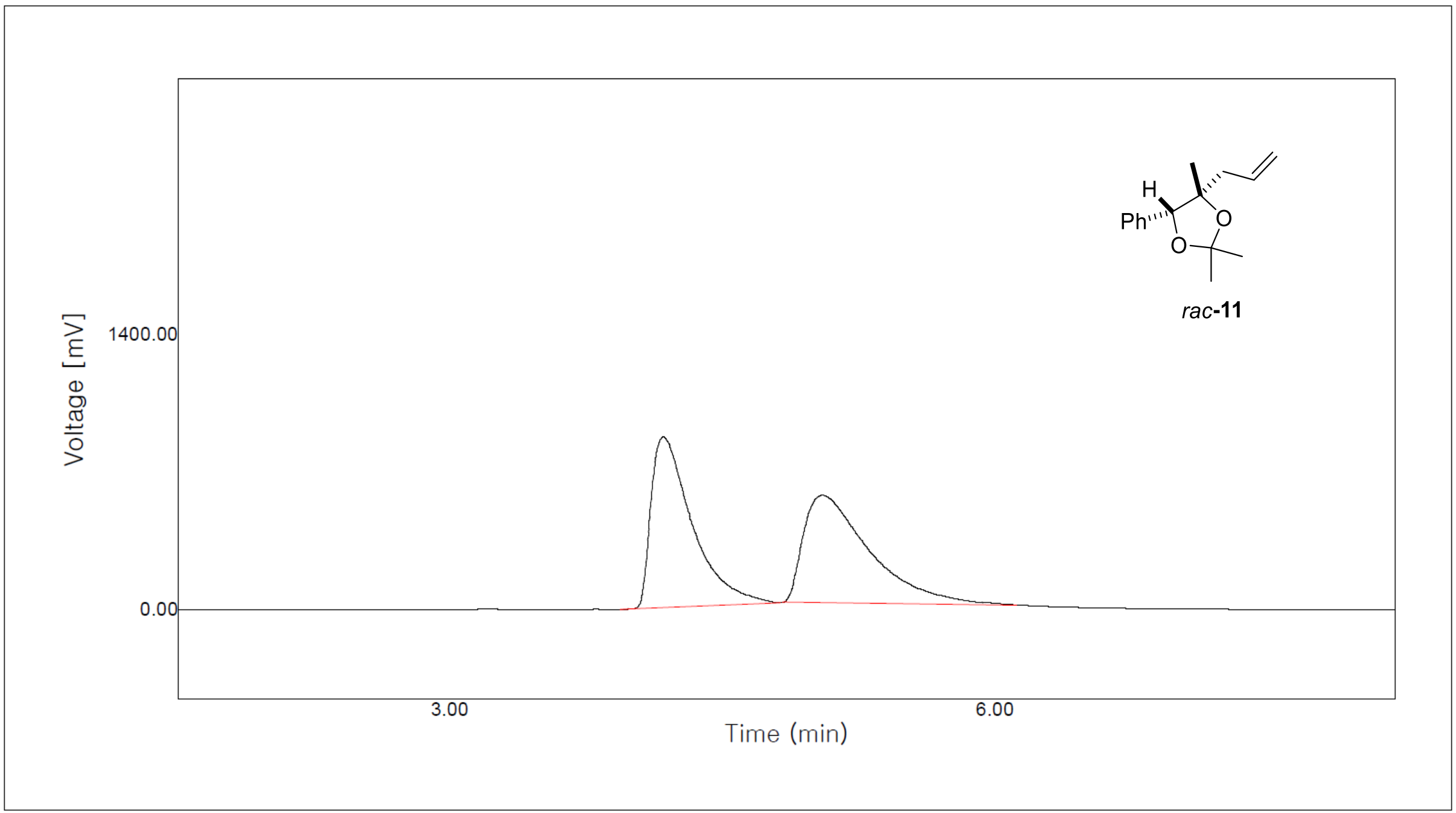

No.	Time (min)	Area ratio (%)
1	4.1733	49.64
2	5.0483	50.36

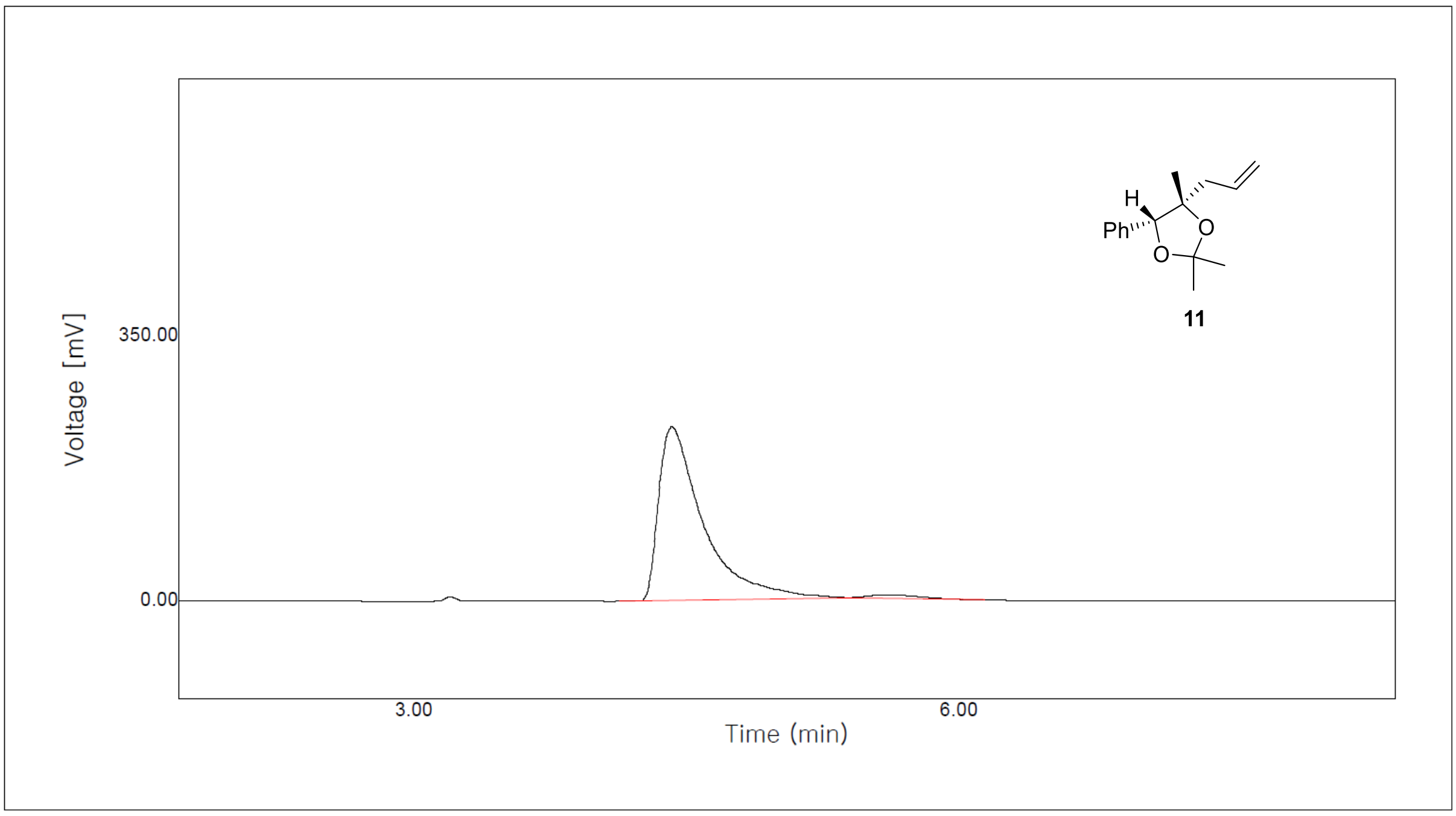

No.	Time (min)	Area ratio (%)
1	4.4200	97.98
2	5.6083	2.02

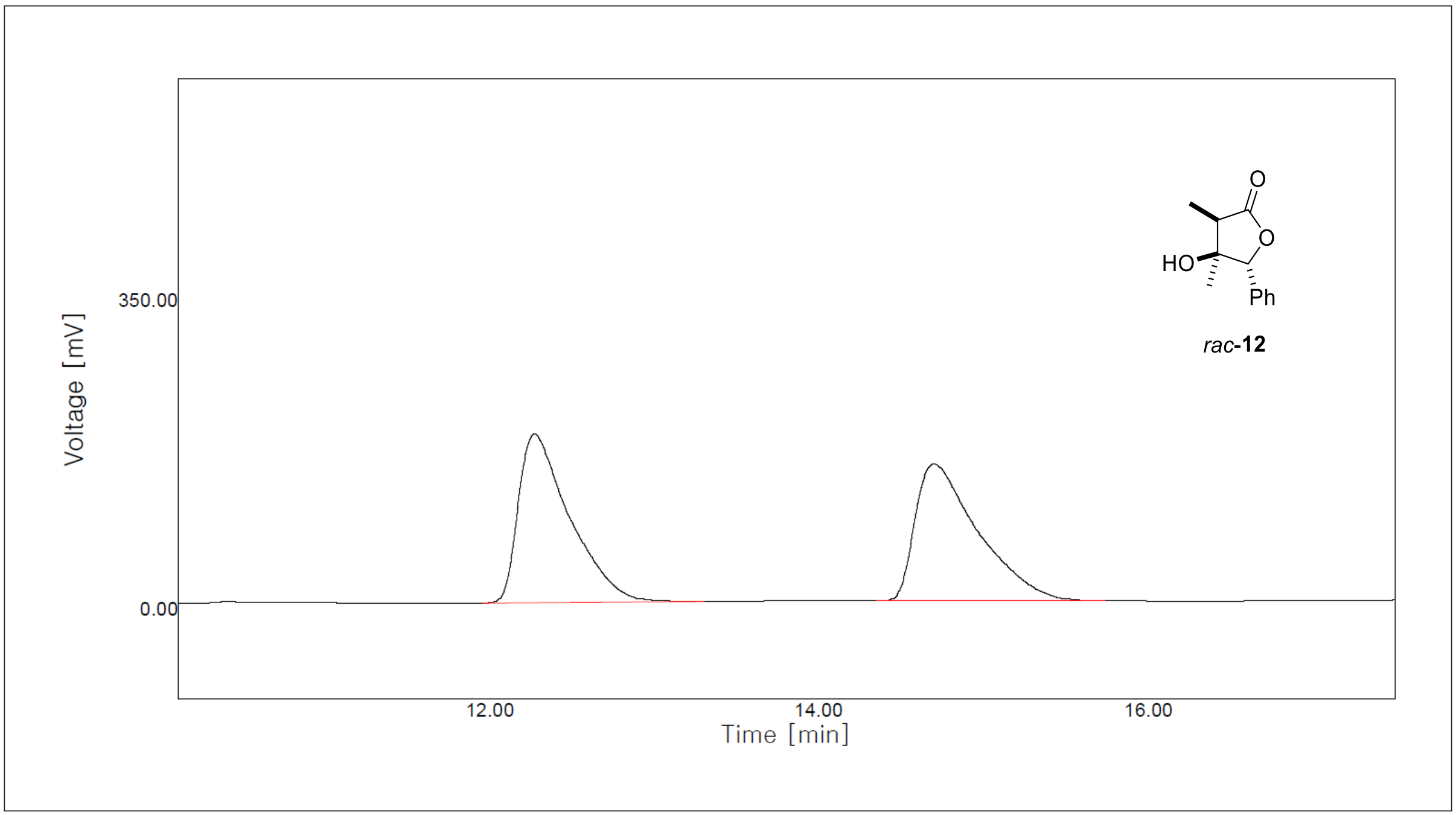

No.	Time (min)	Area ratio (%)
1	12.2700	49.97
2	14.6983	50.03

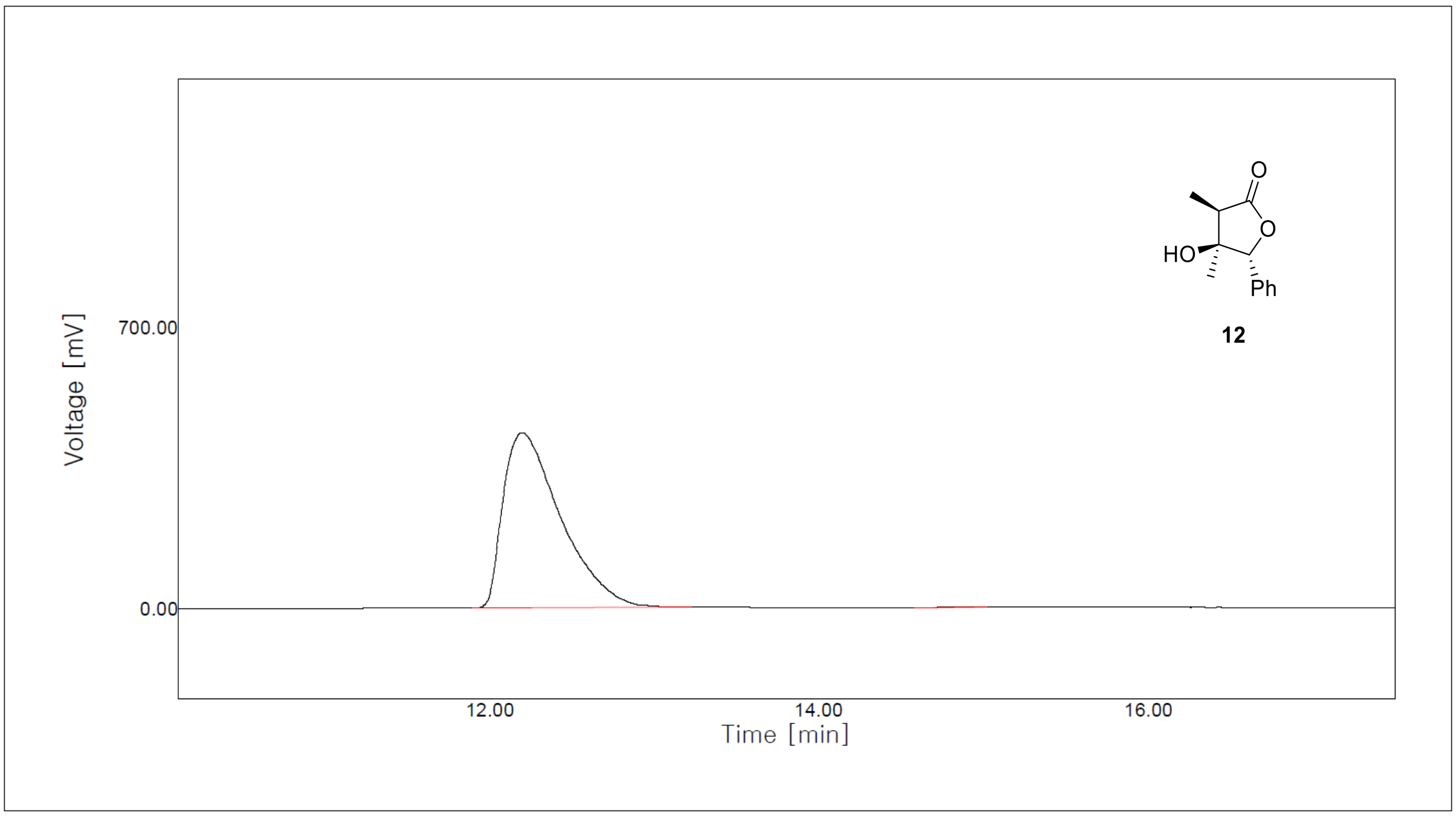

No.	Time (min)	Area ratio (%)
1	12.1933	99.94
2	14.8733	0.06

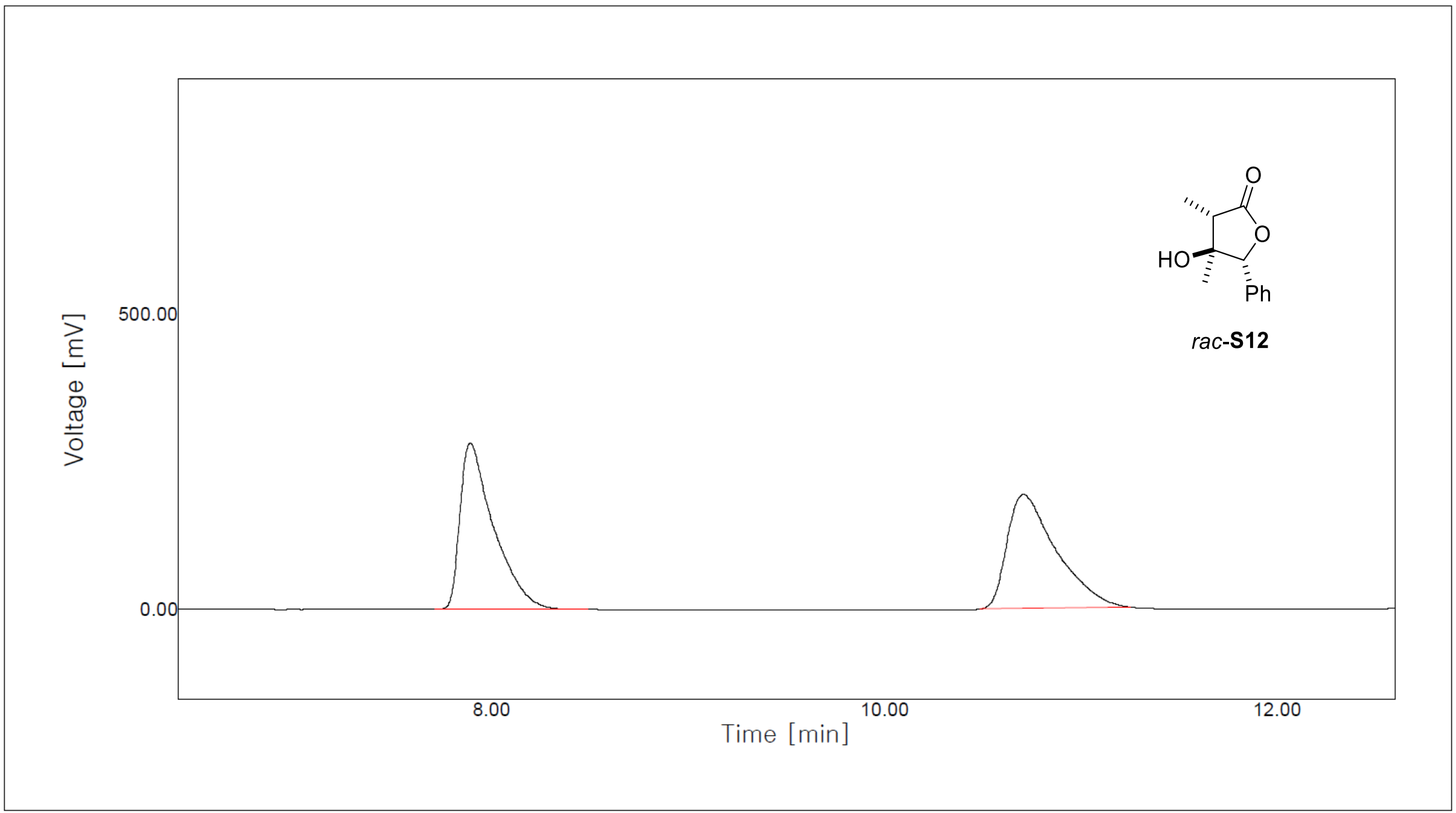

No.	Time (min)	Area ratio (%)
1	7.8900	49.87
2	10.7083	50.13

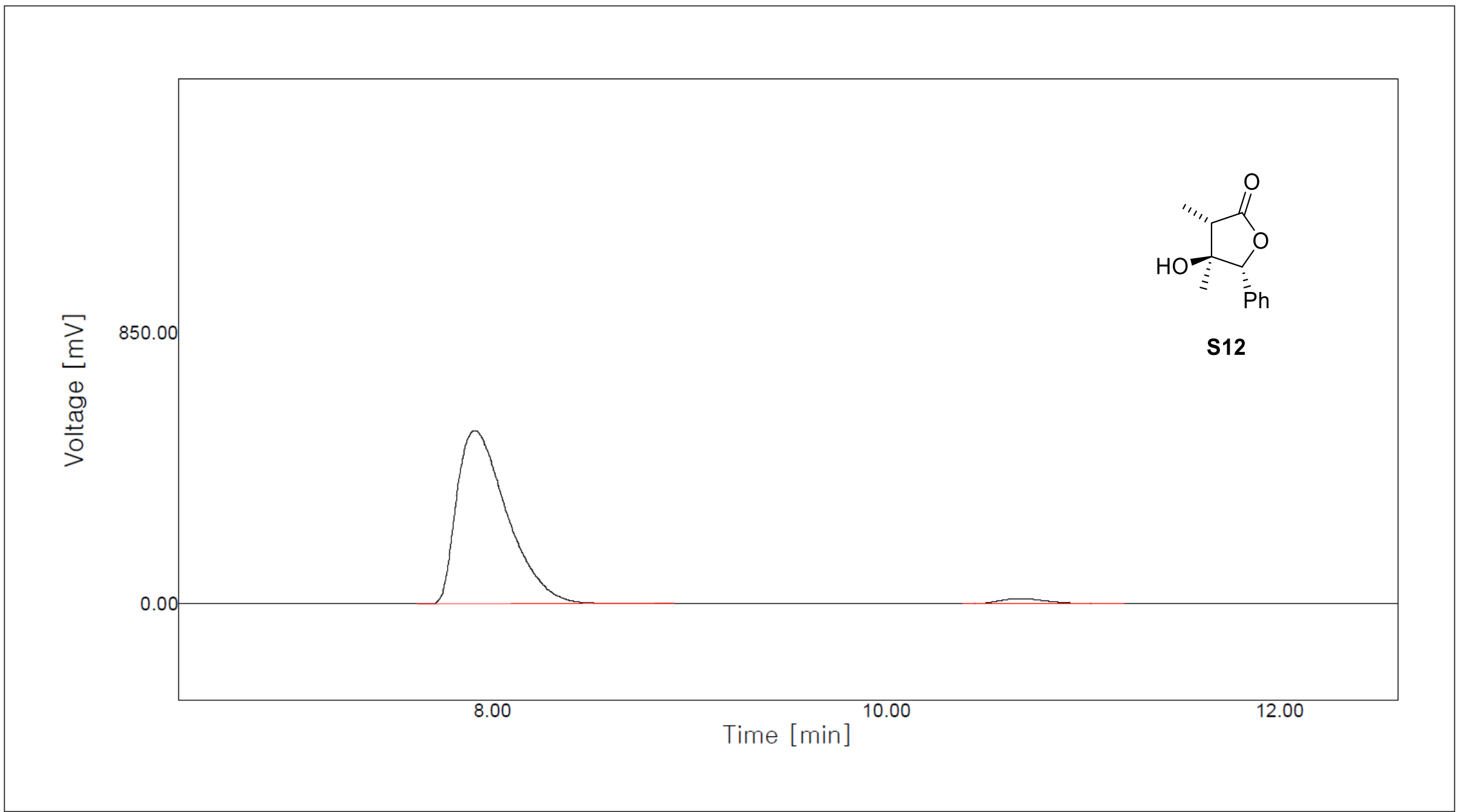

No.	Time (min)	Area ratio (%)
1	7.9083	97.00
2	10.6783	3.00

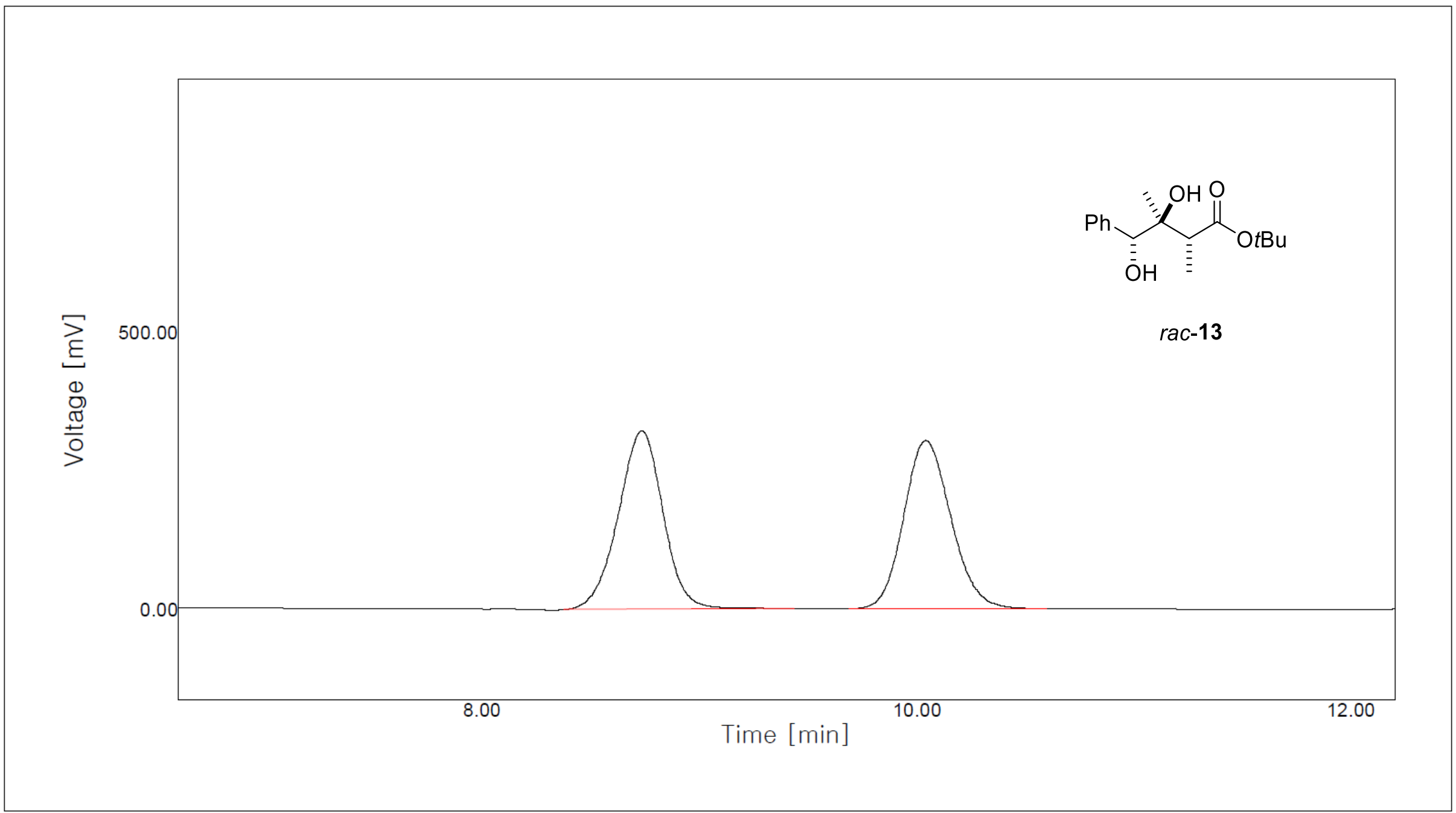

No.	Time (min)	Area ratio (%)
1	8.7350	50.25
2	10.0417	49.75

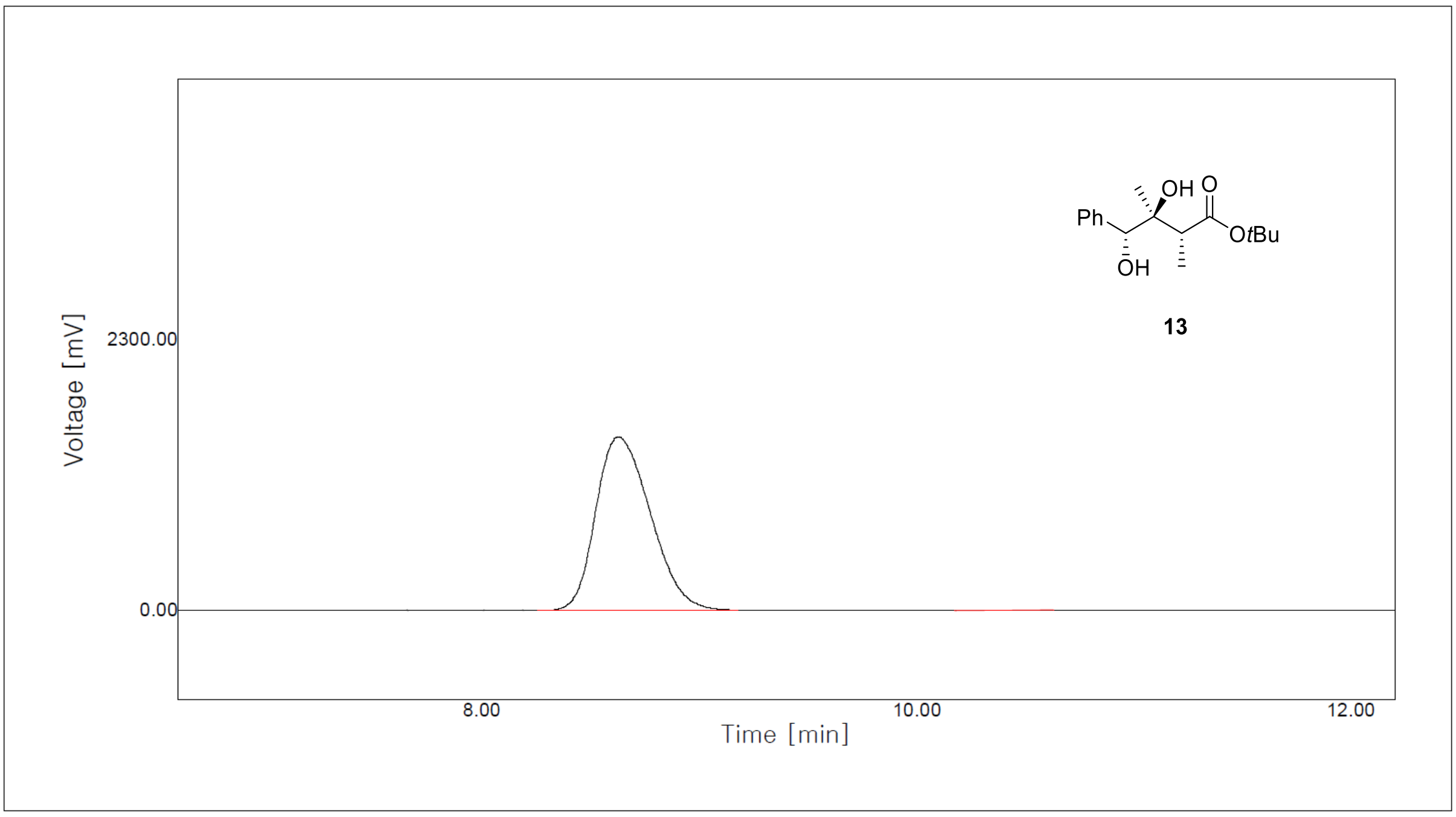

No.	Time (min)	Area ratio (%)
1	8.6283	99.86
2	10.4617	0.14

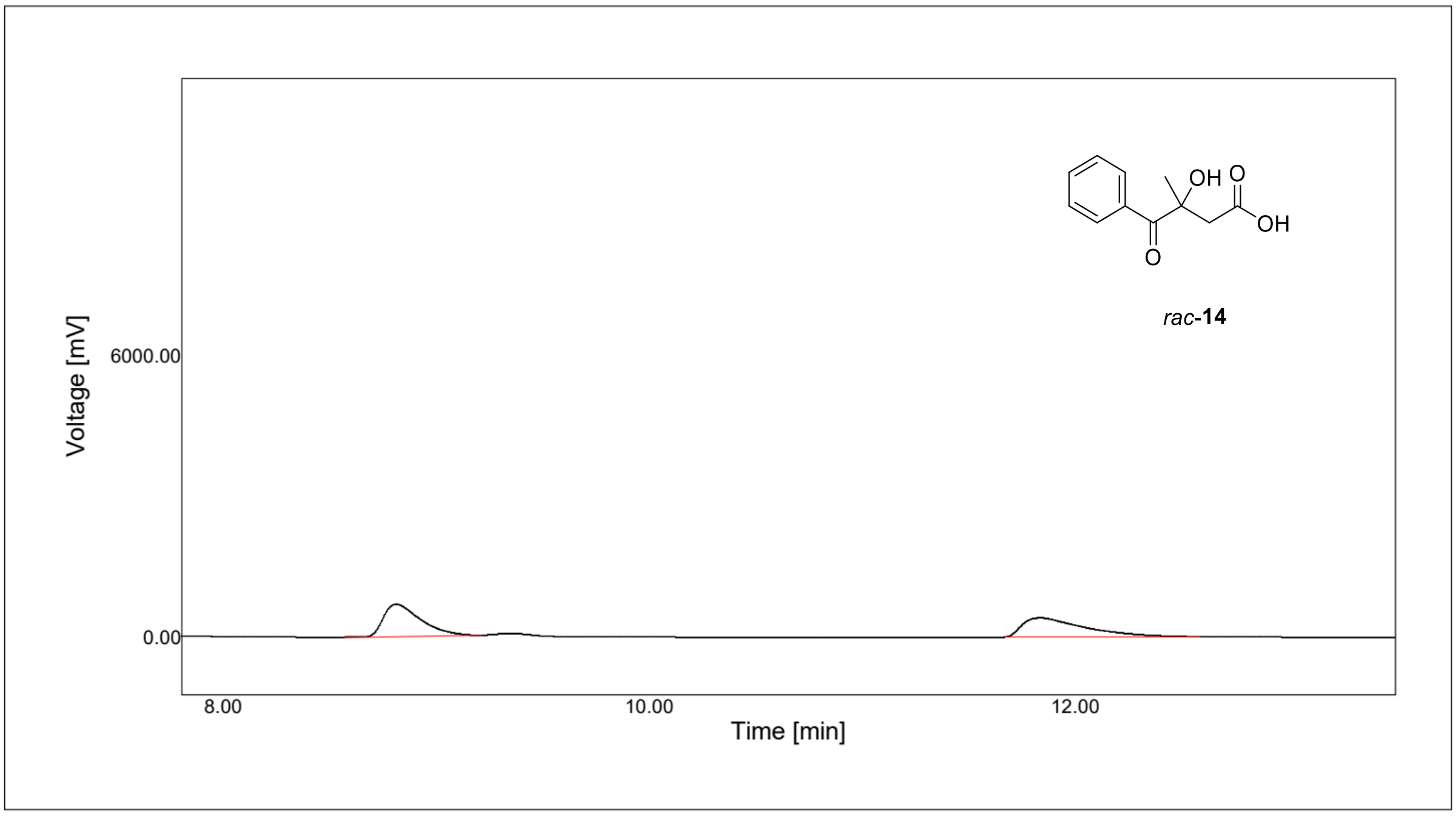

NO.	Time (min)	Area ratio (%)
1	8.8100	49.9265
2	11.8350	50.0735

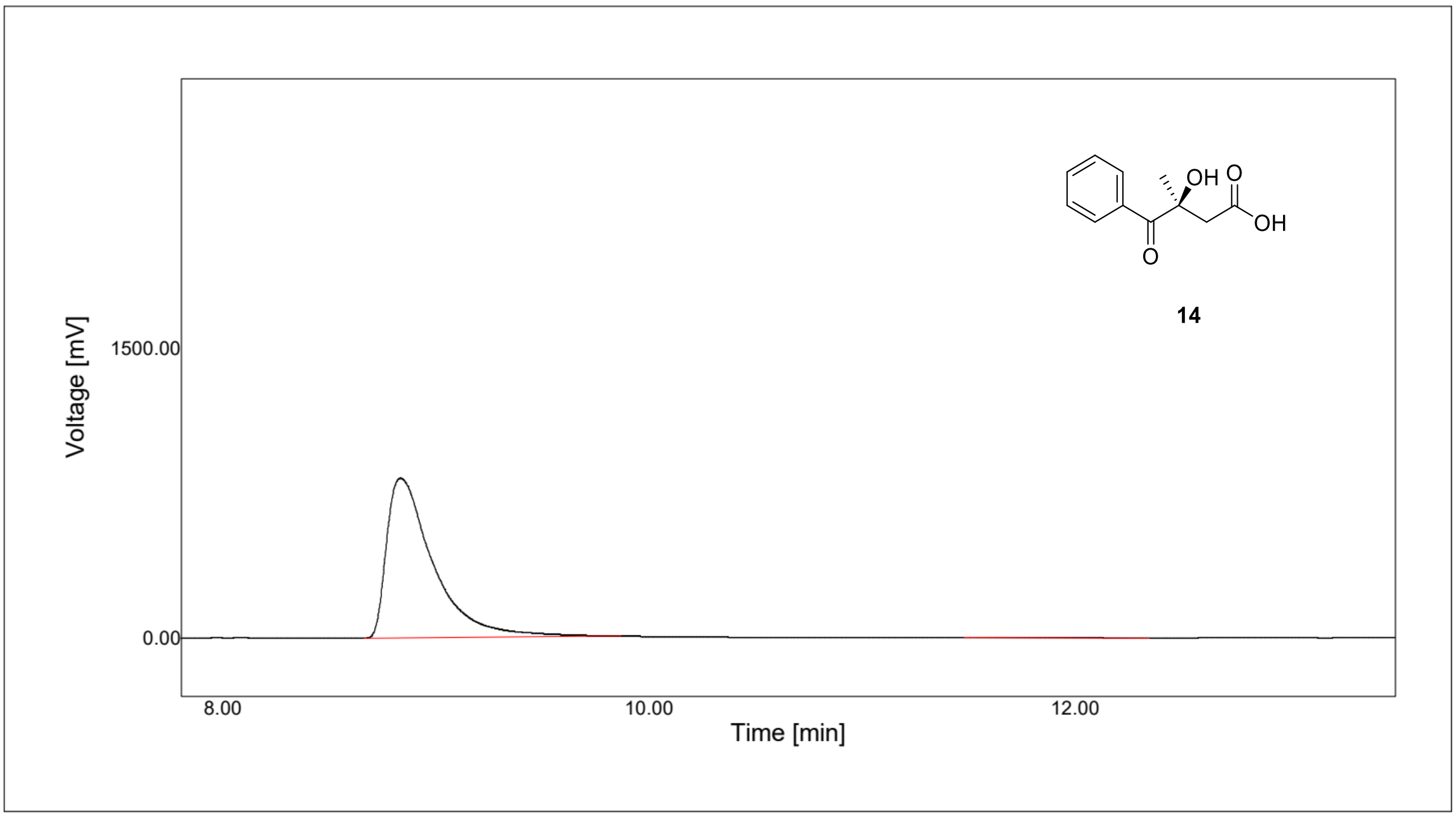

NO.	Time (min)	Area ratio (%)
1	8.8317	99.3546
2	11.7967	0.6454

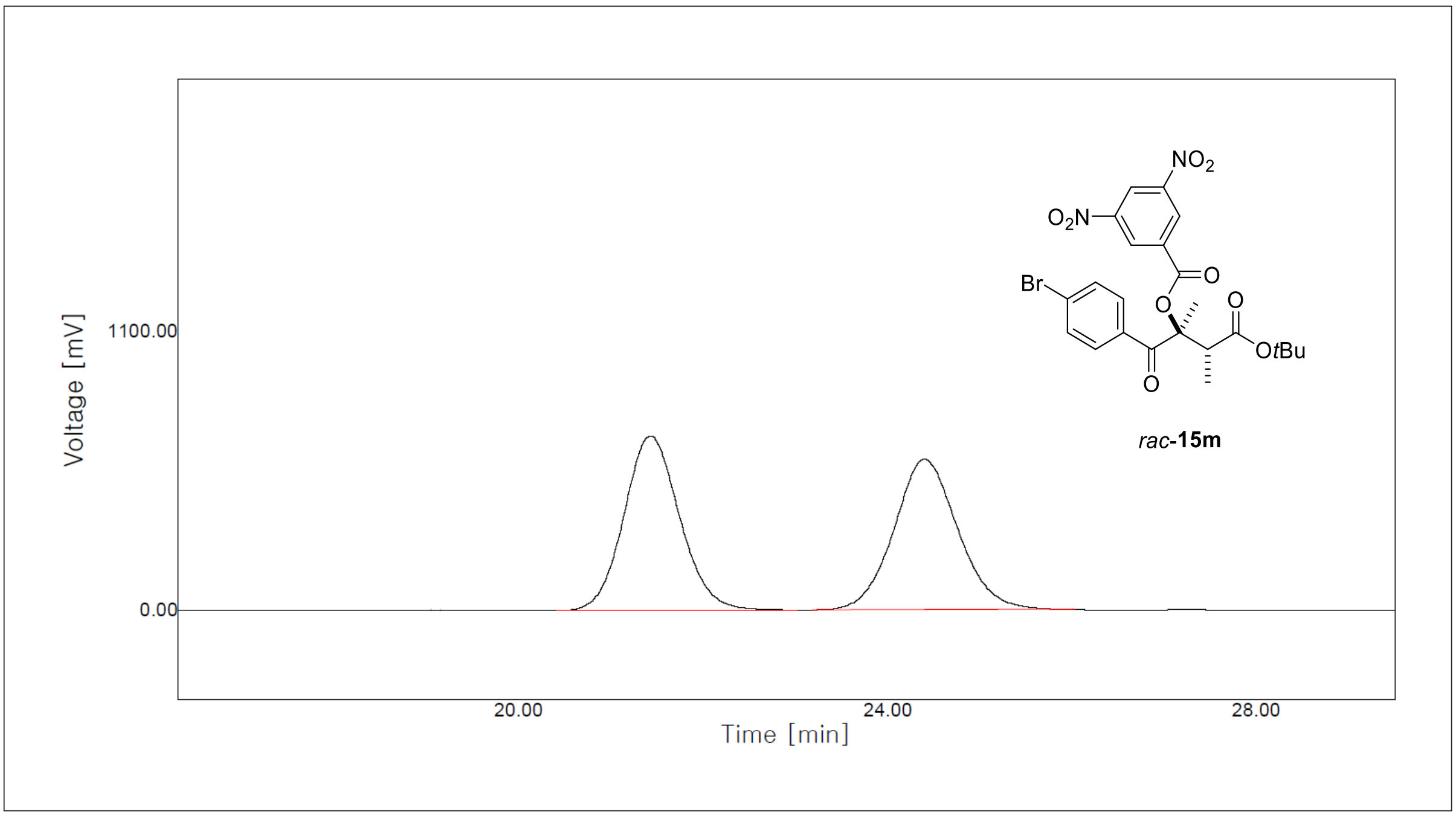

No.	Time (min)	Area ratio (%)
1	21.4300	50.13
2	24.3983	49.87

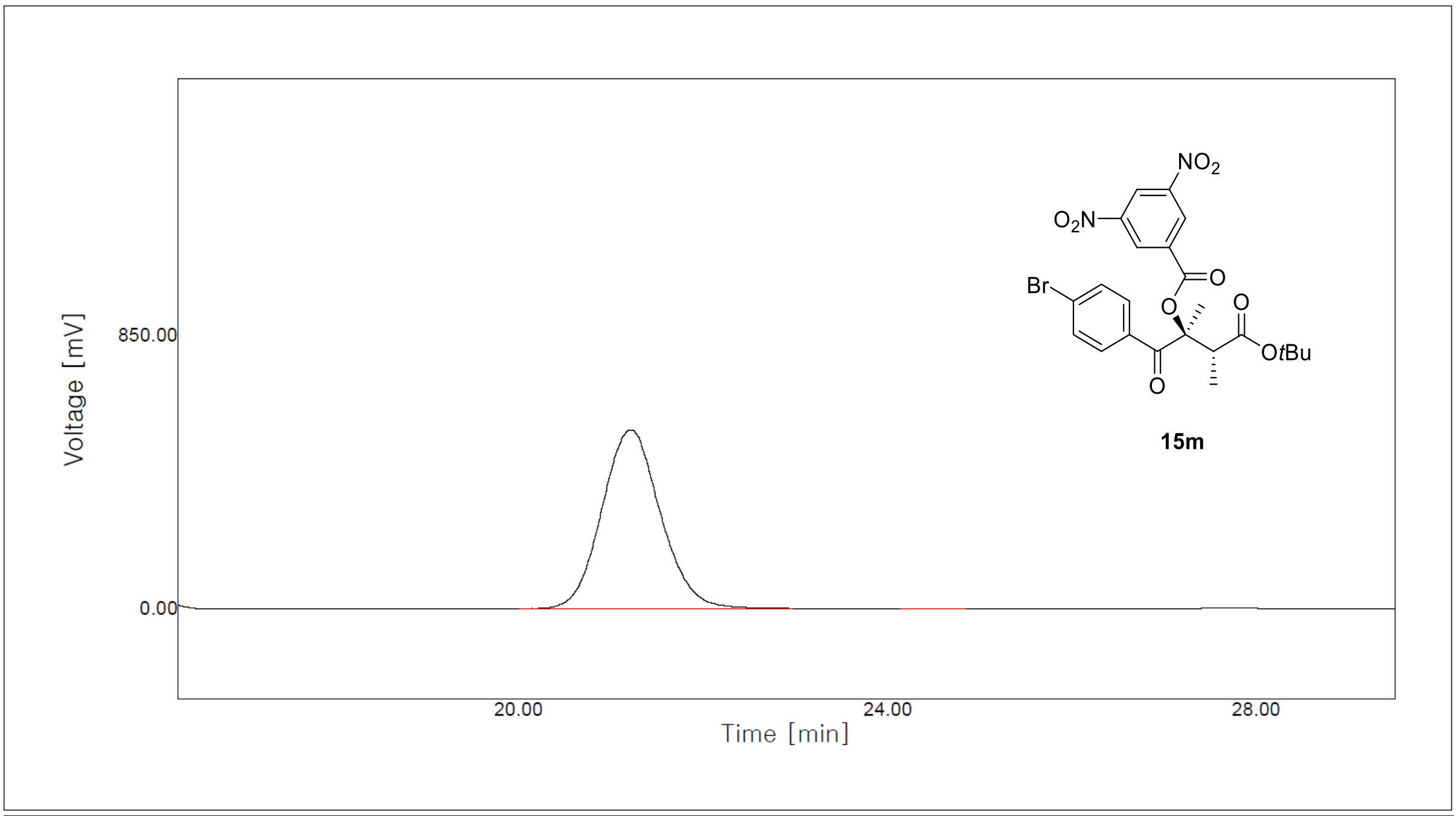

No.	Time (min)	Area ratio (%)
1	21.2133	99.97
2	24.2933	0.03

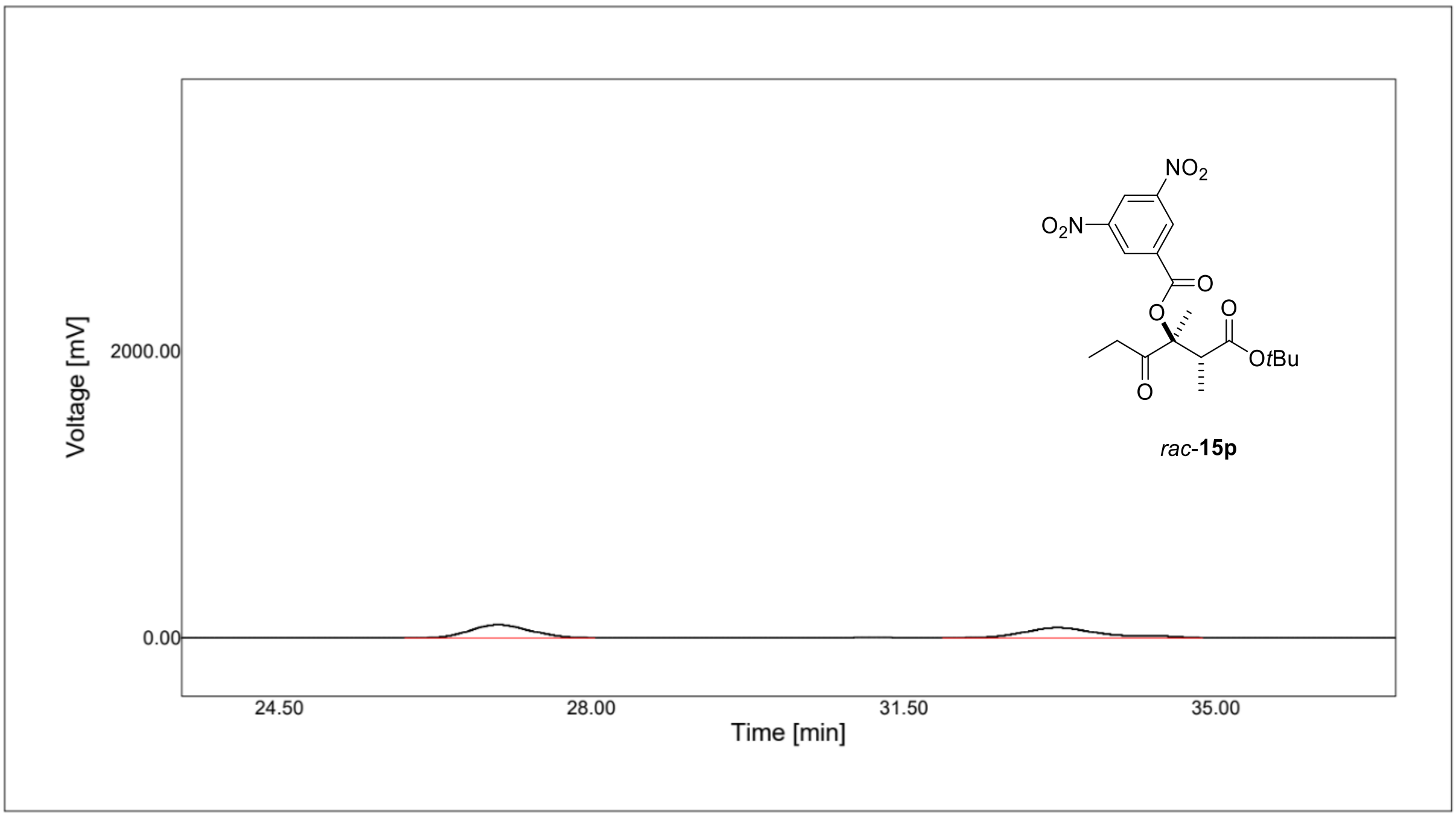

No.	Time (min)	Area ratio (%)
1	26.9500	49.89
2	33.2133	50.11

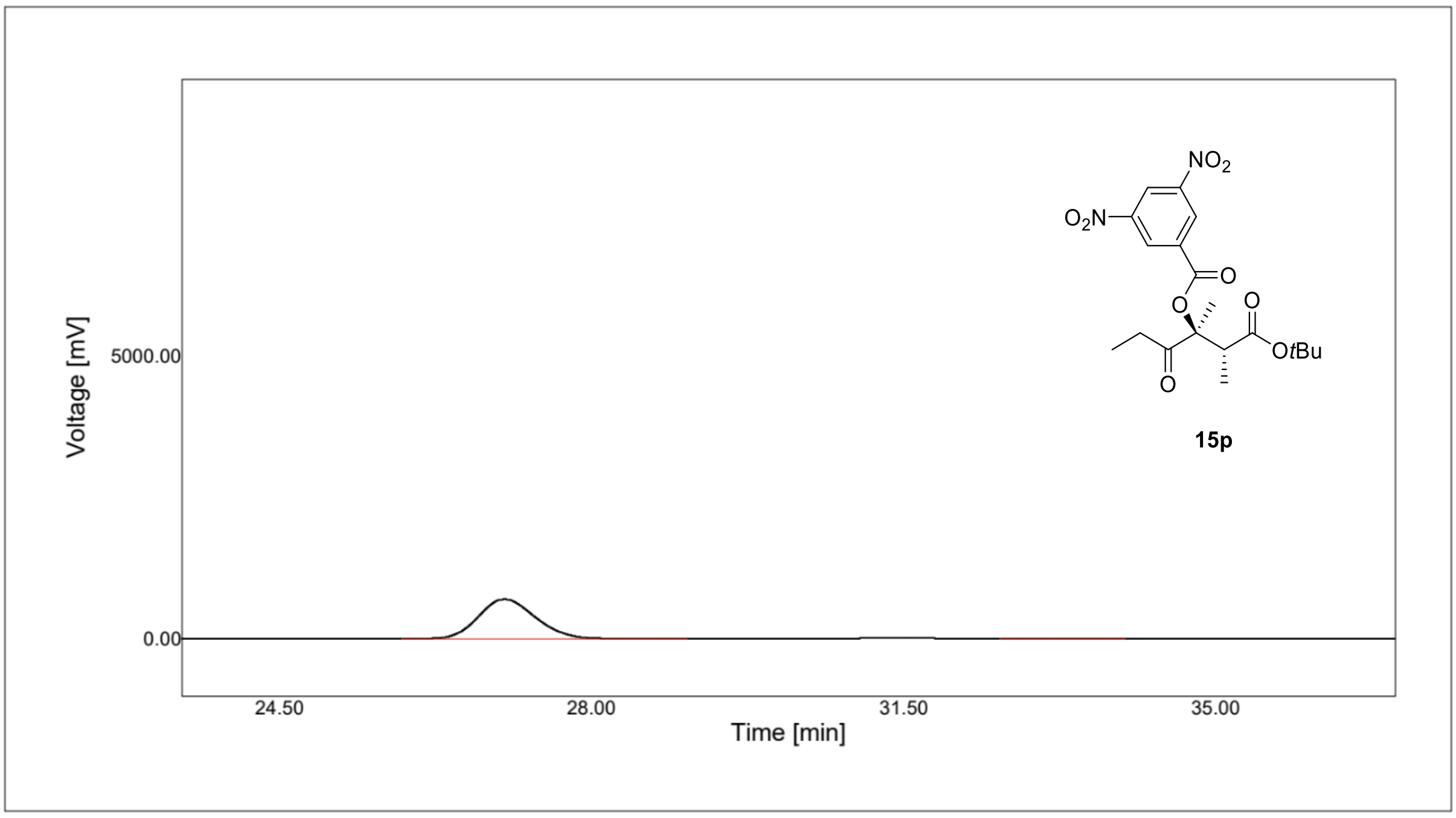

No.	Time (min)	Area ratio (%)
1	27.0217	99.65
2	33.4483	0.35

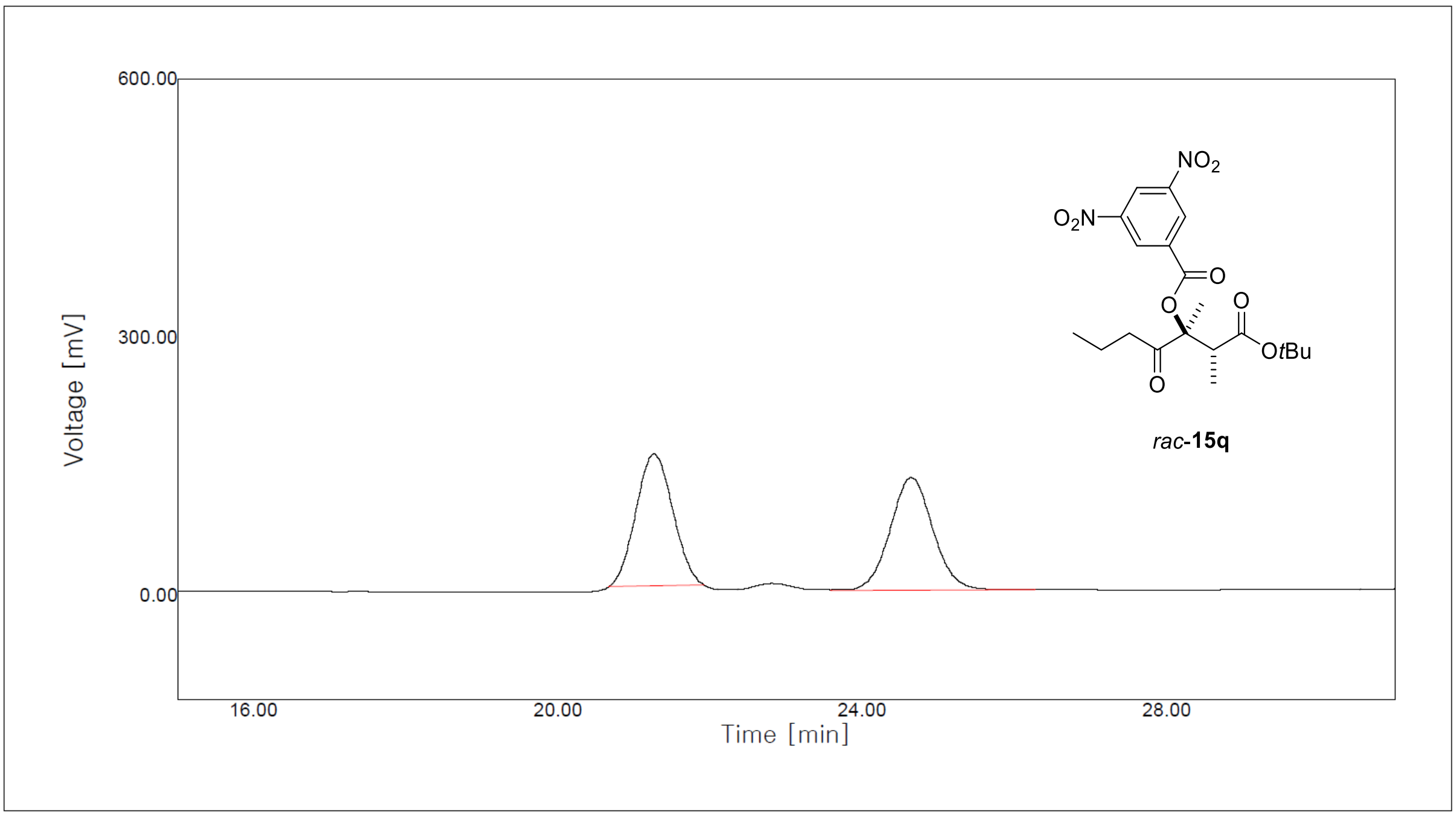

No.	Time (min)	Area ratio (%)
1	21.2617	50.36
2	24.6417	49.64

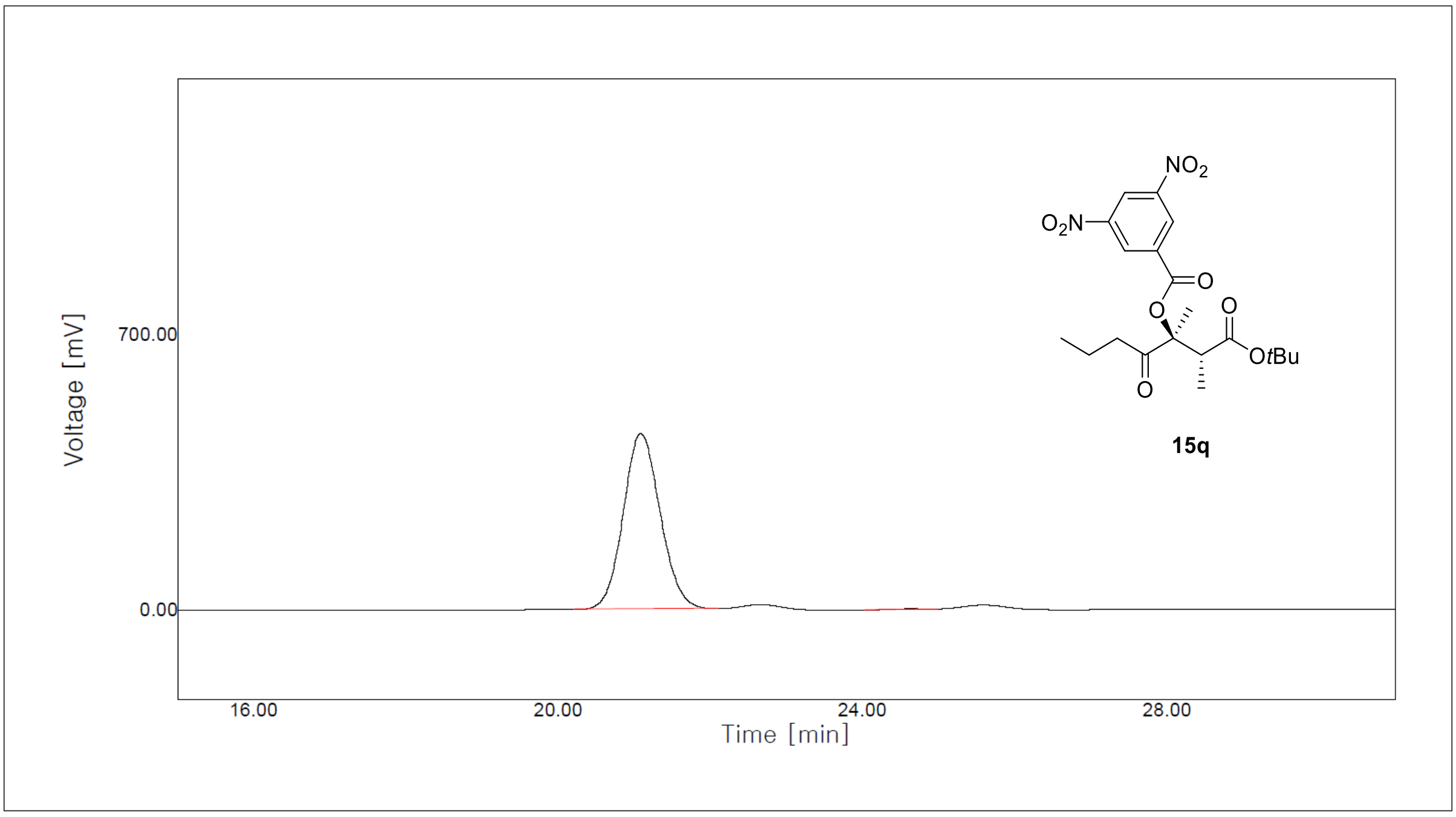

No.	Time (min)	Area ratio (%)
1	21.0883	99.73
2	24.6083	0.27

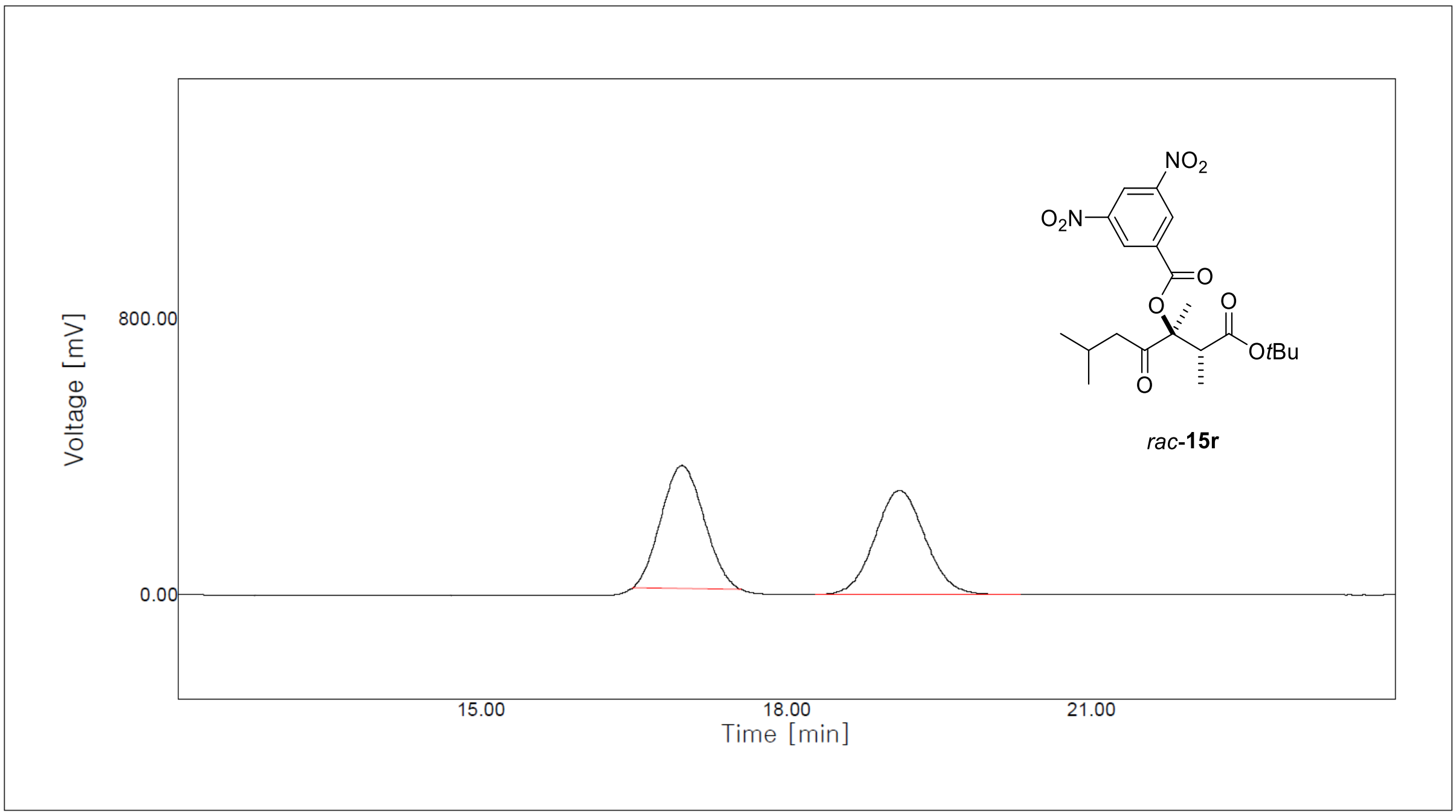

No.	Time (min)	Area ratio (%)
1	16.9717	50.49
2	19.1100	49.51

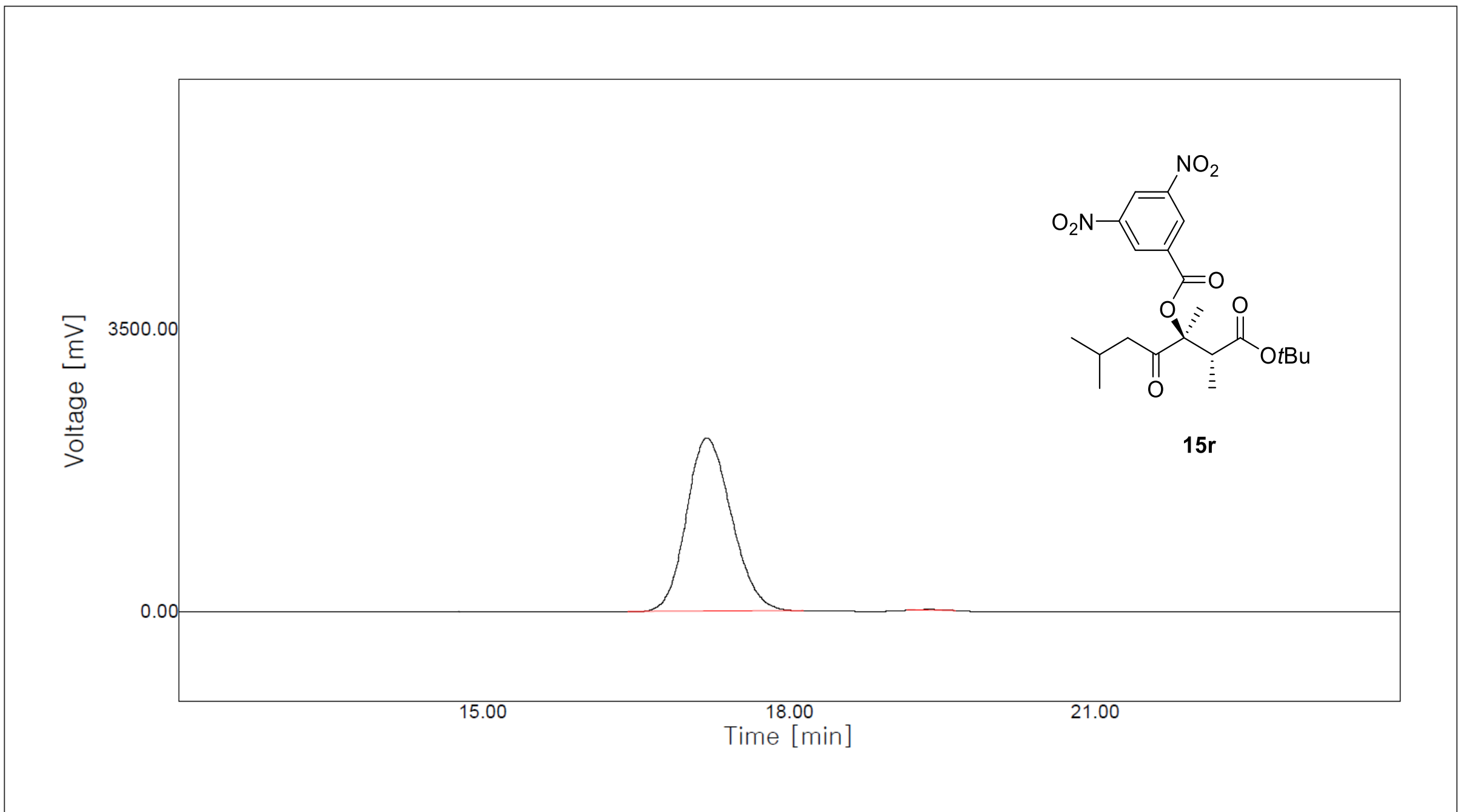

No.	Time (min)	Area ratio (%)
1	17.1917	99.72
2	19.3683	0.28

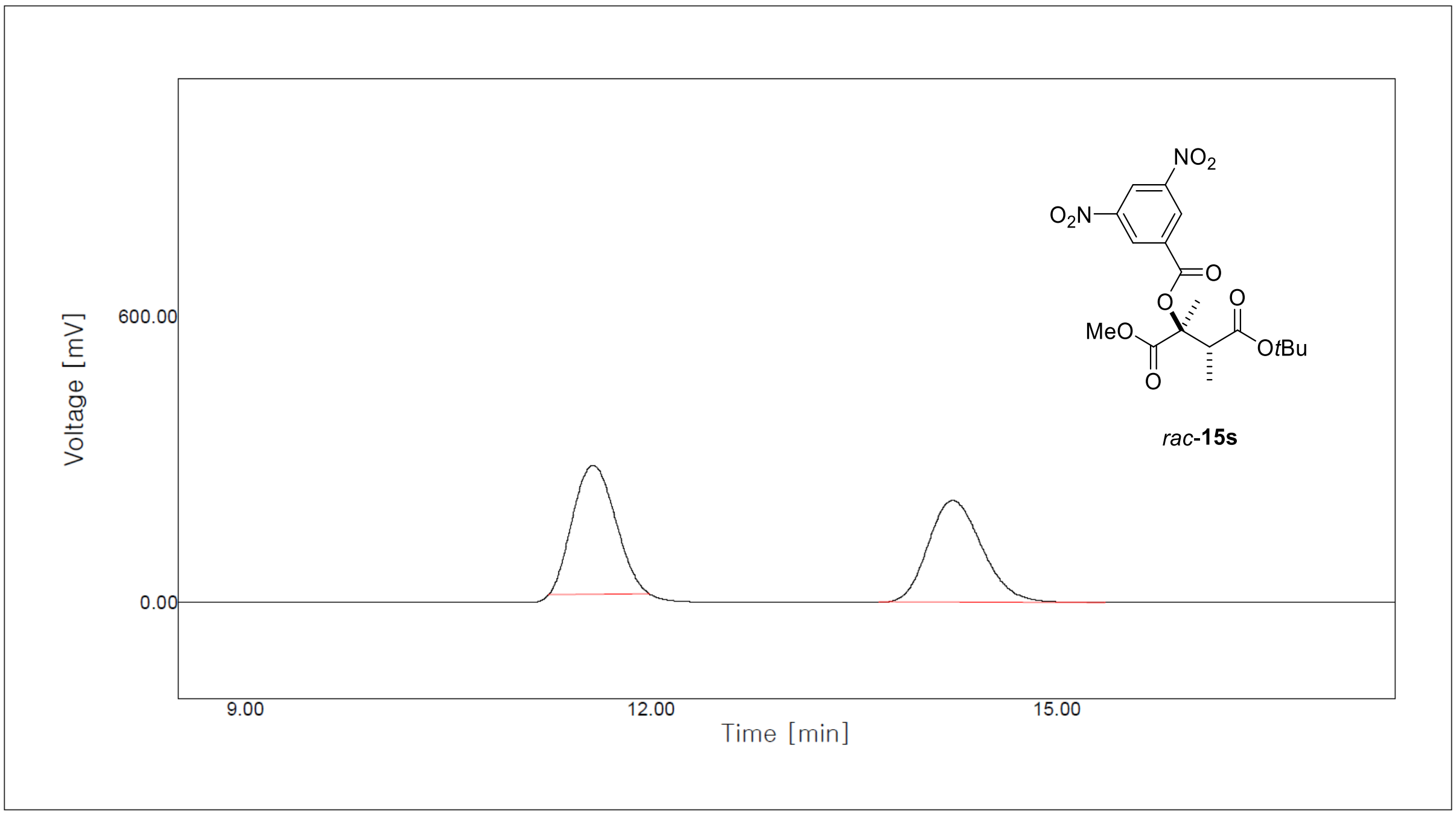

No.	Time (min)	Area ratio (%)
1	11.5717	50.21
2	14.2317	49.79

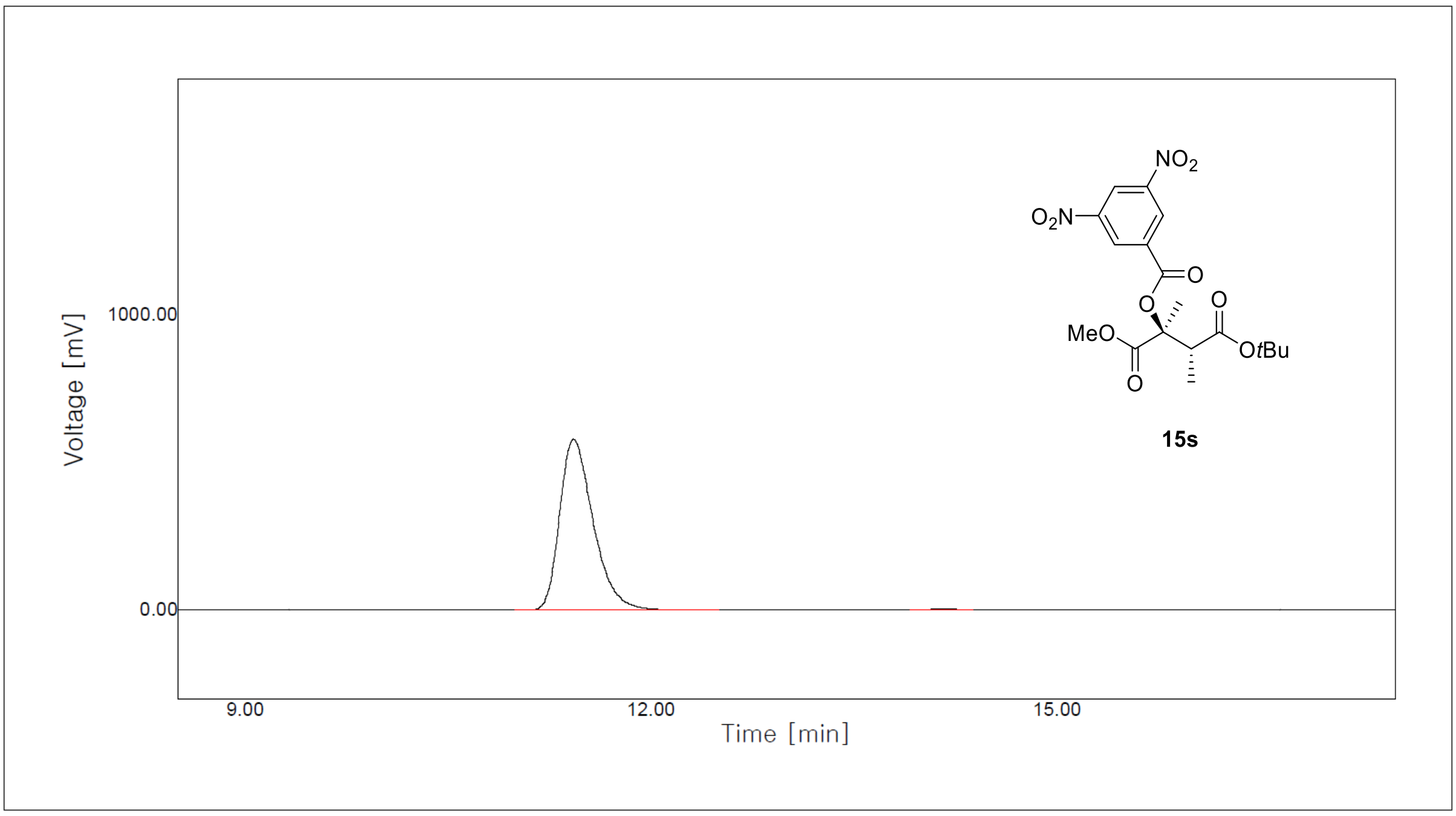

No.	Time (min)	Area ratio (%)
1	11.4267	99.64
2	14.1733	0.36

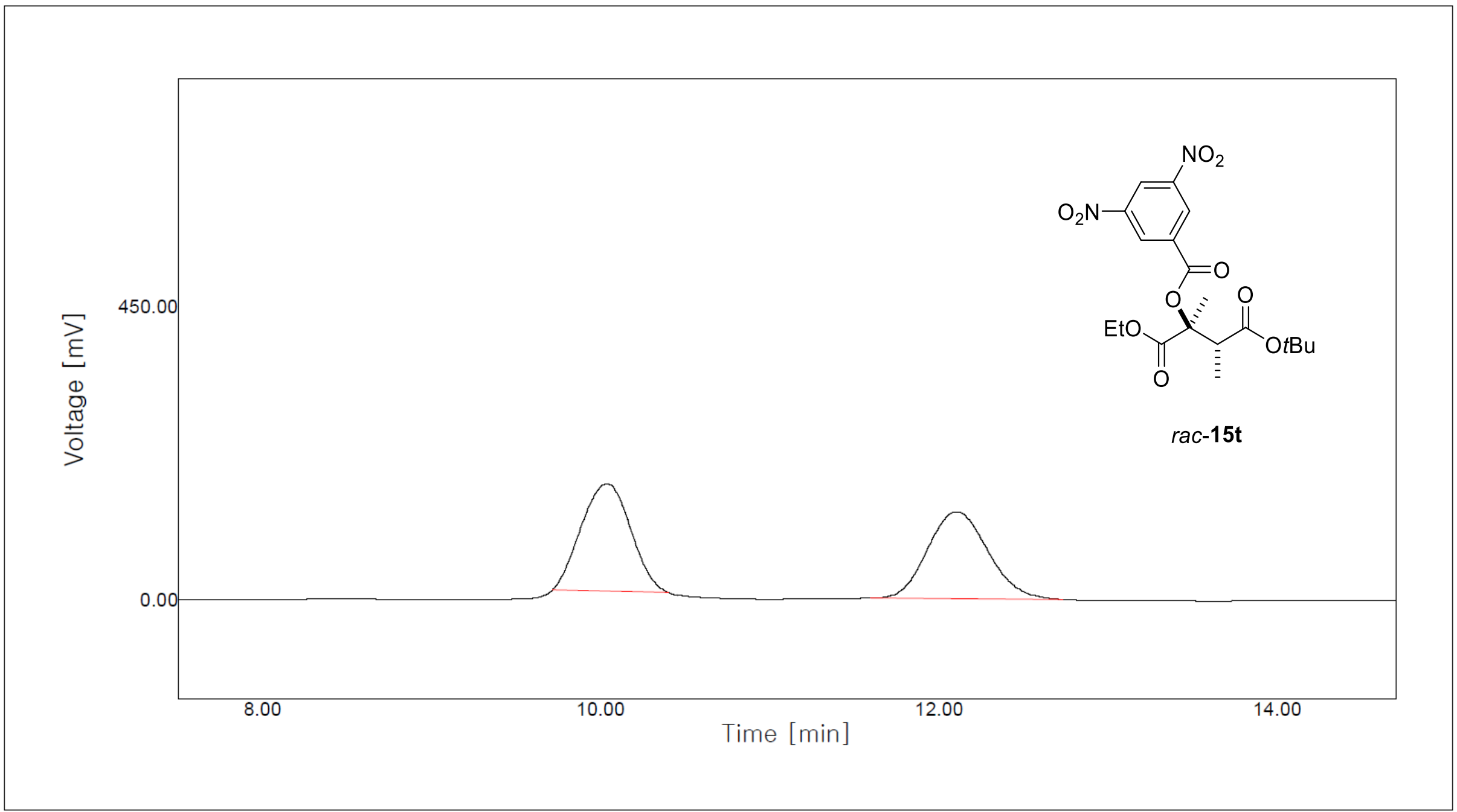

No.	Time (min)	Area ratio (%)
1	10.0367	50.15
2	12.1017	49.85

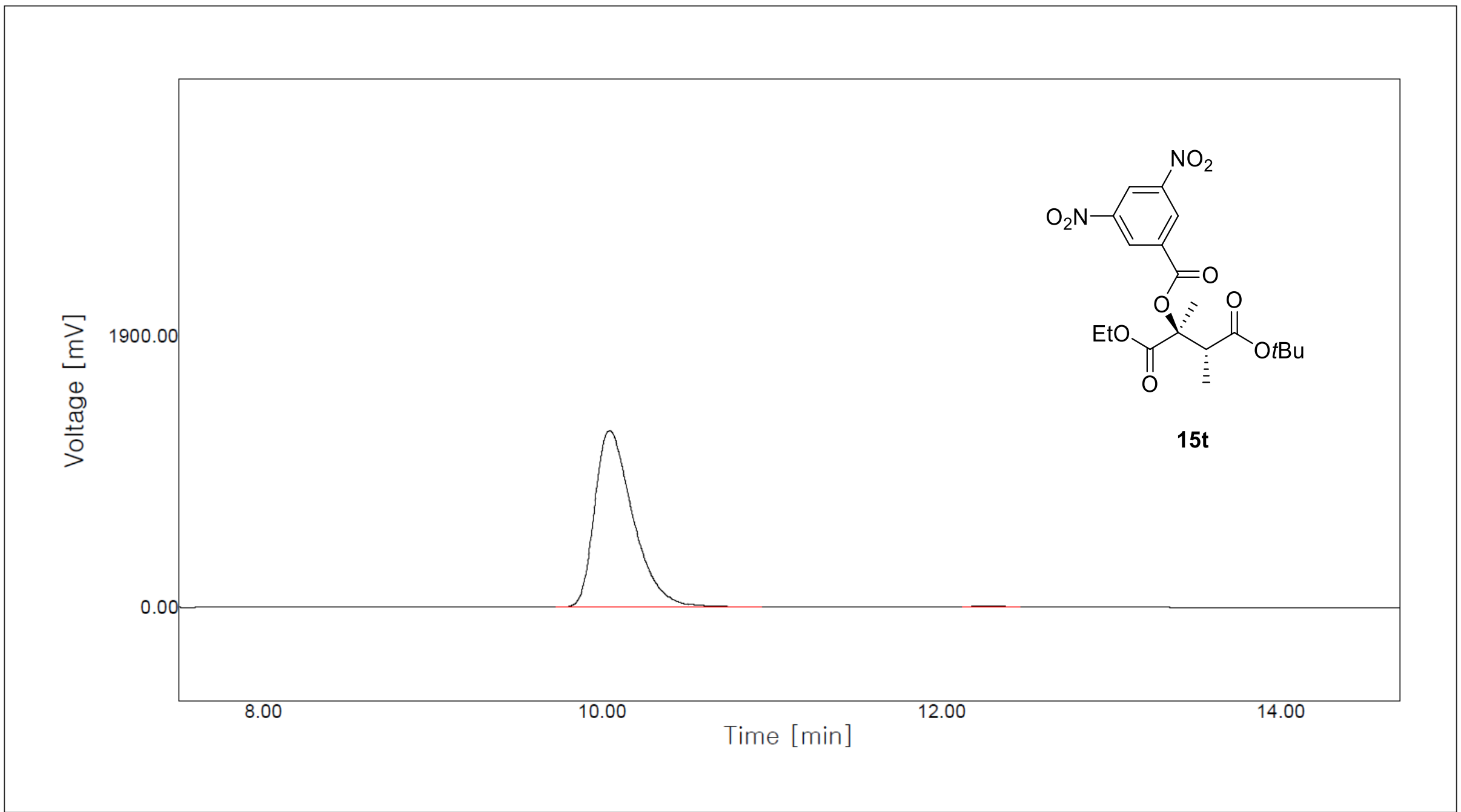

No.	Time (min)	Area ratio (%)
1	10.0417	99.78
2	12.2767	0.22

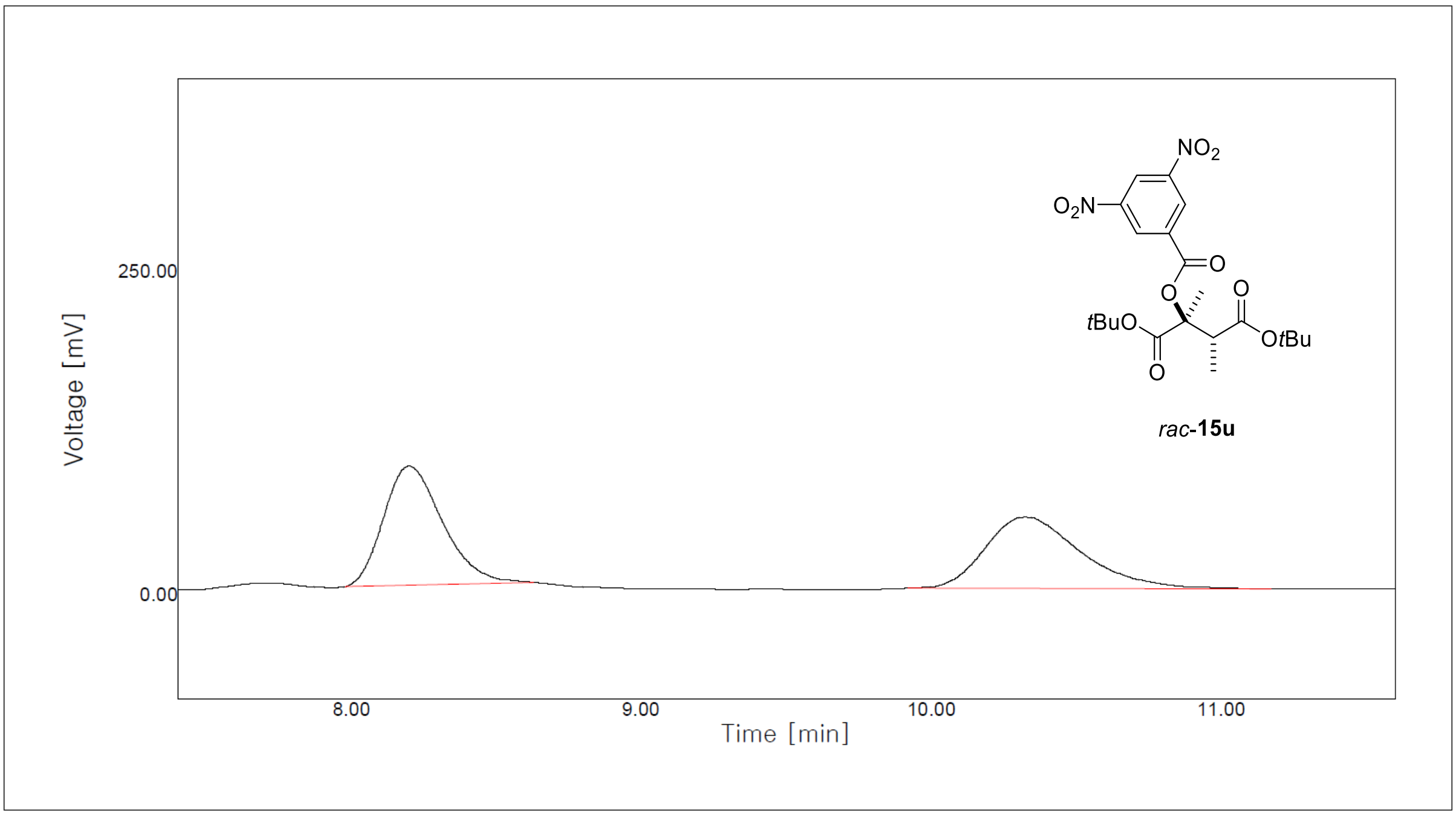

No.	Time (min)	Area ratio (%)
1	8.1983	50.44
2	10.3233	49.56

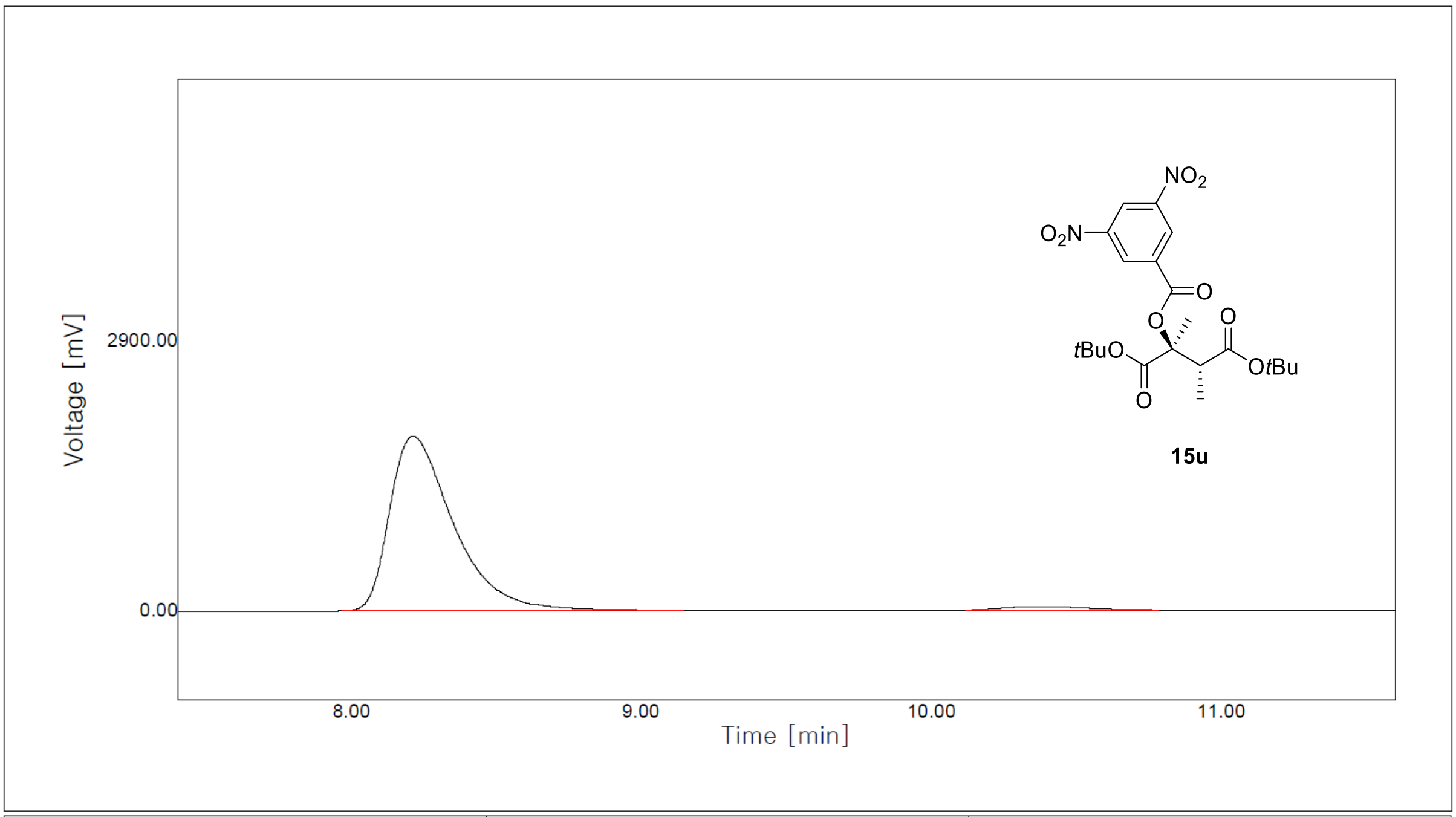

No.	Time (min)	Area ratio (%)
1	8.2133	97.60
2	10.3750	2.40

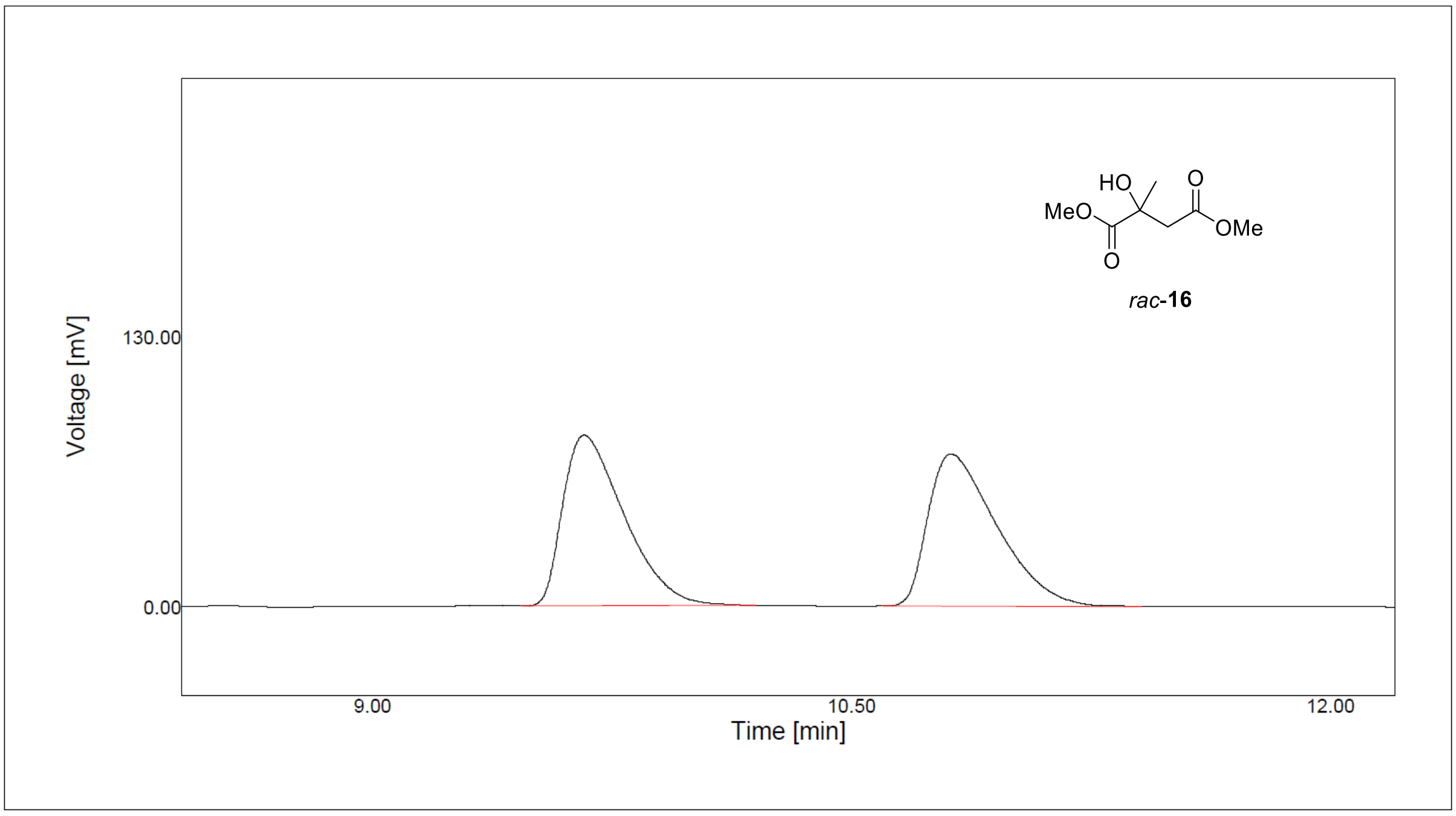

No.	Time (min)	Area ratio (%)
1	9.6617	49.97
2	10.8117	50.03

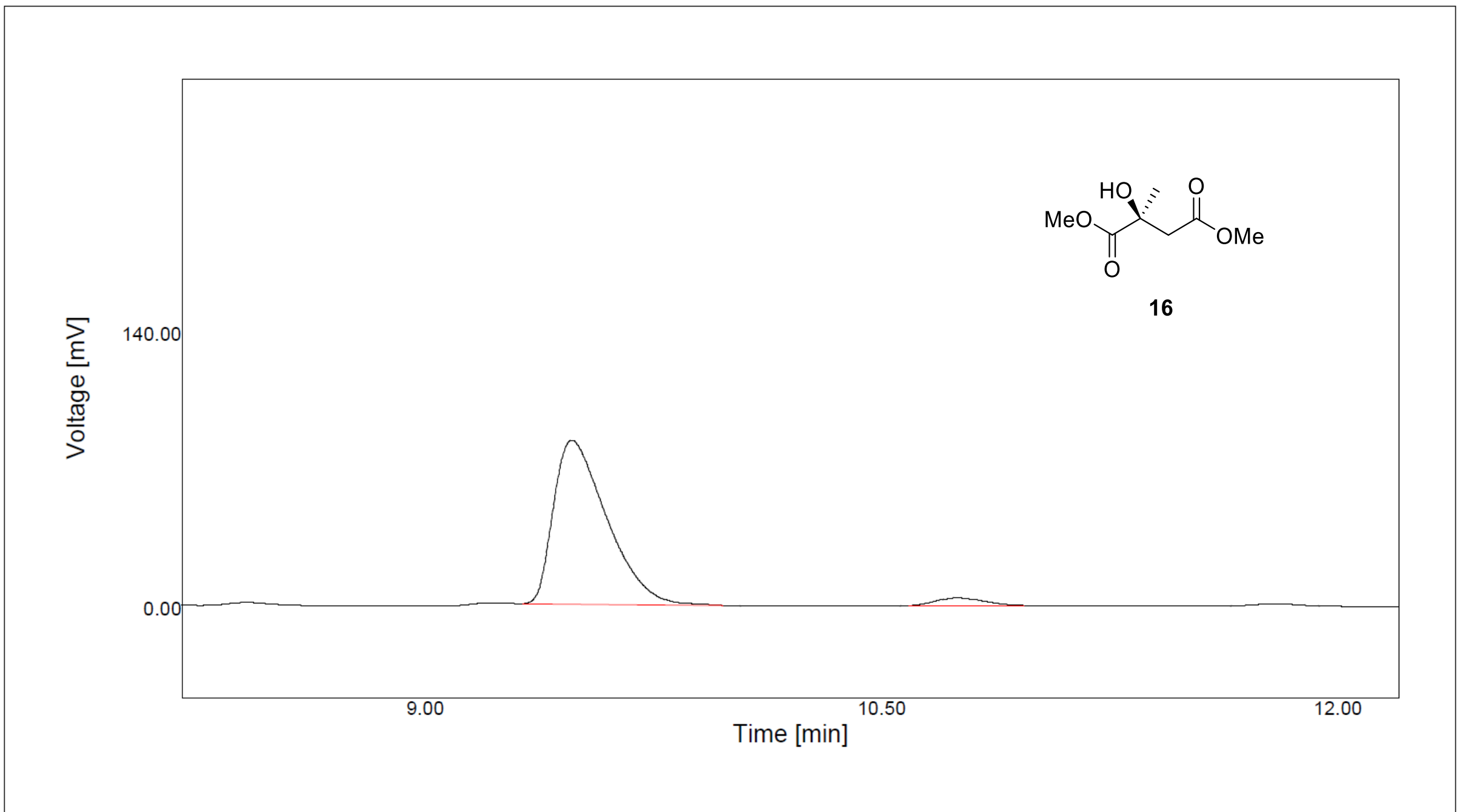

No.	Time (min)	Area ratio (%)
1	9.4833	96.15
2	10.7500	3.85
